# Supplementary material for: Regio- and enantioselective remote hydroarylation using a ligand-relay strategy
Source: Nat Commun. 2022 May 5;13:2471. doi: 10.1038/s41467-022-30006-2 (PMC9072428; doi:10.1038/s41467-022-30006-2)
Supplement: Supplementary file 1 — SUPPLEMENTARY INFORMATION [file 41467_2022_30006_MOESM1_ESM.pdf]

Supplementary Information

Regio- and enantioselective remote hydroarylation using a ligand-relay strategy

Yuli He[1], Jiawei Ma[1], Huayue Song[1], Yao Zhang[1], Yong Liang[1,*], You Wang[1,*], and Shaolin Zhu[1,2,*]

[1]*State Key Laboratory of Coordination Chemistry, Jiangsu Key Laboratory of Advanced Organic Materials, Chemistry and Biomedicine Innovation Center (ChemBIC), School of Chemistry and Chemical Engineering, Nanjing University, Nanjing, 210093, China*

[2]*School of Chemistry and Chemical Engineering, Henan Normal University, Xinxiang, 453007, China*

*email: yongliang@nju.edu.cn; wangyou@nju.edu.cn; shaolinzhu@nju.edu.cn

1. Supplementary Notes

Solvents were either purified and dried by passage through alumina and Q5 reactant-packed columns on a solvent purification system or bought from the commercial sources and transferred to the glovebox without exposure to air. Other commercial reagents were purchased from Sigma-Aldrich, Acros, Alfa Aesar, TCI, J&K, Energy Chemical, Bide Pharmatech Ltd. and were used as received. Flash chromatography was either performed using glass columns with *SiliaFlash*® P60 (SiliCycle, 230-400 mesh), or on pre-packed *Biotage*® SNAP columns using a Biotage Isolera Automated Flash Chromatography System.

$NiCl_2$·dme: (CAS 29046-78-4) was purchased from Strem Chemical;

4-Phenyl-1-butene: (CAS 768-56-9) was purchased from TCI and stored under nitrogen at –20 °C in glove box;

2,9-Dimethyl-10-phenanthroline: (Neocuproine, CAS 484-11-7) was purchased from Adamas-beta®;

DMMS: (Methyldimethoxysilane, CAS 16881-77-9) was purchased from TCI and stored under nitrogen at –20 °C in glove box;

KF: (*White powder*, CAS 7789-23-3) was purchased from Alfa Aesar (Stock No. 42216);

DMPU: [1,3-Dimethyl-3,4,5,6-tetrahydro-2(1*H*)-pyrimidinone, CAS 7226-23-5] was purchased from TCI and stored under nitrogen in glove box;

All compounds (starting materials and products) were characterized by ^{1}H NMR, ^{13}C NMR, IR spectroscopy, melting point (where applicable), and high-resolution mass spectrometry. ^{1}H NMR spectra were recorded on Bruker 500 M MHz spectrometer and are referenced relative to residual $CDCl_3$ proton signals at δ 7.26 ppm. ^{19}F NMR spectra were recorded on a Bruker 500 MHz spectrometer and are referenced to $CFCl_3$ (δ 0.0 ppm). Data for ^{1}H and ^{19}F NMR are reported as follows: chemical shift (δ ppm), multiplicity (s = singlet, d = doublet, t = triplet, q = quartet, m = multiplet, ap = apparent), integration, and coupling constant (Hz). ^{13}C NMR spectra were recorded on a Bruker 500 MHz spectrometer and are referenced to $CDCl_3$ at δ 77.16 ppm. The ^{13}C NMR spectra were obtained with ^{1}H decoupling. Data for ^{13}C NMR are reported in terms of chemical

shift and multiplicity where appropriate. IR spectra were obtained on a Bruker Alpha and was reported in terms of frequency of absorption (cm^{-1}). GC analyses were performed on an Agilent 8890 gas chromatograph with an FID detector using a J&W DB-1 column (10 m, 0.1 mm I.D.). High Resolution Mass spectra were obtained on a Bruker Daltonics, Inc. APEXIII 7.0 TESLA FTMS instrument (ESI) or Atmospheric Pressure Chemical Ionization (ESI) mode. High pressure liquid chromatography (HPLC) was performed on Agilent 1260 Series chromatographs using Daicel Chiralcel columns (250 mm). Optical rotations were measured on a S3 Rudolph Research Analytical Autopol VI automatic polarimeter using a 50 mm pathlength cell at 589 nm with $[\alpha]_D$ values reported in degrees; concentration (c) is in g/100 mL. Melting points (m.p.) were obtained on a Mel-Temp capillary melting point apparatus. The powder X-ray diffraction pattern (PXRD) measurements were carried out on a Philips X'pert MPD Pro *X*-ray diffractometer using Cu Kα radiation (λ = 0.15418 nm), and the *X*-ray tube was operated at 40 kV and 40 mA at room temperature. Reactions were monitored by GC analysis and thin-layer chromatography (TLC) carried out on 0.25 mm Jiang you silica gel plates (HSGF254) using UV light as a visualizing agent.

2. Supplementary Methods

2.1. Regio- and Enantioselective C(sp^3)−H Arylation

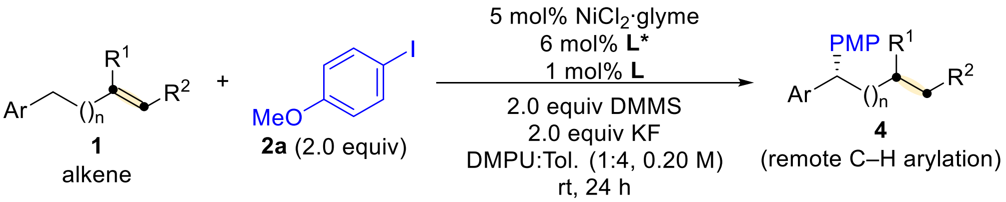

General procedure (A) for the regio- and enantioselective C(sp^3)−H arylation. In a nitrogen-filled glove box, to an oven-dried 8 mL screw-cap vial equipped with a magnetic stir bar was added $NiCl_2$·glyme (2.2 mg, 5.0 mol%), **L*** (7.2 mg, 6.0 mol%), KF (23.2 mg, 2.0 equiv), **L** [0.42 mg, 0.2 mL (2.1 mg/ mL in toluene)], anhydrous toluene (0.60 mL) and DMPU (0.20 mL) were added, and the mixture was stirred for 15 min at room temperature, at which time 4-phenyl-1-butene (30 µL, 0.20 mmol), 4-iodoanisole (94.0 mg, 0.40 mmol) and DMMS (49.3 µL, 0.40 mmol) were added to the resulting mixture in this order. The tube was sealed with a teflon-lined screw cap, removed from the glove box and the reaction was stirred at rt (22~26 °C) for up to 24 h (the mixture was stirred at 750 rpm, ensuring that the base was uniformly suspended). After the reaction was complete, the reaction mixture was directly filtered through a short pad of silica gel [EtOAc in petroleum ether (PE)] to give the crude product. Dodecane (20 µL) was added as an internal standard for GC analysis. The product was purified by chromatography on silica gel for each substrate. The yields reported are the average of at least two experiments, unless otherwise indicated. The enantiomeric excesses (% *ee*) were determined by HPLC analysis using chiral stationary phases.

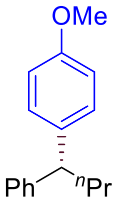

(*R*)-1-Methoxy-4-(1-phenylpropyl)benzene (Figure 3, **3a**)[1,2]. From **4-Iodoanisole** (94.0 mg, 0.40 mmol, 2.0 equiv), the title compound was prepared following the general

procedure **A** using $NiCl_2$·glyme (2.2 mg, 5.0 mol%), **L*** (7.2 mg, 6.0 mol%), **L** [0.42 mg, 0.2 mL (2.1 mg/mL in toluene)], KF (23.2 mg, 2.0 equiv), 4-phenyl-1-butene (**1a**) (30.0 μL, 0.20 mmol), DMMS (49.3 μL, 0.40 mmol), anhydrous toluene (0.60 mL) and DMPU (0.20 mL). The reaction mixture was stirred for 24 h at rt. The crude material was purified by flash column chromatography (0–1% EtOAc in PE) to provide the title compound as a colorless liquid in 84% yield (40.1 mg) with 99:1 rr.

Rf 0.2 (2% EtOAc in PE), UV;

^{1}H NMR (500 MHz, $CDCl_3$) δ 7.32 (t, J = 7.5 Hz, 2H), 7.28 (d, J = 8.2 Hz, 2H), 7.21 (d, J = 8.8 Hz, 3H), 6.88 (d, J = 8.7 Hz, 2H), 3.92 (t, J = 7.8 Hz, 1H), 3.81 (s, 3H), 2.05 (q, J = 7.8 Hz, 2H), 1.38 – 1.29 (m, 2H), 0.98 (t, J = 7.3 Hz, 3H);

^{13}C NMR (126 MHz, $CDCl_3$) δ 157.8, 145.8, 137.6, 128.8, 128.4, 127.8, 125.9, 113.8, 55.2, 50.2, 38.1, 21.2, 14.1;

HRMS (ESI) calcd. for $C_{17}H_{21}O$ $[M+H]^+$ m/z 241.1587, found 241.1588;

IR (neat, cm^{-1}) 2955, 1609, 1509, 1246, 697;

$[\alpha]_D^{20}$ = –7.6 (c = 1.4, $CHCl_3$); 95% *ee*;

HPLC analysis CHIRALCEL OJ-H column, 1% EtOH in hexane, 0.5 mL/min, 220 nm UV detector, t_R (minor) = 23.6 min, t_R (major) = 26.5 min.

Note:

From **1-Bromo-4-methoxybenzene** (**2a'**) (74.8 mg, 0.40 mmol), the title compound was prepared following the general procedure **A**, and the crude material was purified by flash column chromatography (0–1% EtOAc in PE) to provide the title compound as a colorless liquid in 67% yield (38.9 mg) with 95% *ee* and >99:1 rr.

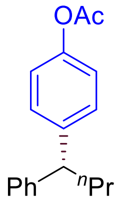

(*R*)-4-(1-Phenylbutyl)phenyl acetate (Figure 3, **3b**). From **4-Iodophenyl acetate** (104.8 mg, 0.40 mmol, 2.0 equiv), the title compound was prepared following the general procedure **A** using $NiCl_2$·glyme (2.2 mg, 5.0 mol%), **L*** (7.2 mg, 6.0 mol%), **L** [0.42 mg, 0.20 mL (2.1 mg/mL in toluene)], KF (23.2 mg, 2.0 equiv), 4-phenyl-1-butene (**1a**) (30.0

μL, 0.20 mmol), DMMS (49.3 μL, 0.40 mmol), anhydrous toluene (0.72 mL) and DMPU (0.20 mL). The reaction mixture was stirred for 24 h at rt. The crude material was purified by flash column chromatography (0–5% EtOAc in PE) to provide the title compound as a colorless liquid in 90% yield (48.3 mg) with 99:1 rr.

Rf 0.3 (5% EtOAc in PE), UV;

^{1}H NMR (500 MHz, $CDCl_3$) δ 7.34 – 7.30 (m, 2H), 7.30 – 7.26 (m, 4H), 7.22 (t, *J* = 7.2 Hz, 1H), 7.04 (d, *J* = 8.3 Hz, 2H), 3.96 (t, *J* = 7.8 Hz, 1H), 2.31 (s, 3H), 2.09 – 2.02 (m, 2H), 1.36 – 1.29 (m, 2H), 0.97 (t, *J* = 7.4 Hz, 3H);

^{13}C NMR (126 MHz, $CDCl_3$) δ 169.6, 148.8, 144.9, 142.9, 128.8, 128.5, 127.9, 126.2, 121.3, 50.5, 38.0, 21.2, 21.1, 14.1;

HRMS (ESI) calcd. for $C_{18}H_{20}NaO_2$ $[M+Na]^+$ *m/z* 291.1356, found 291.1354;

IR (neat, cm^{-1}) 2956, 1758, 1367, 1191, 698;

$[\alpha]_D^{20}$ = –4.2 (*c* = 2.2, $CHCl_3$); 90% *ee*;

HPLC analysis CHIRALCEL OJ-H column, 1% ^{i}PrOH in hexane, 0.8 mL/min, 220 nm UV detector, t_R (minor) = 28.9 min, t_R (major)= 31.6 min.

Note:

From **4-Bromophenyl acetate** (**2b**') (85.6 mg, 0.40 mmol), the title compound was prepared following the general procedure **A**, and the crude material was purified by flash column chromatography (0–5% EtOAc in PE) to provide the title compound as a colorless liquid in 74% yield (39.5 mg) with 90% *ee* and 99:1 rr.

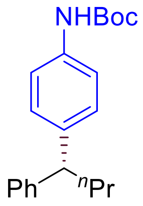

tert*-Butyl (*R*)-(4-(1-phenylbutyl)phenyl)carbamate** (Figure 3, **3c**). From ***tert*-Butyl (4-iodophenyl)carbamate** (127.7 mg, 0.40 mmol, 2.0 equiv), the title compound was prepared following the general procedure **A** using $NiCl_2$·glyme (2.2 mg, 5.0 mol%), **L (7.2 mg, 6.0 mol%), **L** [0.42 mg, 0.20 mL (2.1 mg/mL in toluene)], KF (23.2 mg, 2.0 equiv), 4-phenyl-1-butene (**1a**) (30.0 μL, 0.20 mmol), DMMS (49.3 μL, 0.40 mmol), anhydrous toluene (0.60 mL) and DMPU (0.20 mL). The reaction mixture was stirred for

24 h at rt. The crude material was purified by flash column chromatography (0–3% EtOAc in PE) to provide the title compound as a colorless liquid in 73% yield (47.5 mg) with 98:2 rr.

Rf 0.3 (5% EtOAc in PE), UV;

^{1}H NMR (500 MHz, $CDCl_3$) δ 7.34 – 7.27 (m, 4H), 7.26 – 7.23 (m, 2H), 7.23 – 7.15 (m, 3H), 6.49 (s, 1H), 3.90 (t, J = 7.8 Hz, 1H), 2.03 (q, J = 7.7 Hz, 2H), 1.54 (s, 9H), 1.36 – 1.25 (m, 2H), 0.95 (t, J = 7.4 Hz, 3H);

^{13}C NMR (126 MHz, $CDCl_3$) δ 152.9, 145.5, 140.1, 136.3, 128.4, 128.4, 127.8, 125.9, 118.8, 80.4, 50.4, 37.9, 28.4, 21.1, 14.1;

HRMS (ESI) calcd. for $C_{21}H_{27}NNaO_2$ $[M+Na]^+$ m/z 348.1934, found 348.1933;

IR (neat, cm^{-1}) 2929, 1699, 1519, 1154, 697;

$[\alpha]_D^{20}$ = –8.2 (c = 2.2, $CHCl_3$); 96% *ee*;

HPLC analysis CHIRALCEL OJ-H column, 1% ^{i}PrOH in hexane, 0.8 mL/min, 220 nm UV detector, t_R (minor) = 38.7 min, t_R (major) = 46.6 min.

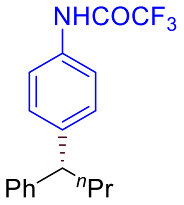

(*R*)-2,2,2-Trifluoro-*N*-(4-(1-phenylbutyl)phenyl)acetamide (Figure 3, **3d**). From **2,2,2-Trifluoro-*N*-(4-iodophenyl)acetamide** (126.0 mg, 0.40 mmol, 2.0 equiv), the title compound was prepared following the general procedure **A** using $NiCl_2$·glyme (2.2 mg, 5.0 mol%), **L*** (7.2 mg, 6.0 mol%), **L** [0.17 mg, 0.08 mL (2.1 mg/mL in toluene)], KF (23.2 mg, 2.0 equiv), 4-phenyl-1-butene (**1a**) (30.0 μL, 0.20 mmol), DMMS (49.3 μL, 0.40 mmol), anhydrous toluene (0.72 mL) and DMPU (0.20 mL). The reaction mixture was stirred for 24 h at rt. The crude material was purified by flash column chromatography (0–5% EtOAc in PE) to provide the title compound as a colorless liquid in 63% yield (39.4 mg) with 97:3 rr.

Rf 0.3 (5% EtOAc in PE), UV;

^{1}H NMR (500 MHz, $CDCl_3$) δ 8.00 (s, 1H), 7.50 (d, *J* = 8.3 Hz, 2H), 7.33 – 7.27 (m, 4H), 7.27 – 7.18 (m, 3H), 3.94 (t, *J* = 7.8 Hz, 1H), 2.11 – 1.99 (m, 2H), 1.36 – 1.25 (m, 3H), 0.96 (t, *J* = 7.4 Hz, 3H);

^{13}C NMR (126 MHz, $CDCl_3$) δ 154.8 (q, *J* = 37.2 Hz), 144.8, 143.8, 133.0, 128.8, 128.5, 127.8, 126.2, 120.6, 115.8 (q, *J* = 289.2 Hz), 50.5, 37.8, 21.1, 14.0;

^{19}F NMR (471 MHz, $CDCl_3$) δ –75.7;

HRMS (ESI) calcd. for $C_{18}H_{18}F_3NaO$ $[M+Na]^+$ *m/z* 344.1233, found 344.1234;

IR (neat, cm^{-1}) 2958, 1702, 1151, 697;

$[\alpha]_D^{20}$ = –9.5 (*c* = 1.1, $CHCl_3$); 92% *ee*;

HPLC analysis CHIRALCEL AD-H column, 5% ^{i}PrOH in hexane, 0.5 mL/min, 220 nm UV detector, t_R (minor) = 18.1 min, t_R (major) = 19.2 min.

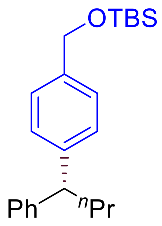

(*R*)-*tert*-Butyldimethyl((4-(1-phenylbutyl)benzyl)oxy)silane (Figure 3, **3e**). From ***tert*-Butyl((4-iodobenzyl)oxy)dimethylsilane** (139.2 mg, 0.40 mmol, 2.0 equiv), the title compound was prepared following the general procedure **A** using $NiCl_2$·glyme (2.2 mg, 5.0 mol%), **L*** (7.2 mg, 6.0 mol%), **L** [0.42 mg, 0.20 mL (2.1 mg/mL in toluene)], KF (23.2 mg, 2.0 equiv), 4-phenyl-1-butene (**1a**) (30.0 μL, 0.20 mmol), DMMS (49.3 μL, 0.40 mmol), anhydrous toluene (0.60 mL) and DMPU (0.20 mL). The reaction mixture was stirred for 24 h at rt. The crude material was purified by flash column chromatography (PE) to provide the title compound as a colorless liquid in 77% yield (54.3 mg) with 98:2 rr.

Rf 0.2 (PE), UV;

^{1}H NMR (500 MHz, $CDCl_3$) δ 7.33 – 7.24 (m, 8H), 7.20 (t, *J* = 6.8 Hz, 1H), 4.75 (s, 2H), 3.95 (t, *J* = 7.8 Hz, 1H), 2.07 (q, *J* = 7.8 Hz, 2H), 1.35 – 1.31 (m, 2H), 1.01 – 0.95 (m, 12H), 0.14 (s, 6H);

^{13}C NMR (126 MHz, $CDCl_3$) δ 145.5, 144.0, 139.1, 128.4, 127.9, 127.7, 126.2, 125.9, 64.8, 50.8, 38.0, 29.7, 26.0, 21.2, 18.5, 14.1, –5.2;

HRMS (ESI) calcd. for $C_{23}H_{35}OSi$ $[M+H]^+$ *m/z* 355.2452, found 355.2453;

IR (neat, cm^{-1}) 2955, 1252, 1082, 697;

$[\alpha]_D^{20}$ = –4.8 (*c* = 1.8, $CHCl_3$); 92% *ee*;

HPLC analysis CHIRALCEL OD-H column, hexane, 0.8 mL/min, 220 nm UV detector, t_R (minor) = 8.2 min, t_R (major) = 9.2 min.

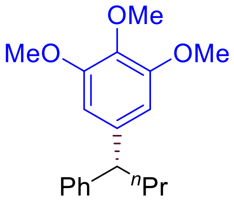

(*R*)-1,2,3-Trimethoxy-5-(1-phenylbutyl)benzene (Figure 3, **3f**). From **5-Iodo-1,2,3-trimethoxybenzene** (117.6 mg, 0.40 mmol, 2.0 equiv), the title compound was prepared following the general procedure **A** using $NiCl_2$·glyme (2.2 mg, 5.0 mol%), **L*** (7.2 mg, 6.0 mol%), **L** [0.17 mg, 0.08 mL (2.1 mg/mL in toluene)], KF (23.2 mg, 2.0 equiv), 4-phenyl-1-butene (**1a**) (30.0 μL, 0.20 mmol), DMMS (49.3 μL, 0.40 mmol), anhydrous toluene (0.72 mL) and DMPU (0.20 mL). The reaction mixture was stirred for 24 h at rt. The crude material was purified by flash column chromatography (0–3% EtOAc in PE) to provide the title compound as a colorless liquid in 71% yield (42.8 mg) with 99:1 rr.

Rf 0.3 (5% EtOAc in PE), UV;

^{1}H NMR (500 MHz, $CDCl_3$) δ 7.35 – 7.26 (m, 4H), 7.21 (t, *J* = 7.0 Hz, 1H), 6.49 (s, 2H), 3.90 – 3.83 (m, 10H), 2.07 – 1.98 (m, 2H), 1.36 – 1.30 (m, 2H), 0.97 (t, *J* = 7.4 Hz, 3H);

^{13}C NMR (126 MHz, $CDCl_3$) δ 153.1, 145.1, 141.1, 136.2, 128.4, 127.7, 126.1, 104.8, 60.8, 56.1, 51.4, 38.0, 21.2, 14.1;

HRMS (ESI) calcd. for $C_{19}H_{25}O_3$ $[M+H]^+$ *m/z* 301.1798, found 301.1797;

IR (neat, cm^{-1}) 2930, 1586, 1123, 700;

$[\alpha]_D^{20}$= –4.8 (*c* = 1.5, $CHCl_3$); 97% *ee*;

HPLC analysis CHIRALCEL IC column, 5% ^{i}PrOH in hexane, 1.0 mL/min, 220 nm UV detector, t_R (major) = 12.2 min, t_R (minor) = 15.5 min.

Note:

From **5-Bromo-1,2,3-trimethoxybenzene** (**2f'**) (98.8 mg, 0.40 mmol), the title compound was prepared following the general procedure **A**, and the crude material was purified by

flash column chromatography (0–3% EtOAc in PE) to provide the title compound as a colorless liquid in 55% yield (32.8 mg) with 96% *ee* and 99:1 rr.

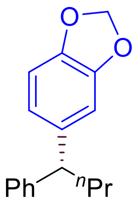

(*R*)-5-(1-Phenylbutyl)benzo[*d*][1,3]dioxole (Figure 3, **3g**). From **5-Iodobenzo[*d*][1,3]dioxole** (99.2 mg, 0.40 mmol, 2.0 equiv), the title compound was prepared following the general procedure **A** using $NiCl_2$·glyme (2.2 mg, 5.0 mol%), **L*** (7.2 mg, 6.0 mol%), **L** [0.17 mg, 0.08 mL (2.1 mg/mL in toluene)], KF (23.2 mg, 2.0 equiv), 4-phenyl-1-butene (**1a**) (30.0 μL, 0.20 mmol), DMMS (49.3 μL, 0.40 mmol), anhydrous toluene (0.72 mL) and DMPU (0.20 mL). The reaction mixture was stirred for 24 h at rt. The crude material was purified by flash column chromatography (0–2% EtOAc in PE) to provide the title compound as a colorless liquid in 70% yield (35.5 mg) with 99:1 rr.

Rf 0.2 (2% EtOAc in PE), UV;

^{1}H NMR (500 MHz, $CDCl_3$) δ 7.32 (t, J = 7.5 Hz, 2H), 7.27 (d, J = 6.7 Hz, 2H), 7.21 (t, J = 7.2 Hz, 1H), 6.77 (s, 3H), 5.93 (d, J = 1.9 Hz, 2H), 3.88 (t, J = 7.8 Hz, 1H), 2.07 – 1.96 (m, 2H), 1.40 – 1.26 (m, 2H), 0.97 (t, J = 7.4 Hz, 3H);

^{13}C NMR (126 MHz, $CDCl_3$) δ 147.7, 145.7, 145.4, 139.4, 128.4, 127.7, 126.1, 120.8, 108.3, 108.1, 100.8, 50.7, 38.0, 21.2, 14.1;

HRMS (ESI) calcd. for $C_{17}H_{19}O_2$ $[M+H]^+$ *m/z* 277.1199, found 277.1197;

IR (neat, cm^{-1}) 2955, 1484, 1243, 1037, 697;

$[\alpha]_D^{20}$ = –8.9 (c = 1.7, $CHCl_3$); 95% *ee*;

HPLC analysis CHIRALCEL OJ-H column, 5% ^{i}PrOH in hexane, 0.5 mL/min, 220 nm UV detector, t_R (minor) = 17.2 min, t_R (major) = 18.8 min.

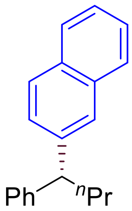

(*R*)-2-(1-Phenylbutyl)naphthalene (Figure 3, **3h**). From **2-Iodonaphthalene** (101.6 mg, 0.40 mmol, 2.0 equiv), the title compound was prepared following the general procedure **A** using $NiCl_2$·glyme (2.2 mg, 5.0 mol%), **L*** (7.2 mg, 6.0 mol%), **L** [0.17 mg, 0.08 mL (2.1 mg/mL in toluene)], KF (23.2 mg, 2.0 equiv), 4-phenyl-1-butene (**1a**) (30.0 μL, 0.20 mmol), DMMS (49.3 μL, 0.40 mmol), anhydrous toluene (0.72 mL) and DMPU (0.20 mL). The reaction mixture was stirred for 24 h at rt. The crude material was purified by flash column chromatography (PE) to provide the title compound as a colorless liquid in 74% yield (38.3 mg) with 99:1 rr.

Rf 0.5 (PE), UV;

^{1}H NMR (500 MHz, $CDCl_3$) δ 7.85 (dd, *J* = 11.6, 7.7 Hz, 2H), 7.82 – 7.77 (m, 2H), 7.54 – 7.44 (m, 2H), 7.42 (d, *J* = 8.4 Hz, 1H), 7.39 – 7.31 (m, 4H), 7.27 – 7.21 (m, 1H), 4.15 (t, *J* = 7.8 Hz, 1H), 2.28 – 2.13 (m, 2H), 1.45 – 1.36 (m, 2H), 1.03 (t, *J* = 7.4 Hz, 3H);

^{13}C NMR (126 MHz, $CDCl_3$) δ 145.3, 142.8, 133.6, 132.2, 128.4, 128.0, 128.0, 127.7, 127.6, 126.9, 126.1, 125.9, 125.9, 125.4, 51.1, 37.7, 21.3, 14.2;

HRMS (ESI) calcd. for $C_{20}H_{20}Na$ $[M+Na]^+$ *m/z* 283.1457, found 283.1459;

IR (neat, cm^{-1}) 2927, 1598, 742, 697;

$[\alpha]_D^{20}$ = –15.6 (*c* = 1.4, $CHCl_3$); 95% *ee*;

HPLC analysis CHIRALCEL OD-H column, hexane, 0.8 mL/min, 220 nm UV detector, t_R (minor) = 25.7 min, t_R (major) = 40.3 min.

Note:

From **2-Bromonaphthalene** (**2h**') (82.8 mg, 0.40 mmol), the title compound was prepared following the general procedure **A**, and the crude material was purified by flash column chromatography (PE) to provide the title compound as a colorless liquid in 55% yield (28.7 mg) with 95% *ee* and 99:1 rr.

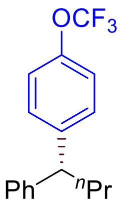

(*R*)-1-(1-Phenylbutyl)-4-(trifluoromethoxy)benzene (Figure 3, **3i**). From **1-Iodo-4-(trifluoromethoxy)benzene** (115.2 mg, 0.40 mmol, 2.0 equiv), the title compound was prepared following the general procedure **A** using $NiCl_2$·glyme (2.2 mg, 5.0 mol%), **L*** (7.2 mg, 6.0 mol%), **L** [0.42 mg, 0.2 mL (2.1 mg/mL in toluene)], KF (23.2 mg, 2.0 equiv), 4-phenyl-1-butene (**1a**) (30.0 μL, 0.20 mmol), DMMS (49.3 μL, 0.40 mmol), anhydrous toluene (0.60 mL) and DMPU (0.20 mL). The reaction mixture was stirred for 24 h at rt. The crude material was purified by flash column chromatography (0–1% EtOAc in PE) to provide the title compound as a colorless liquid in 67% yield (39.4 mg) with 98:2 rr.

Rf 0.2 (3% EtOAc in PE), UV;

^{1}H NMR (500 MHz, $CDCl_3$) δ 7.33 (t, *J* = 7.6 Hz, 2H), 7.30 – 7.25 (m, 4H), 7.23 (t, *J* = 7.2 Hz, 1H), 7.15 (d, *J* = 8.2 Hz, 2H), 3.96 (t, *J* = 7.8 Hz, 1H), 2.10 – 1.99 (m, 2H), 1.32 (q, *J* = 7.5 Hz, 2H), 0.97 (t, *J* = 7.4 Hz, 3H);

^{13}C NMR (126 MHz, $CDCl_3$) δ 147.4 (q, *J* = 1.7 Hz), 144.7, 144.1, 129.1, 128.5, 127.8, 126.3, 120.9, 120.5 (q, *J* = 256.6 Hz), 50.5, 37.9, 21.1, 14.0;

^{19}F NMR (471 MHz, $CDCl_3$) δ –57.9;

HRMS (ESI) calcd. for $C_{17}H_{18}F_3O$ $[M+H]^+$ *m/z* 295.1304, found 295.1306;

IR (neat, cm^{-1}) 1507, 1253, 1156, 697;

$[\alpha]_D^{20}$ = –2.1 (*c* = 1.2, $CHCl_3$); 92% *ee*;

HPLC analysis CHIRALCEL OJ-H column, hexane, 0.5 mL/min, 220 nm UV detector, t_R (minor) = 11.9 min, t_R (major) = 13.1 min.

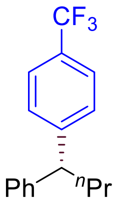

(*R*)-1-(1-Phenylbutyl)-4-(trifluoromethyl)benzene (Figure 3, **3j**). From **1-Iodo-4-(trifluoromethyl)benzene** (108.8 mg, 0.40 mmol, 2.0 equiv), the title compound was prepared following the general procedure **A** using $NiCl_2$·glyme (2.2 mg, 5.0 mol%), **L*** (7.2 mg, 6.0 mol%), **L** [0.17 mg, 0.08 mL (2.1 mg/mL in toluene)], KF (23.2 mg, 2.0 equiv), 4-phenyl-1-butene (**1a**) (30.0 μL, 0.20 mmol), DMMS (49.3 μL, 0.40 mmol), anhydrous toluene (0.72 mL) and DMPU (0.20 mL). The reaction mixture was stirred for 24 h at rt. The crude material was purified by flash column chromatography (0–1% EtOAc in PE) to provide the title compound as a colorless liquid in 67% yield (37.5 mg) with 97:3 rr.

Rf 0.5 (1% EtOAc in PE), UV;

^{1}H NMR (500 MHz, $CDCl_3$) δ 7.57 (d, *J* = 8.2 Hz, 2H), 7.40 (d, *J* = 8.1 Hz, 2H), 7.34 (t, *J* = 7.6 Hz, 2H), 7.27 (d, *J* = 6.7 Hz, 2H), 7.24 (t, *J* = 7.2 Hz, 1H), 4.02 (t, *J* = 7.8 Hz, 1H), 2.16 – 2.01 (m, 2H), 1.38 – 1.28 (m, 2H), 0.98 (t, *J* = 7.3 Hz, 3H);

^{13}C NMR (126 MHz, $CDCl_3$) δ 149.5, 144.3, 128.6, 128.2, 127.9, 127.8, 126.4, 125.4 (q, *J* = 3.9 Hz), 122.2 (q, *J* = 272.3 Hz), 50.9, 37.7, 21.1, 14.0;

^{19}F NMR (471 MHz, $CDCl_3$) δ –62.3;

HRMS (ESI) calcd. for $C_{17}H_{18}F_3$ $[M+H]^+$ *m/z* 279.1355, found 279.1358;

IR (neat, cm^{-1}) 2960, 1322, 1116, 1017, 698;

$[\alpha]_D^{20}$ = +1.6 (*c* = 1.7, $CHCl_3$); 95% *ee*;

HPLC analysis CHIRALCEL OJ-H*2 column, hexane, 0.4 mL/min, 220 nm UV detector, t_R (minor) = 31.5 min, t_R (major) = 34.2 min.

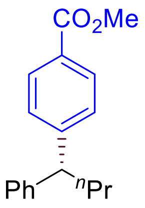

Methyl (*R*)-4-(1-phenylbutyl)benzoate (Figure 3, **3k**). From **Methyl 4-iodobenzoate** (104.8 mg, 0.40 mmol, 2.0 equiv), the title compound was prepared following the general procedure **A** using $NiCl_2$·glyme (2.2 mg, 5.0 mol%), **L*** (7.2 mg, 6.0 mol%), **L** [0.17 mg, 0.08 mL (2.1 mg/mL in toluene)], KF (23.2 mg, 2.0 equiv), 4-phenyl-1-butene (**1a**) (30.0 μL, 0.20 mmol), DMMS (49.3 μL, 0.40 mmol), anhydrous toluene (0.72 mL) and DMPU

(0.20 mL). The reaction mixture was stirred for 24 h at rt. The crude material was purified by flash column chromatography (0–5% EtOAc in PE) to provide the title compound as a colorless liquid in 80% yield (42.9 mg) with 98:2 rr.

Rf 0.2 (5% EtOAc in PE), UV;

^{1}H NMR (500 MHz, $CDCl_3$) δ 7.99 (d, *J* = 8.4 Hz, 2H), 7.37 – 7.28 (m, 4H), 7.26 (d, *J* = 8.1 Hz, 2H), 7.22 (t, *J* = 7.2 Hz, 1H), 4.00 (t, *J* = 7.8 Hz, 1H), 3.92 (s, 3H), 2.13 – 2.00 (m, 2H), 1.34 – 1.28 (m, 2H), 0.96 (t, *J* = 7.4 Hz, 3H);

^{13}C NMR (126 MHz, $CDCl_3$) δ 167.1, 150.8, 144.4, 129.8, 128.5, 128.0, 127.9, 127.9, 126.3, 52.0, 51.1, 37.6, 21.1, 14.1;

HRMS (ESI) calcd. for $C_{18}H_{21}O_2$ $[M+H]^+$ *m/z* 269.1536, found 269.1534;

IR (neat, cm^{-1}) 2954, 1718, 1274, 697;

$[\alpha]_D^{20}$ = –5.5 (*c* = 1.5, $CHCl_3$); 90% *ee*;

HPLC analysis CHIRALCEL IC column, 1% ^{i}PrOH in hexane, 0.8 mL/min, 220 nm UV detector, t_R (minor) = 13.2 min, t_R (major) = 15.1 min.

Note:

From **Methyl 4-bromobenzoate** (**2k**') (86.0 mg, 0.40 mmol), the title compound was prepared following the general procedure **A**, and the crude material was purified by flash column chromatography (0–1% EtOAc in PE) to provide the title compound as a colorless liquid in 50% yield (26.9 mg) with 91% *ee* and 98:2 rr.

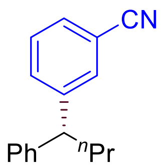

(*R*)-3-(1-Phenylbutyl)benzonitrile (Figure 3, **3l**). From **3-Iodobenzonitrile** (91.6 mg, 0.40 mmol, 2.0 equiv), the title compound was prepared following the general procedure **A** using $NiCl_2$·glyme (2.2 mg, 5.0 mol%), **L*** (7.2 mg, 6.0 mol%), **L** [0.17 mg, 0.08 mL (2.1 mg/mL in toluene)], KF (23.2 mg, 2.0 equiv), 4-phenyl-1-butene (**1a**) (30.0 μL, 0.20 mmol), DMMS (49.3 μL, 0.40 mmol), anhydrous toluene (0.72 mL) and DMPU (0.20 mL). The reaction mixture was stirred for 24 h at rt. The crude material was purified by flash column chromatography (0–1% EtOAc in PE) to provide the title compound as a colorless liquid in 71% yield (33.4 mg) with 96:4 rr.

Rf 0.4 (1% EtOAc in PE), UV;

^{1}H NMR (500 MHz, $CDCl_3$) δ 7.56 (s, 1H), 7.52 – 7.47 (m, 2H), 7.39 (t, *J* = 7.8 Hz, 1H), 7.35 – 7.31 (m, 2H), 7.23 (d, *J* = 7.7 Hz, 3H), 3.97 (t, *J* = 7.8 Hz, 1H), 2.12 – 1.98 (m, 2H), 1.33 – 1.27 (m, 2H), 0.96 (t, *J* = 7.3 Hz, 3H);

^{13}C NMR (126 MHz, $CDCl_3$) δ 146.9, 143.7, 132.6, 131.5, 129.8, 129.2, 128.7, 127.8, 126.6, 119.1, 112.4, 50.7, 37.6, 21.0, 14.0;

HRMS (ESI) calcd. for $C_{17}H_{18}N$ $[M+H]^+$ *m/z* 236.1434, found 236.1435;

IR (neat, cm^{-1}) 2957, 2228, 1452, 691;

$[\alpha]_D^{20}$ = –7.5 (*c* = 1.1, $CHCl_3$); 94% *ee*;

HPLC analysis CHIRALCEL OJ-H column, 5% ^{i}PrOH in pentane, 0.5 mL/min, 220 nm UV detector, t_R (major) = 25.7 min, t_R (minor) = 28.0 min.

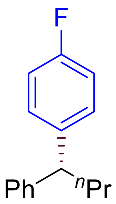

(*R*)-1-Fluoro-4-(1-phenylbutyl)benzene (Figure 3, **3m**). From **1-Fluoro-4-iodobenzene** (88.8 mg, 0.40 mmol, 2.0 equiv), the title compound was prepared following the general procedure **A** using $NiCl_2$·glyme (2.2 mg, 5.0 mol%), **L*** (7.2 mg, 6.0 mol%), **L** [0.17 mg, 0.08 mL (2.1 mg/mL in toluene)], KF (23.2 mg, 2.0 equiv), 4-phenyl-1-butene (**1a**) (30.0 μL, 0.20 mmol), DMMS (49.3 μL, 0.40 mmol), anhydrous toluene (0.72 mL) and DMPU (0.20 mL). The reaction mixture was stirred for 24 h at rt. The crude material was purified by flash column chromatography (0–1% EtOAc in PE) to provide the title compound as a colorless liquid in 74% yield (34.0 mg) with 97:3 rr.

Rf 0.5 (1% EtOAc in PE), UV;

^{1}H NMR (500 MHz, $CDCl_3$) δ 7.32 (t, *J* = 7.6 Hz, 2H), 7.27 – 7.20 (m, 5H), 7.00 (t, *J* = 8.7 Hz, 2H), 3.94 (t, *J* = 7.8 Hz, 1H), 2.11 – 1.97 (m, 2H), 1.37 – 1.26 (m, 2H), 0.97 (t, *J* = 7.3 Hz, 3H);

^{13}C NMR (126 MHz, $CDCl_3$) δ 161.3 (d, *J* = 243.8 Hz), 145.2, 141.1 (d, *J* = 3.0 Hz), 129.2 (d, *J* = 7.6 Hz), 128.5, 127.8, 126.2, 115.1 (d, *J* = 21.3 Hz), 50.3, 38.1, 21.13, 14.1;

^{19}F NMR (471 MHz, $CDCl_3$) δ –117.5;

HRMS (ESI) calcd. for $C_{17}H_{18}F$ $[M+H]^+$ *m/z* 229.1387, found 229.1390;

IR (neat, cm^{-1}) 2957, 1507, 1222, 697;

$[\alpha]_D^{20}$ = –2.7 (*c* = 1.1, $CHCl_3$); 94% *ee*;

HPLC analysis CHIRALCEL OJ-H column, 20% ^{i}PrOH in hexane, 0.8 mL/min, 220 nm UV detector, t_R (minor) = 5.5 min, t_R (major) = 6.3 min.

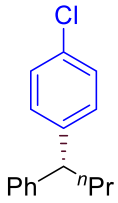

(*R*)-1-Chloro-4-(1-phenylbutyl)benzene (Figure 3, **3n**). From **1-Chloro-4-iodobenzene** (95.4 mg, 0.40 mmol, 2.0 equiv), the title compound was prepared following the general procedure **A** using $NiCl_2$·glyme (2.2 mg, 5.0 mol%), **L*** (7.2 mg, 6.0 mol%), **L** [0.17 mg, 0.08 mL (2.1 mg/mL in toluene)], KF (23.2 mg, 2.0 equiv), 4-phenyl-1-butene (**1a**) (30.0 µL, 0.20 mmol), DMMS (49.3 µL, 0.40 mmol), anhydrous toluene (0.72 mL) and DMPU (0.20 mL). The reaction mixture was stirred for 24 h at rt. The crude material was purified by flash column chromatography (0–1% EtOAc in PE) to provide the title compound as a colorless liquid in 74% yield (36.3 mg) with 98:2 rr.

Rf 0.4 (1% EtOAc in PE), UV;

^{1}H NMR (500 MHz, $CDCl_3$) δ 7.32 (t, *J* = 7.6 Hz, 2H), 7.28 (d, *J* = 8.5 Hz, 2H), 7.26 – 7.18 (m, 5H), 3.93 (t, *J* = 7.8 Hz, 1H), 2.11 – 1.97 (m, 2H), 1.36 – 1.27 (m, 2H), 0.97 (t, *J* = 7.4 Hz, 3H);

^{13}C NMR (126 MHz, $CDCl_3$) δ 144.8, 143.8, 131.7, 129.2, 128.5, 127.8, 126.2, 50.5, 37.8, 21.1, 14.0;

HRMS (ESI) calcd. for $C_{16}H_{17}ClNa$ $[M+Na]^+$ *m/z* 267.0911, found 267.0909;

IR (neat, cm^{-1}) 2956, 1488, 1091, 1013, 697;

$[\alpha]_D^{20}$ = –6.0 (*c* = 1.4, $CHCl_3$); 91% *ee*;

HPLC analysis CHIRALCEL OJ-H column, 5% ^{i}PrOH in hexane, 1.0 mL/min, 220 nm UV detector, t_R (minor) = 4.5 min, t_R (major) = 4.9 min.

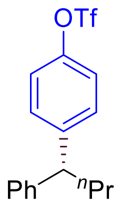

(*R*)-4-(1-Phenylbutyl)phenyl trifluoromethanesulfonate (Figure 3, **3o**). From **4-Iodophenyl trifluoromethanesulfonate** (140.8 mg, 0.40 mmol, 2.0 equiv), the title compound was prepared following the general procedure **A** using $NiCl_2$·glyme (2.2 mg, 5.0 mol%), **L*** (7.2 mg, 6.0 mol%), **L** [0.17 mg, 0.08 mL (2.1 mg/mL in toluene)], KF (23.2 mg, 2.0 equiv), 4-phenyl-1-butene (**1a**) (30.0 µL, 0.20 mmol), DMMS (49.3 µL, 0.40 mmol), anhydrous toluene (0.72 mL) and DMPU (0.20 mL). The reaction mixture was stirred for 24 h at rt. The crude material was purified by flash column chromatography (0–1% EtOAc in PE) to provide the title compound as a colorless liquid in 68% yield (48.8 mg) with 97:3 rr.

Rf 0.4 (1% EtOAc in PE), UV;

^{1}H NMR (500 MHz, $CDCl_3$) δ 7.46 – 7.28 (m, 5H), 7.28 – 7.15 (m, 4H), 3.97 (t, *J* = 7.3 Hz, 1H), 2.25 – 1.98 (m, 2H), 1.39 – 1.26 (m, 2H), 0.96 (t, *J* = 6.9 Hz, 3H);

^{13}C NMR (126 MHz, $CDCl_3$) δ 147.8, 146.0, 144.6, 129.6, 127.9, 126.5, 123.4, 121.2, 116.2 (d, *J* = 322.3 Hz), 50.5, 37.9, 21.1, 14.0;

^{19}F NMR (471 MHz, $CDCl_3$) δ –72.9;

HRMS (ESI) calcd. for $C_{17}H_{17}F_3NaO_3S[M+Na]^+$ *m/z* 381.0743, found 381.0740;

IR (neat, cm^{-1}) 2960, 1421, 1207, 1137, 885;

$[\alpha]_D^{20}$ = –1.6 (*c* = 2.3, $CHCl_3$); 94% *ee*;

HPLC analysis CHIRALCEL OJ-H column, 1% ^{i}PrOH in hexane, 0.8 mL/min, 220 nm UV detector, t_R (minor) = 8.3 min, t_R (major) = 9.8 min.

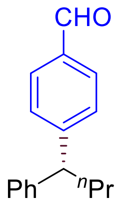

(*R*)-4-(1-Phenylbutyl)benzaldehyde (Figure 3, **3p**). From **4-Iodobenzaldehyde** (93.0 mg, 0.40 mmol, 2.0 equiv), the title compound was prepared following the general

procedure **A** using $NiCl_2$·glyme (2.2 mg, 5.0 mol%), **L*** (7.2 mg, 6.0 mol%), **L** [0.17 mg, 0.08 mL (2.1 mg/mL in toluene)], KF (23.2 mg, 2.0 equiv), 4-phenyl-1-butene (**1a**) (30.0 μL, 0.20 mmol), DMMS (49.3 μL, 0.40 mmol), anhydrous toluene (0.72 mL) and DMPU (0.20 mL). The reaction mixture was stirred for 24 h at rt. The crude material was purified by flash column chromatography (0–3% EtOAc in PE) to provide the title compound as a colorless liquid in 71% yield (34.2 mg) with 97:3 rr.

Rf 0.2 (4% EtOAc in PE), UV;

^{1}H NMR (500 MHz, $CDCl_3$) δ 9.98 (s, 1H), 7.82 (d, *J* = 8.2 Hz, 2H), 7.44 (d, *J* = 8.1 Hz, 2H), 7.32 (t, *J* = 7.6 Hz, 2H), 7.26 (d, *J* = 6.9 Hz, 2H), 7.22 (t, *J* = 7.2 Hz, 1H), 4.02 (t, *J* = 7.8 Hz, 1H), 2.12 – 2.03 (m, 2H), 1.34 – 1.29 (m, 2H), 0.96 (t, *J* = 7.3 Hz, 3H);

^{13}C NMR (126 MHz, $CDCl_3$) δ 192.0, 152.7, 144.0, 134.6, 130.0, 128.6, 128.6, 127.9, 126.5, 51.3, 37.6, 21.1, 14.0;

HRMS (ESI) calcd. for $C_{17}H_{19}O$ $[M+H]^+$ *m/z* 239.1430, found 239.1431;

IR (neat, cm^{-1}) 2956, 1698, 1603, 698;

$[\alpha]_D^{20}$ = –1.9 (*c* = 1.5, $CHCl_3$); 90% *ee*;

HPLC analysis CHIRALCEL OJ-H column, 10% ^{i}PrOH in hexane, 0.8 mL/min, 220 nm UV detector, t_R (minor) = 10.4 min, t_R (major) =11.5 min.

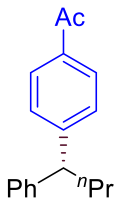

(*R*)-1-(4-(1-Phenylbutyl)phenyl)ethan-1-one (Figure 3, **3q**). From **1-(4-Iodophenyl)ethan-1-one** (98.4 mg, 0.40 mmol, 2.0 equiv), the title compound was prepared following the general procedure **A** using $NiCl_2$·glyme (2.2 mg, 5.0 mol%), **L*** (7.2 mg, 6.0 mol%), **L** [0.17 mg, 0.08 mL (2.1 mg/mL in toluene)], KF (23.2 mg, 2.0 equiv), 4-phenyl-1-butene (**1a**) (30.0 μL, 0.20 mmol), DMMS (49.3 μL, 0.40 mmol), anhydrous toluene (0.72 mL) and DMPU (0.20 mL). The reaction mixture was stirred for 24 h at rt. The crude material was purified by flash column chromatography (0–3% EtOAc in PE) to provide the title compound as a colorless liquid in 71% yield (35.8 mg) with 97:3 rr.

Rf 0.2 (3% EtOAc in PE), UV;

^{1}H NMR (500 MHz, $CDCl_3$) δ 7.91 (d, *J* = 8.4 Hz, 2H), 7.37 (d, *J* = 8.4 Hz, 2H), 7.34 – 7.30 (m, 2H), 7.26 (d, *J* = 8.5 Hz, 2H), 7.22 (t, *J* = 7.2 Hz, 1H), 4.01 (t, *J* = 7.8 Hz, 1H), 2.59 (s, 3H), 2.12 – 2.04 (m, 2H), 1.35 – 1.29 (m, 2H), 0.97 (t, *J* = 7.4 Hz, 3H);

^{13}C NMR (126 MHz, $CDCl_3$) δ 197.8, 151.1, 144.3, 135.2, 128.6, 128.6, 128.1, 127.9, 126.4, 51.1, 37.6, 26.6, 21.1, 14.0;

HRMS (ESI) calcd. for $C_{18}H_{21}O$ $[M+H]^+$ *m/z* 253.1587, found 253.1586;

IR (neat, cm^{-1}) 2957, 1679, 1604, 1266, 699;

$[\alpha]_D^{20}$ = –1.9 (*c* = 1.5, $CHCl_3$); 90% *ee*;

HPLC analysis CHIRALCEL OJ-H column, 10% EtOH in hexane, 0.5 mL/min, 220 nm UV detector, t_R (minor) = 9.0 min, t_R (major) = 9.6 min.

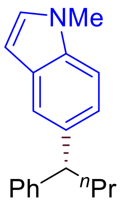

(*R*)-1-Methyl-5-(1-phenylbutyl)-1*H*-indole (Figure 3, **3r**). From **5-Iodo-1-methyl-1*H*-indole** (102.8 mg, 0.40 mmol, 2.0 equiv), the title compound was prepared following the general procedure **A** using $NiCl_2$·glyme (2.2 mg, 5.0 mol%), **L*** (7.2 mg, 6.0 mol%), **L** [0.17 mg, 0.08 mL (2.1 mg/mL in toluene)], KF (23.2 mg, 2.0 equiv), 4-phenyl-1-butene (**1a**) (30.0 μL, 0.20 mmol), DMMS (49.3 μL, 0.40 mmol), anhydrous toluene (0.72 mL) and DMPU (0.20 mL). The reaction mixture was stirred for 24 h at rt. The crude material was purified by flash column chromatography (0–1% EtOAc in PE) to provide the title compound as a colorless liquid in 60% yield (31.5 mg) with 98:2 rr.

Rf 0.2 (5% EtOAc in PE), UV;

^{1}H NMR (500 MHz, $CDCl_3$) δ 7.59 (s, 1H), 7.38 – 7.31 (m, 4H), 7.29 (d, *J* = 8.4 Hz, 1H), 7.21 (t, *J* = 7.1 Hz, 1H), 7.18 (d, *J* = 8.5 Hz, 1H), 7.06 (d, *J* = 3.1 Hz, 1H), 6.50 (d, *J* = 3.1 Hz, 1H), 4.10 (t, *J* = 7.8 Hz, 1H), 3.79 (s, 3H), 2.21 – 2.12 (m, 2H), 1.43 – 1.36 (m, 2H), 1.02 (t, *J* = 7.3 Hz, 3H);

^{13}C NMR (126 MHz, $CDCl_3$) δ 146.6, 136.4, 135.5, 128.9, 128.6, 128.3, 127.9, 125.7, 122.2, 119.6, 109.1, 100.8, 51.1, 38.4, 32.9, 21.4, 14.2;

HRMS (ESI) calcd. for $C_{19}H_{21}NNa$ $[M+Na]^+$ *m/z* 286.1566, found 286.1565;

IR (neat, cm^{-1}) 2926, 1512, 1245, 698;

$[\alpha]_D^{20}$ = –15.7 (*c* = 1.4, $CHCl_3$); 95% *ee*;

HPLC analysis CHIRALCEL IC column, 1% EtOH in hexane, 0.5 mL/min, 220 nm UV detector, t_R (major) = 10.0 min, t_R (minor) = 10.7 min.

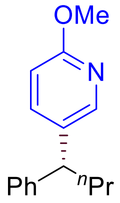

(*R*)-2-Methoxy-5-(1-phenylbutyl)pyridine (Figure 3, **3s**). From **5-Iodo-2-methoxypyridine** (94.0 mg, 0.40 mmol, 2.0 equiv), the title compound was prepared following the general procedure **A** using $NiCl_2$·glyme (2.2 mg, 5.0 mol%), **L*** (7.2 mg, 6.0 mol%), **L** [0.42 mg, 0.2 mL (2.1 mg/mL in toluene)], KF (23.2 mg, 2.0 equiv), 4-phenyl-1-butene (**1a**) (30.0 μL, 0.20 mmol), DMMS (49.3 μL, 0.40 mmol), anhydrous toluene (0.60 mL) and DMPU (0.20 mL). The reaction mixture was stirred for 24 h at rt. The crude material was purified by flash column chromatography (0–4% EtOAc in PE) to provide the title compound as a colorless liquid in 76% yield (36.4 mg) with 97:3 rr.

Rf 0.2 (5% EtOAc in PE), UV;

^{1}H NMR (500 MHz, $CDCl_3$) δ 8.09 (s, 1H), 7.44 (dd, *J* = 8.6, 2.5 Hz, 1H), 7.31 (t, *J* = 7.6 Hz, 2H), 7.24 (d, *J* = 6.9 Hz, 2H), 7.20 (t, *J* = 7.2 Hz, 1H), 6.69 (d, *J* = 8.5 Hz, 1H), 3.94 (s, 3H), 3.89 (t, *J* = 7.8 Hz, 1H), 2.07 – 1.96 (m, 2H), 1.34 – 1.29 (m, 2H), 0.95 (t, *J* = 7.4 Hz, 3H);

^{13}C NMR (126 MHz, $CDCl_3$) δ 162.7, 145.6, 144.7, 138.3, 133.3, 128.5, 127.7, 126.3, 110.7, 53.4, 47.7, 37.7, 21.0, 14.0;

HRMS (ESI) calcd. for $C_{16}H_{20}NO$ $[M+H]^+$ *m/z* 242.1539, found 242.1540;

IR (neat, cm^{-1}) 2955, 1603, 1489, 1026, 697;

$[\alpha]_D^{20}$ = +8.9 (*c* = 1.5, $CHCl_3$); 90% *ee*;

HPLC analysis CHIRALCEL AD-H column, 5% ^{i}PrOH in hexane, 0.8 mL/min, 220 nm UV detector, t_R (major) = 5.2 min, t_R (minor) = 5.9 min.

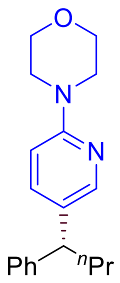

(*R*)-4-(5-(1-Phenylbutyl)pyridin-2-yl)morpholine (Figure 3, **3t**). From **4-(5-Iodopyridin-2-yl)morpholine** (116.0 mg, 0.40 mmol, 2.0 equiv), the title compound was prepared following the general procedure **A** using $NiCl_2$·glyme (2.2 mg, 5.0 mol%), **L*** (7.2 mg, 6.0 mol%), **L** [0.42 mg, 0.2 mL (2.1 mg/mL in toluene)], KF (23.2 mg, 2.0 equiv), 4-phenyl-1-butene (**1a**) (30.0 μL, 0.20 mmol), DMMS (49.3 μL, 0.40 mmol), anhydrous toluene (0.60 mL) and DMPU (0.20 mL). The reaction mixture was stirred for 24 h at rt. The crude material was purified by flash column chromatography (0–1% EtOAc in PE) to provide the title compound as a colorless liquid in 61% yield (36.2 mg) with 97:3 rr.

Rf 0.4 (15% EtOAc in PE), UV;

^{1}H NMR (500 MHz, $CDCl_3$) δ 8.15 (s, 1H), 7.38 (dd, *J* = 2.5, 2.5 Hz, 1H), 7.33 – 7.22 (m, 4H), 7.19 (t, *J* = 7.2 Hz, 1H), 6.60 (d, *J* = 8.7 Hz, 1H), 3.86 – 3.81 (m, 5H), 3.52 – 3.45 (m, 4H), 2.07 – 1.96 (m, 2H), 1.37 – 1.27 (m, 2H), 0.95 (t, *J* = 7.4 Hz, 3H);

^{13}C NMR (126 MHz, $CDCl_3$) δ 158.2, 145.1, 137.3, 130.5, 128.5, 127.7, 126.1, 107.0, 66.8, 47.7, 45.8, 37.6, 21.0, 14.0;

HRMS (ESI) calcd. for $C_{19}H_{25}N_2O$ $[M+H]^+$ *m/z* 297.1961, found 297.1962;

IR (neat, cm^{-1}) 2956, 1602, 1488, 1243, 943, 698;

$[\alpha]_D^{20}$ = –0.5 (*c* = 1.6, $CHCl_3$); 95% *ee*;

HPLC analysis CHIRALCEL AD-H column, 2% ^{i}PrOH in hexane, 1.0 mL/min, 220 nm UV detector, t_R (major) = 8.1 min, t_R (minor) = 10.4 min.

Note:

From **4-(5-Bromopyridin-2-yl)morpholine** (**2t'**) (97.2 mg, 0.40 mmol), the title compound was prepared following the general procedure **A**, and the crude material was purified by flash column chromatography (0–1% EtOAc in PE) to provide the title compound as a colorless liquid in 47% yield (38.9 mg) with 95% *ee* and 97:3 rr.

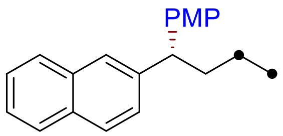

(*S*)-2-(1-(4-Methoxypenyl)butyl)naphthalene (Figure 4, **4b**). From **2-(But-3-en-1-yl)naphthalene** (36.4 mg, 0.20 mmol), the title compound was prepared following the general procedure **A** using $NiCl_2$·glyme (2.2 mg, 5.0 mol%), **L*** (7.2 mg, 6.0 mol%), **L** [0.42 mg, 0.2 mL (2.1 mg/mL in toluene)], KF (23.2 mg, 2.0 equiv), 4-iodoanisole (94.0 mg, 0.40 mmol, 2.0 equiv), DMMS (49.3 μL, 0.40 mmol), anhydrous toluene (0.60 mL) and DMPU (0.20 mL). The reaction mixture was stirred for 24 h at rt. The crude material was purified by flash column chromatography (0–2% EtOAc in PE) to provide the title compound as a colorless liquid in 78% yield (45.2 mg) with 97:3 rr.

Rf 0.2 (2% EtOAc in PE), UV;

^{1}H NMR (500 MHz, $CDCl_3$) δ 7.86 (dd, *J* = 11.8, 8.1 Hz, 2H), 7.80 (d, *J* = 8.5 Hz, 1H), 7.77 (s, 1H), 7.54 – 7.46 (m, 2H), 7.40 (d, *J* = 8.5 Hz, 1H), 7.27 (d, *J* = 8.8 Hz, 2H), 6.90 (d, *J* = 8.7 Hz, 2H), 4.11 (t, *J* = 7.8 Hz, 1H), 3.83 (s, 3H), 2.22 – 2.10 (m, 2H), 1.43 – 1.36 (m, 2H), 1.03 (t, *J* = 7.3 Hz, 3H);

^{13}C NMR (126 MHz, $CDCl_3$) δ 157.9, 143.2, 137.4, 133.6, 132.2, 128.9, 128.1, 127.7, 127.6, 126.9, 125.9, 125.7, 125.3, 113.8, 55.3, 50.3, 37.9, 21.3, 14.2;

HRMS (ESI) calcd. for $C_{21}H_{23}O$ $[M+H]^+$ *m/z* 291.1743, found 291.1745;

IR (neat, cm^{-1}) 2955, 1508, 1245, 1035, 813;

$[\alpha]_D^{20}$ = +7.8 (*c* = 2.1, $CHCl_3$); 90% *ee*;

HPLC analysis CHIRALCEL AD-H column, 5% ^{i}PrOH in hexane, 0.8 mL/min, 220 nm UV detector, t_R (minor) = 6.5 min, t_R (major) = 7.3 min.

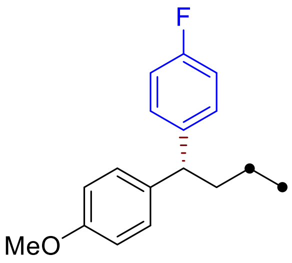

(*R*)-1-Fluoro-4-(1-(4-methoxyphenyl)butyl)benzene (Figure 4, **4c**). From **1-(But-3-en-1-yl)-4-methoxybenzene** (32.4 mg, 0.20 mmol), the title compound was prepared

following the general procedure **A** using $NiCl_2$·glyme (2.2 mg, 5.0 mol%), **L*** (7.2 mg, 6.0 mol%), **L** [0.84 mg, 0.4 mL (2.1 mg/mL in toluene)], KF (23.2 mg, 2.0 equiv), 1-fluoro-4-iodobenzene (88.8 mg, 0.40 mmol, 2.0 equiv), DMMS (49.3 μL, 0.40 mmol), anhydrous toluene (0.40 mL) and DMPU (0.20 mL). The reaction mixture was stirred for 24 h at rt. The crude material was purified by flash column chromatography (0–2% EtOAc in PE) to provide the title compound as a colorless liquid in 74% yield (38.2 mg) with 98:2 rr.

Rf 0.3 (3% EtOAc in PE), UV;

^{1}H NMR (500 MHz, $CDCl_3$) δ 7.20 (dd, *J* = 8.6, 5.5 Hz, 2H), 7.16 (d, *J* = 8.6 Hz, 2H), 6.98 (t, *J* = 8.7 Hz, 2H), 6.86 (d, *J* = 8.7 Hz, 2H), 3.88 (t, *J* = 7.8 Hz, 1H), 3.81 (s, 3H), 2.03 – 1.95 (m, 2H), 1.32 – 1.27 (m, 2H), 0.95 (t, *J* = 7.3 Hz, 3H);

^{13}C NMR (126 MHz, $CDCl_3$) δ 161.2 (d, *J* = 243.8 Hz), 157.9, 137.3, 141.5 (d, *J* = 3.0 Hz), 129.1 (d, *J* = 7.9 Hz), 128.6, 115.1 (d, *J* = 20.9 Hz), 113.8, 55.2, 49.4, 38.2, 21.1, 14.1;

^{19}F NMR (471 MHz, $CDCl_3$) δ –117.7;

HRMS (ESI) calcd. for $C_{17}H_{19}FNaO$ $[M+Na]^+$ *m/z* 281.1312, found 281.1313;

IR (neat, cm^{-1}) 2957, 1505, 1247, 1036, 824;

$[\alpha]_D^{20}$ = +5.3 (*c* = 1.5, $CHCl_3$); 90% *ee*;

HPLC analysis CHIRALCEL OJ-H*2 column, 1% EtOH in hexane, 0.5 mL/min, 220 nm UV detector, t_R (minor) = 37.6 min, t_R (major) = 41.6 min.

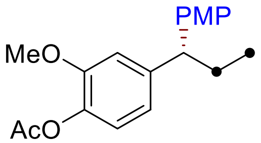

(*S*)-2-Methoxy-4-(1-(4-methoxyphenyl)propyl)phenyl acetate (Figure 4, **4d**). From **4-Allyl-2-methoxyphenyl acetate** (41.2 mg, 0.20 mmol), the title compound was prepared following the general procedure **A** using $NiCl_2$·glyme (2.2 mg, 5.0 mol%), **L*** (7.2 mg, 6.0 mol%), **L** [0.17 mg, 0.08 mL (2.1 mg/mL in toluene)], KF (23.2 mg, 2.0 equiv), 4-phenyl-1-butene (**1a**) (30.0 μL, 0.20 mmol), DMMS (49.3 μL, 0.40 mmol), anhydrous toluene (0.72 mL) and DMPU (0.20 mL). The reaction mixture was stirred for 24 h at rt.

The crude material was purified by flash column chromatography (0–8% EtOAc in PE) to provide the title compound as a colorless liquid in 72% yield (45.0 mg) with 99:1 rr.

Rf 0.3 (10% EtOAc in PE), UV;

^{1}H NMR (500 MHz, $CDCl_3$) δ 7.18 (d, *J* = 8.7 Hz, 2H), 6.96 (d, *J* = 7.9 Hz, 1H), 6.90 – 6.80 (m, 4H), 3.81 (s, 6H), 3.76 (t, *J* = 7.8 Hz, 1H), 2.32 (s, 3H), 2.05 (p, *J* = 7.4 Hz, 2H), 0.93 (t, *J* = 7.3 Hz, 3H);

^{13}C NMR (126 MHz, $CDCl_3$) δ 169.2, 157.9, 150.8, 144.6, 137.8, 136.8, 128.8, 122.4, 119.8, 113.8, 112.2, 55.8, 55.2, 52.3, 28.9, 20.7, 12.8;

HRMS (ESI) calcd. for $C_{19}H_{22}NaO_4$ $[M+Na]^+$ *m/z* 337.1410, found 337.1412;

IR (neat, cm^{-1}) 2967, 1762, 1508, 1195, 1033;

$[\alpha]_D^{20}$ = –7.2 (*c* = 1.6, $CHCl_3$); 90% *ee*;

HPLC analysis CHIRALCEL OJ-H column, 5% EtOH in hexane, 0.5 mL/min, 220 nm UV detector, t_R (minor) = 45.3 min, t_R (major) = 49.2 min.

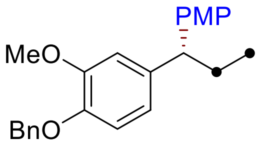

(*S*)-1-(Benzyloxy)-2-methoxy-4-(1-(4-methoxyphenyl)propyl)benzene (Figure 4, **4e**). From **4-Allyl-1-(benzyloxy)-2-methoxybenzene** (50.9 mg, 0.20 mmol), the title compound was prepared following the general procedure **A** using $NiCl_2$·glyme (2.2 mg, 5.0 mol%), **L*** (7.2 mg, 6.0 mol%), **L** [0.17 mg, 0.08 mL (2.1 mg/mL in toluene)], KF (23.2 mg, 2.0 equiv), 4-phenyl-1-butene (**1a**) (30.0 μL, 0.20 mmol), DMMS (49.3 μL, 0.40 mmol), anhydrous toluene (0.72 mL) and DMPU (0.20 mL). The reaction mixture was stirred for 24 h at rt. The crude material was purified by flash column chromatography (0–5% EtOAc in PE) to provide the title compound as a colorless liquid in 72% yield (52.0 mg) with 99:1 rr.

Rf 0.2 (5% EtOAc in PE), UV;

^{1}H NMR (500 MHz, $CDCl_3$) δ 7.47 (d, *J* = 7.5 Hz, 2H), 7.39 (t, *J* = 7.5 Hz, 2H), 7.33 (t, *J* = 7.3 Hz, 1H), 7.18 (d, *J* = 8.6 Hz, 2H), 6.89 – 6.83 (m, 3H), 6.79 (s, 1H), 6.75 (d, *J* = 8.2 Hz, 1H), 5.15 (s, 2H), 3.88 (s, 3H), 3.81 (s, 3H), 3.72 (t, *J* = 7.8 Hz, 1H), 2.04 (p, *J* = 7.4 Hz, 2H), 0.93 (t, *J* = 7.3 Hz, 3H);

^{13}C NMR (126 MHz, $CDCl_3$) δ 157.8, 149.5, 146.5, 138.9, 137.5, 137.5, 128.7, 128.5, 127.7, 127.3, 119.6, 113.9, 113.7, 111.9, 71.1, 56.0, 55.2, 52.0, 28.9, 12.9;

HRMS (ESI) calcd. for $C_{24}H_{26}NaO_3$ $[M+Na]^+$ *m/z* 385.1774, found 385.1777;

IR (neat, cm^{-1}) 2966, 1508, 1245, 1034, 695;

$[\alpha]_D^{20}$ = +0.9 (c = 2.4, $CHCl_3$); 95% *ee*;

HPLC analysis CHIRALCEL AD-H column, 10% EtOH in hexane, 1.0 mL/min, 220 nm UV detector, t_R (minor) = 8.5 min, t_R (major) = 9.7 min.

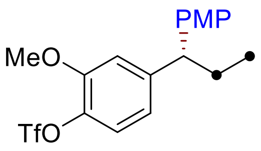

(*S*)-2-Methoxy-4-(1-(4-methoxyphenyl)propyl)phenyl trifluoromethanesulfonate (Figure 4, **4f**). From **4-Allyl-2-methoxyphenyl trifluoromethanesulfonate** (59.3 mg, 0.20 mmol), the title compound was prepared following the general procedure **A** using $NiCl_2$·glyme (2.2 mg, 5.0 mol%), **L*** (7.2 mg, 6.0 mol%), **L** [0.17 mg, 0.08 mL (2.1 mg/mL in toluene)], KF (23.2 mg, 2.0 equiv), 4-phenyl-1-butene (**1a**) (30.0 μL, 0.20 mmol), DMMS (49.3 μL, 0.40 mmol), anhydrous toluene (0.72 mL) and DMPU (0.20 mL). The reaction mixture was stirred for 24 h at rt. The crude material was purified by flash column chromatography (0–2% EtOAc in PE) to provide the title compound as a colorless liquid in 58% yield (47.1 mg) with 96:4 rr.

Rf 0.2 (3% EtOAc in PE), UV;

^{1}H NMR (500 MHz, $CDCl_3$) δ 7.15 (dd, J = 13.9, 8.4 Hz, 3H), 6.92 – 6.83 (m, 4H), 3.88 (s, 3H), 3.81 (s, 3H), 3.77 (t, J = 7.8 Hz, 1H), 2.10 – 2.00 (m, 2H), 0.92 (t, J = 7.3 Hz, 3H);

^{13}C NMR (126 MHz, $CDCl_3$) δ 158.2, 151.1, 147.4, 136.9, 136.0, 128.8, 122.1, 119.9, 116.2 (q, J = 320.4 Hz), 113.9, 112.7, 56.1, 55.2, 52.3, 28.8, 12.7;

^{19}F NMR (471 MHz, $CDCl_3$) δ –73.9;

HRMS (ESI) calcd. for $C_{18}H_{20}F_3O_5S$ $[M+H]^+$ *m/z* 405.0978, found 405.0978;

IR (neat, cm^{-1}) 2969, 1416, 1202, 878, 612;

$[\alpha]_D^{20}$ = –7.2 (c = 2.3, $CHCl_3$); 80% *ee*;

HPLC analysis CHIRALCEL OJ-H column, 10% EtOH in hexane, 0.8 mL/min, 220 nm UV detector, t_R (minor) = 12.4 min, t_R (major) = 14.1 min.

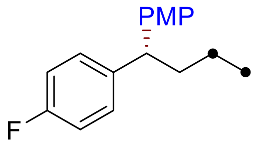

(*S*)-1-Fluoro-4-(1-(4-methoxyphenyl)butyl)benzene (Figure 4, **4g**). From **1-(But-3-en-1-yl)-4-fluorobenzene** (30.1 mg, 0.20 mmol), the title compound was prepared following the general procedure **A** using $NiCl_2$·glyme (2.2 mg, 5.0 mol%), **L*** (7.2 mg, 6.0 mol%), **L** [0.17 mg, 0.08 mL (2.1 mg/mL in toluene)], KF (23.2 mg, 2.0 equiv), 4-phenyl-1-butene (**1a**) (30.0 μL, 0.20 mmol), DMMS (49.3 μL, 0.40 mmol), anhydrous toluene (0.72 mL) and DMPU (0.20 mL). The reaction mixture was stirred for 24 h at rt. The crude material was purified by flash column chromatography (0–1% EtOAc in PE) to provide the title compound as a colorless liquid in 66% yield (33.9 mg) with 97:3 rr.

Rf 0.2 (2% EtOAc in PE), UV;

^{1}H NMR (500 MHz, $CDCl_3$) δ 7.20 (dd, *J* = 8.6, 5.5 Hz, 2H), 7.16 (d, *J* = 8.6 Hz, 2H), 6.99 (t, *J* = 8.7 Hz, 2H), 6.86 (d, *J* = 8.7 Hz, 2H), 3.88 (t, *J* = 7.9 Hz, 1H), 3.81 (s, 3H), 2.05 – 1.94 (m, 2H), 1.33 – 1.26 (m, 2H), 0.96 (t, *J* = 7.4 Hz, 3H);

^{13}C NMR (126 MHz, $CDCl_3$) δ 161.2 (d, *J* = 243.5 Hz), 157.9, 141.5 (d, *J* = 3.2 Hz), 137.3, 129.1 (d, *J* = 7.6 Hz), 128.7, 115.1 (d, *J* = 20.9 Hz), 113.8, 55.2, 49.4, 38.2, 21.1, 14.1;

^{19}F NMR (471 MHz, $CDCl_3$) δ –117.7;

HRMS (ESI) calcd. for $C_{17}H_{20}FO$ $[M+H]^+$ *m/z* 259.1493, found 259.1493;

IR (neat, cm^{-1}) 2957, 1506, 1124, 1037, 824;

$[\alpha]_D^{20}$ = –4.3 (*c* = 1.4, $CHCl_3$); 92% *ee*;

HPLC analysis CHIRALCEL OJ-H column, 1% EtOH in hexane, 0.5 mL/min, 220 nm UV detector, t_R (major) = 35.6 min, t_R (minor) = 40.5 min.

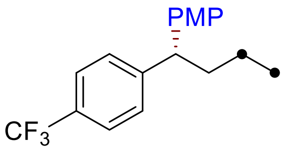

(*R*)-1-Methoxy-4-(1-(4-(trifluoromethyl)phenyl)butyl)benzene (Figure 4, **4h**). From **1-(Prop-1-en-1-yl)-4-(trifluoromethyl)benzene** (40.0 mg, 0.20 mmol), the title compound was prepared following the general procedure **A** using $NiCl_2$·glyme (2.2 mg, 5.0 mol%), **L*** (7.2 mg, 6.0 mol%), **L** [0.17 mg, 0.08 mL (2.1 mg/mL in toluene)], KF (23.2 mg, 2.0 equiv), 4-phenyl-1-butene (**1a**) (30.0 μL, 0.20 mmol), DMMS (49.3 μL, 0.40 mmol), anhydrous toluene (0.72 mL) and DMPU (0.20 mL). The reaction mixture was stirred for 24 h at rt. The crude material was purified by flash column chromatography (0–1% EtOAc in PE) to provide the title compound as a colorless liquid in 70% yield (43.6 mg) with 97:3 rr.

Rf 0.3 (2% EtOAc in PE), UV;

^{1}H NMR (500 MHz, $CDCl_3$) δ 7.55 (d, *J* = 8.2 Hz, 2H), 7.36 (d, *J* = 8.0 Hz, 2H), 7.17 (d, *J* = 8.7 Hz, 2H), 6.87 (d, *J* = 8.7 Hz, 2H), 3.96 (t, *J* = 7.8 Hz, 1H), 3.81 (s, 3H), 2.08 – 1.99 (m, 2H), 1.34 – 1.28 (m, 2H), 0.96 (t, *J* = 7.3 Hz, 3H);

^{13}C NMR (126 MHz, $CDCl_3$) δ 158.1, 149.9, 136.4, 128.8, 128.1, 125.3 (q, *J* = 3.6 Hz), 122.2 (q, *J* = 271.7 Hz), 113.9, 55.2, 50.1, 37.8, 21.1, 14.0;

^{19}F NMR (471 MHz, $CDCl_3$) δ –62.3;

HRMS (ESI) calcd. for $C_{18}H_{19}F_3NaO$ $[M+Na]^+$ *m/z* 331.1280, found 331.1283;

IR (neat, cm^{-1}) 2959, 1510, 1323, 1117, 824;

$[\alpha]_D^{20}$ = –8.9 (*c* = 1.5, $CHCl_3$); 92% *ee*;

HPLC analysis CHIRALCEL AD-H column, 1% ^{i}PrOH in hexane, 0.5 mL/min, 220 nm UV detector, t_R (minor) = 8.4 min, t_R (major) = 8.8 min.

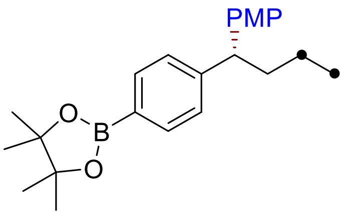

(*R*)-2-(4-(1-(4-methoxyphenyl)butyl)phenyl)-4,4,5,5-tetramethyl-1,3,2-dioxaborolane (Figure 4, **4i**). From **2-(4-(But-3-en-1-yl)phenyl)-4,4,5,5-tetramethyl-1,3,2-dioxaborolane** (51.6 mg, 0.20 mmol), the title compound was prepared following the general procedure **A** using $NiCl_2$·glyme (2.2 mg, 5.0 mol%), **L*** (7.2 mg, 6.0 mol%), **L** [0.17 mg, 0.08 mL (2.1 mg/mL in toluene)], KF (23.2 mg, 2.0 equiv), 4-phenyl-1-butene

(**1a**) (30.0 μL, 0.20 mmol), DMMS (49.3 μL, 0.40 mmol), anhydrous toluene (0.72 mL) and DMPU (0.20 mL). The reaction mixture was stirred for 24 h at rt. The crude material was purified by flash column chromatography (0–1% EtOAc in PE) to provide the title compound as a colorless liquid in 43% yield (31.4 mg) with 97:3 rr.

Rf 0.2 (2% EtOAc in PE), UV;

^{1}H NMR (500 MHz, $CDCl_3$) δ 7.76 (d, *J* = 7.9 Hz, 2H), 7.27 (d, *J* = 7.9 Hz, 2H), 7.17 (d, *J* = 8.6 Hz, 2H), 6.84 (d, *J* = 8.7 Hz, 2H), 3.91 (t, *J* = 7.8 Hz, 1H), 3.81 – 3.77 (m, 3H), 2.08 – 1.98 (m, 2H), 1.35 (s, 12H), 1.32 – 1.26 (m, 2H), 0.94 (t, *J* = 7.4 Hz, 3H);

^{13}C NMR (126 MHz, $CDCl_3$) δ 157.8, 149.1, 137.3, 134.9, 128.8, 127.3, 113.7, 83.6, 55.2, 50.4, 37.9, 24.9, 21.1, 14.1;

HRMS (ESI) calcd. for $C_{23}H_{31}BNaO_3$ $[M+Na]^+$ *m/z* 389.2258, found 389.2253;

IR (neat, cm^{-1}) 2975, 1358, 1087, 658;

$[\alpha]_D^{20}$ = –0.3 (*c* = 1.5, $CHCl_3$); 92% *ee*;

HPLC analysis CHIRALCEL ID-3 column, 3% EtOH in hexane, 0.4 mL/min, 220 nm UV detector, t_R (minor) = 10.0 min, t_R (major) = 11.1 min.

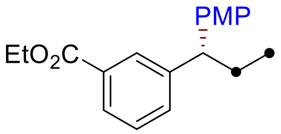

Ethyl (*S*)-3-(1-(4-methoxyphenyl)propyl)benzoate (Figure 4, **4j**). From **Ethyl 3-allylbenzoate** (38.0 mg, 0.20 mmol), the title compound was prepared following the general procedure **A** using $NiCl_2$·glyme (2.2 mg, 5.0 mol%), **L*** (7.2 mg, 6.0 mol%), **L** [0.17 mg, 0.08 mL (2.1 mg/mL in toluene)], KF (23.2 mg, 2.0 equiv), 4-phenyl-1-butene (**1a**) (30.0 μL, 0.20 mmol), DMMS (49.3 μL, 0.40 mmol), anhydrous toluene (0.72 mL) and DMPU (0.20 mL). The reaction mixture was stirred for 24 h at rt. The crude material was purified by flash column chromatography (0–5% EtOAc in PE) to provide the title compound as a colorless liquid in 73% yield (43.7 mg) with 96:4 rr.

Rf 0.4 (5% EtOAc in PE), UV;

^{1}H NMR (500 MHz, $CDCl_3$) δ 7.97 (s, 1H), 7.88 (d, *J* = 7.6 Hz, 1H), 7.42 (d, *J* = 7.8 Hz, 1H), 7.36 (t, *J* = 7.7 Hz, 1H), 7.18 (d, *J* = 8.7 Hz, 2H), 6.86 (d, *J* = 8.7 Hz, 2H), 4.39 (q, *J*

= 7.1 Hz, 2H), 3.83 (t, *J* = 7.8 Hz, 1H), 3.79 (s, 3H), 2.15 – 2.05 (m, 2H), 1.42 (t, *J* = 7.2 Hz, 3H), 0.92 (t, *J* = 7.3 Hz, 3H);

^{13}C NMR (126 MHz, $CDCl_3$) δ 166.8, 157.9, 145.9, 136.7, 132.4, 130.5, 128.9, 128.7, 128.4, 127.2, 113.8, 60.9, 55.2, 52.2, 28.6, 14.3, 12.7;

HRMS (ESI) calcd. for $C_{19}H_{22}NaO_3$ $[M+Na]^+$ *m/z* 321.1461, found 321.1463;

IR (neat, cm^{-1}) 2967, 1714, 1510, 1246, 756;

$[\alpha]_D^{20}$ = –3.2 (*c* = 1.9, $CHCl_3$); 86% *ee*;

HPLC analysis CHIRALCEL AD-H column, 5% ^{i}PrOH in hexane, 0.8 mL/min, 220 nm UV detector, t_R (minor) = 7.2 min, t_R (major) = 7.8 min.

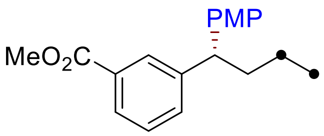

Methyl (*S*)-3-(1-(4-methoxyphenyl)butyl)benzoate (Figure 4, **4k**). From **Methyl 3-(but-3-en-1-yl)benzoate** (38.0 mg, 0.20 mmol), the title compound was prepared following the general procedure **A** using $NiCl_2$·glyme (2.2 mg, 5.0 mol%), **L*** (7.2 mg, 6.0 mol%), **L** [0.17 mg, 0.08 mL (2.1 mg/mL in toluene)], KF (23.2 mg, 2.0 equiv), 4-phenyl-1-butene (**1a**) (30.0 μL, 0.20 mmol), DMMS (49.3 μL, 0.40 mmol), anhydrous toluene (0.72 mL) and DMPU (0.20 mL). The reaction mixture was stirred for 24 h at rt. The crude material was purified by flash column chromatography (0–4% EtOAc in PE) to provide the title compound as a colorless liquid in 68% yield (40.4 mg) with 97:3 rr.

Rf 0.4 (4% EtOAc in PE), UV;

^{1}H NMR (500 MHz, $CDCl_3$) δ 7.97 (s, 1H), 7.88 (d, *J* = 7.7 Hz, 1H), 7.44 (d, *J* = 7.7 Hz, 1H), 7.36 (t, *J* = 7.7 Hz, 1H), 7.19 (d, *J* = 8.7 Hz, 2H), 6.86 (d, *J* = 8.7 Hz, 2H), 3.99 – 3.92 (m, 4H), 3.79 (s, 3H), 2.10 – 2.01 (m, 2H), 1.38 – 1.23 (m, 2H), 0.96 (t, *J* = 7.3 Hz, 3H);

^{13}C NMR (126 MHz, $CDCl_3$) δ 167.2, 158.0, 146.2, 136.8, 132.5, 130.2, 128.8, 128.7, 128.4, 127.3, 113.9, 55.1, 52.0, 50.1, 37.9, 21.1, 14.0;

HRMS (ESI) calcd. for $C_{19}H_{23}O_3$ $[M+H]^+$ *m/z* 299.1642, found 299.1637;

IR (neat, cm^{-1}) 2955, 1719, 1509, 1246, 1035, 750;

$[\alpha]_D^{20}$ = –0.4 (*c* = 2.0, $CHCl_3$); 89% *ee*;

HPLC analysis CHIRALCEL AD-H column, 5% ^{i}PrOH in hexane, 0.8 mL/min, 220 nm UV detector, t_R (minor) = 7.2 min, t_R (major) = 7.6 min.

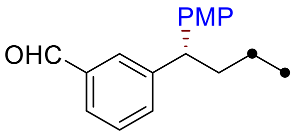

(*S*)-3-(1-(4-Methoxyphenyl)butyl)benzaldehyde (Figure 4, **4l**). From **3-(But-3-en-1-yl)benzaldehyde** (32.0 mg, 0.20 mmol), the title compound was prepared following the general procedure **A** using $NiCl_2$·glyme (2.2 mg, 5.0 mol%), **L*** (7.2 mg, 6.0 mol%), **L** [0.17 mg, 0.08 mL (2.1 mg/mL in toluene)], KF (23.2 mg, 2.0 equiv), 4-phenyl-1-butene (**1a**) (30.0 μL, 0.20 mmol), DMMS (49.3 μL, 0.40 mmol), anhydrous toluene (0.72 mL) and DMPU (0.20 mL). The reaction mixture was stirred for 24 h at rt. The crude material was purified by flash column chromatography (0–3% EtOAc in PE) to provide the title compound as a colorless liquid in 51% yield (27.5 mg) with 96:4 rr.

Rf 0.3 (4% EtOAc in PE), UV;

^{1}H NMR (500 MHz, $CDCl_3$) δ 10.00 (s, 1H), 7.78 (s, 1H), 7.70 (d, *J* = 7.4 Hz, 1H), 7.52 (d, *J* = 7.6 Hz, 1H), 7.46 (t, *J* = 7.6 Hz, 1H), 7.18 (d, *J* = 8.7 Hz, 2H), 6.86 (d, *J* = 8.7 Hz, 2H), 3.97 (t, *J* = 7.8 Hz, 1H), 3.80 (s, 3H), 2.11 – 2.00 (m, 2H), 1.37 – 1.23 (m, 2H), 0.96 (t, *J* = 7.4 Hz, 3H);

^{13}C NMR (126 MHz, $CDCl_3$) δ 192.6, 158.1, 147.0, 136.6, 136.5, 134.2, 129.1, 128.7, 128.6, 127.9, 113.9, 55.2, 49.9, 37.9, 21.1, 14.0;

HRMS (ESI) calcd. for $C_{18}H_{21}O_2$ $[M+H]^+$ *m/z* 269.1536, found 269.1534;

IR (neat, cm^{-1}) 2957, 1694, 1509, 1245, 1035;

$[\alpha]_D^{20}$ = +3.6 (*c* = 1.0, $CHCl_3$); 86% *ee*;

HPLC analysis CHIRALCEL IG-3 column, 5% ^{i}PrOH in hexane, 0.5 mL/min, 220 nm UV detector, t_R (minor) = 15.6 min, t_R (major) = 16.3 min.

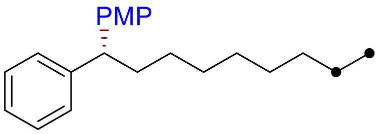

(*R*)-1-methoxy-4-(1-phenylnonyl)benzene (Figure 4, **4m**). From **Non-8-en-1-ylbenzene** (40.9 mg, 0.20 mmol), the title compound was prepared following the general procedure

A using $NiCl_2$·glyme (2.2 mg, 5.0 mol%), **L*** (7.2 mg, 6.0 mol%), **L** [0.42 mg, 0.2 mL (2.1 mg/mL in toluene)], KF (23.2 mg, 2.0 equiv), 4-iodoanisole (94.0 mg, 0.40 mmol, 2.0 equiv), DMMS (49.3 μL, 0.40 mmol), anhydrous toluene (0.60 mL) and DMPU (0.20 mL). The reaction mixture was stirred for 24 h at rt. The crude material was purified by flash column chromatography (0–1% EtOAc in PE) to provide the title compound as a colorless liquid in 38% yield (23.7 mg) with 96:4 rr.

Rf 0.2 (2% EtOAc in PE), UV;

^{1}H NMR (500 MHz, $CDCl_3$) δ 7.30 – 7.23 (m, 4H), 7.21 – 7.15 (m, 3H), 6.84 (d, *J* = 8.6 Hz, 2H), 3.86 (t, *J* = 7.7 Hz, 1H), 3.79 (s, 3H), 2.06 – 1.98 (m, 2H), 1.32 – 1.22 (m, 12H), 0.89 (t, *J* = 7.0 Hz, 3H);

^{13}C NMR (126 MHz, $CDCl_3$) δ 157.8, 145.8, 137.6, 128.7, 128.3, 127.8, 125.9, 113.7, 55.2, 50.5, 35.9, 31.9, 29.7, 29.5, 29.3, 28.1, 22.7, 14.1;

HRMS (ESI) calcd. for $C_{22}H_{30}NaO$ $[M+Na]^+$ *m/z* 333.2189, found 333.2188;

IR (neat, cm^{-1}) 2923, 1510, 1246, 1039, 698;

$[\alpha]_D^{20}$ = –4.0 (*c* = 0.5, $CHCl_3$); 96% *ee*;

HPLC analysis CHIRALCEL OJ-H*2 column, 1% EtOH in hexane, 0.5 mL/min, 220 nm UV detector, t_R (minor) = 28.6 min, t_R (major) = 30.5 min.

Note:

Using **L** [0.84 mg, 0.4 mL (2.1 mg/mL in toluene)] and anhydrous toluene (0.40 mL), the title compound was prepared following the general procedure **A**, and the crude material was purified by flash column chromatography (0–1% EtOAc in PE) to provide the title compound as a colorless liquid in 57% yield (35.2 mg) with 93% *ee*.

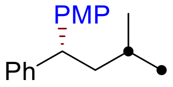

(*R*)-1-Methoxy-4-(3-methyl-1-phenylbutyl)benzene (Figure 4, **4n**)[2]. From **(3-Methylbut-3-en-1-yl)benzene** (29.4 mg, 0.20 mmol), the title compound was prepared following the general procedure **A** using $NiCl_2$·glyme (2.2 mg, 5.0 mol%), **L*** (7.2 mg, 6.0 mol%), **L** [0.42 mg, 0.2 mL (2.1 mg/mL in toluene)], KF (23.2 mg, 2.0 equiv), 4-iodoanisole (94.0 mg, 0.40 mmol, 2.0 equiv), DMMS (49.3 μL, 0.40 mmol), anhydrous toluene (0.60 mL) and DMPU (0.20 mL). The reaction mixture was stirred for 24 h at rt.

The crude material was purified by flash column chromatography (0–1% EtOAc in PE) to provide the title compound as a colorless liquid in 41% yield (20.5 mg) with 99:1 rr.

Rf 0.3 (2% EtOAc in PE), UV;

^{1}H NMR (500 MHz, $CDCl_3$) δ 7.32 – 7.25 (m, 4H), 7.19 (d, *J* = 8.6 Hz, 3H), 6.85 (d, *J* = 8.7 Hz, 2H), 4.00 (t, *J* = 8.0 Hz, 1H), 3.80 (s, 3H), 1.96 – 1.89 (m, 2H), 1.52 – 1.43 (m, 1H), 0.95 (d, *J* = 6.7 Hz, 6H);

^{13}C NMR (126 MHz, $CDCl_3$) δ 157.8, 145.8, 137.5, 128.8, 128.4, 127.8, 125.9, 113.8, 55.2, 48.0, 45.2, 25.5, 22.7, 22.6;

HRMS (ESI) calcd. for $C_{18}H_{22}NaO$ $[M+Na]^+$ *m/z* 277.1563, found 277.1564;

IR (neat, cm^{-1}) 2954, 1509, 1246, 1038, 697;

$[\alpha]_D^{20}$ = –7.5 (*c* = 1.0, $CHCl_3$); 94% *ee*;

HPLC analysis CHIRALCEL OJ-H column, 20% ^{i}PrOH in hexane, 0.5 mL/min, 220 nm UV detector, t_R (major) = 10.7 min, t_R (minor) = 11.6 min.

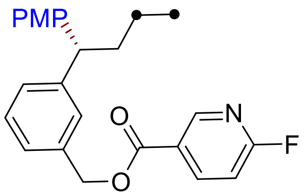

(*S*)-3-(1-(4-Methoxyphenyl)butyl)benzyl 6-fluoronicotinate (Figure 4, **4o**). From **3-(But-3-en-1-yl)benzyl 6-fluoronicotinate** (57.1 mg, 0.20 mmol), the title compound was prepared following the general procedure **A** using $NiCl_2$·glyme (2.2 mg, 5.0 mol%), **L*** (7.2 mg, 6.0 mol%), **L** [0.42 mg, 0.2 mL (2.1 mg/mL in toluene)], KF (23.2 mg, 2.0 equiv), 4-iodoanisole (94.0 mg, 0.40 mmol, 2.0 equiv), DMMS (49.3 μL, 0.40 mmol), anhydrous toluene (0.60 mL) and DMPU (0.20 mL). The reaction mixture was stirred for 24 h at rt. The crude material was purified by flash column chromatography (0–5% EtOAc in PE) to provide the title compound as a colorless liquid in 79% yield (61.9 mg) with 98:2 rr.

Rf 0.5 (20% EtOAc in PE), UV;

^{1}H NMR (500 MHz, $CDCl_3$) δ 8.93 (s, 1H), 8.46 – 8.38 (m, 1H), 7.37 – 7.24 (m, 4H), 7.19 (d, *J* = 8.7 Hz, 2H), 7.02 (dd, *J* = 8.5, 2.8 Hz, 1H), 6.86 (d, *J* = 8.7 Hz, 2H), 5.37 (s,

2H), 3.93 (t, J = 7.8 Hz, 1H), 3.80 (s, 3H), 2.09 – 1.98 (m, 2H), 1.37 – 1.28 (m, 2H), 0.96 (t, J = 7.3 Hz, 3H);

^{13}C NMR (126 MHz, $CDCl_3$) δ 165.9 (d, J = 245.7 Hz), 164.1, 157.9, 150.5 (d, J = 16.3 Hz), 146.5, 142.7 (d, J = 9.3 Hz), 137.0, 135.3, 128.8, 128.8, 127.9, 127.8, 125.9, 124.5 (d, J = 4.5 Hz), 113.8, 109.6 (d, J = 37.5 Hz), 67.1, 55.2, 50.1, 38.1, 21.2, 14.1;

^{19}F NMR (471 MHz, $CDCl_3$) δ –61.1;

HRMS (ESI) calcd. for $C_{24}H_{24}FNNaO_3$ $[M+Na]^+$ *m/z* 416.1632, found 416.1633;

IR (neat, cm^{-1}) 2958, 1721, 1510, 1246, 1109, 775;

$[\alpha]_D^{20}$ = –6.5 (c = 2.3, $CHCl_3$); 92% *ee*;

HPLC analysis CHIRALCEL OJ-H column, 10% ^{i}PrOH in hexane, 0.8 mL/min, 220 nm UV detector, t_R (major) = 30.7 min, t_R (minor) = 35.1 min.

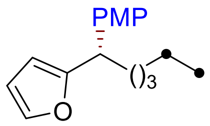

(*S*)-2-(1-(4-Methoxyphenyl)hexyl)furan (Figure 4, **4p**). From **2-(Hex-5-en-1-yl)furan** (30.0 mg, 0.20 mmol), the title compound was prepared following the general procedure **A** using $NiCl_2$·glyme (2.2 mg, 5.0 mol%), **L*** (7.2 mg, 6.0 mol%), **L** [0.17 mg, 0.08 mL (2.1 mg/mL in toluene)], KF (23.2 mg, 2.0 equiv), 4-phenyl-1-butene (**1a**) (30.0 μL, 0.20 mmol), DMMS (49.3 μL, 0.40 mmol), anhydrous toluene (0.72 mL) and DMPU (0.20 mL). The reaction mixture was stirred for 24 h at rt. The crude material was purified by flash column chromatography (0–2% EtOAc in PE) to provide the title compound as a colorless liquid in 42% yield (21.9 mg) with 96:4 rr.

Rf 0.4 (5% EtOAc in PE), UV;

^{1}H NMR (500 MHz, $CDCl_3$) δ 7.33 (s, 1H), 7.17 (d, J = 8.7 Hz, 2H), 6.87 (d, J = 8.7 Hz, 2H), 6.30 (dd, J = 3.2, 1.9 Hz, 1H), 6.05 (d, J = 3.1 Hz, 1H), 3.89 (t, J = 7.7 Hz, 1H), 3.81 (s, 3H), 2.14 – 2.04 (m, 1H), 1.92 – 1.83 (m, 1H), 1.34 – 1.27 (m, 6H), 0.89 (t, J = 6.7 Hz, 3H);

^{13}C NMR (126 MHz, $CDCl_3$) δ 158.7, 158.1, 141.1, 135.1, 128.8, 113.8, 113.7, 109.9, 104.9, 55.2, 44.5, 34.8, 31.7, 27.3, 22.5, 14.1;

HRMS (ESI) calcd. for $C_{17}H_{22}NaO_2$ $[M+Na]^+$ m/z 281.1512, found 281.1511;

IR (neat, cm^{-1}) 2926, 1510, 1244, 1037, 726;

[α]$_D^{20}$ = +35.3 (*c* = 0.9, $CHCl_3$); 90% *ee*;

HPLC analysis CHIRALCEL OJ-H column, 20% ^{i}PrOH in hexane, 0.5 mL/min, 220 nm UV detector, t_R (minor) = 11.7 min, t_R (major) = 13.5 min.

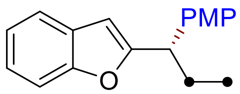

(*S*)-2-(1-(4-Methoxyphenyl)propyl)benzofuran (Figure 4, **4q**). From **2-Allylbenzofuran** (31.6 mg, 0.20 mmol), the title compound was prepared following the general procedure **A** using $NiCl_2$·glyme (2.2 mg, 5.0 mol%), **L*** (7.2 mg, 6.0 mol%), **L** [0.17 mg, 0.08 mL (2.1 mg/mL in toluene)], KF (23.2 mg, 2.0 equiv), 4-phenyl-1-butene (**1a**) (30.0 μL, 0.20 mmol), DMMS (49.3 μL, 0.40 mmol), anhydrous toluene (0.72 mL) and DMPU (0.20 mL). The reaction mixture was stirred for 24 h at rt. The crude material was purified by flash column chromatography (0–3% EtOAc in PE) to provide the title compound as a colorless liquid in 52% yield (27.9 mg) with 97:3 rr.

Rf 0.4 (5% EtOAc in PE), UV;

^{1}H NMR (500 MHz, $CDCl_3$) δ 7.53 (d, *J* = 6.8 Hz, 1H), 7.44 (d, *J* = 7.7 Hz, 1H), 7.27 (d, *J* = 8.6 Hz, 2H), 7.24 – 7.19 (m, 2H), 6.90 (d, *J* = 8.7 Hz, 2H), 6.47 (s, 1H), 3.95 (t, *J* = 7.7 Hz, 1H), 3.83 (s, 3H), 2.33 – 2.24 (m, 1H), 2.06 – 1.96 (m, 1H), 0.99 (t, *J* = 7.4 Hz, 3H);

^{13}C NMR (126 MHz, $CDCl_3$) δ 161.7, 158.4, 154.8, 134.3, 129.0, 128.7, 123.3, 122.4, 120.4, 113.9, 110.9, 102.2, 55.3, 46.7, 27.7, 12.4;

HRMS (ESI) calcd. for $C_{18}H_{19}O_2$ $[M+H]^+$ *m/z* 267.1380, found 267.1381;

IR (neat, cm^{-1}) 2966, 1510, 1247, 1037, 740;

[α]$_D^{20}$ = +33.7 (*c* = 1.3, $CHCl_3$); 84% *ee*;

HPLC analysis CHIRALCEL OJ-H column, 20% ^{i}PrOH in hexane, 0.5 mL/min, 220 nm UV detector, t_R (minor) = 16.7 min, t_R (major) = 20.8 min.

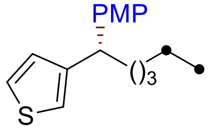

(*S*)-3-(1-(4-Methoxyphenyl)hexyl)thiophene (Figure 4, **4r**)[1]. From **3-(Hex-5-en-1-yl)thiophene** (33.3 mg, 0.20 mmol), the title compound was prepared following the general procedure **A** using $NiCl_2$·glyme (2.2 mg, 5.0 mol%), **L*** (7.2 mg, 6.0 mol%), **L** [0.42 mg, 0.2 mL (2.1 mg/mL in toluene)], KF (23.2 mg, 2.0 equiv), 4-iodoanisole (94.0 mg, 0.40 mmol, 2.0 equiv), DMMS (49.3 μL, 0.40 mmol), anhydrous toluene (0.60 mL) and DMPU (0.20 mL). The reaction mixture was stirred for 24 h at rt. The crude material was purified by flash column chromatography (0–1% EtOAc in PE) to provide the title compound as a colorless liquid in 52% yield (28.4 mg) with 98:2 rr.

Rf 0.2 (2% EtOAc in PE), UV;

^{1}H NMR (500 MHz, $CDCl_3$) δ 7.25 (dd, *J* = 5.0, 2.9 Hz, 1H), 7.16 (d, *J* = 8.7 Hz, 2H), 6.99 (d, *J* = 2.9 Hz, 1H), 6.93 (d, *J* = 3.6 Hz, 1H), 6.87 (d, *J* = 8.7 Hz, 2H), 3.93 (t, *J* = 7.7 Hz, 1H), 3.82 (s, 3H), 2.12 – 2.01 (m, 1H), 2.01 – 1.91 (m, 1H), 1.35 – 1.27 (m, 6H), 0.90 (t, *J* = 6.7 Hz, 3H);

^{13}C NMR (126 MHz, $CDCl_3$) δ 157.9, 146.9, 137.2, 128.7, 127.8, 125.3, 119.7, 113.7, 55.2, 46.1, 36.3, 31.8, 27.6, 22.6, 14.1;

HRMS (ESI) calcd. for $C_{17}H_{22}NaOS$ $[M+Na]^+$ *m/z* 297.1284, found 297.1280;

IR (neat, cm^{-1}) 2925, 1609, 1509, 1243, 1037, 827;

$[\alpha]_D^{20}$ = +44.2 (*c* = 1.1, $CHCl_3$); 94% *ee*;

HPLC analysis CHIRALCEL OJ-H column, 30% ^{i}PrOH in hexane, 0.8 mL/min, 220 nm UV detector, t_R (minor) = 7.7 min, t_R (major) = 9.5 min.

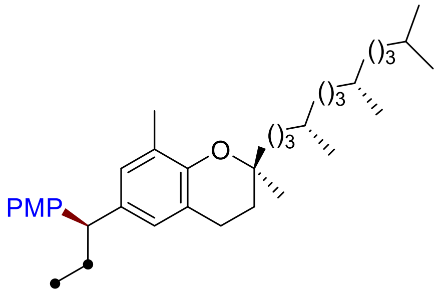

(*S*)-6-((*S*)-1-(4-Methoxyphenyl)propyl)-2,8-dimethyl-2-((4*S*,8*S*)-4,8,12-trimethyltridecyl)chromane (Figure 4, **4s**). From **(*S*)-6-Allyl-2,8-dimethyl-2-((4*S*,8*S*)-4,8,12-trimethyltridecyl)chromane** (85.3 mg, 0.20 mmol), the title compound was prepared following the general procedure **A** using $NiCl_2$·glyme (2.2 mg, 5.0 mol%), **L***

(7.2 mg, 6.0 mol%), **L** [0.42 mg, 0.2 mL (2.1 mg/mL in toluene)], KF (23.2 mg, 2.0 equiv), 4-iodoanisole (94.0 mg, 0.40 mmol, 2.0 equiv), DMMS (49.3 μL, 0.40 mmol), anhydrous toluene (0.60 mL) and DMPU (0.20 mL). The reaction mixture was stirred for 24 h at rt. The crude material was purified by flash column chromatography (0–1% EtOAc in PE) to provide the title compound as a colorless liquid in 88% yield (94.0 mg) with >99:1 rr.

Rf 0.2 (2% EtOAc in PE), UV;

^{1}H NMR (500 MHz, $CDCl_3$) δ 7.18 (d, *J* = 8.6 Hz, 2H), 6.85 (d, *J* = 8.6 Hz, 2H), 6.82 (s, 1H), 6.75 (s, 1H), 3.80 (s, 3H), 3.62 (t, *J* = 7.8 Hz, 1H), 2.76 – 2.68 (m, 2H), 2.14 (s, 3H), 2.05 – 1.96 (m, 2H), 1.81 (dt, *J* = 13.8, 7.0 Hz, 1H), 1.74 (dt, *J* = 13.2, 6.4 Hz, 1H), 1.60 – 1.55 (m, 3H), 1.48 – 1.35 (m, 5H), 1.33 – 1.26 (m, 9H), 1.21 – 1.05 (m, 7H), 0.90 (d, *J* = 6.7 Hz, 9H), 0.88 (d, *J* = 2.6 Hz, 3H), 0.87 (d, *J* = 2.8 Hz, 3H);

^{13}C NMR (126 MHz, $CDCl_3$) δ 157.6, 150.3, 138.2, 135.7, 128.7, 127.7, 125.8, 119.9, 113.6, 75.8, 55.2, 51.7, 40.3, 39.4, 37.5, 37.4, 37.3, 32.8, 32.7, 31.3, 29.1, 28.0, 24.8, 24.5, 24.3, 24.3, 22.8, 22.7, 22.5, 21.0, 19.8, 19.7, 16.2, 13.0;

HRMS (ESI) calcd. for $C_{37}H_{58}NaO_2$ $[M+Na]^+$ *m/z* 557.4329, found 557.4332;

IR (neat, cm^{-1}) 2923, 1510, 1244, 1041, 828;

$[\alpha]_D^{20}$ = +6.5 (*c* = 4.5, $CHCl_3$); >20:1 dr.

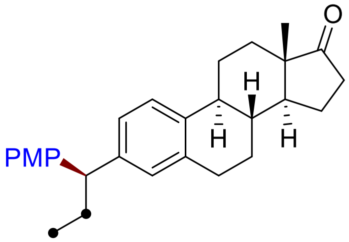

(8*R*,9*S*,13*S*,14*S*)-3-((*S*)-1-(4-Methoxyphenyl)propyl)-13-methyl-6,7,8,9,11,12,13,14,15,16-decahydro-17*H*-cyclopenta[*a*]phenanthren-17-one (Figure 4, **4t**). From **(8*R*,9*S*,13*S*,14*S*)-3-Allyl-13-methyl-6,7,8,9,11,12,13,14,15,16-decahydro-17*H*-cyclopenta[*a*]phenanthren-17-one** (58.9 mg, 0.20 mmol), the title compound was prepared following the general procedure **A** using $NiCl_2$·glyme (2.2 mg, 5.0 mol%), **L*** (7.2 mg, 6.0 mol%), **L** [0.17 mg, 0.08 mL (2.1 mg/mL in toluene)], KF (23.2 mg, 2.0 equiv), 4-phenyl-1-butene (**1a**) (30.0 μL, 0.20 mmol), DMMS (49.3 μL, 0.40 mmol),

anhydrous toluene (0.72 mL) and DMPU (0.20 mL). The reaction mixture was stirred for 24 h at rt. The crude material was purified by flash column chromatography (0–1% EtOAc in PE) to provide the title compound as a white solid in 82% yield (66.3 mg) with >99:1 rr.

Rf 0.2 (2% EtOAc in PE), UV;

^{1}H NMR (500 MHz, $CDCl_3$) δ 7.22 (d, *J* = 8.1 Hz, 1H), 7.19 (d, *J* = 8.7 Hz, 2H), 7.05 (d, *J* = 8.1 Hz, 1H), 6.97 (s, 1H), 6.86 (d, *J* = 8.7 Hz, 2H), 3.80 (s, 3H), 3.71 (t, *J* = 7.8 Hz, 1H), 2.94 – 2.87 (m, 2H), 2.53 (dd, *J* = 19.0, 8.7 Hz, 1H), 2.47 – 2.39 (m, 1H), 2.29 (t, *J* = 13.0 Hz, 1H), 2.21 – 2.13 (m, 1H), 2.11 – 2.00 (m, 4H), 1.98 (d, *J* = 11.8 Hz, 1H), 1.69 – 1.58 (m, 2H), 1.58 – 1.39 (m, 4H), 0.97 – 0.87 (m, 6H);

^{13}C NMR (126 MHz, $CDCl_3$) δ 221.1, 157.8, 143.1, 137.4, 137.3, 136.3, 128.8, 128.4, 125.3, 125.1, 113.8, 55.2, 52.0, 50.6, 48.0, 44.3, 38.2, 35.9, 31.6, 29.7, 29.5, 28.8, 26.6, 25.7, 21.6, 13.9, 12.9;

HRMS (ESI) calcd. for $C_{28}H_{34}NaO_2$ $[M+Na]^+$ *m/z* 425.2451, found 425.2451;

IR (neat, cm^{-1}) 3674, 2920, 1734, 1250, 1051;

m.p. 103.8 – 105.3 °C;

$[\alpha]_D^{20}$ = +123.5 (*c* = 2.1, $CHCl_3$); >20:1 dr;

HPLC analysis CHIRALCEL AD-H column, 10% EtOH in hexane, 1.0 mL/min, 220 nm UV detector, t_R (minor) = 8.4 min, t_R (major) = 11.0 min.

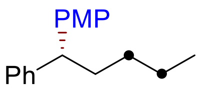

(*R*)-1-Methoxy-4-(1-phenylpentyl)benzene (Figure 4, **4u**). From **Pent-3-en-1-ylbenzene** (29.2 mg, 0.20 mmol), the title compound was prepared following the general procedure **A** using $NiCl_2$·glyme (2.2 mg, 5.0 mol%), **L*** (7.2 mg, 6.0 mol%), **L** [0.42 mg, 0.2 mL (2.1 mg/mL in toluene)], KF (23.2 mg, 2.0 equiv), 4-iodoanisole (94.0 mg, 0.40 mmol, 2.0 equiv), DMMS (49.3 μL, 0.40 mmol), anhydrous toluene (0.60 mL) and DMPU (0.20 mL). The reaction mixture was stirred for 24 h at rt. The crude material was purified by flash column chromatography (0–1% EtOAc in PE) to provide the title compound as a colorless liquid in 80% yield (40.8 mg) with >99:1 rr.

Rf 0.2 (2% EtOAc in PE), UV;

^{1}H NMR (500 MHz, $CDCl_3$) δ 7.34 – 7.25 (m, 4H), 7.25 – 7.16 (m, 3H), 6.87 (d, J = 8.6 Hz, 2H), 3.89 (t, J = 7.8 Hz, 1H), 3.81 (s, 3H), 2.10 – 2.02 (m, 2H), 1.43 – 1.35 (m, 2H), 1.32 – 1.26 (m, 2H), 0.92 (t, J = 7.3 Hz, 3H);

^{13}C NMR (126 MHz, $CDCl_3$) δ 157.8, 145.8, 137.6, 128.8, 128.4, 127.8, 125.9, 113.8, 55.2, 50.5, 35.7, 30.3, 22.8, 14.1;

HRMS (ESI) calcd. for $C_{18}H_{23}O$ $[M+H]^+$ *m/z* 255.1743, found 255.1738;

IR (neat, cm^{-1}) 2927, 1322, 1116, 1017, 698;

$[\alpha]_D^{20}$ = –5.9 (c = 1.6, $CHCl_3$); 95% *ee*;

HPLC analysis CHIRALCEL OJ-H column, 1% EtOH in hexane, 0.5 mL/min, 220 nm UV detector, t_R (minor) = 20.4 min, t_R (major) = 22.4 min.

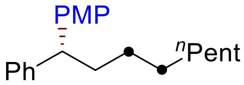

(*R*)-1-Methoxy-4-(1-phenylnonyl)benzene (Figure 4, **4v**)[1]. From **Non-3-en-1-ylbenzene** (40.9 mg, 0.20 mmol), the title compound was prepared following the general procedure **A** using $NiCl_2$·glyme (2.2 mg, 5.0 mol%), **L*** (7.2 mg, 6.0 mol%), **L** [0.42 mg, 0.2 mL (2.1 mg/mL in toluene)], KF (23.2 mg, 2.0 equiv), 4-iodoanisole (94.0 mg, 0.40 mmol, 2.0 equiv), DMMS (49.3 μL, 0.40 mmol), anhydrous toluene (0.60 mL) and DMPU (0.20 mL). The reaction mixture was stirred for 24 h at rt. The crude material was purified by flash column chromatography (0–1% EtOAc in PE) to provide the title compound as a colorless liquid in 58% yield (36.2 mg) with >99:1 rr.

Rf 0.2 (2% EtOAc in PE), UV;

^{1}H NMR (500 MHz, $CDCl_3$) δ 7.31 (t, J = 7.5 Hz, 2H), 7.26 (d, J = 6.7 Hz, 2H), 7.23 – 7.16 (m, 3H), 6.87 (d, J = 8.7 Hz, 2H), 3.88 (t, J = 7.8 Hz, 1H), 3.81 (s, 3H), 2.09 – 2.01 (m, 2H), 1.38 – 1.25 (m, 12H), 0.92 (t, J = 7.0 Hz, 3H);

^{13}C NMR (126 MHz, $CDCl_3$) δ 157.8, 145.8, 137.6, 128.8, 128.4, 127.8, 125.9, 113.7, 55.2, 50.5, 35.9, 31.9, 29.7, 29.5, 29.4, 28.1, 22.7, 14.2;

HRMS (ESI) calcd. for $C_{22}H_{30}NaO$ $[M+Na]^+$ *m/z* 333.2189, found 333.2191;

IR (neat, cm^{-1}) 2923, 1509, 1245, 1038, 697;

$[\alpha]_D^{20}$ = –4.4 (c = 0.7, $CHCl_3$); 95% *ee*;

HPLC analysis CHIRALCEL OJ-H column, 1% EtOH in hexane, 0.5 mL/min, 220 nm UV detector, t_R (minor) = 28.0 min, t_R (major) = 30.1 min.

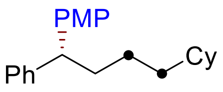

(*R*)-1-(4-Cyclohexyl-1-phenylbutyl)-4-methoxybenzene (Figure 4, **4w**). From **(4-Cyclohexylbut-3-en-1-yl)benzene** (42.8 mg, 0.20 mmol), the title compound was prepared following the general procedure **A** using $NiCl_2$·glyme (2.2 mg, 5.0 mol%), **L*** (7.2 mg, 6.0 mol%), **L** [0.42 mg, 0.2 mL (2.1 mg/mL in toluene)], KF (23.2 mg, 2.0 equiv), 4-iodoanisole (94.0 mg, 0.40 mmol, 2.0 equiv), DMMS (49.3 μL, 0.40 mmol), anhydrous toluene (0.60 mL) and DMPU (0.20 mL). The reaction mixture was stirred for 24 h at rt. The crude material was purified by flash column chromatography (0–1% EtOAc in PE) to provide the title compound as a white solid in 61% yield (39.5 mg) with >99:1 rr.

Rf 0.2 (2% EtOAc in PE), UV;

^{1}H NMR (500 MHz, $CDCl_3$) δ 7.35 – 7.25 (m, 4H), 7.20 (d, *J* = 8.4 Hz, 3H), 6.87 (d, *J* = 8.4 Hz, 2H), 3.89 (t, *J* = 7.8 Hz, 1H), 3.81 (s, 3H), 2.06 – 1.99 (m, 2H), 1.72 – 1.64 (m, 5H), 1.32 – 1.17 (m, 8H), 0.90 – 0.81 (m, 2H);

^{13}C NMR (126 MHz, $CDCl_3$) δ 157.8, 145.8, 137.6, 128.8, 128.4, 127.8, 125.9, 113.8, 113.7, 55.2, 50.6, 37.6, 37.5, 36.2, 33.5, 26.8, 26.5, 25.3;

HRMS (ESI) calcd. for $C_{23}H_{31}O$ $[M+H]^+$ *m/z* 323.2369, found 323.2365;

IR (neat, cm^{-1}) 2920, 1509, 1246, 1037, 697;

m.p. 59.1 – 60.0 °C;

$[\alpha]_D^{20}$ = –3.8 (*c* = 1.7, $CHCl_3$); 96% *ee*;

HPLC analysis CHIRALCEL OJ-H*2 column, 1% EtOH in hexane, 0.5 mL/min, 220 nm UV detector, t_R (minor) = 30.5 min, t_R (major) = 32.3 min.

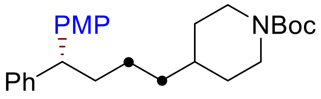

***tert*-Butyl (*R*)-4-(4-(4-methoxyphenyl)-4-phenylbutyl)piperidine-1-carboxylate** (Figure 4, **4x**). From ***tert*-Butyl 4-(4-phenylbut-1-en-1-yl)piperidine-1-carboxylate**

(63.0 mg, 0.20 mmol), the title compound was prepared following the general procedure **A** using $NiCl_2$·glyme (2.2 mg, 5.0 mol%), **L*** (7.2 mg, 6.0 mol%), **L** [0.42 mg, 0.2 mL (2.1 mg/mL in toluene)], KF (23.2 mg, 2.0 equiv), 4-iodoanisole (94.0 mg, 0.40 mmol, 2.0 equiv), DMMS (49.3 μL, 0.40 mmol), anhydrous toluene (0.60 mL) and DMPU (0.20 mL). The reaction mixture was stirred for 24 h at rt. The crude material was purified by flash column chromatography (0–2% EtOAc in PE) to provide the title compound as a white solid in 75% yield (63.9 mg) with >99:1 rr.

Rf 0.3 (4% EtOAc in PE), UV;

^{1}H NMR (500 MHz, $CDCl_3$) δ 7.29 (t, *J* = 7.5 Hz, 2H), 7.24 (d, *J* = 6.7 Hz, 2H), 7.18 (dd, *J* = 10.9, 7.9 Hz, 3H), 6.85 (d, *J* = 8.7 Hz, 2H), 4.06 (s, 2H), 3.86 (t, *J* = 7.8 Hz, 1H), 3.79 (s, 3H), 2.73 – 2.55 (m, 2H), 2.06 – 1.97 (m, 2H), 1.63 – 1.57 (m, 2H), 1.47 (s, 9H), 1.37 – 1.27 (m, 5H), 1.10 – 0.98 (m, 2H);

^{13}C NMR (126 MHz, $CDCl_3$) δ 157.8, 154.9, 145.6, 137.3, 128.7, 128.4, 127.7, 125.9, 113.8, 79.2, 55.2, 50.5, 44.4, 43.7, 36.5, 36.0, 35.8, 28.5, 25.1;

HRMS (ESI) calcd. for $C_{27}H_{37}NNaO_3$ $[M+Na]^+$ *m/z* 446.2666, found 446.2666;

IR (neat, cm^{-1}) 3674, 2987, 1681, 1408, 1066;

m.p. 75.1 – 78.6 °C;

$[\alpha]_D^{20}$ = –1.7 (*c* = 2.5, $CHCl_3$); 96% *ee*;

HPLC analysis CHIRALCEL IC column, 5% ^{i}PrOH in hexane, 0.8 mL/min, 220 nm UV detector, t_R (minor) = 20.8 min, t_R (major) = 22.4 min.

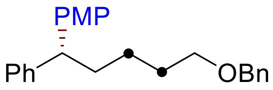

(*R*)-1-(5-(Benzyloxy)-1-phenylpentyl)-4-methoxybenzene (Figure 4, **4y**). From **(*E*)-(5-(Benzyloxy)pent-3-en-1-yl)benzene** (50.4 mg, 0.20 mmol), the title compound was prepared following the general procedure **A** using $NiCl_2$·glyme (2.2 mg, 5.0 mol%), **L*** (7.2 mg, 6.0 mol%), **L** [0.42 mg, 0.2 mL (2.1 mg/mL in toluene)], KF (23.2 mg, 2.0 equiv), 4-iodoanisole (94.0 mg, 0.40 mmol, 2.0 equiv), DMMS (49.3 μL, 0.40 mmol), anhydrous toluene (0.60 mL) and DMPU (0.20 mL). The reaction mixture was stirred for 24 h at rt. The crude material was purified by flash column chromatography (0–2%

EtOAc in PE) to provide the title compound as a colorless liquid in 37% yield (26.6 mg) with >99:1 rr.

Rf 0.4 (5% EtOAc in PE), UV;

^{1}H NMR (500 MHz, $CDCl_3$) δ 7.39 – 7.32 (m, 5H), 7.29 (d, *J* = 8.0 Hz, 2H), 7.25 (d, *J* = 7.0 Hz, 2H), 7.19 (dd, *J* = 11.0, 7.9 Hz, 3H), 6.85 (d, *J* = 8.7 Hz, 2H), 4.50 (s, 2H), 3.88 (t, *J* = 7.8 Hz, 1H), 3.80 (s, 3H), 3.47 (t, *J* = 6.6 Hz, 2H), 2.11 – 2.02 (m, 2H), 1.74 – 1.64 (m, 2H), 1.43 – 1.33 (m, 2H);

^{13}C NMR (126 MHz, $CDCl_3$) δ 157.8, 145.6, 138.6, 137.3, 128.8, 128.4, 128.4, 127.8, 127.7, 127.5, 125.9, 113.8, 72.9, 70.3, 55.2, 50.5, 35.7, 29.7, 24.7;

HRMS (ESI) calcd. for $C_{25}H_{29}O_2$ $[M+H]^+$ *m/z* 361.2162, found 361.2160;

IR (neat, cm^{-1}) 2931, 1509, 1245, 1036, 696;

$[\alpha]_D^{20}$ = –12.3 (*c* = 0.7, $CHCl_3$); 93% *ee*;

HPLC analysis CHIRALCEL IG*2 column, 1% ^{i}PrOH in hexane, 0.5 mL/min, 220 nm UV detector, t_R (minor) = 42.2 min, t_R (major) = 43.8 min.

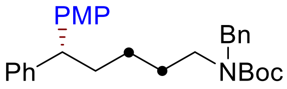

tert*-Butyl (*R*)-benzyl(5-(4-methoxyphenyl)-5-phenylpentyl)carbamate** (Figure 4, **4z**). From ***tert*-Butyl (*E*)-benzyl(5-phenylpent-2-en-1-yl)carbamate** (70.2 mg, 0.20 mmol), the title compound was prepared following the general procedure **A** using $NiCl_2$·glyme (2.2 mg, 5.0 mol%), **L (7.2 mg, 6.0 mol%), **L** [0.42 mg, 0.2 mL (2.1 mg/mL in toluene)], KF (23.2 mg, 2.0 equiv), 4-iodoanisole (94.0 mg, 0.40 mmol, 2.0 equiv), DMMS (49.3 μL, 0.40 mmol), anhydrous toluene (0.60 mL) and DMPU (0.20 mL). The reaction mixture was stirred for 24 h at rt. The crude material was purified by flash column chromatography (0–5% EtOAc in PE) to provide the title compound as a colorless liquid in 51% yield (47.3 mg) with >99:1 rr.

Rf 0.2 (5% EtOAc in PE), UV;

^{1}H NMR (500 MHz, $CDCl_3$) δ 7.35 (t, *J* = 7.3 Hz, 2H), 7.32 – 7.27 (m, 3H), 7.25 – 7.22 (m, 3H), 7.20 (d, *J* = 7.3 Hz, 1H), 7.17 (d, *J* = 8.6 Hz, 2H), 6.86 (d, *J* = 8.5 Hz, 2H), 4.43 (d, *J* = 25.0 Hz, 2H), 3.84 (t, *J* = 7.8 Hz, 1H), 3.80 (d, *J* = 1.6 Hz, 3H), 3.17 (d, *J* = 45.9 Hz, 2H), 2.07 – 1.99 (m, 2H), 1.63 – 1.39 (m, 12H), 1.24 (s, 2H);

^{13}C NMR (126 MHz, $CDCl_3$) δ 157.9, 156.1, 155.6, 145.5, 138.8, 137.2, 128.7, 128.5, 128.4, 127.7, 127.1, 126.0, 113.8, 79.6, 55.2, 50.5, 49.8, 46.7&46.4 (*due to rotamer*), 35.6, 28.5, 28.2&27.9 (*due to rotamer*), 25.4;

HRMS (ESI) calcd. for $C_{30}H_{37}NNaO_3$ $[M+Na]^+$ *m/z* 482.2666, found 482.2668;

IR (neat, cm^{-1}) 2972, 1687, 1510, 1244, 698;

$[\alpha]_D^{20}$ = –2.9 (*c* = 2.3, $CHCl_3$); 93% *ee*;

HPLC analysis CHIRALCEL OD-H column, 10% ^{i}PrOH in hexane, 0.8 mL/min, 220 nm UV detector, t_R (minor) = 7.7 min, t_R (major) = 8.5 min.

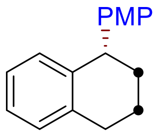

(*S*)-1-(4-Methoxyphenyl)-1,2,3,4-tetrahydronaphthalene (Figure 4, **4a**')[1]. From **1,4-Dihydronaphthalene** (26.0 mg, 0.20 mmol), the title compound was prepared following the general procedure **A** using $NiCl_2$·glyme (2.2 mg, 5.0 mol%), **L*** (7.2 mg, 6.0 mol%), **L** [0.42 mg, 0.2 mL (2.1 mg/mL in toluene)], KF (23.2 mg, 2.0 equiv), 4-iodoanisole (94.0 mg, 0.40 mmol, 2.0 equiv), DMMS (49.3 μL, 0.40 mmol), anhydrous toluene (0.60 mL) and DMPU (0.20 mL). The reaction mixture was stirred for 24 h at rt. The crude material was purified by flash column chromatography (0–1% EtOAc in PE) to provide the title compound as a colorless liquid in 62% yield (29.7 mg) with >99:1 rr.

Rf 0.2 (2% EtOAc in PE), UV;

^{1}H NMR (500 MHz, $CDCl_3$) δ 7.16 (d, *J* = 6.7 Hz, 2H), 7.06 (d, *J* = 8.7 Hz, 3H), 6.88 (dd, *J* = 11.8, 8.4 Hz, 3H), 4.11 (t, *J* = 6.7 Hz, 1H), 3.83 (s, 3H), 3.00 – 2.83 (m, 2H), 2.22 – 2.13 (m, 1H), 1.96 – 1.85 (m, 2H), 1.83 – 1.75 (m, 1H);

^{13}C NMR (126 MHz, $CDCl_3$) δ 157.8, 139.8, 139.7, 137.6, 130.2, 129.7, 128.9, 125.8, 125.6, 113.6, 55.3, 44.8, 33.4, 29.8, 21.0;

HRMS (ESI) calcd. for $C_{17}H_{18}NaO$ $[M+Na]^+$ *m/z* 261.1250, found 261.1252;

IR (neat, cm^{-1}) 3674, 2987, 1510, 1243, 1066, 740;

$[\alpha]_D^{20}$ = +23.8 (*c* = 0.5, $CHCl_3$); 90% *ee*;

HPLC analysis CHIRALCEL OJ-H column, 1% EtOH in hexane, 0.5 mL/min, 220 nm UV detector, t_R (major) = 23.5 min, t_R (minor) = 27.1 min.

2.2. Suboptimal Substrate

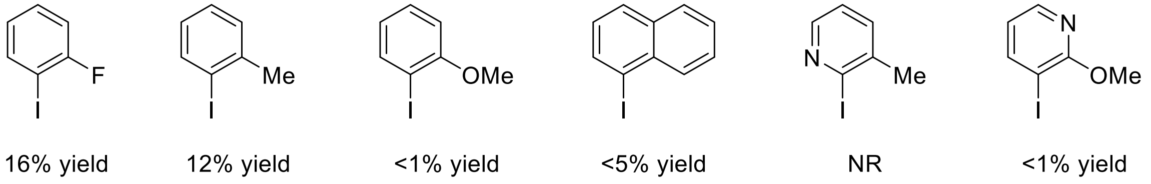

2.3. Crystal Data and Structure Refinement for 4t

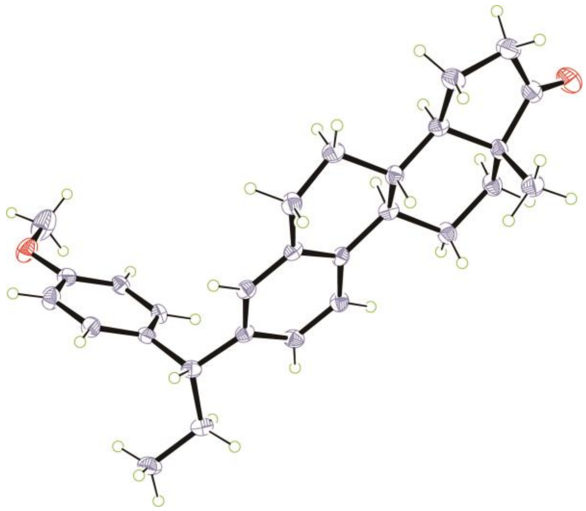

Supplementary Fig. 1. Crystal data and structure refinement for **4t**

Identification code	**4t**_a
Empirical formula	$C_{28}H_{34}O_2$
Formula weight	402.55
Temperature/K	193
Crystal system	monoclinic
Space group	P21
a/Å	6.8064(14)
b/Å	7.3932(12)
c/Å	22.709(5)
α/°	90
β/°	98.375(8)

γ/°	90
Volume/Å3	1130.6(4)
Z	2
ρcalcg/cm3	1.183
μ/mm-1	0.072
F(000)	436.0
Crystal size/mm3	0.18 × 0.14 × 0.12
Radiation	MoKα (λ = 0.71073)
2Θ range for data collection/°	5.802 to 53.82
Index ranges	-8 ≤ h ≤ 8, -9 ≤ k ≤ 9, -23 ≤ l ≤ 28
Reflections collected	9271
Independent reflections	4542 [Rint = 0.0492, Rsigma = 0.0682]
Data/restraints/parameters	4542/1/274
Goodness-of-fit on F2	1.072
Final R indexes [I>=2σ (I)]	R1 = 0.0584, wR2 = 0.1245
Final R indexes [all data]	R1 = 0.0809, wR2 = 0.1480
Largest diff. peak/hole / e Å-3	0.26/-0.30
Flack parameter	-0.3(10)

2.4. GC Trace and GC-MS Analysis of 3a

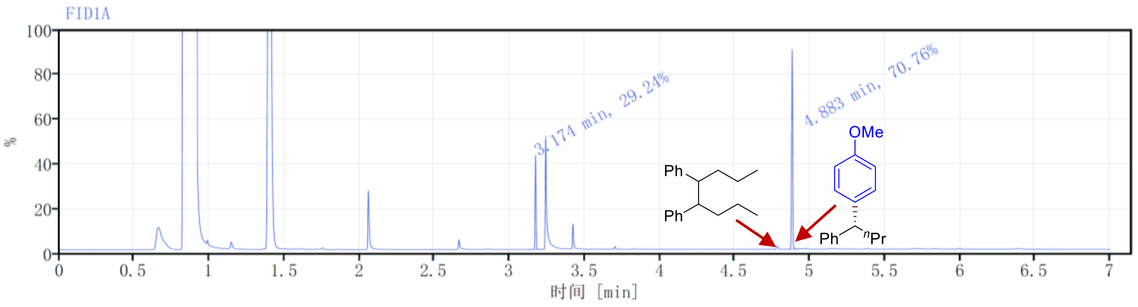

RetTime (min)	Signal	Width(min)	Area	Height	Area%
3.174	FID1A	0.083	11.0	25.7	29.24
4.883	FID1A	0.093	26.6	54.9	70.76
		Total FID1A	37.6		

Supplementary Fig. 2. GC Trace of **3a**

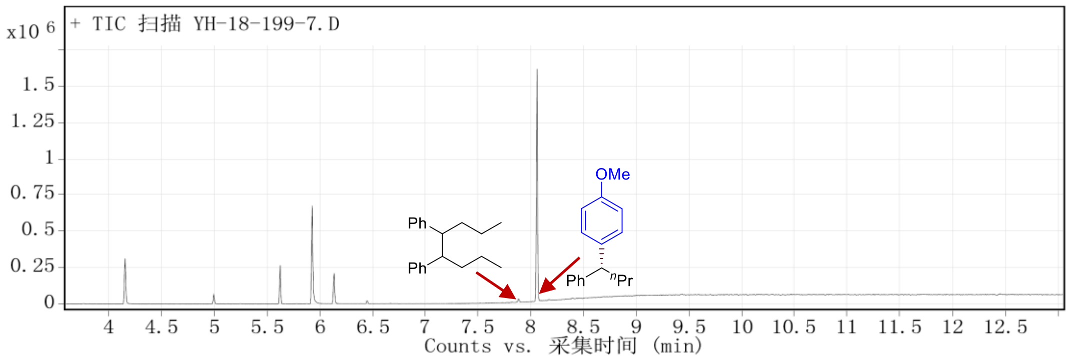

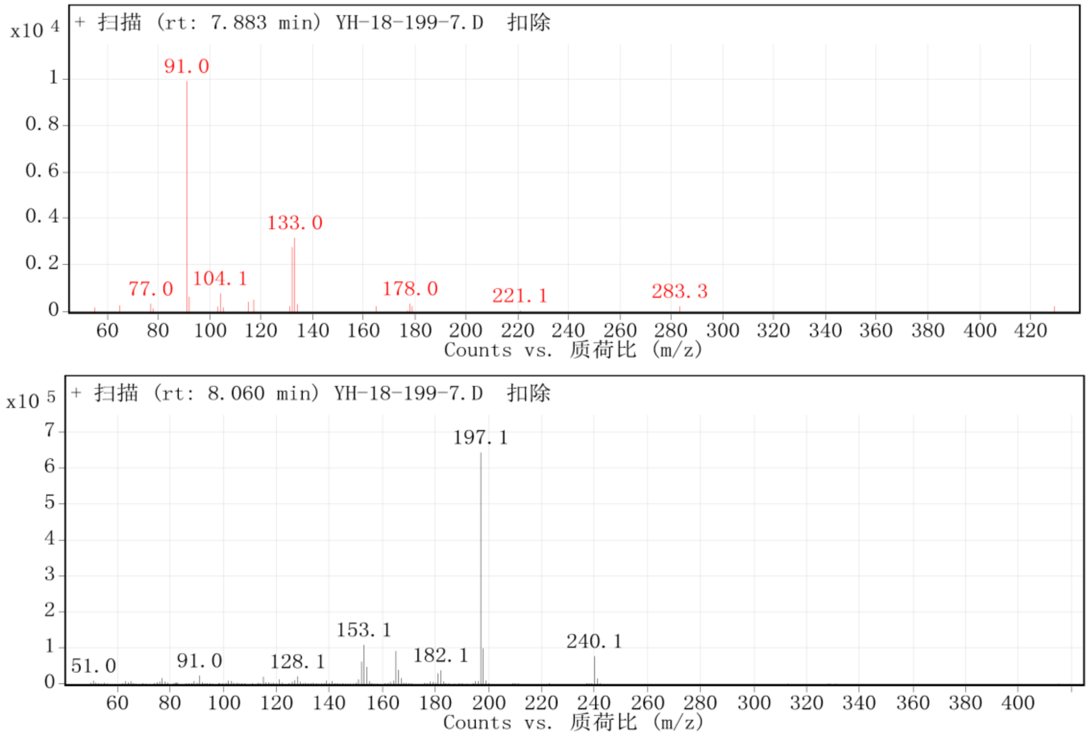

Supplementary Fig. 3. GC-MS Analysis of **3a**

2.5. Detailed Condition Optimization

Supplementary Table 1. Nickel salts screening for enantioselective C(sp^3)−H arylation

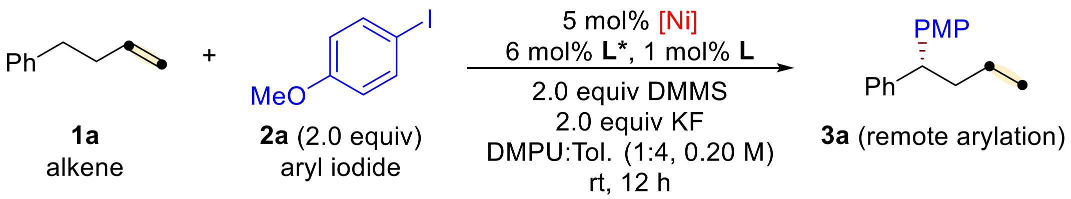

entry	[Ni]	yield of **3a** (%)[a]	rr[b]	ee[c]
1	***$NiCl_2$·glyme***	***90 (84)***	***99:1***	***95***
2	$NiCl_2$	47	97:3	95
3	$NiBr_2$	76	98:2	94
4	NiI_2	80	99:1	94
5	$NiBr_2$·glyme	82	98:2	95
6	$Ni(COD)_2$	85	99:1	94

Supplementary Table 2. Chiral ligand screening for enantioselective C(sp^3)−H arylation

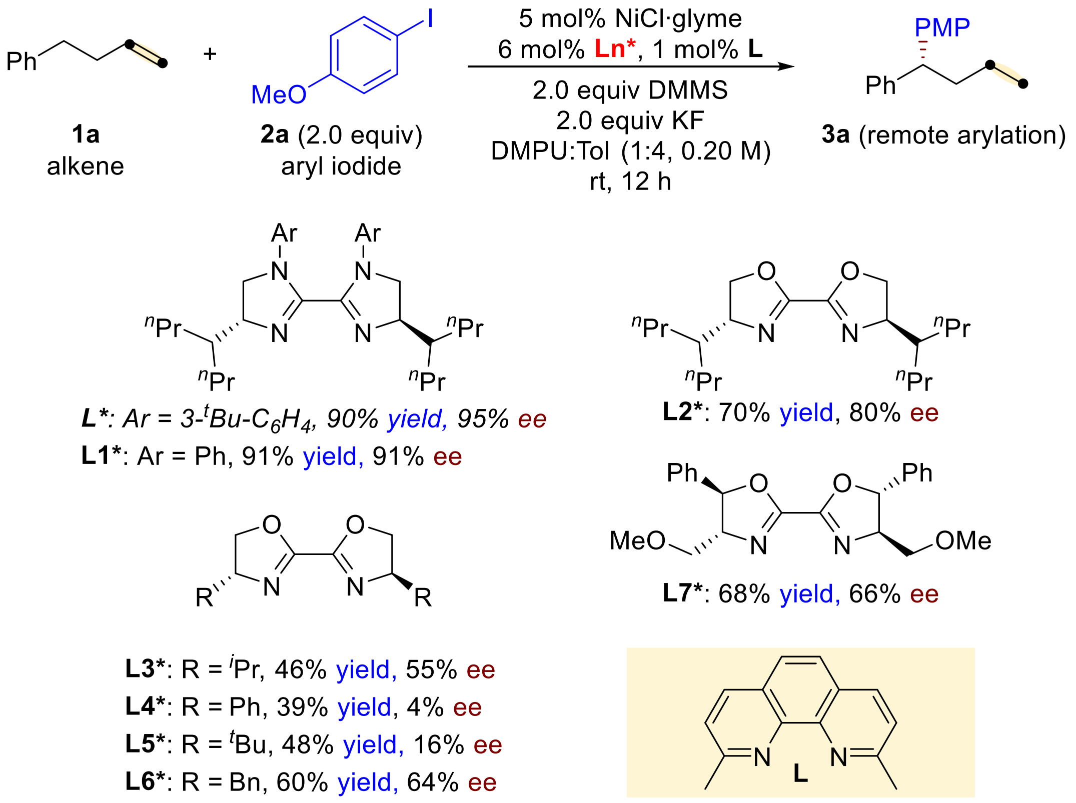

Supplementary Table 3. Base screening for enantioselective C(sp^3)−H arylation

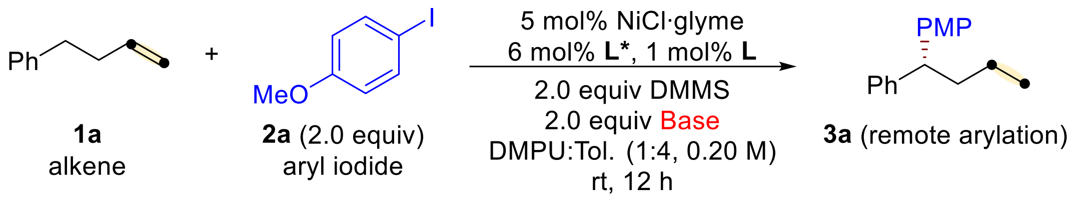

entry	Base	yield of **3a** (%)[a]	rr[b]	ee[c]
1	***KF***	***90 (84)***	***99:1***	***95***
2	CsF	42	95:5	91
3	NaF	trace	-	-
4	K_3PO_4	19	89:11	-
5	$K_3PO_4 \cdot H_2O$	79	99:1	93
6	LiOMe	24	85:15	-

Supplementary Table 4. Solvent screening for enantioselective C(sp^3)−H arylation

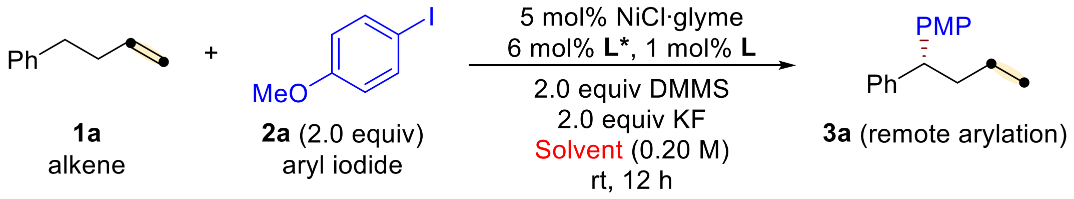

entry	Solvent	yield of **3a** (%)[a]	rr[b]	ee[c]
1	***DMPU:Tol (1:4)***	***90 (84)***	***99:1***	***95***
2	DMPU:Tol (1:1)	80	95:5	95
3	DMPU:Tol (1:9)	61	99:1	96
4	DMPU	44	94:6	93
5	Tol	3	-	-
6	DMAc	53	96:4	82

Supplementary Table 5. Silane screening for enantioselective C(sp^3)−H arylation

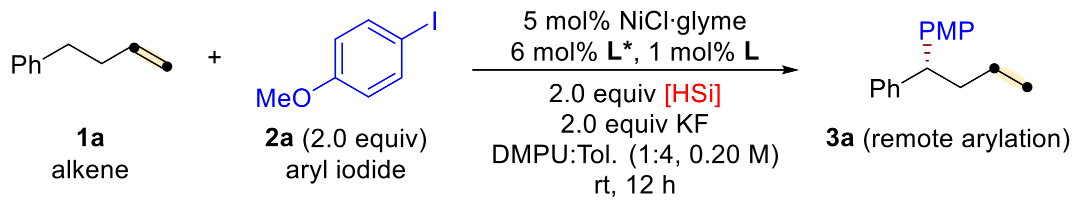

entry	[HSi]	yield of **3a** (%)[a]	rr[b]	ee[c]
1	***(MeO)$_2$MeSiH***	***90 (84)***	***99:1***	***95***
2	(EtO)$_2$MeSiH	93	95:5	95
3	(EtO)$_3$SiH	64	97:3	95
4	(MeO)$_3$SiH	72	97:3	95
5	PMHS	70	98:2	95

Supplementary Table 6. ligand ratio screening for enantioselective C(sp^3)−H arylation

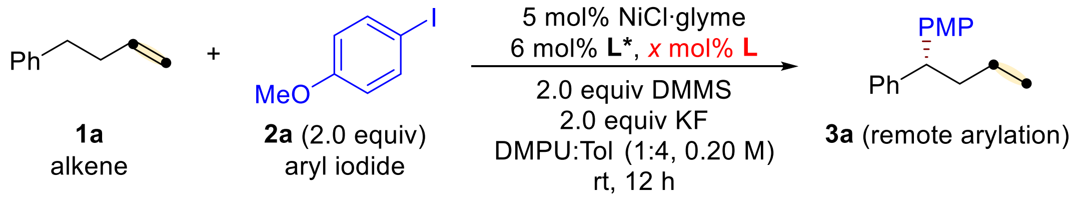

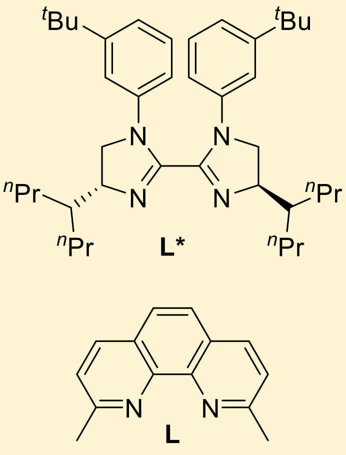

**L** (*x* mol%)	yield (%)[a]	ee (%)[b]	rr[c]
0	52[d]	95	68:32
0.5	84	95	97:3
1	***90***	***95***	***99:1***
2	90	93	99:1
3	88	85	99:1
4	85	68	99:1
5	77	54	99:1
6	60	50	99:1

a.Yields were determined by gas chromatography (GC) analysis using *n*-dodecane as the internal standard, the yield within parentheses is the isolated yield and is an average of two runs (0.20 mmol scale). b. Enantioselectivities were determined by chiral HPLC analysis. c. Regioselectivities (rr) were determined by GC and GC-MS analysis. d.

Without **L**: the other regioisomers were 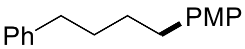 (24% yield) and 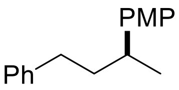 (1% yield), the byproducts were 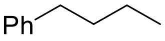 (19% yield) and 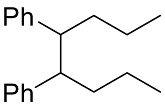 (2% yield).

2.6. Regioconvergent Experiment

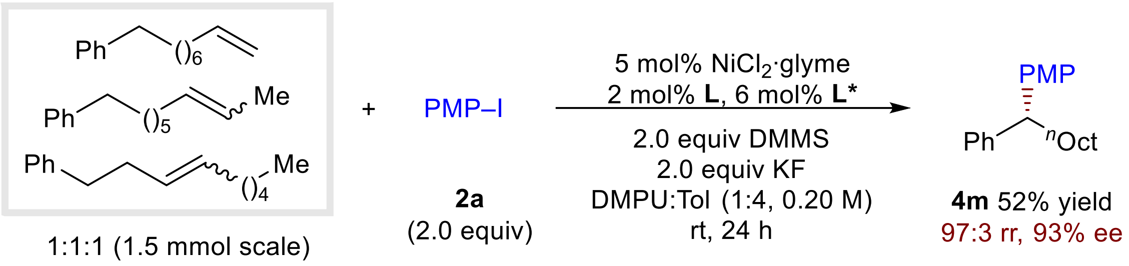

(*R*)-1-methoxy-4-(1-phenylnonyl)benzene (Figure 5, **4m**). From **Non-8-en-1-ylbenzene** (101.2 mg, 0.50 mmol), **Non-7-en-1-ylbenzene** (101.2 mg, 0.50 mmol) and **Non-3-en-1-ylbenzene** (101.2 mg, 0.50 mmol), the title compound was prepared following the general procedure **A** using $NiCl_2$·glyme (16.5 mg, 5.0 mol%), **L*** (57.0 mg, 6.0 mol%), **L** (6.2 mg, 5.0 mol%), KF (174.0 mg, 2.0 equiv), 4-iodoanisole (705.0 mg, 3.0 mmol, 2.0 equiv), DMMS (370.0 μL, 3.0 mmol), anhydrous toluene (6.0 mL) and DMPU (1.5 mL). The reaction mixture was stirred for 24 h at rt. The crude material was purified by flash column chromatography (0–1% EtOAc in PE) to provide the title compound as a colorless liquid in 52% yield (242.7 mg) with 97:3 rr and 93% *ee*.

2.7. Application of Multiligand-Relay Catalysis in 1,3-Arylboration Reaction

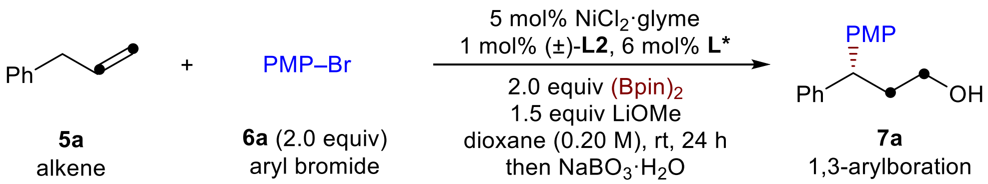

General procedure (B) for the regio- and enantioselective 1,3-arylboration reaction. In a nitrogen-filled glove box, to an oven-dried 8 mL screw-cap vial equipped with a magnetic stir bar was added $NiCl_2$·glyme (2.2 mg, 5.0 mol%), **L*** (7.2 mg, 6.0 mol%), (±)-**L2** (0.50 mg, 1.0 mol%), LiOMe (11.4 mg, 1.5 equiv), and anhydrous dioxane (1.0 mL) were added, and the mixture was stirred for 15 min at room temperature, at which time allylbenzene (26.5 μL, 0.20 mmol), 4-bromoanisole (50.0 μL, 0.40 mmol) and $(Bpin)_2$ (101.6 mg, 0.40 mmol) were added to the resulting mixture in this order. The tube was sealed with a teflon-lined screw cap, removed from the glove box and the reaction was stirred at rt (22~26 °C) for up to 24 h (the mixture was stirred at 750 rpm, ensuring that the base was uniformly suspended). After the reaction was complete, the reaction mixture was directly filtered through a short pad of silica gel [EtOAc in Petroleum ether (PE)] to give the crude product. Dodecane (20 μL) was added as an internal standard for GC analysis. The crude product was then oxidized to the corresponding alcohol with $NaBO_3 \cdot H_2O$ and purified by chromatography on silica gel for each substrate. The yields reported are the average of at least two experiments, unless otherwise indicated. The enantiomeric excesses (% *ee*) were determined by HPLC analysis using chiral stationary phases.

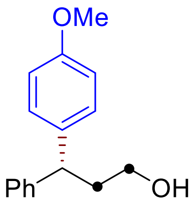

(*R*)-3-(4-Methoxyphenyl)-3-phenylpropan-1-ol (Figure 5, **7a**). From **4-Bromoanisole** (50.0 μL, 0.40 mmol), the title compound was prepared following the general procedure **B** using $NiCl_2$·glyme (2.2 mg, 5.0 mol%), **L*** (7.2 mg, 6.0 mol%), (±)-**L2** (0.50 mg, 1.0 mol%), LiOMe (11.4 mg, 1.5 equiv), allylbenzene (26.5 μL, 0.20 mmol), $(Bpin)_2$ (101.6 mg, 0.40 mmol) and anhydrous dioxane (1.0 mL). The reaction mixture was stirred for 24 h at rt. The crude material was purified by flash column chromatography (0–10% EtOAc in PE) to provide the title compound as a colorless liquid in 61% yield (29.5 mg) with 95:5 rr.

Rf 0.3 (10% EtOAc in PE), UV;

^{1}**H NMR** (500 MHz, $CDCl_3$) δ 7.34 – 7.25 (m, 4H), 7.25 – 7.17 (m, 3H), 6.86 (d, *J* = 8.7 Hz, 2H), 4.12 (t, *J* = 7.9 Hz, 1H), 3.80 (s, 3H), 3.63 (t, *J* = 6.4 Hz, 2H), 2.37 – 2.28 (m, 2H);

^{13}**C NMR** (126 MHz, $CDCl_3$) δ 158.0, 144.9, 136.6, 128.8, 128.6, 127.8, 126.2, 113.9, 61.2, 55.3, 46.5, 38.4;

HRMS (ESI) calcd. for $C_{16}H_{19}O_2$ $[M+H]^+$ *m/z* 243.1380, found 243.1378;

IR (neat, cm^{-1}) 3348(br), 2969, 1583, 1245, 1028, 698;

$[\alpha]_D^{20}$ = –6.8 (*c* = 1.0, $CHCl_3$); 95% *ee*;

HPLC analysis CHIRALCEL AD-H column, 5% ^{i}PrOH in hexane, 0.8 mL/min, 220 nm UV detector, t_R (major) = 38.4 min, t_R (minor) = 42.8 min.

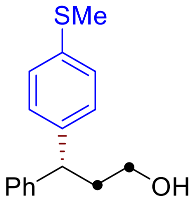

(*R*)-3-(4-(Methylthio)phenyl)-3-phenylpropan-1-ol (Figure 5, **7b**). From **(4-Bromophenyl)(methyl)sulfane** (81.2 mg, 0.40 mmol), the title compound was prepared following the general procedure **B** using $NiCl_2$·glyme (2.2 mg, 5.0 mol%), **L*** (7.2 mg, 6.0 mol%), (±)-**L2** (0.50 mg, 1.0 mol%), LiOMe (11.4 mg, 1.5 equiv), allylbenzene (26.5 μL, 0.20 mmol), $(Bpin)_2$ (101.6 mg, 0.40 mmol) and anhydrous dioxane (1.0 mL). The reaction mixture was stirred for 24 h at rt. The crude material was purified by flash column chromatography (0–10% EtOAc in PE) to provide the title compound as a colorless liquid in 50% yield (25.9 mg) with 96:4 rr.

Rf 0.3 (10% EtOAc in PE), UV;

^{1}**H NMR** (500 MHz, $CDCl_3$) δ 7.33 – 7.29 (m, 2H), 7.28 – 7.25 (m, 2H), 7.24 – 7.18 (m, 5H), 4.13 (t, *J* = 7.9 Hz, 1H), 3.63 (t, *J* = 6.4 Hz, 2H), 2.47 (s, 3H), 2.32 (q, *J* = 7.5 Hz, 2H);

^{13}**C NMR** (126 MHz, $CDCl_3$) δ 144.4, 141.6, 136.0, 128.6, 128.4, 127.8, 127.1, 126.4, 61.0, 46.8, 38.1, 16.1;

HRMS (ESI) calcd. for $C_{16}H_{18}NaOS$ $[M+Na]^+$ *m/z* 281.0971, found 281.0973;

IR (neat, cm^{-1}) 3344(br), 2920, 1491, 1049, 698;

$[\alpha]_D^{20}$ = –5.4 (c = 1.0, $CHCl_3$); 96% *ee*;

HPLC analysis CHIRALCEL AD-H column, 5% ^{i}PrOH in hexane, 1.0 mL/min, 220 nm UV detector, t_R (minor) = 23.8 min, t_R (major) = 26.3 min.

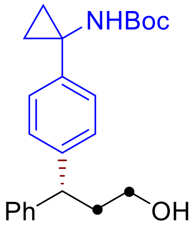

tert*-Butyl (*R*)-(1-(4-(3-hydroxy-1-phenylpropyl)phenyl)cyclopropyl)carbamate** (Figure 5, **7c**). From ***tert*-Butyl (1-(4-bromophenyl)cyclopropyl)carbamate** (124.9 mg, 0.40 mmol), the title compound was prepared following the general procedure **B** using $NiCl_2$·glyme (2.2 mg, 5.0 mol%), **L (7.2 mg, 6.0 mol%), (±)-**L2** (0.50 mg, 1.0 mol%), LiOMe (11.4 mg, 1.5 equiv), allylbenzene (26.5 μL, 0.20 mmol), $(Bpin)_2$ (101.6 mg, 0.40 mmol) and anhydrous dioxane (1.0 mL). The reaction mixture was stirred for 24 h at rt. The crude material was purified by flash column chromatography (0–10% EtOAc in PE) to provide the title compound as a colorless liquid in 41% yield (30.1 mg) with 95:5 rr.

Rf 0.4 (15% EtOAc in PE), UV;

^{1}H NMR (500 MHz, CD_3OD) δ 7.29 – 7.24 (m, 4H), 7.21 (d, J = 7.7 Hz, 2H), 7.18 – 7.08 (m, 3H), 4.10 (t, J = 7.9 Hz, 1H), 3.49 (t, J = 6.6 Hz, 2H), 2.27 (q, J = 7.0 Hz, 2H), 1.45 (s, 9H), 1.21 – 1.12 (m, 4H);

^{13}C NMR (126 MHz, CD_3OD) δ 157.1, 144.9, 142.3, 141.5, 128.0, 127.5, 127.4, 125.7, 124.4, 78.8, 59.7, 46.6, 37.9, 33.9, 27.4, 17.6;

HRMS (ESI) calcd. for $C_{23}H_{29}NNaO_3$ $[M+Na]^+$ m/z 390.2040, found 390.2039;

IR (neat, cm^{-1}) 3348(br), 2973, 1690, 1392, 1066, 699;

$[\alpha]_D^{20}$ = –7.2 (c = 1.0, $CHCl_3$); 94% *ee*;

HPLC analysis CHIRALCEL OD-H column, 25% ^{i}PrOH in hexane, 1.0 mL/min, 220 nm UV detector, t_R (major) = 15.3 min, t_R (minor) = 26.9 min.

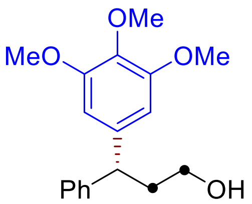

(*R*)-3-Phenyl-3-(3,4,5-trimethoxyphenyl)propan-1-ol (Figure 5, **7d**). From **5-Bromo-1,2,3-trimethoxybenzene** (98.8 mg, 0.40 mmol), the title compound was prepared following the general procedure **B** using $NiCl_2$·glyme (2.2 mg, 5.0 mol%), **L*** (7.2 mg, 6.0 mol%), (±)-**L2** (0.50 mg, 1.0 mol%), LiOMe (11.4 mg, 1.5 equiv), allylbenzene (26.5 μL, 0.20 mmol), $(Bpin)_2$ (101.6 mg, 0.40 mmol) and anhydrous dioxane (1.0 mL). The reaction mixture was stirred for 24 h at rt. The crude material was purified by flash column chromatography (0–20% EtOAc in PE) to provide the title compound as a colorless liquid in 55% yield (33.4 mg) with 92:8 rr.

Rf 0.2 (20% EtOAc in PE), UV;

^{1}H NMR (500 MHz, $CDCl_3$) δ 7.34 – 7.28 (m, 4H), 7.22 (t, *J* = 7.0 Hz, 1H), 6.49 (s, 2H), 4.09 (t, *J* = 7.9 Hz, 1H), 3.84 (s, 6H), 3.83 (s, 3H), 3.64 (t, *J* = 6.4 Hz, 2H), 2.36 – 2.28 (m, 2H);

^{13}C NMR (126 MHz, $CDCl_3$) δ 153.2, 144.3, 140.3, 136.4, 128.6, 127.7, 126.4, 104.9, 61.0, 60.8, 56.1, 47.6, 38.4;

HRMS (ESI) calcd. for $C_{18}H_{22}NaO_4$ $[M+Na]^+$ *m/z* 325.1410, found 325.1409;

IR (neat, cm^{-1}) 3346(br), 2969, 1588, 1122, 702;

$[\alpha]_D^{20}$ = –0.4 (*c* = 1.2, $CHCl_3$); 96% *ee*;

HPLC analysis CHIRALCEL OD-H column, 10% ^{i}PrOH in hexane, 1.0 mL/min, 220 nm UV detector, t_R (minor) = 13.8 min, t_R (major) = 16.9 min.

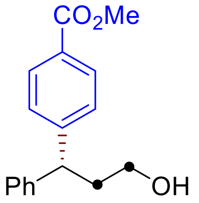

Methyl (*R*)-4-(3-hydroxy-1-phenylpropyl)benzoate (Figure 5, **7e**). From **Methyl 4-bromobenzoate** (86.0 mg, 0.40 mmol), the title compound was prepared following the general procedure **B** using $NiCl_2$·glyme (2.2 mg, 5.0 mol%), **L*** (7.2 mg, 6.0 mol%), (±)-

L2 (0.50 mg, 1.0 mol%), LiOMe (11.4 mg, 1.5 equiv), allylbenzene (26.5 μL, 0.20 mmol), $(Bpin)_2$ (101.6 mg, 0.40 mmol) and anhydrous dioxane (1.0 mL). The reaction mixture was stirred for 24 h at rt. The crude material was purified by flash column chromatography (0–15% EtOAc in PE) to provide the title compound as a colorless liquid in 53% yield (28.8 mg) with 90:10 rr.

Rf 0.3 (20% EtOAc in PE), UV;

^{1}H NMR (500 MHz, $CDCl_3$) δ 7.98 (d, J = 8.4 Hz, 2H), 7.35 (d, J = 8.3 Hz, 2H), 7.33 – 7.29 (m, 2H), 7.26 (d, J = 6.9 Hz, 2H), 7.23 (d, J = 7.2 Hz, 1H), 4.24 (t, J = 7.9 Hz, 1H), 3.91 (s, 3H), 3.62 (t, J = 6.4 Hz, 2H), 2.41 – 2.30 (m, 2H);

^{13}C NMR (126 MHz, $CDCl_3$) δ 167.0, 149.9, 143.6, 129.9, 128.7, 128.2, 127.9, 127.9, 126.6, 60.7, 52.1, 47.2, 37.9;

HRMS (ESI) calcd. for $C_{17}H_{18}NaO_3$ $[M+Na]^+$ *m/z* 293.1148, found 293.1147;

IR (neat, cm^{-1}) 3348(br), 2952, 1717, 1276, 698;

$[\alpha]_D^{20}$ = –4.0 (c = 1.0, $CHCl_3$); 94% *ee*;

HPLC analysis CHIRALCEL OD-H column, 20% ^{i}PrOH in hexane, 1.0 mL/min, 220 nm UV detector, t_R (minor) = 9.0 min, t_R (major) = 12.9 min.

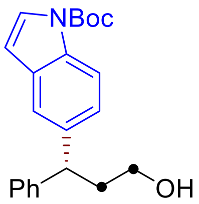

tert*-Butyl (*R*)-5-(3-hydroxy-1-phenylpropyl)-1*H*-indole-1-carboxylate** (Figure 5, **7f**). From ***tert*-Butyl 5-bromo-1*H*-indole-1-carboxylate** (118.5 mg, 0.40 mmol), the title compound was prepared following the general procedure **B** using $NiCl_2$·glyme (2.2 mg, 5.0 mol%), **L (7.2 mg, 6.0 mol%), (±)-**L2** (0.50 mg, 1.0 mol%), LiOMe (11.4 mg, 1.5 equiv), allylbenzene (26.5 μL, 0.20 mmol), $(Bpin)_2$ (101.6 mg, 0.40 mmol) and anhydrous dioxane (1.0 mL). The reaction mixture was stirred for 24 h at rt. The crude material was purified by flash column chromatography (0–10% EtOAc in PE) to provide the title compound as a colorless liquid in 66% yield (46.3 mg) with 95:5 rr.

Rf 0.3 (10% EtOAc in PE), UV;

^{1}H NMR (500 MHz, CD_3OD) δ 8.01 (d, *J* = 8.6 Hz, 1H), 7.56 (d, *J* = 3.7 Hz, 1H), 7.48 (s, 1H), 7.30 (d, *J* = 6.7 Hz, 2H), 7.27 (d, *J* = 7.4 Hz, 2H), 7.21 (dd, *J* = 8.6, 1.8 Hz, 1H), 7.14 (t, *J* = 7.2 Hz, 1H), 6.55 (d, *J* = 3.7 Hz, 1H), 4.22 (t, *J* = 7.9 Hz, 1H), 3.51 (t, *J* = 6.6 Hz, 2H), 2.33 (q, *J* = 7.3, 6.7 Hz, 2H), 1.65 (s, 9H);

^{13}C NMR (126 MHz, CD_3OD) δ 149.7, 145.3, 139.4, 133.7, 130.8, 128.0, 127.6, 125.7, 125.6, 124.2, 119.5, 114.5, 107.1, 83.4, 59.8, 46.9, 38.3, 26.9;

HRMS (ESI) calcd. for $C_{22}H_{25}NNaO_3$ $[M+Na]^+$ *m/z* 374.1727, found 374.1729;

IR (neat, cm^{-1}) 3348(br), 2974, 1729, 1467, 1371, 1160, 699;

$[\alpha]_D^{20}$ = –9.2 (*c* = 1.0, $CHCl_3$); 95% *ee*;

HPLC analysis CHIRALCEL AD-H column, 30% ^{i}PrOH in hexane, 0.8 mL/min, 220 nm UV detector, t_R (major) = 6.3 min, t_R (minor) = 16.6 min.

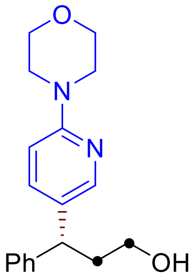

(*R*)-3-(6-Morpholinopyridin-3-yl)-3-phenylpropan-1-ol (Figure 5, **7g**). From **4-(5-Bromopyridin-2-yl)morpholine** (97.2 mg, 0.40 mmol), the title compound was prepared following the general procedure **B** using $NiCl_2$·glyme (2.2 mg, 5.0 mol%), **L*** (7.2 mg, 6.0 mol%), (±)-**L2** (0.50 mg, 1.0 mol%), LiOMe (11.4 mg, 1.5 equiv), allylbenzene (26.5 µL, 0.20 mmol), $(Bpin)_2$ (101.6 mg, 0.40 mmol) and anhydrous dioxane (1.0 mL). The reaction mixture was stirred for 24 h at rt. The crude material was purified by flash column chromatography (0–30% EtOAc in PE) to provide the title compound as a colorless liquid in 47% yield (28.2 mg) with 91:9 rr.

Rf 0.2 (30% EtOAc in PE), UV;

^{1}H NMR (500 MHz, $CDCl_3$) δ 8.16 (d, *J* = 2.5 Hz, 1H), 7.40 (dd, *J* = 8.7, 2.5 Hz, 1H), 7.29 (d, *J* = 7.8 Hz, 2H), 7.25 (d, *J* = 7.1 Hz, 2H), 7.20 (t, *J* = 7.2 Hz, 1H), 6.61 (d, *J* = 8.8 Hz, 1H), 4.08 (t, *J* = 7.9 Hz, 1H), 3.82 (t, *J* = 4.9 Hz, 4H), 3.62 (t, *J* = 6.4 Hz, 2H), 3.48 (t, 4H), 2.34 – 2.24 (m, 2H);

^{13}C NMR (126 MHz, $CDCl_3$) δ 158.1, 146.5, 144.1, 137.6, 129.8, 128.6, 127.7, 126.4, 107.2, 66.7, 60.7, 45.8, 43.9;

HRMS (ESI) calcd. for $C_{18}H_{22}N_2NaO_2$ $[M+Na]^+$ *m/z* 321.1573, found 321.1568;

IR (neat, cm^{-1}) 3348(br), 2968, 1603, 1490, 1243, 699;

$[\alpha]_D^{20}$ = –1.6 (*c* = 1.0, $CHCl_3$); 93% *ee*;

HPLC analysis CHIRALCEL AD-H column, 20% ^{i}PrOH in hexane, 1.0 mL/min, 220 nm UV detector, t_R (major) = 12.2 min, t_R (minor) = 14.6 min.

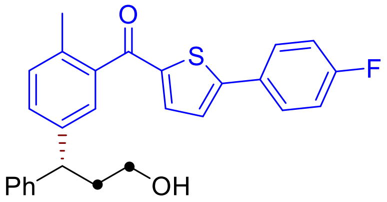

(*R*)-(5-(4-Fluorophenyl)thiophen-2-yl)(5-(3-hydroxy-1-phenylpropyl)-2-methylphenyl)methanone (Figure 5, **7h**). From **(5-Bromo-2-methylphenyl)(5-(4-fluorophenyl)thiophen-2-yl)methanone** (150.1 mg, 0.40 mmol), the title compound was prepared following the general procedure **B** using $NiCl_2$·glyme (2.2 mg, 5.0 mol%), **L*** (7.2 mg, 6.0 mol%), (±)-**L2** (0.50 mg, 1.0 mol%), LiOMe (11.4 mg, 1.5 equiv), allylbenzene (26.5 μL, 0.20 mmol), $(Bpin)_2$ (101.6 mg, 0.40 mmol) and anhydrous dioxane (1.0 mL). The reaction mixture was stirred for 24 h at rt. The crude material was purified by flash column chromatography (0–20% EtOAc in PE) to provide the title compound as a colorless liquid in 44% yield (38.1 mg) with 92:8 rr.

Rf 0.4 (20% EtOAc in PE), UV;

^{1}H NMR (500 MHz, $CDCl_3$) δ 7.65 (dd, *J* = 8.8, 5.2 Hz, 2H), 7.39 (d, *J* = 1.9 Hz, 1H), 7.31 (t, *J* = 7.3 Hz, 3H), 7.27 (d, *J* = 6.3 Hz, 3H), 7.25 – 7.20 (m, 3H), 7.14 (t, *J* = 8.6 Hz, 2H), 4.20 (t, *J* = 7.9 Hz, 1H), 3.64 (t, *J* = 6.4 Hz, 2H), 2.37 (s, 3H), 2.33 (q, *J* = 6.4 Hz, 2H);

^{13}C NMR (126 MHz, $CDCl_3$) δ 190.2, 163.3 (d, *J* = 250.2 Hz), 152.7, 144.2, 143.5, 141.7, 138.2, 136.5, 134.5, 131.4, 129.9, 129.6 (d, *J* = 3.5 Hz), 128.6, 128.2 (d, *J* = 8.5 Hz), 127.8, 127.7, 126.5, 124.0, 116.3 (d, *J* = 22.0 Hz), 60.9, 46.7, 37.9, 19.3;

^{19}F NMR (471 MHz, $CDCl_3$) δ –111.6;

HRMS (ESI) calcd. for $C_{27}H_{23}FNaO_2S$ $[M+Na]^+$ *m/z* 453.1295, found 453.1292;

IR (neat, cm^{-1}) 3348(br), 2971, 1630, 1437, 1055, 698;

$[\alpha]_D^{20}$ = +24.4 (*c* = 1.5, $CHCl_3$); 94% *ee*;

HPLC analysis CHIRALCEL AD-H column, 20% ^{i}PrOH in hexane, 1.0 mL/min, 220 nm UV detector, t_R (major) = 18.7 min, t_R (minor) = 22.9 min.

2.8. Tracing Experiment

Supplementary Table 7. Tracing experiment for the enantioselective C(sp^3)−H arylation

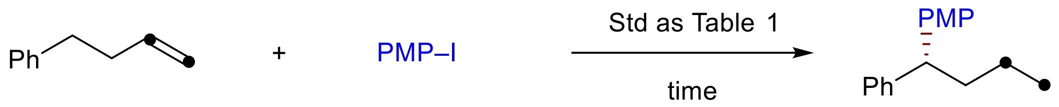

1a **2a** (2.0 equiv) **3a**

Entry	Reaction time	GC yield **3a** [%][a]	**1a**:**1a'**:**1a''**[b]
1	1 h	6	71:19:10
2	2 h	10	35:42:23
3	4 h	46	0:55:45
4	6 h	78	0:36: 64
5	12 h	92	trace

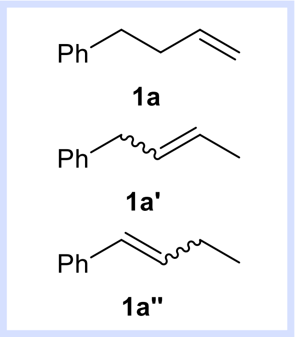

[a]Yields were determined by GC using dodecane as the internal standard. [b]The ratio of **1a**, **1a**' and **1a**'' was determined by GC and GC-MS analysis.

2.9. Olefin Isomerization in the Absence of Aryl Iodide

Supplementary Table 8. Olefin isomerization in the absence of aryl iodide for the enantioselective C(sp^3)−H arylation

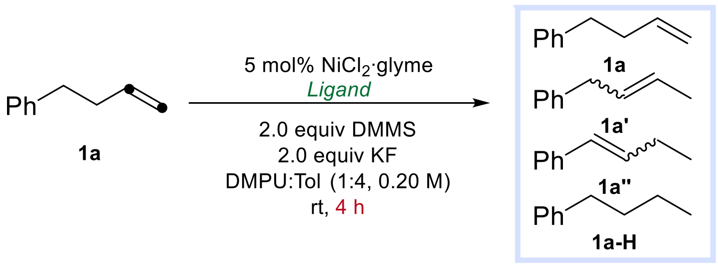

isomer[a]	1 mol% L	6 mol% L*	1 mol% L and 6 mol% L*
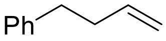	12.4%	83.6%	59.6%
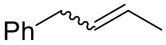	33.6%	3.1%	9.3%
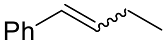	23.4%	6.6%	14.3%
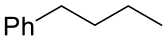	30.6%	6.7%	16.7%

[a]The ratio of **1a**, **1a'**, **1a''** and **1a-H** was determined by GC and GC-MS analysis.

2.10. NiD Experiment

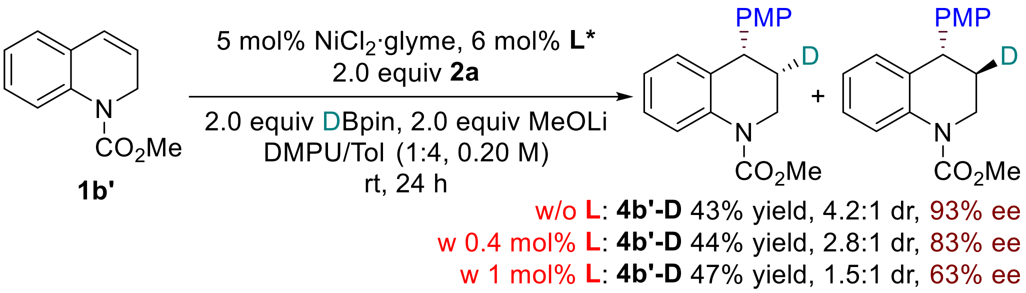

Methyl (3*S*,4*S*)-4-(4-methoxyphenyl)-3,4-dihydroquinoline-1(2*H*)-carboxylate-3-*d* (Figure 6, **4b'-D**). From **Methyl quinoline-1(2*H*)-carboxylate** (18.9 mg, 0.1 mmol), the title compound was prepared following the general procedure **A** using $NiCl_2$·glyme (1.1 mg, 5.0 mol%), **L*** (3.6 mg, 6.0 mol%), LiOMe (7.6 mg, 2.0 equiv), 4-iodoanisole (**2a**) (47.0 mg, 0.20 mmol, 2.0 equiv), DBpin (29 μL, 0.20 mmol), anhydrous toluene (0.40 mL) and DMPU (0.10 mL). The reaction mixture was stirred for 24 h at rt. The crude material was purified by flash column chromatography (0–5% EtOAc in PE) to provide the title compound as a colorless liquid in ***43% yield (13.4 mg) (w/o L)***, ***44% yield (13.6 mg) (w 0.4 mol% L)***, ***47% yield (14.5 mg) (w 1 mol% L)***.

w/o L:

^{1}H NMR (500 MHz, $CDCl_3$) δ 7.78 (d, *J* = 8.4 Hz, 1H), 7.25 – 7.19 (m, 1H), 7.06 (d, *J* = 8.7 Hz, 2H), 7.01 – 6.96 (m, 1H), 6.87 (d, *J* = 8.6 Hz, 3H), 4.09 (d, *J* = 6.0 Hz, 1H), 3.91 – 3.79 (m, 7H), 3.78 – 3.73 (m, 1H), [2.30 – 2.23 (m, 0.84H) & 2.08 – 2.01 (m, 0.20H), *due to dr*];

$[\alpha]_D^{20}$ = +33.7 (*c* = 2.0, $CHCl_3$); 93% *ee*;

HPLC analysis CHIRALCEL OJ-H column, 20% ^{i}PrOH in hexane, 1.0 mL/min, 220 nm UV detector, t_R (minor) = 13.9 min, t_R (major) = 21.5 min.

w 0.4 mol% L:

^{1}H NMR (500 MHz, $CDCl_3$) δ 7.78 (d, *J* = 8.4 Hz, 1H), 7.25 – 7.19 (m, 1H), 7.06 (d, *J* = 8.7 Hz, 2H), 7.01 – 6.96 (m, 1H), 6.87 (d, *J* = 8.6 Hz, 3H), 4.09 (d, *J* = 6.0 Hz, 1H), 3.91 – 3.79 (m, 7H), 3.78 – 3.73 (m, 1H), [2.30 – 2.23 (m, 0.72H) & 2.08 – 2.01 (m, 0.26H), *due to dr*];

$[\alpha]_D^{20}$ = +21.1 (*c* = 2.0, $CHCl_3$); 83% *ee*;

HPLC analysis CHIRALCEL OJ-H column, 20% ^{i}PrOH in hexane, 1.0 mL/min, 220 nm UV detector, t_R (minor) = 13.8 min, t_R (major) = 21.3 min.

w 1 mol% L:

^{1}H NMR (500 MHz, $CDCl_3$) δ 7.78 (d, *J* = 8.4 Hz, 1H), 7.25 – 7.19 (m, 1H), 7.06 (d, *J* = 8.7 Hz, 2H), 7.01 – 6.96 (m, 1H), 6.87 (d, *J* = 8.6 Hz, 3H), 4.09 (d, *J* = 6.0 Hz, 1H), 3.91 – 3.79 (m, 7H), 3.78 – 3.73 (m, 1H), [2.30 – 2.23 (m, 0.61H) & 2.08 – 2.01 (m, 0.41H), *due to dr*];

$[\alpha]_D^{20}$ = +9.9 (*c* = 2.0, $CHCl_3$); 63% *ee*;

HPLC analysis CHIRALCEL OJ-H column, 20% ^{i}PrOH in hexane, 1.0 mL/min, 220 nm UV detector, t_R (minor) = 13.9 min, t_R (major) = 21.5 min.

2.11. Computational Study

Computational details

All calculations were performed with Gaussian 09 program[3]. Geometry optimizations were performed using (U)B3LYP functionals[4,5], and LANL2DZ pseudo-potential[6,7] was set for Ni and I atoms and 6-31G(d) basis set for all the other atoms. After optimization, frequency calculations were subsequently performed to confirm that all stationary points had correct number of imaginary frequencies (zero for minima and one for transition

states) and to provide thermodynamic corrections at 298 K, 1 atm. For all transition states, Intrinsic Reaction Coordinate (IRC)[8] were calculated to confirm that they indeed connected between correct minima. Single-point energies of optimized structures were calculated using (U)M06 functionals[9] with SDD[10,11] for Ni and I atoms and 6-311+G(d,p) for all the other atoms. SMD solvation model[12] was used in single-point energy calculations to consider solvent effect, while dichloromethane (ε = 8.93) was set as implicit solvent to fit the polarity of the mixed solvent in experiments (DMPU [ε = 36.12][13]: toluene [ε = 2.37] = 1:4). The final Gibbs free energy was calculated as the sum of Gibbs free energy correction (from frequency calculation) and the single point energy in solution. For each species, extensive conformational search was done and the reported value is from the conformer with the lowest Gibbs free energy. See supplementary data 1 for detailed energies and atom coordinates for all computed species.

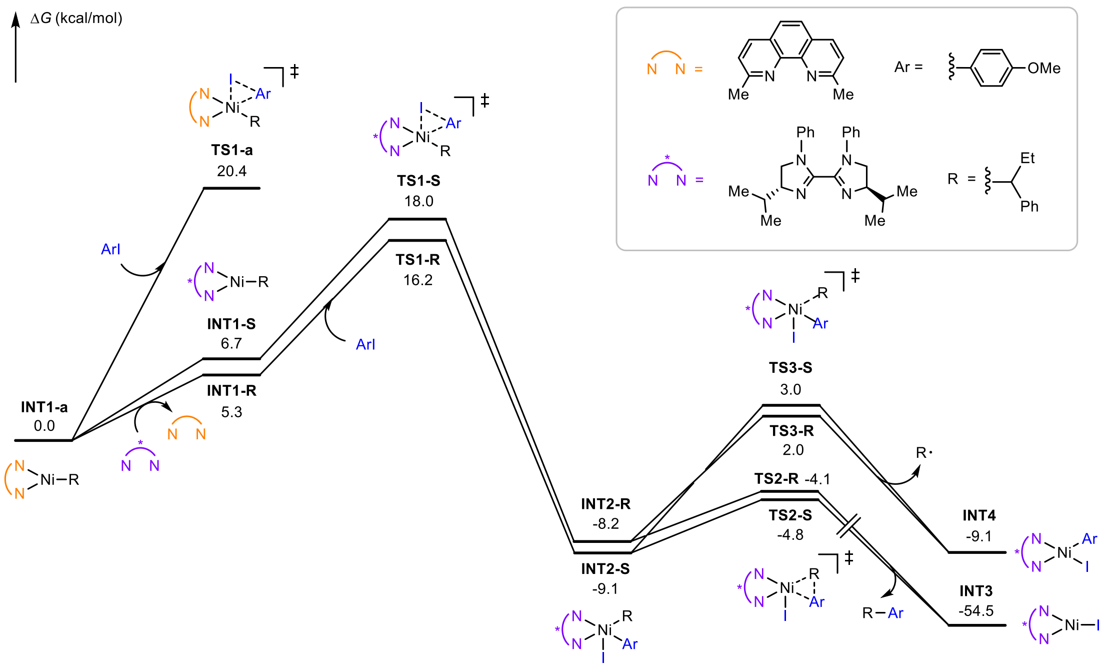

Supplementary Fig 4. Potential energy surface of multi-ligand enabled asymmetric arylation. Computed at SMD(DCM)-(U)M06/6-311+G(d,p)[SDD for Ni and I]//(U)B3LYP/6-31G(d)[LANL2DZ for Ni and I]. Values are relative Gibbs free energies in kcal/mol.

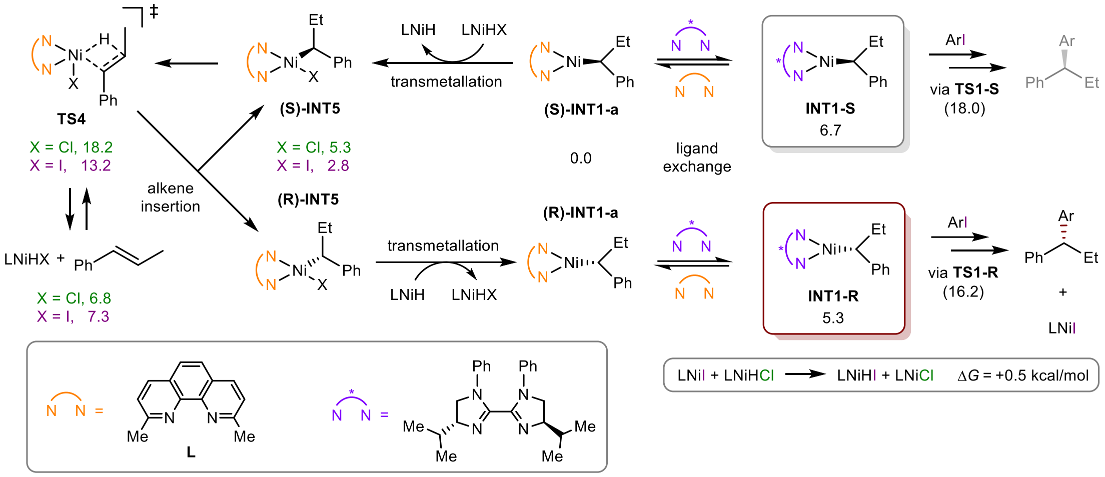

Supplementary Fig 5. Conversion of **INT1-S** to **INT1-R** via a reversible ligand exchange, transmetallation and alkene insertion process. Computed at SMD(DCM)-(U)M06/6-311+G(d,p)[SDD for Ni and I]//(U)B3LYP/6-31G(d)[LANL2DZ for Ni and I]. Values are relative Gibbs free energies in kcal/mol. When X = Cl (from $NiCl_2$), the interconversion needs an activation free energy of 18.2 kcal/mol, which is very close to that of the undesired oxidative addition via **TS1-S** (18.0 kcal/mol). However, as the reaction proceeds, LNiI generates, which can undergo the exchange reaction with LNiHCl to give LNiHI. This process is approximately neutral in energy (+0.5 kcal/mol). When X = I, the barrier for the interconversion is lowered to 13.2 kcal/mol, ensuring a fast conversion of **INT1-S** to **INT1-R**.

2.12. Spectroscopic Data (NMR Spectrum)

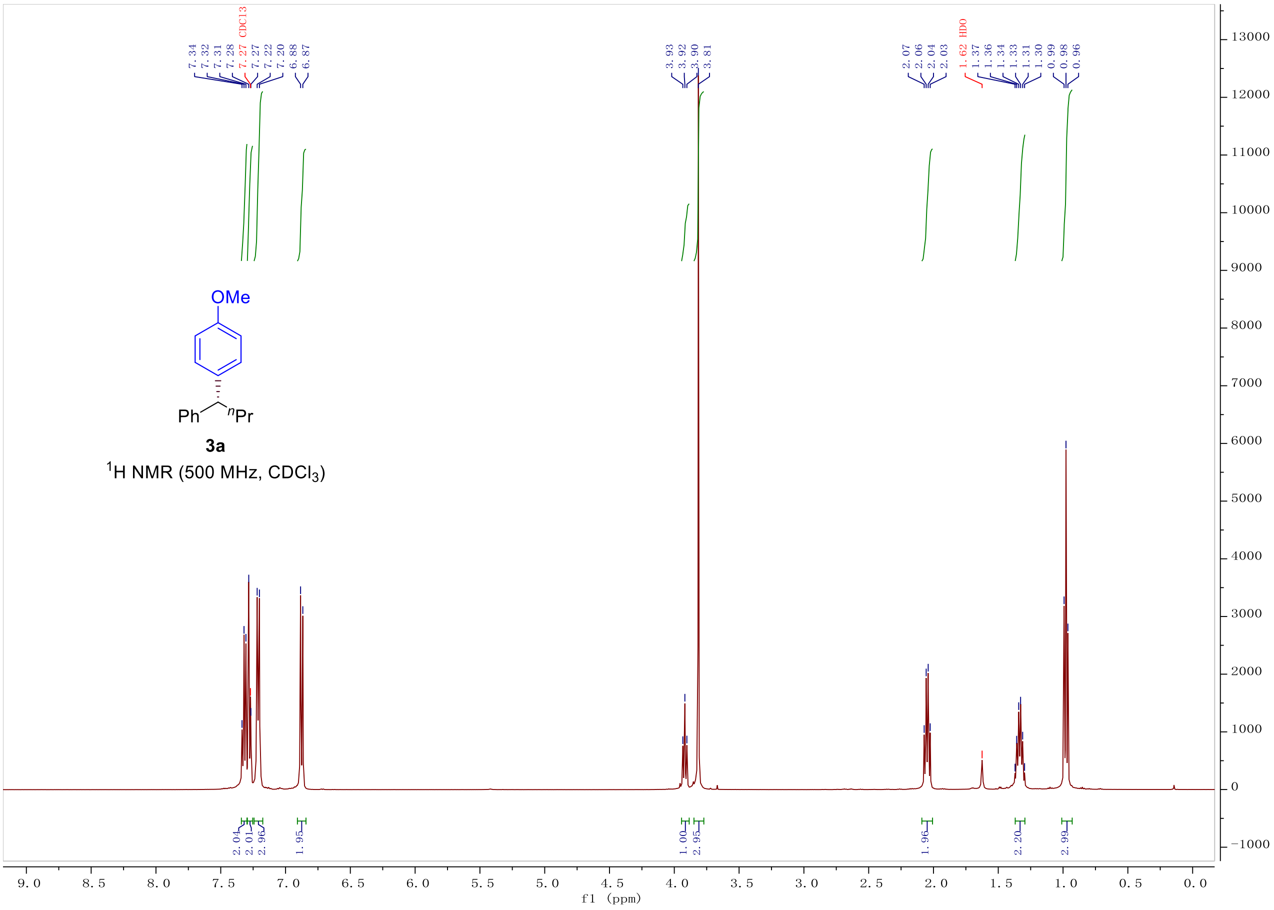

Supplementary Fig. 6. ^{1}H NMR of compound **3a**

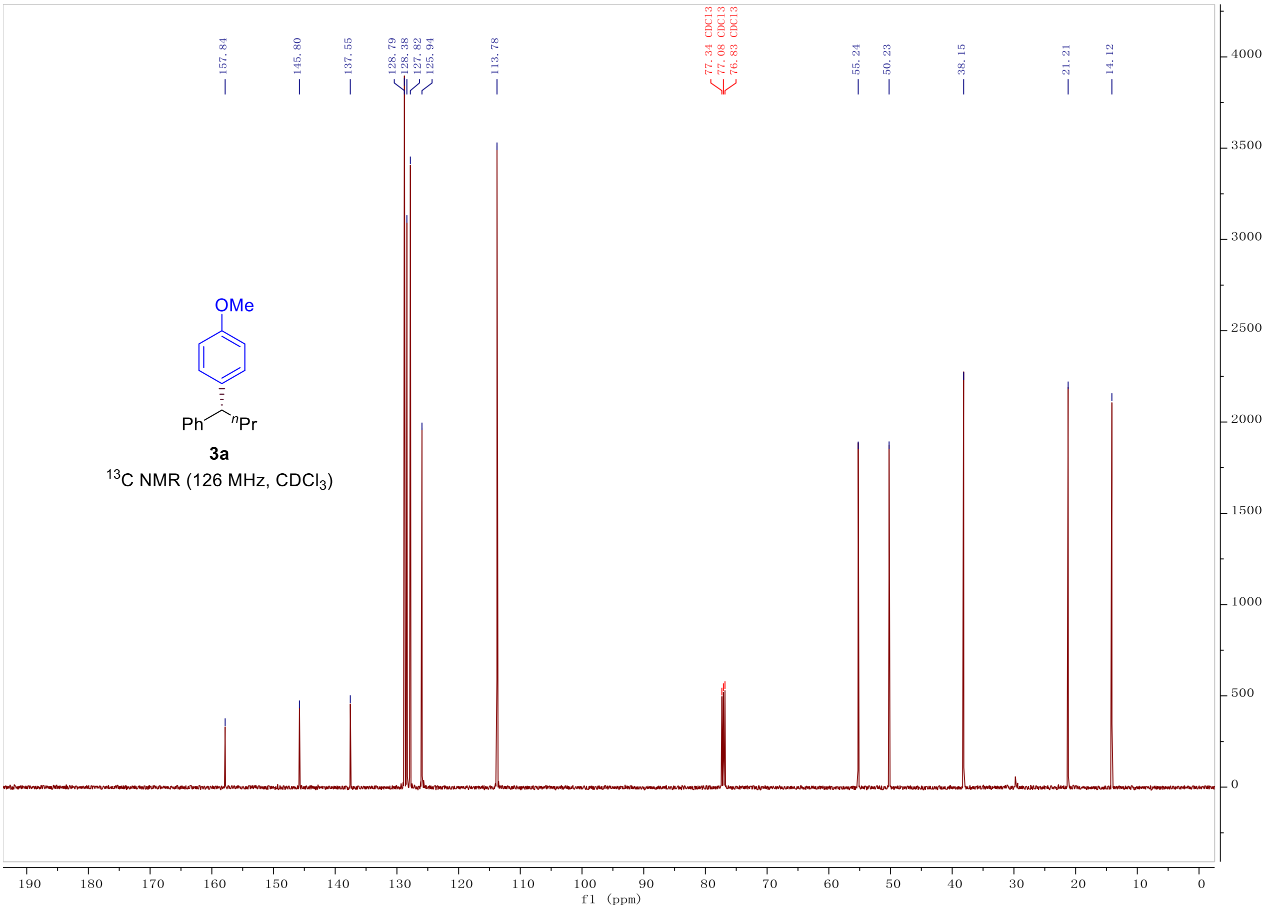

Supplementary Fig. 7. ^{13}C NMR of compound **3a**

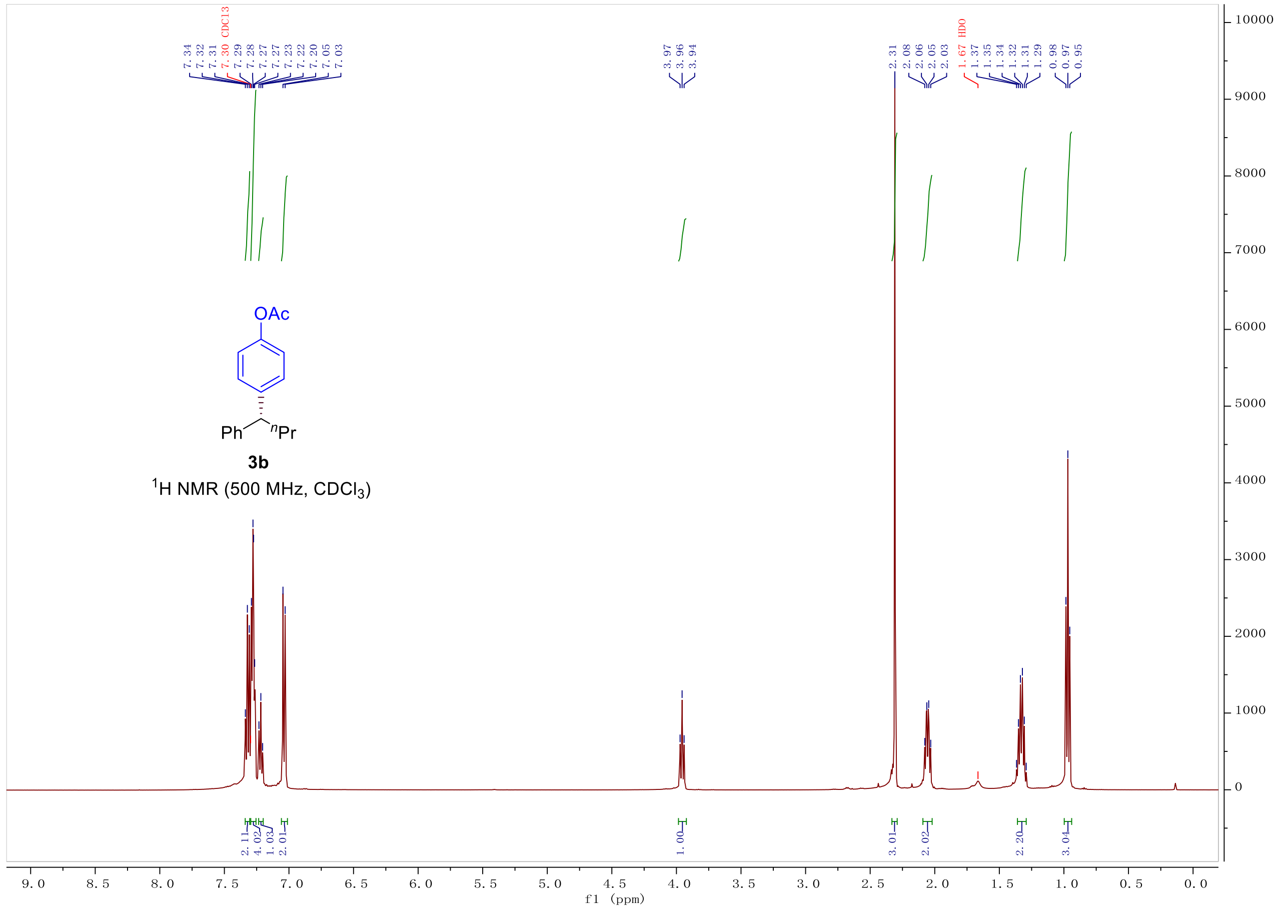

Supplementary Fig. 8. ^{1}H NMR of compound **3b**

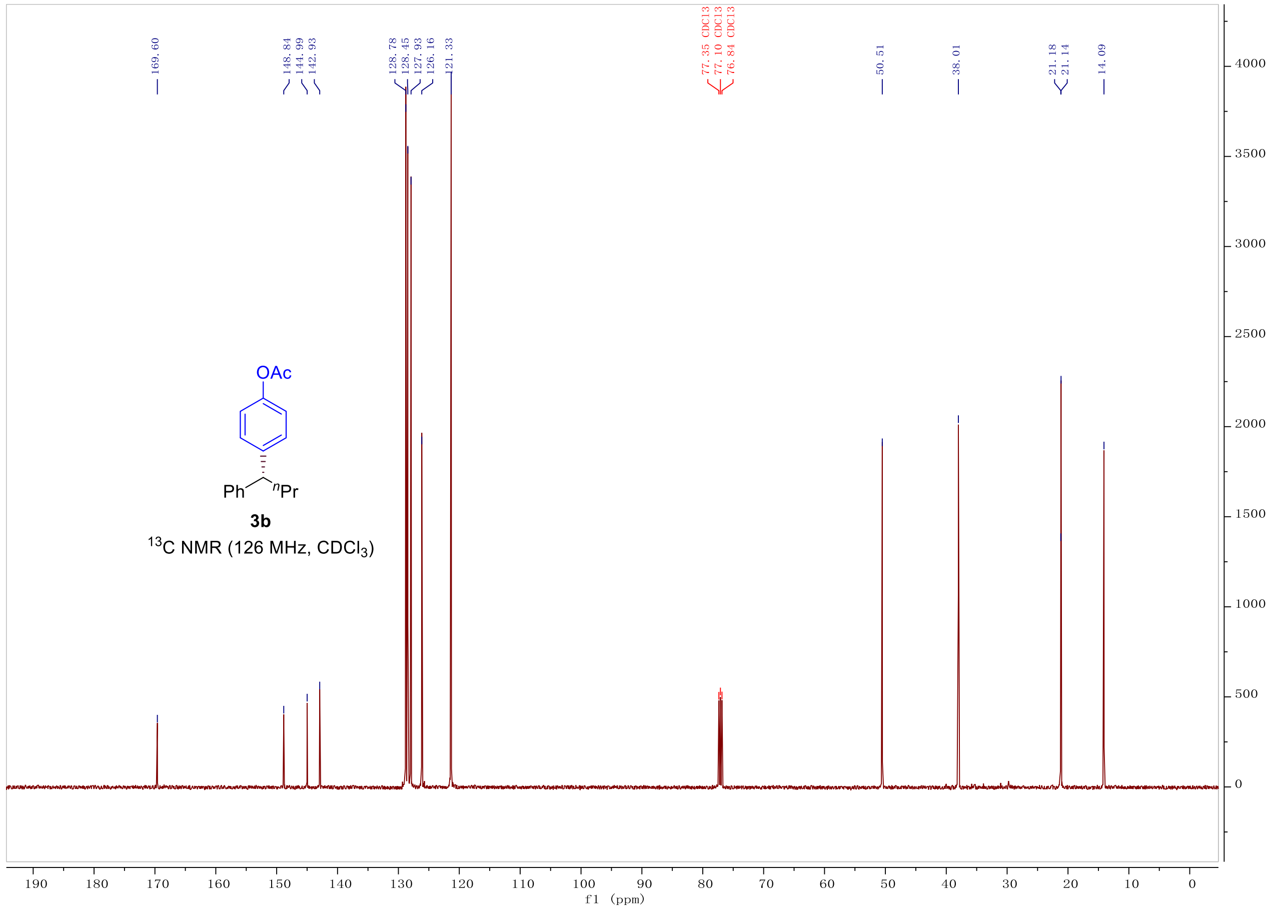

Supplementary Fig. 9. ^{13}C NMR of compound **3b**

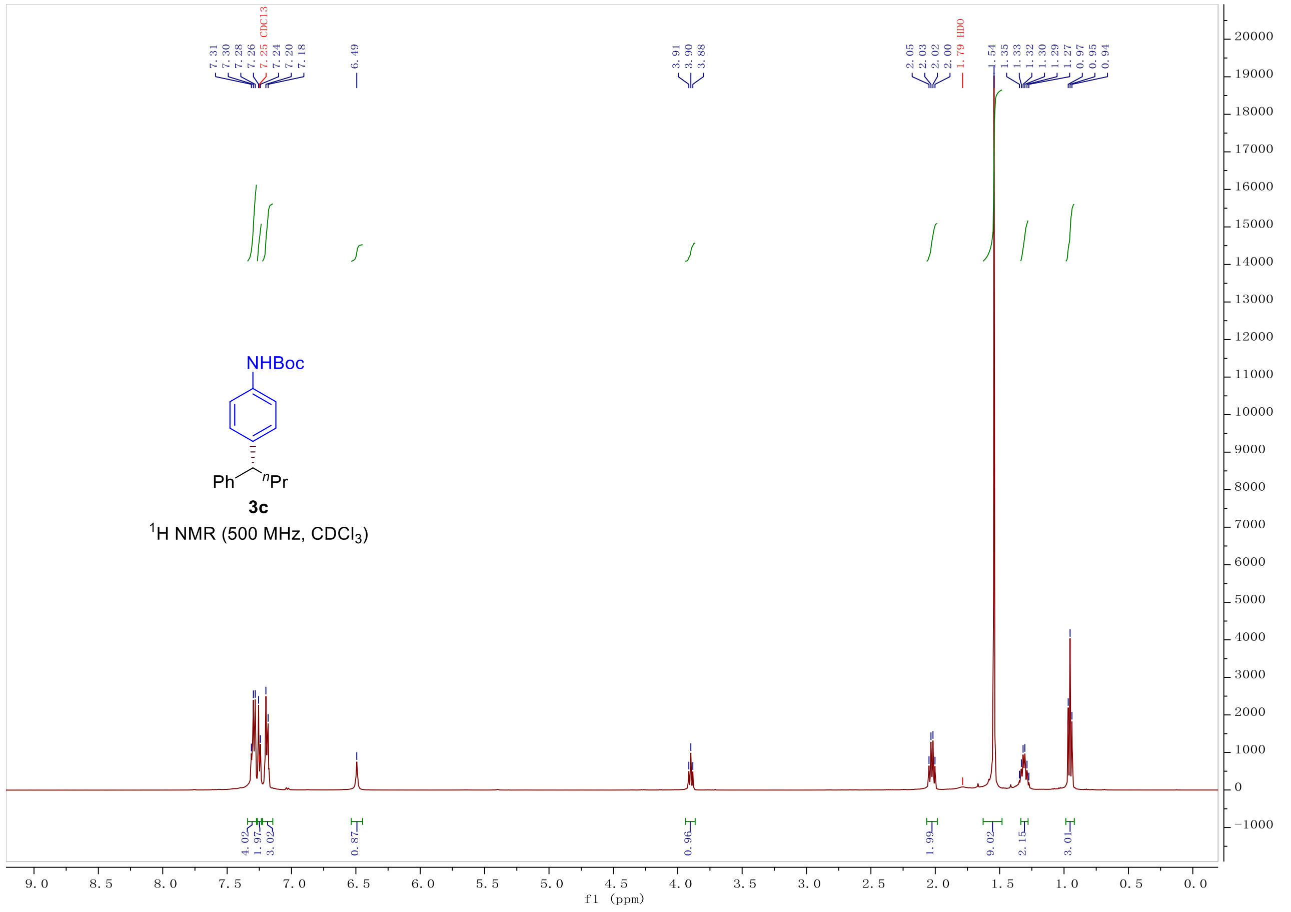

Supplementary Fig. 10. ^{1}H NMR of compound **3c**

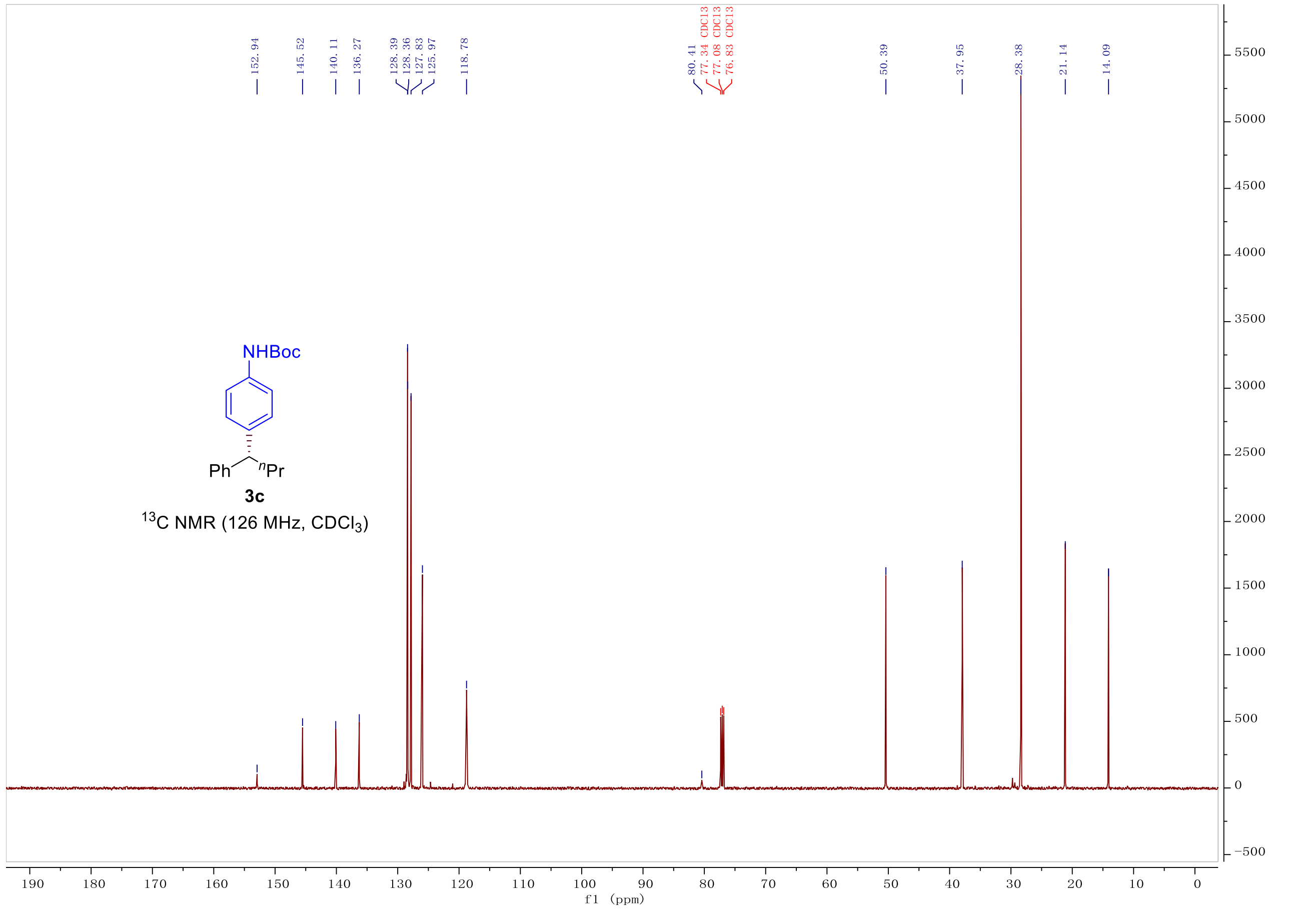

Supplementary Fig. 11. ^{13}C NMR of compound **3c**

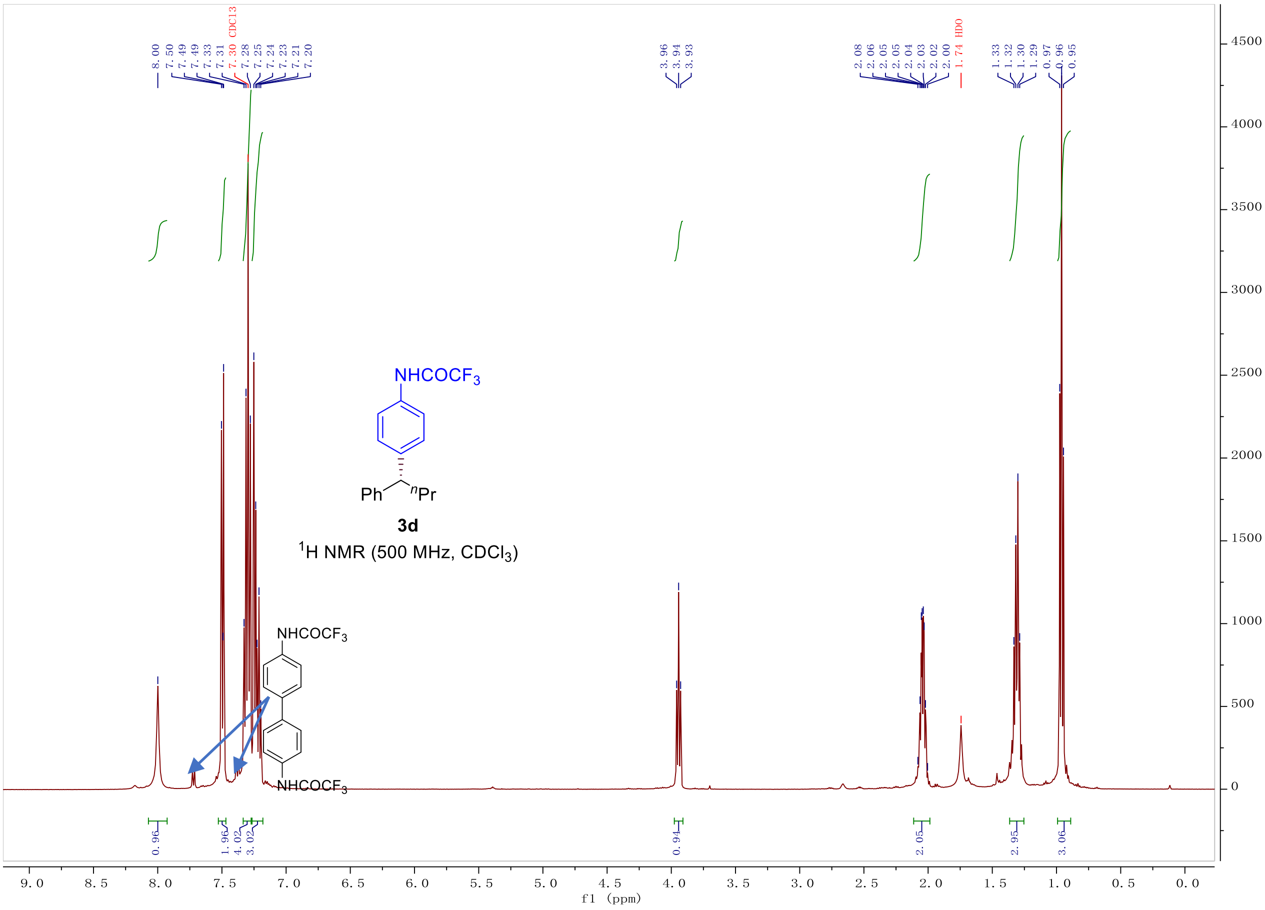

Supplementary Fig. 12. ^{1}H NMR of compound **3d**

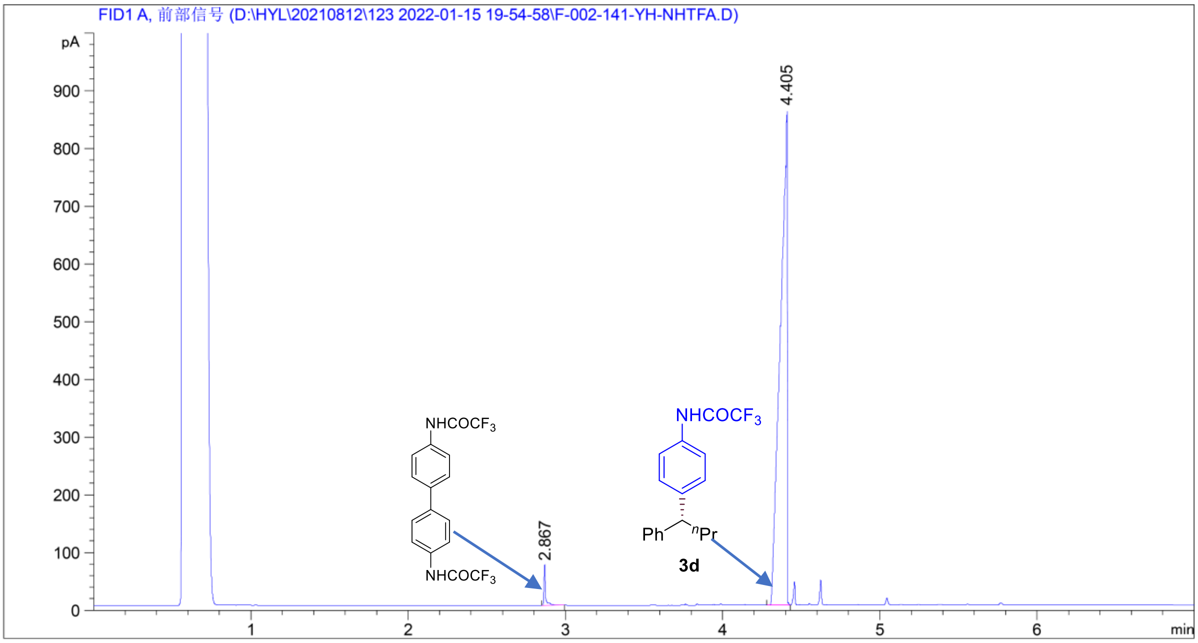

Peak #	RetTime [min]	Type	Width [min]	Area [pA*s]	Height [pA]	Area %
1	2.867	BB	0.0115	49.50231	69.88297	1.85979
2	4.405	BV	0.0379	2612.21851	857.58795	98.14021

Supplementary Fig. 13. GC trace of compound **3d**

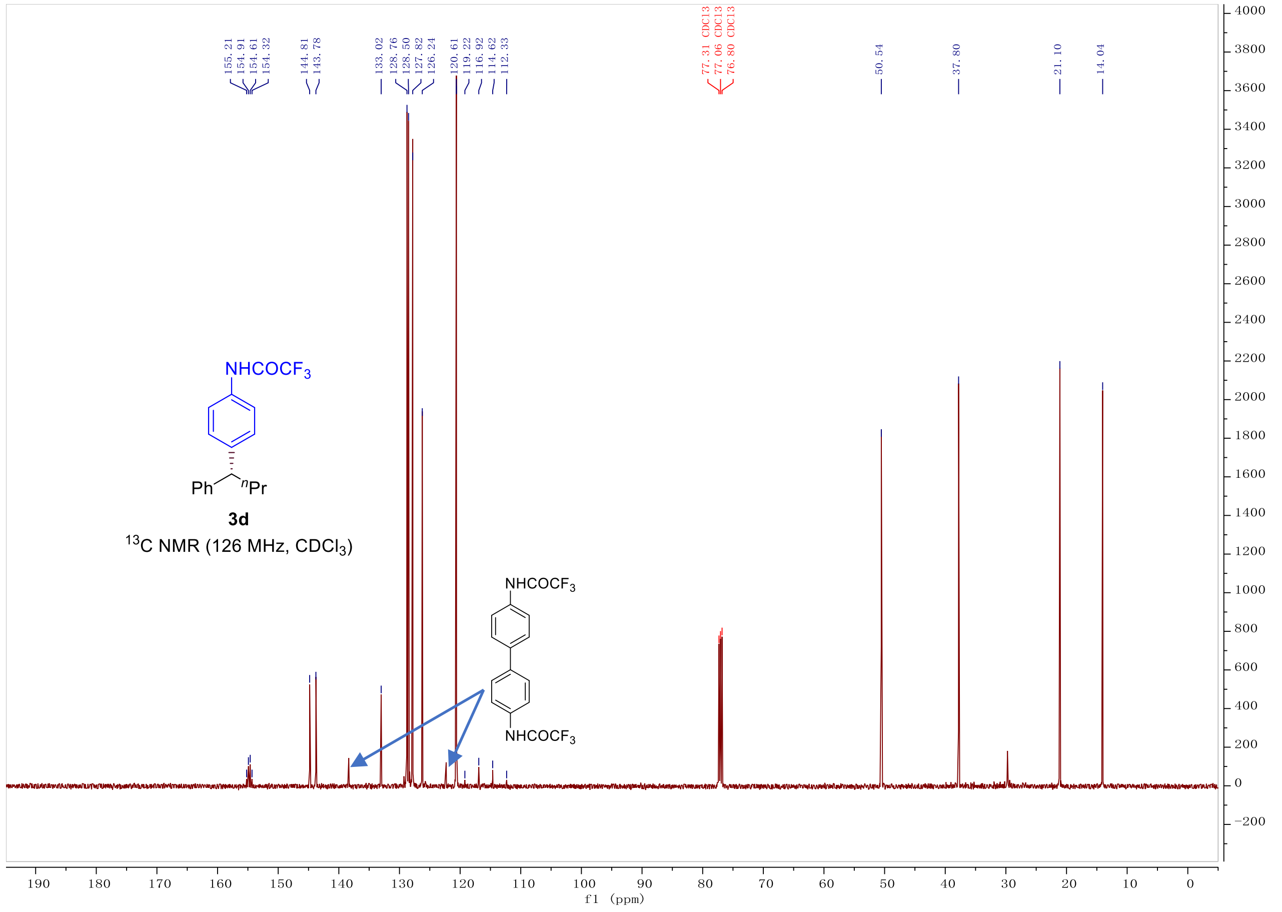

Supplementary Fig. 14. ^{3}C NMR of compound **3d**

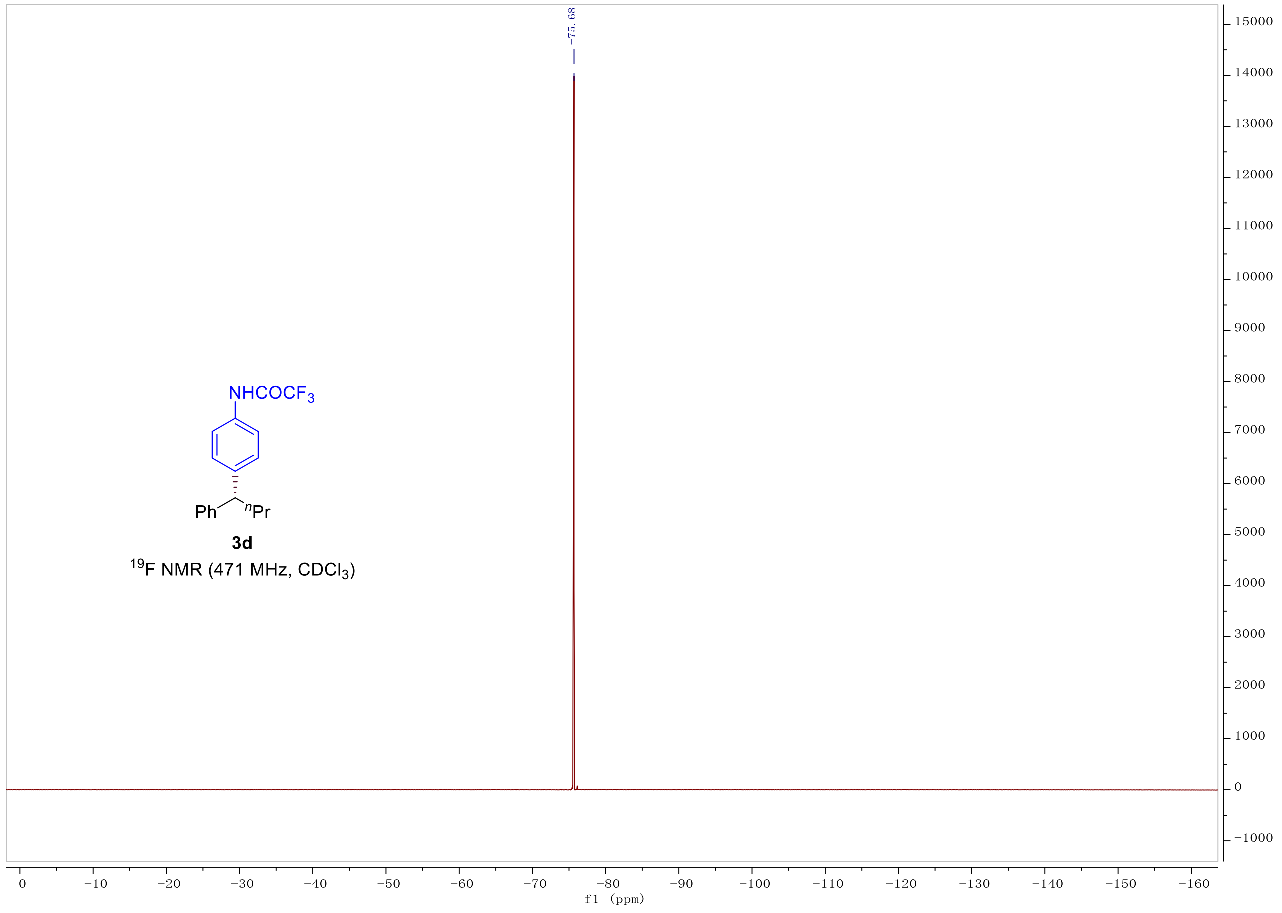

Supplementary Fig. 15. ^{19}F NMR of compound **3d**

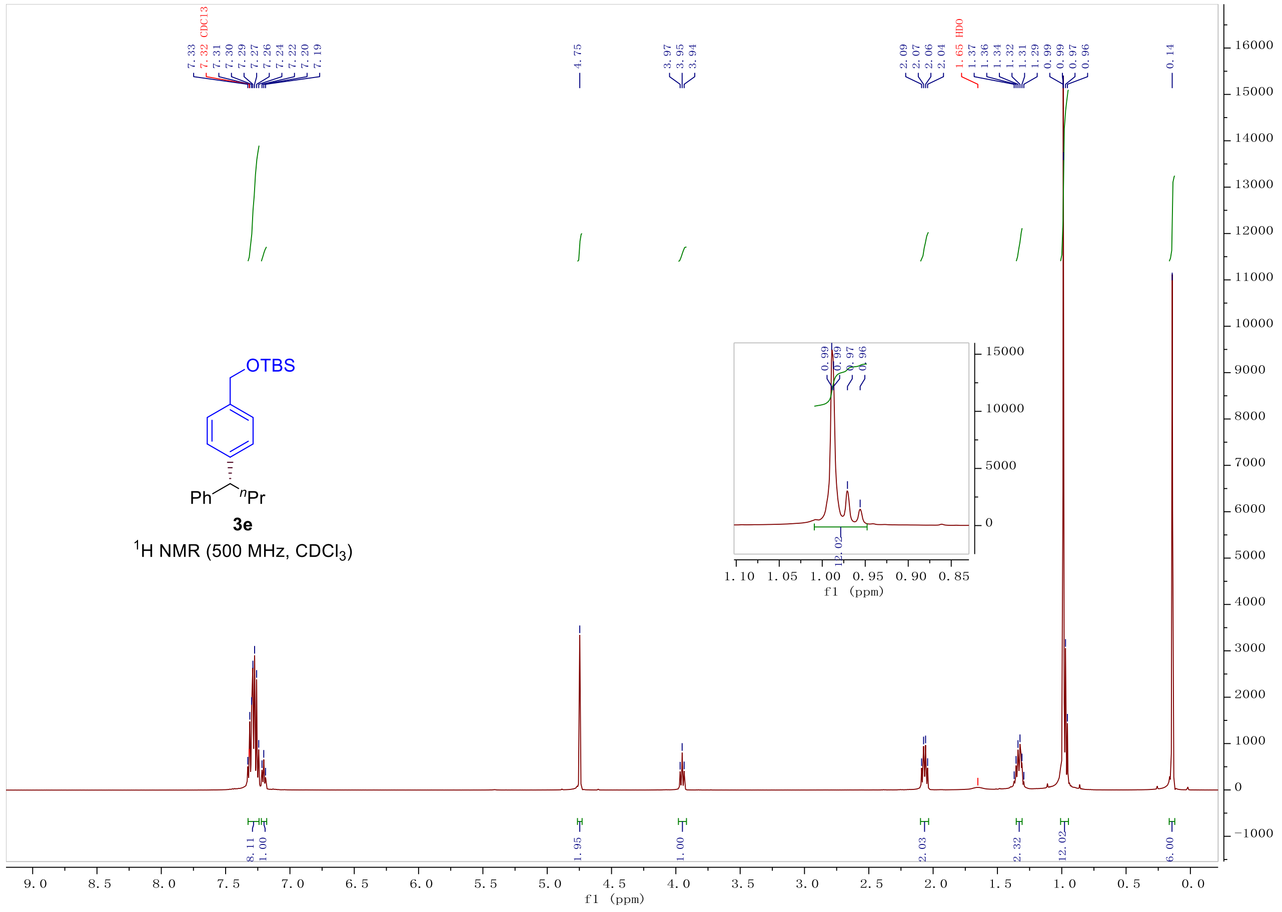

Supplementary Fig. 16. ^{1}H NMR of compound **3e**

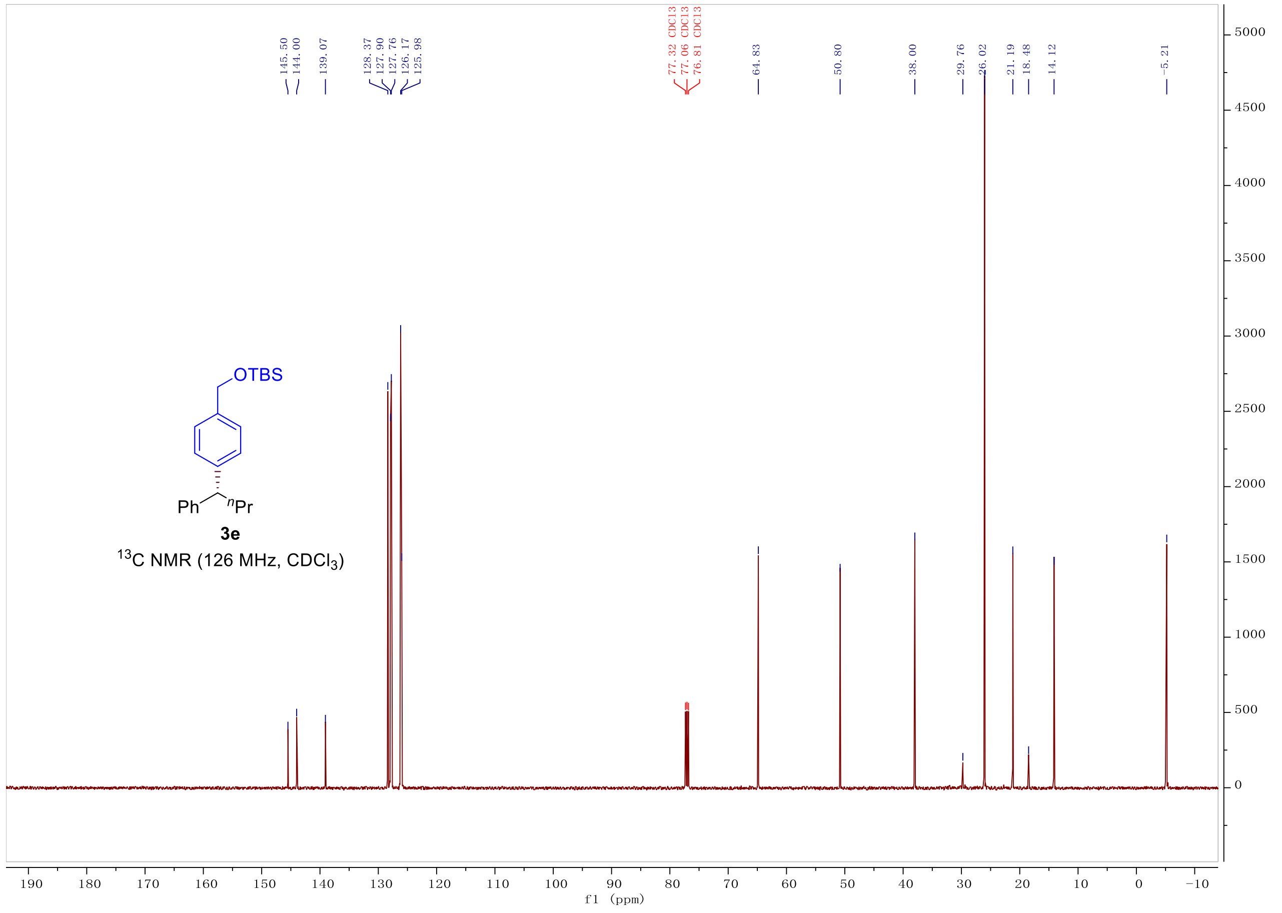

Supplementary Fig. 17. ^{13}C NMR of compound **3e**

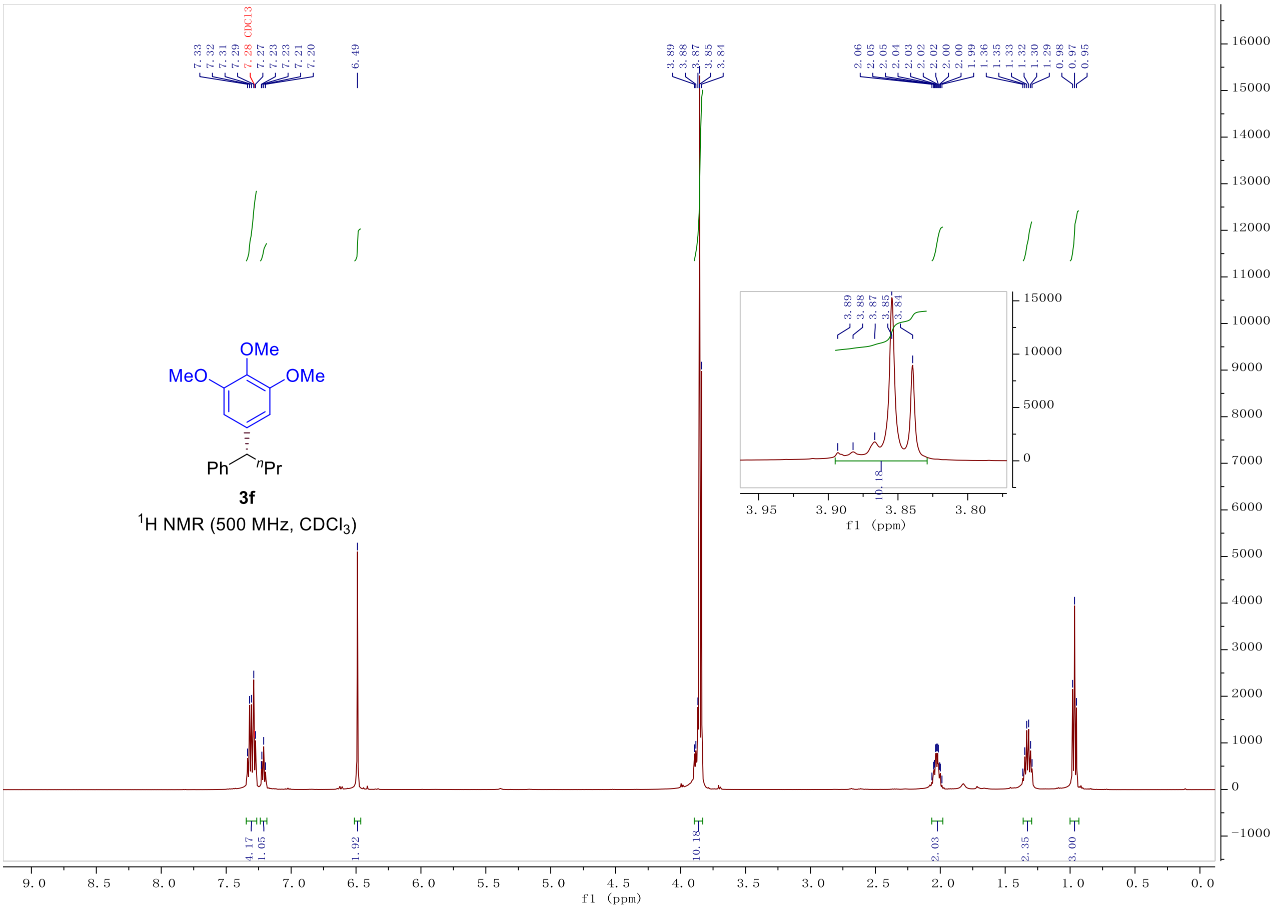

Supplementary Fig. 18. ^{1}H NMR of compound **3f**

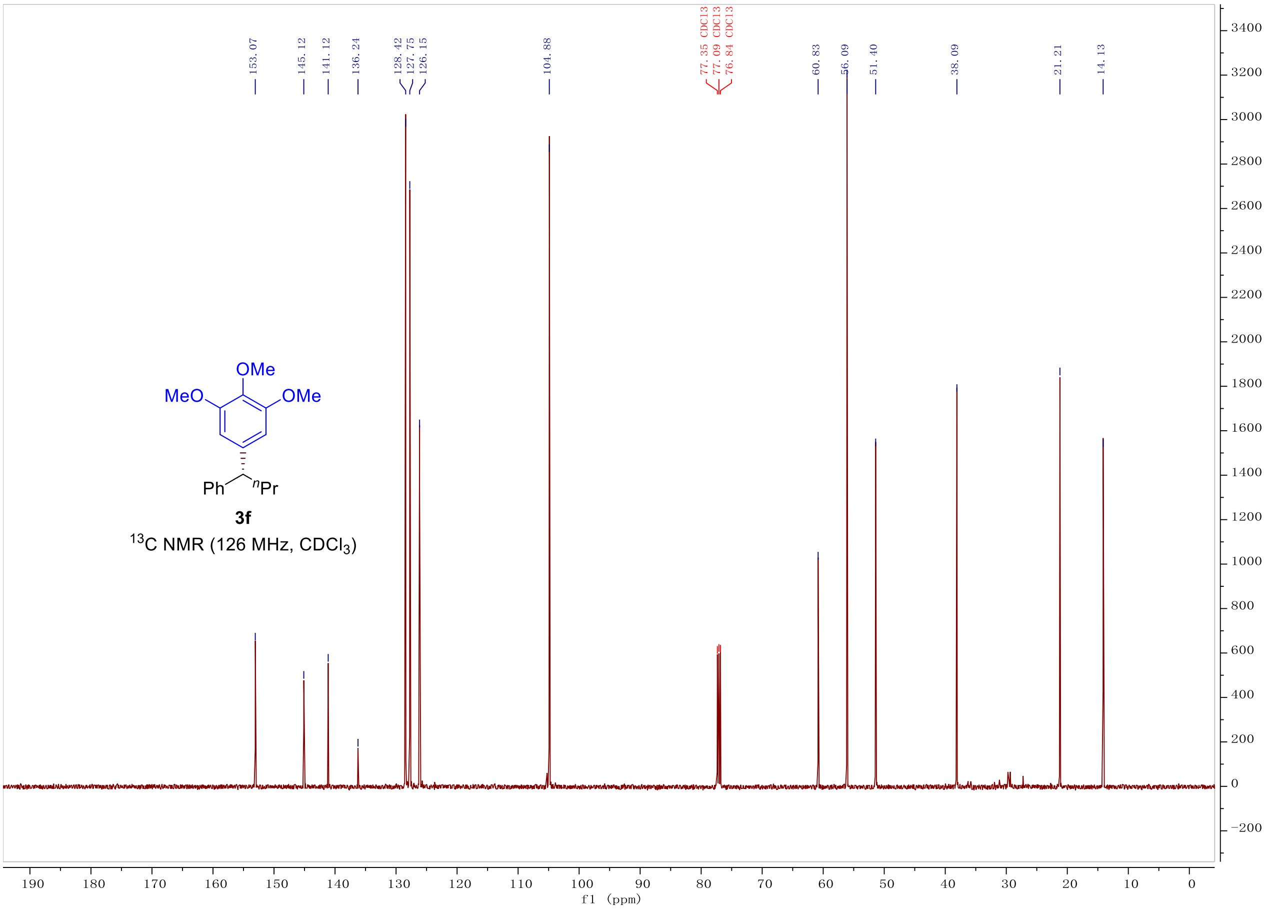

Supplementary Fig. 19. ^{13}C NMR of compound **3f**

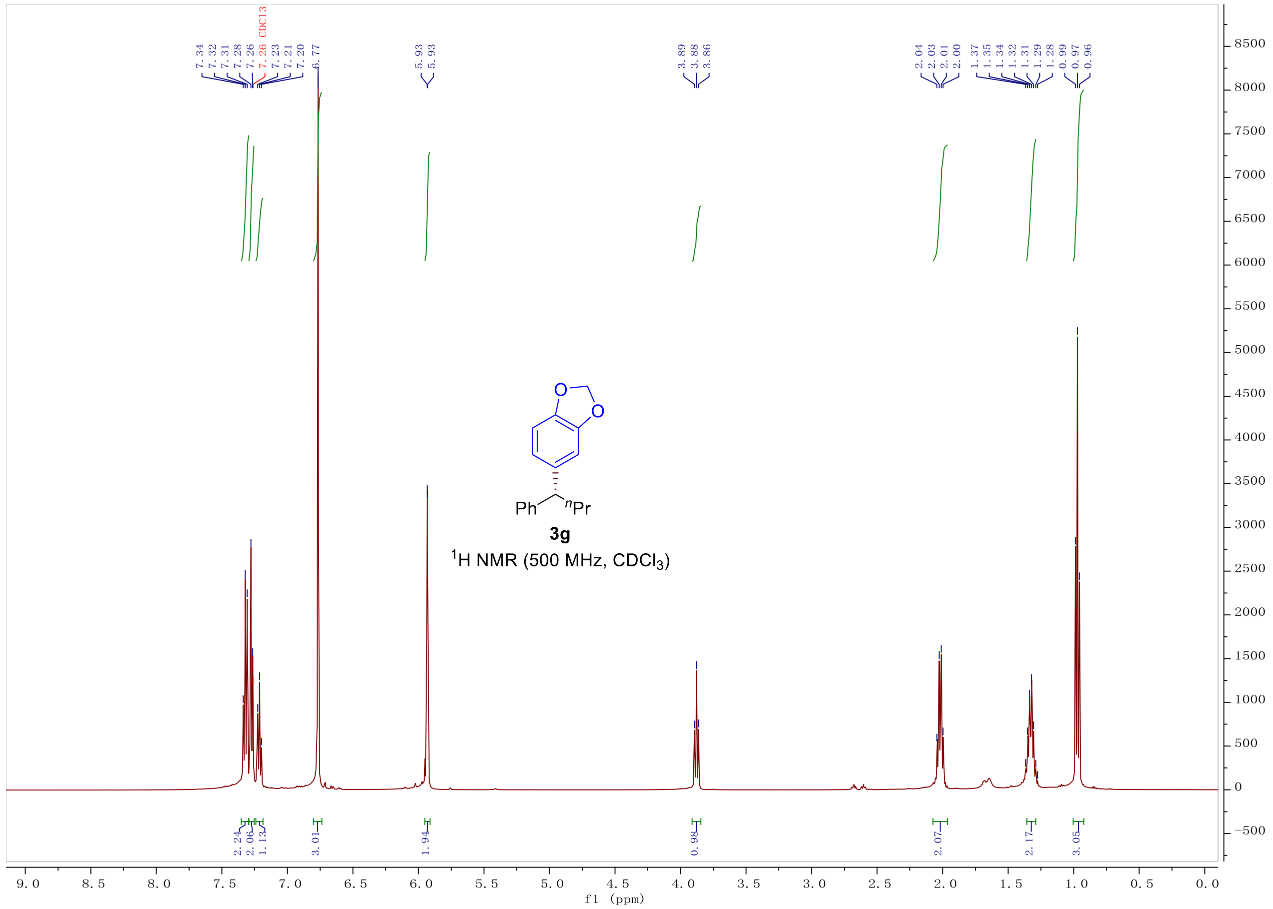

Supplementary Fig. 20. ^{1}H NMR of compound **3g**

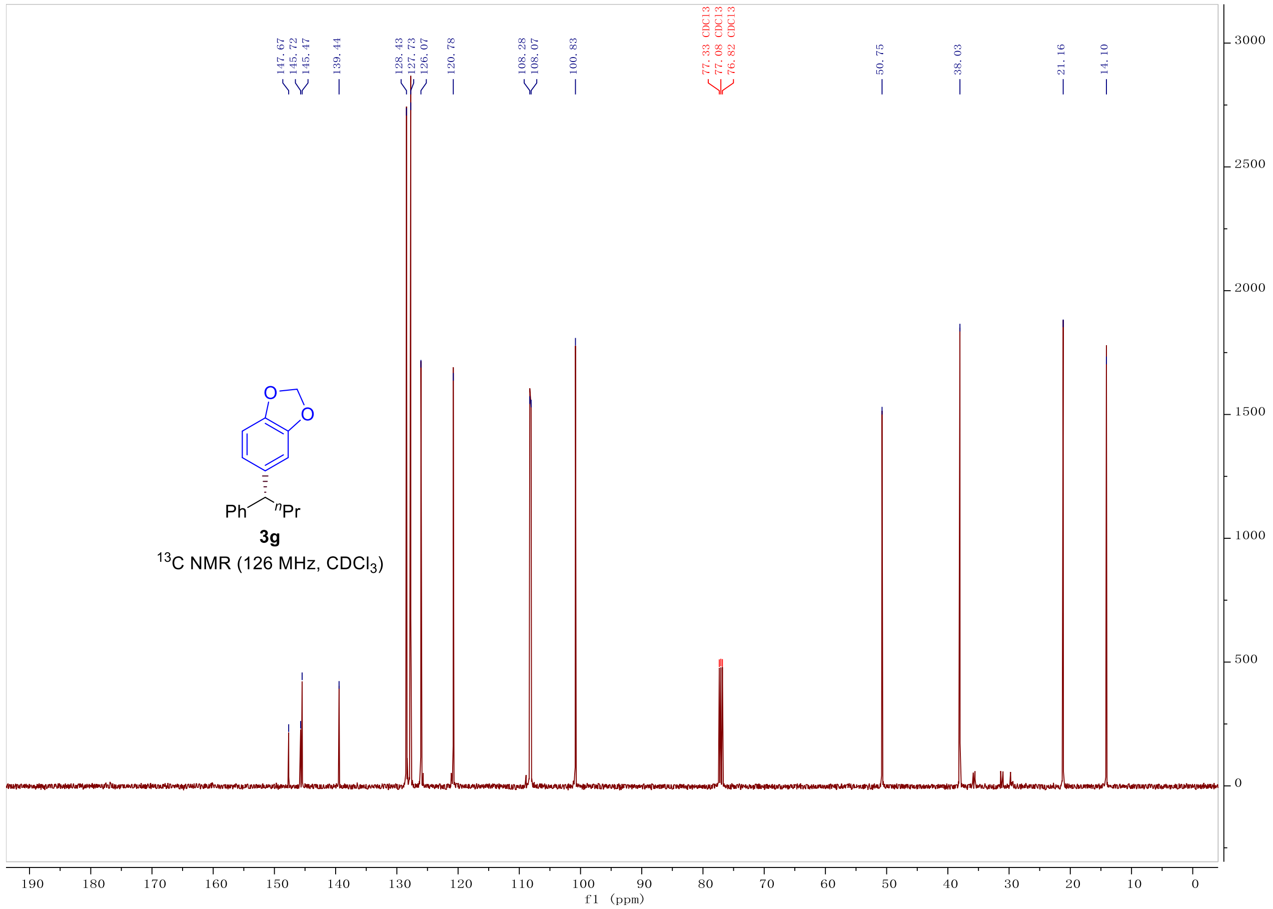

Supplementary Fig. 21. ^{13}C NMR of compound **3g**

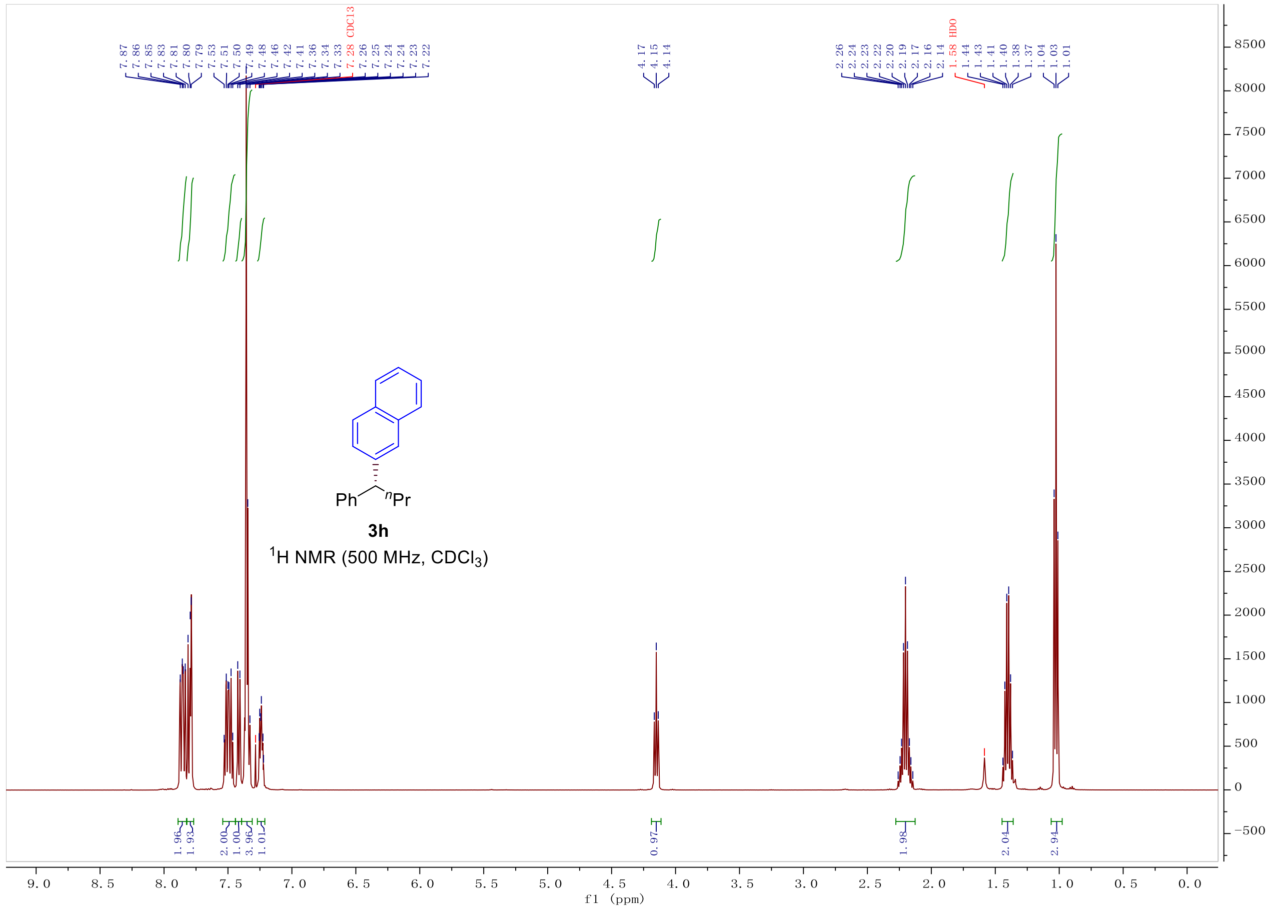

Supplementary Fig. 22. ^{1}H NMR of compound **3h**

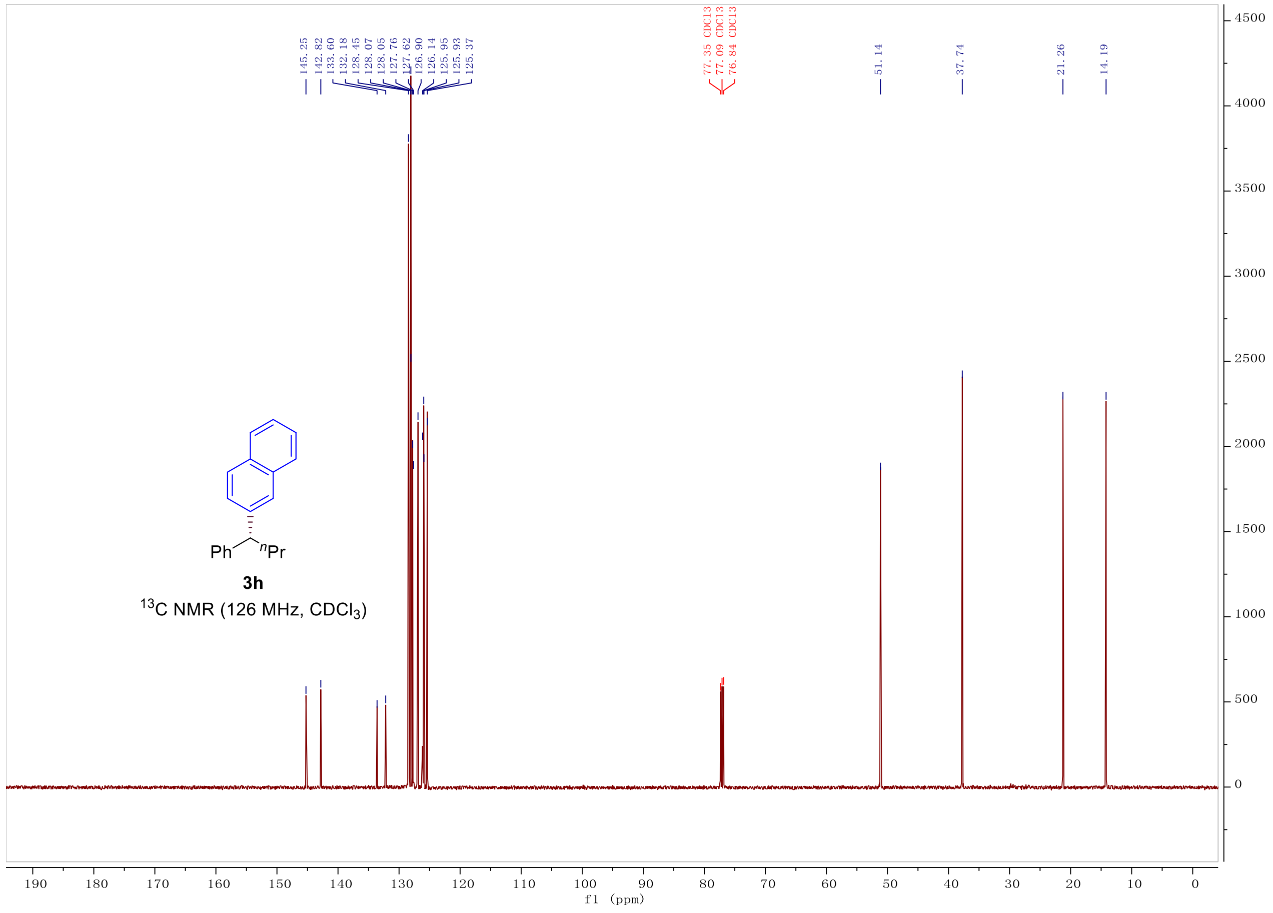

Supplementary Fig. 23. ^{13}C NMR of compound **3h**

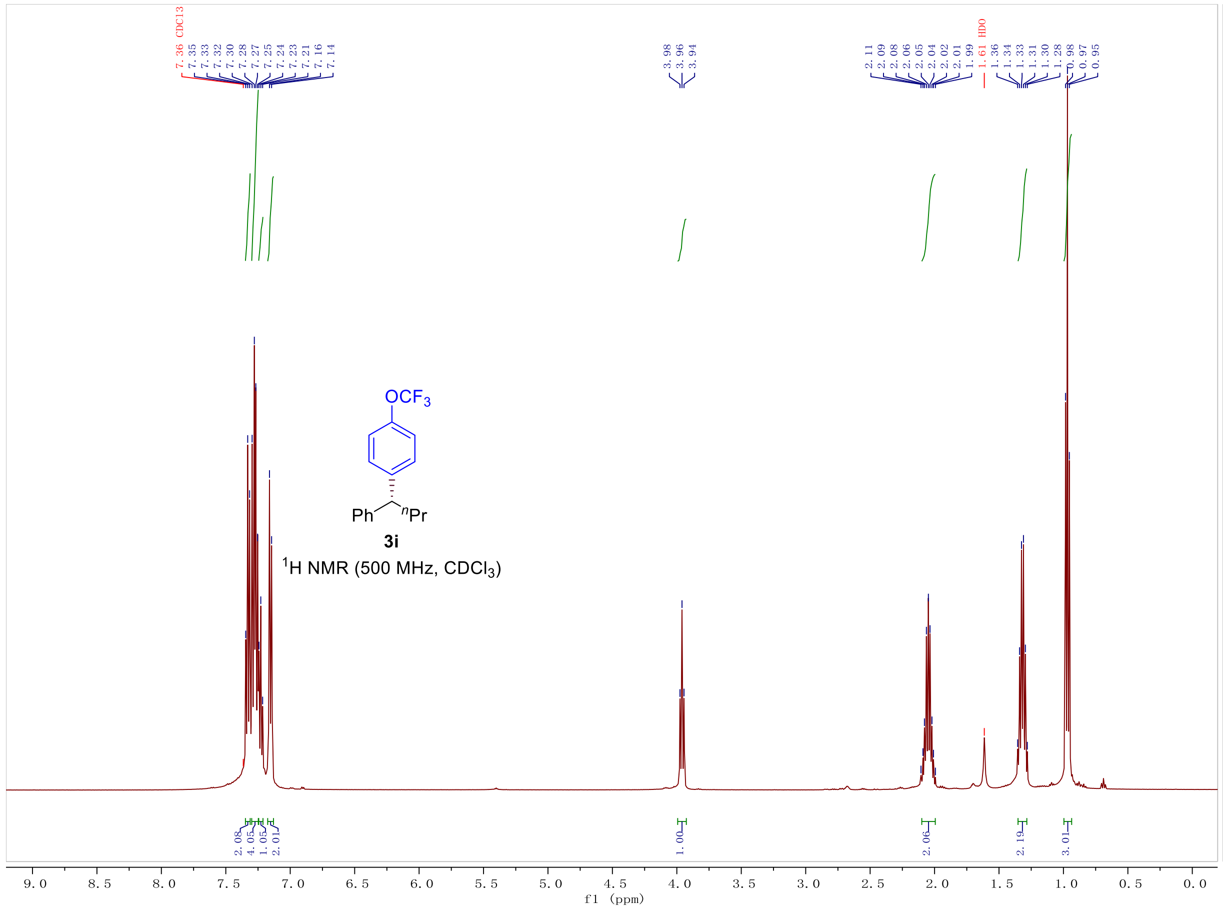

Supplementary Fig. 24. ^{1}H NMR of compound **3i**

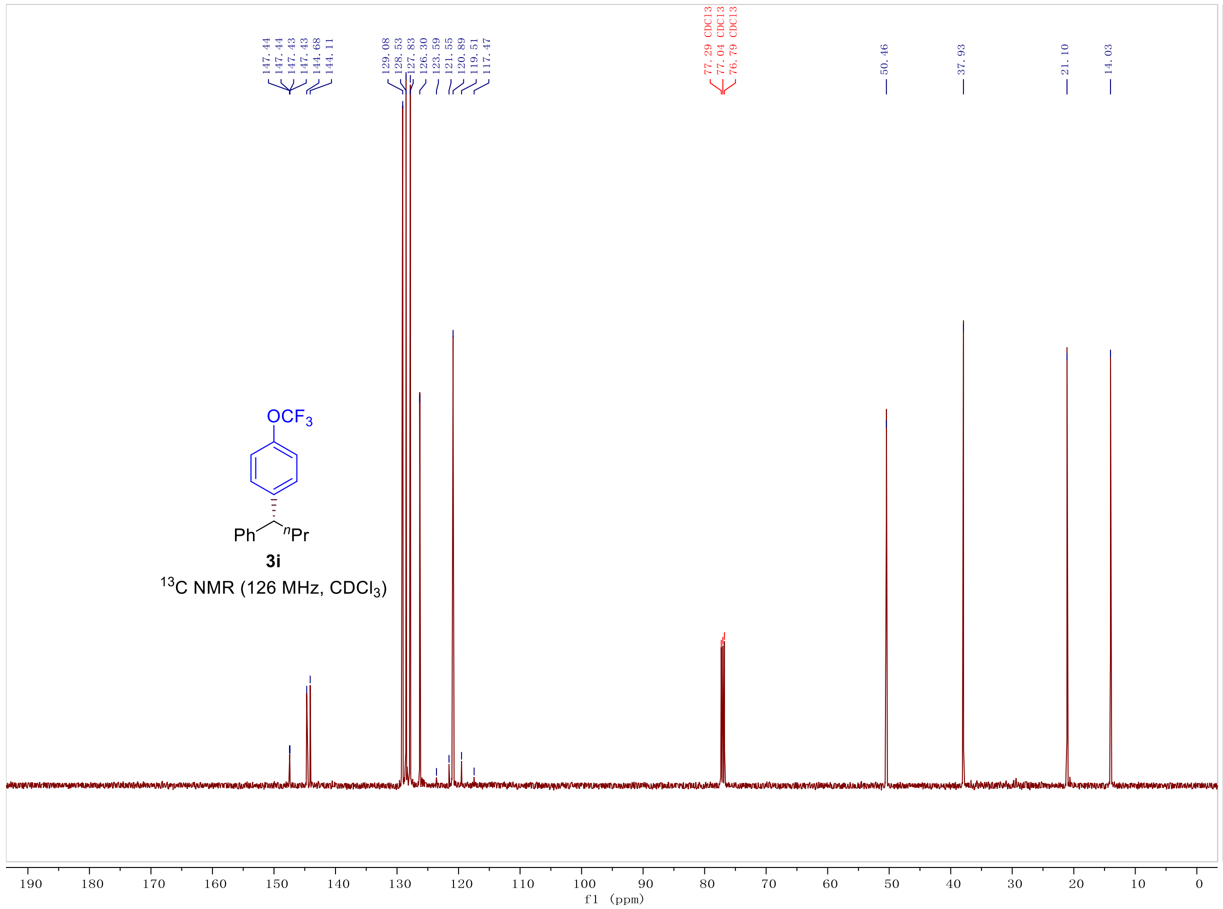

Supplementary Fig. 25. ^{13}C NMR of compound **3i**

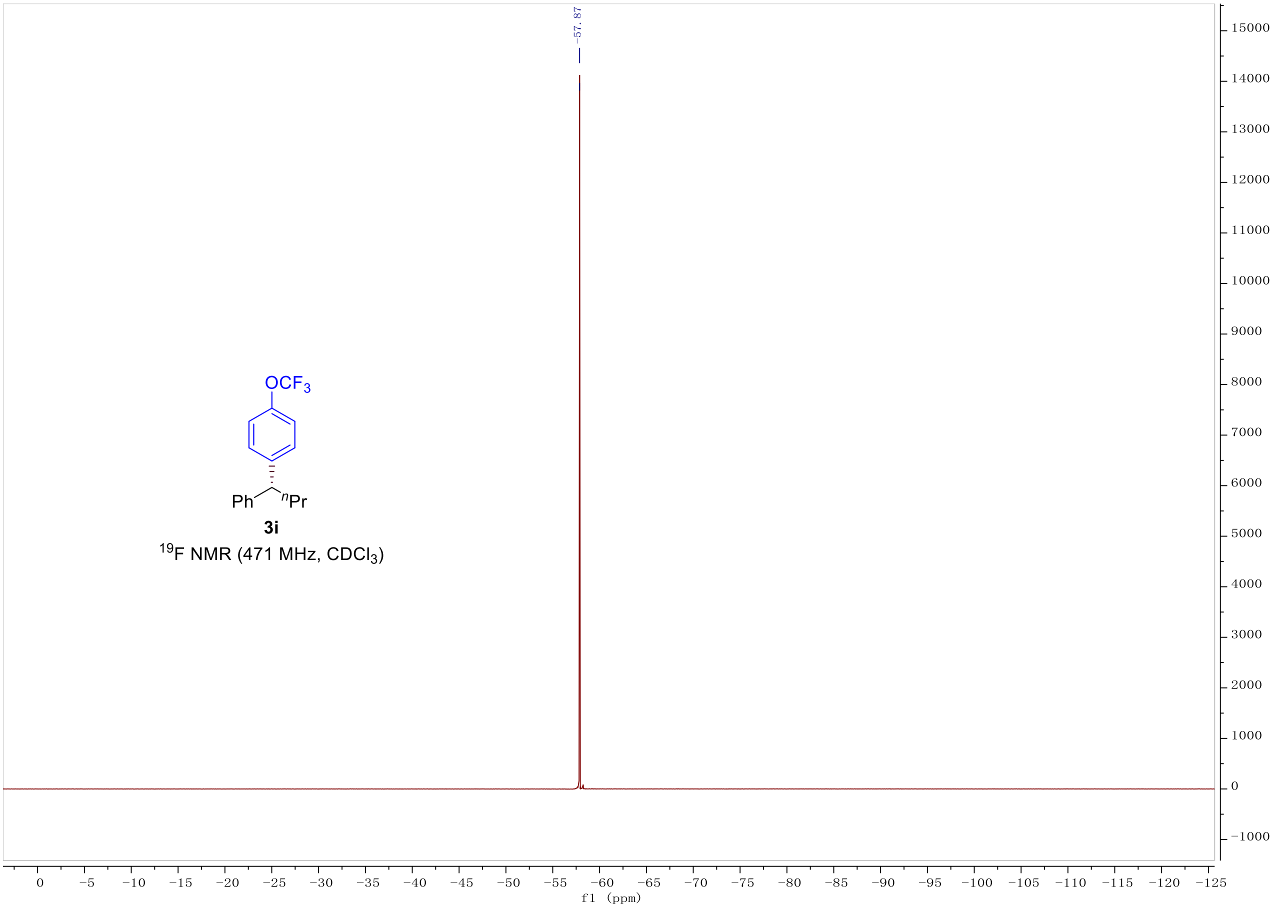

Supplementary Fig. 26. ^{19}F NMR of compound **3i**

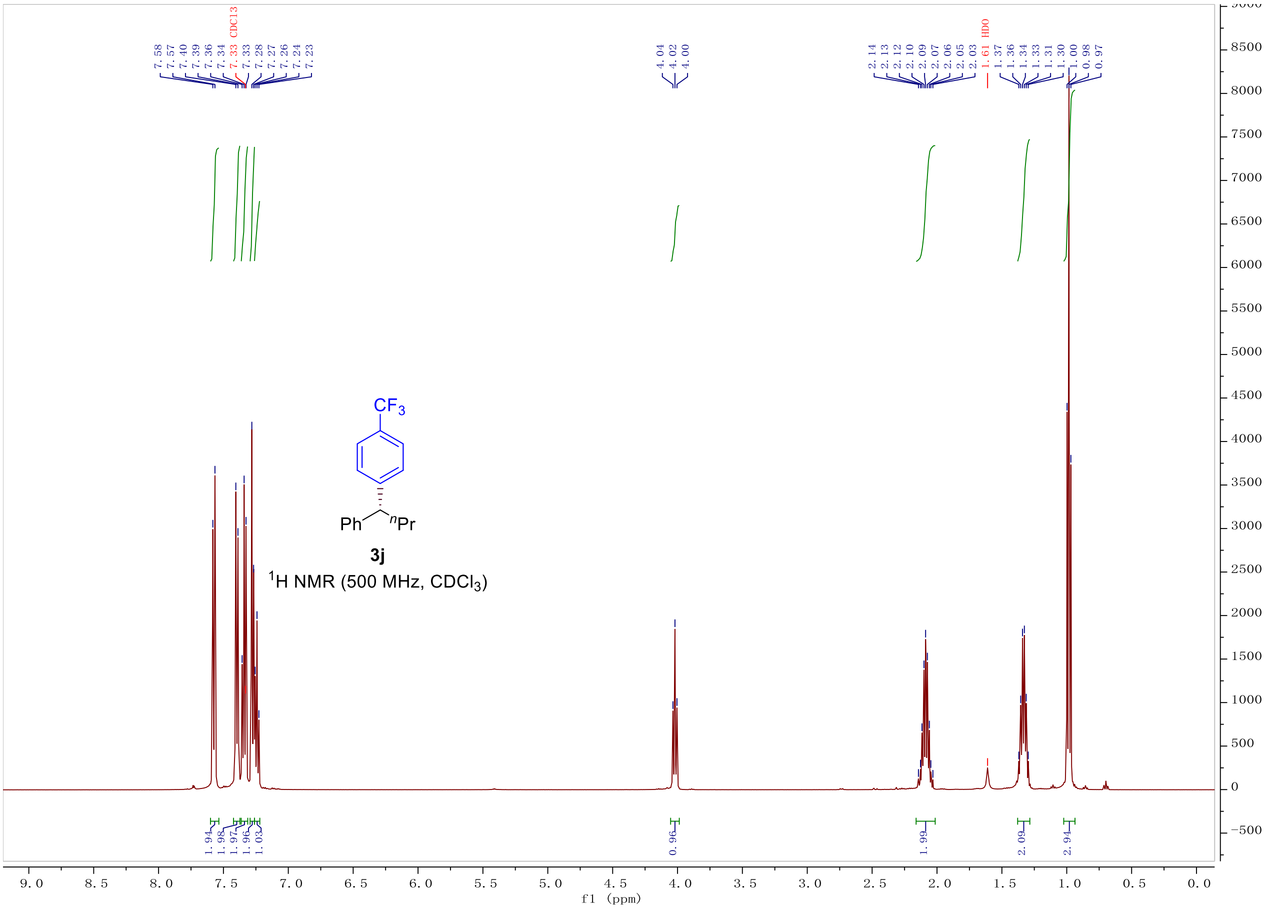

Supplementary Fig. 27. ^{1}H NMR of compound **3j**

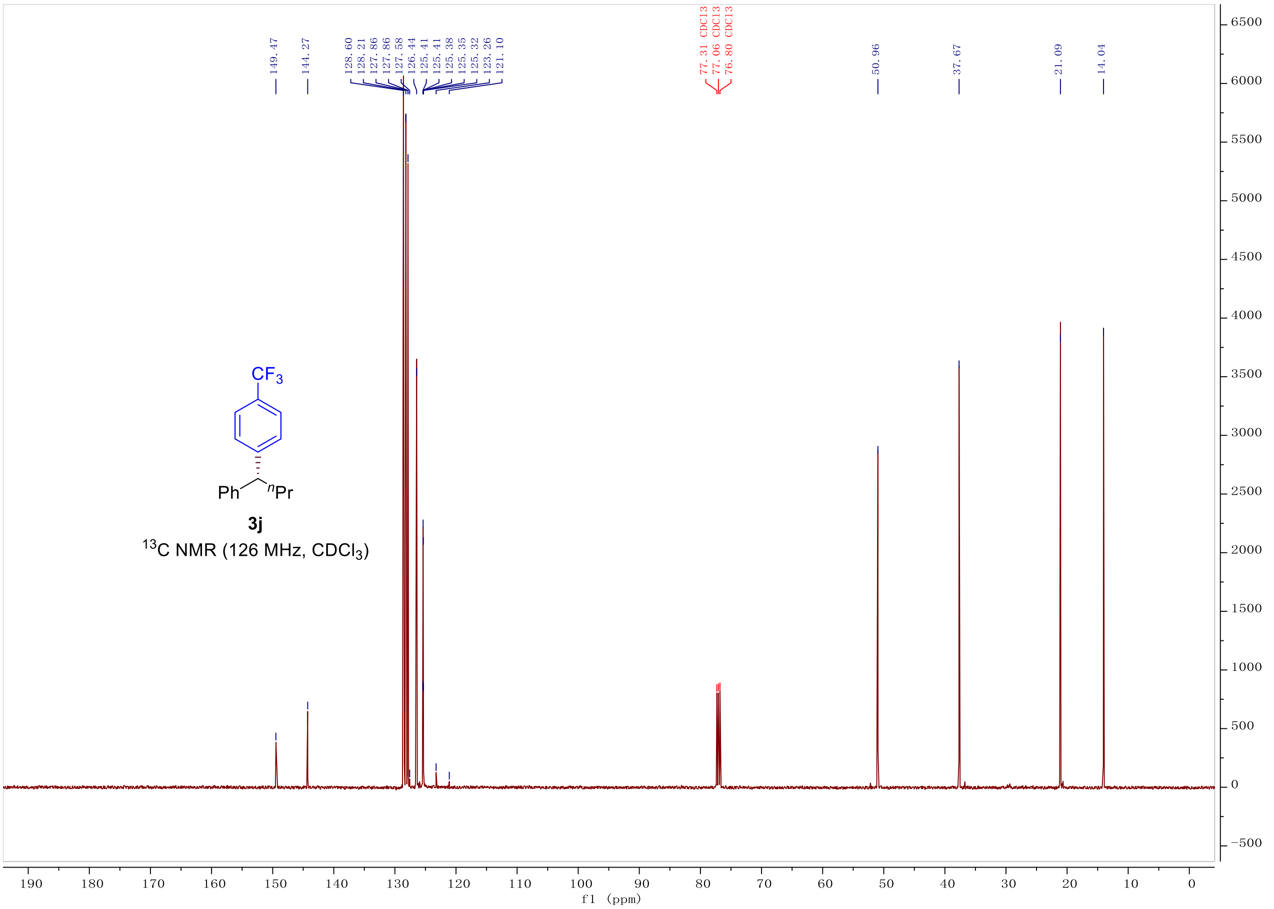

Supplementary Fig. 28. ^{13}C NMR of compound **3j**

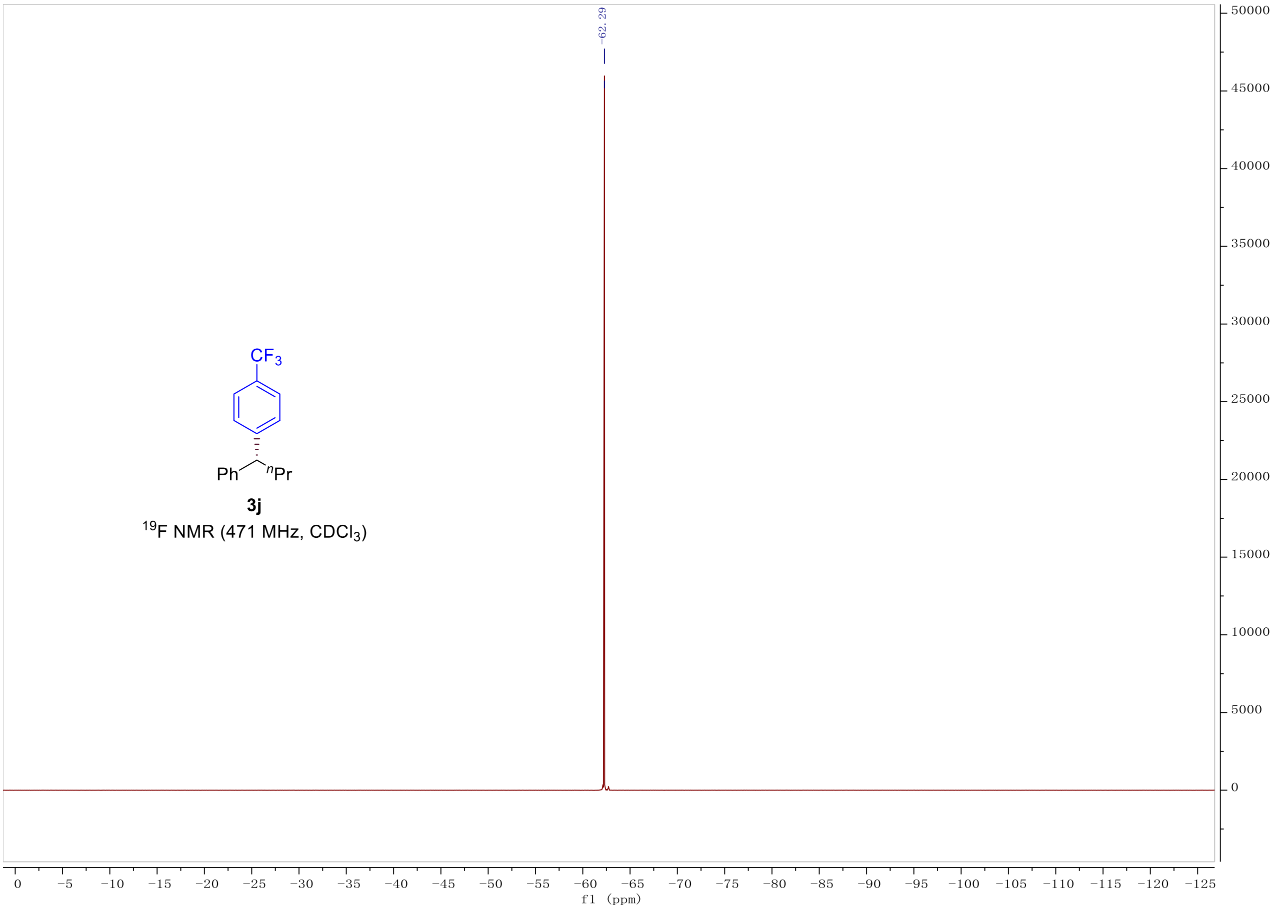

Supplementary Fig. 29. ^{19}F NMR of compound **3j**

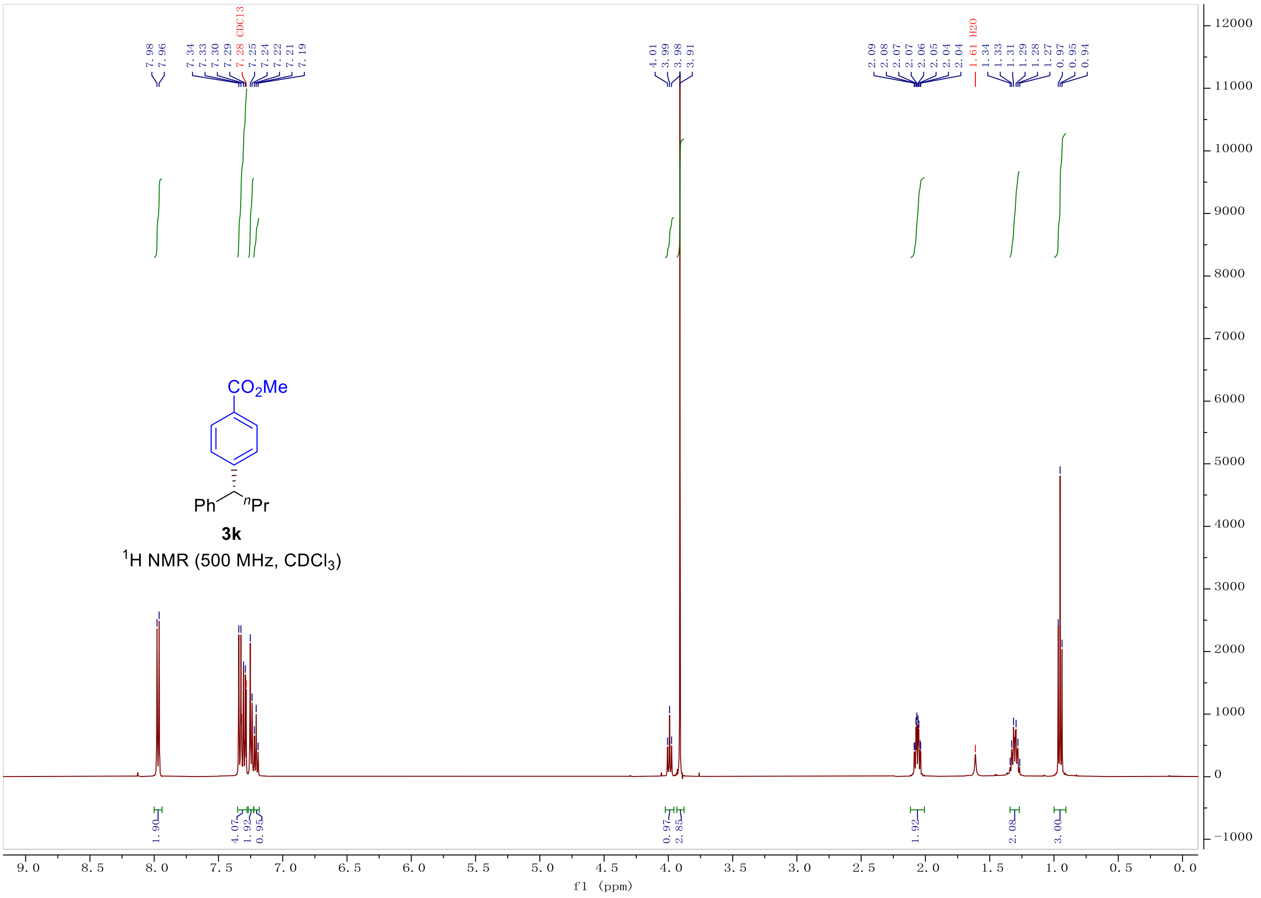

Supplementary Fig. 30. ^{1}H NMR of compound **3k**

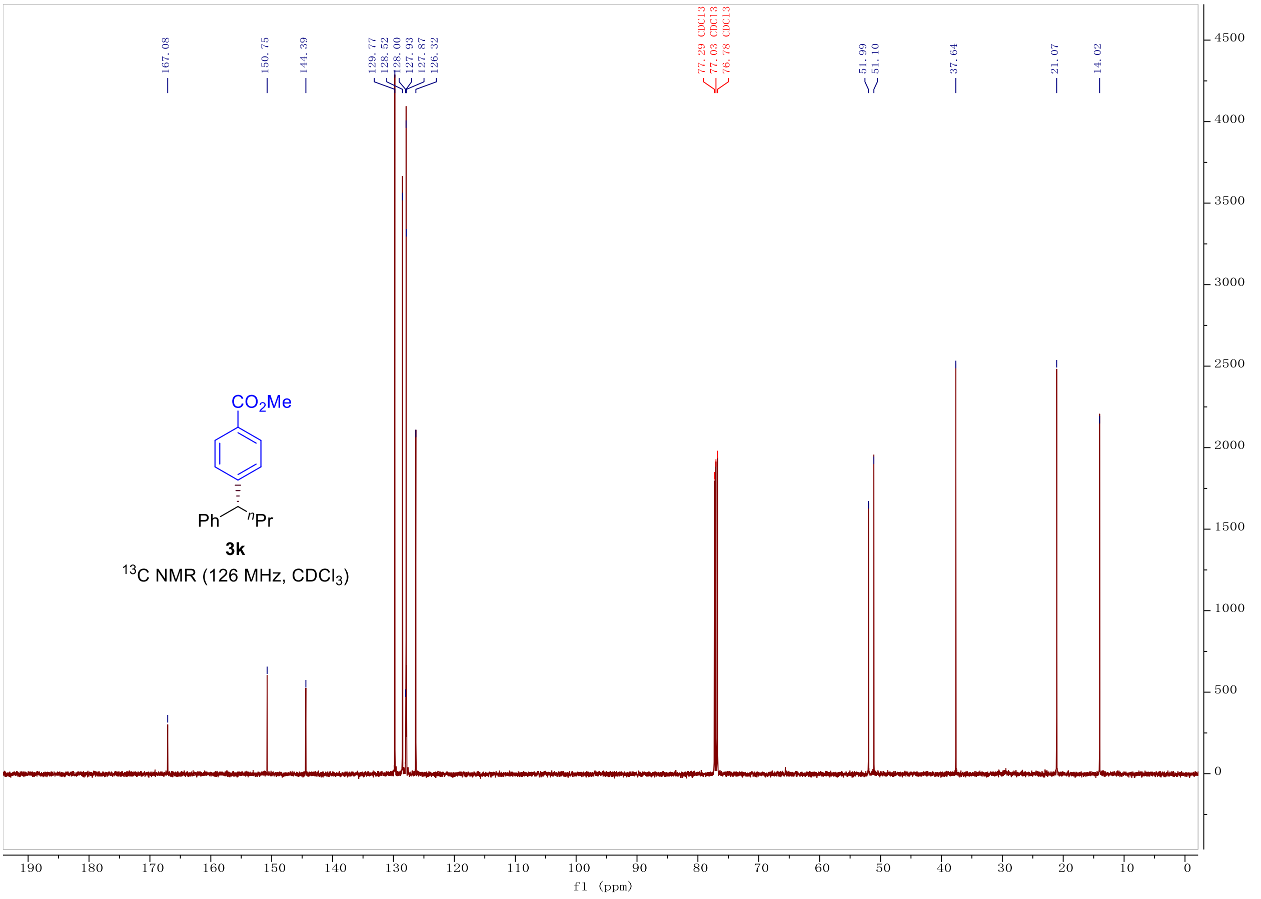

Supplementary Fig. 31. ^{13}C NMR of compound **3k**

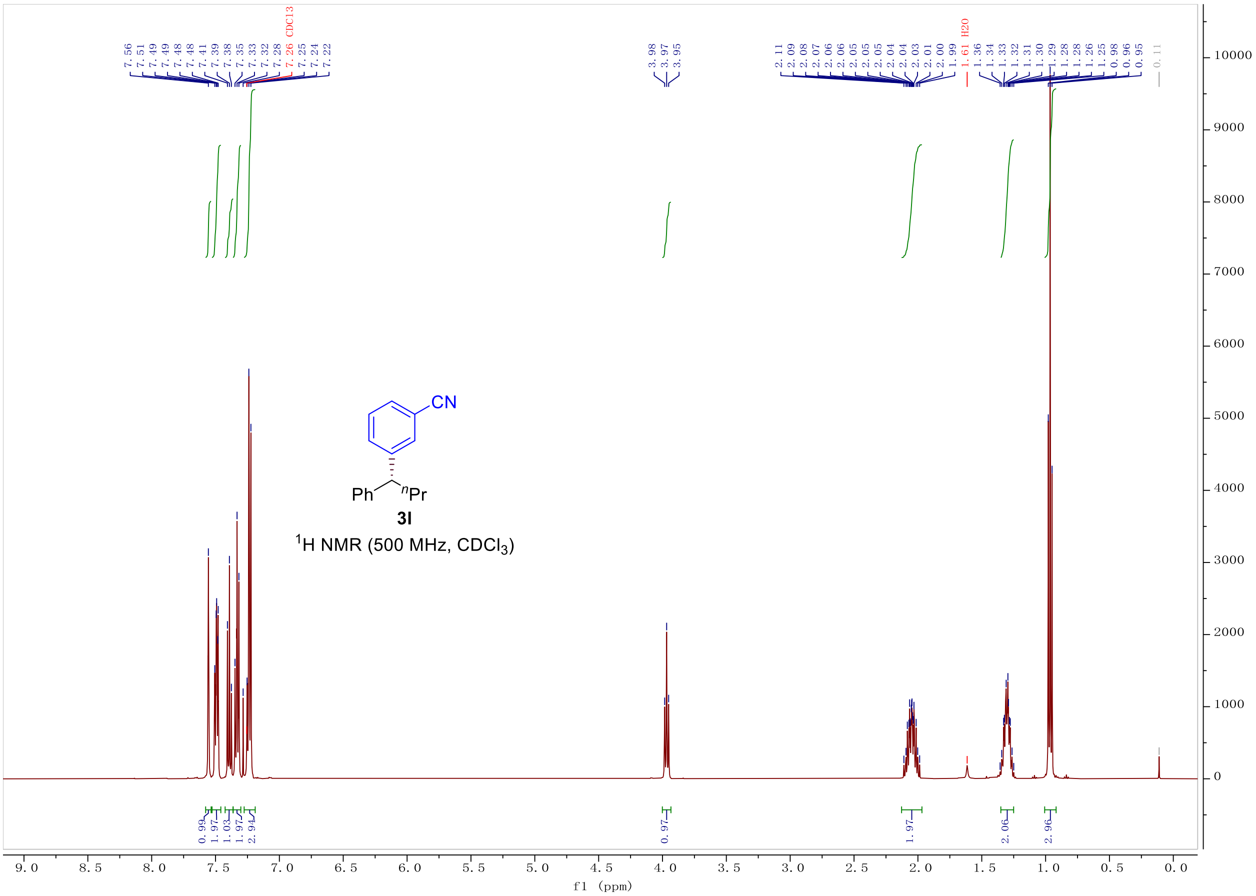

Supplementary Fig. 32. ^{1}H NMR of compound **3l**

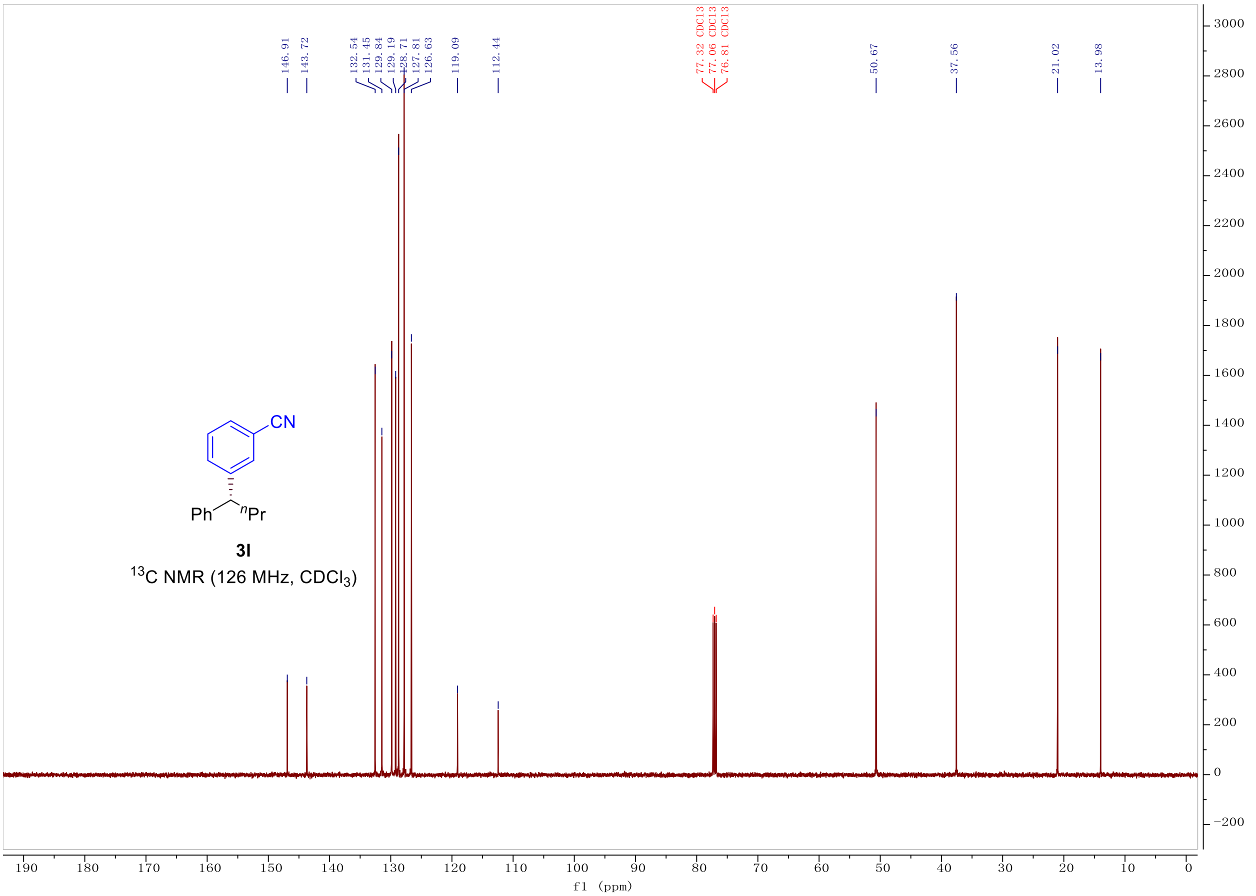

Supplementary Fig. 33. ^{13}C NMR of compound **3l**

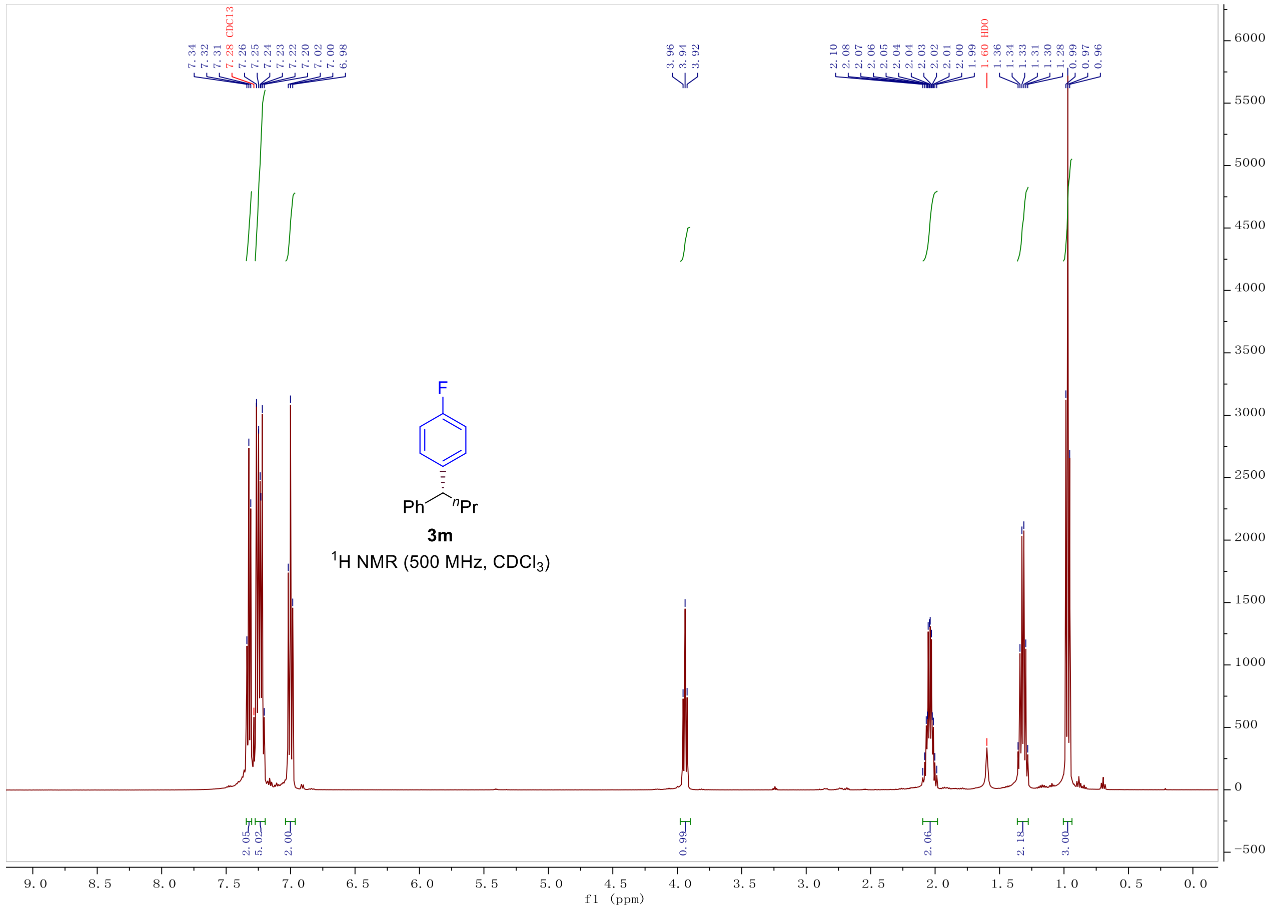

Supplementary Fig. 34. ^{1}H NMR of compound **3m**

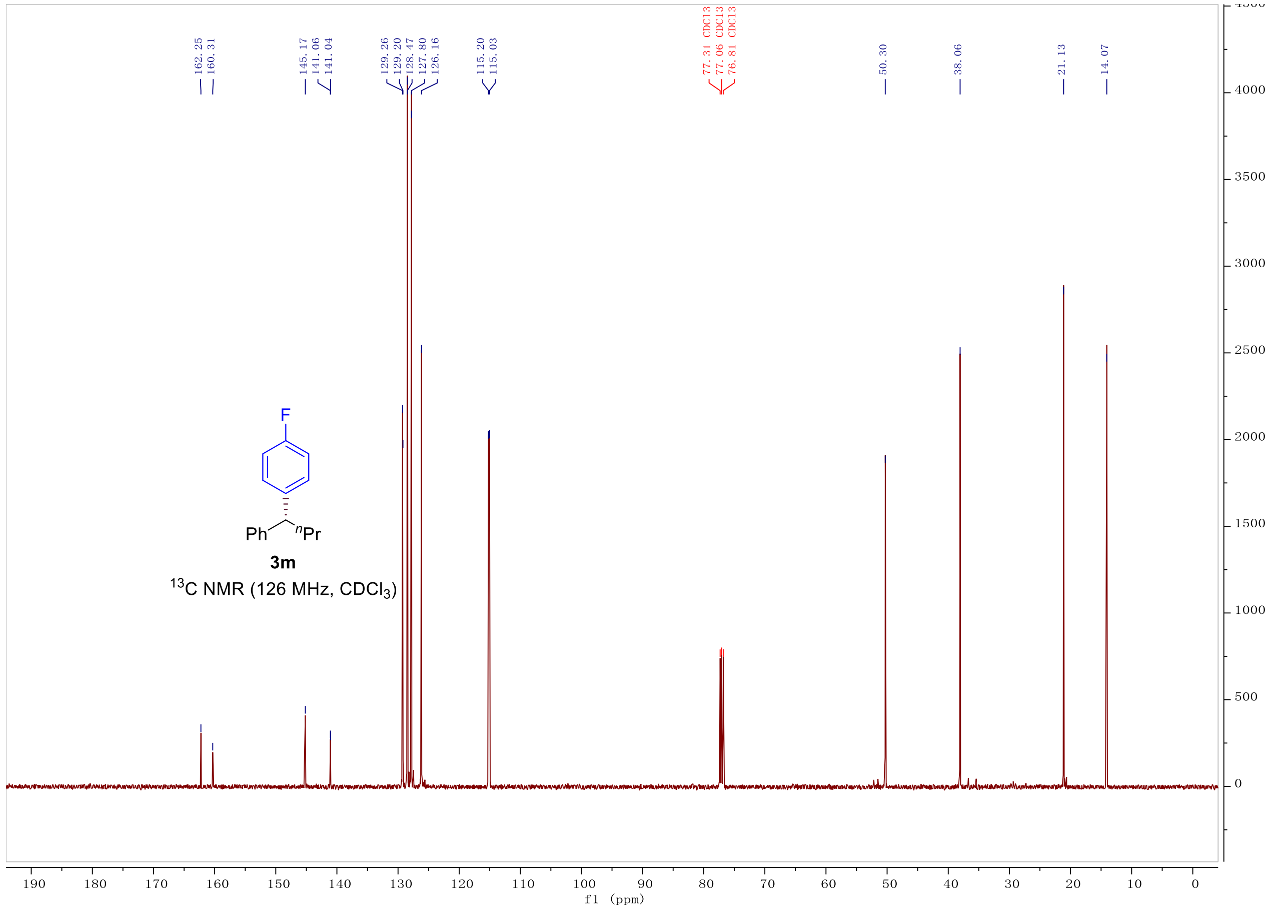

Supplementary Fig. 35. ^{13}C NMR of compound **3m**

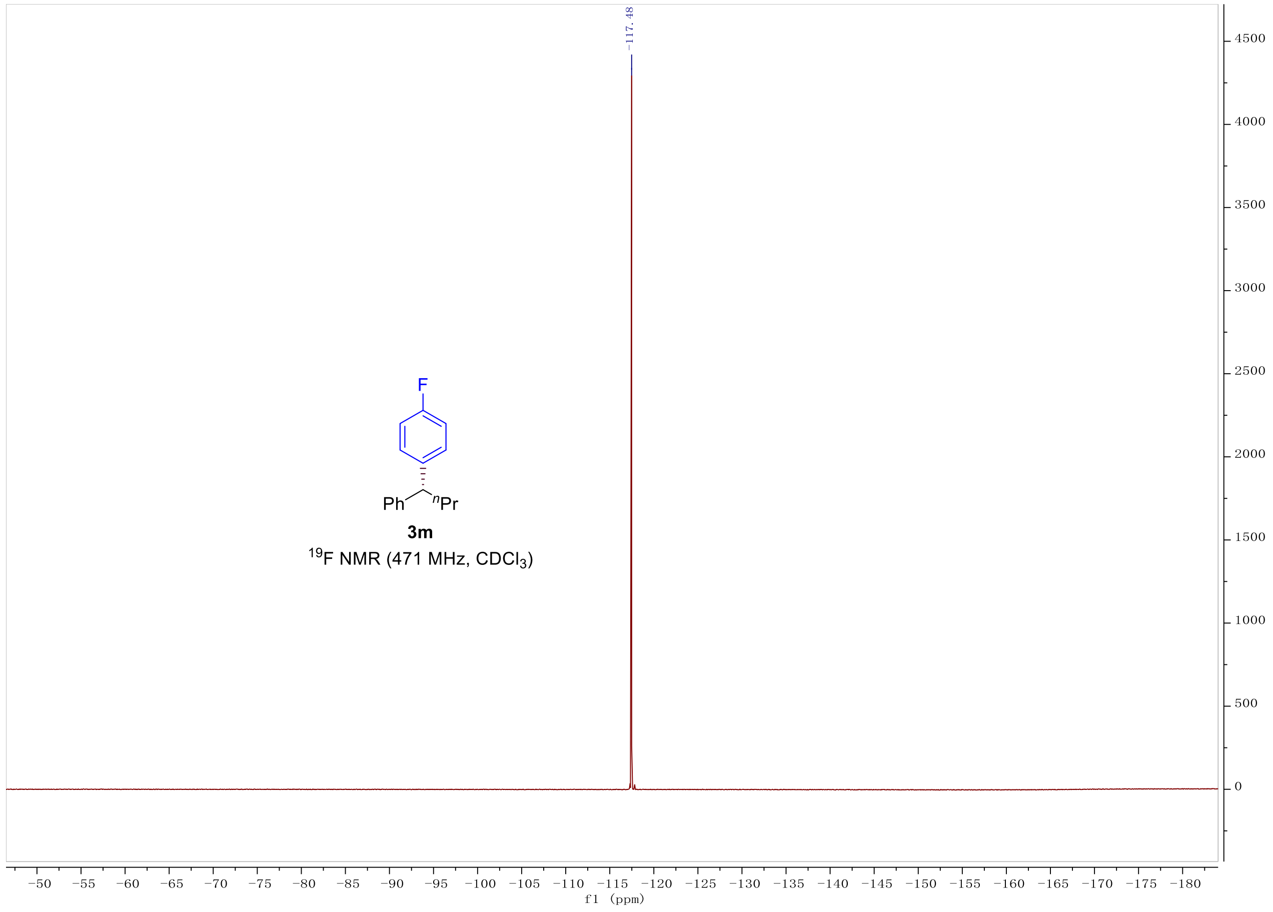

Supplementary Fig. 36. ^{19}F NMR of compound **3m**

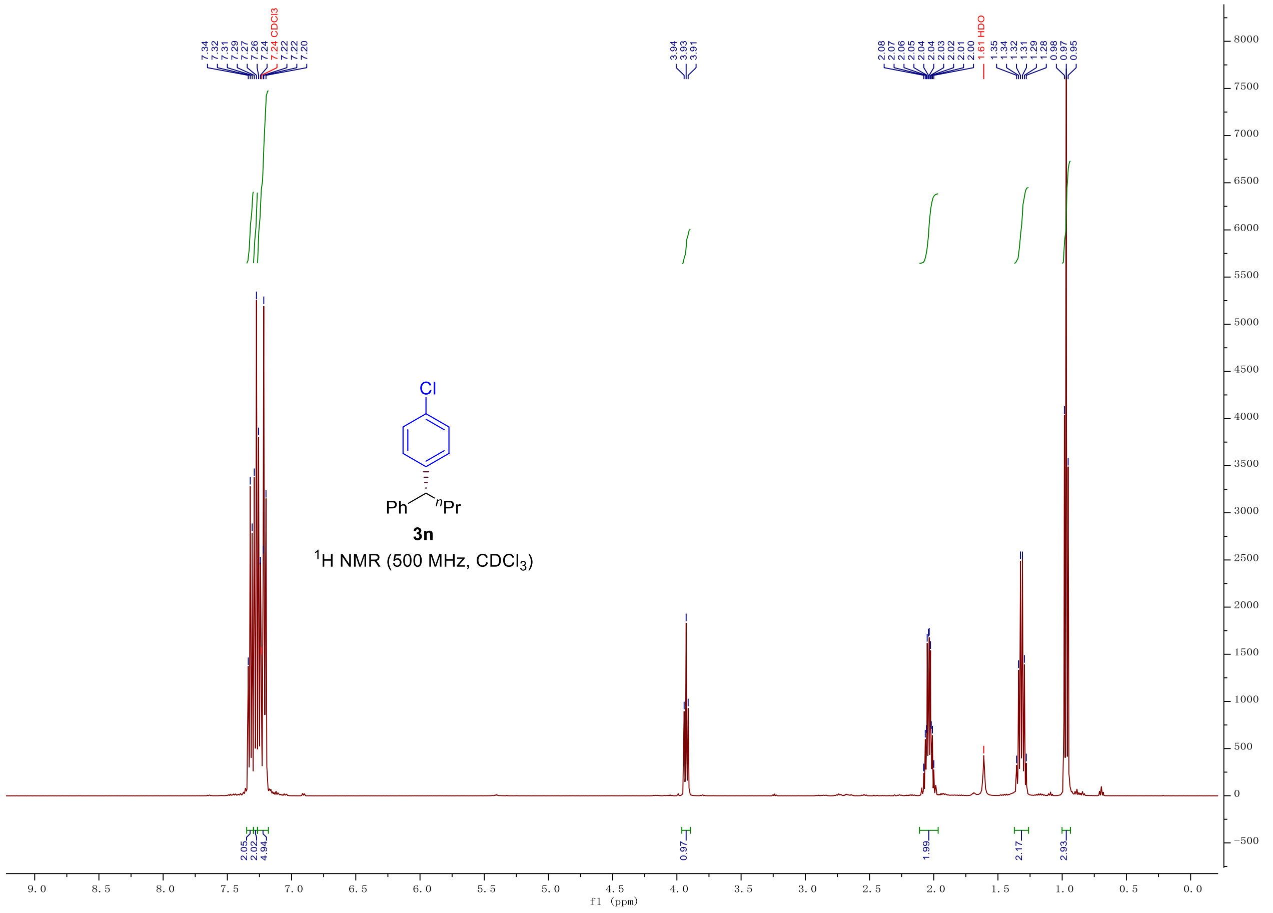

Supplementary Fig. 37. ^{1}H NMR of compound **3n**

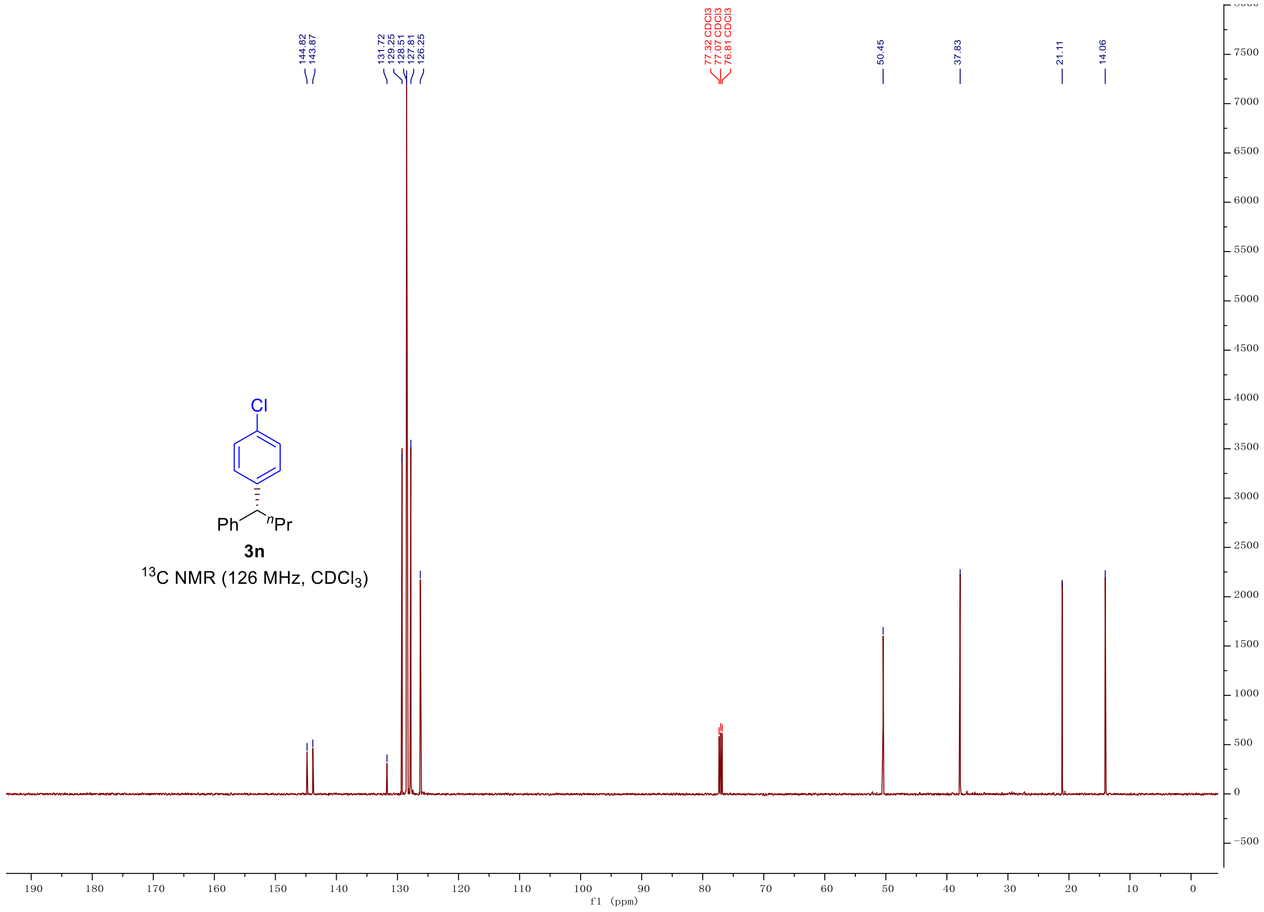

Supplementary Fig. 38. ^{13}C NMR of compound **3n**

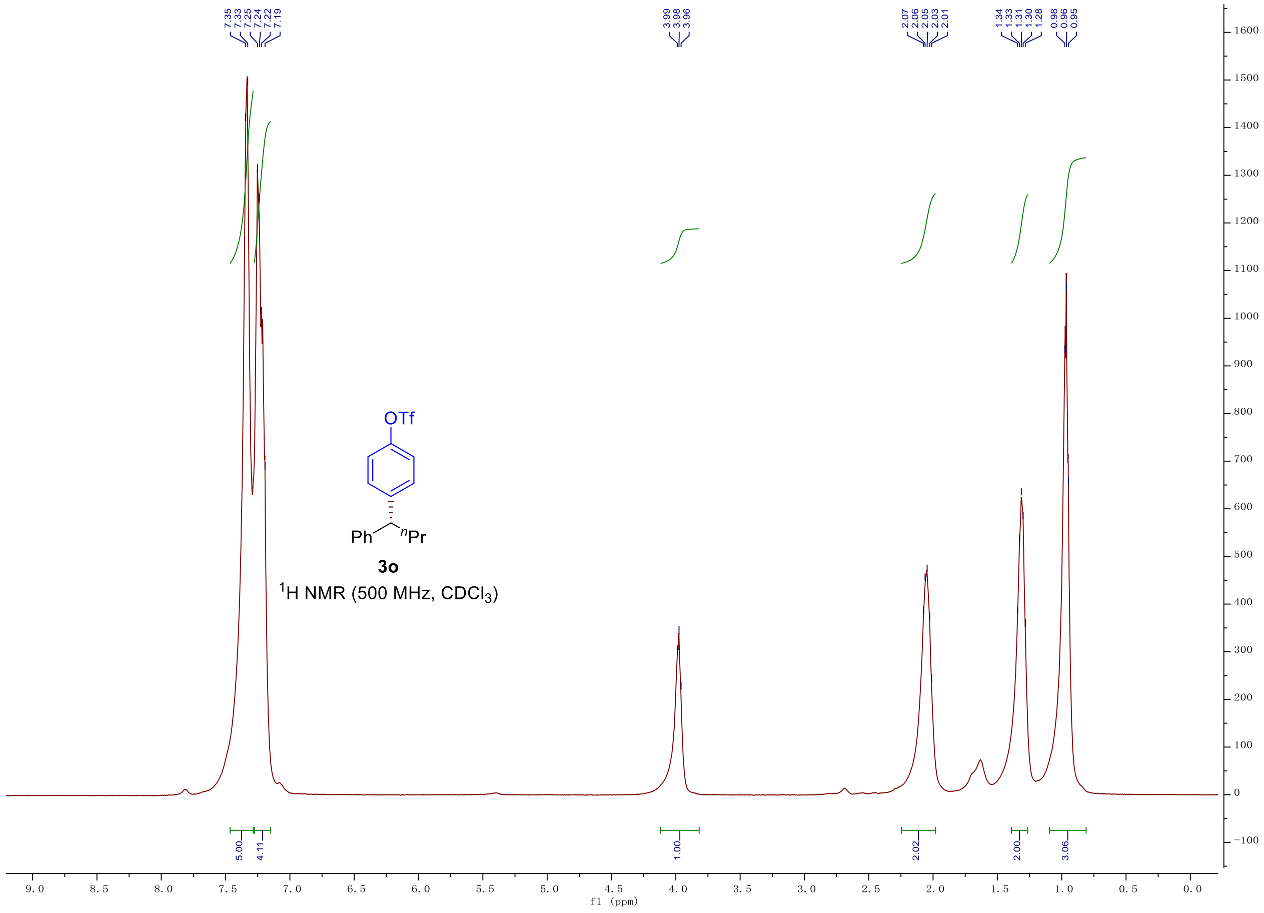

Supplementary Fig. 39. ^{1}H NMR of compound **3o**

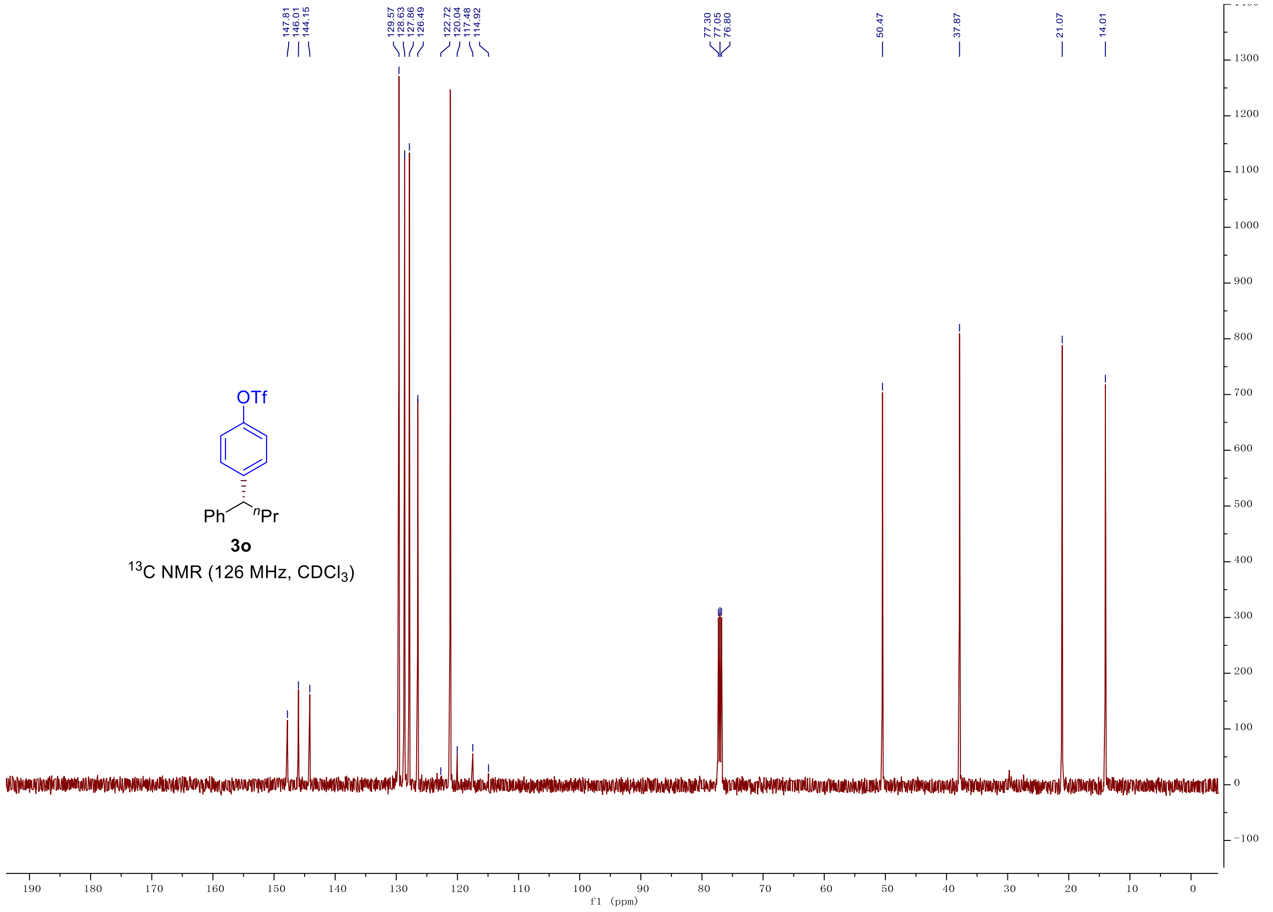

Supplementary Fig. 40. ^{13}C NMR of compound **3o**

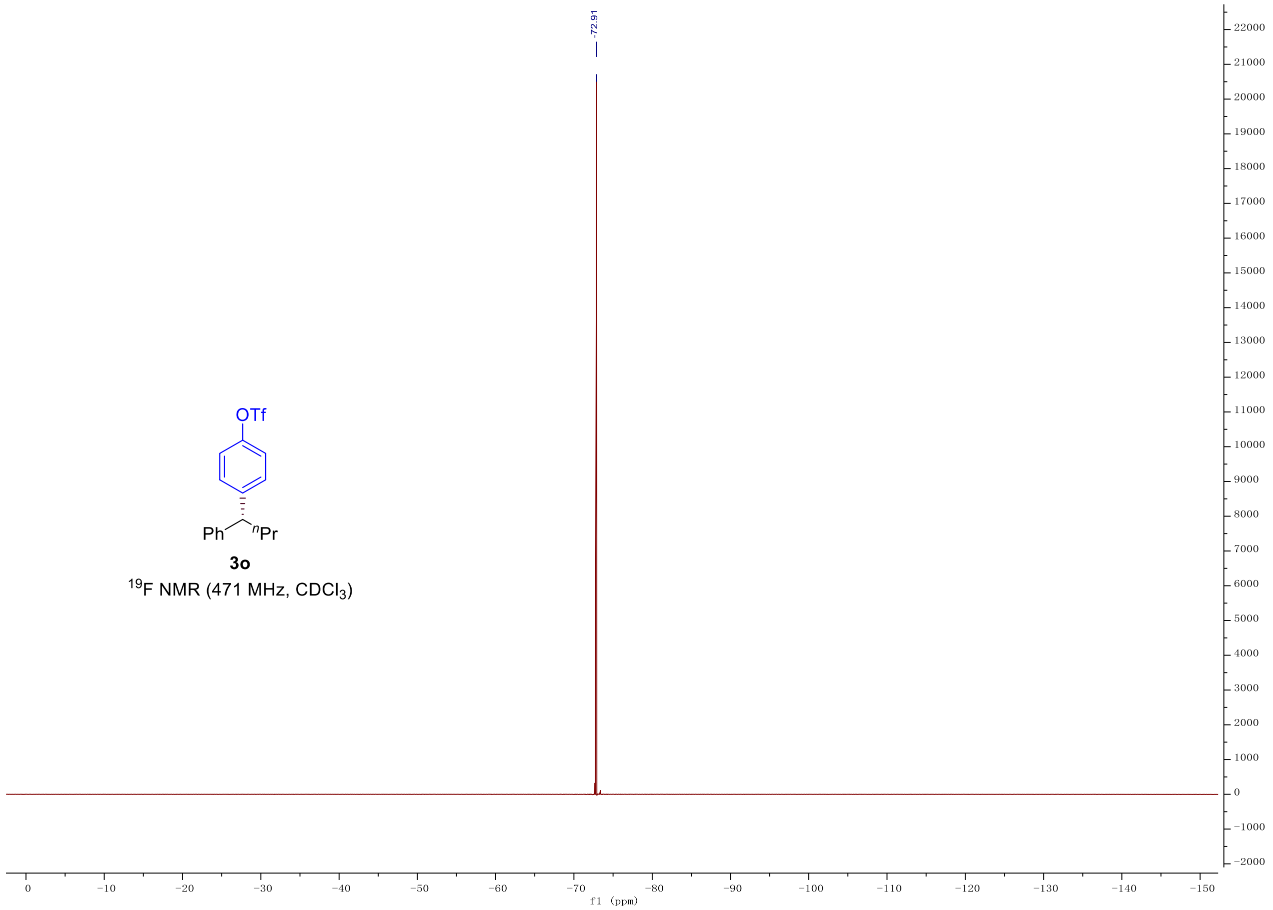

Supplementary Fig. 41. ^{19}F NMR of compound **3o**

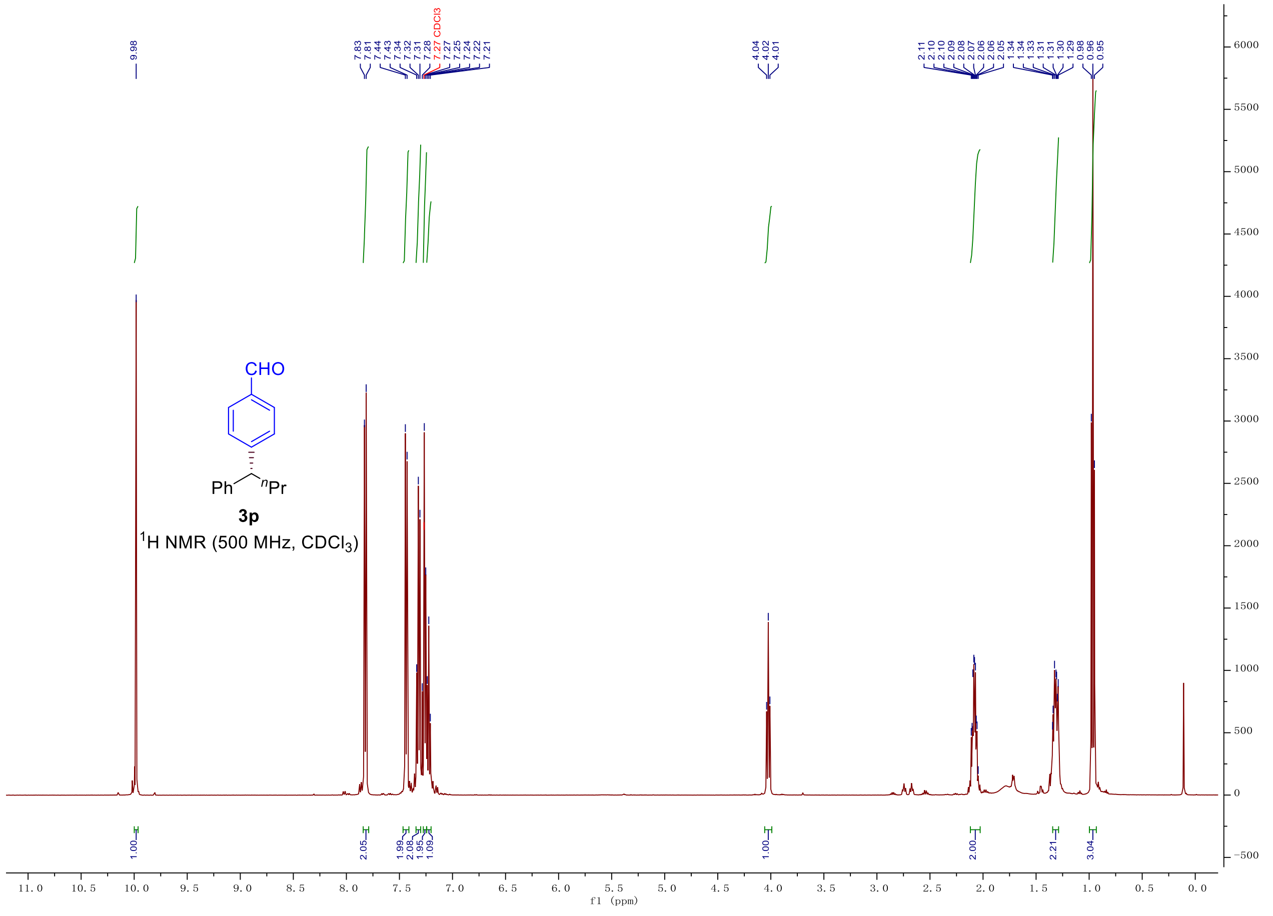

Supplementary Fig. 42. ^{1}H NMR of compound **3p**

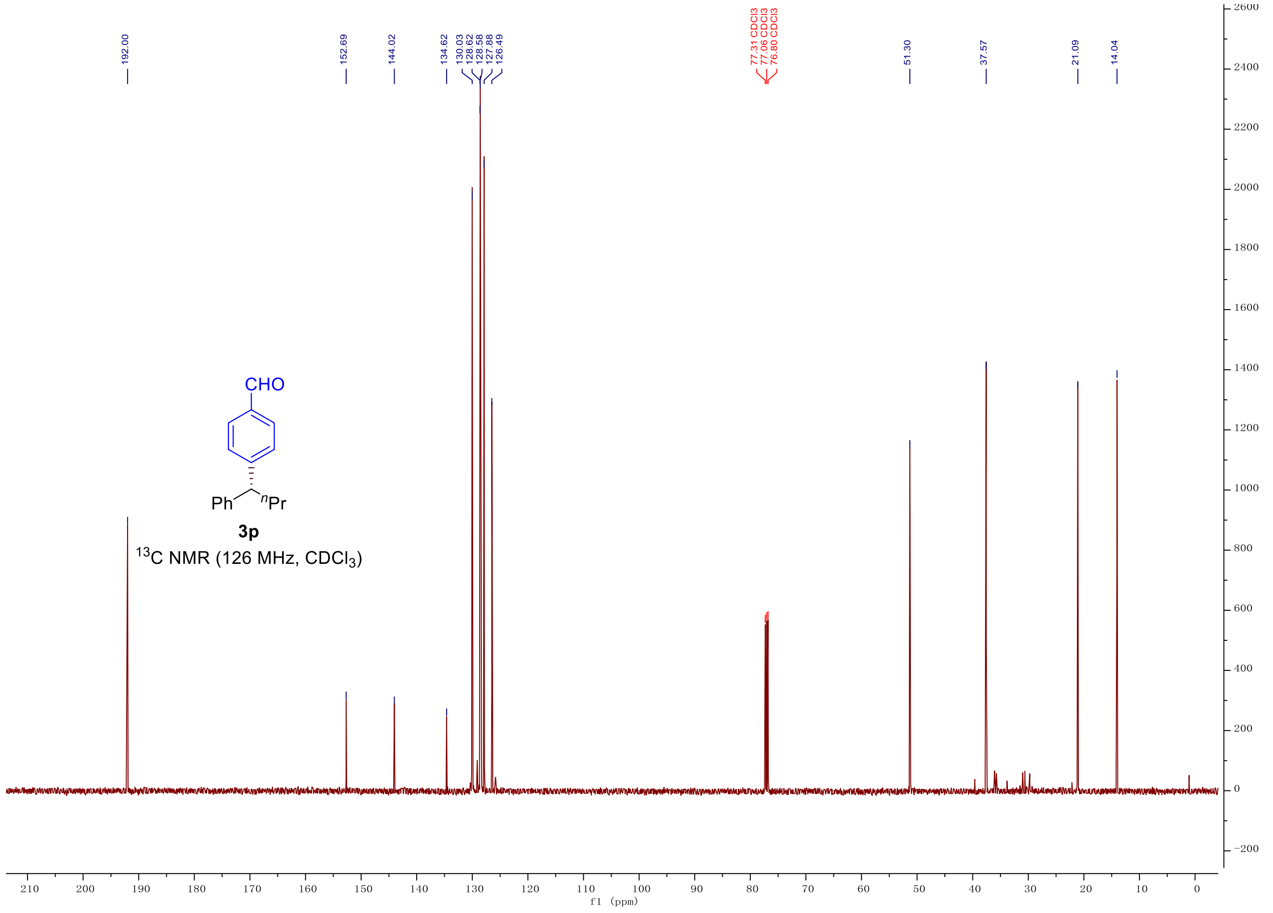

Supplementary Fig. 43. ^{13}C NMR of compound **3p**

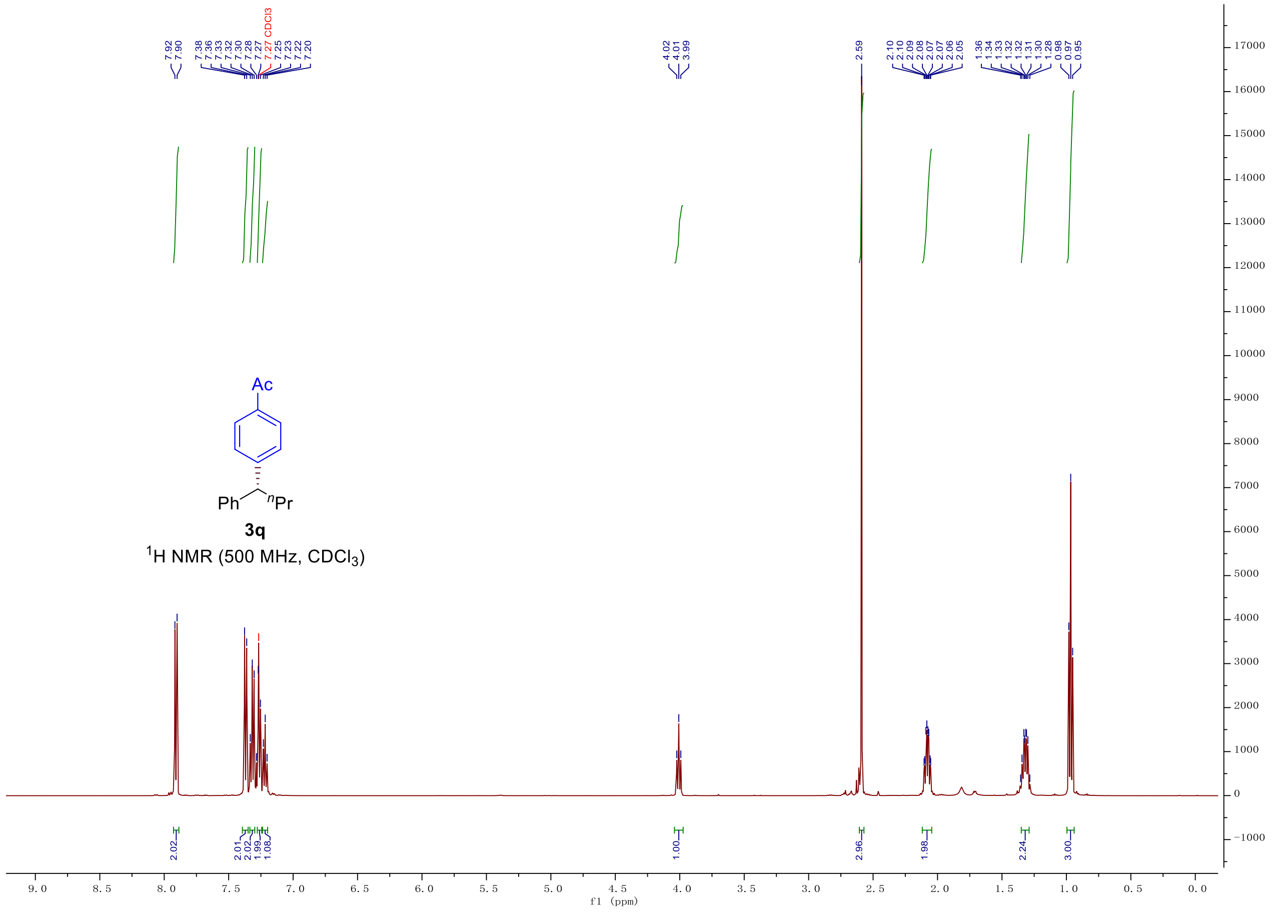

Supplementary Fig. 44. ^{1}H NMR of compound **3q**

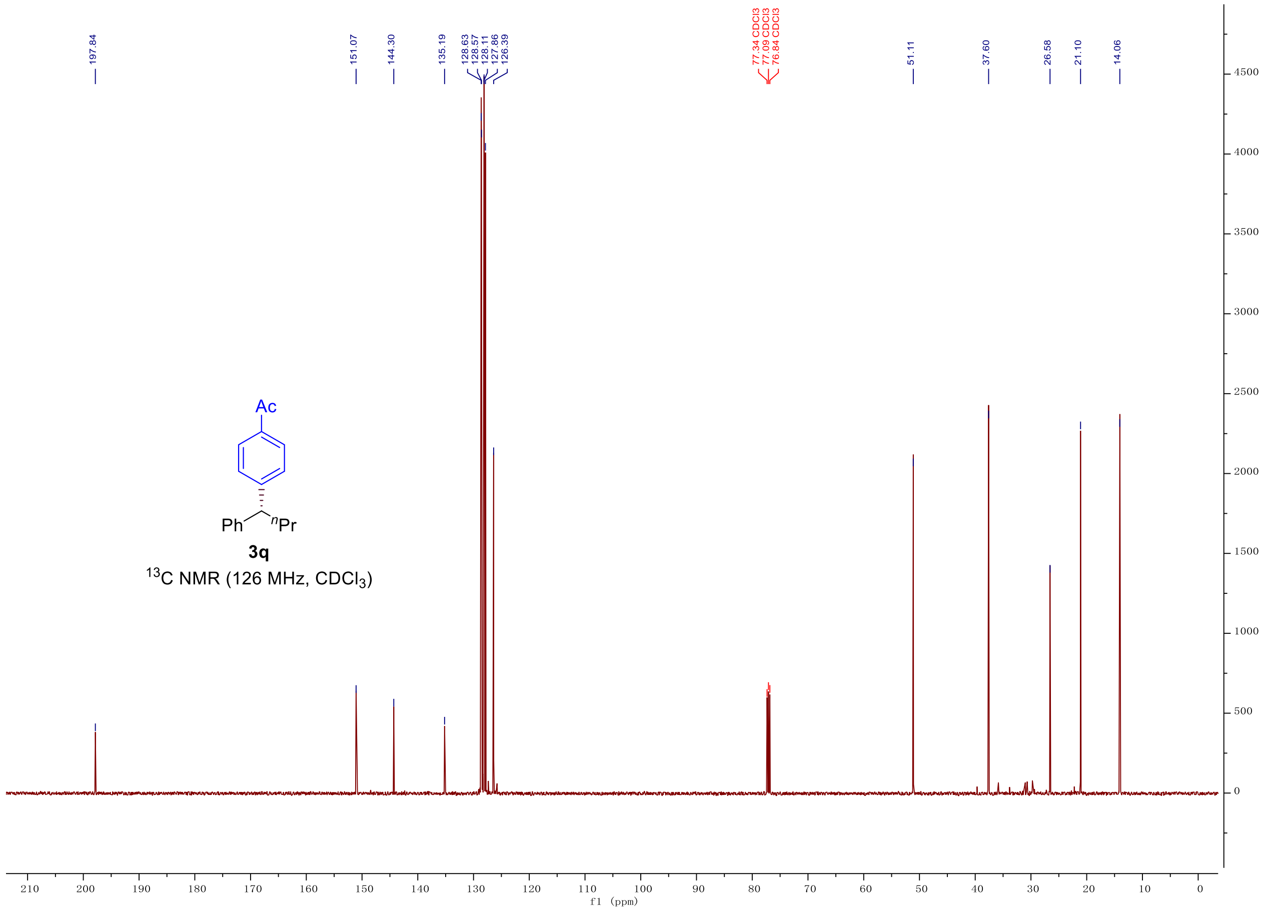

Supplementary Fig. 45. ^{13}C NMR of compound **3q**

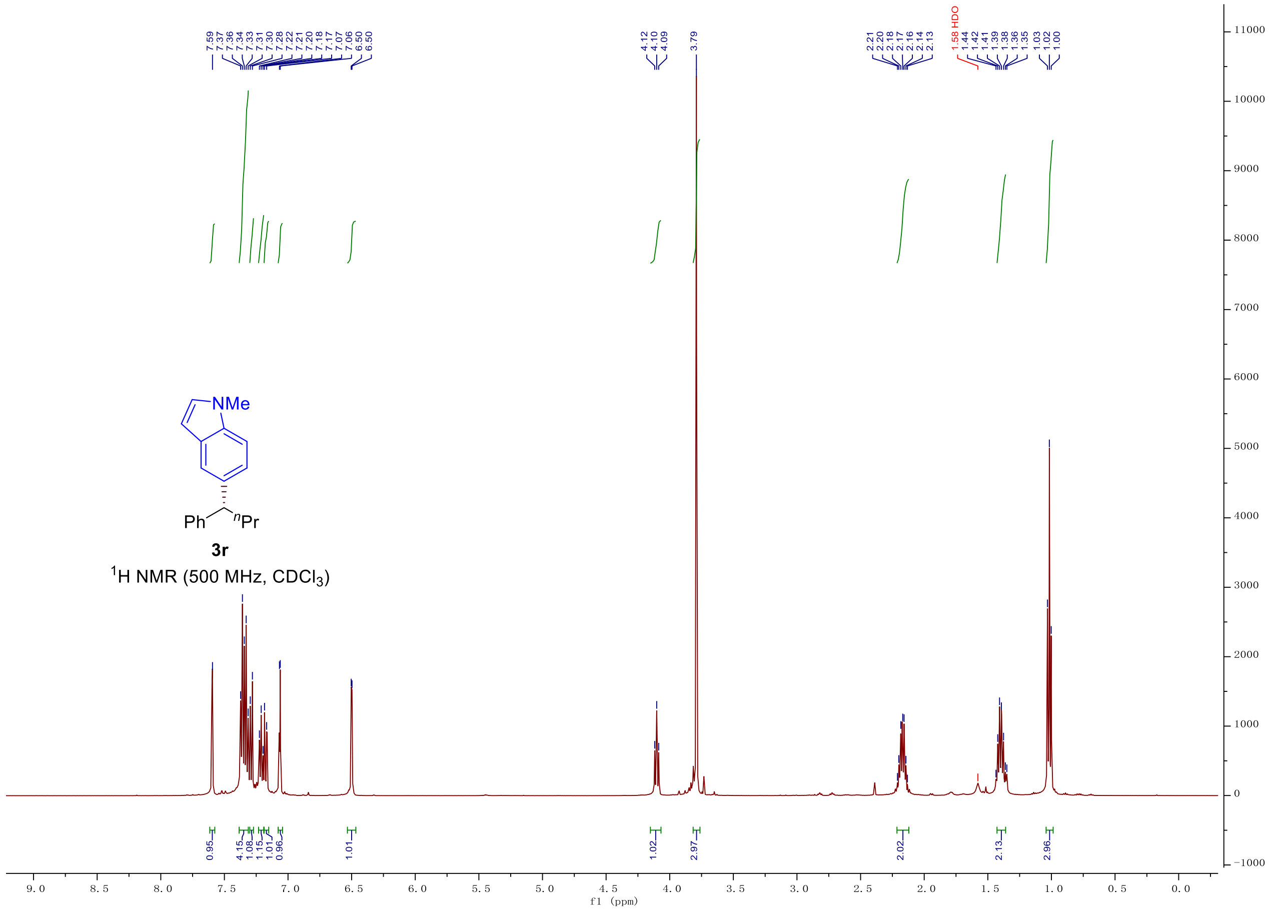

Supplementary Fig. 46. ^{1}H NMR of compound **3r**

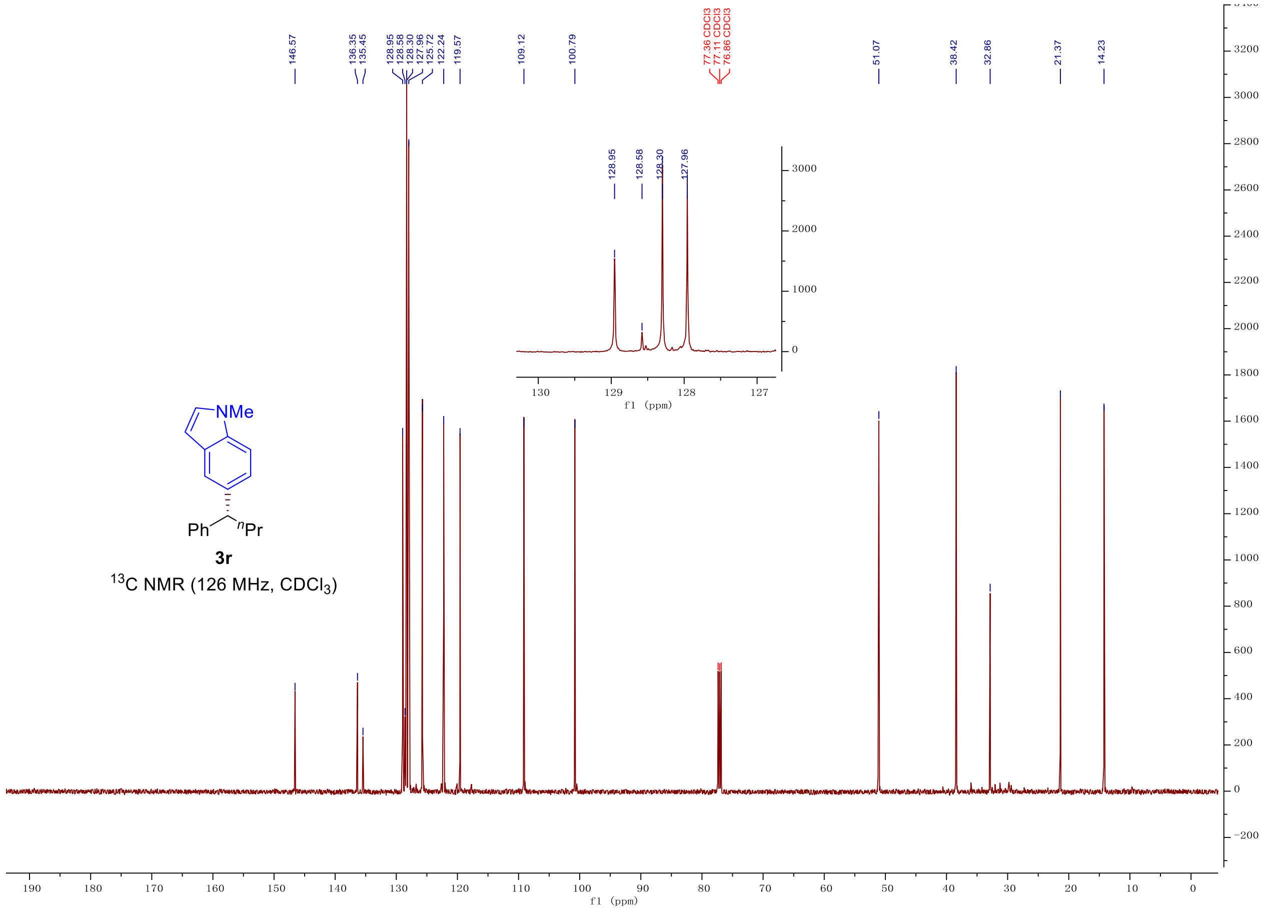

Supplementary Fig. 47. ^{13}C NMR of compound **3r**

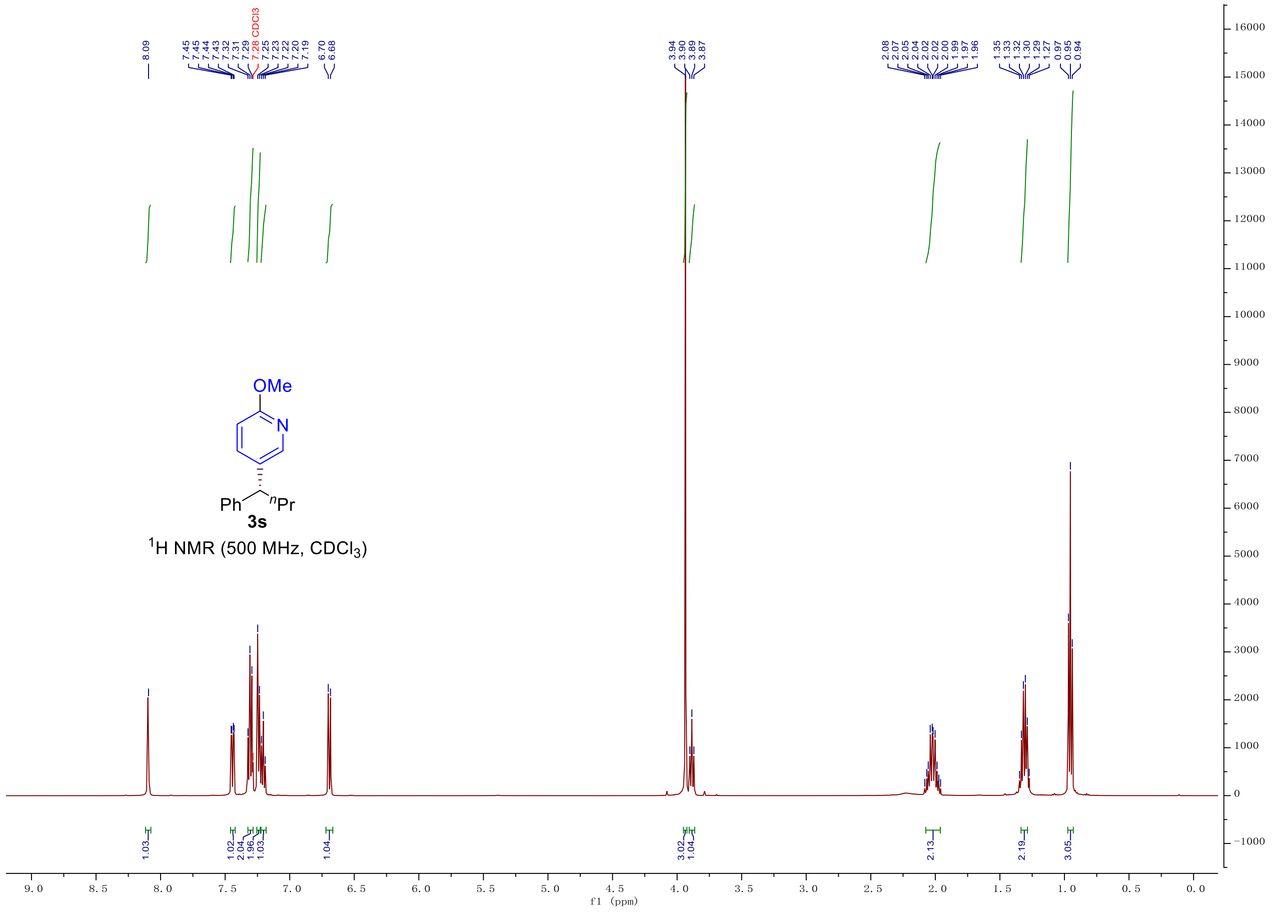

Supplementary Fig. 48. ^{1}H NMR of compound **3s**

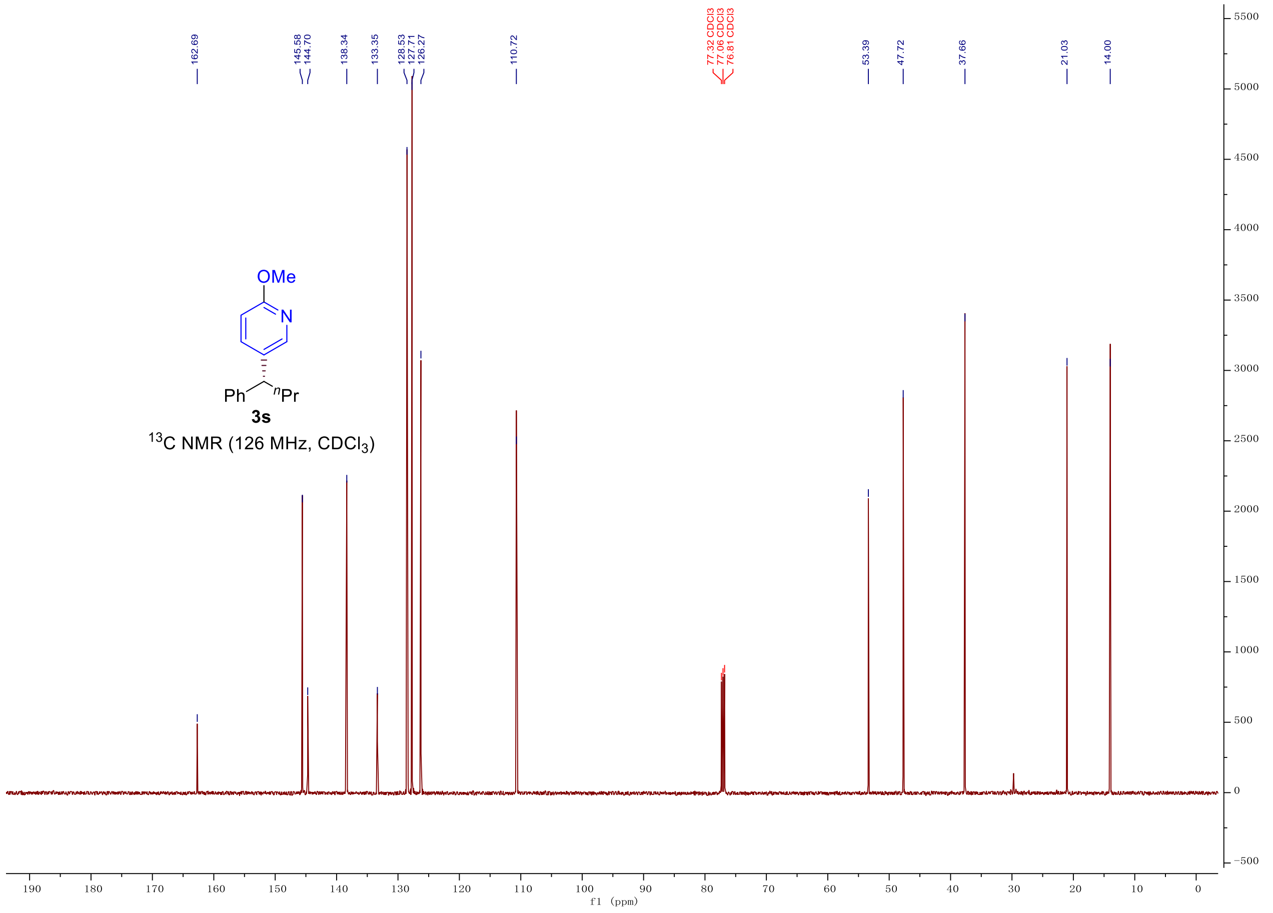

Supplementary Fig. 49. ^{13}C NMR of compound **3s**

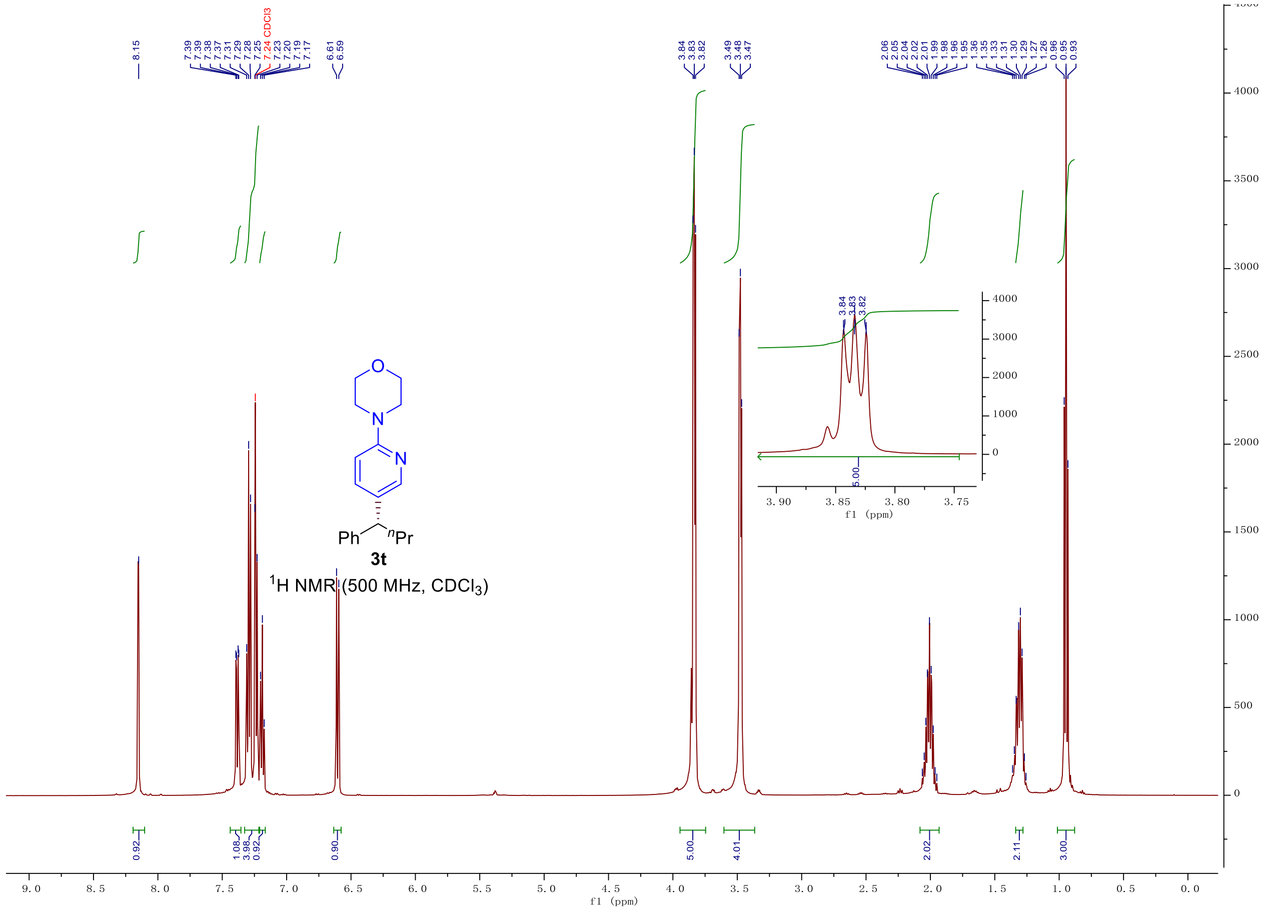

Supplementary Fig. 50. ^{1}H NMR of compound **3t**

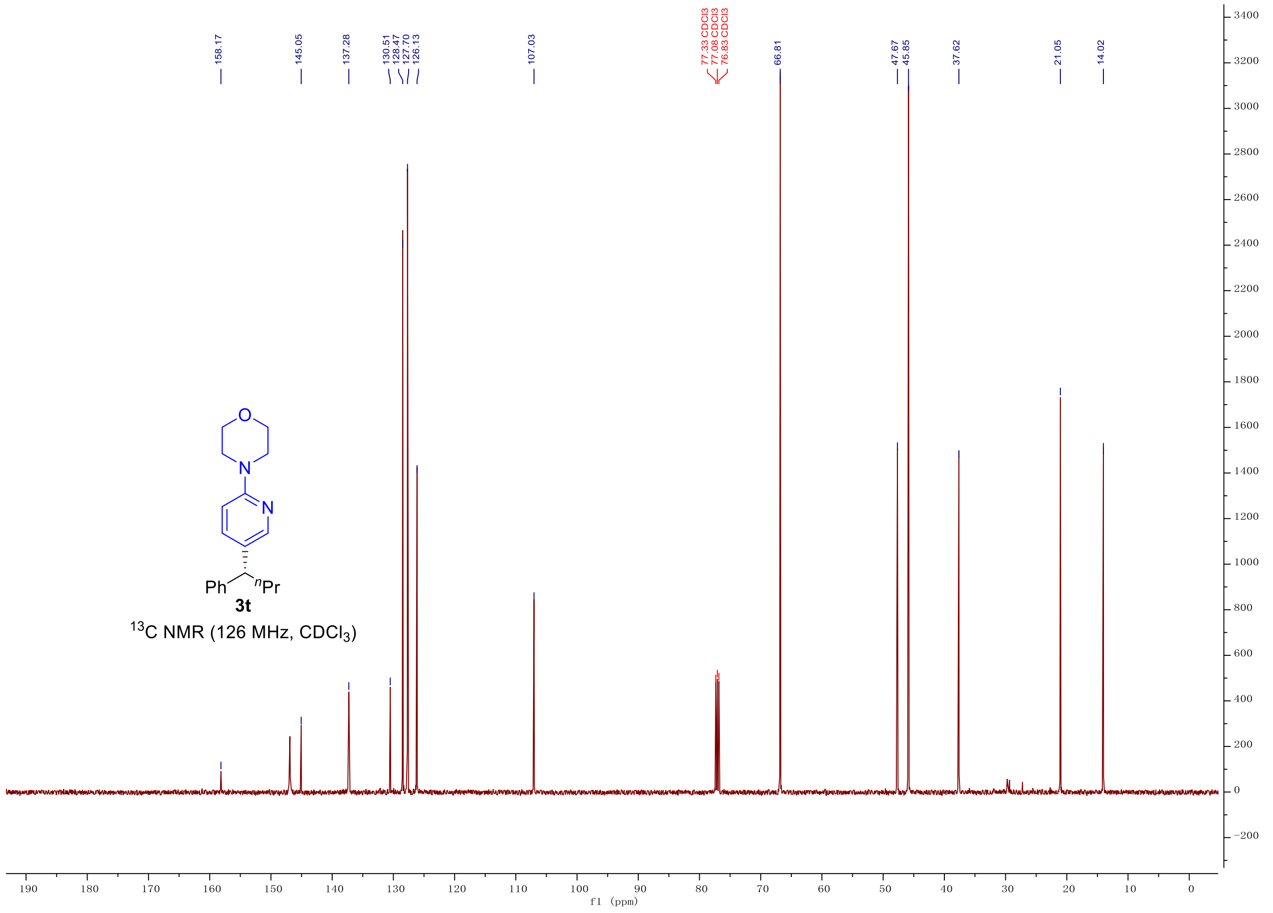

Supplementary Fig. 51. ^{13}C NMR of compound **3t**

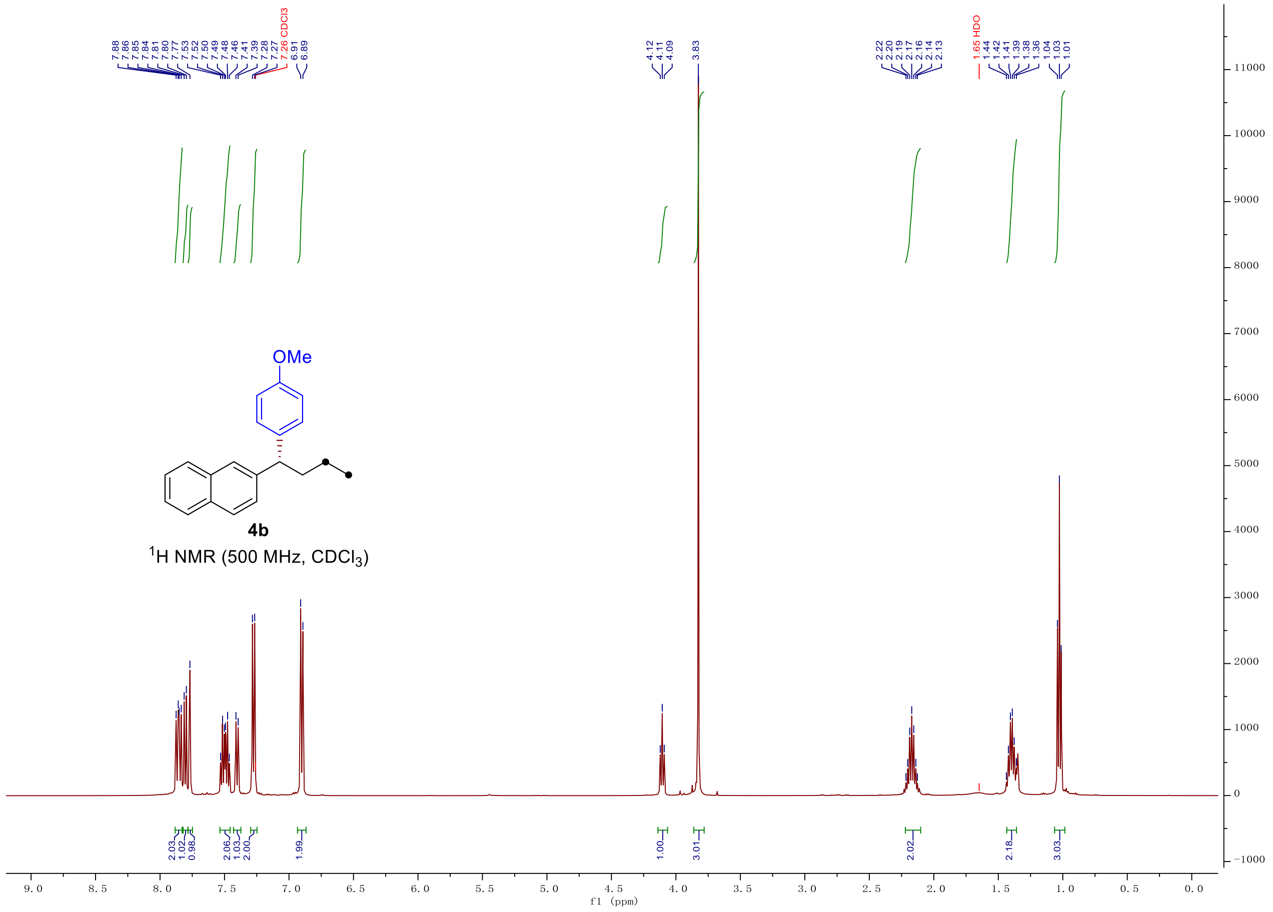

Supplementary Fig. 52. ^{1}H NMR of compound **4b**

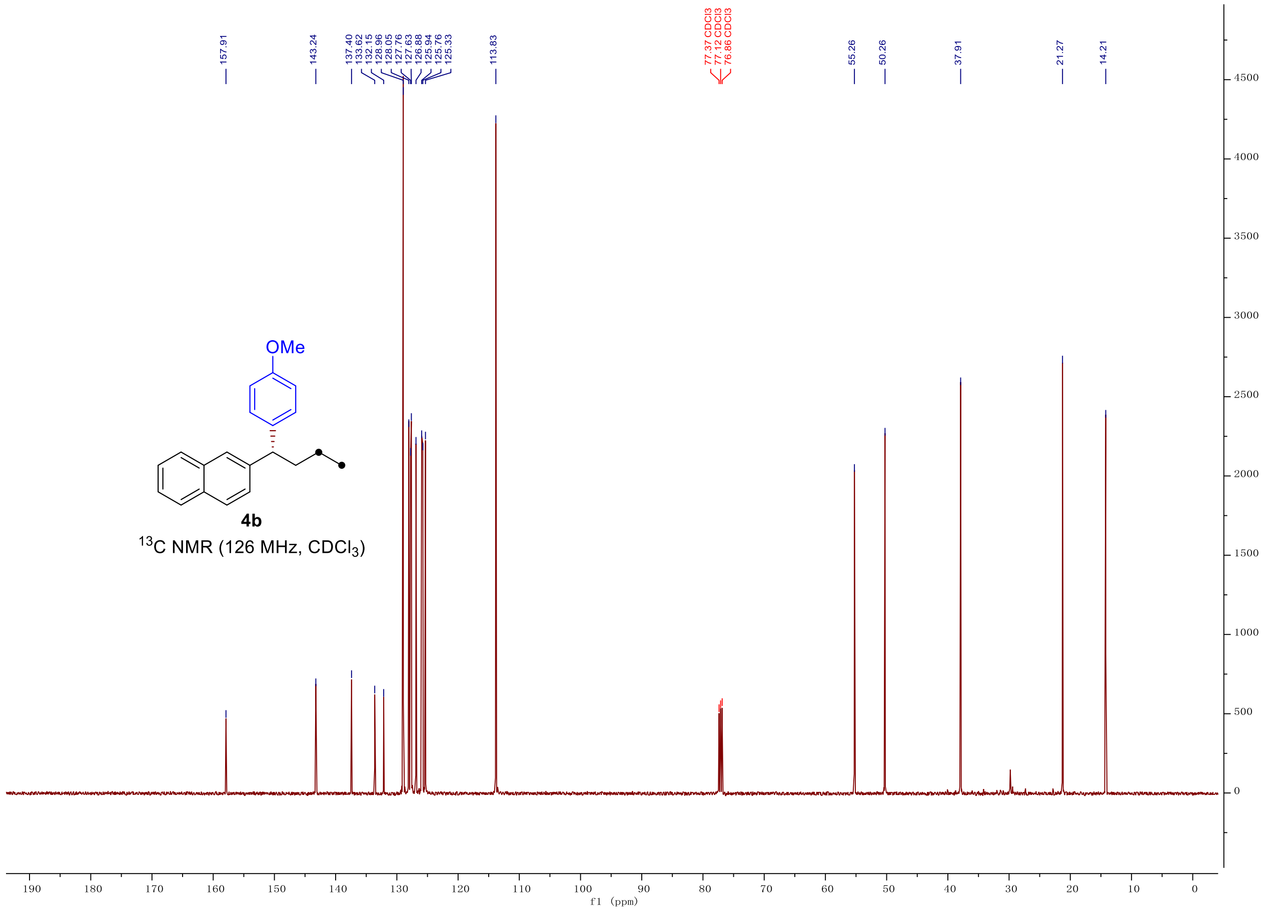

Supplementary Fig. 53. ^{13}C NMR of compound **4b**

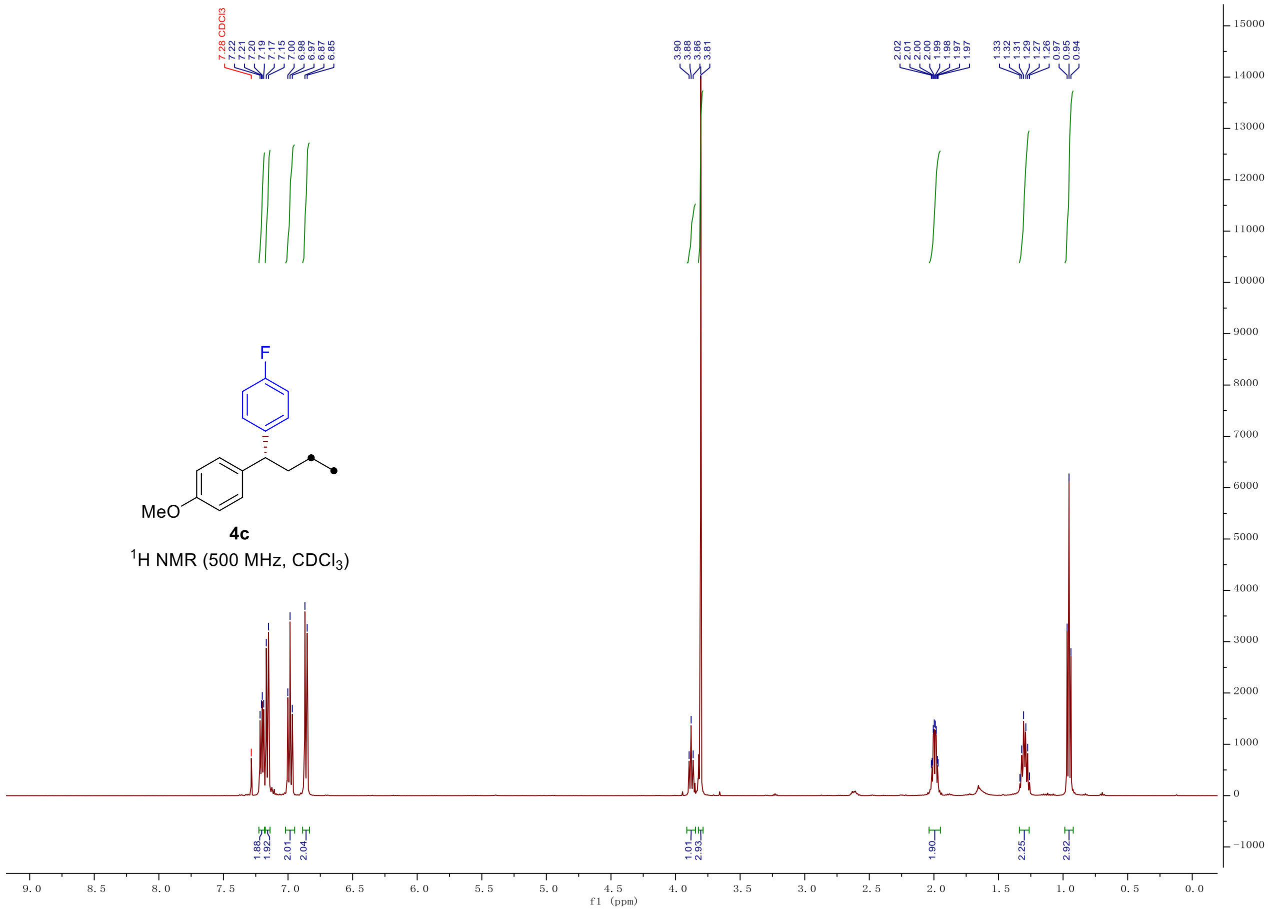

Supplementary Fig. 54. ^{1}H NMR of compound **4c**

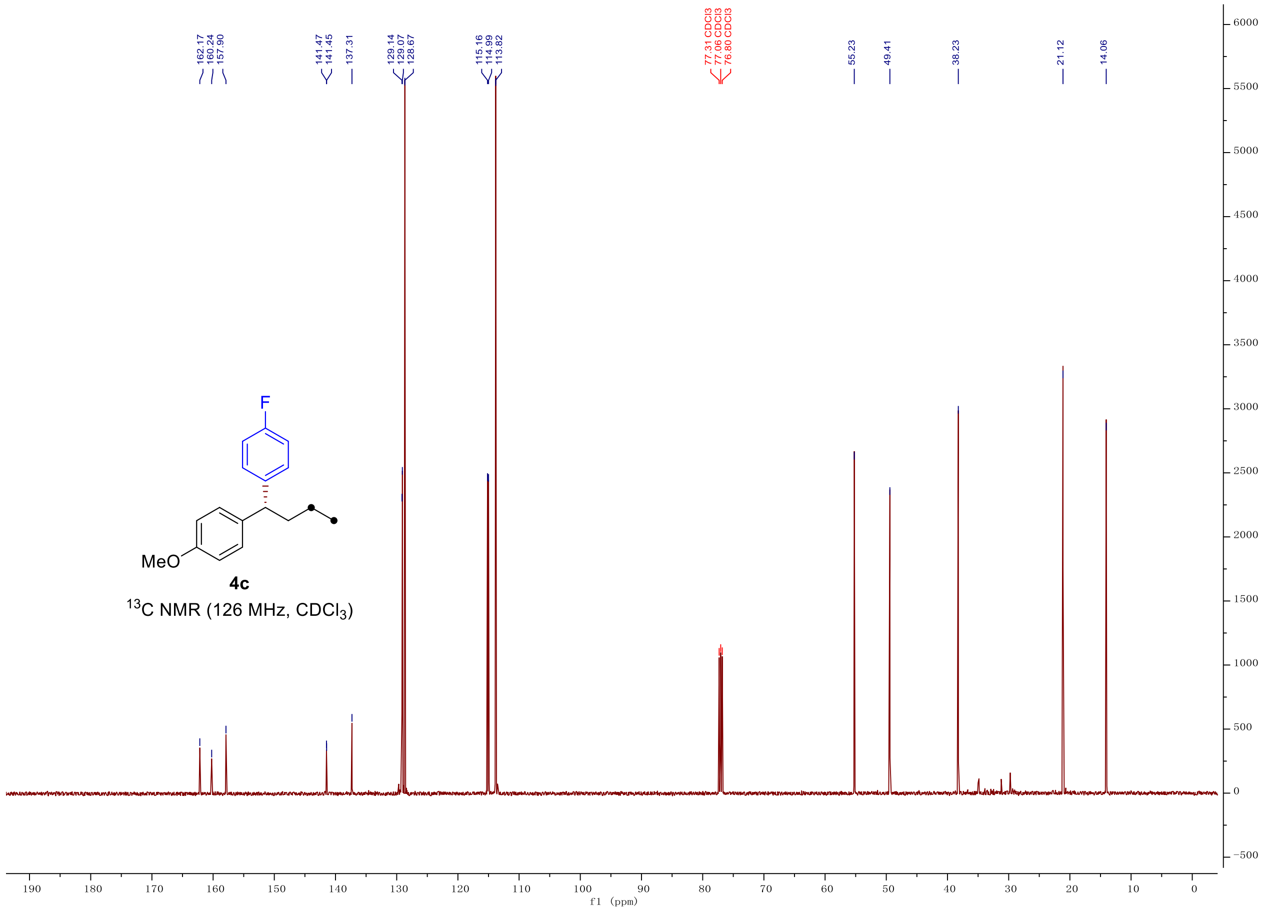

Supplementary Fig. 55. ^{13}C NMR of compound **4c**

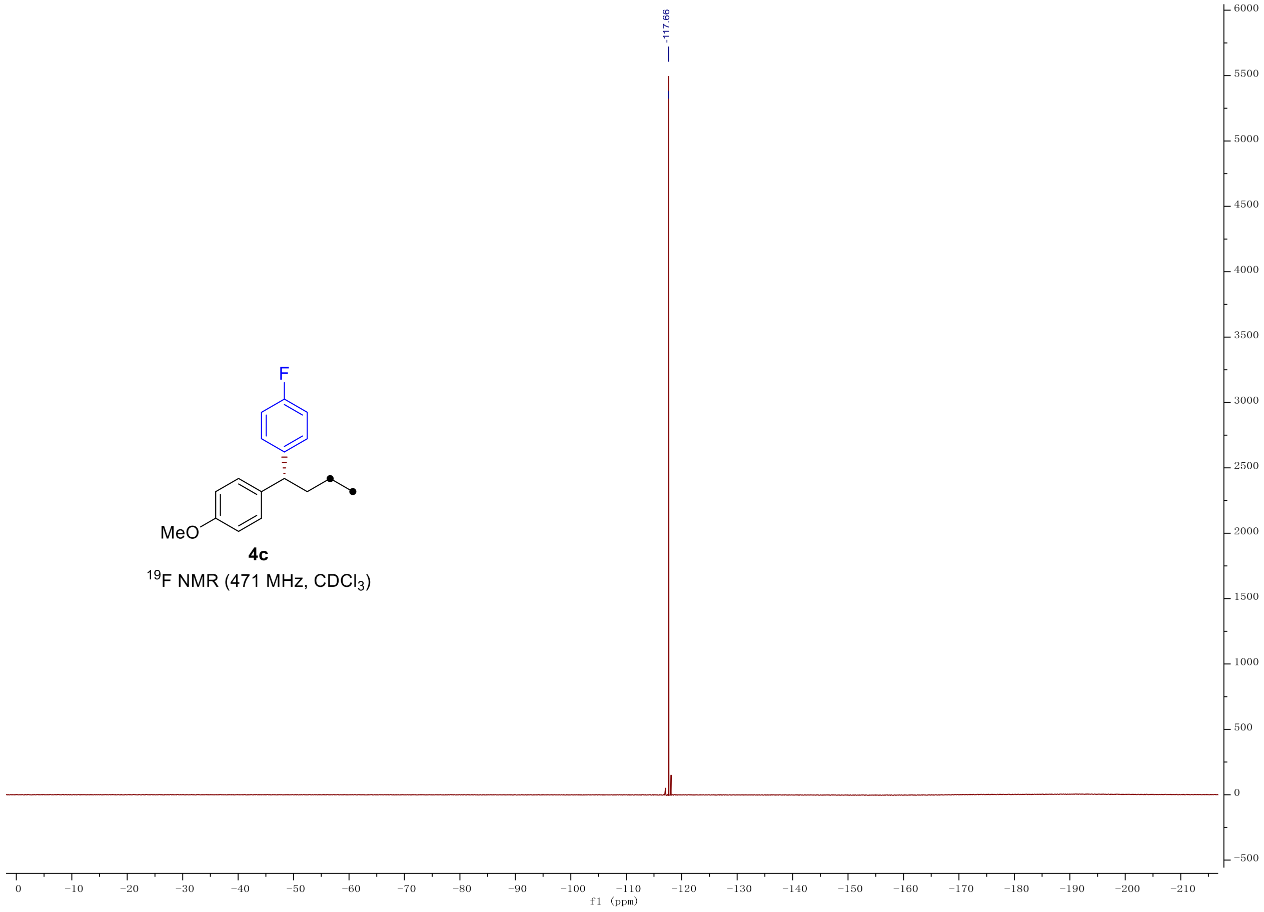

Supplementary Fig. 56. ^{19}F NMR of compound **4c**

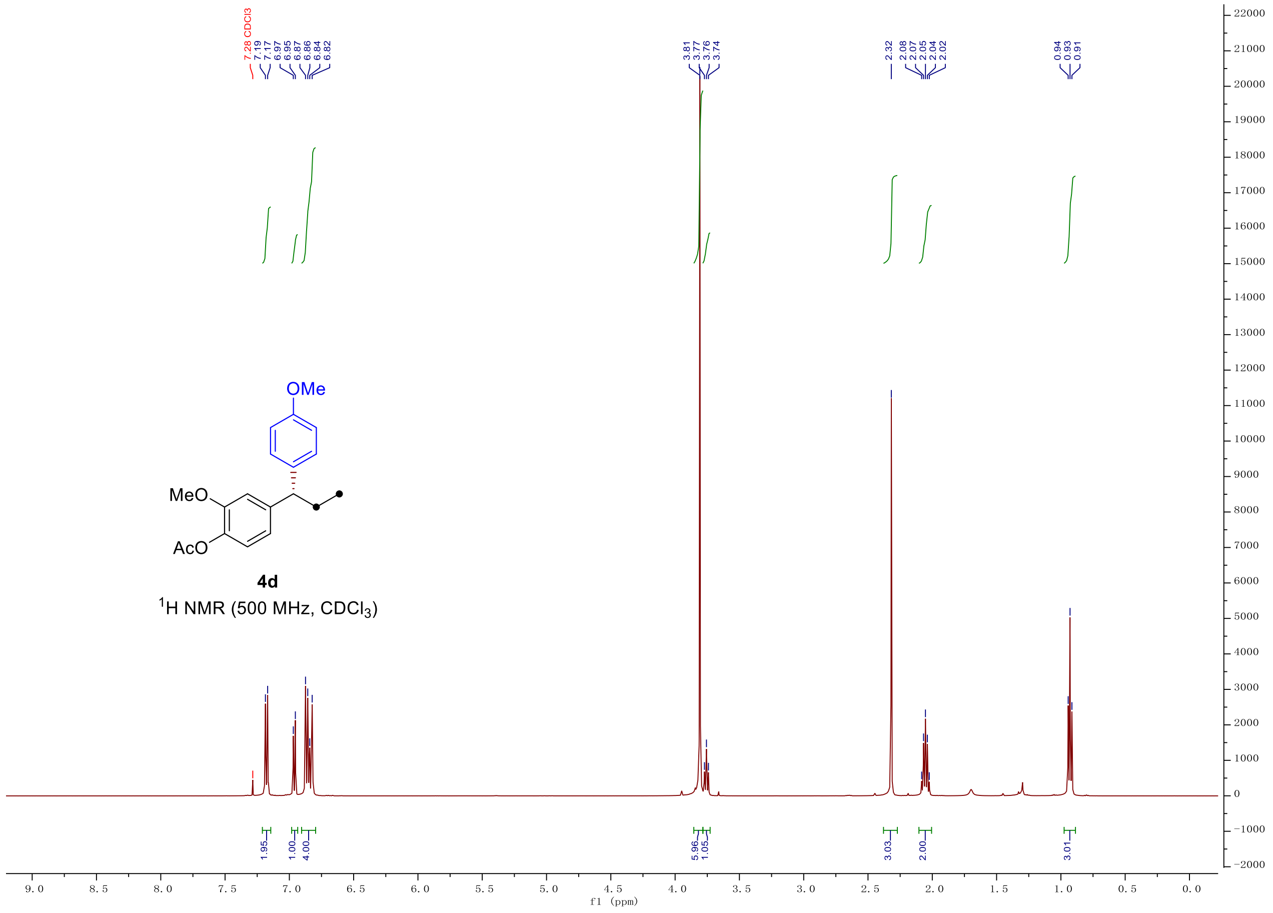

Supplementary Fig. 57. ^{1}H NMR of compound **4d**

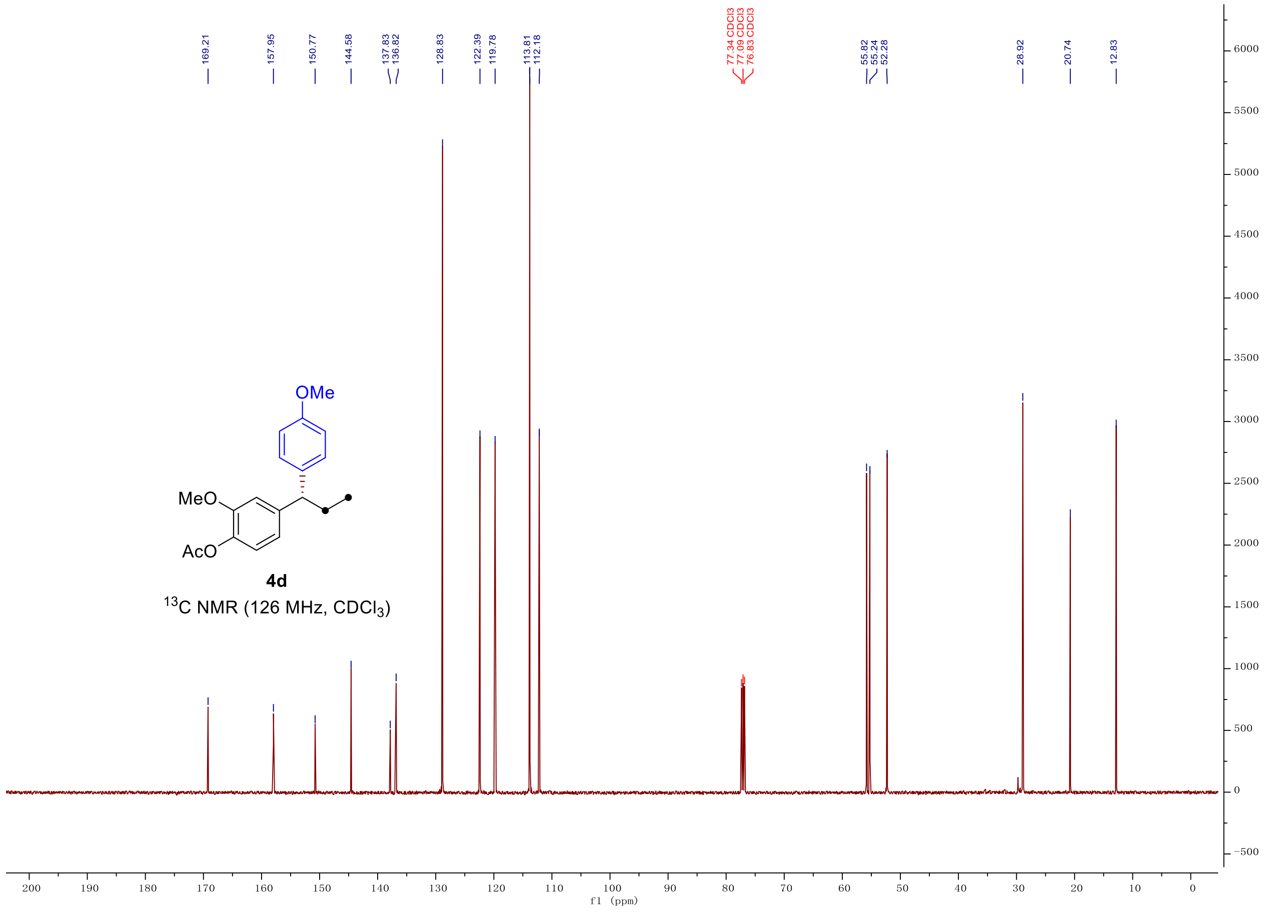

Supplementary Fig. 58. ^{13}C NMR of compound **4d**

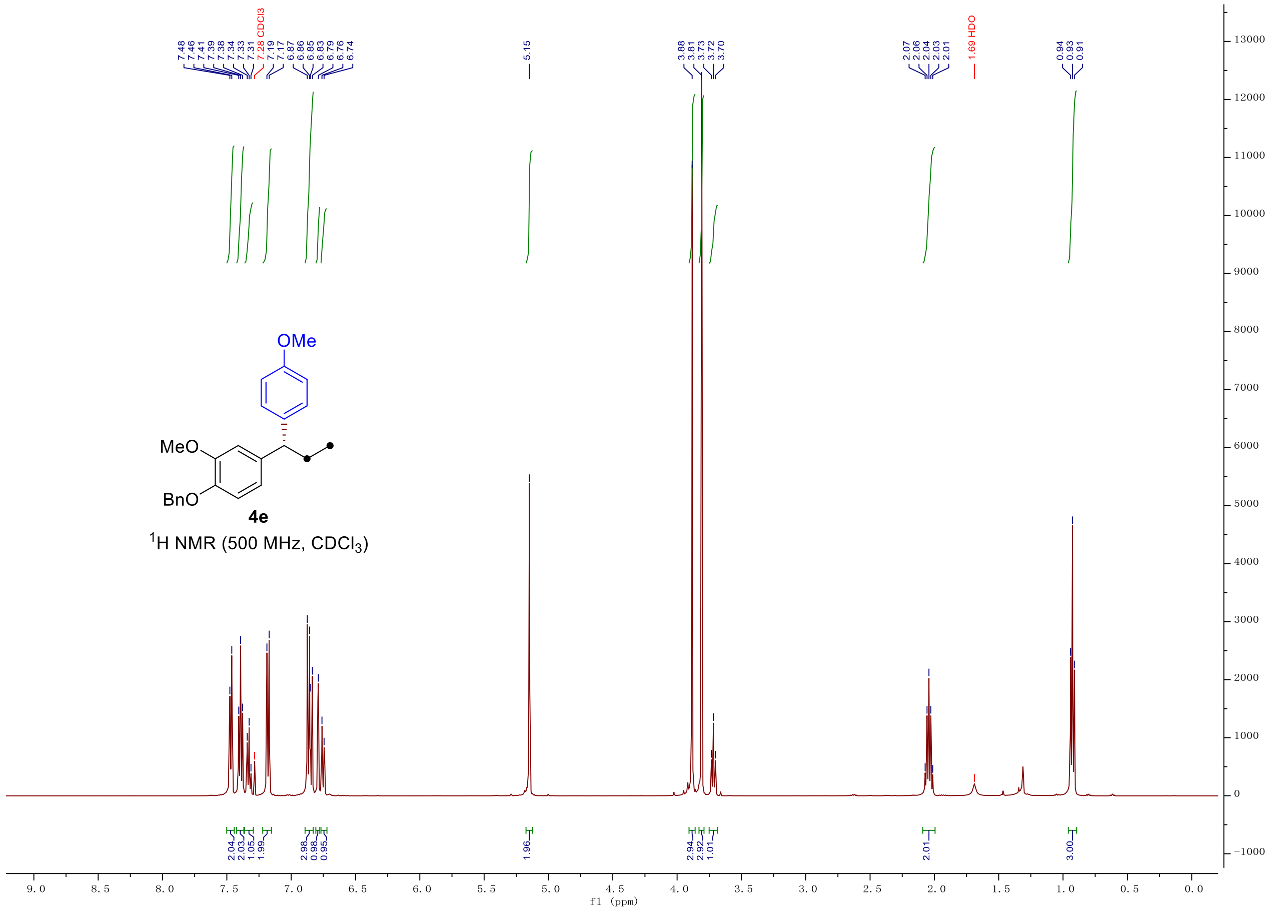

Supplementary Fig. 59. ^{1}H NMR of compound **4e**

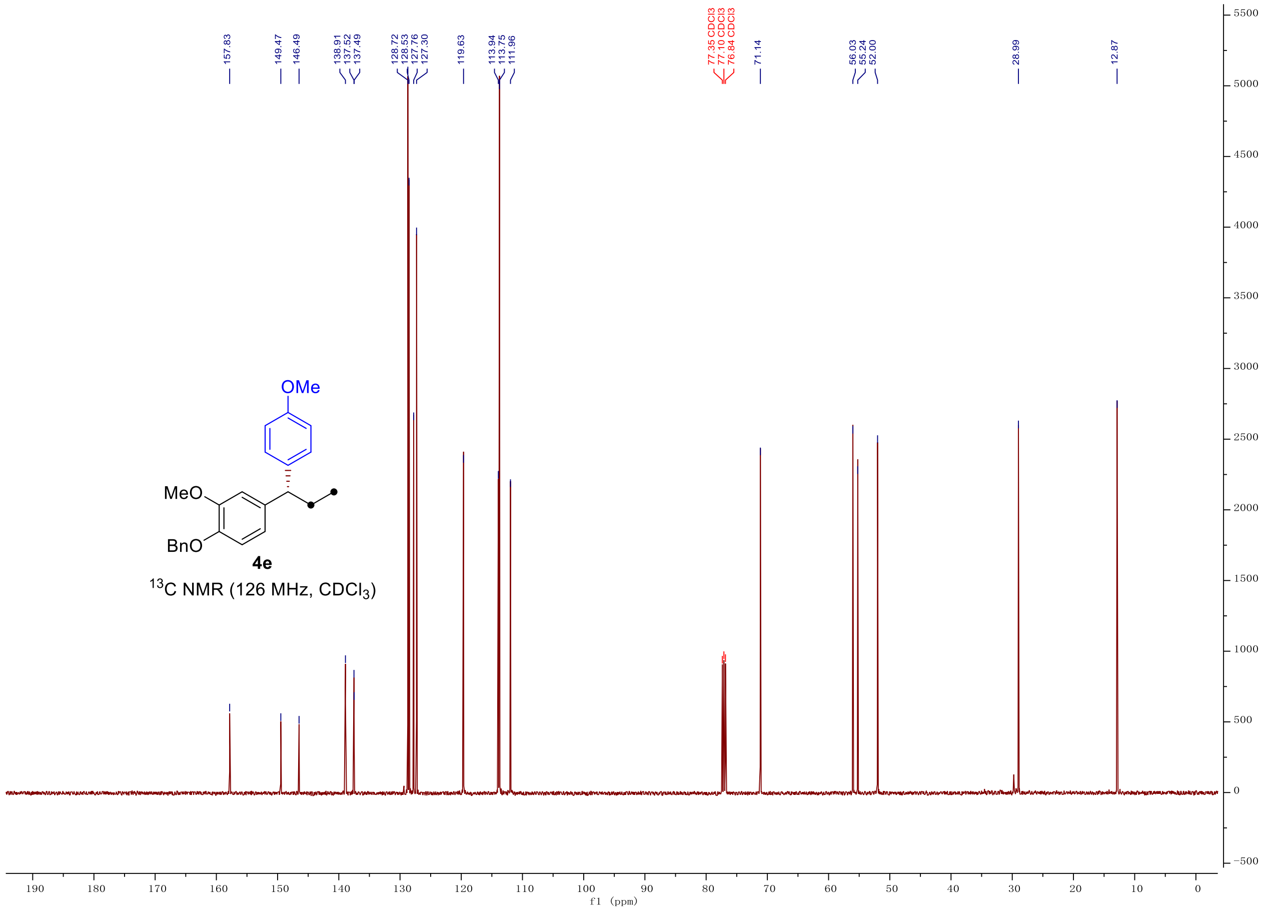

Supplementary Fig. 60. ^{13}C NMR of compound **4e**

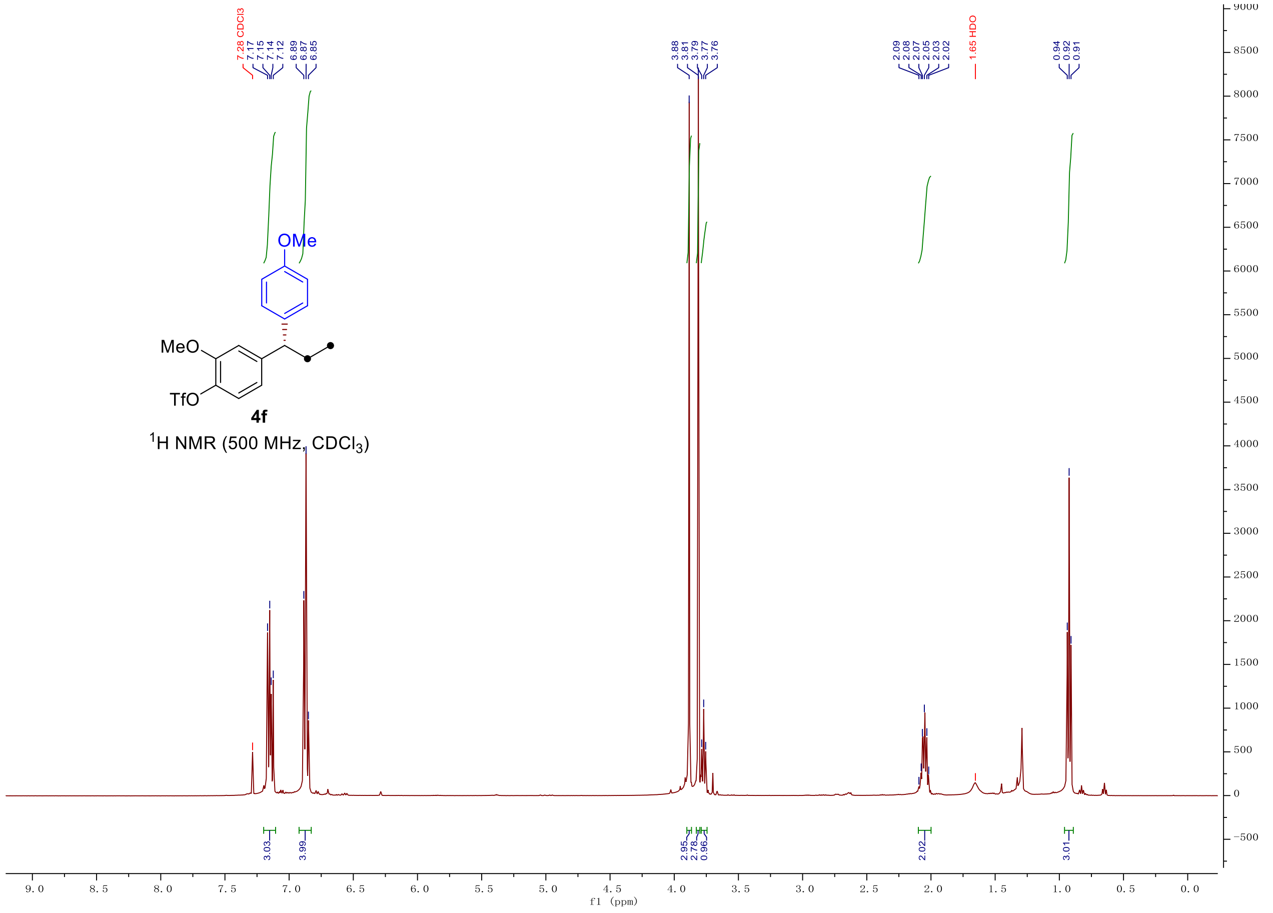

Supplementary Fig. 61. ^{1}H NMR of compound **4f**

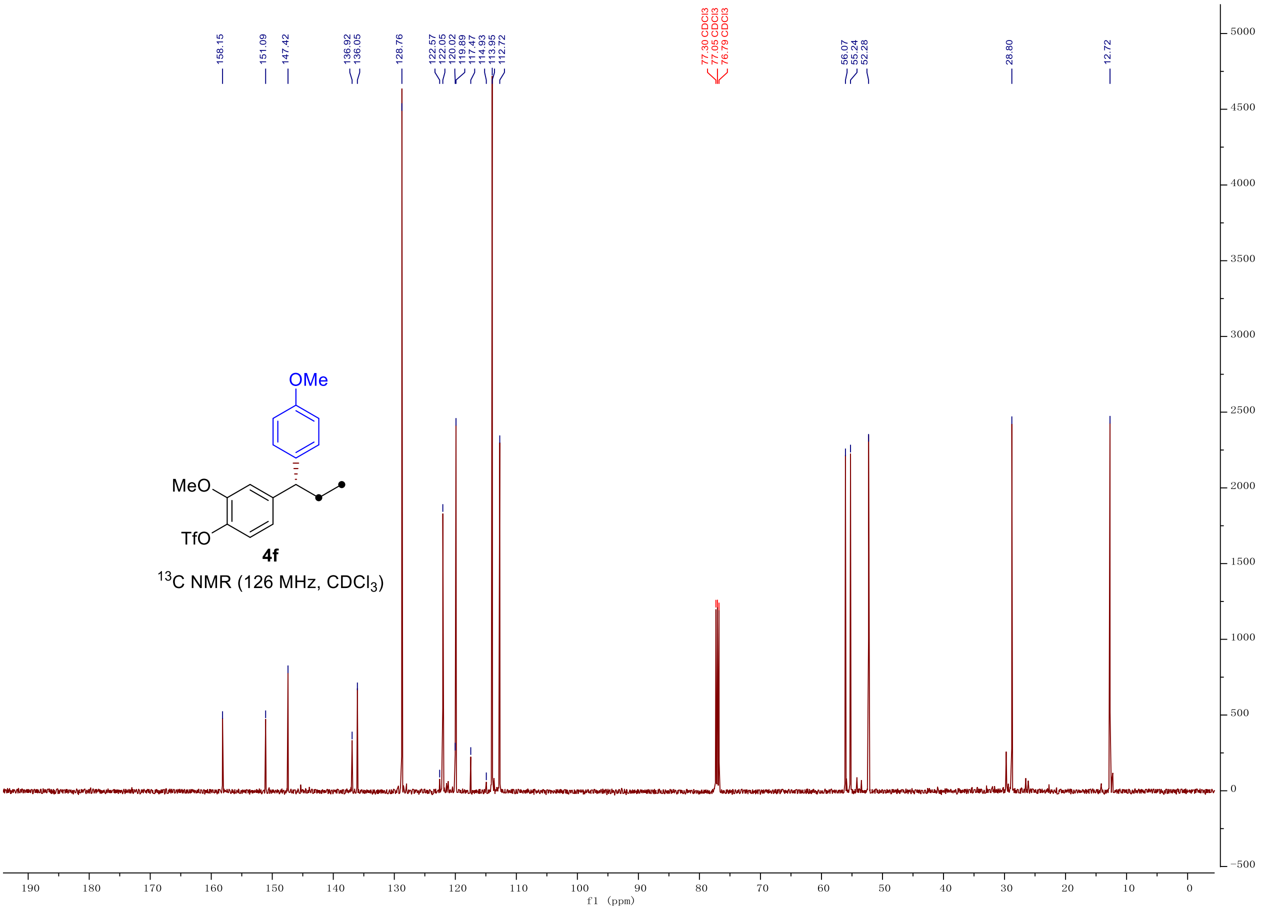

Supplementary Fig. 62. ^{13}C NMR of compound **4f**

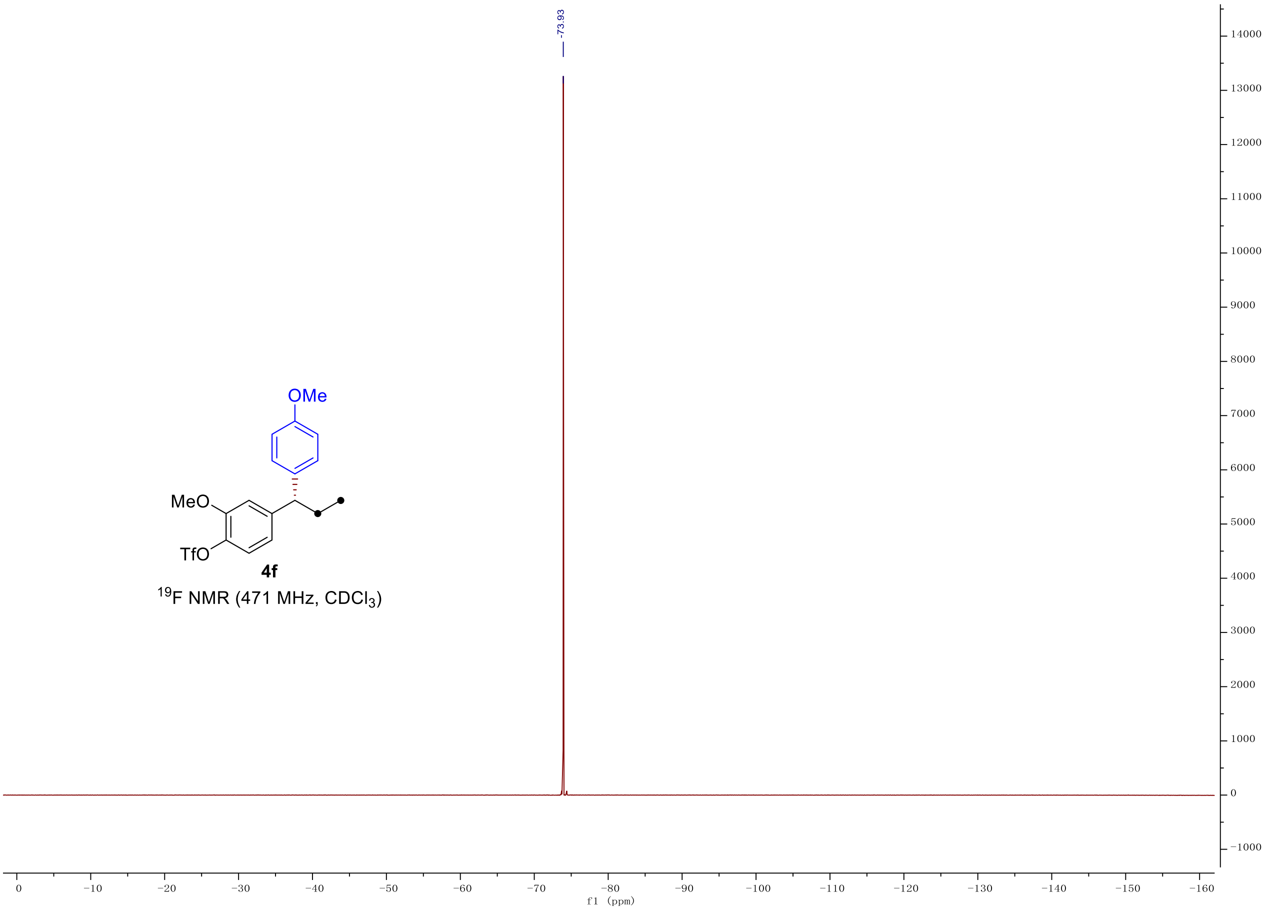

Supplementary Fig. 63. ^{19}F NMR of compound **4f**

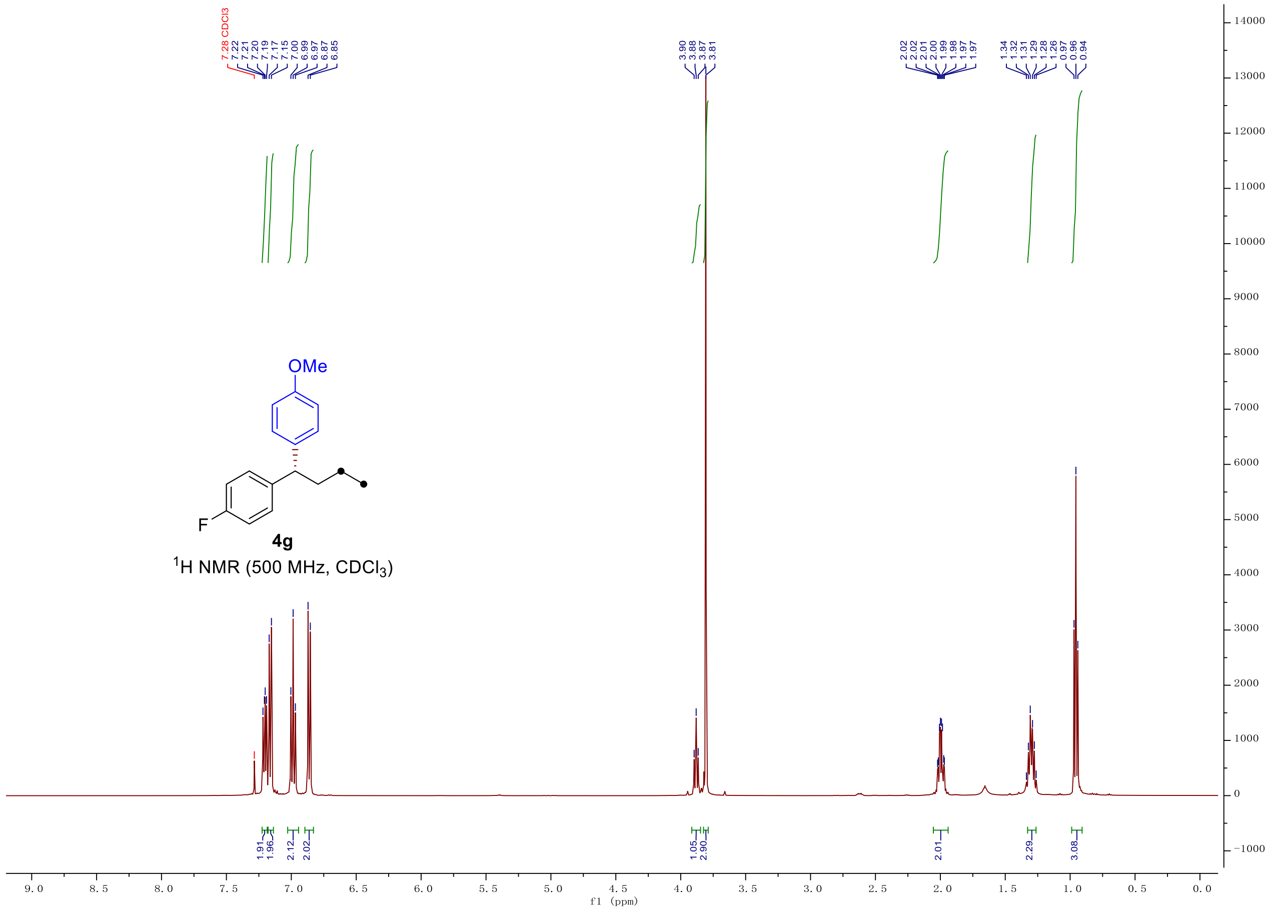

Supplementary Fig. 64. ^{1}H NMR of compound **4g**

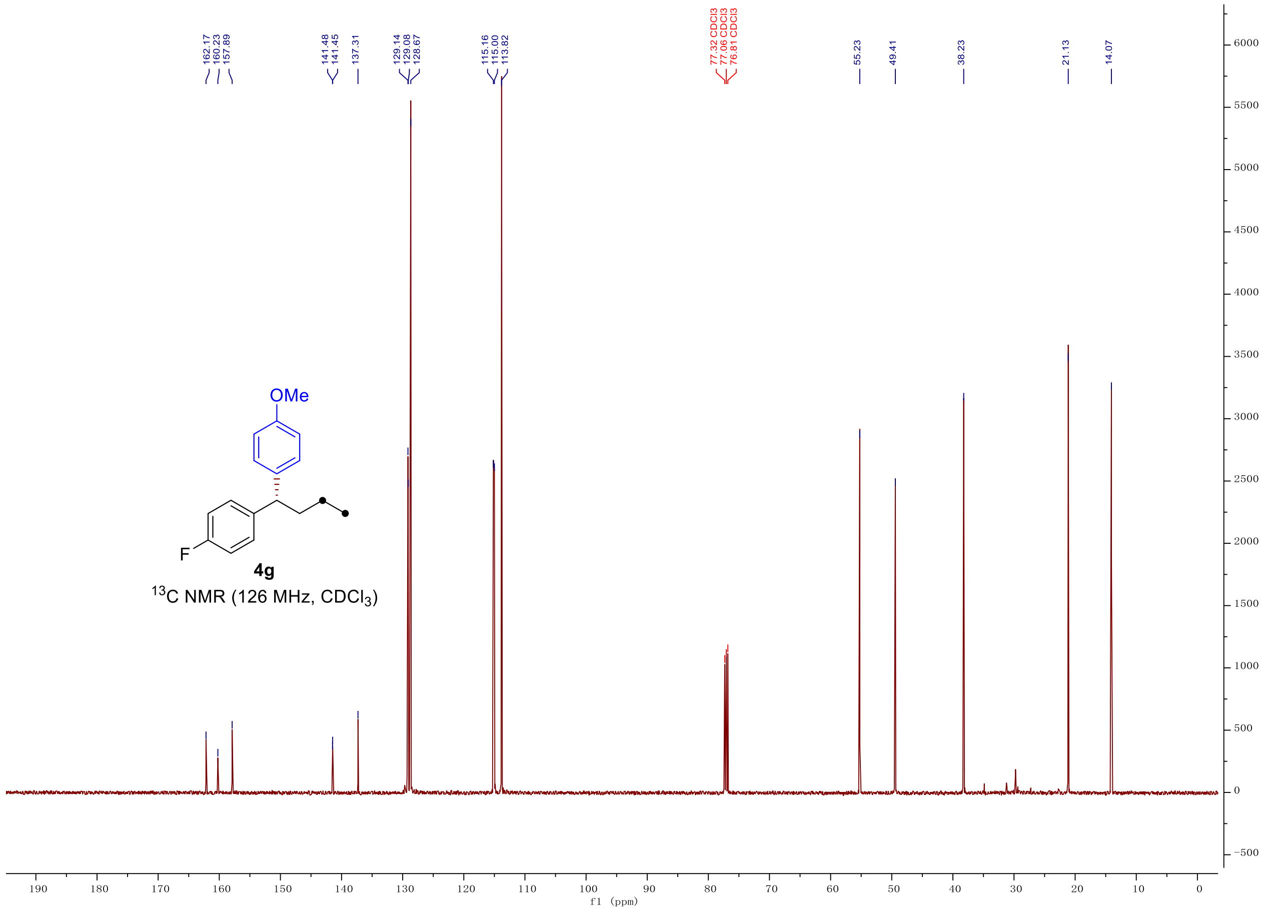

Supplementary Fig. 65. ^{13}C NMR of compound **4g**

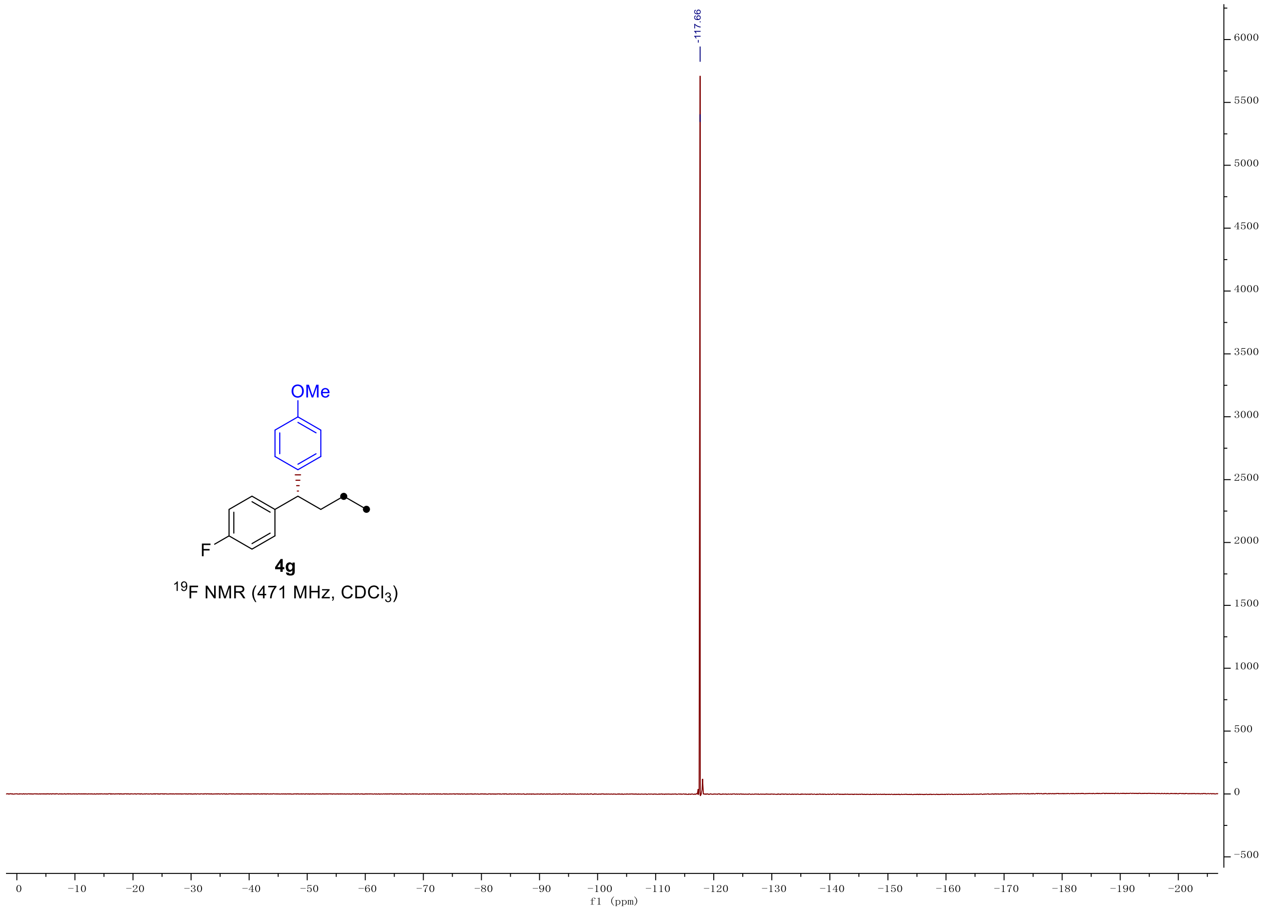

Supplementary Fig. 66. ^{19}F NMR of compound **4g**

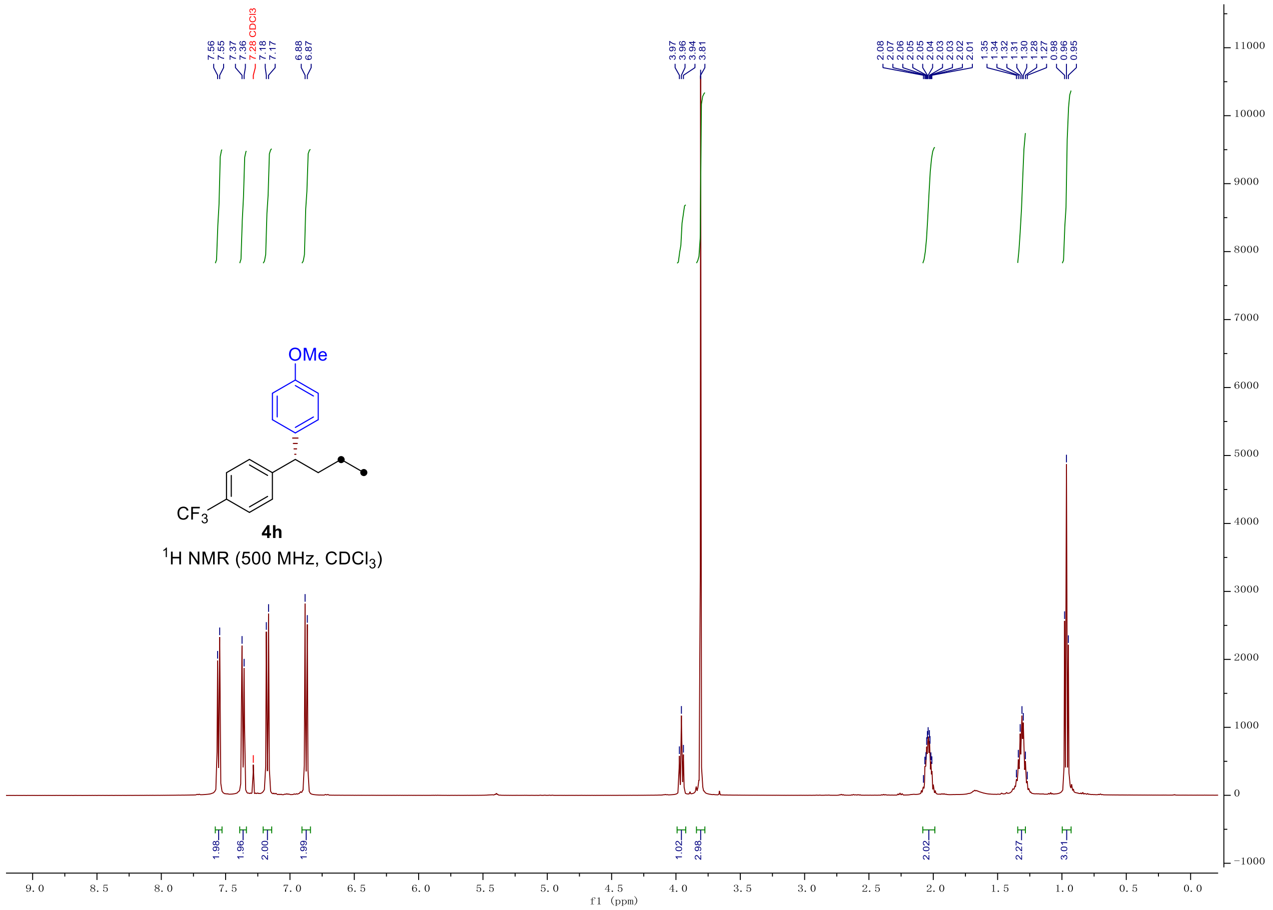

Supplementary Fig. 67. ^{1}H NMR of compound **4h**

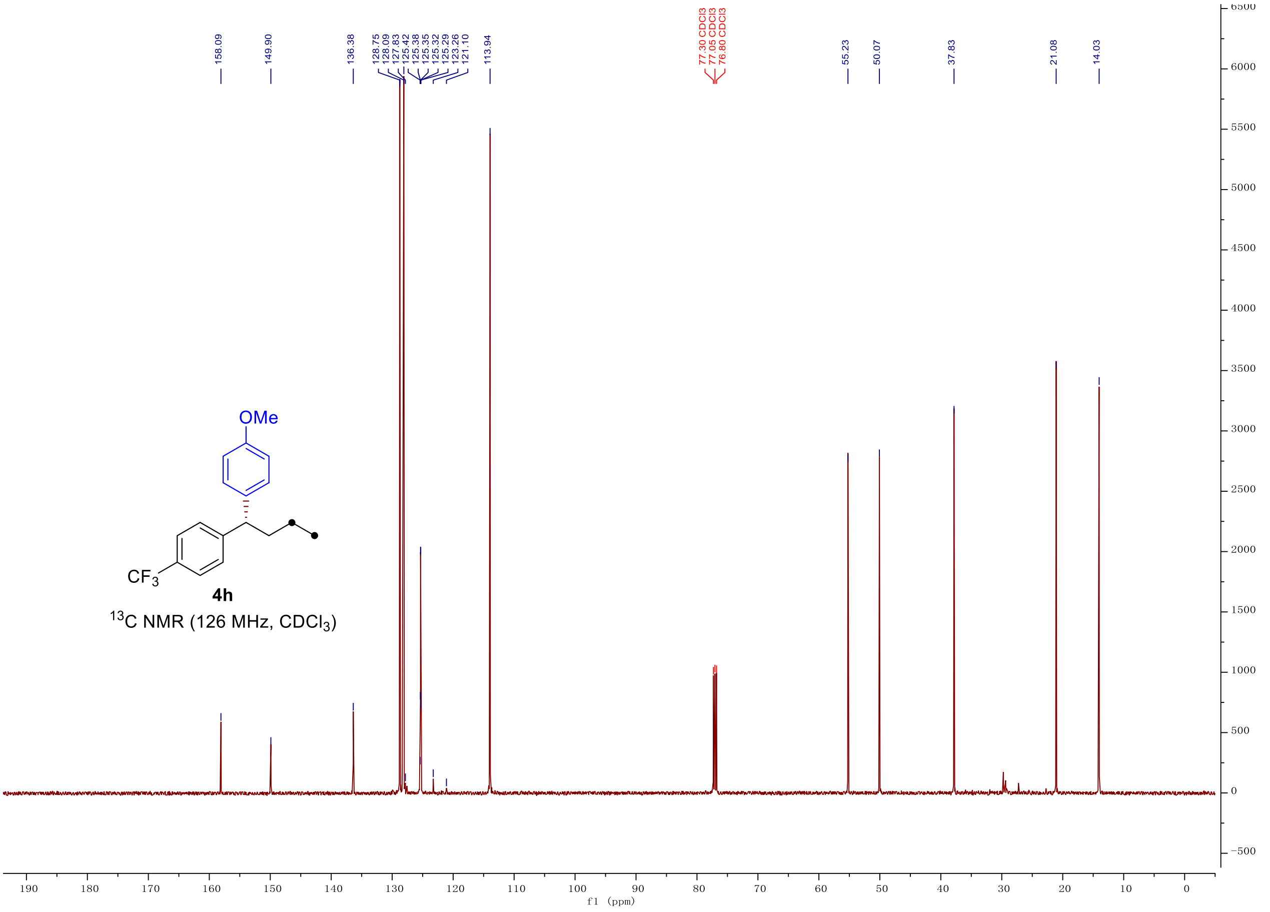

Supplementary Fig. 68. ^{13}C NMR of compound **4h**

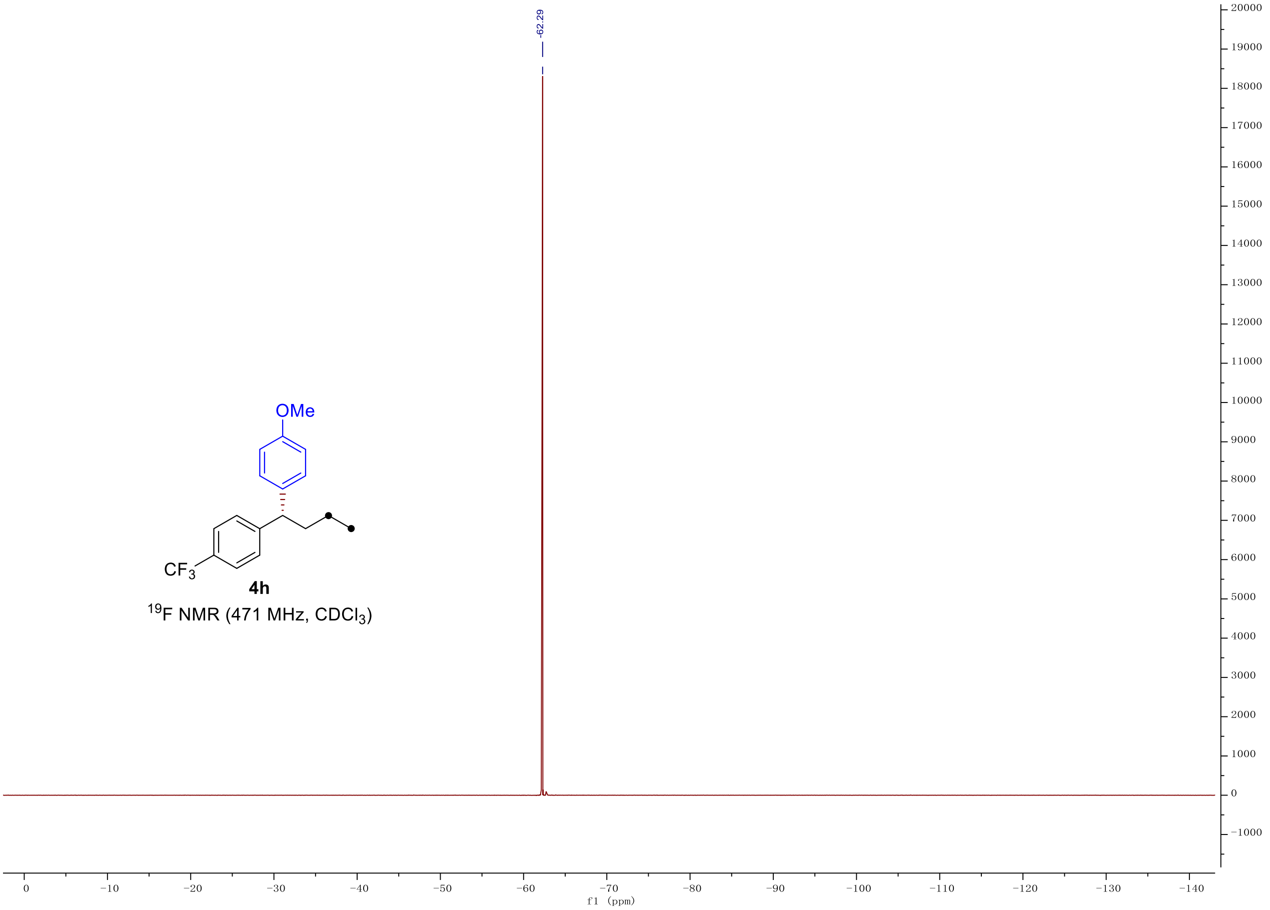

Supplementary Fig. 69. ^{19}F NMR of compound **4h**

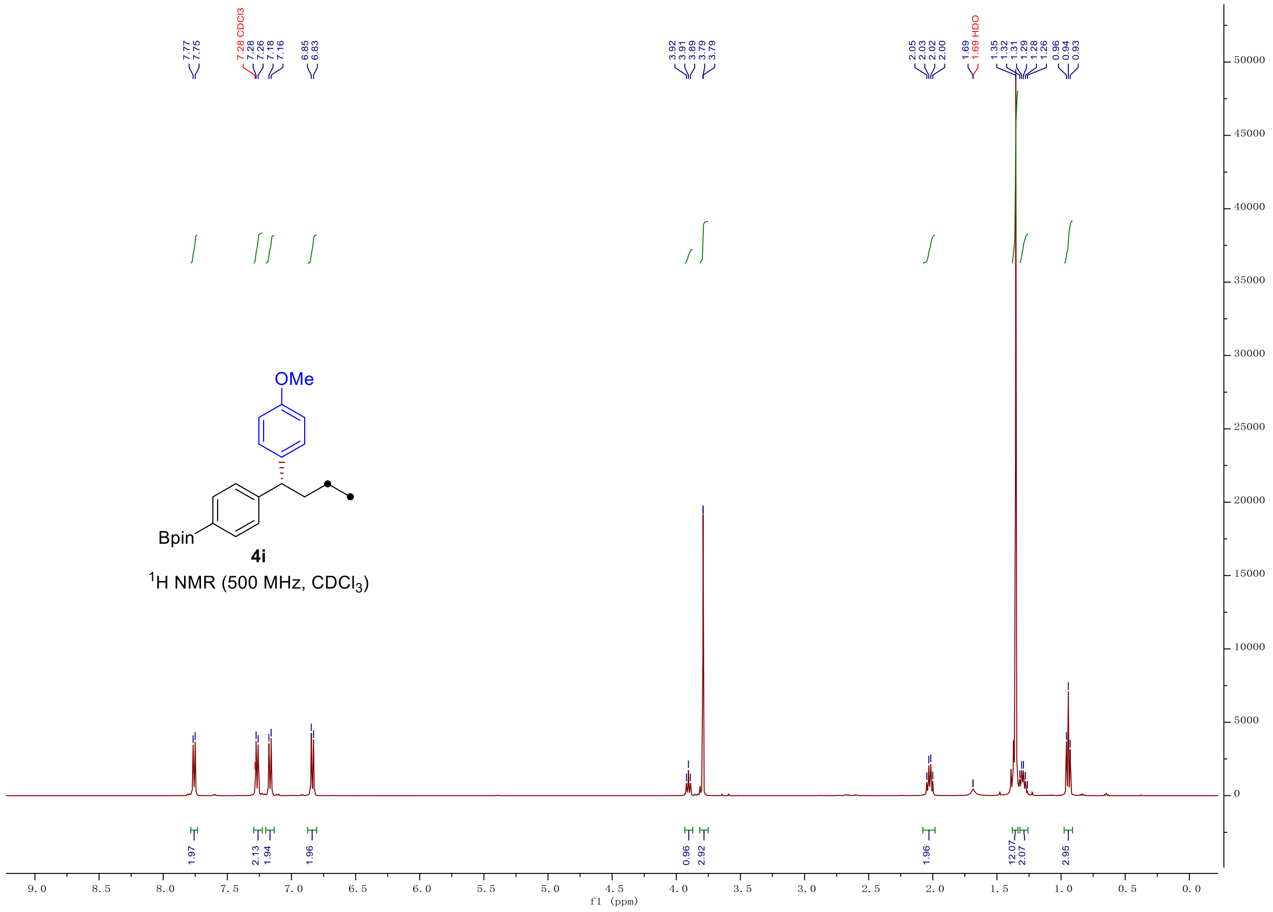

Supplementary Fig. 70. ^{1}H NMR of compound **4i**

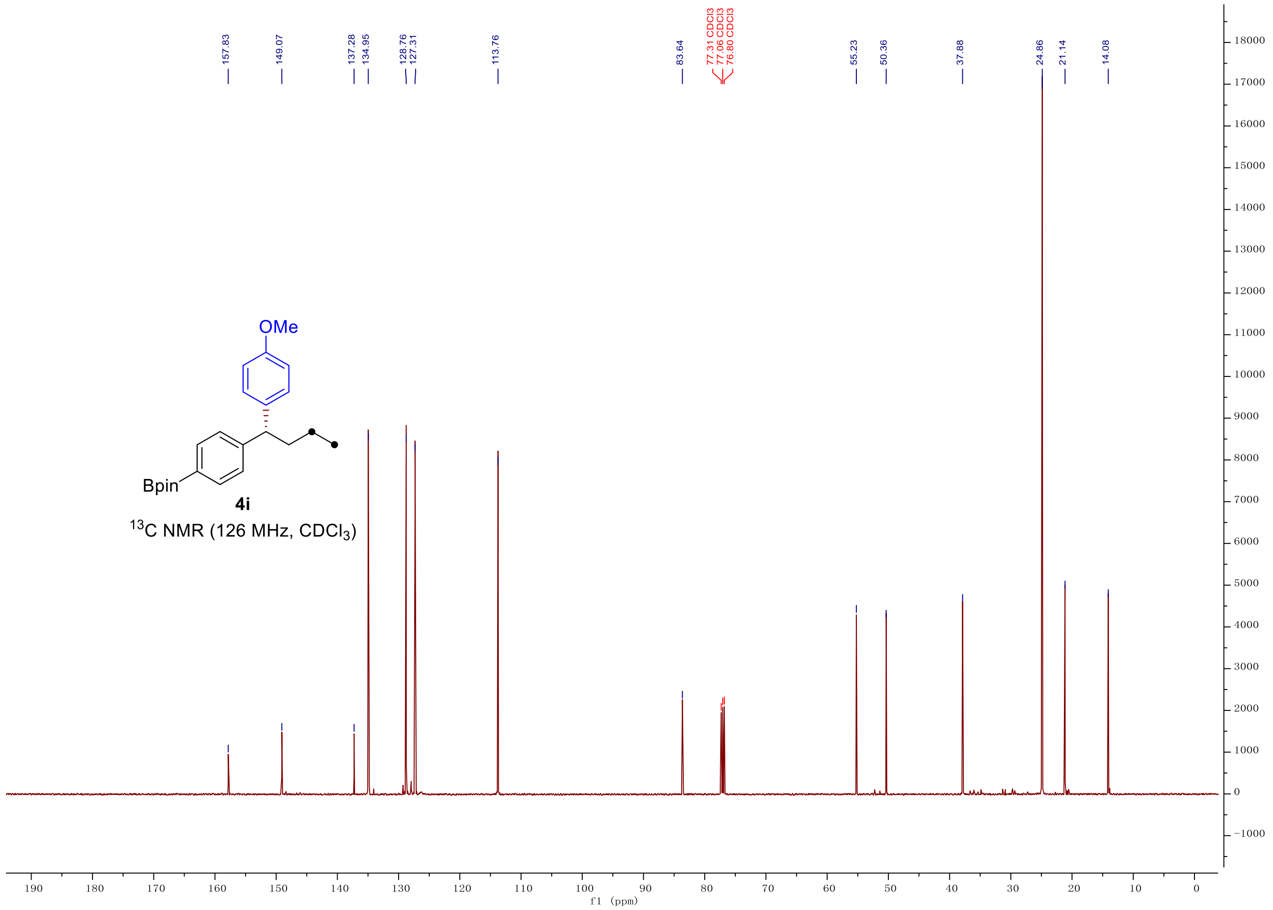

Supplementary Fig. 71. ^{13}C NMR of compound **4i**

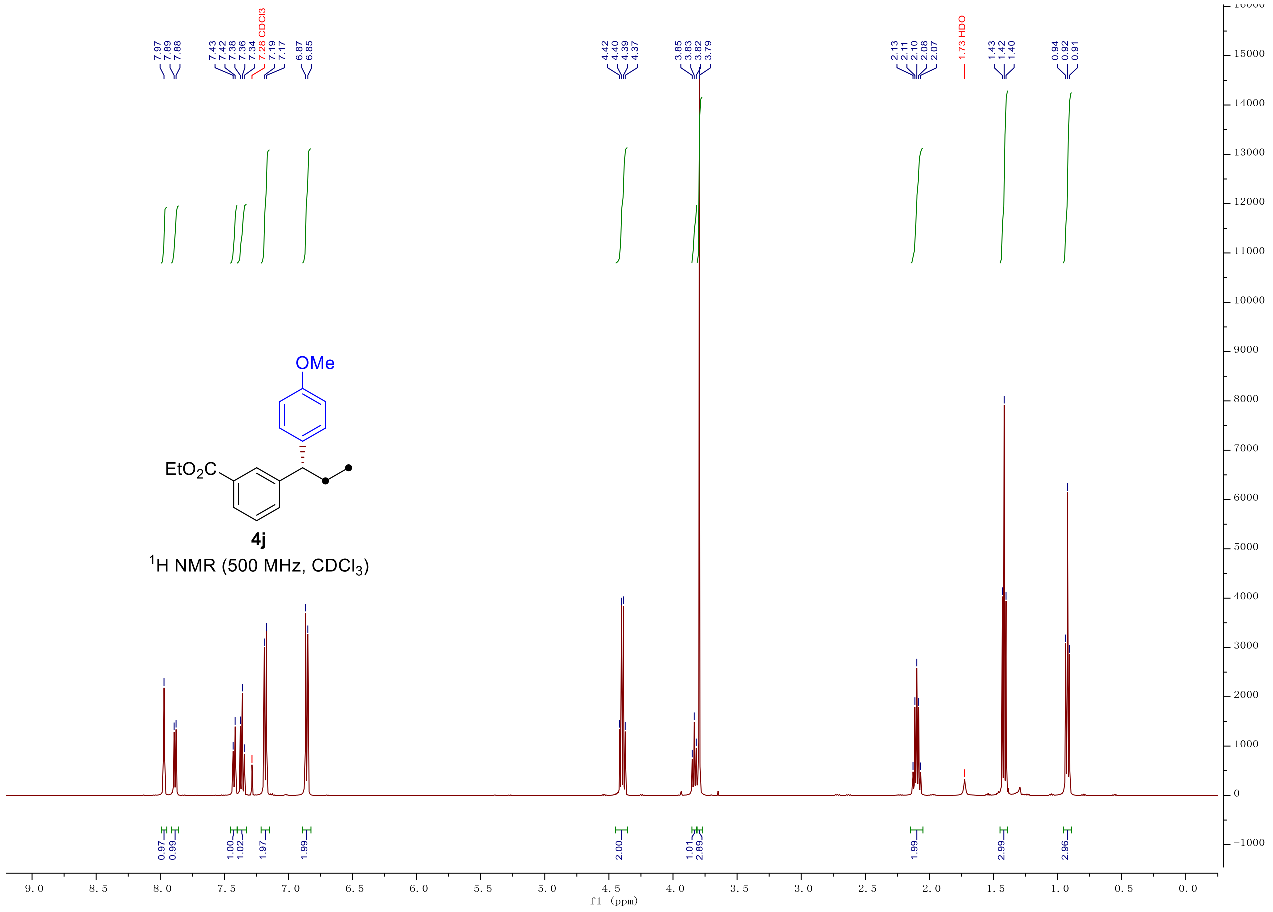

Supplementary Fig. 72. ^{1}H NMR of compound **4j**

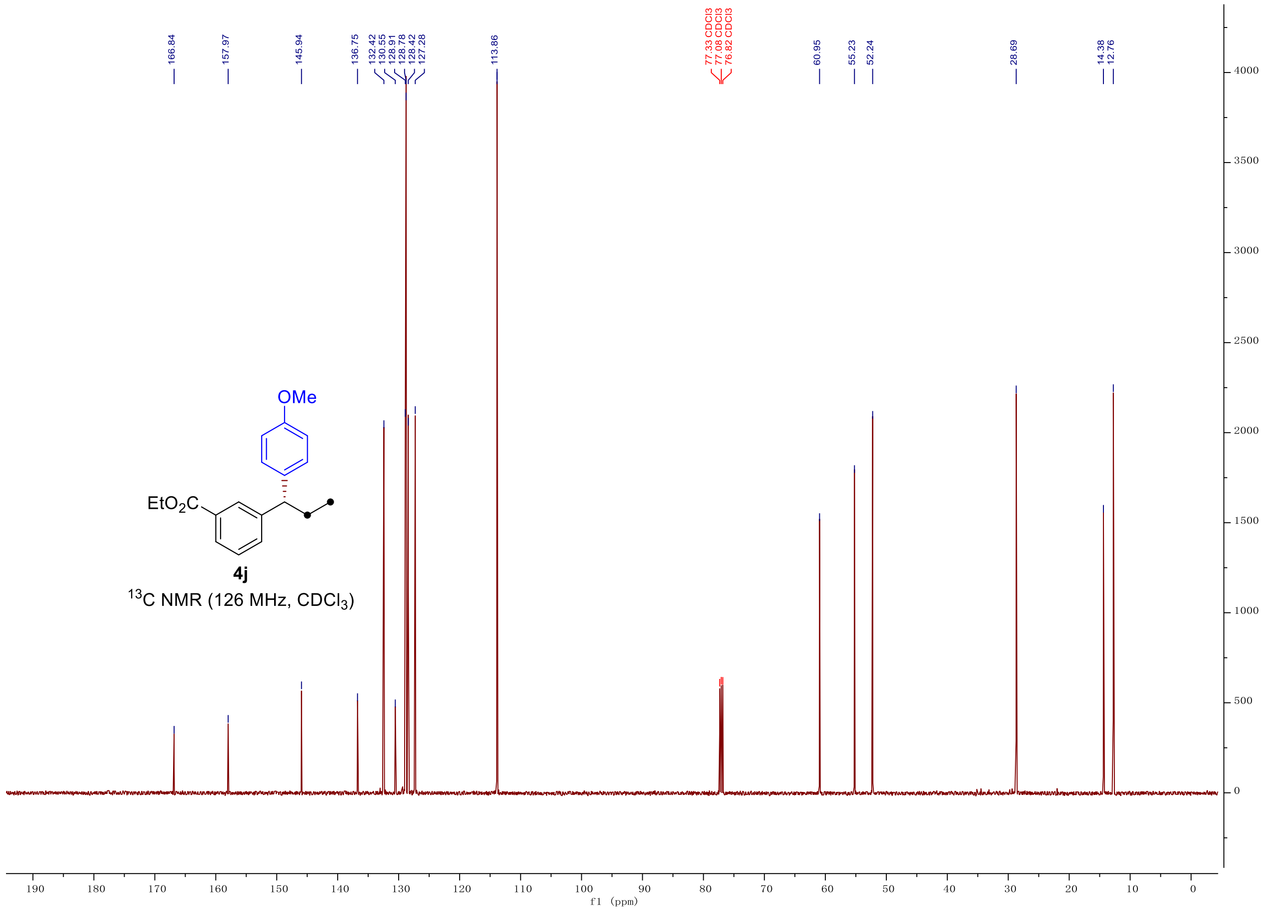

Supplementary Fig. 73. ^{13}C NMR of compound **4j**

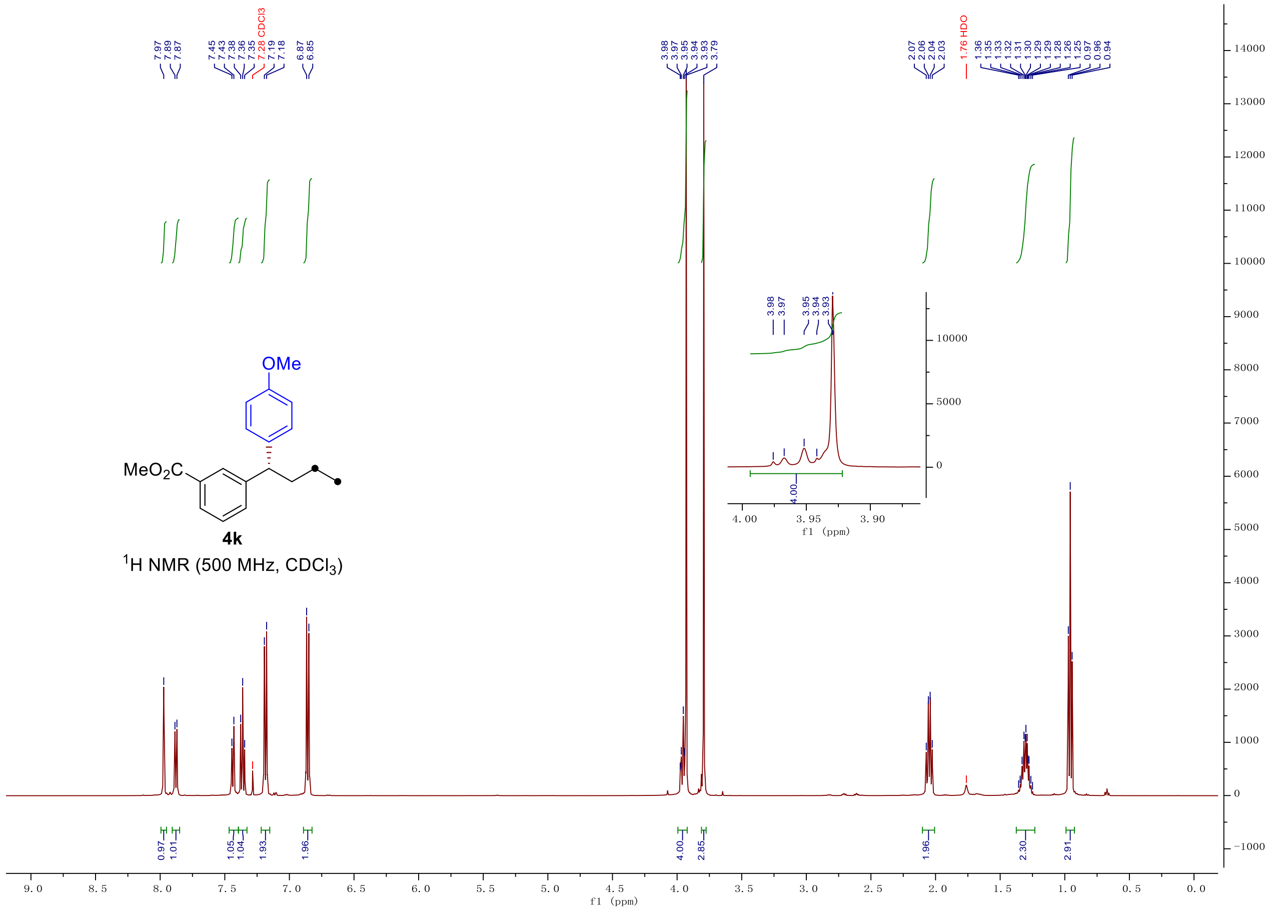

Supplementary Fig. 74. ^{1}H NMR of compound **4k**

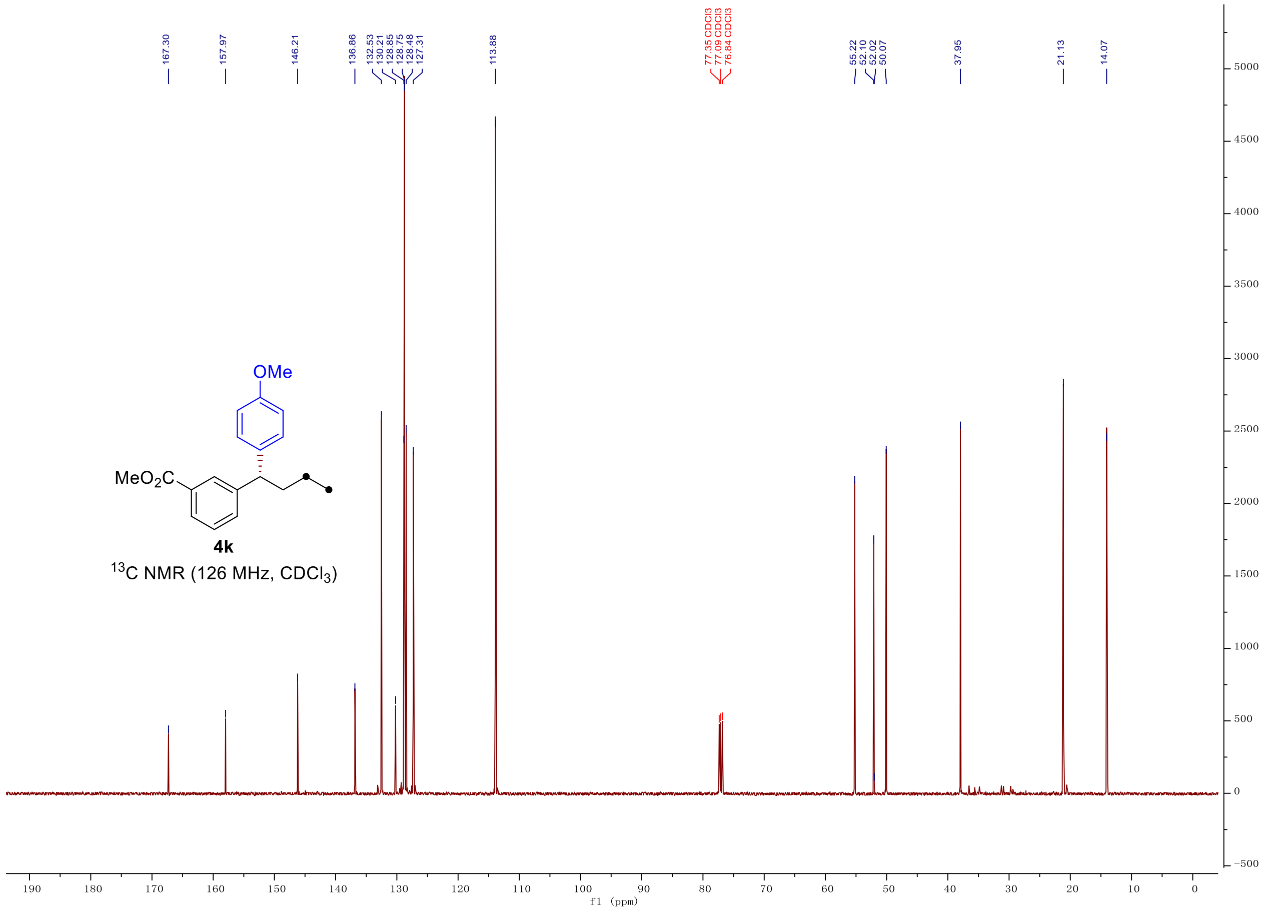

Supplementary Fig. 75. ^{13}C NMR of compound **4k**

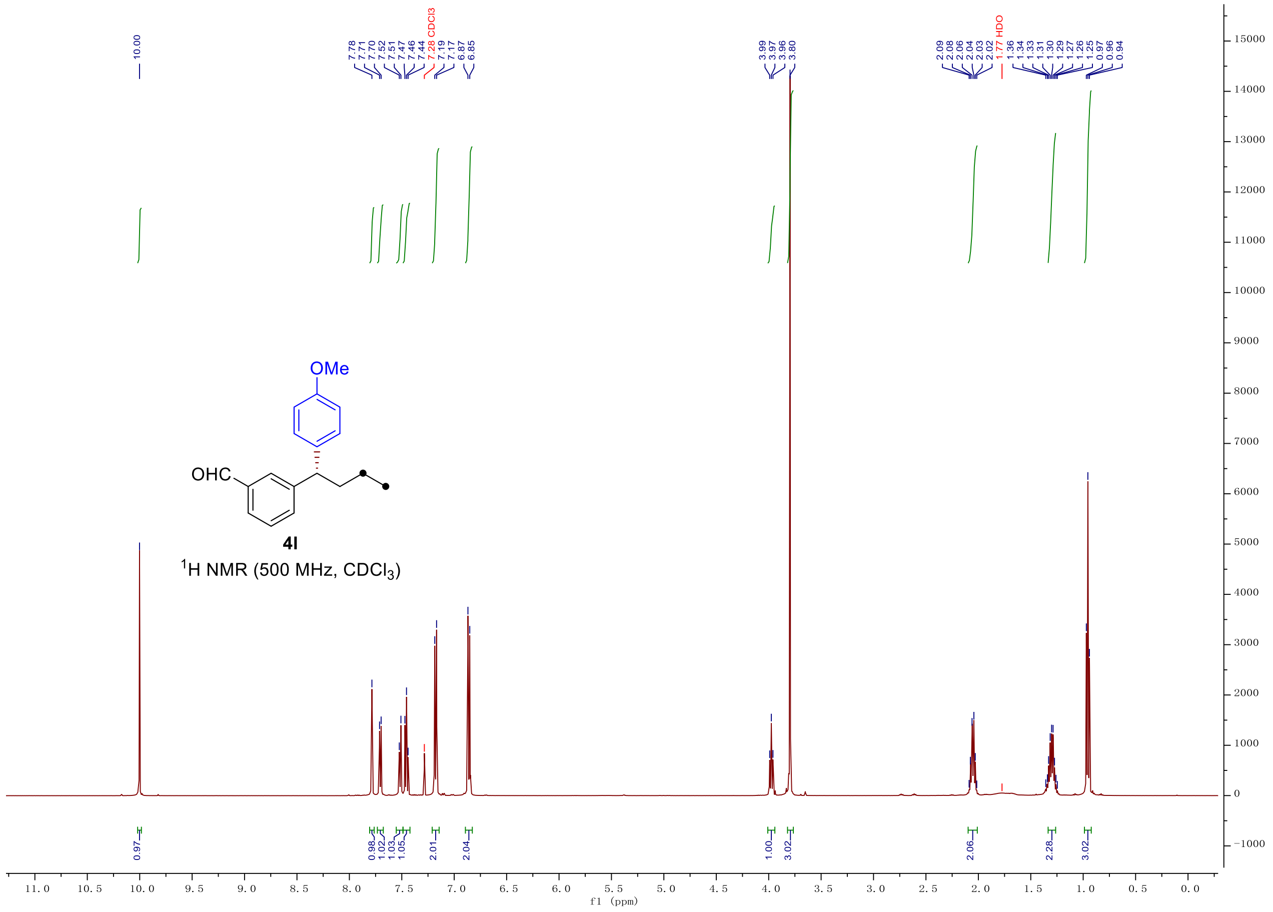

Supplementary Fig. 76. ^{1}H NMR of compound **4l**

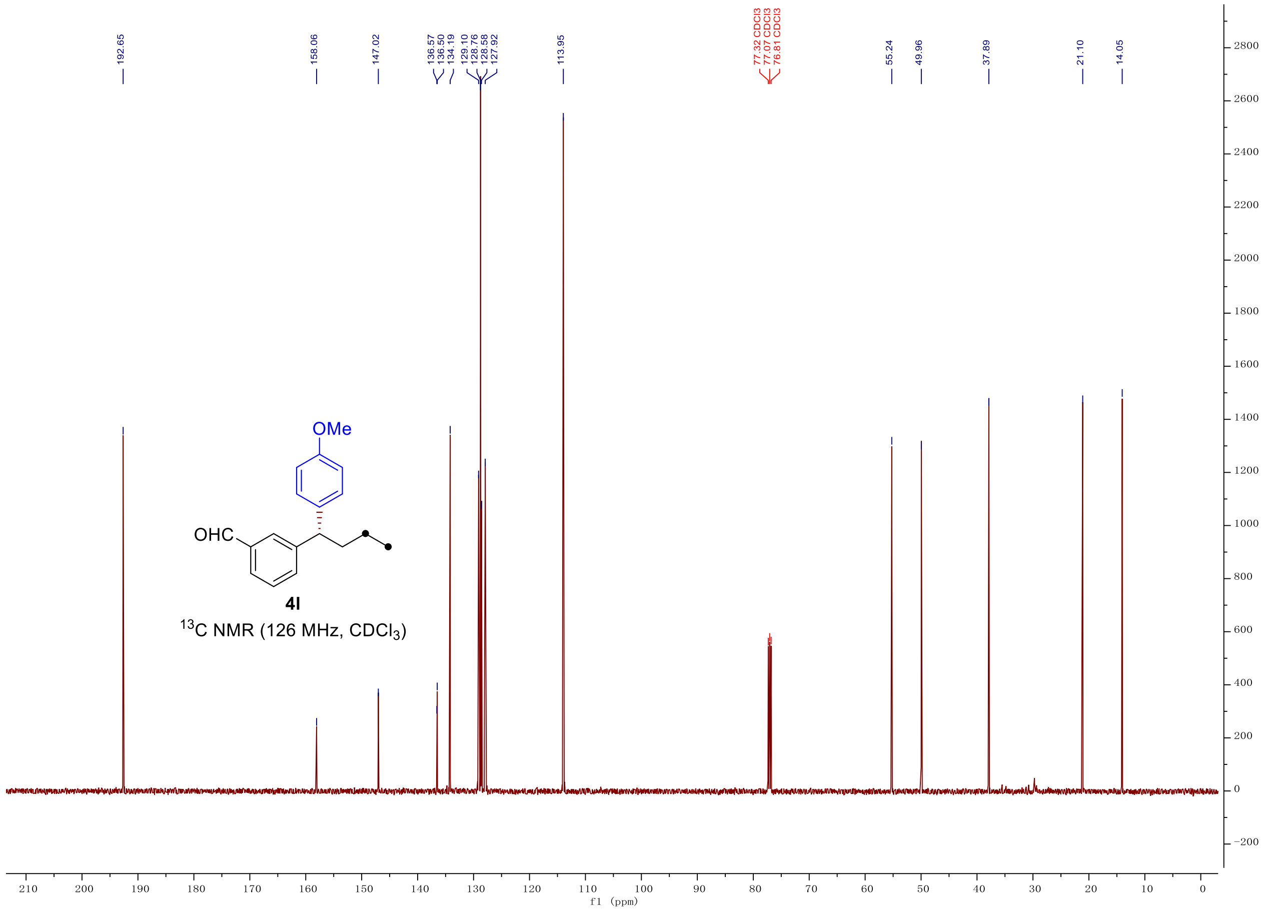

Supplementary Fig. 77. ^{13}C NMR of compound **4l**

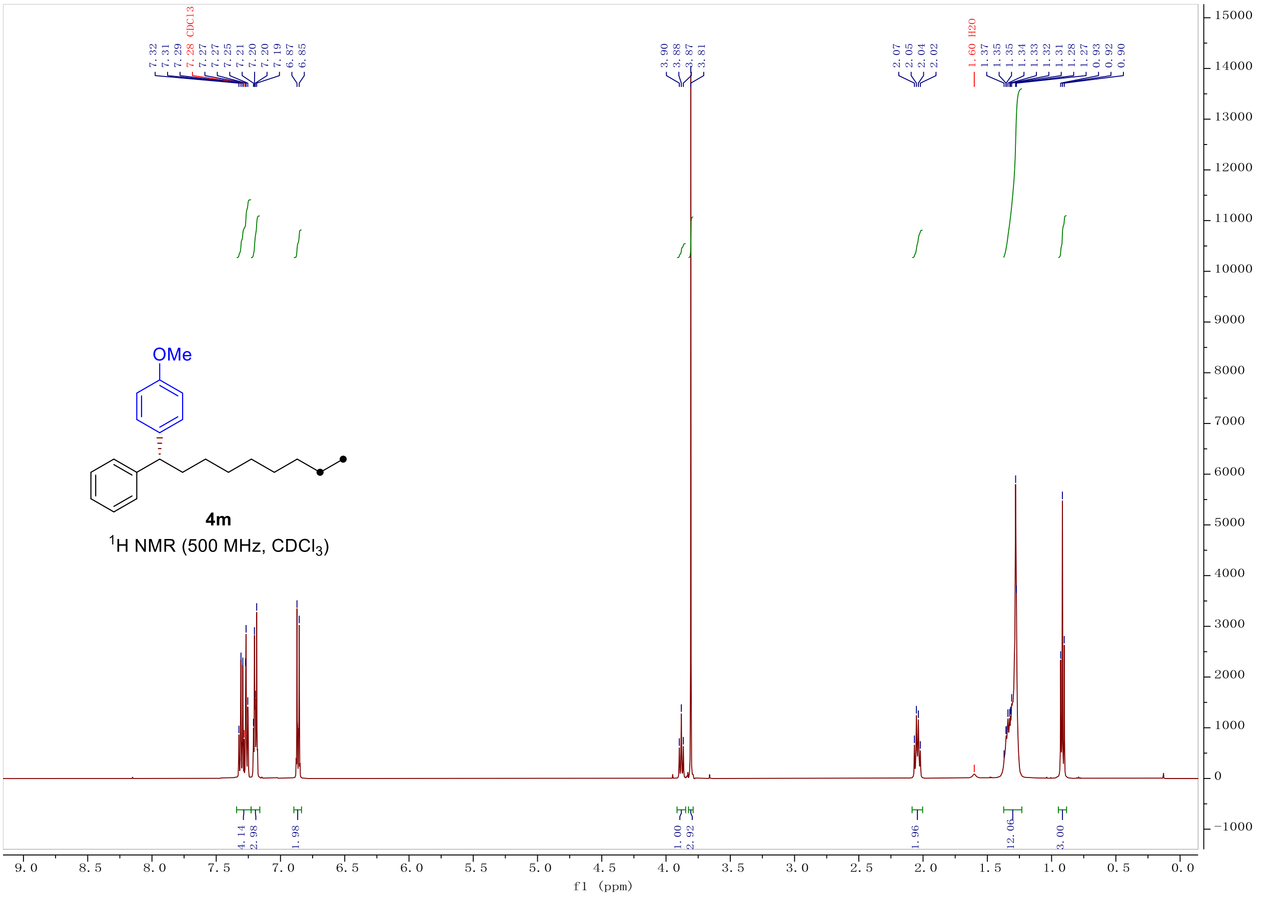

Supplementary Fig. 78. ^{1}H NMR of compound **4m**

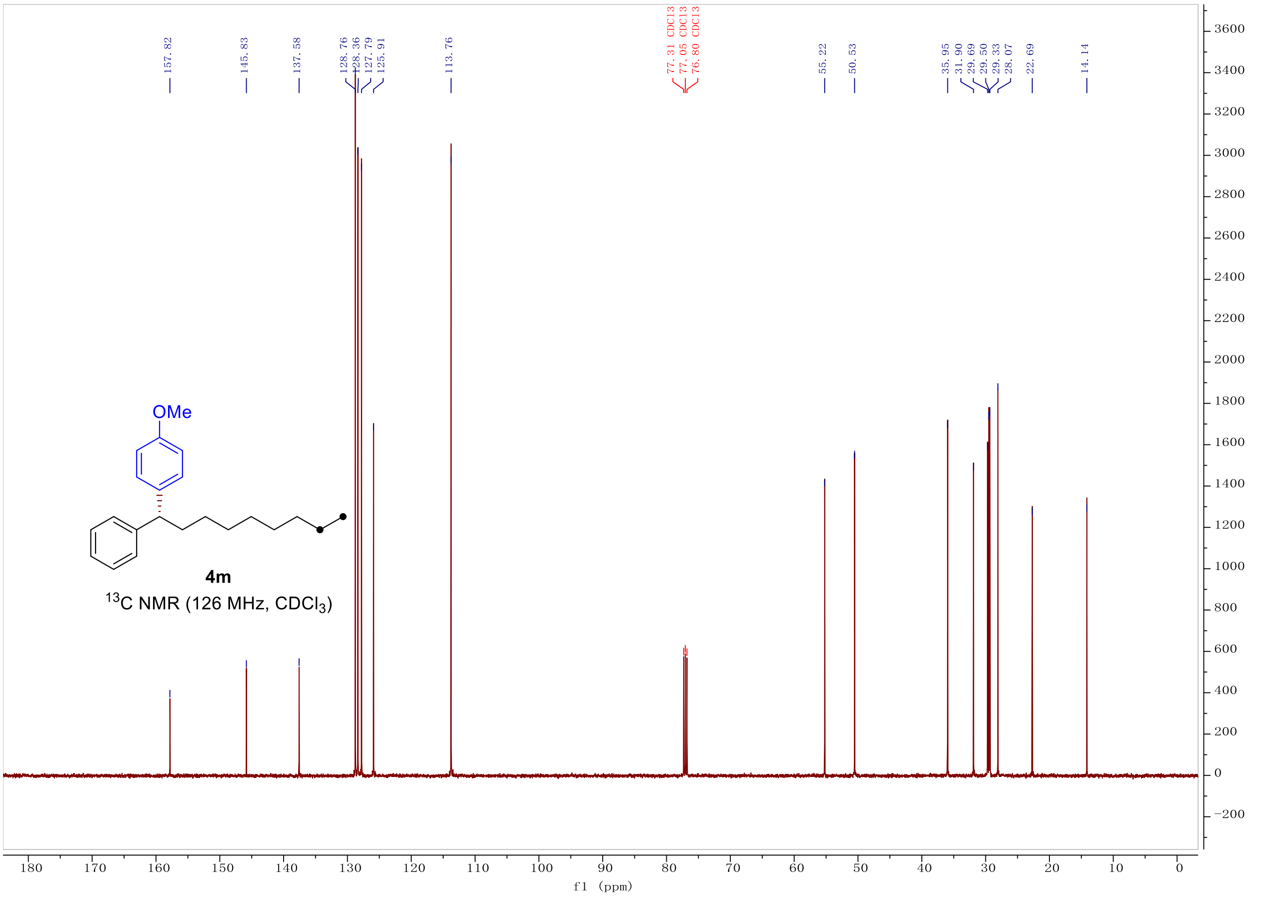

Supplementary Fig. 79. ^{13}C NMR of compound **4m**

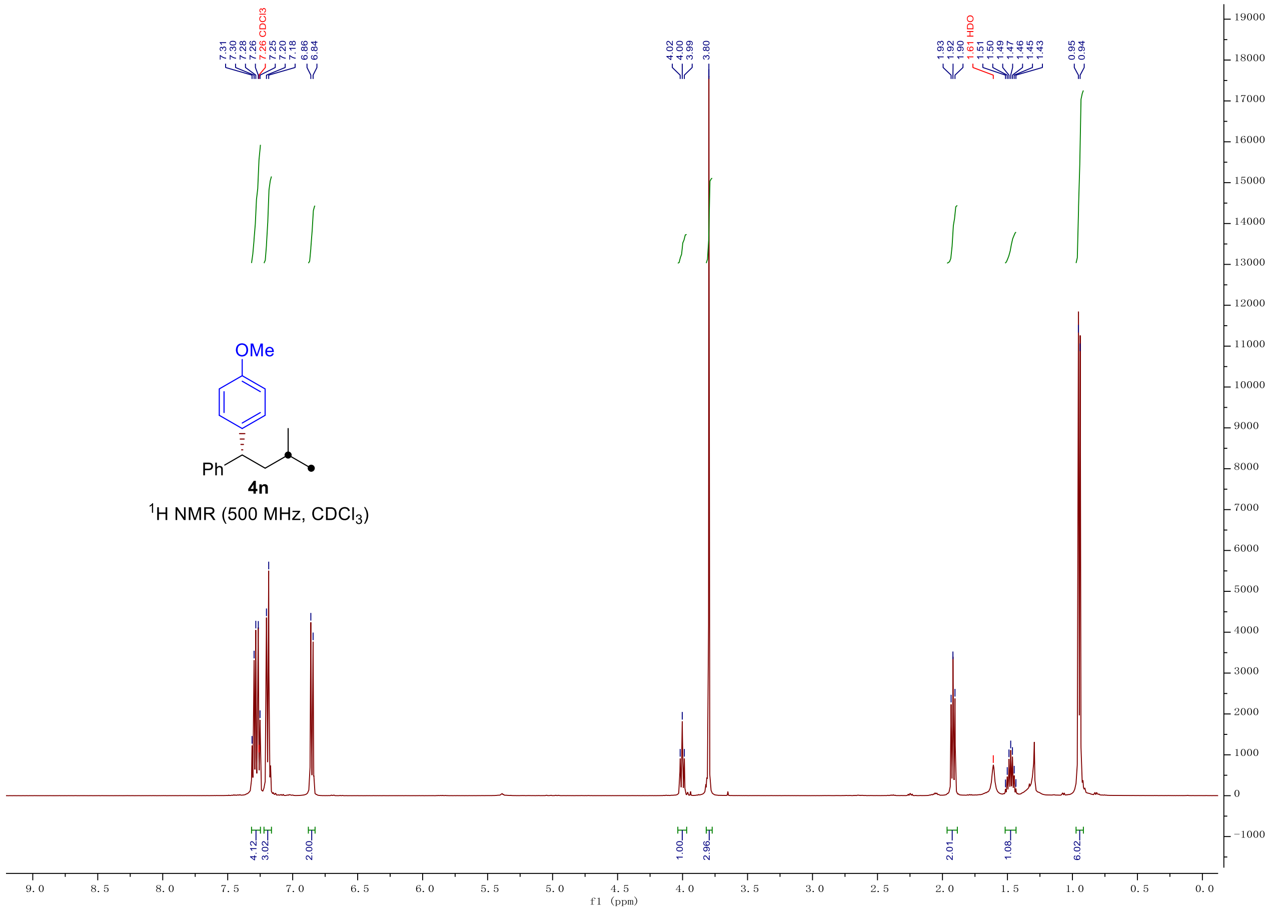

Supplementary Fig. 80. ^{1}H NMR of compound **4n**

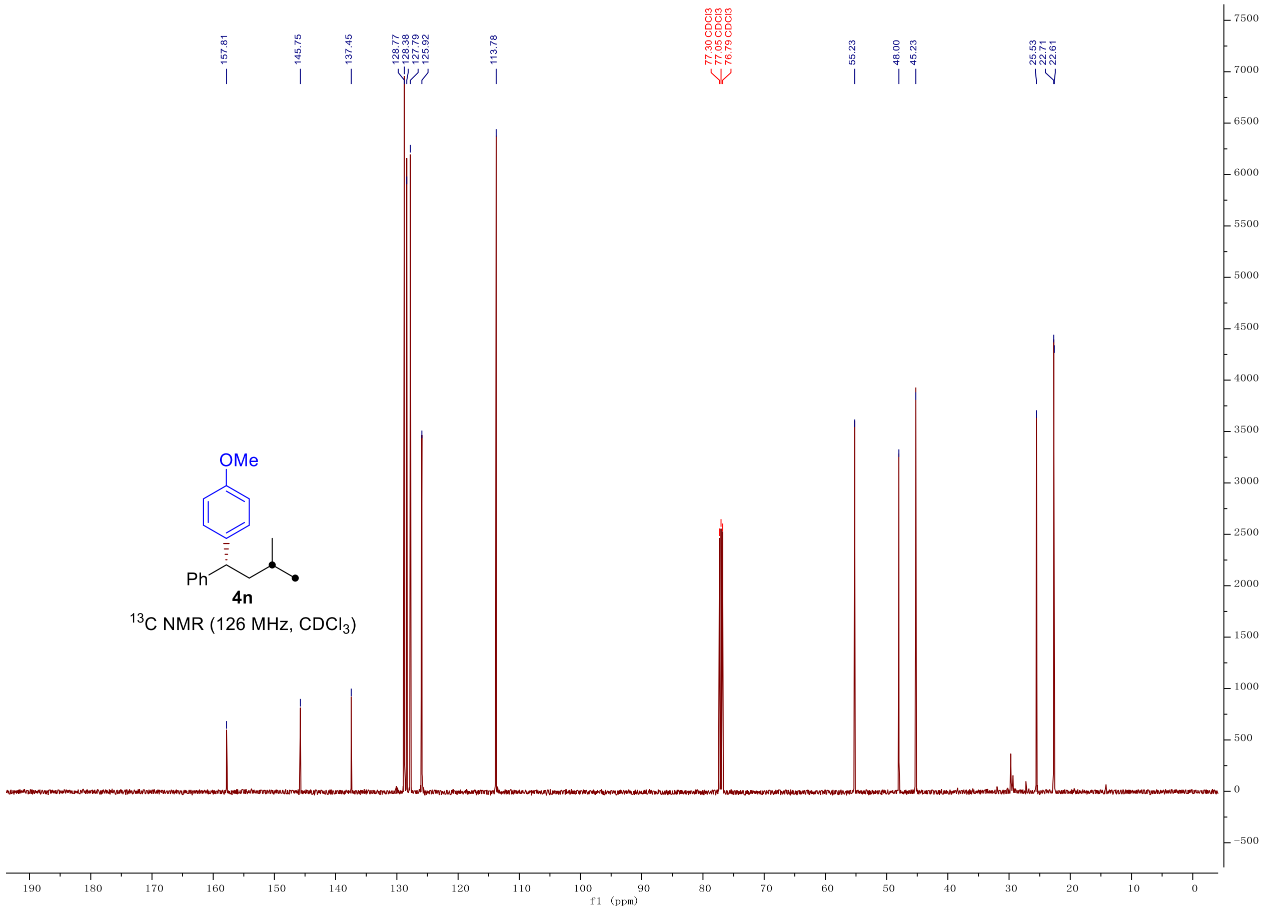

Supplementary Fig. 81. ^{13}C NMR of compound **4n**

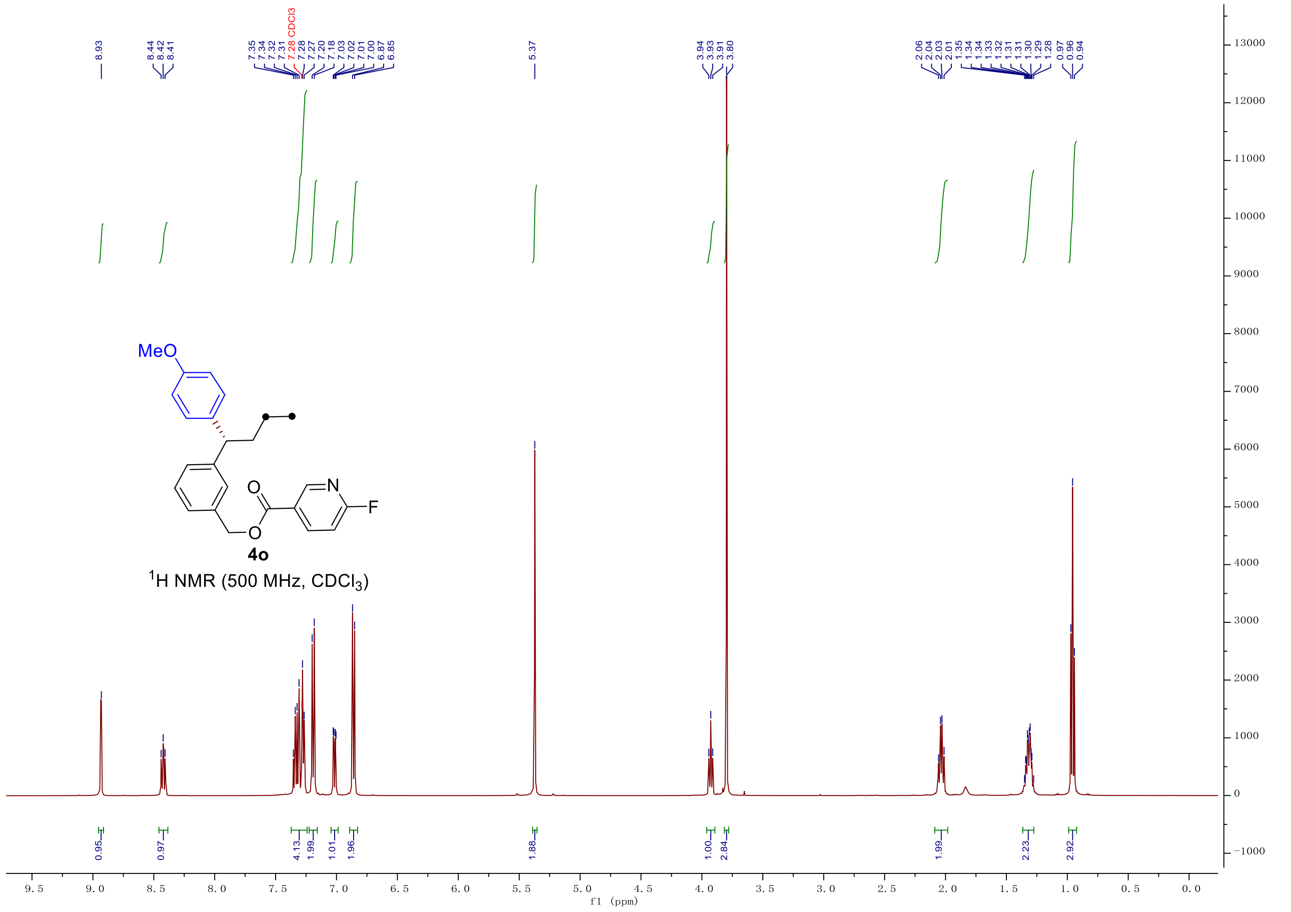

Supplementary Fig. 82. ^{1}H NMR of compound **4o**

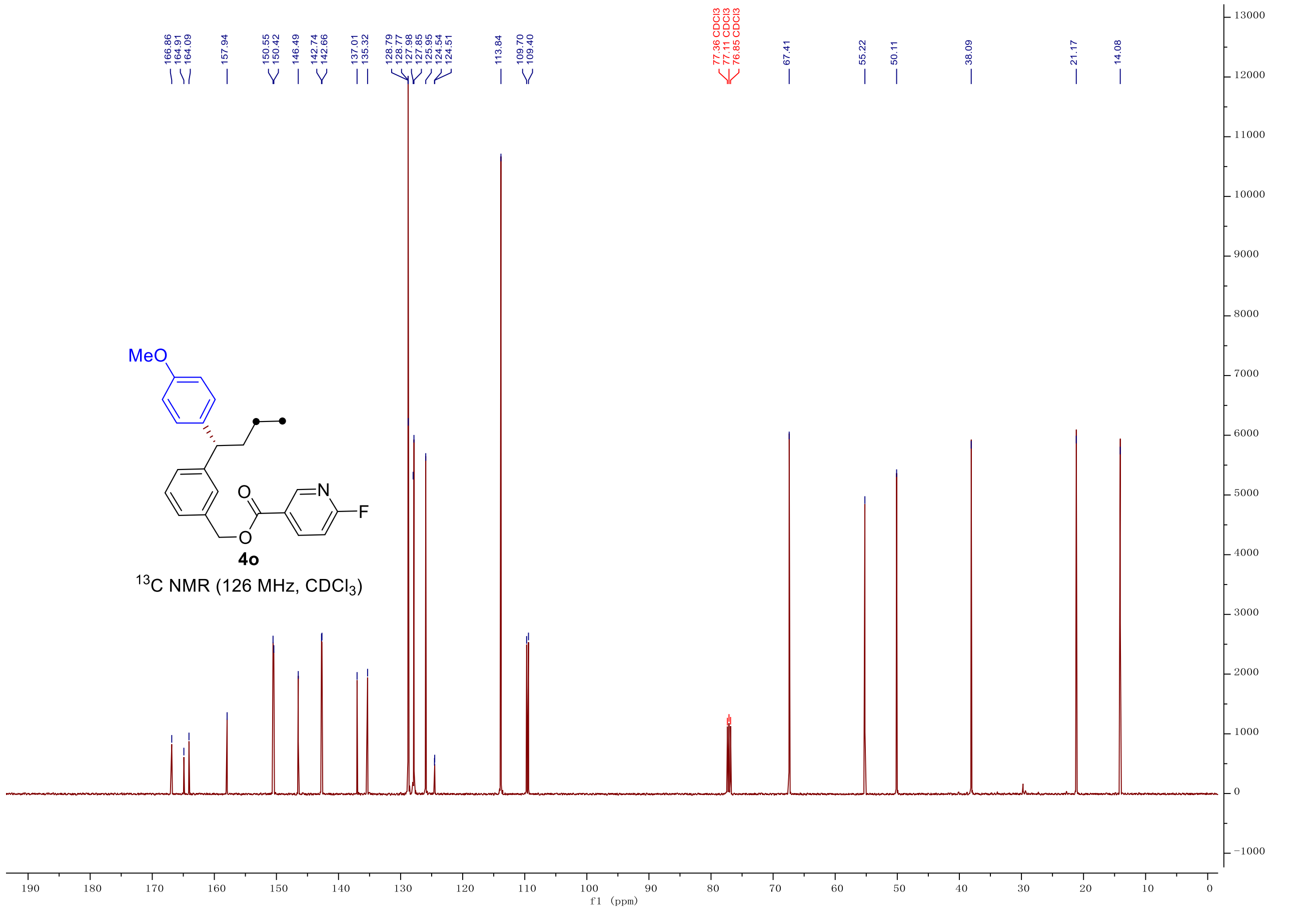

Supplementary Fig. 83. ^{13}C NMR of compound **4o**

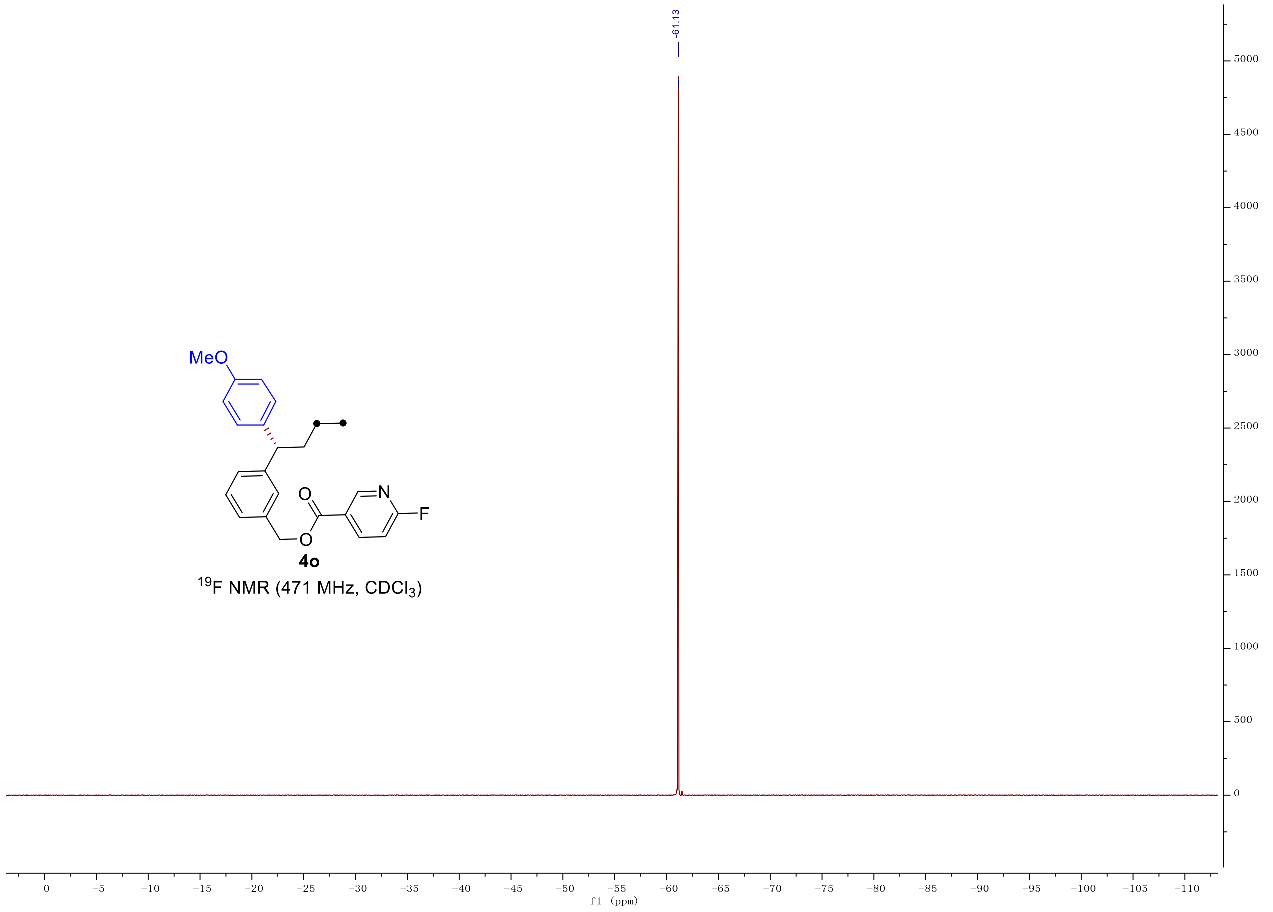

Supplementary Fig. 84. ^{19}F NMR of compound **4o**

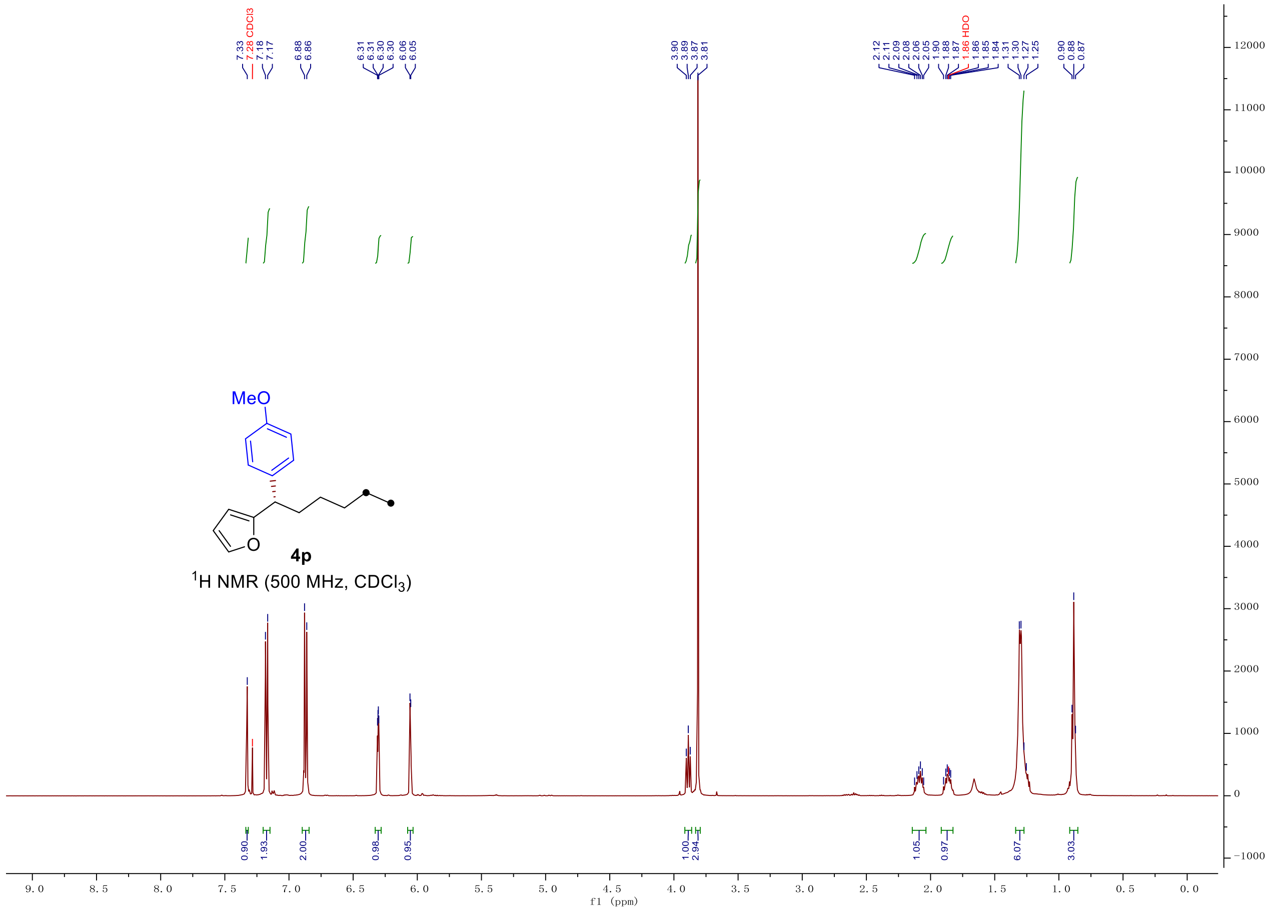

Supplementary Fig. 85. ^{1}H NMR of compound **4p**

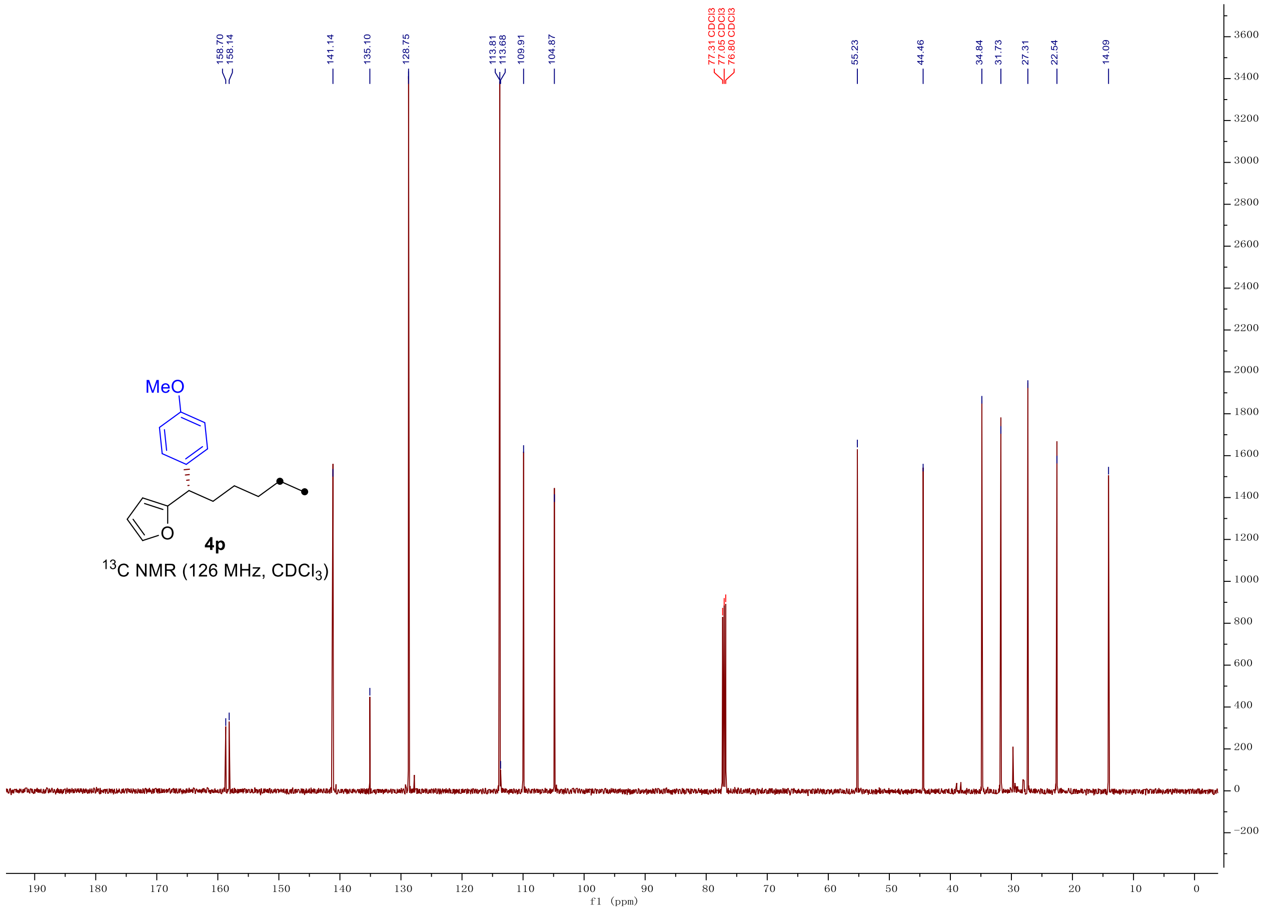

Supplementary Fig. 86. ^{13}C NMR of compound **4p**

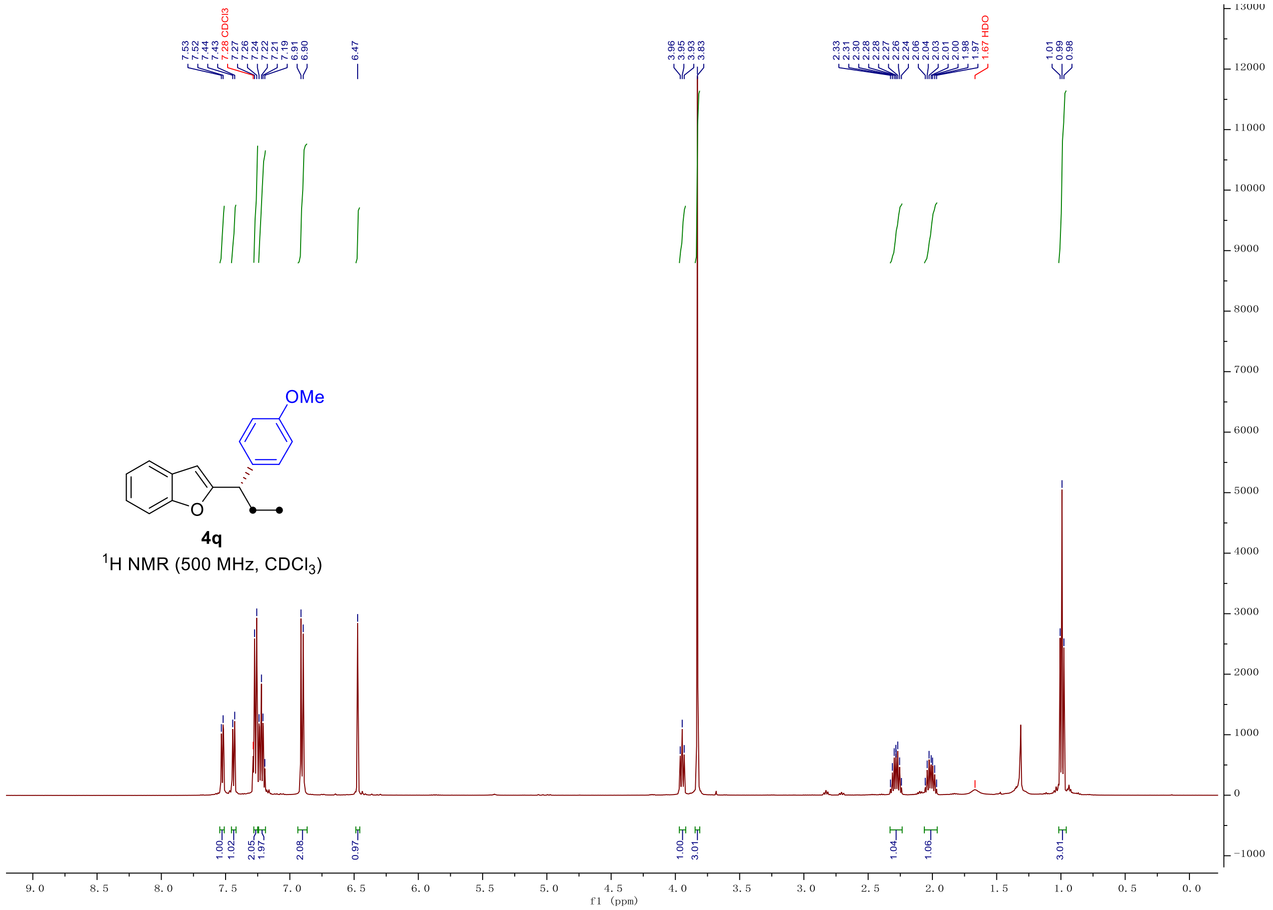

Supplementary Fig. 87. ^{1}H NMR of compound **4q**

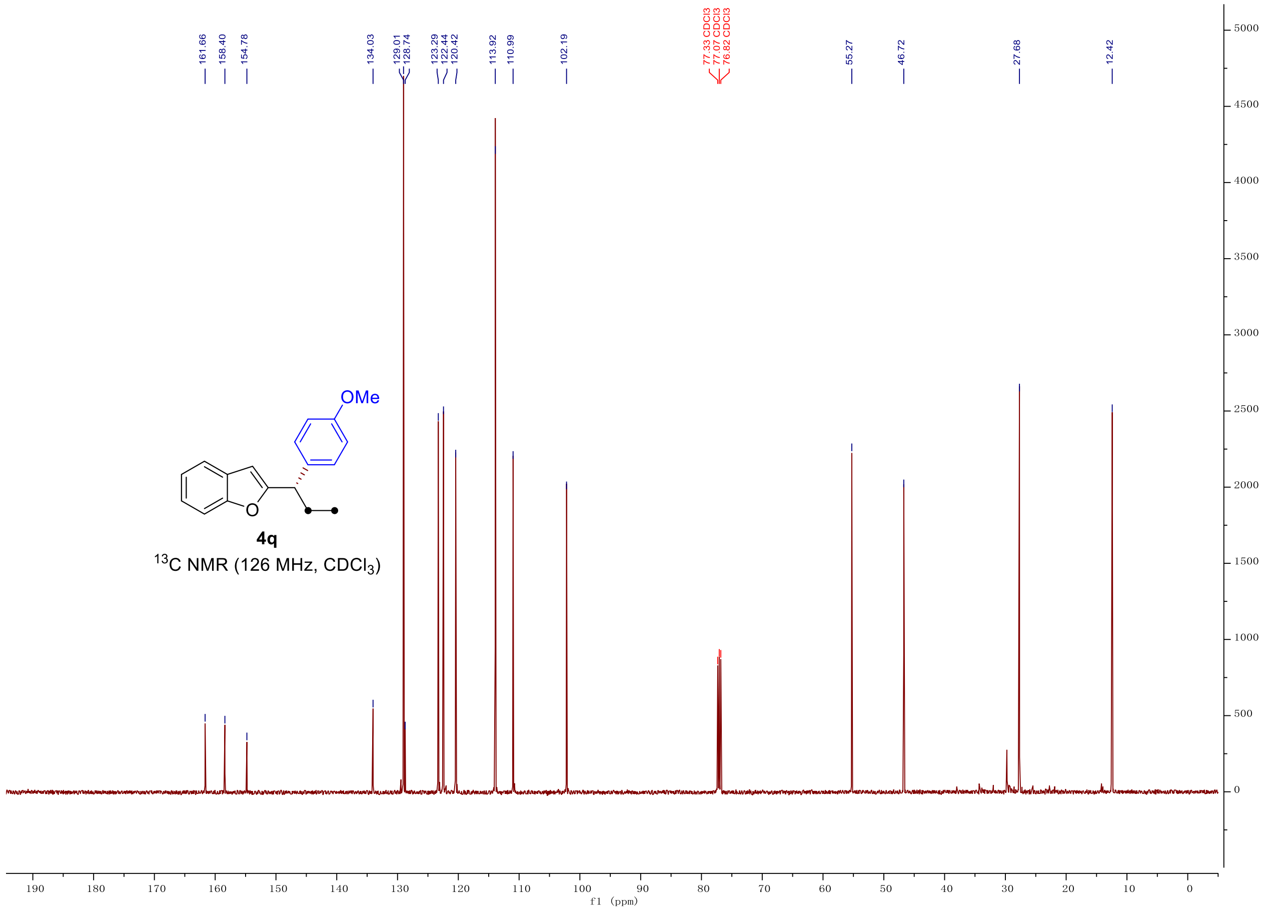

Supplementary Fig. 88. ^{13}C NMR of compound **4q**

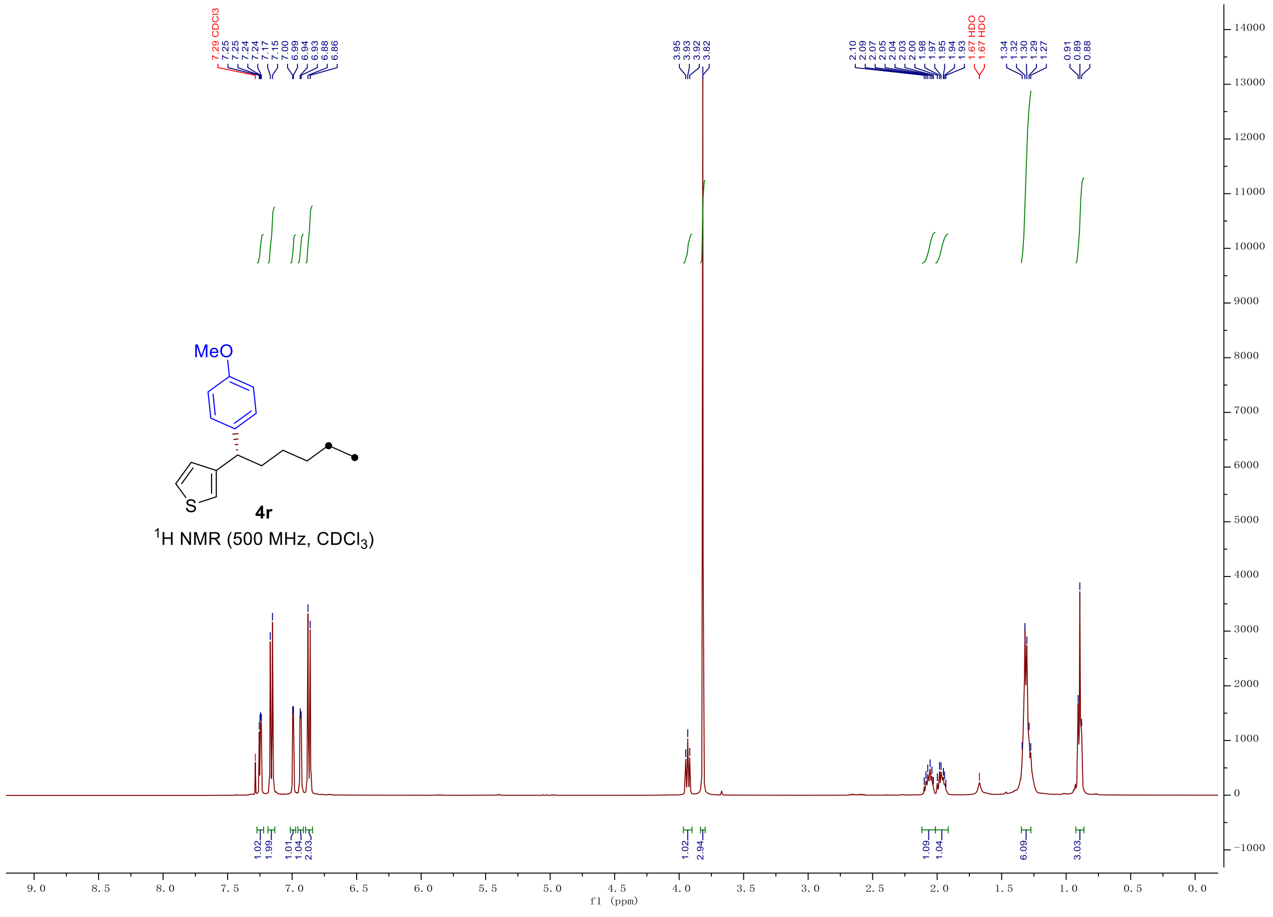

Supplementary Fig. 89. ^{1}H NMR of compound **4r**

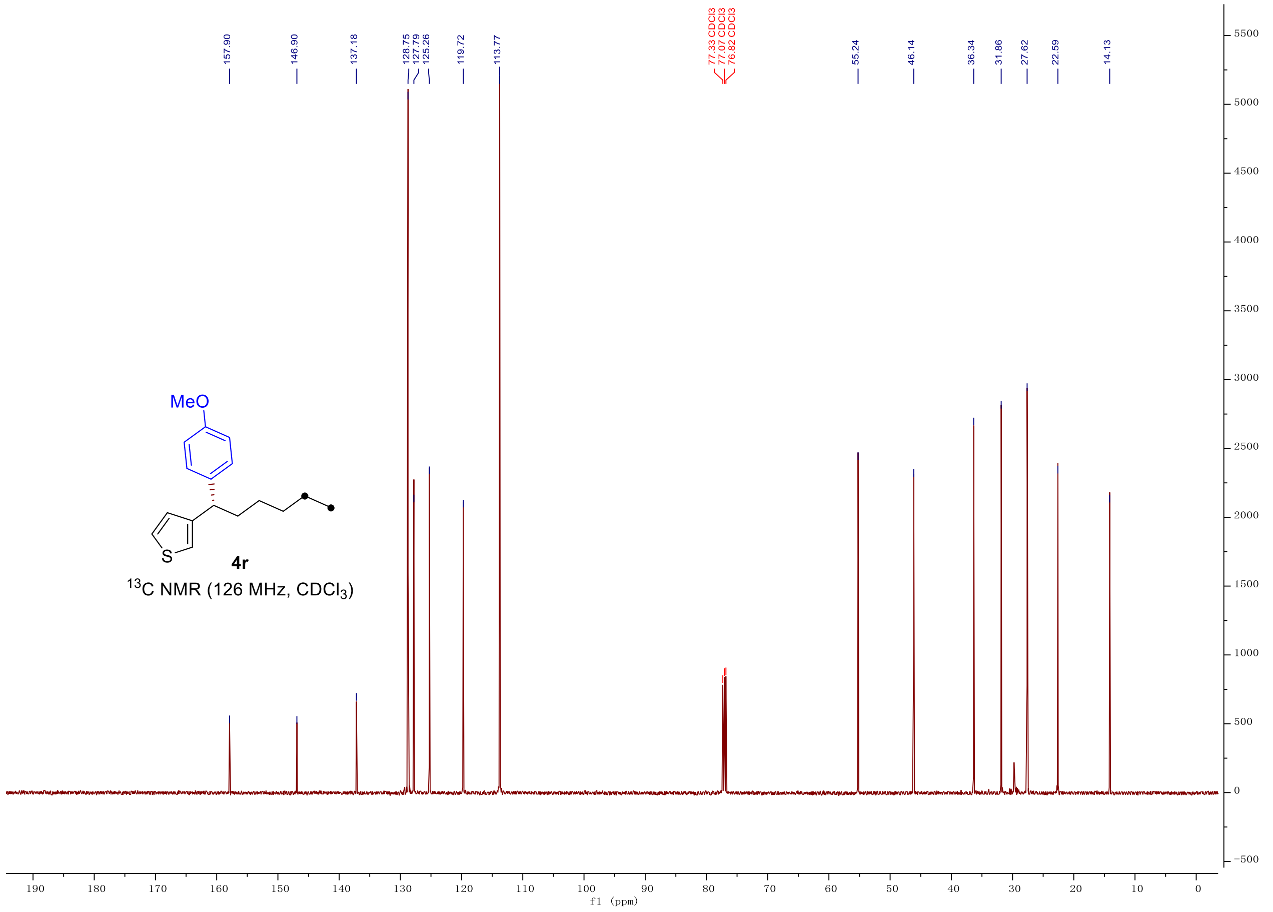

Supplementary Fig. 90. ^{13}C NMR of compound **4r**

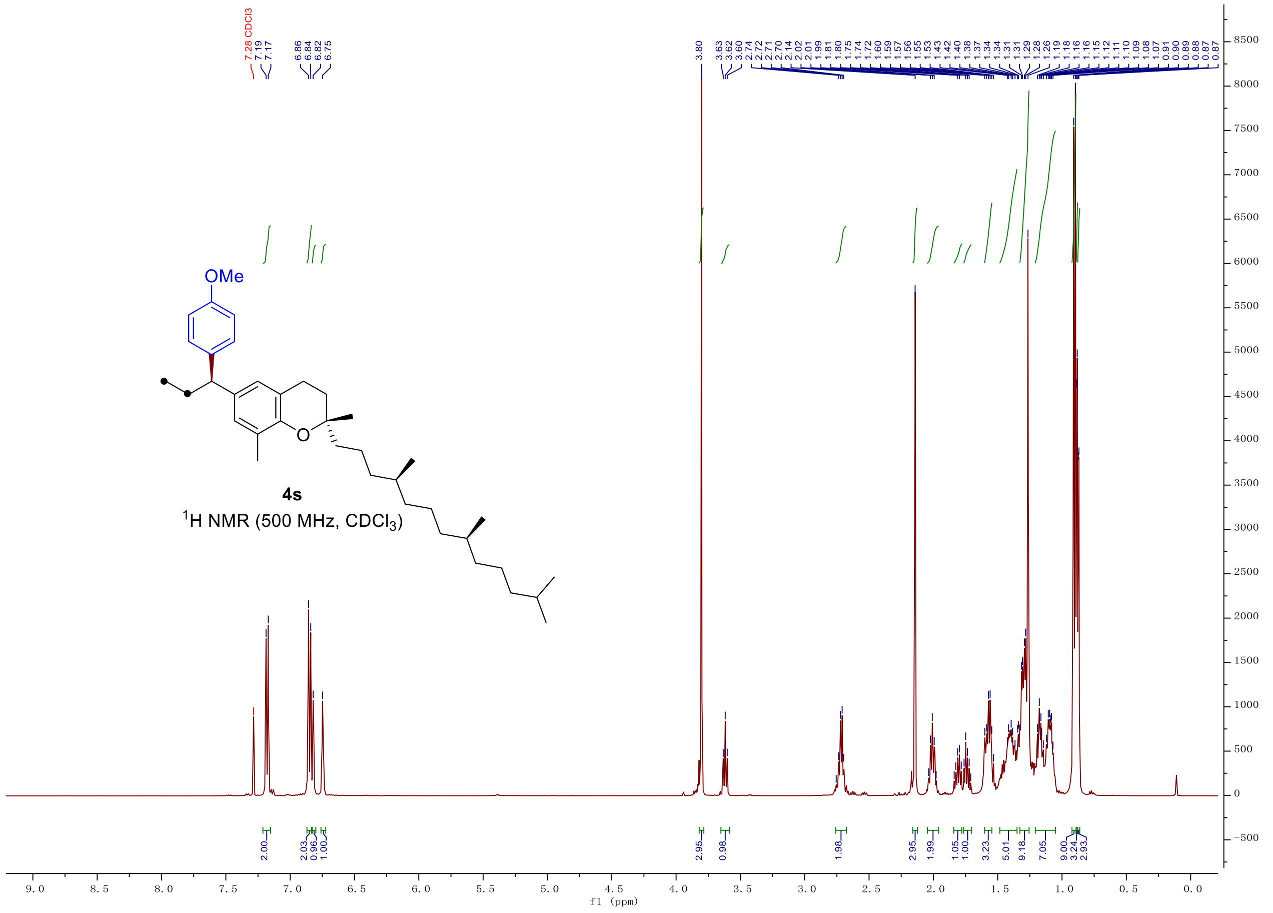

Supplementary Fig. 91. ^{1}H NMR of compound **4s**

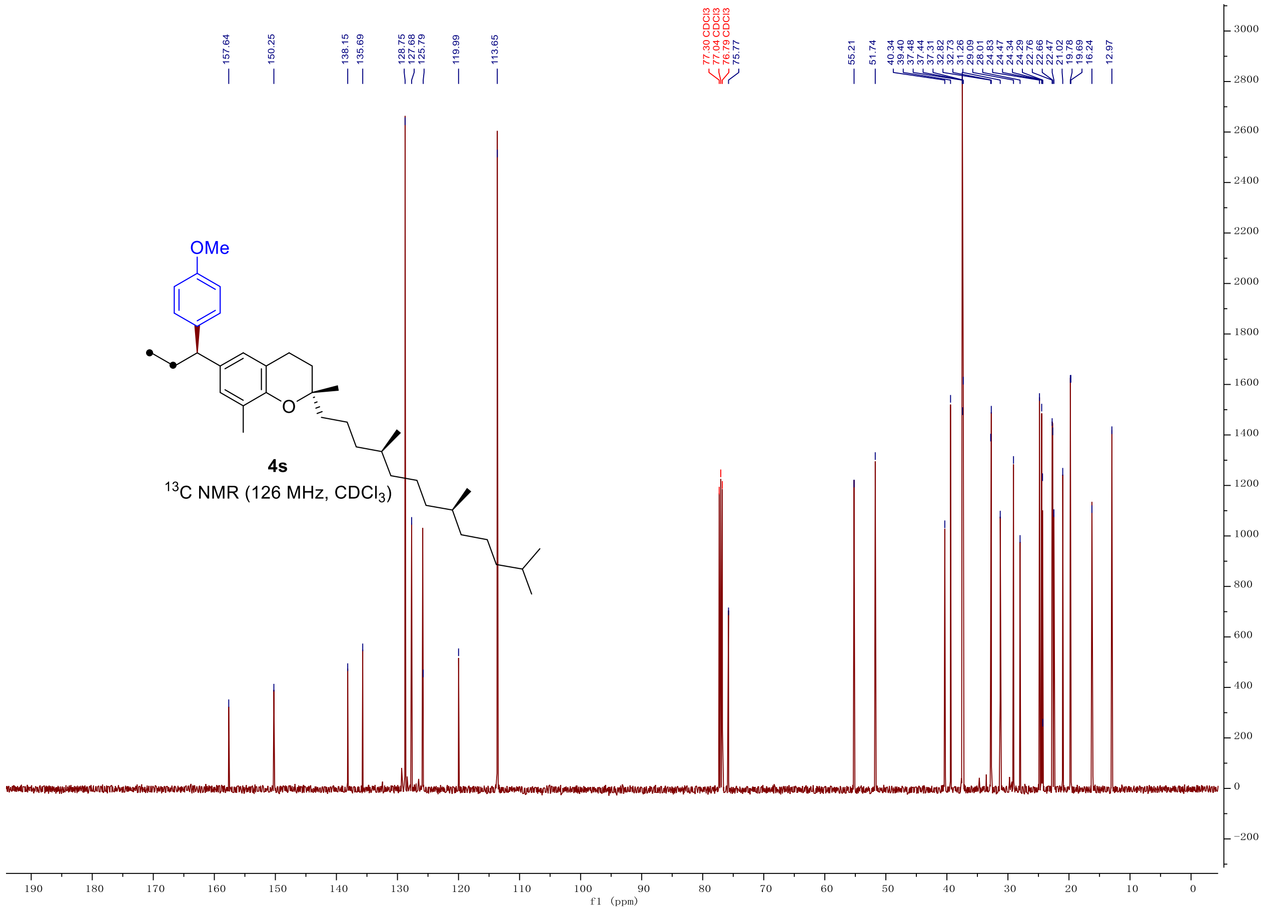

Supplementary Fig. 92. ^{13}C NMR of compound **4s**

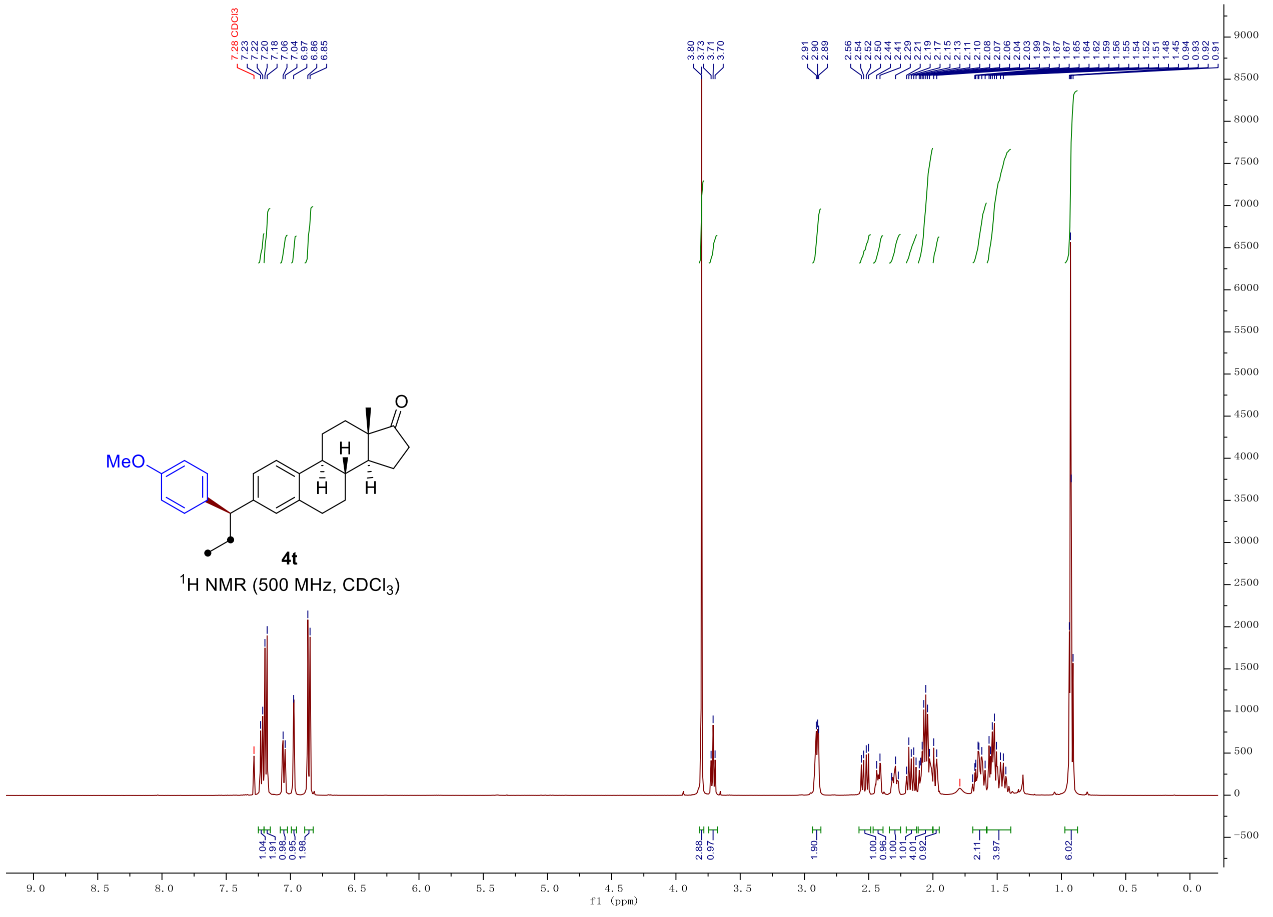

Supplementary Fig. 93. ^{1}H NMR of compound **4t**

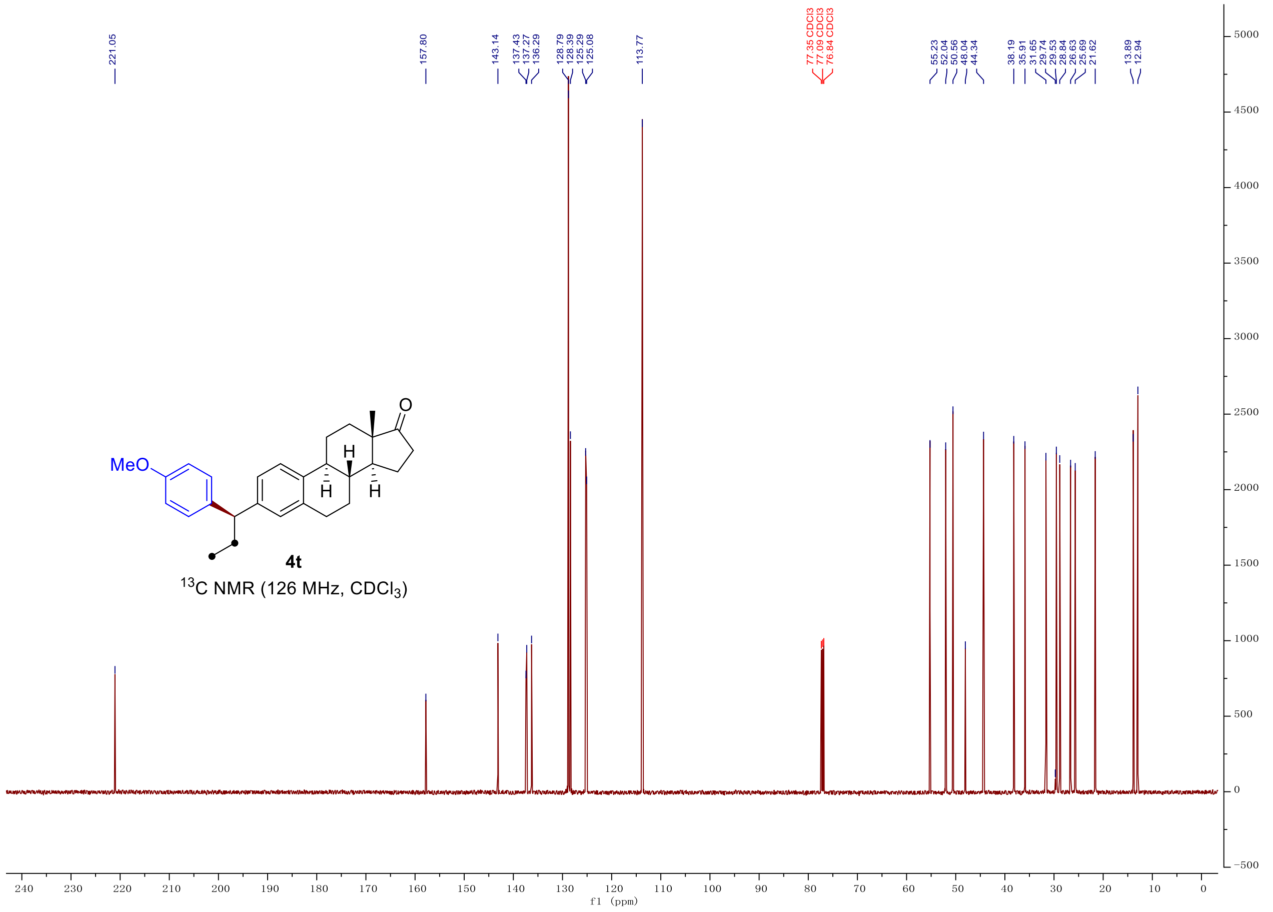

Supplementary Fig. 94. ^{13}C NMR of compound **4t**

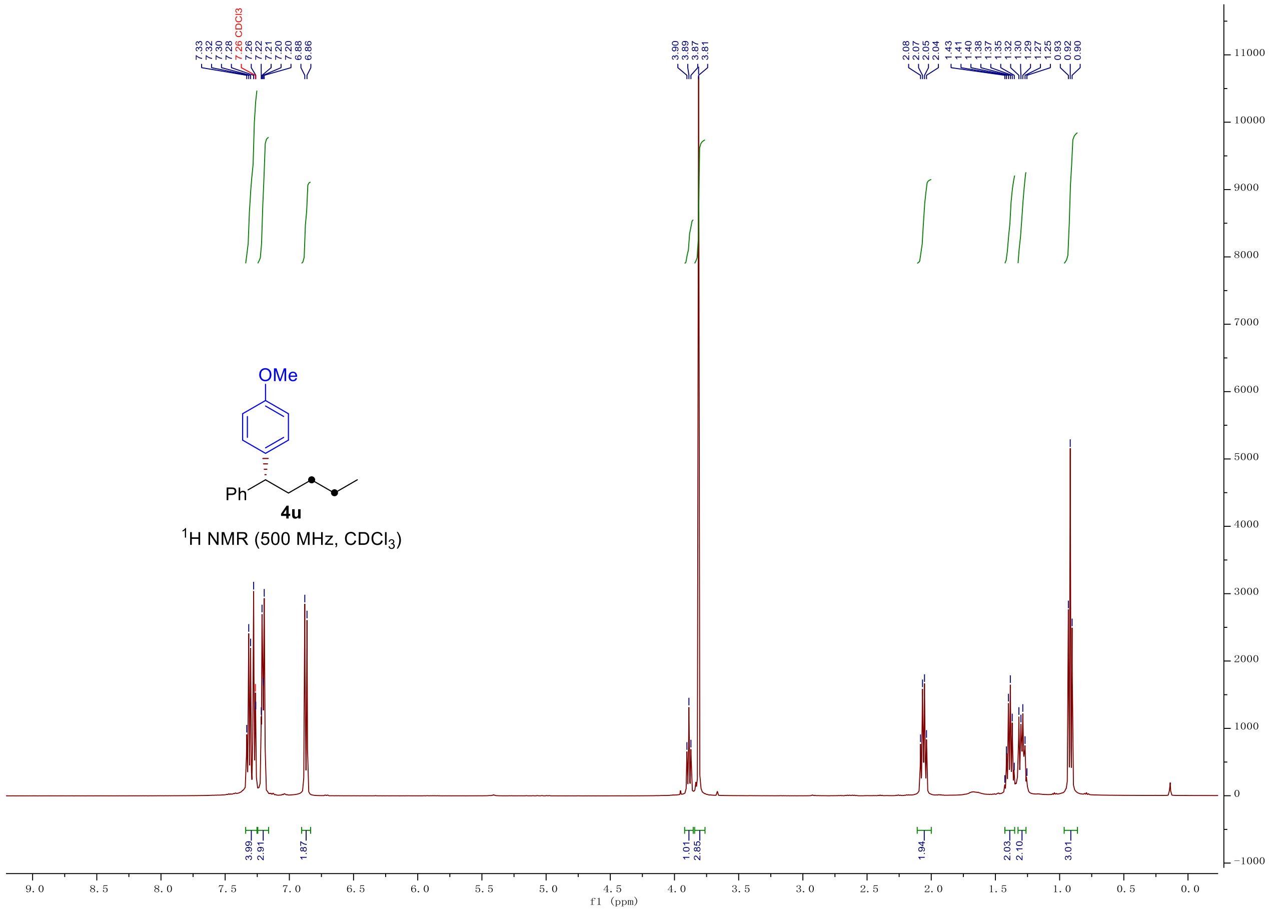

Supplementary Fig. 95. ^{1}H NMR of compound **4u**

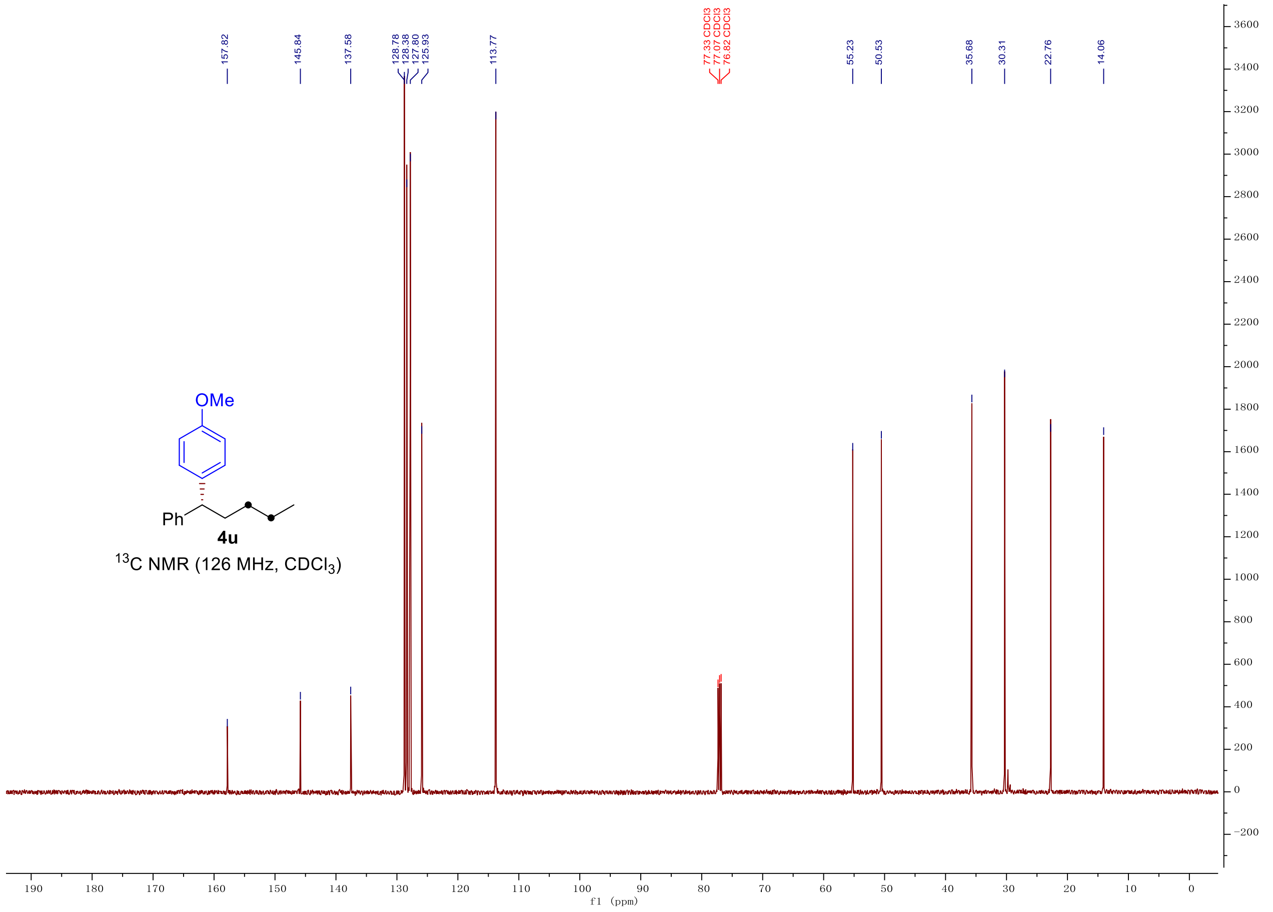

Supplementary Fig. 96. ^{13}C NMR of compound **4u**

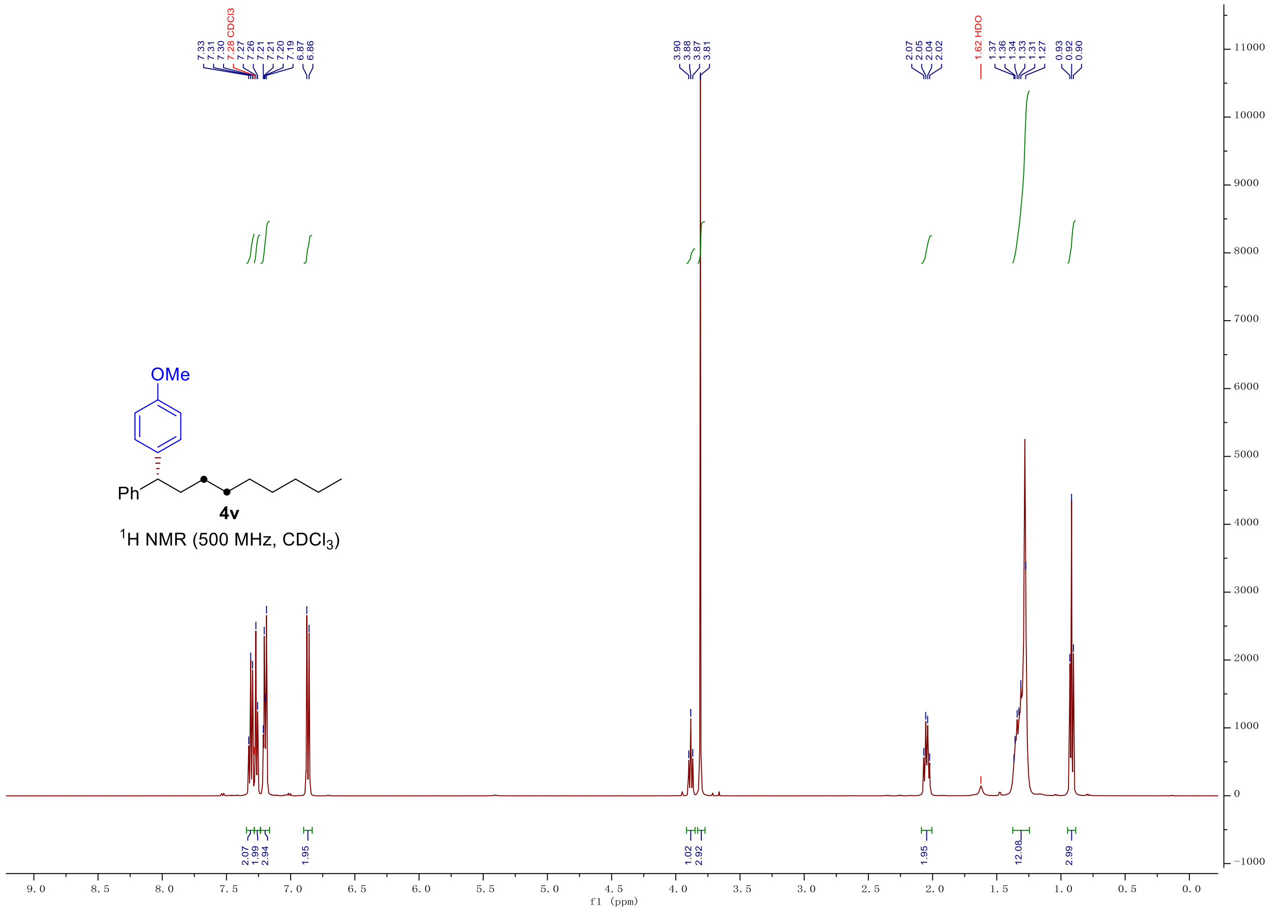

Supplementary Fig. 97. ^{1}H NMR of compound **4v**

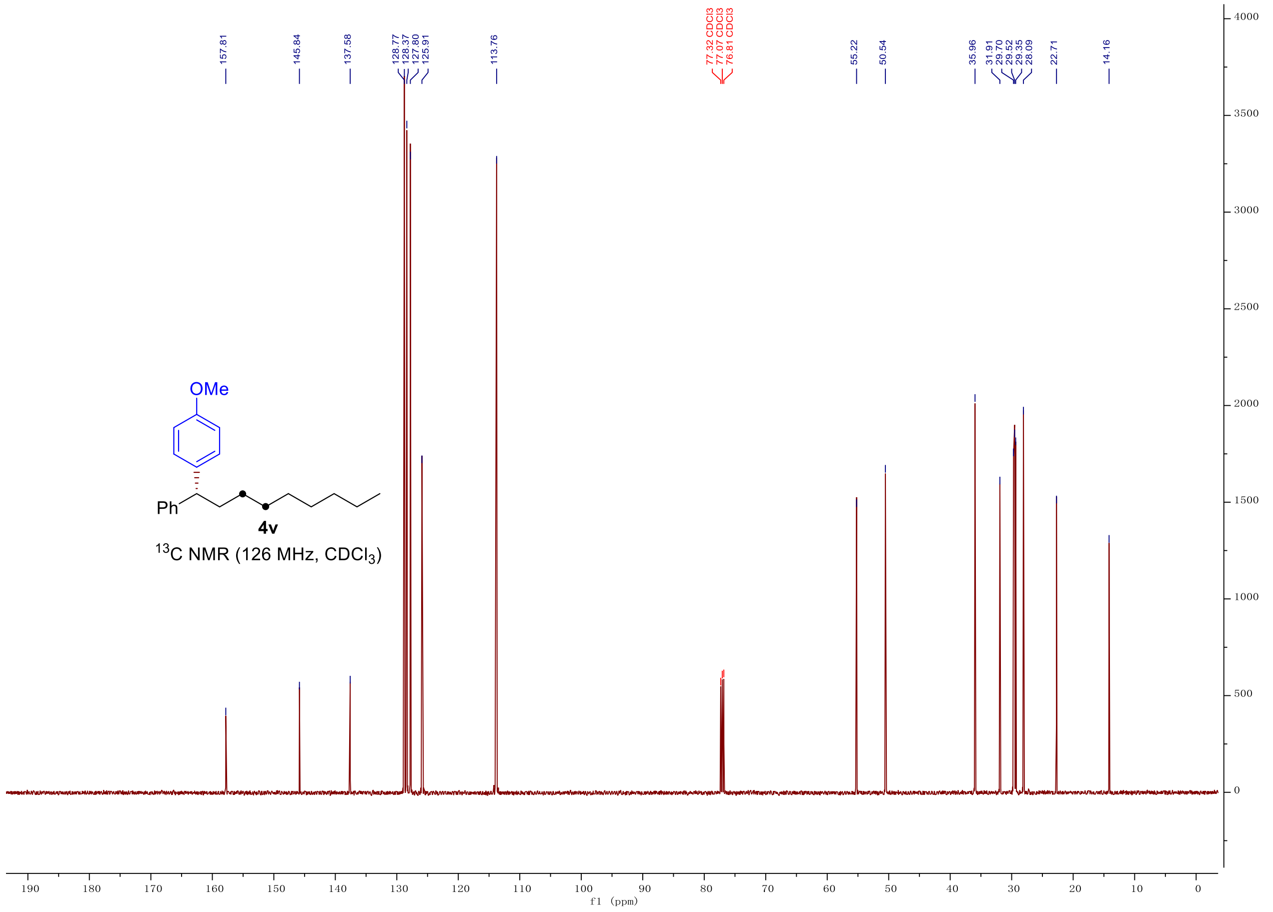

Supplementary Fig. 98. ^{13}C NMR of compound **4v**

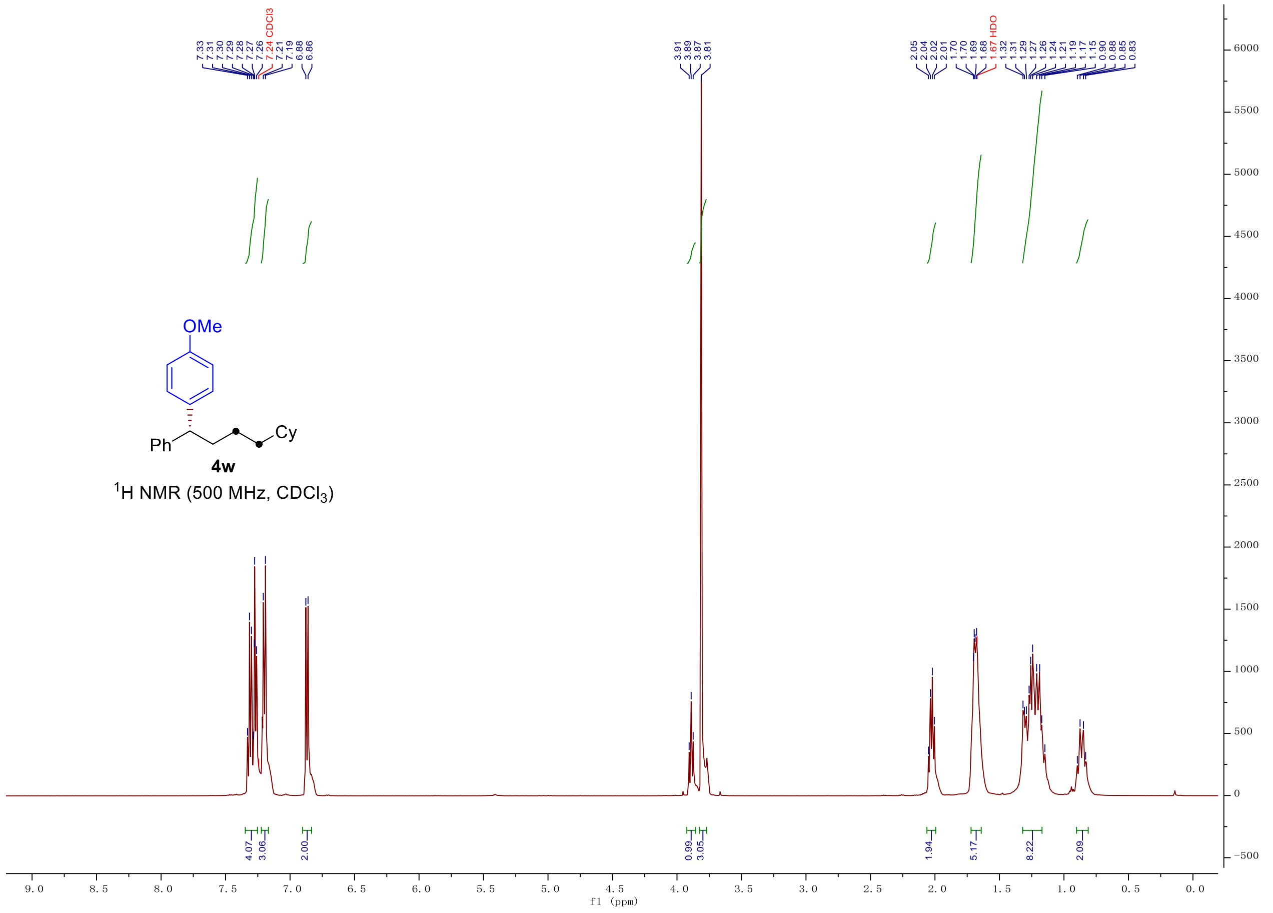

Supplementary Fig. 99. ^{1}H NMR of compound **4w**

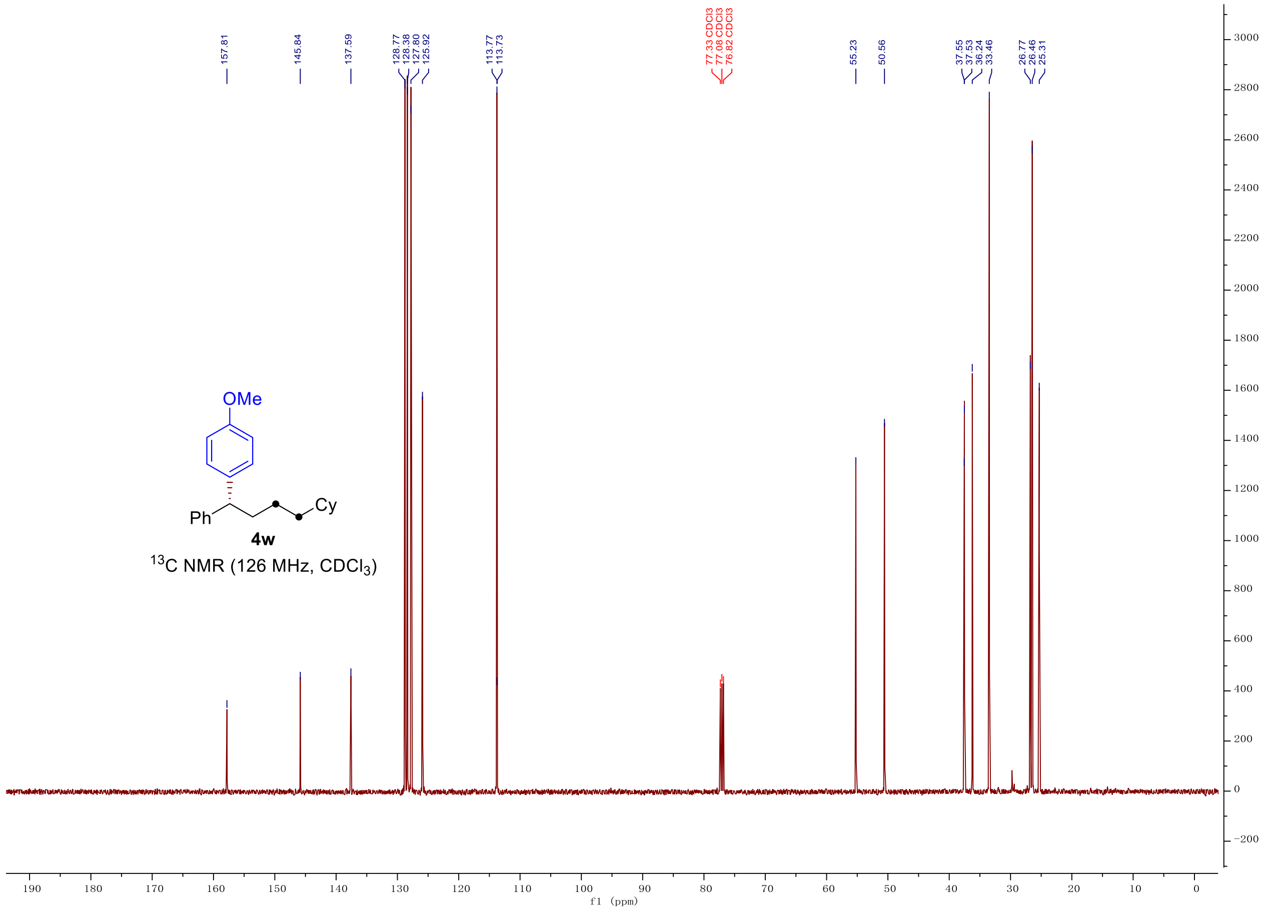

Supplementary Fig. 100. ^{13}C NMR of compound **4w**

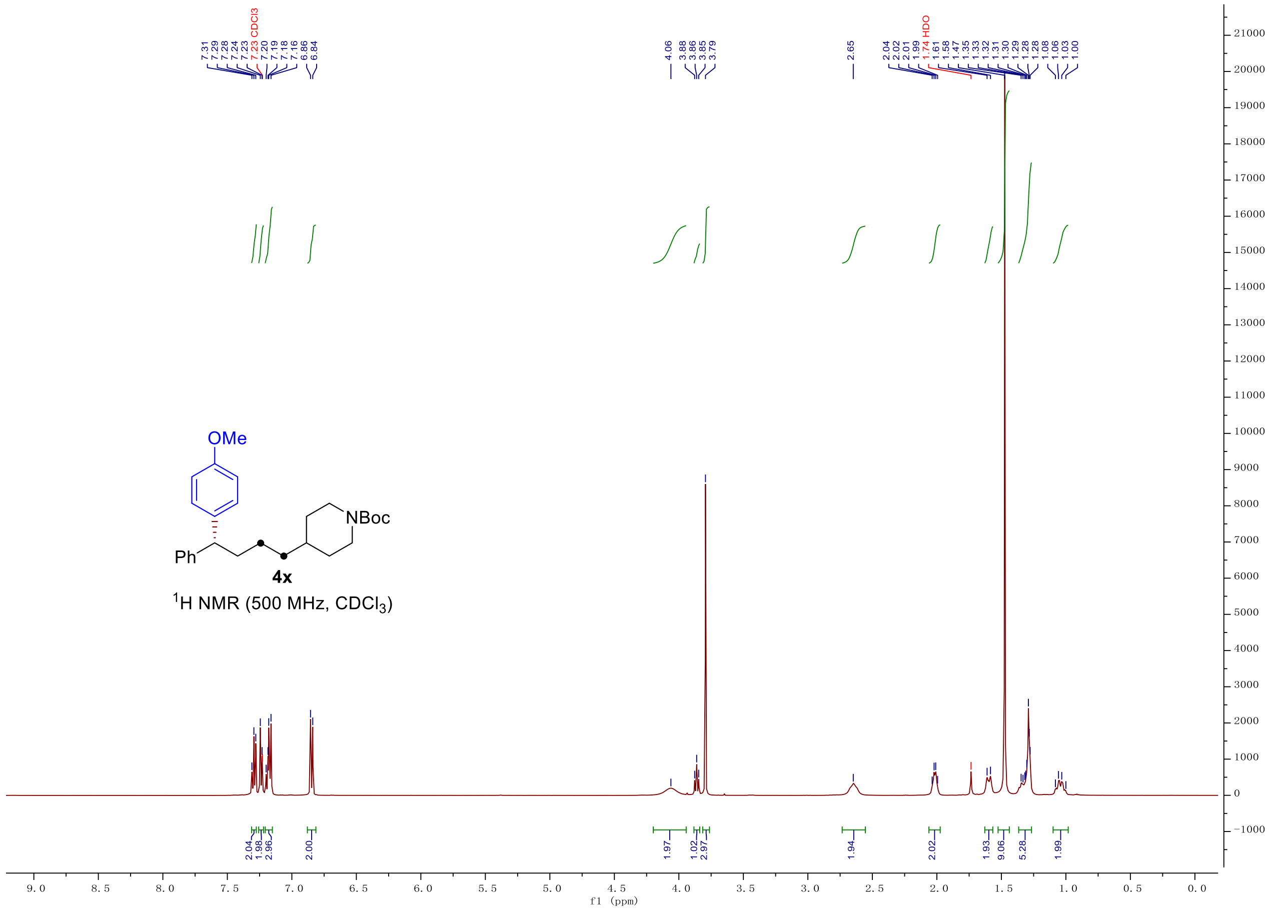

Supplementary Fig. 101. ^{1}H NMR of compound **4x**

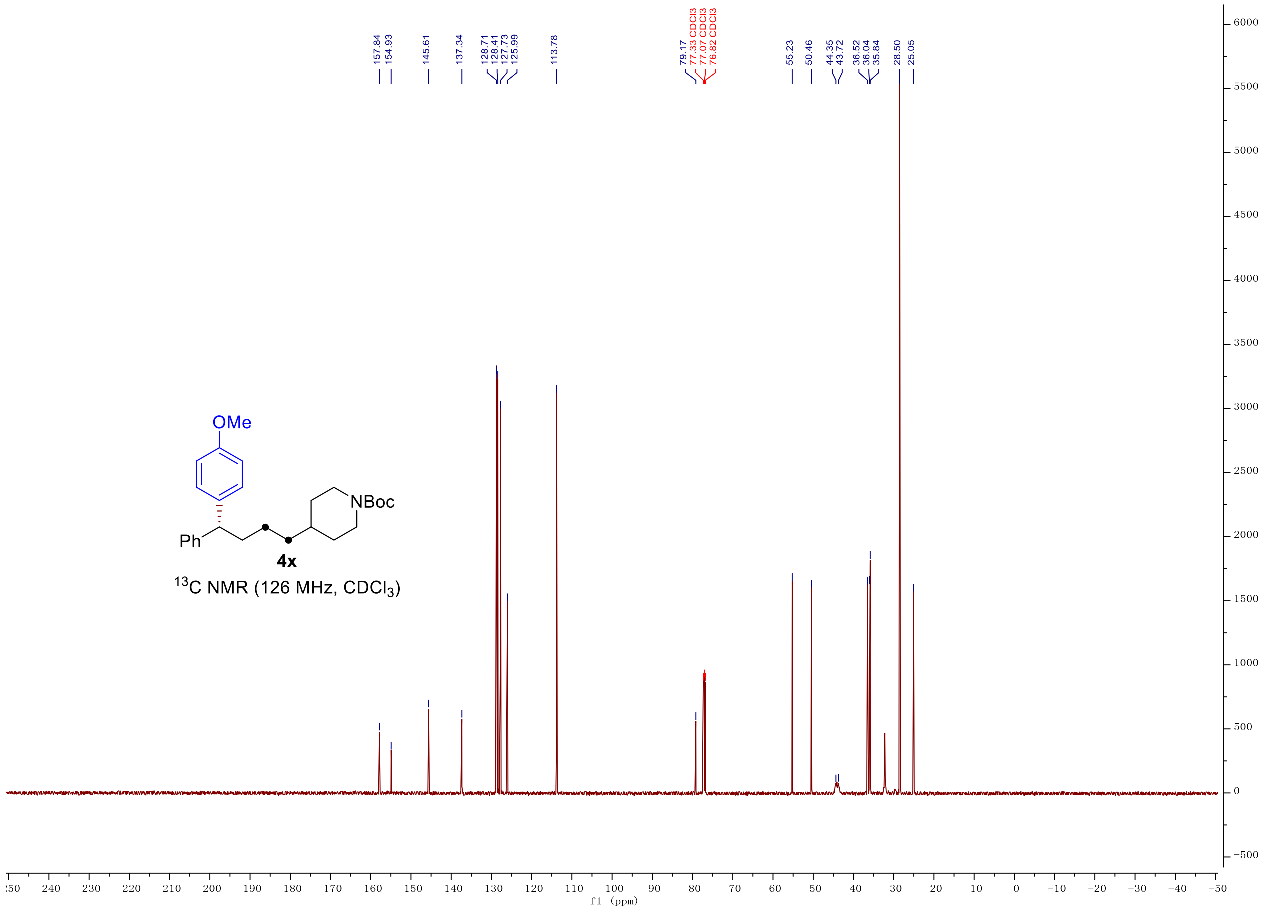

Supplementary Fig. 102. ^{13}C NMR of compound **4x**

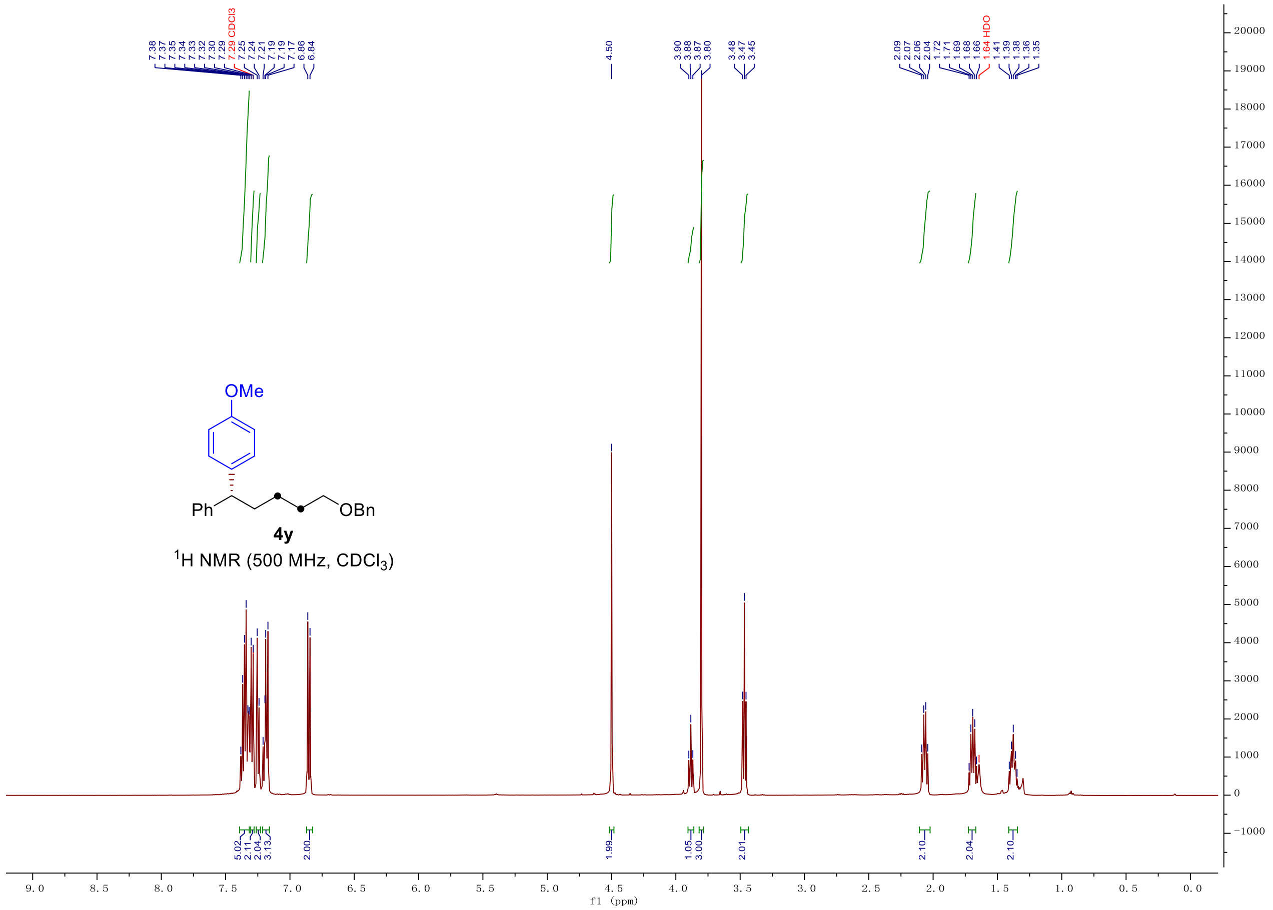

Supplementary Fig. 103. ^{1}H NMR of compound **4y**

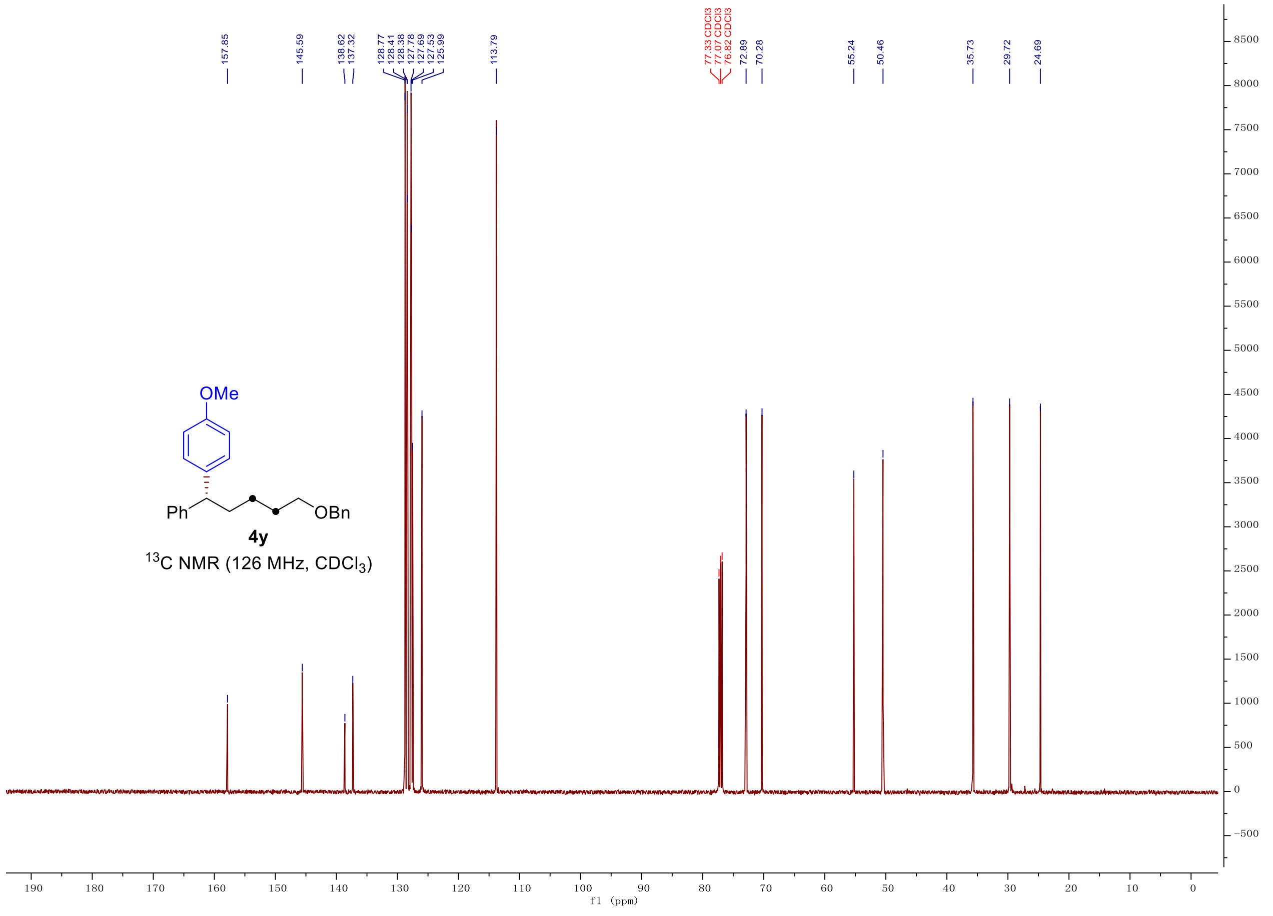

Supplementary Fig. 104. ^{13}C NMR of compound **4y**

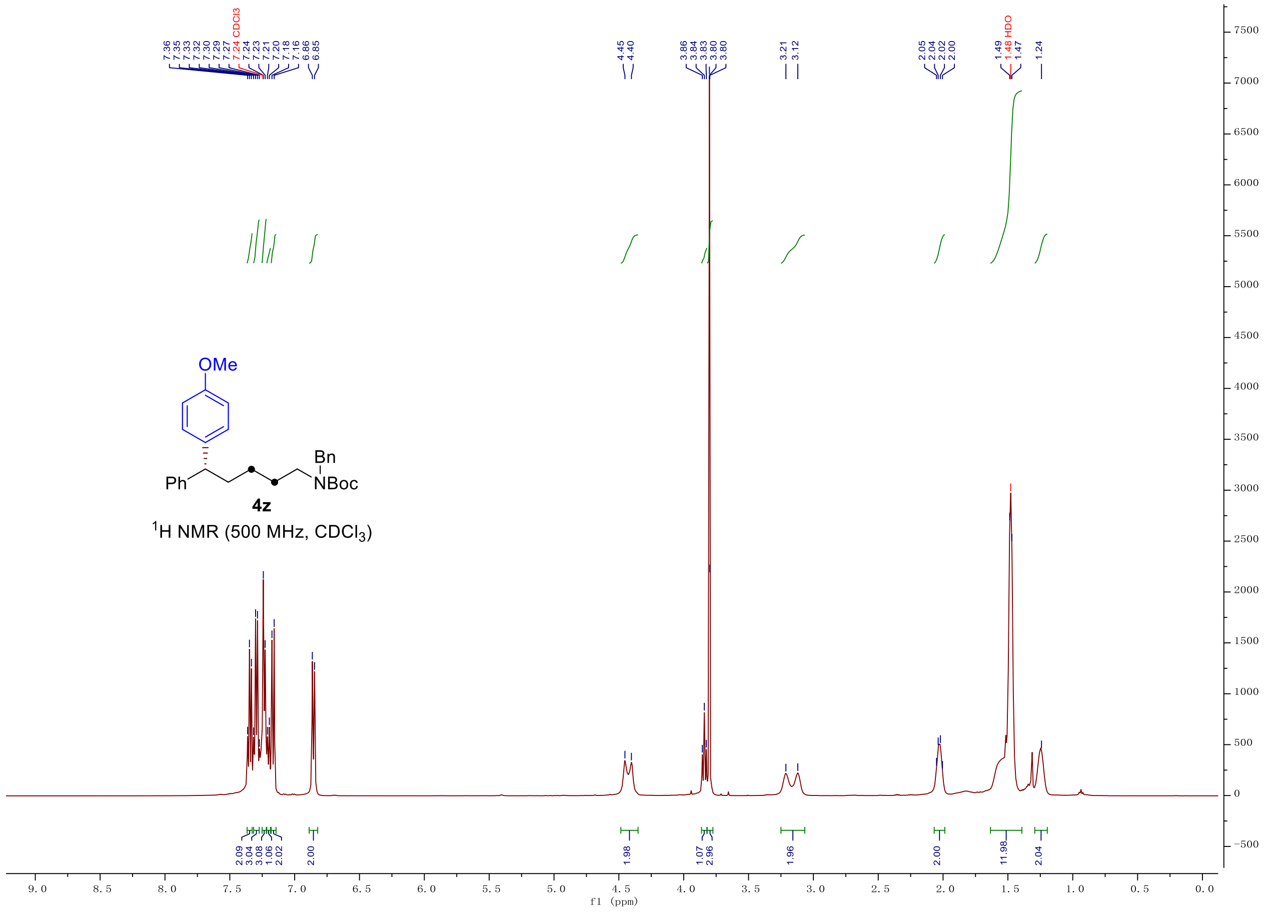

Supplementary Fig. 105. ^{1}H NMR of compound **4z**

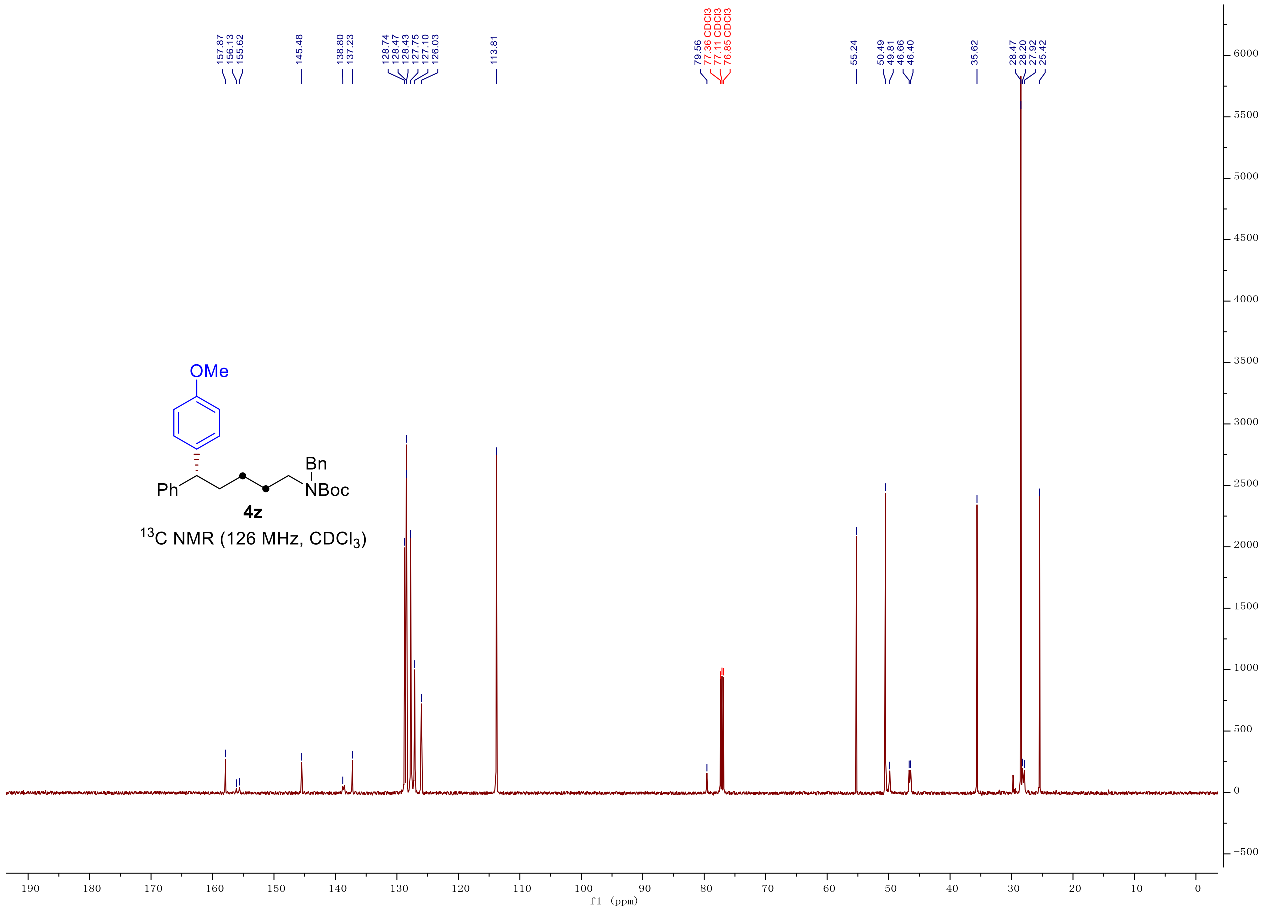

Supplementary Fig. 106. ^{13}C NMR of compound **4z**

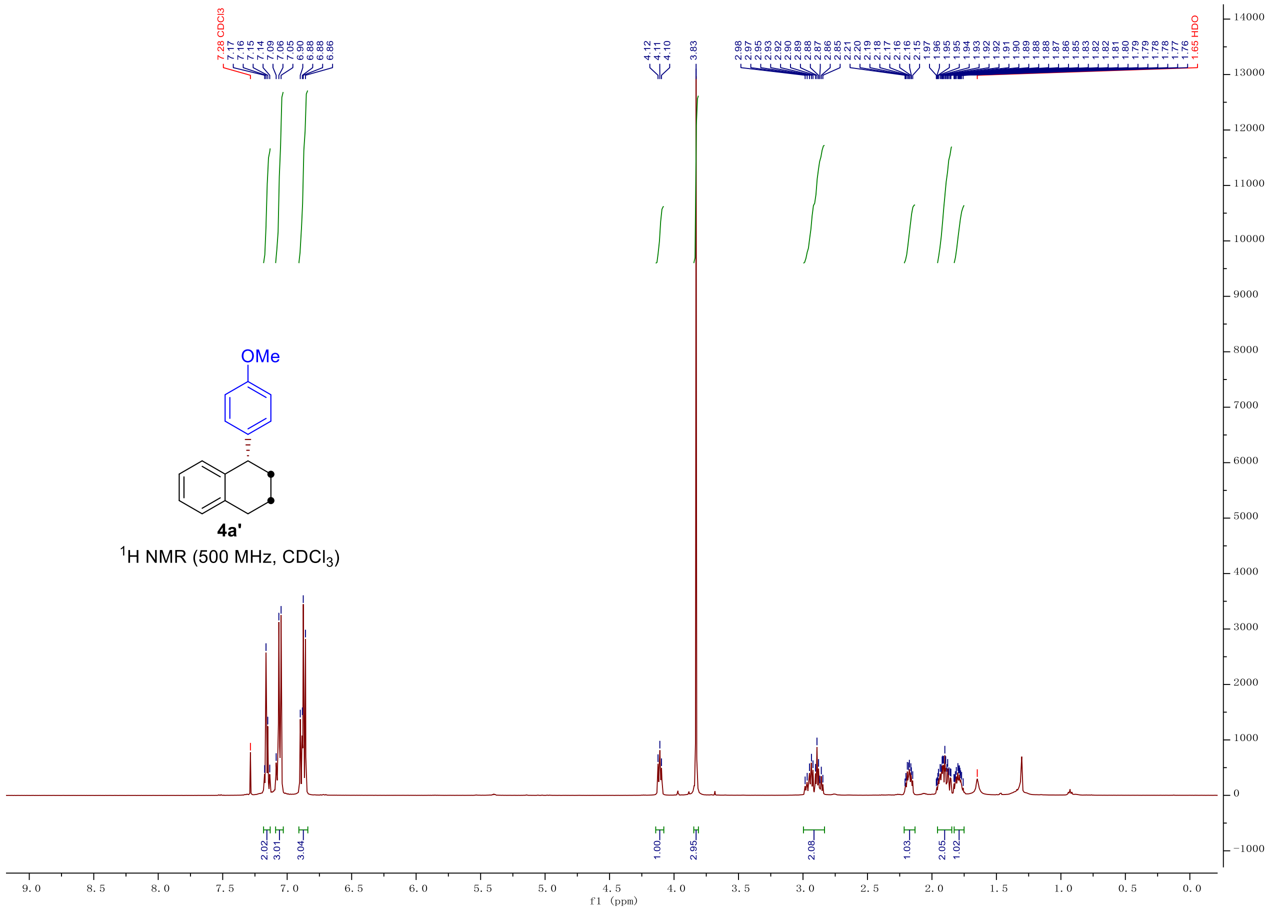

Supplementary Fig. 107. ^{1}H NMR of compound **4a**'

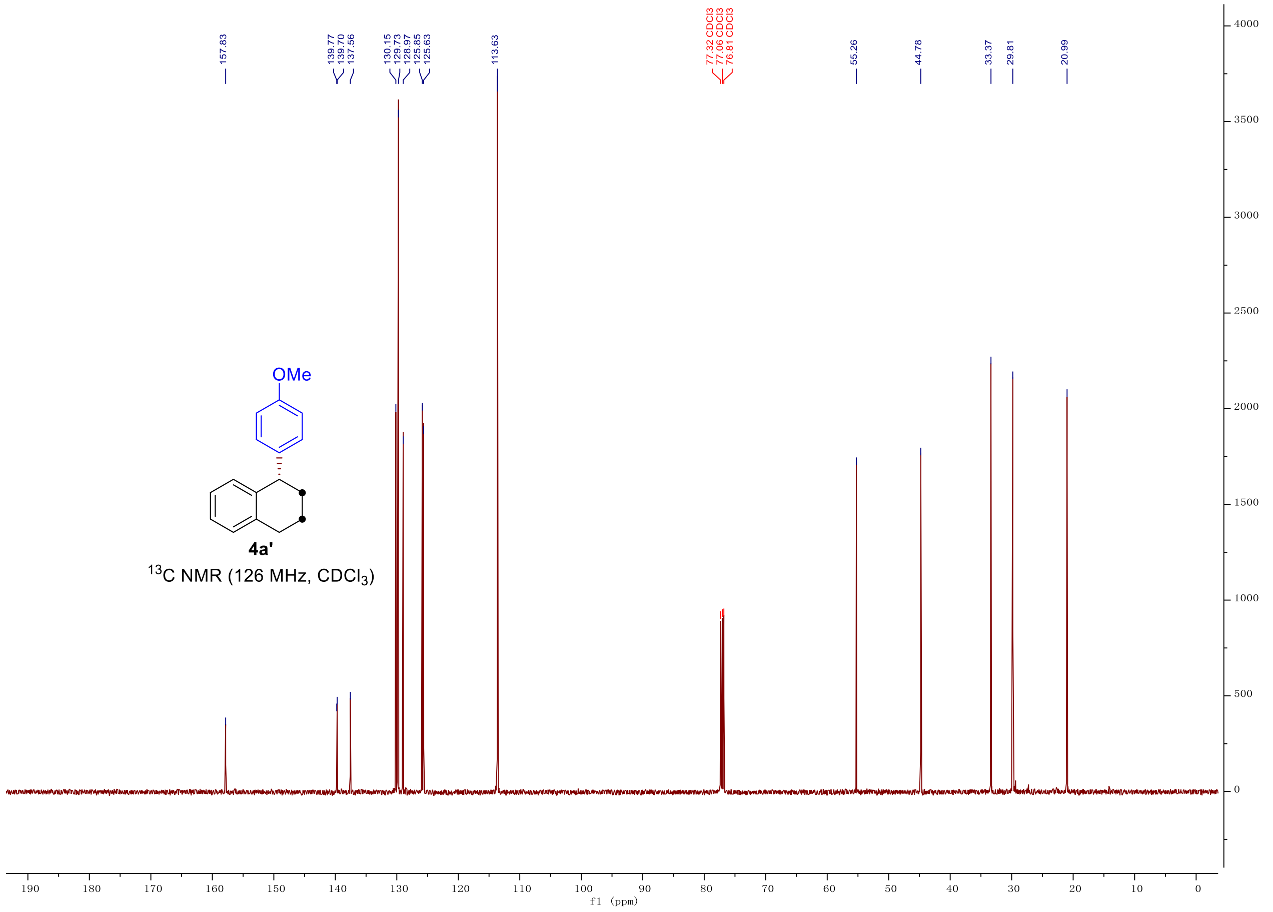

Supplementary Fig. 108. ^{13}C NMR of compound **4a'**

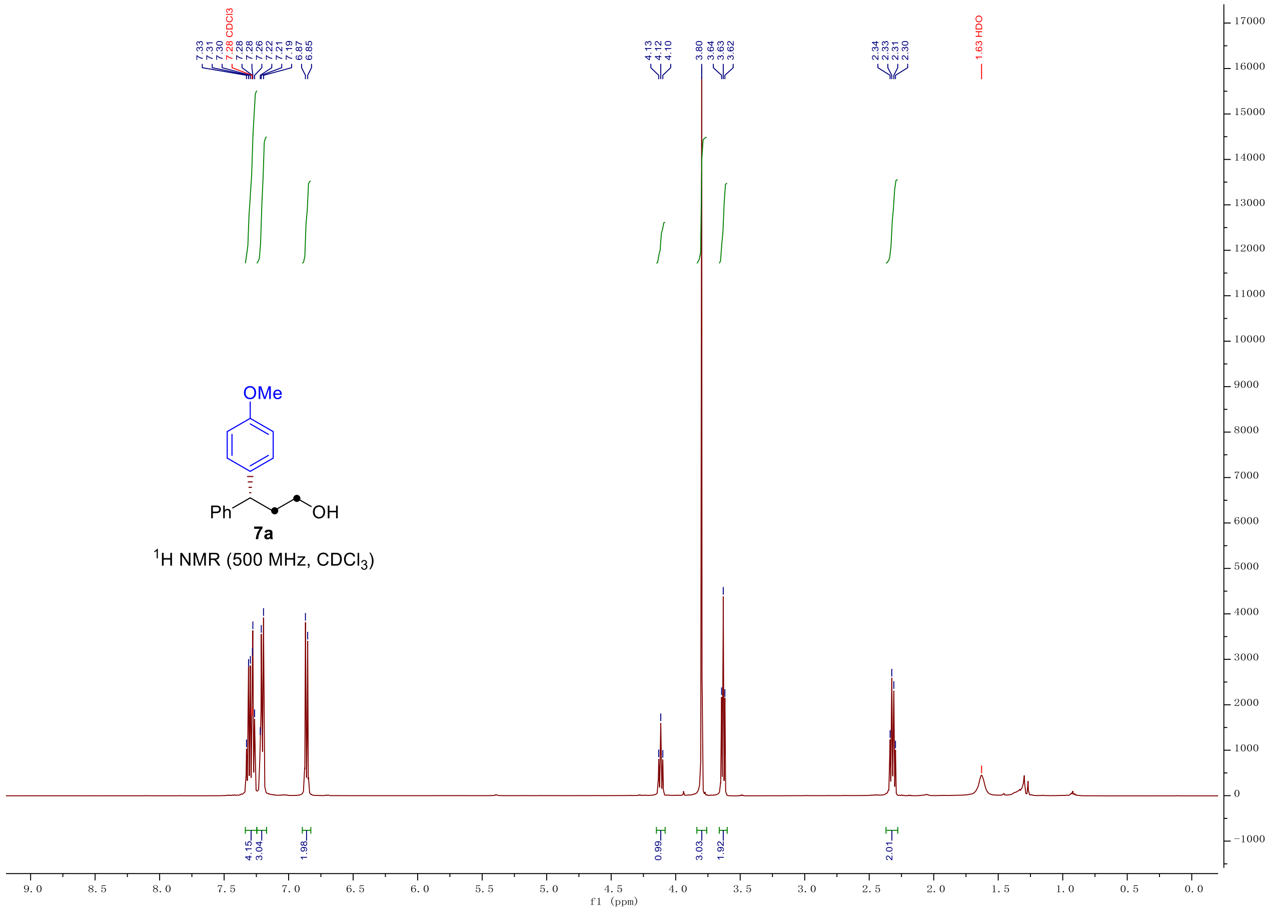

Supplementary Fig. 109. ^{1}H NMR of compound **7a**

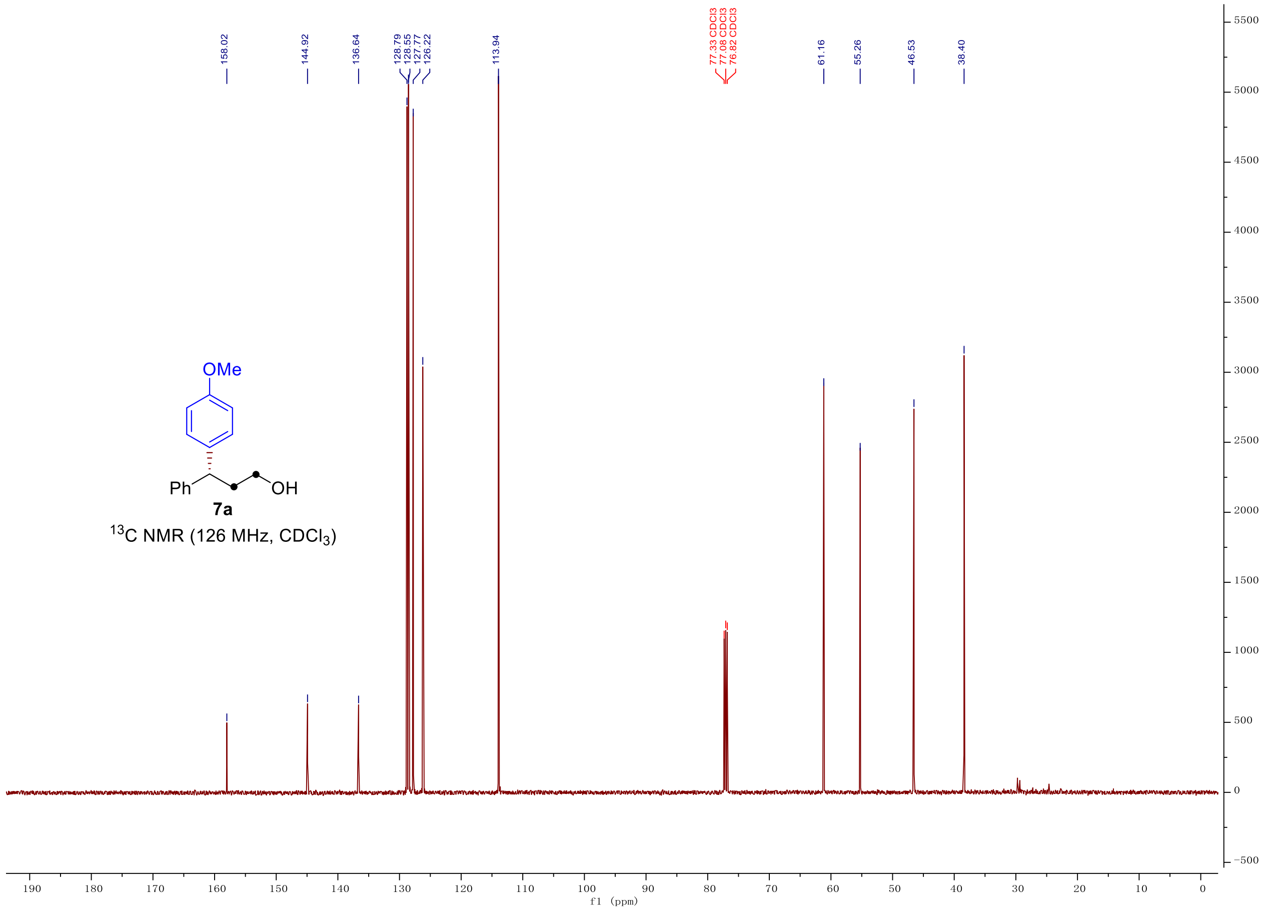

Supplementary Fig. 110. ^{13}C NMR of compound **7a**

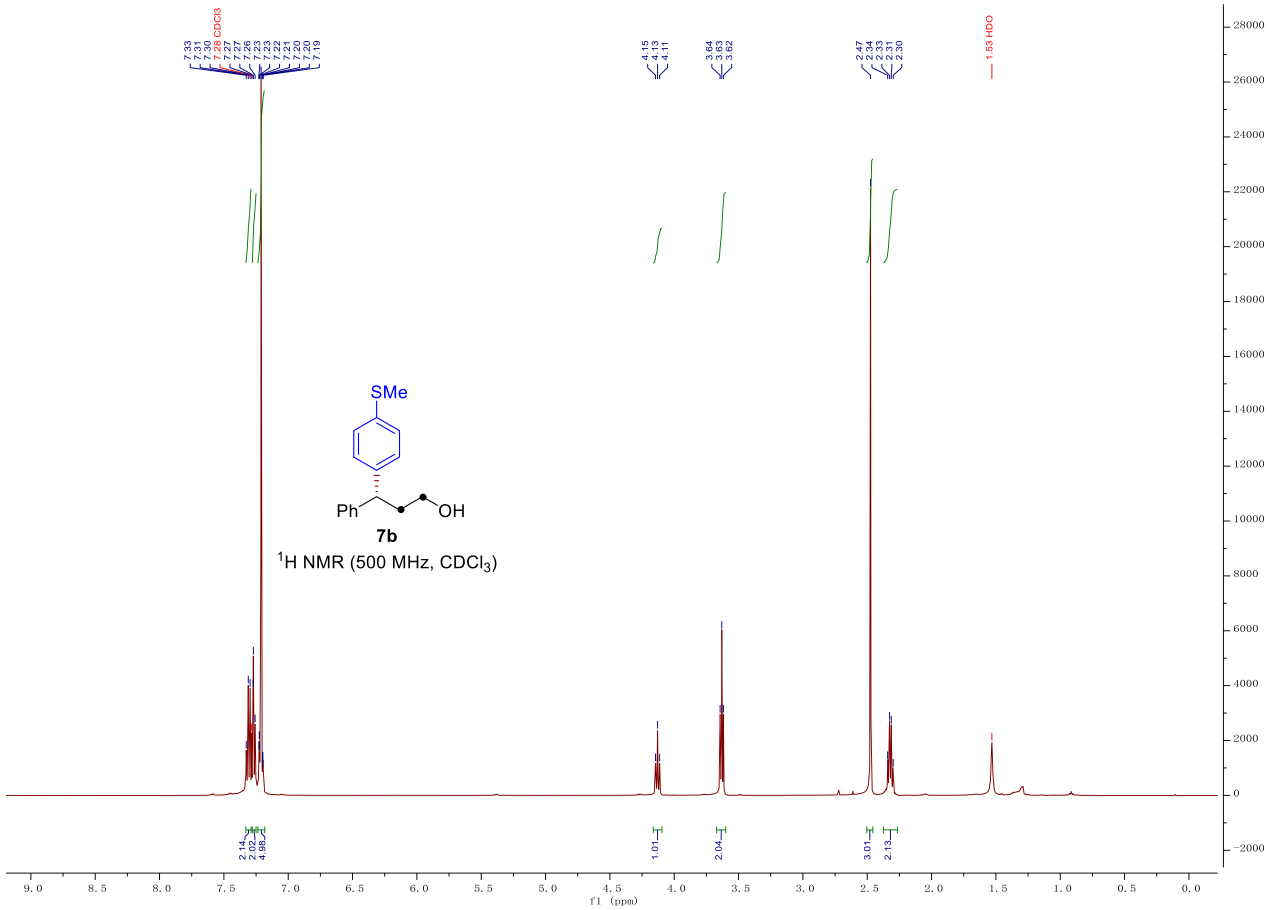

Supplementary Fig. 111. ^{1}H NMR of compound **7b**

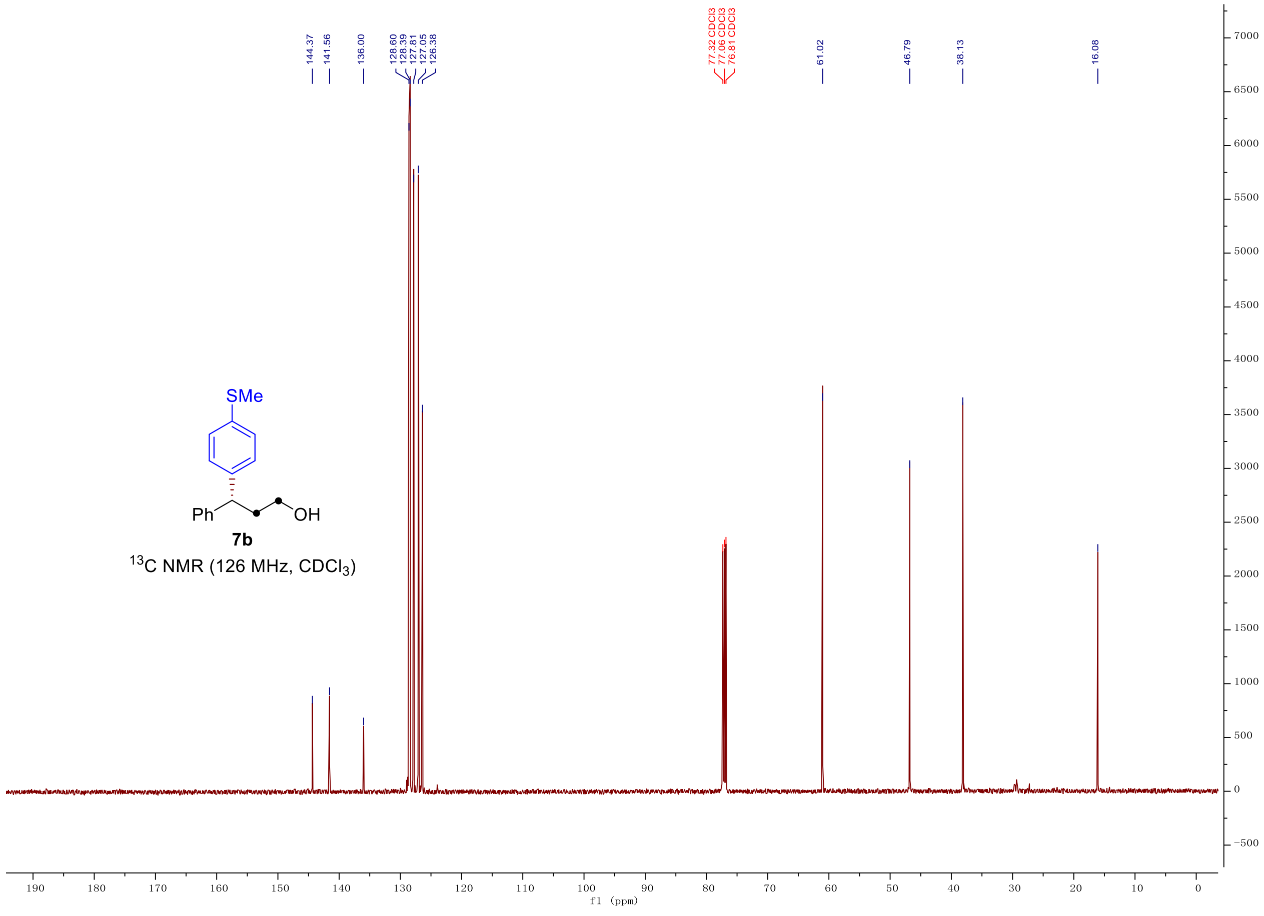

Supplementary Fig. 112. ^{13}C NMR of compound **7b**

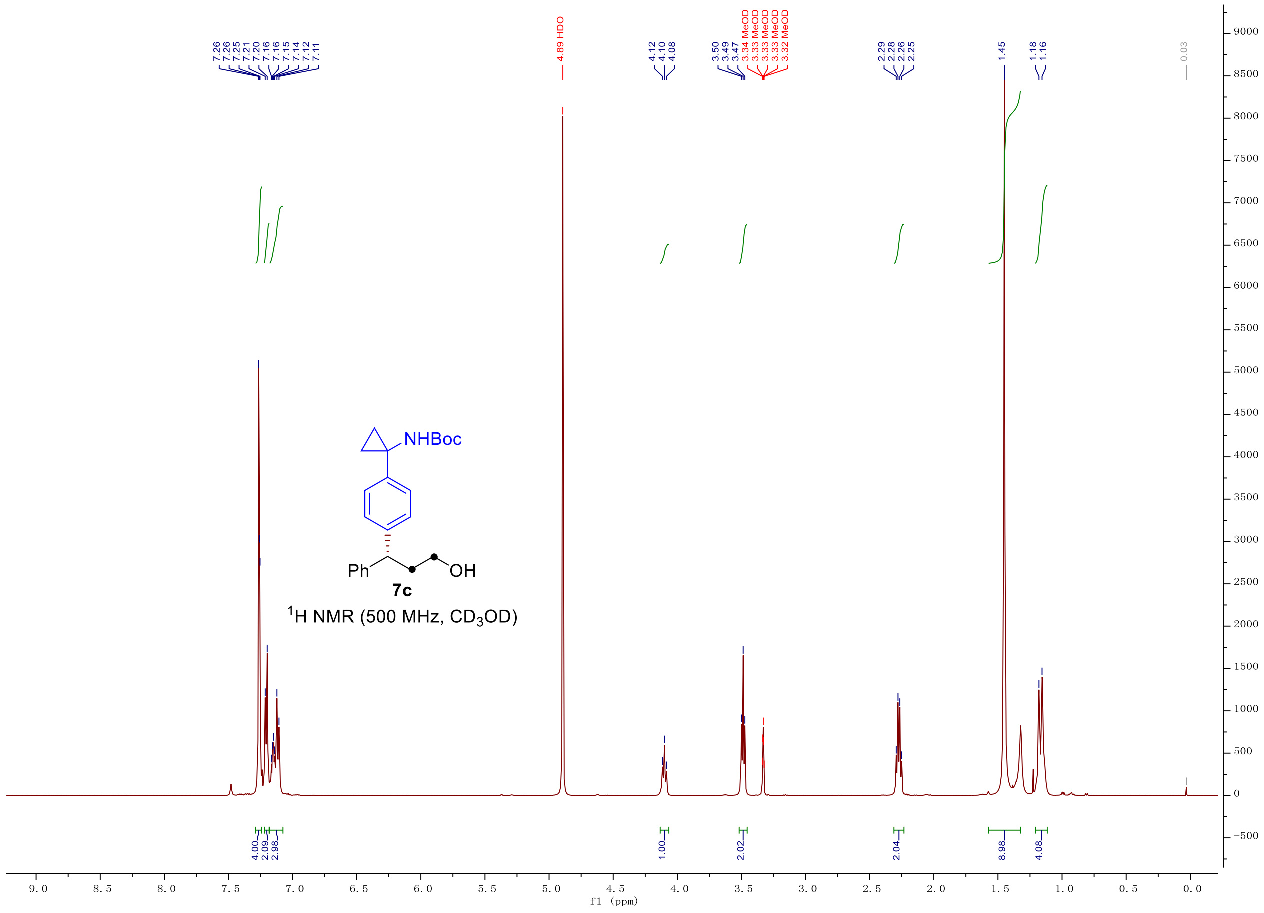

Supplementary Fig. 113. ^{1}H NMR of compound **7c**

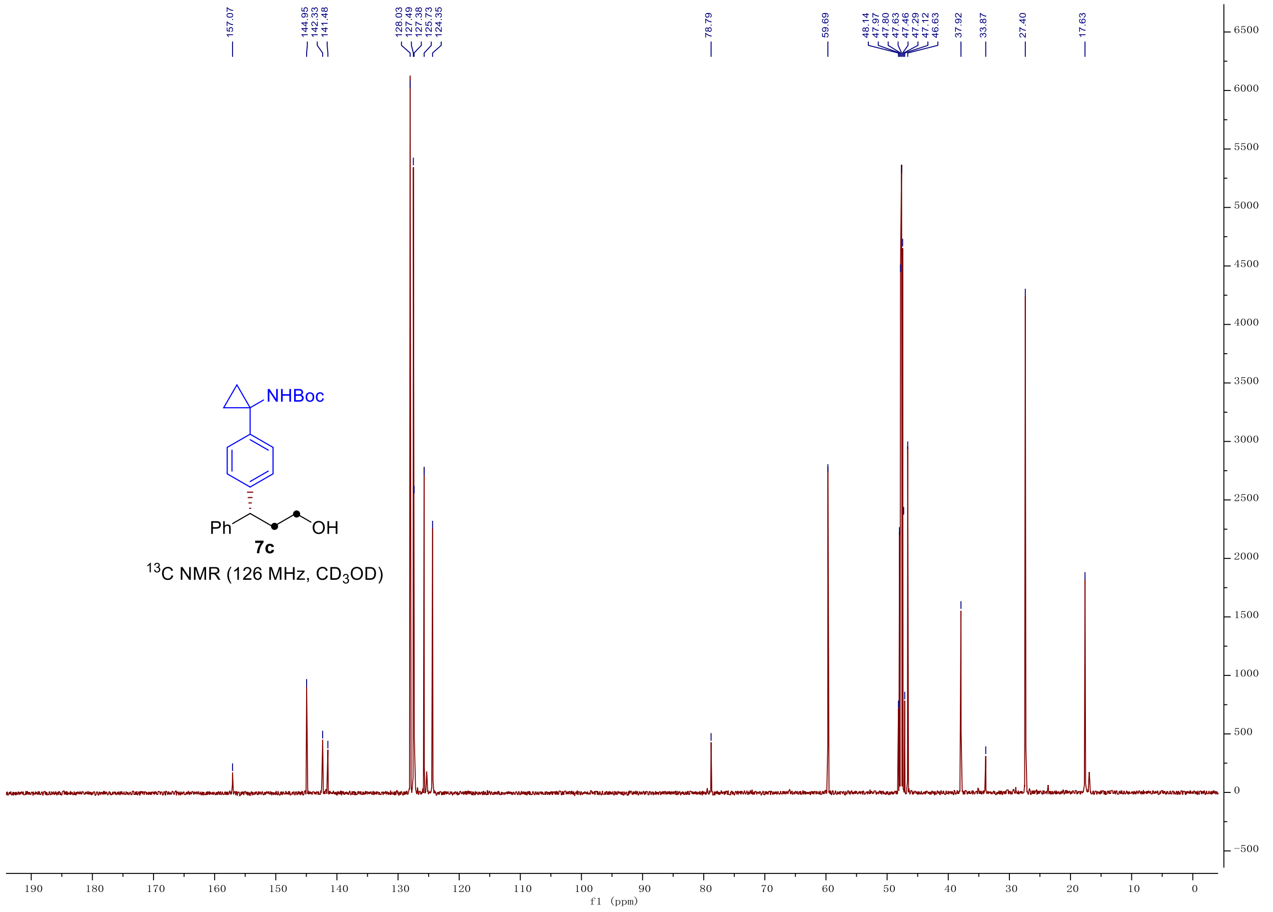

Supplementary Fig. 114. ^{13}C NMR of compound **7c**

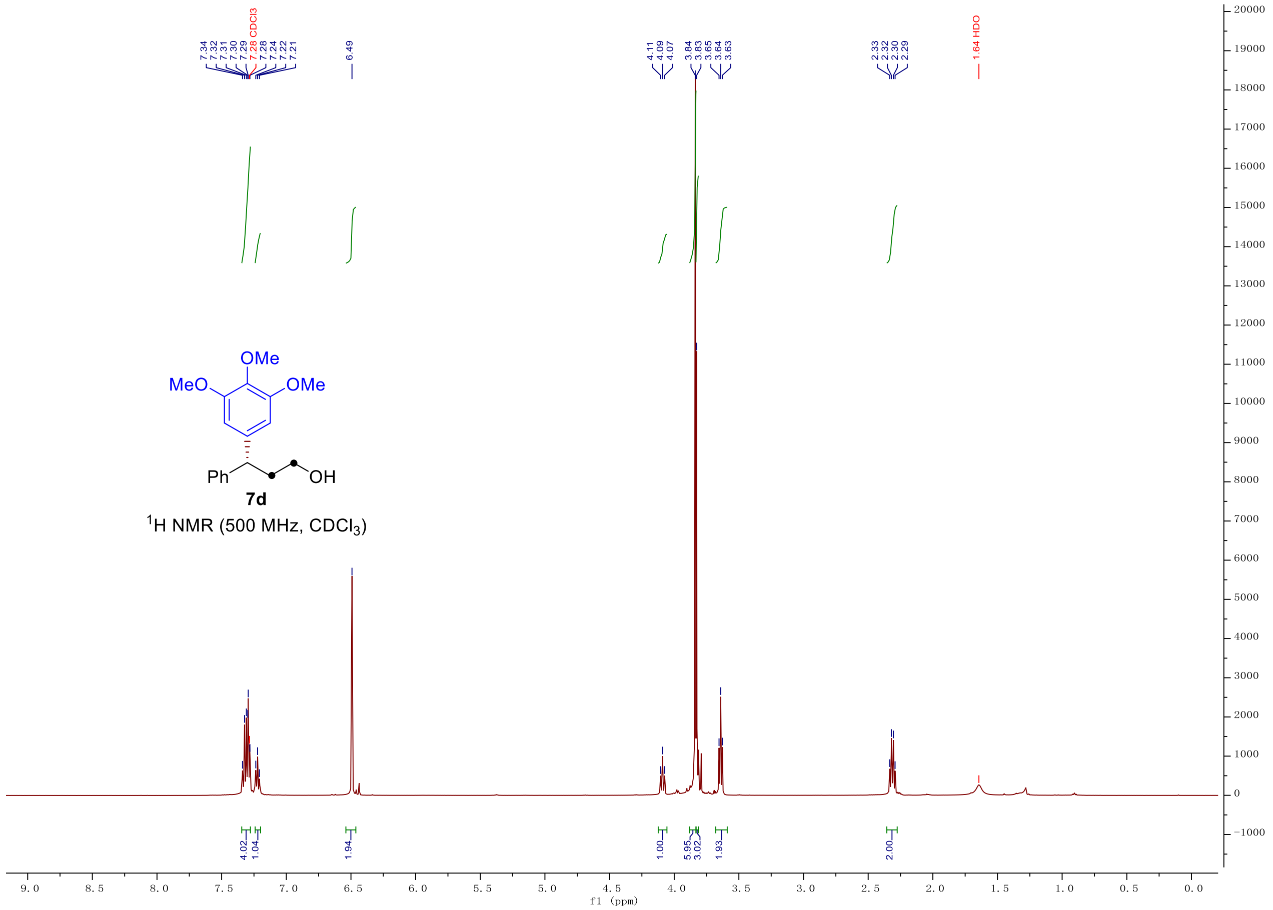

Supplementary Fig. 115. ^{1}H NMR of compound **7d**

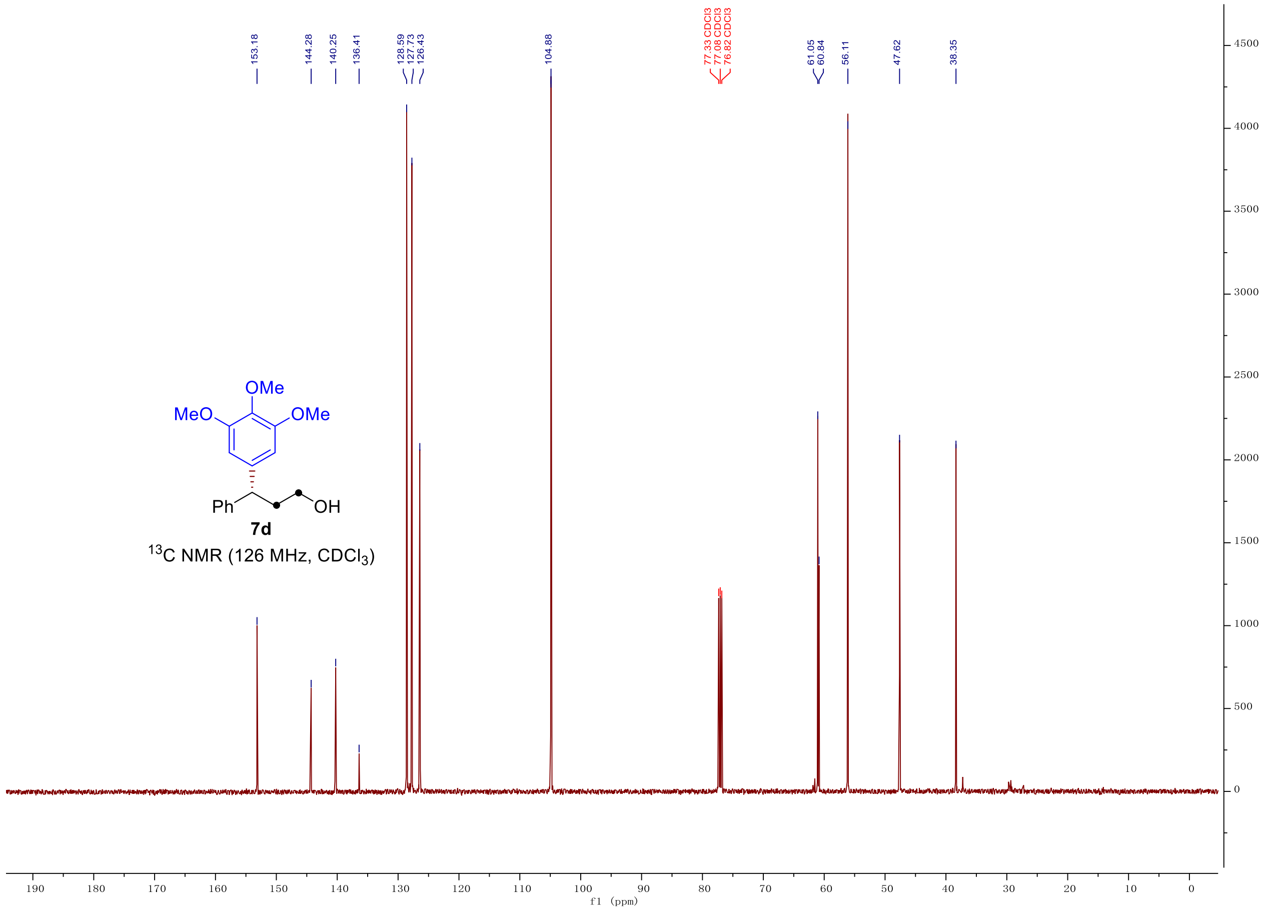

Supplementary Fig. 116. ^{13}C NMR of compound **7d**

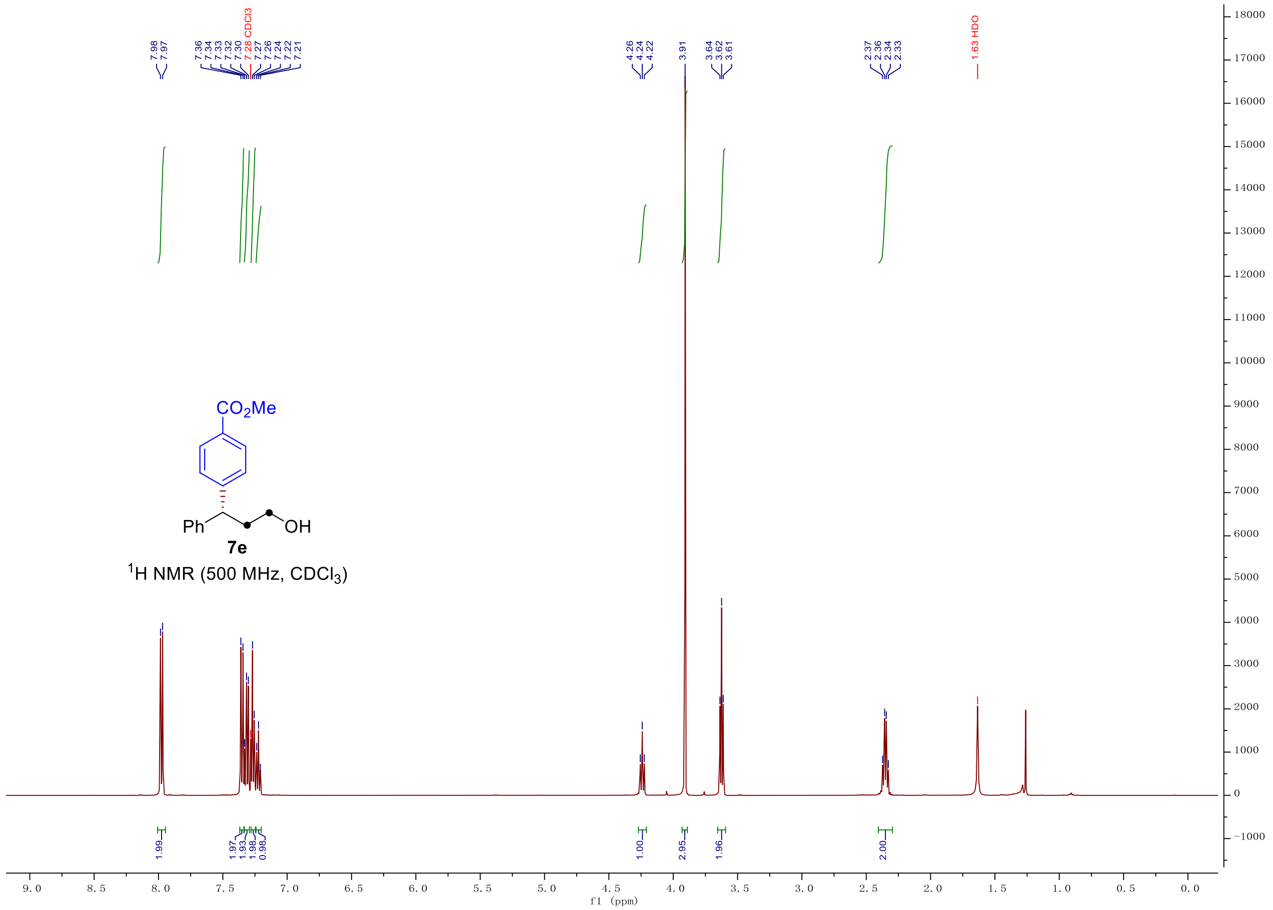

Supplementary Fig. 117. ^{1}H NMR of compound **7e**

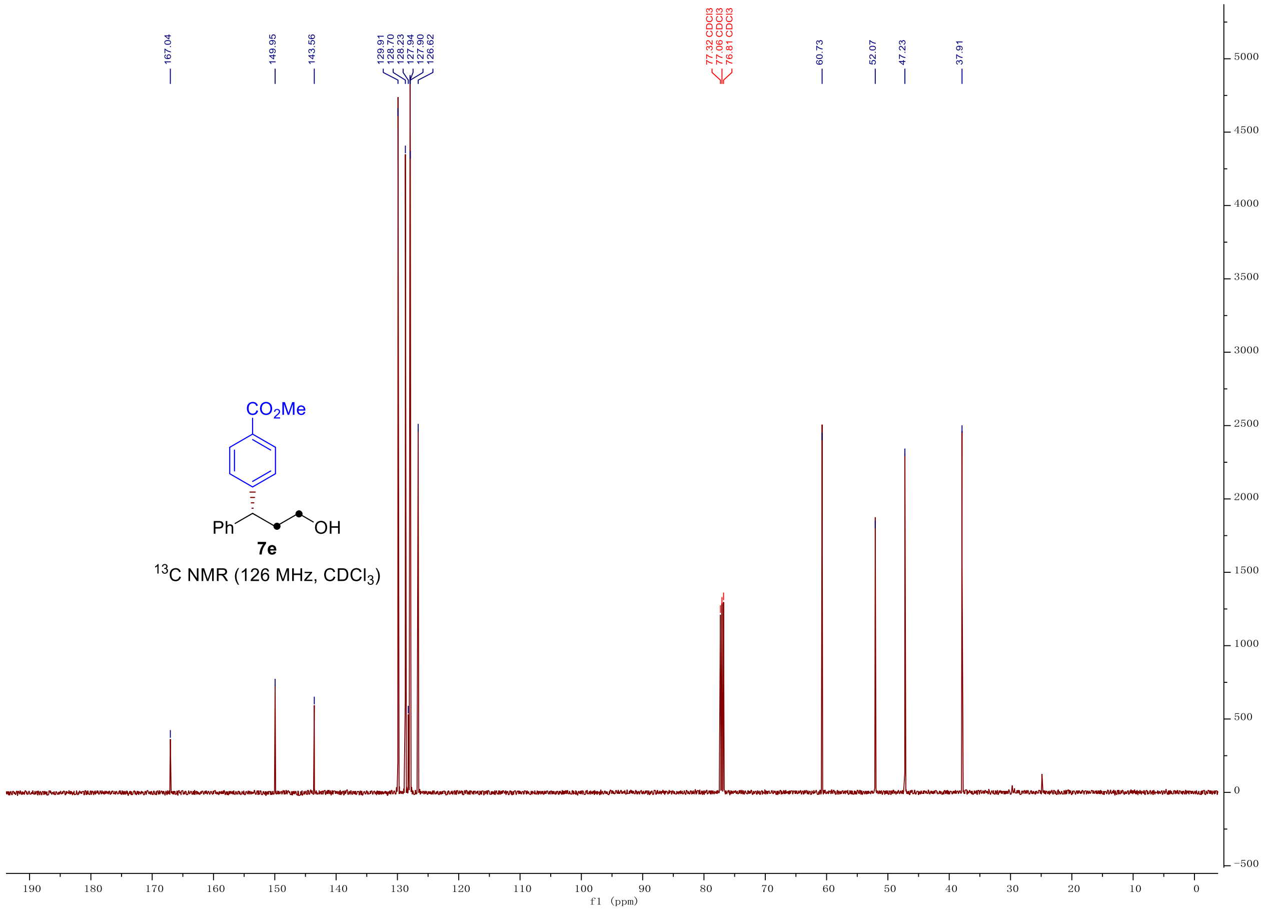

Supplementary Fig. 118. ^{13}C NMR of compound **7e**

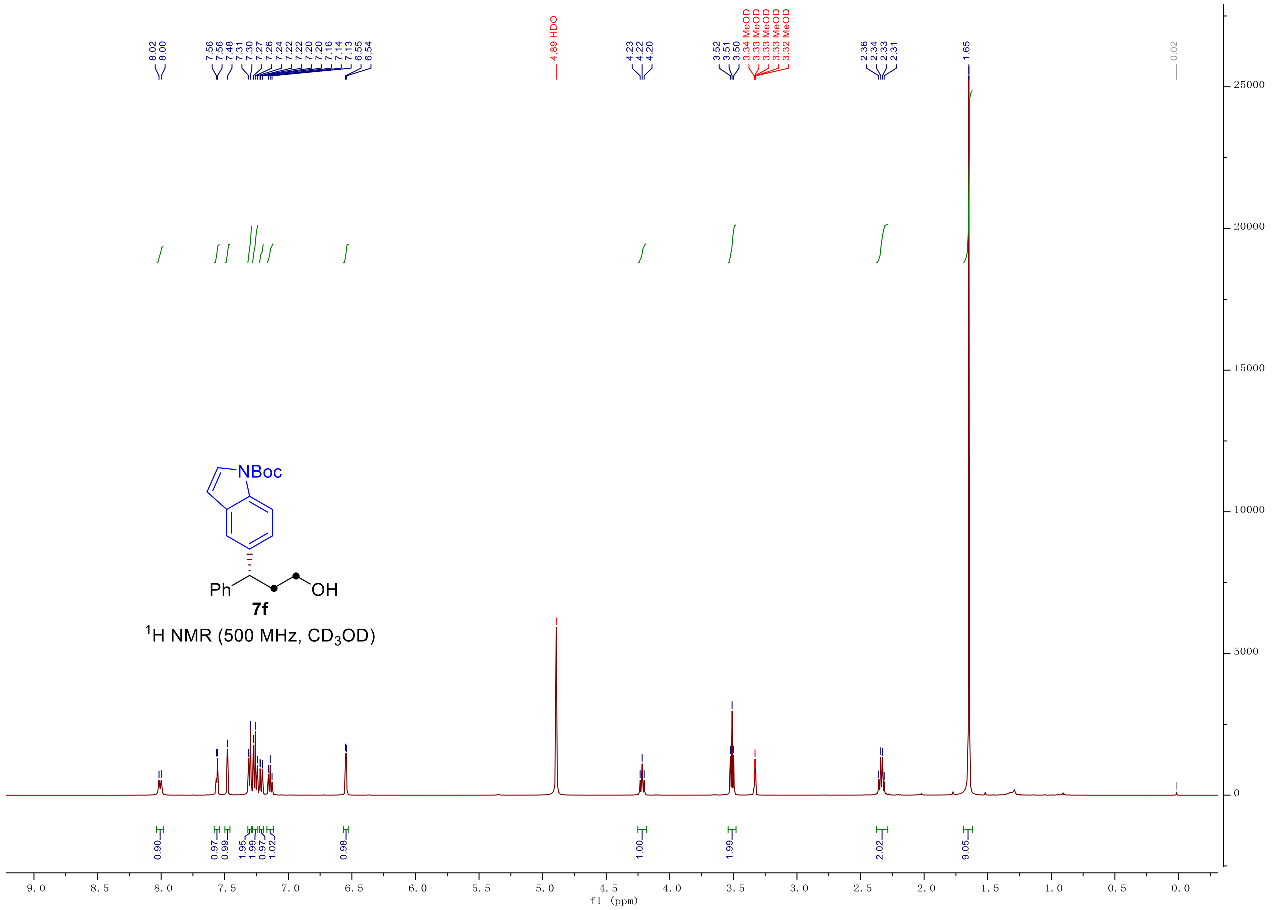

Supplementary Fig. 119. ^{1}H NMR of compound **7f**

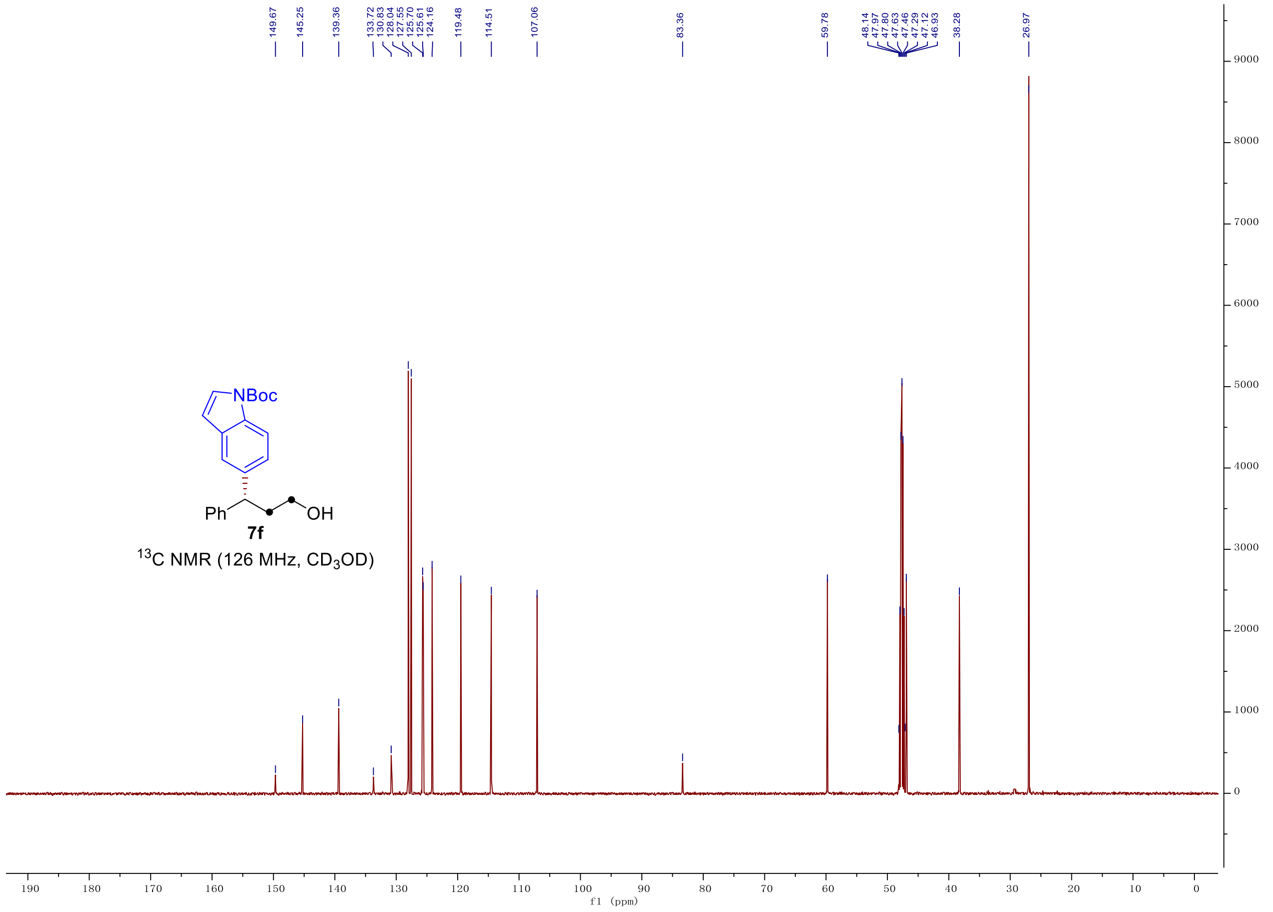

Supplementary Fig. 120. ^{13}C NMR of compound **7f**

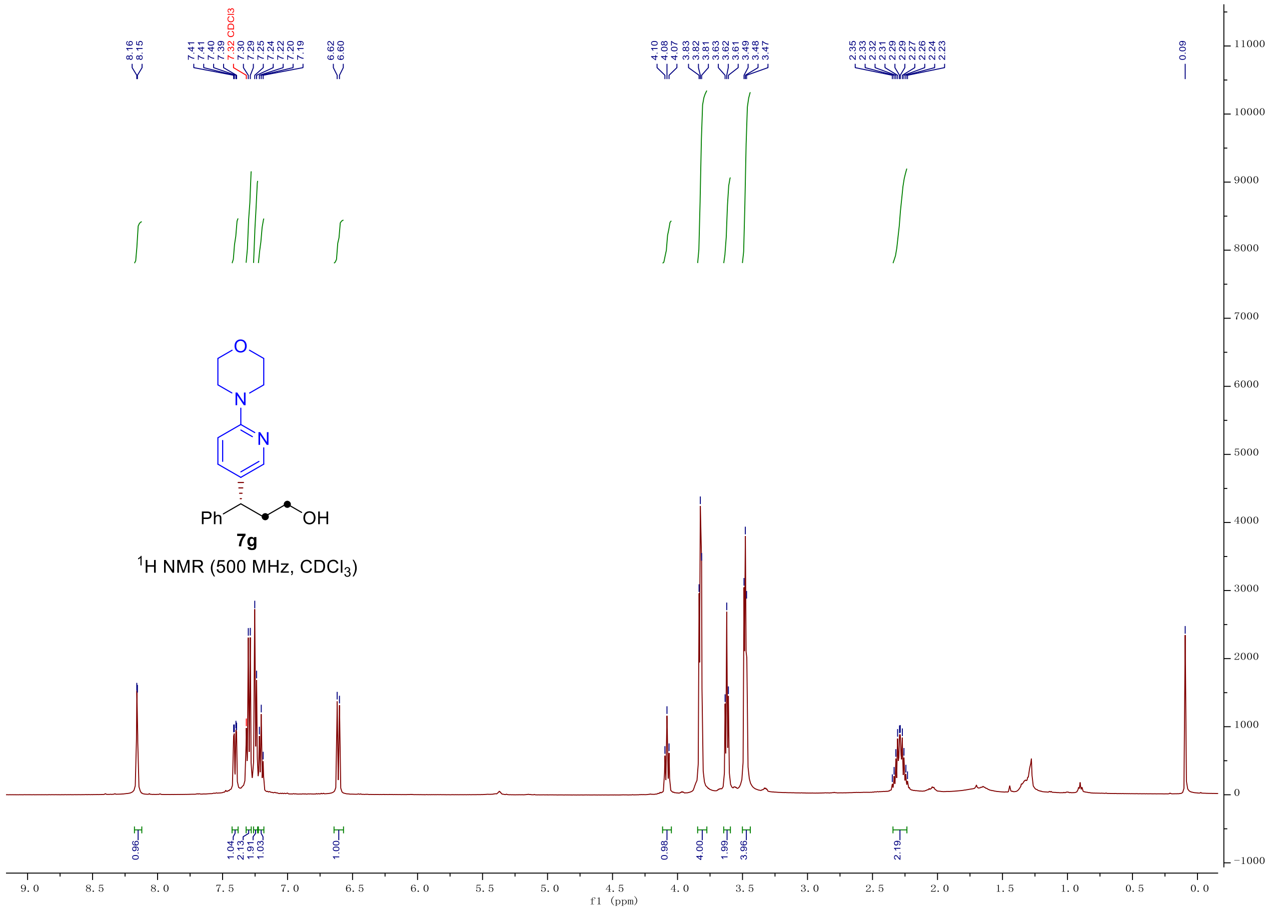

Supplementary Fig. 121. ^{1}H NMR of compound **7g**

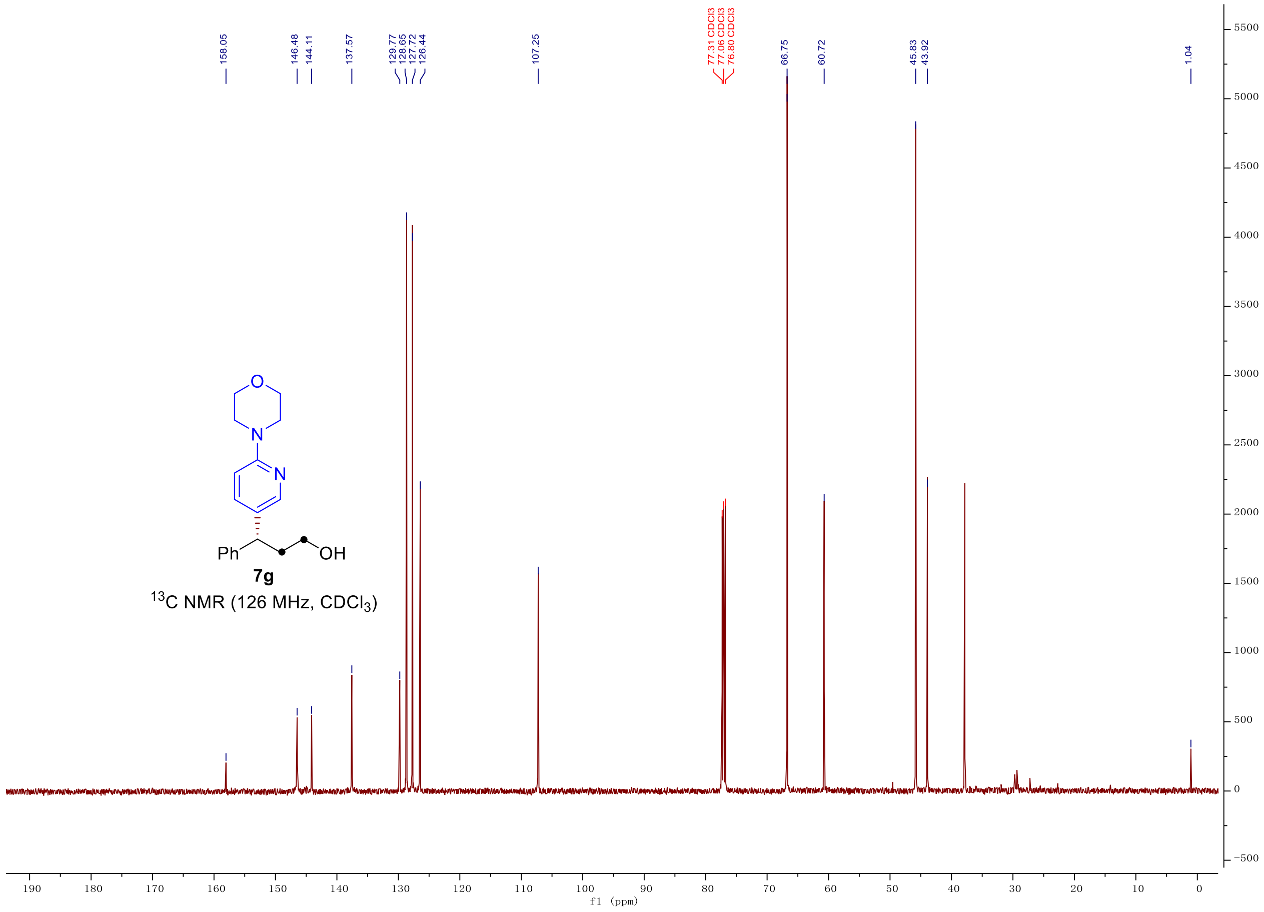

Supplementary Fig. 122. ^{13}C NMR of compound **7g**

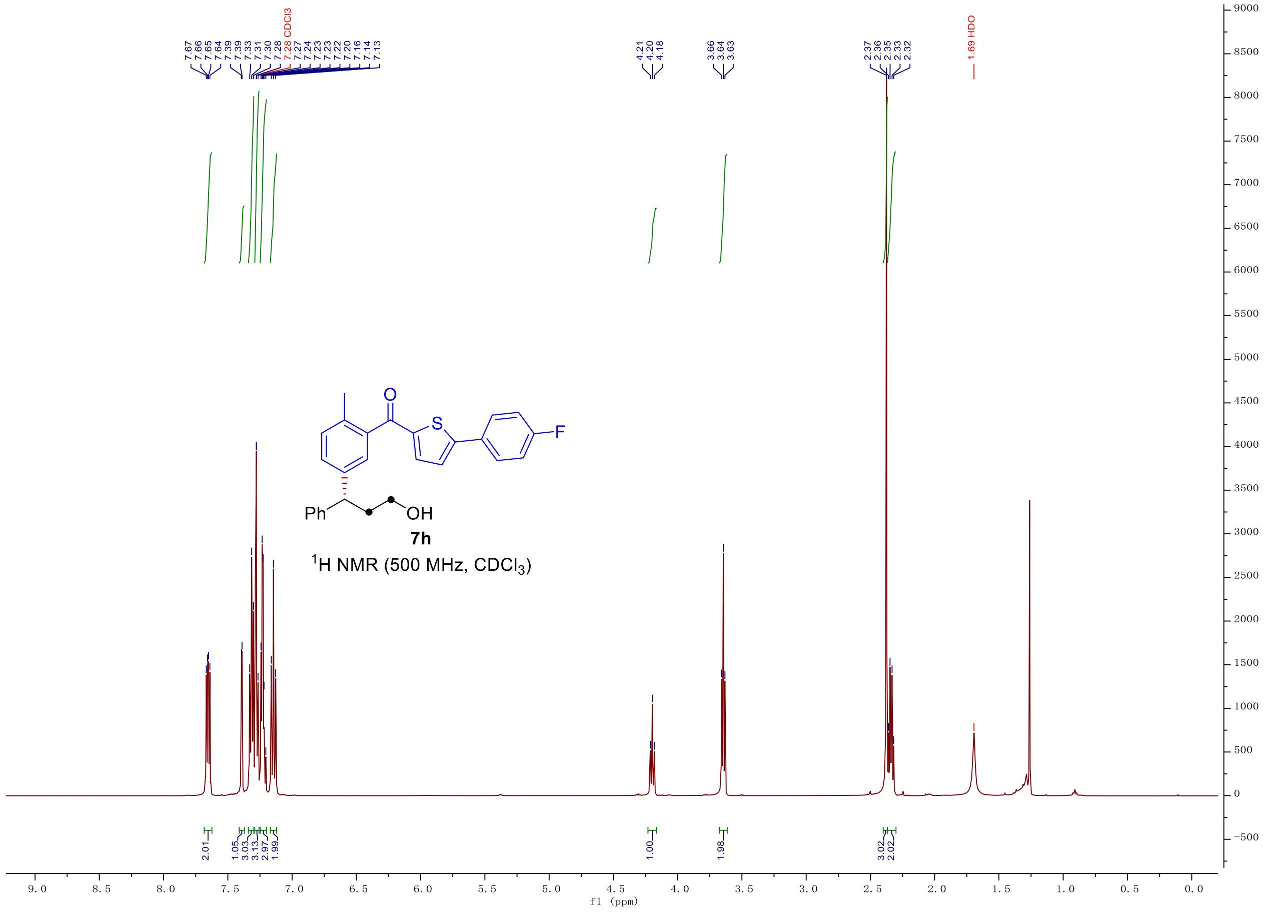

Supplementary Fig. 123. ^{1}H NMR of compound **7h**

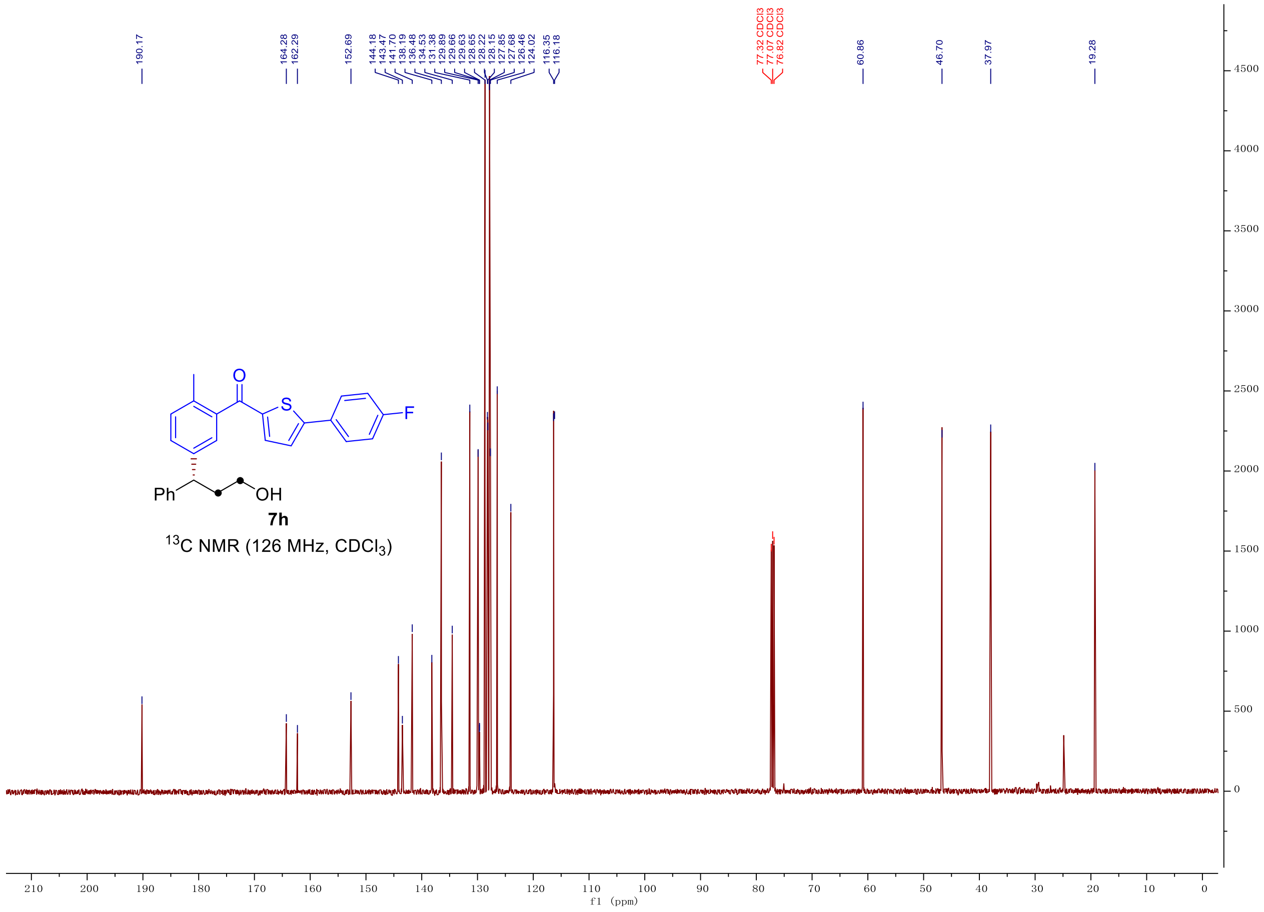

Supplementary Fig. 124. ^{13}C NMR of compound **7h**

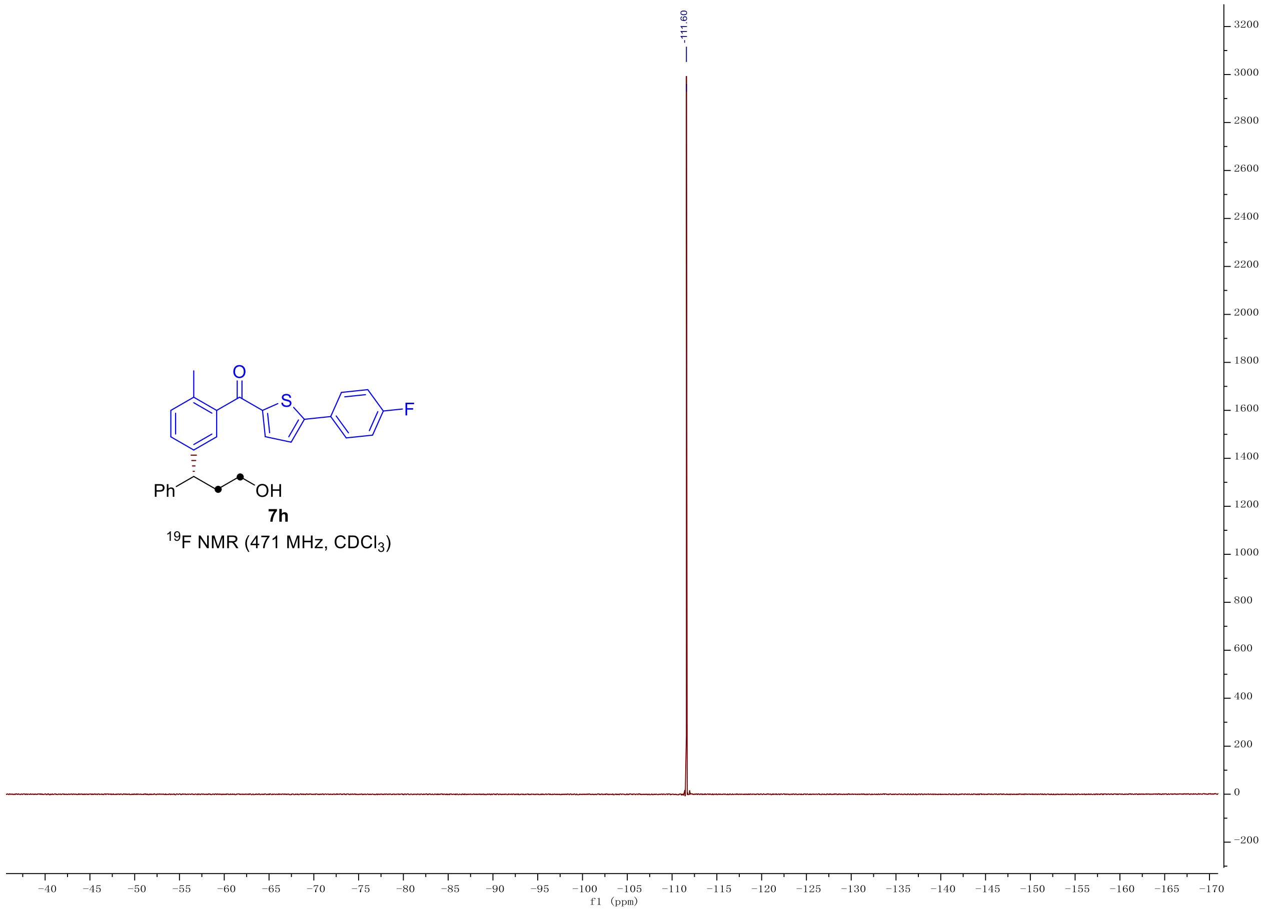

Supplementary Fig. 125. ^{19}F NMR of compound **7h**

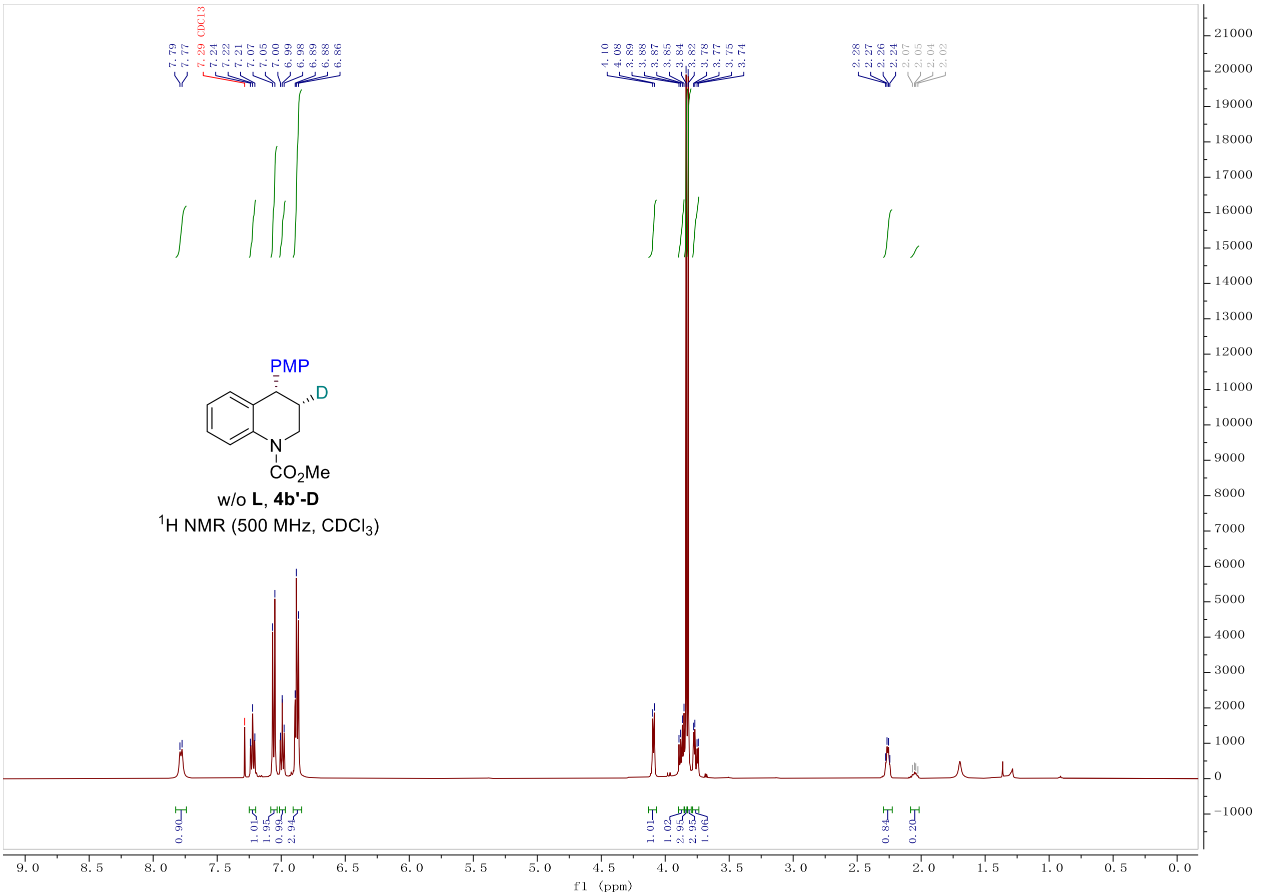

Supplementary Fig. 126. ^{1}H NMR of compound **4b'-D** (w/o **L**)

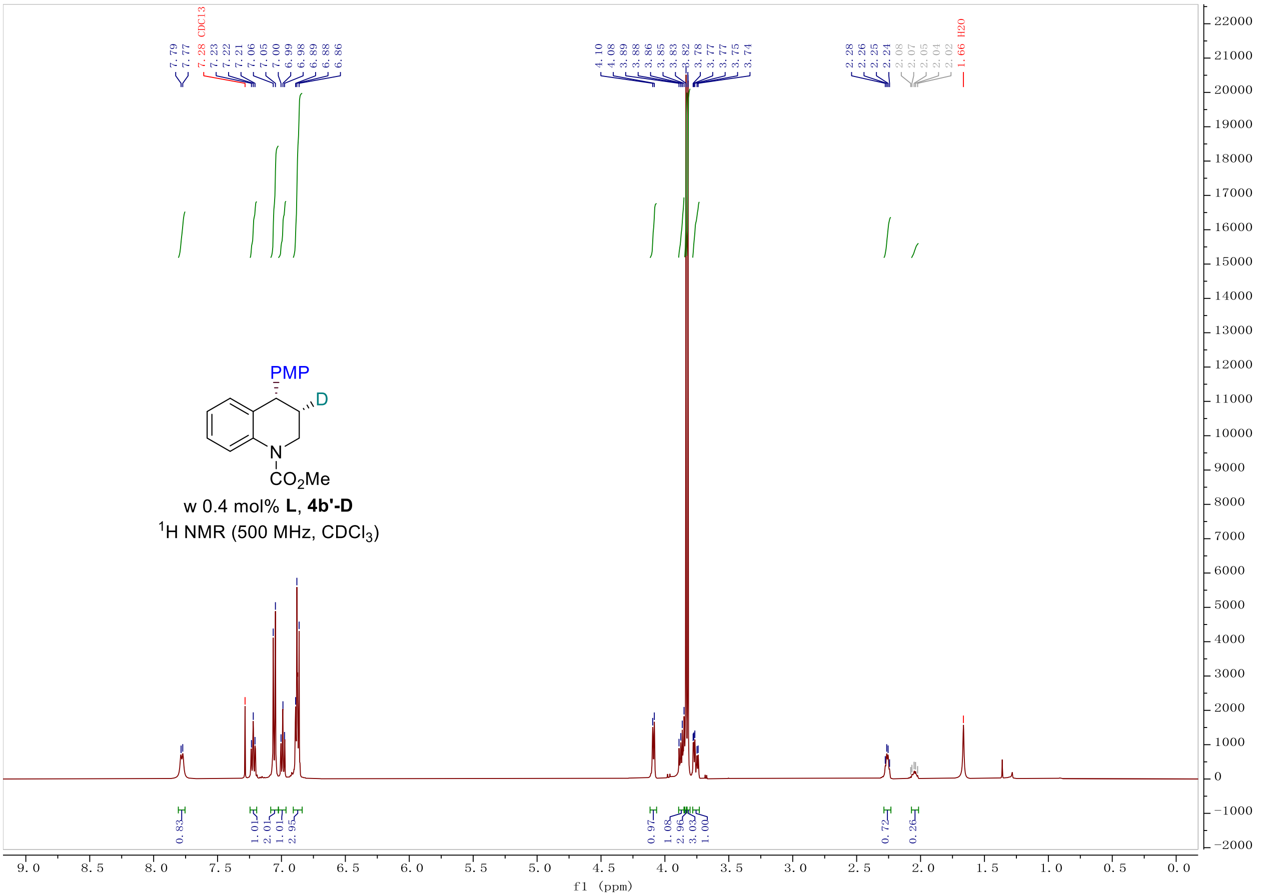

Supplementary Fig. 127. ^{1}H NMR of compound **4b'-D** (w 0.4 mol% **L**)

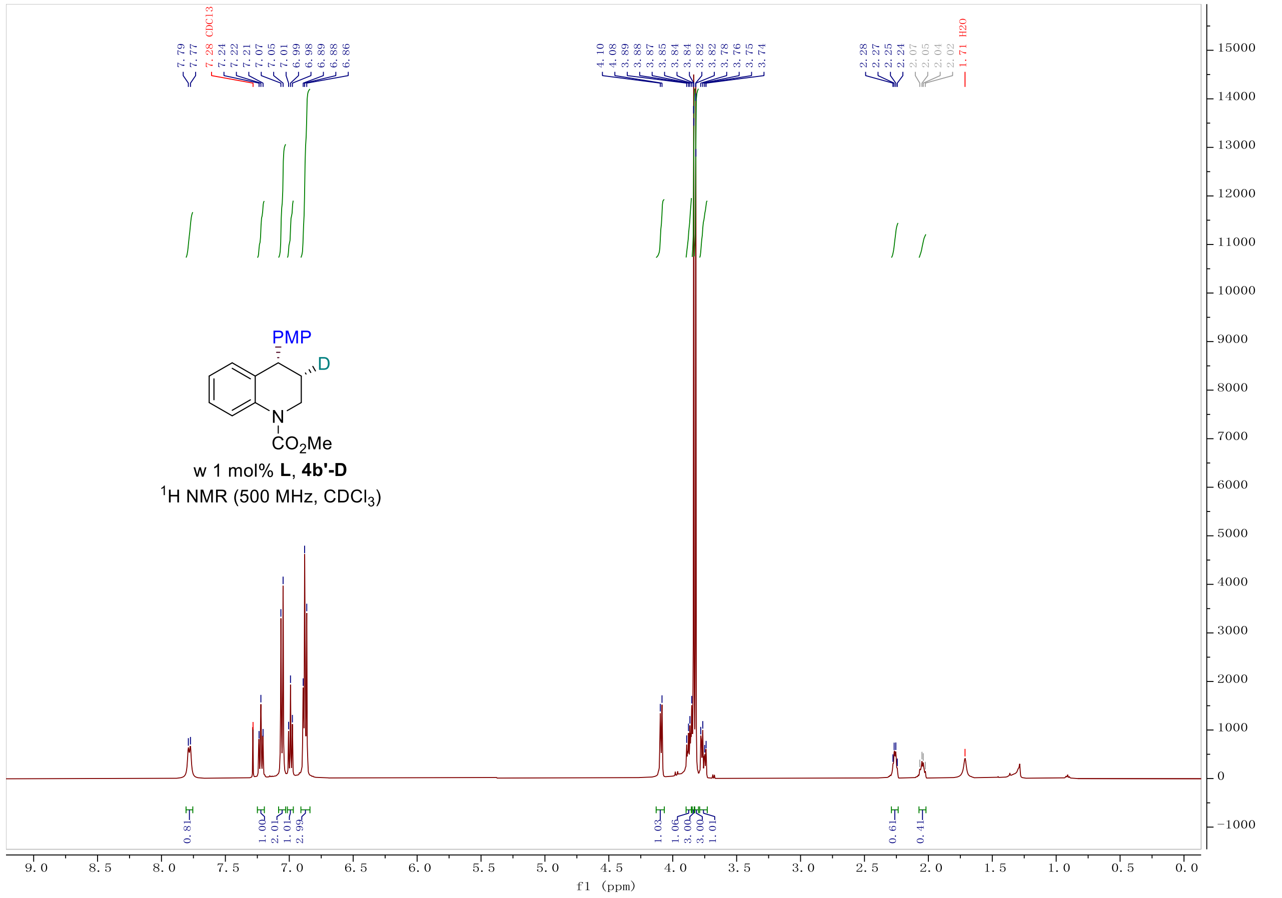

Supplementary Fig. 128. ^{1}H NMR of compound **4b'-D** (w 1 mol% **L**)

2.13. Spectroscopic Data (HPLC Trace)

Data File E:\DATA\20200823\LC 2020-08-23 20-27-59\OnlineEdited--005.D
Sample Name: YH-17-99-RAC

===

Acq. Operator : SYSTEM   Seq. Line : 5
Acq. Instrument : HPLC1260   Location : P1-B1
Injection Date : 8/23/2020 10:42:48 PM   Inj : 1
Inj Volume : 3.000 µl
Different Inj Volume from Sample Entry! Actual Inj Volume : 1.000 µl
Method : E:\DATA\20200823\LC 2020-08-23 20-27-59\1EtOH_40_5_1.M (Sequence Method)
Last changed : 8/23/2020 9:45:14 PM by SYSTEM
Additional Info : Peak(s) manually integrated

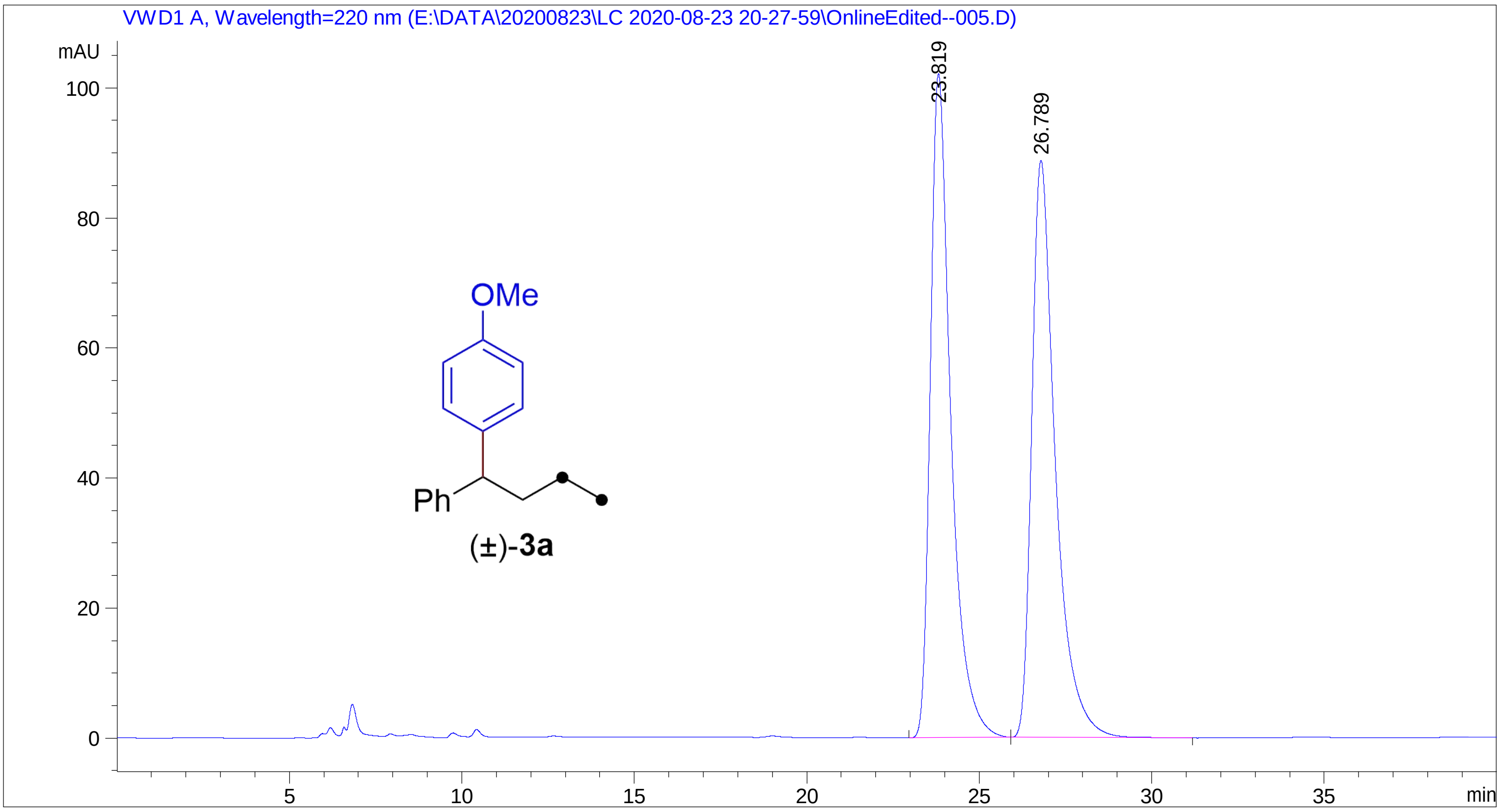

===

Area Percent Report

===

Sorted By : Signal
Multiplier : 1.0000
Dilution : 1.0000
Use Multiplier & Dilution Factor with ISTDs

Signal 1: VWD1 A, Wavelength=220 nm

Peak #	RetTime [min]	Type	Width [min]	Area [mAU*s]	Height [mAU]	Area %
1	23.819	BB	0.6182	4220.85645	102.11806	49.9447
2	26.789	BB	0.7080	4230.19873	88.77055	50.0553

Totals : 8451.05518 190.88861

===

*** End of Report ***

Data File E:\DATA\20200823\LC 2020-08-23 20-27-59\OnlineEdited--006.D
Sample Name: YH-17-99-EE

===

Acq. Operator : SYSTEM   Seq. Line : 6
Acq. Instrument : HPLC1260   Location : P1-B2
Injection Date : 8/23/2020 11:23:33 PM   Inj : 1
Inj Volume : 3.000 µl
Different Inj Volume from Sample Entry! Actual Inj Volume : 1.000 µl
Method : E:\DATA\20200823\LC 2020-08-23 20-27-59\1EtOH_40_5_1.M (Sequence Method)
Last changed : 8/23/2020 9:45:14 PM by SYSTEM
Additional Info : Peak(s) manually integrated

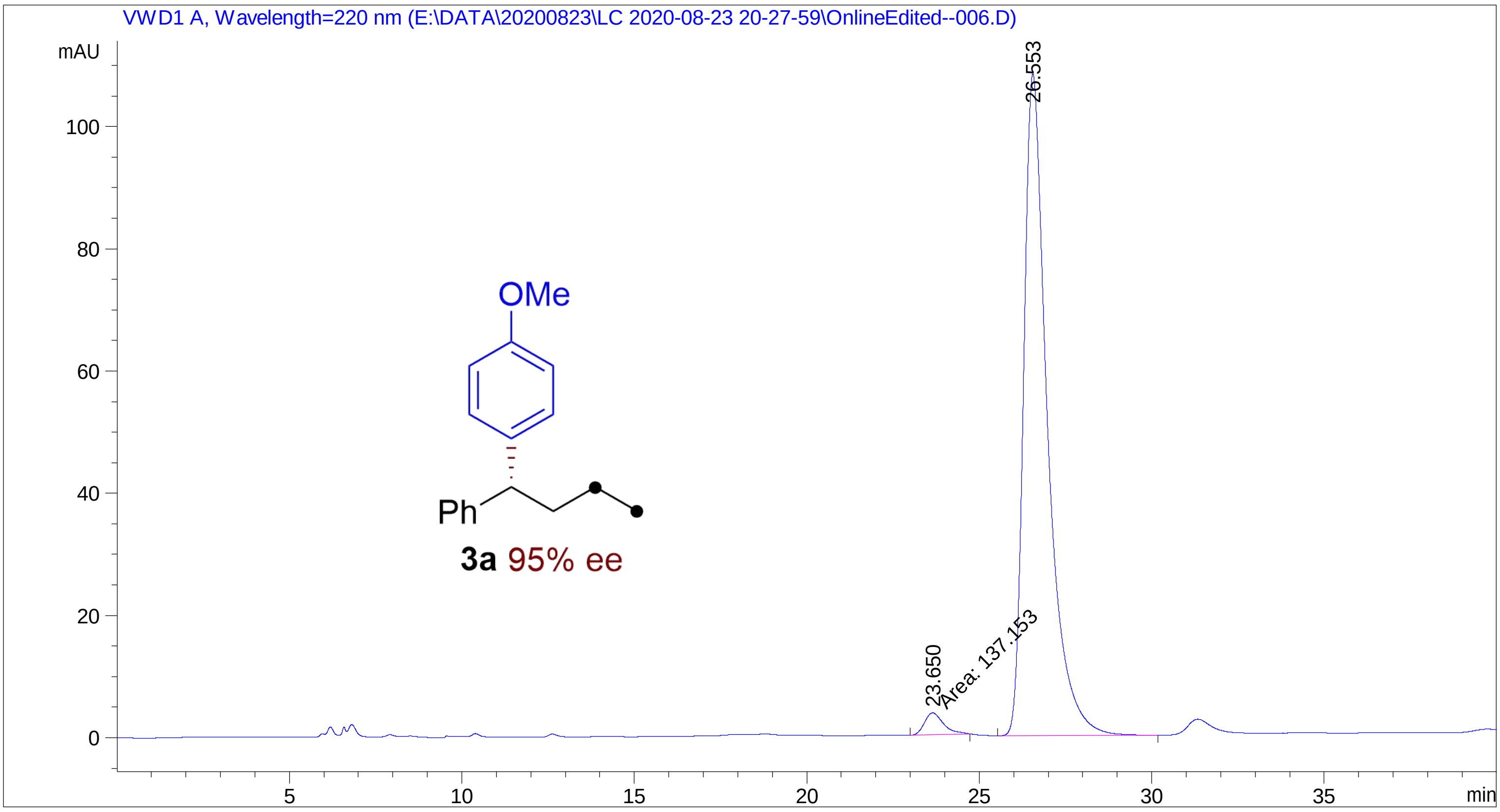

===

Area Percent Report

===

Sorted By : Signal
Multiplier : 1.0000
Dilution : 1.0000
Use Multiplier & Dilution Factor with ISTDs

Signal 1: VWD1 A, Wavelength=220 nm

Peak #	RetTime [min]	Type	Width [min]	Area [mAU*s]	Height [mAU]	Area %
1	23.650	MM	0.6355	137.15309	3.59698	2.5840
2	26.553	BB	0.7092	5170.54736	108.26321	97.4160

Totals : 5307.70045 111.86019

===

*** End of Report ***

Data File E:\DATA\20200829\LC 2020-08-30 17-08-41\OnlineEdited--010.D
Sample Name: YH-17-114-RAC

===

Acq. Operator : SYSTEM Seq. Line : 10
Acq. Instrument : HPLC1260 Location : P1-A3
Injection Date : 8/30/2020 11:52:20 PM Inj : 1
Inj Volume : 3.000 µl
Different Inj Volume from Sample Entry! Actual Inj Volume : 1.000 µl
Acq. Method : E:\DATA\20200829\LC 2020-08-30 17-08-41\1IPA_40_8_1.M
Last changed : 8/30/2020 10:59:52 PM by SYSTEM
Analysis Method : E:\DATA\20200829\LC 2020-08-30 17-08-41\1IPA_40_8_1.M (Sequence Method)
Last changed : 8/31/2020 8:29:58 AM by SYSTEM
(modified after loading)
Additional Info : Peak(s) manually integrated

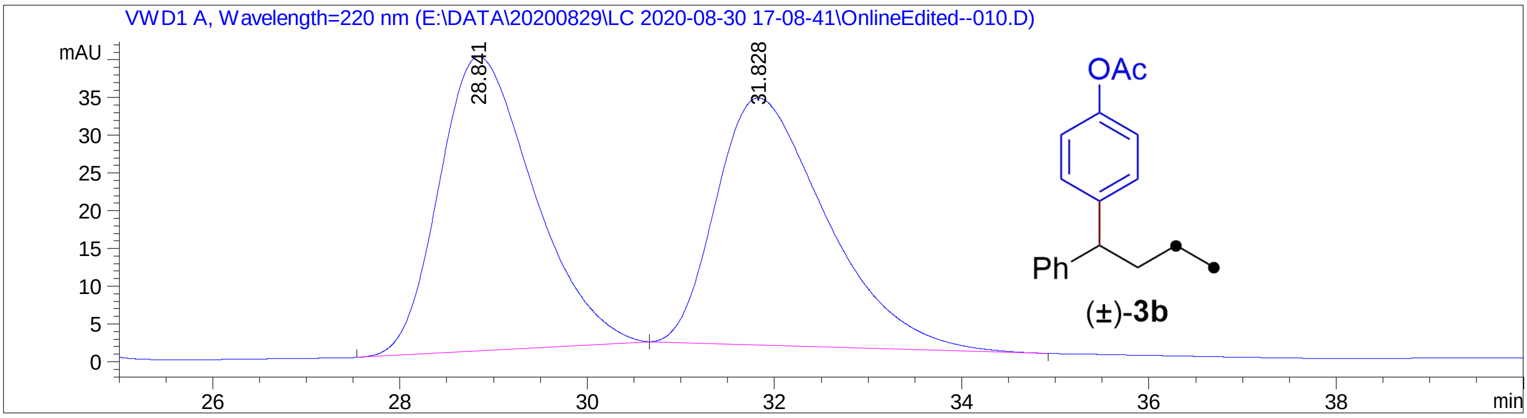

===

Area Percent Report

===

Sorted By : Signal
Multiplier : 1.0000
Dilution : 1.0000
Do not use Multiplier & Dilution Factor with ISTDs

Signal 1: VWD1 A, Wavelength=220 nm

Peak #	RetTime [min]	Type	Width [min]	Area [mAU*s]	Height [mAU]	Area %
1	28.841	BB	1.0787	2737.25195	39.00799	50.5082
2	31.828	BB	1.2281	2682.16821	32.72819	49.4918

Totals : 5419.42017 71.73618

===

*** End of Report ***

===

Acq. Operator : SYSTEM Seq. Line : 11
Acq. Instrument : HPLC1260 Location : P1-A4
Injection Date : 8/31/2020 12:33:04 AM Inj : 1
Inj Volume : 3.000 µl
Different Inj Volume from Sample Entry! Actual Inj Volume : 1.000 µl
Acq. Method : E:\DATA\20200829\LC 2020-08-30 17-08-41\1IPA_40_8_1.M
Last changed : 8/30/2020 10:59:52 PM by SYSTEM
Analysis Method : E:\DATA\20200829\LC 2020-08-30 17-08-41\1IPA_40_8_1.M (Sequence Method)
Last changed : 8/31/2020 8:29:58 AM by SYSTEM
(modified after loading)
Additional Info : Peak(s) manually integrated

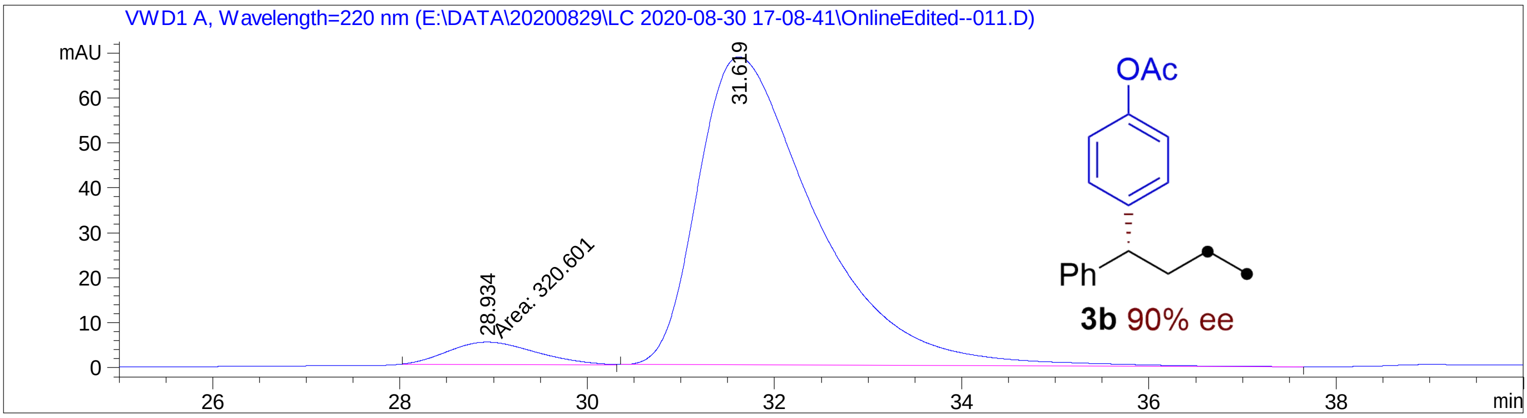

===

Area Percent Report

===

Sorted By : Signal
Multiplier : 1.0000
Dilution : 1.0000
Do not use Multiplier & Dilution Factor with ISTDs

Signal 1: VWD1 A, Wavelength=220 nm

Peak #	RetTime [min]	Type	Width [min]	Area [mAU*s]	Height [mAU]	Area %
1	28.934	MM	1.0810	320.60114	4.94317	5.0726
2	31.619	BB	1.3067	5999.65186	68.51117	94.9274

Totals : 6320.25299 73.45434

===

*** End of Report ***

===

Acq. Operator : SYSTEM Seq. Line : 6
Acq. Instrument : HPLC1260 Location : P1-B1
Injection Date : 9/1/2020 11:33:42 AM Inj : 1
Inj Volume : 3.000 µl
Different Inj Volume from Sample Entry! Actual Inj Volume : 5.000 µl
Acq. Method : E:\DATA\20200831\LC 2020-09-01 08-57-38\1IPA_80_8_1.M
Last changed : 9/1/2020 9:51:26 AM by SYSTEM
Analysis Method : E:\DATA\20200831\LC 2020-09-01 08-57-38\1IPA_80_8_1.M (Sequence Method)
Last changed : 9/1/2020 2:27:42 PM by SYSTEM
(modified after loading)
Additional Info : Peak(s) manually integrated

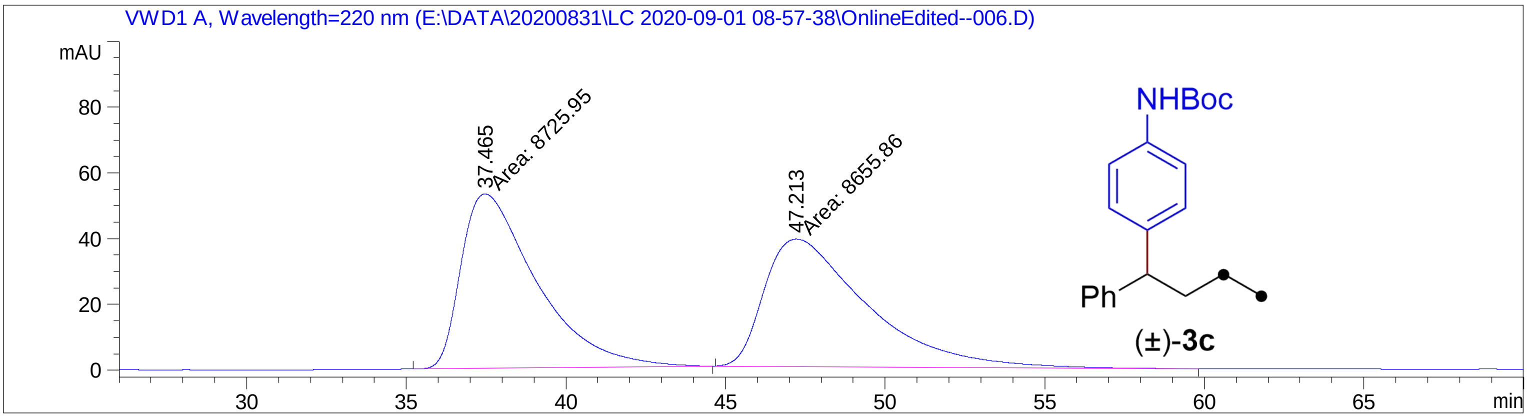

===

Area Percent Report

===

Sorted By : Signal
Multiplier : 1.0000
Dilution : 1.0000
Do not use Multiplier & Dilution Factor with ISTDs

Signal 1: VWD1 A, Wavelength=220 nm

Peak #	RetTime [min]	Type	Width [min]	Area [mAU*s]	Height [mAU]	Area %
1	37.465	MM	2.7428	8725.95410	53.02317	50.2016
2	47.213	MM	3.7236	8655.86230	38.74373	49.7984

Totals : 1.73818e4 91.76690

===

*** End of Report ***

===

Acq. Operator : SYSTEM   Seq. Line : 7
Acq. Instrument : HPLC1260   Location : P1-B2
Injection Date : 9/1/2020 12:54:30 PM   Inj : 1
Inj Volume : 3.000 µl
Acq. Method : E:\DATA\20200831\LC 2020-09-01 08-57-38\1IPA_80_8_1.M
Last changed : 9/1/2020 9:51:26 AM by SYSTEM
Analysis Method : E:\DATA\20200831\LC 2020-09-01 08-57-38\1IPA_80_8_1.M (Sequence Method)
Last changed : 9/1/2020 2:27:42 PM by SYSTEM
(modified after loading)
Additional Info : Peak(s) manually integrated

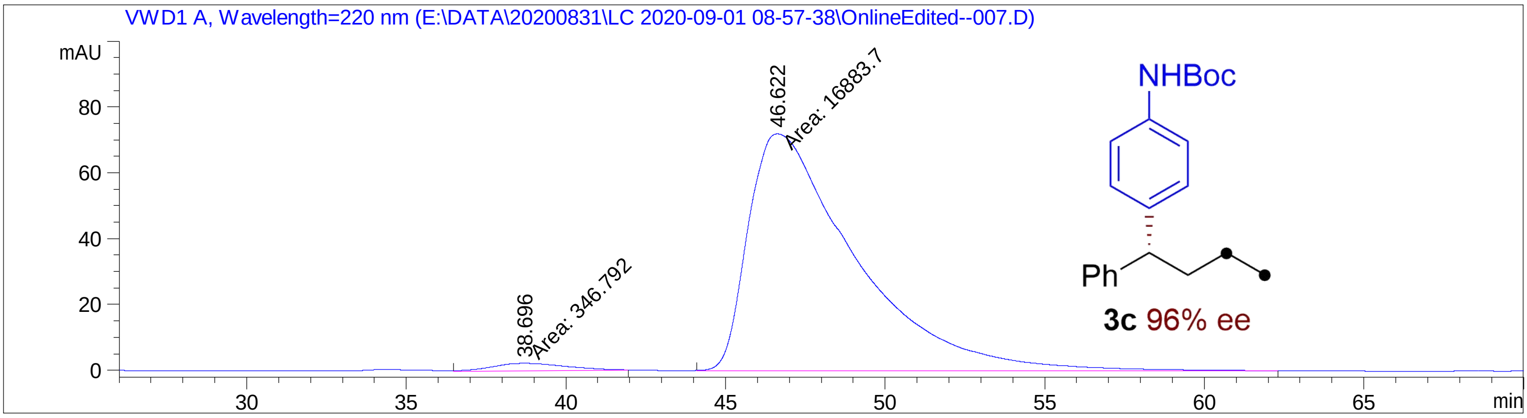

===

Area Percent Report

===

Sorted By : Signal
Multiplier : 1.0000
Dilution : 1.0000
Do not use Multiplier & Dilution Factor with ISTDs

Signal 1: VWD1 A, Wavelength=220 nm

Peak #	RetTime [min]	Type	Width [min]	Area [mAU*s]	Height [mAU]	Area %
1	38.696	MM	1.8465	346.79208	2.20724	2.0127
2	46.622	MM	3.9112	1.68837e4	71.94666	97.9873

Totals : 1.72305e4 74.15389

===

*** End of Report ***

Data File E:\DATA\20200831\LC 2020-09-01 08-57-38\OnlineEdited--017.D
Sample Name: YH-17-117-RAC

===

Acq. Operator : SYSTEM   Seq. Line : 17
Acq. Instrument : HPLC1260   Location : P1-B7
Injection Date : 9/1/2020 4:52:28 PM   Inj : 1
Inj Volume : 3.000 µl
Different Inj Volume from Sample Entry! Actual Inj Volume : 5.000 µl
Acq. Method : E:\DATA\20200831\LC 2020-09-01 08-57-38\5IPA_30_5_1.M
Last changed : 9/1/2020 4:57:29 PM by SYSTEM
(modified after loading)
Analysis Method : E:\DATA\20200831\LC 2020-09-01 08-57-38\5IPA_30_5_1.M (Sequence Method)
Last changed : 9/1/2020 6:08:47 PM by SYSTEM
(modified after loading)
Additional Info : Peak(s) manually integrated

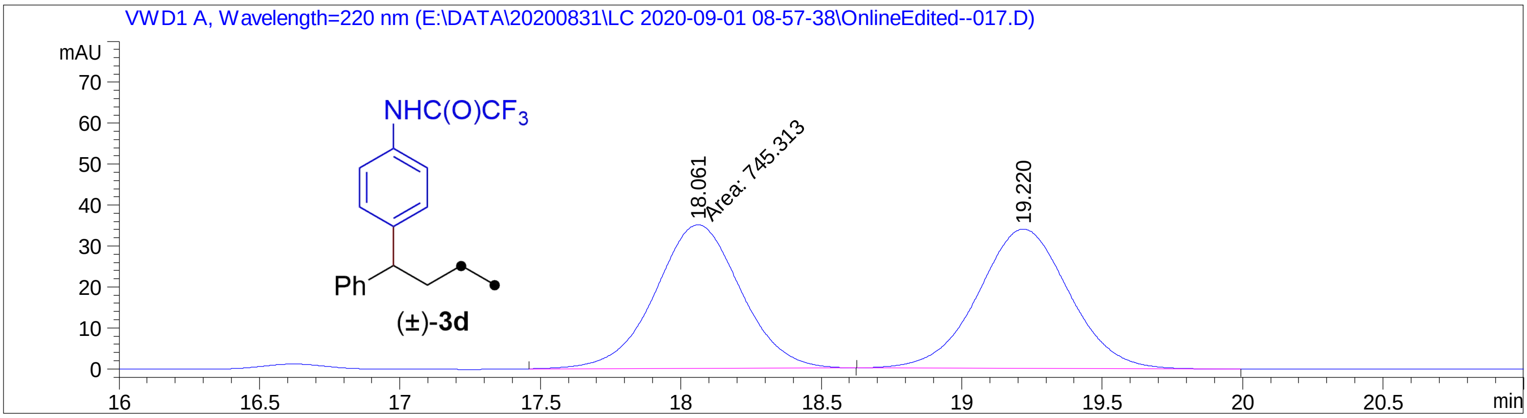

===

Area Percent Report

===

Sorted By : Signal
Multiplier : 1.0000
Dilution : 1.0000
Do not use Multiplier & Dilution Factor with ISTDs

Signal 1: VWD1 A, Wavelength=220 nm

Peak #	RetTime [min]	Type	Width [min]	Area [mAU*s]	Height [mAU]	Area %
1	18.061	FM	0.3541	745.31293	35.08218	49.2961
2	19.220	BB	0.3470	766.59802	34.00572	50.7039

Totals : 1511.91095 69.08790

===

*** End of Report ***

Data File E:\DATA\20200831\LC 2020-09-01 08-57-38\OnlineEdited--018.D
Sample Name: YH-17-117-EE

===

Acq. Operator : SYSTEM Seq. Line : 18
Acq. Instrument : HPLC1260 Location : P1-B2
Injection Date : 9/1/2020 5:33:15 PM Inj : 1
Inj Volume : 3.000 µl
Different Inj Volume from Sample Entry! Actual Inj Volume : 1.000 µl
Acq. Method : E:\DATA\20200831\LC 2020-09-01 08-57-38\5IPA_30_5_1.M
Last changed : 9/1/2020 6:07:00 PM by SYSTEM
(modified after loading)
Analysis Method : E:\DATA\20200831\LC 2020-09-01 08-57-38\5IPA_30_5_1.M (Sequence Method)
Last changed : 9/1/2020 6:09:32 PM by SYSTEM
(modified after loading)
Additional Info : Peak(s) manually integrated

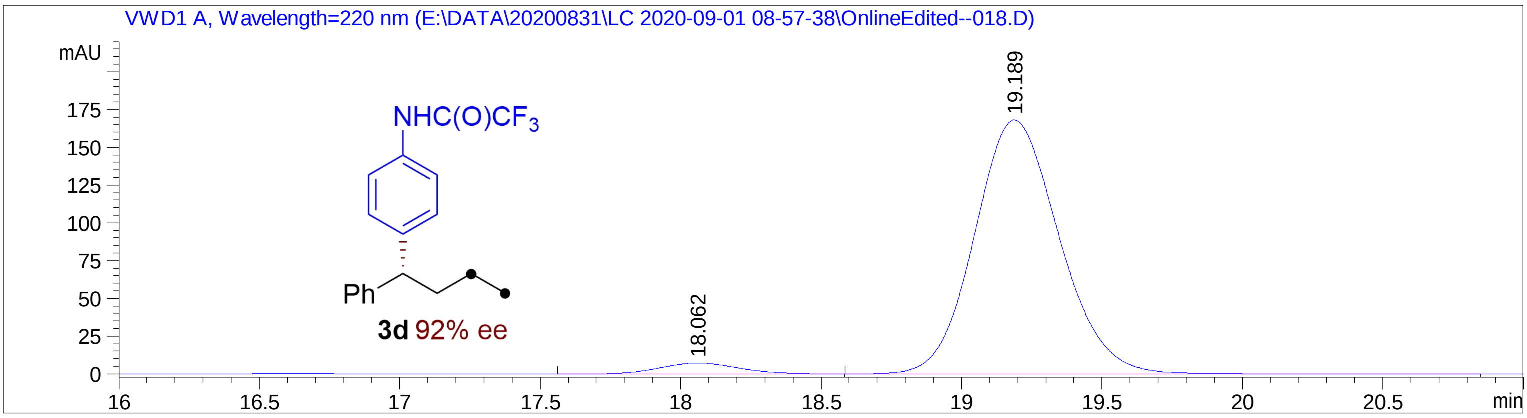

===

Area Percent Report

===

Sorted By : Signal
Multiplier : 1.0000
Dilution : 1.0000
Do not use Multiplier & Dilution Factor with ISTDs

Signal 1: VWD1 A, Wavelength=220 nm

Peak #	RetTime [min]	Type	Width [min]	Area [mAU*s]	Height [mAU]	Area %
1	18.062	BB	0.3017	141.60934	7.25616	3.8666
2	19.189	BB	0.3244	3520.74316	168.05702	96.1334

Totals : 3662.35251 175.31318

===

*** End of Report ***

Data File E:\DATA\20200831\LC 2020-09-04 14-19-23\1AA-0201.D
Sample Name: YH-17-119-RAC

===

Acq. Operator : SYSTEM Seq. Line : 2
Acq. Instrument : HPLC1260 Location : P1-A1
Injection Date : 9/4/2020 2:42:22 PM Inj : 1
Inj Volume : 3.000 µl
Different Inj Volume from Sample Entry! Actual Inj Volume : 1.000 µl
Acq. Method : E:\DATA\20200831\LC 2020-09-04 14-19-23\0IPA_25_8_3.M
Last changed : 9/4/2020 2:27:06 PM by SYSTEM
Analysis Method : E:\DATA\20200831\LC 2020-09-04 14-19-23\0IPA_25_8_3.M (Sequence Method)
Last changed : 9/4/2020 2:41:43 PM by SYSTEM
Additional Info : Peak(s) manually integrated

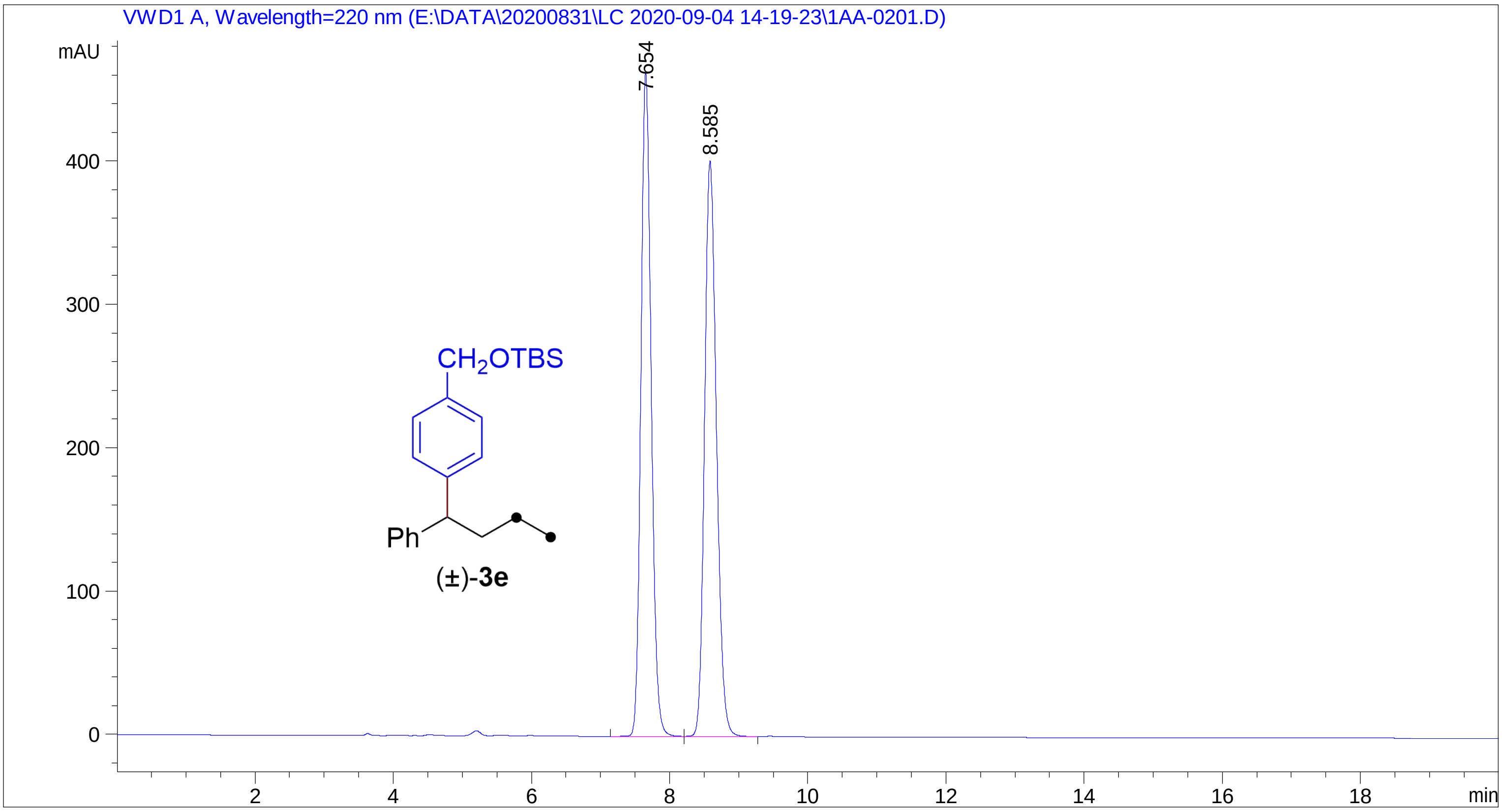

===

Area Percent Report

===

Sorted By : Signal
Multiplier : 1.0000
Dilution : 1.0000
Use Multiplier & Dilution Factor with ISTDs

Signal 1: VWD1 A, Wavelength=220 nm

Peak #	RetTime [min]	Type	Width [min]	Area [mAU*s]	Height [mAU]	Area %
1	7.654	BB	0.1607	4785.52490	462.56711	49.9838
2	8.585	BB	0.1852	4788.62549	401.44867	50.0162
Totals :				9574.15039	864.01578	

===

Data File E:\DATA\20200831\LC 2020-09-04 14-19-23\1AB-0301.D
Sample Name: YH-17-119-EE

===

Acq. Operator : SYSTEM   Seq. Line : 3
Acq. Instrument : HPLC1260   Location : P1-A2
Injection Date : 9/4/2020 3:03:07 PM   Inj : 1
                                       Inj Volume : 3.000 µl
Different Inj Volume from Sample Entry! Actual Inj Volume : 1.000 µl
Acq. Method : E:\DATA\20200831\LC 2020-09-04 14-19-23\0IPA_25_8_3.M
Last changed : 9/4/2020 2:27:06 PM by SYSTEM
Analysis Method : E:\DATA\20200831\LC 2020-09-04 14-19-23\0IPA_25_8_3.M (Sequence Method)
Last changed : 9/4/2020 2:41:43 PM by SYSTEM
Additional Info : Peak(s) manually integrated

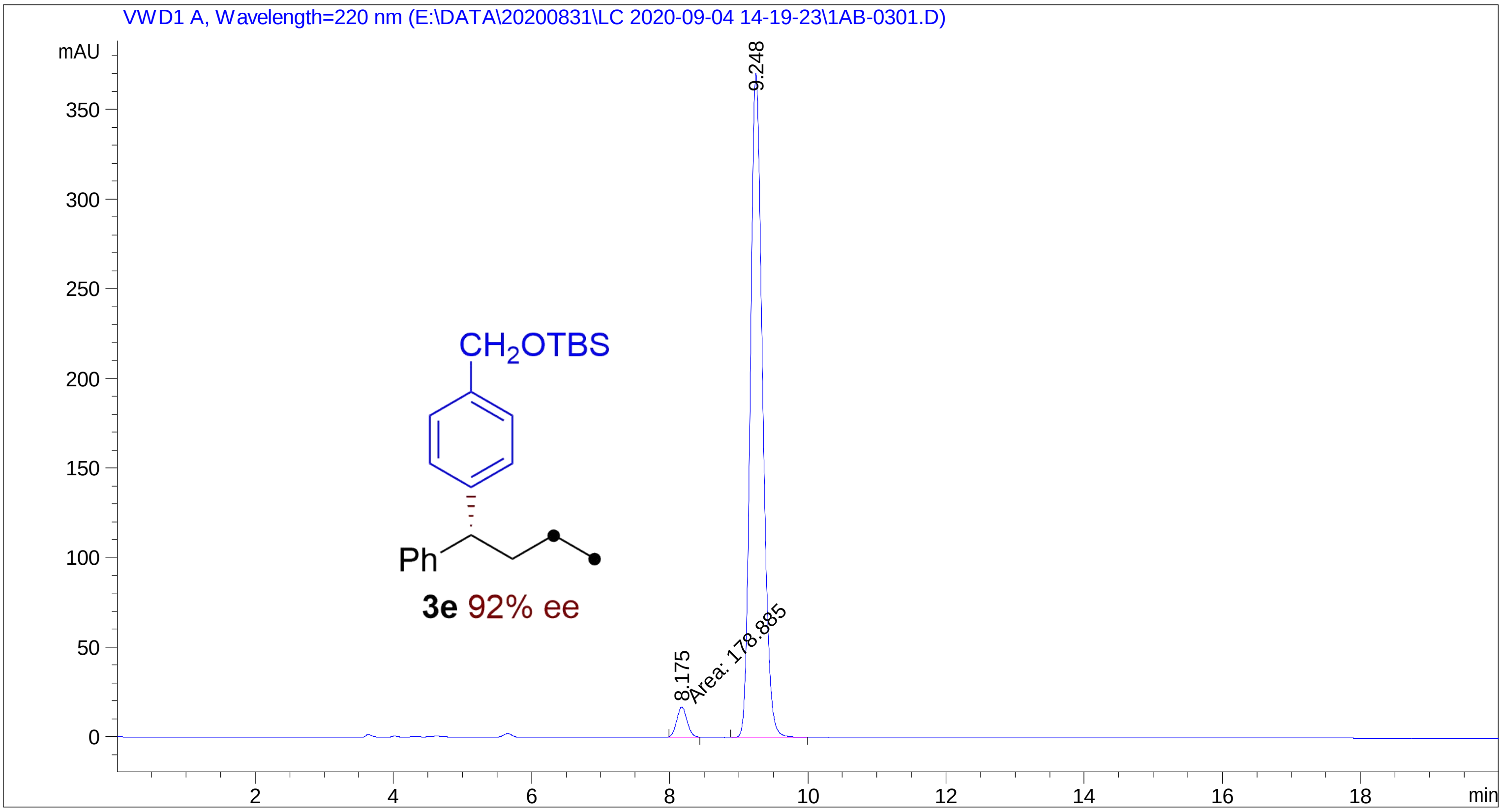

===

Area Percent Report

===

Sorted By : Signal
Multiplier : 1.0000
Dilution : 1.0000
Use Multiplier & Dilution Factor with ISTDs

Signal 1: VWD1 A, Wavelength=220 nm

Peak #	RetTime [min]	Type	Width [min]	Area [mAU*s]	Height [mAU]	Area %
1	8.175	FM	0.1756	178.88454	16.97795	3.7939
2	9.248	BB	0.1889	4536.12598	370.32483	96.2061

Totals : 4715.01051 387.30278

===

Data File E:\DATA\20200828\LC 2020-08-28 17-26-16\OnlineEdited--026.D
Sample Name: YH-17-108-RAC

===

Acq. Operator : SYSTEM Seq. Line : 26
Acq. Instrument : HPLC1260 Location : P1-A1
Injection Date : 8/29/2020 7:41:50 AM Inj : 1
Inj Volume : 3.000 µl
Different Inj Volume from Sample Entry! Actual Inj Volume : 1.000 µl
Acq. Method : E:\DATA\20200828\LC 2020-08-28 17-26-16\5IPA_35_10_1.M
Last changed : 8/29/2020 7:59:24 AM by SYSTEM
(modified after loading)
Analysis Method : E:\DATA\20200828\LC 2020-08-28 17-26-16\5IPA_35_10_1.M (Sequence Method)
Last changed : 8/29/2020 8:51:39 AM by SYSTEM
(modified after loading)
Additional Info : Peak(s) manually integrated

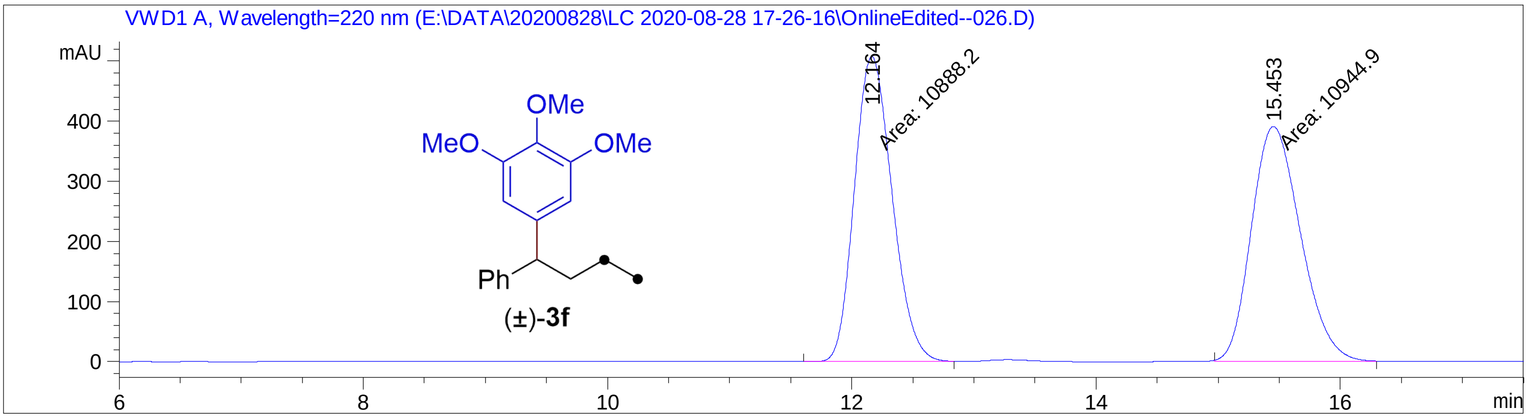

===

Area Percent Report

===

Sorted By : Signal
Multiplier : 1.0000
Dilution : 1.0000
Do not use Multiplier & Dilution Factor with ISTDs

Signal 1: VWD1 A, Wavelength=220 nm

Peak #	RetTime [min]	Type	Width [min]	Area [mAU*s]	Height [mAU]	Area %
1	12.164	MF	0.3575	1.08882e4	507.60840	49.8701
2	15.453	MF	0.4665	1.09449e4	391.03992	50.1299

Totals : 2.18330e4 898.64832

===

*** End of Report ***

Data File E:\DATA\20200828\LC 2020-08-28 17-26-16\OnlineEdited--027.D
Sample Name: YH-17-108-EE

===

Acq. Operator : SYSTEM Seq. Line : 27
Acq. Instrument : HPLC1260 Location : P1-A2
Injection Date : 8/29/2020 8:12:36 AM Inj : 1
Inj Volume : 3.000 µl
Different Inj Volume from Sample Entry! Actual Inj Volume : 1.000 µl
Acq. Method : E:\DATA\20200828\LC 2020-08-28 17-26-16\5IPA_35_10_1.M
Last changed : 8/29/2020 7:59:24 AM by SYSTEM
Analysis Method : E:\DATA\20200828\LC 2020-08-28 17-26-16\5IPA_35_10_1.M (Sequence Method)
Last changed : 8/29/2020 8:51:39 AM by SYSTEM
(modified after loading)
Additional Info : Peak(s) manually integrated

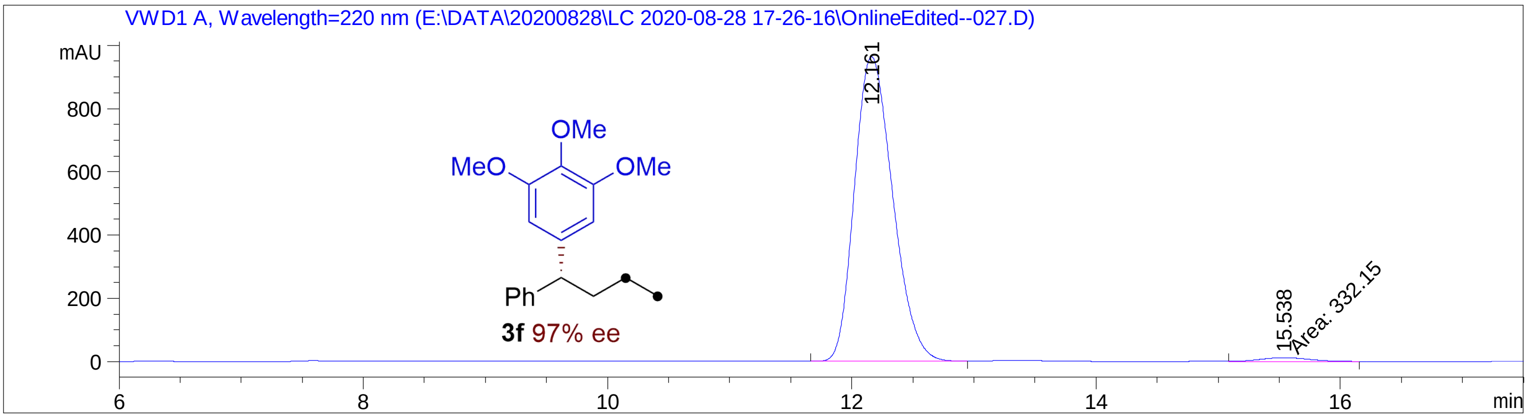

===

Area Percent Report

===

Sorted By : Signal
Multiplier : 1.0000
Dilution : 1.0000
Do not use Multiplier & Dilution Factor with ISTDs

Signal 1: VWD1 A, Wavelength=220 nm

Peak #	RetTime [min]	Type	Width [min]	Area [mAU*s]	Height [mAU]	Area %
1	12.161	BB	0.3418	2.09569e4	963.44781	98.4398
2	15.538	MF	0.4604	332.14978	12.02373	1.5602

Totals : 2.12890e4 975.47155

===

*** End of Report ***

===

Acq. Operator : SYSTEM Seq. Line : 6
Acq. Instrument : HPLC1260 Location : P1-A1
Injection Date : 8/30/2020 9:34:17 PM Inj : 2
Inj Volume : 3.000 µl
Different Inj Volume from Sample Entry! Actual Inj Volume : 1.000 µl
Acq. Method : E:\DATA\20200829\LC 2020-08-30 17-08-41\5IPA_30_5_1.M
Last changed : 8/30/2020 8:45:42 PM by SYSTEM
Analysis Method : E:\DATA\20200829\LC 2020-08-30 17-08-41\5IPA_30_5_1.M (Sequence Method)
Last changed : 8/30/2020 10:35:01 PM by SYSTEM
(modified after loading)
Additional Info : Peak(s) manually integrated

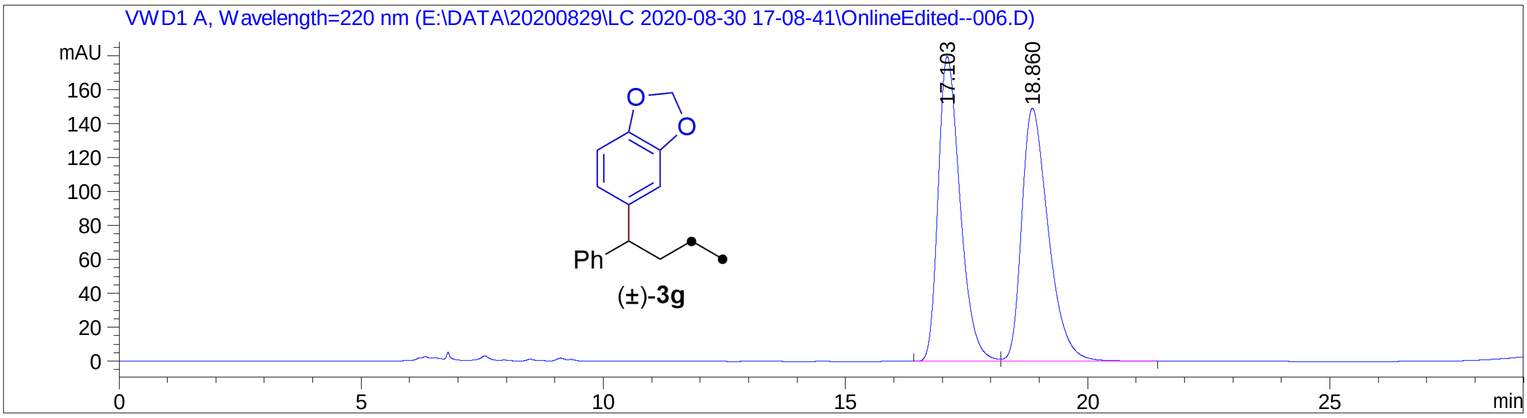

===

Area Percent Report

===

Sorted By : Signal
Multiplier : 1.0000
Dilution : 1.0000
Do not use Multiplier & Dilution Factor with ISTDs

Signal 1: VWD1 A, Wavelength=220 nm

Peak #	RetTime [min]	Type	Width [min]	Area [mAU*s]	Height [mAU]	Area %
1	17.103	BV	0.4842	5670.10205	179.65312	49.8935
2	18.860	VB	0.5823	5694.31055	149.19333	50.1065

Totals : 1.13644e4 328.84645

===

*** End of Report ***

Data File E:\DATA\20200829\LC 2020-08-30 17-08-41\OnlineEdited--007.D
Sample Name: YH-17-116-EE

===

Acq. Operator : SYSTEM Seq. Line : 7
Acq. Instrument : HPLC1260 Location : P1-A2
Injection Date : 8/30/2020 10:05:03 PM Inj : 1
Inj Volume : 3.000 µl
Different Inj Volume from Sample Entry! Actual Inj Volume : 1.000 µl
Acq. Method : E:\DATA\20200829\LC 2020-08-30 17-08-41\5IPA_30_5_1.M
Last changed : 8/30/2020 8:45:42 PM by SYSTEM
Analysis Method : E:\DATA\20200829\LC 2020-08-30 17-08-41\5IPA_30_5_1.M (Sequence Method)
Last changed : 8/30/2020 9:02:51 PM by SYSTEM
Additional Info : Peak(s) manually integrated

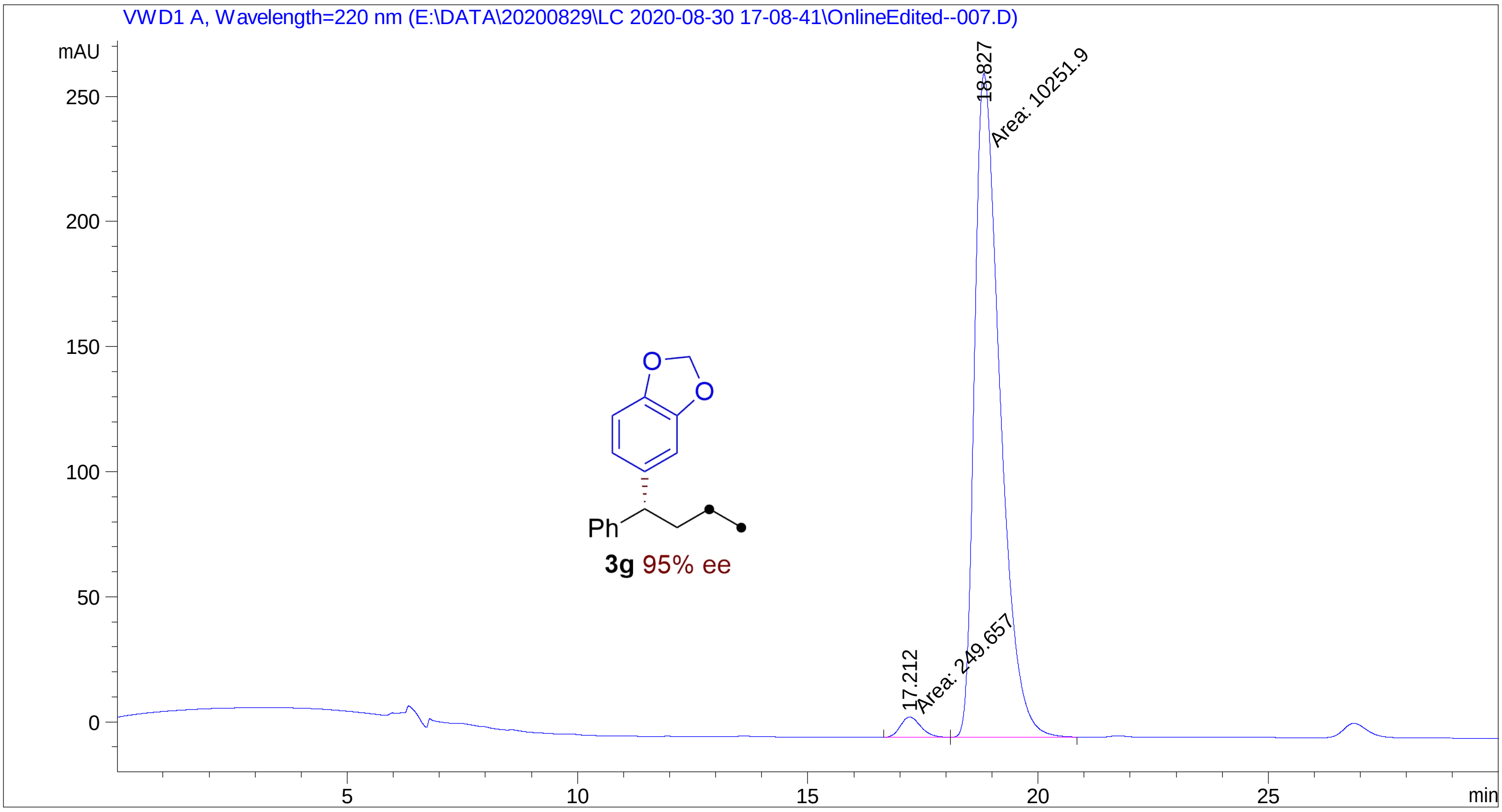

===

Area Percent Report

===

Sorted By : Signal
Multiplier : 1.0000
Dilution : 1.0000
Use Multiplier & Dilution Factor with ISTDs

Signal 1: VWD1 A, Wavelength=220 nm

Peak #	RetTime [min]	Type	Width [min]	Area [mAU*s]	Height [mAU]	Area %
1	17.212	FM	0.5118	249.65678	8.12981	2.3773
2	18.827	MF	0.6441	1.02519e4	265.28772	97.6227

Totals : 1.05016e4 273.41753

===

Data File E:\DATA\20200829\LC 2020-08-30 17-08-41\OnlineEdited--016.D
Sample Name: YH-17-112-RAC

===

Acq. Operator	: SYSTEM	Seq. Line :	16
Acq. Instrument	: HPLC1260	Location :	P1-A1
Injection Date	: 8/31/2020 5:17:01 AM	Inj :	1
		Inj Volume :	3.000 µl

Different Inj Volume from Sample Entry!   Actual Inj Volume : 0.500 µl
Method   : E:\DATA\20200829\LC 2020-08-30 17-08-41\0IPA_60_8_2.M (Sequence Method)
Last changed   : 8/30/2020 10:56:56 PM by SYSTEM

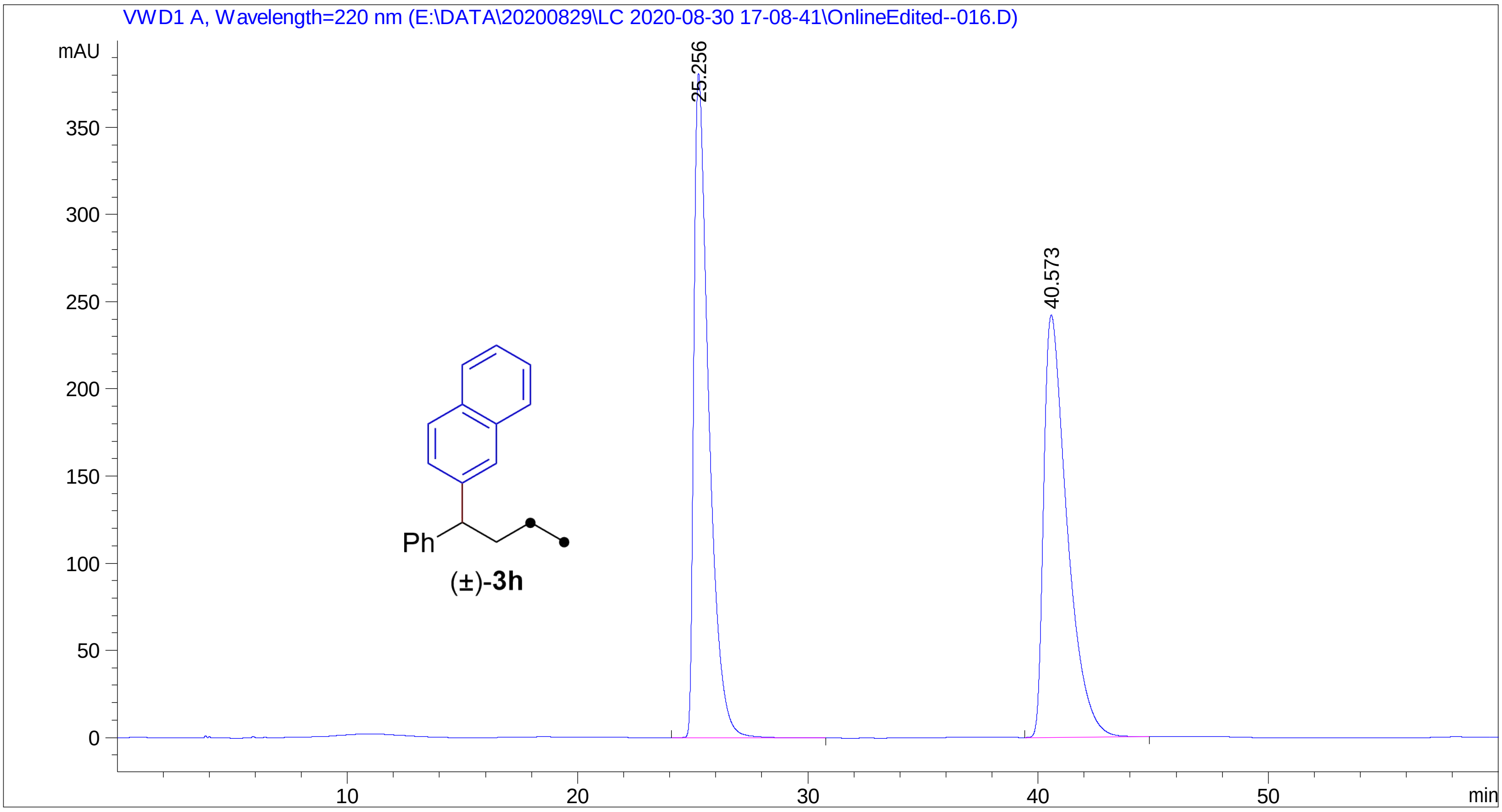

===

Area Percent Report

===

Sorted By   :   Signal
Multiplier   :   1.0000
Dilution   :   1.0000
Use Multiplier & Dilution Factor with ISTDs

Signal 1: VWD1 A, Wavelength=220 nm

Peak #	RetTime [min]	Type	Width [min]	Area [mAU*s]	Height [mAU]	Area %
1	25.256	BB	0.6736	1.71626e4	380.94769	50.1021
2	40.573	BB	1.0553	1.70926e4	242.22771	49.8979
Totals :				3.42552e4	623.17540	

===

*** End of Report ***

Data File E:\DATA\20200829\LC 2020-08-30 17-08-41\OnlineEdited--017.D
Sample Name: YH-17-112-EE

===

Acq. Operator : SYSTEM Seq. Line : 17
Acq. Instrument : HPLC1260 Location : P1-A2
Injection Date : 8/31/2020 6:17:47 AM Inj : 1
Inj Volume : 3.000 µl
Different Inj Volume from Sample Entry! Actual Inj Volume : 1.000 µl
Method : E:\DATA\20200829\LC 2020-08-30 17-08-41\0IPA_60_8_2.M (Sequence Method)
Last changed : 8/30/2020 10:56:56 PM by SYSTEM
Additional Info : Peak(s) manually integrated

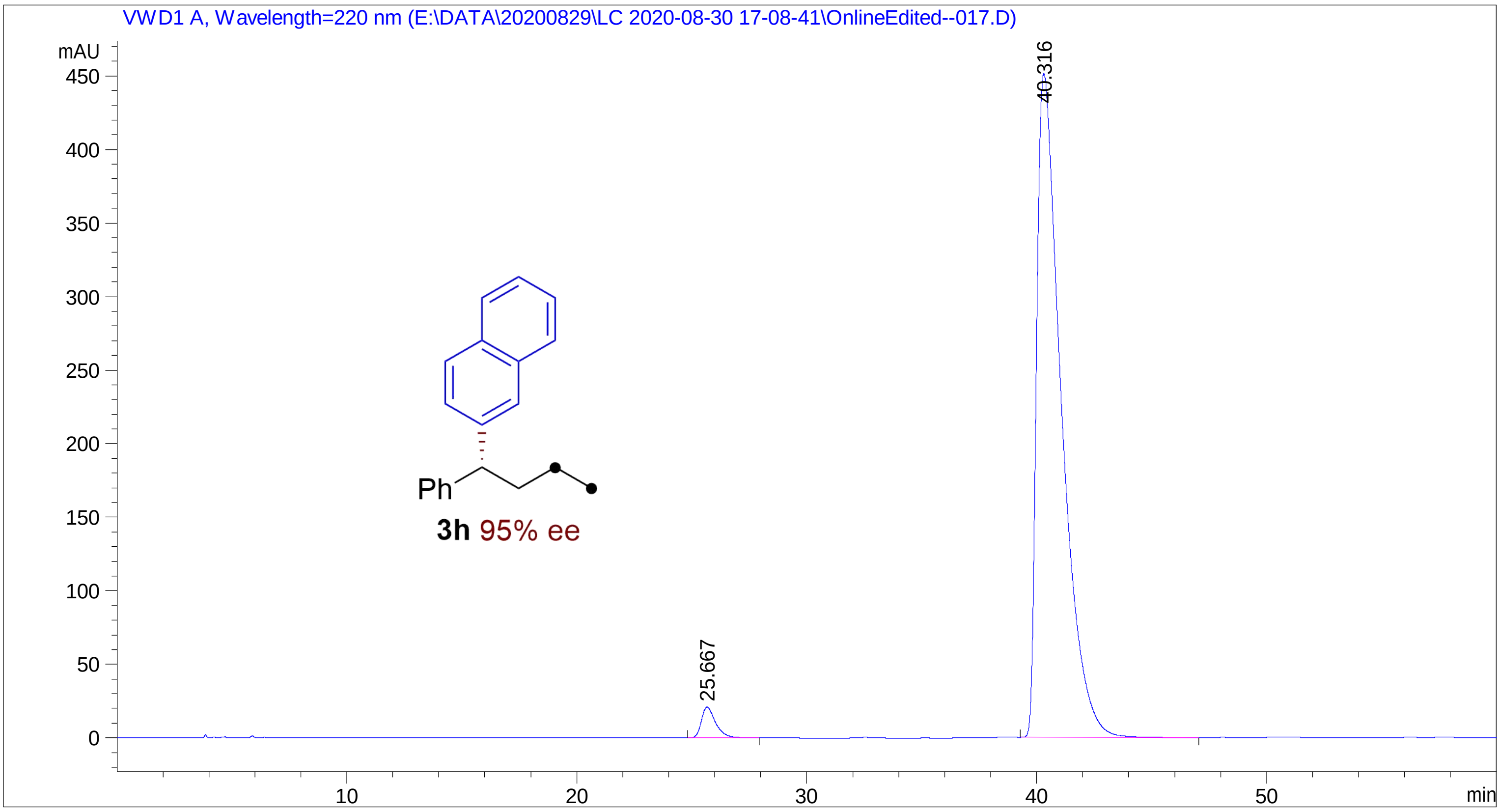

===

Area Percent Report

===

Sorted By : Signal
Multiplier : 1.0000
Dilution : 1.0000
Use Multiplier & Dilution Factor with ISTDs

Signal 1: VWD1 A, Wavelength=220 nm

Peak #	RetTime [min]	Type	Width [min]	Area [mAU*s]	Height [mAU]	Area %
1	25.667	BB	0.6395	893.69812	20.95575	2.5802
2	40.316	BB	1.1145	3.37432e4	451.20450	97.4198

Totals : 3.46369e4 472.16025

===

*** End of Report ***

===

Acq. Operator : SYSTEM Seq. Line : 3
Acq. Instrument : HPLC1260 Location : P1-A1
Injection Date : 8/29/2020 11:36:05 AM Inj : 2
Inj Volume : 3.000 µl
Different Inj Volume from Sample Entry! Actual Inj Volume : 0.500 µl
Acq. Method : E:\DATA\20200828\LC 2020-08-29 10-07-26\1EtOH_35_5_1.M
Last changed : 8/29/2020 11:01:44 AM by SYSTEM
Analysis Method : E:\DATA\20200828\LC 2020-08-29 10-07-26\1EtOH_35_5_1.M (Sequence Method)
Last changed : 8/29/2020 4:38:45 PM by SYSTEM
(modified after loading)
Additional Info : Peak(s) manually integrated

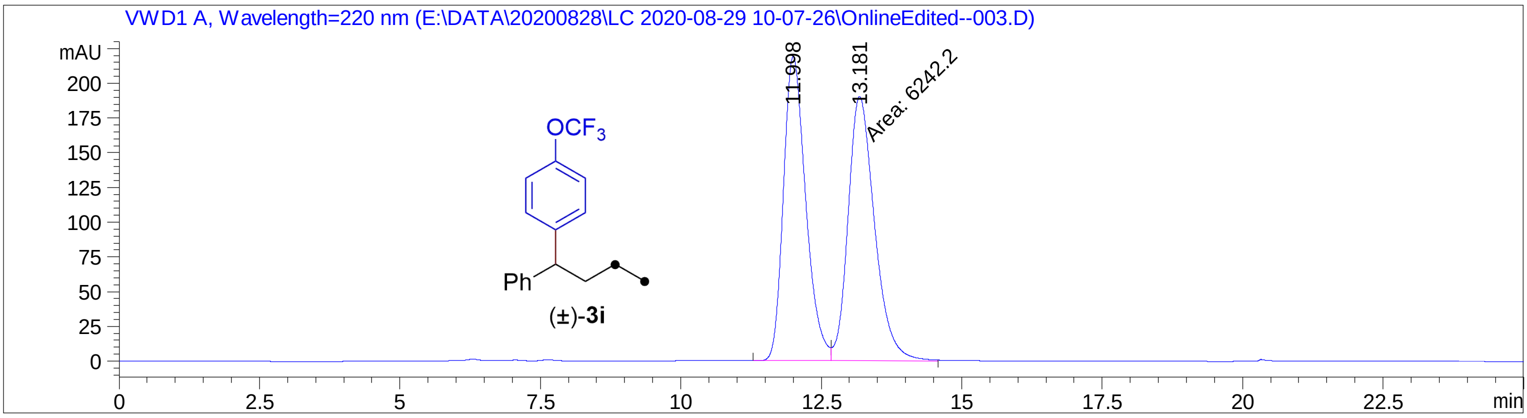

===

Area Percent Report

===

Sorted By : Signal
Multiplier : 1.0000
Dilution : 1.0000
Do not use Multiplier & Dilution Factor with ISTDs

Signal 1: VWD1 A, Wavelength=220 nm

Peak #	RetTime [min]	Type	Width [min]	Area [mAU*s]	Height [mAU]	Area %
1	11.998	BV	0.4249	6057.66504	218.84766	49.2499
2	13.181	MF	0.5470	6242.19678	190.17905	50.7501

Totals : 1.22999e4 409.02670

===

*** End of Report ***

Data File E:\DATA\20200828\LC 2020-08-29 10-07-26\OnlineEdited--004.D
Sample Name: YH-17-109-EE

===

Acq. Operator : SYSTEM Seq. Line : 4
Acq. Instrument : HPLC1260 Location : P1-A2
Injection Date : 8/29/2020 12:26:53 PM Inj : 1
Inj Volume : 3.000 µl
Different Inj Volume from Sample Entry! Actual Inj Volume : 1.000 µl
Acq. Method : E:\DATA\20200828\LC 2020-08-29 10-07-26\1EtOH_35_5_1.M
Last changed : 8/29/2020 11:01:44 AM by SYSTEM
Analysis Method : E:\DATA\20200828\LC 2020-08-29 10-07-26\1EtOH_35_5_1.M (Sequence Method)
Last changed : 8/29/2020 4:38:45 PM by SYSTEM
(modified after loading)
Additional Info : Peak(s) manually integrated

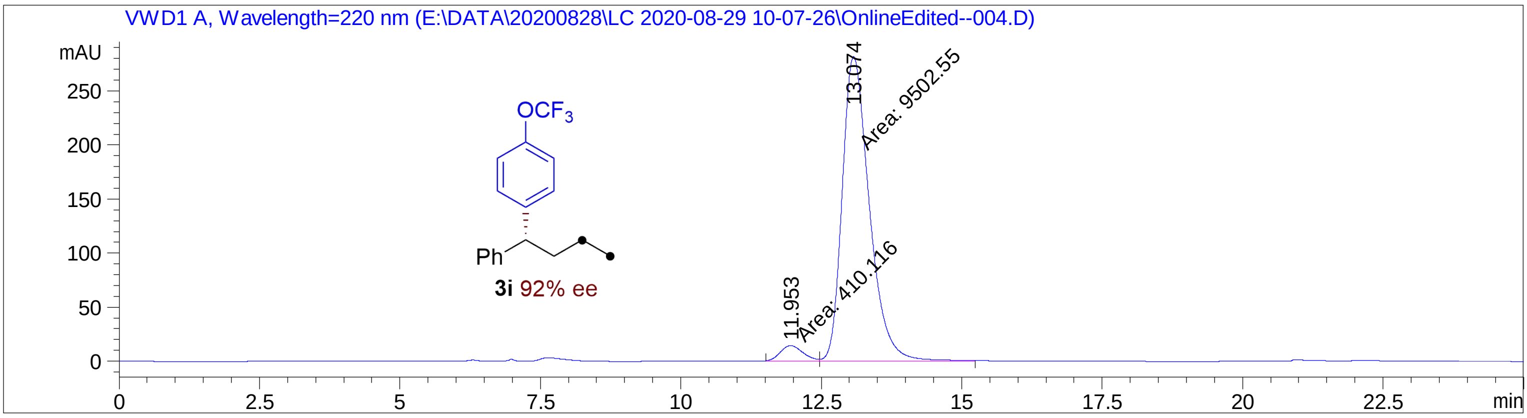

===

Area Percent Report

===

Sorted By : Signal
Multiplier : 1.0000
Dilution : 1.0000
Do not use Multiplier & Dilution Factor with ISTDs

Signal 1: VWD1 A, Wavelength=220 nm

Peak #	RetTime [min]	Type	Width [min]	Area [mAU*s]	Height [mAU]	Area %
1	11.953	FM	0.4739	410.11621	14.42195	4.1373
2	13.074	MF	0.5618	9502.54980	281.88467	95.8627

Totals : 9912.66602 296.30663

===

*** End of Report ***

Data File E:\DATA\20200905\LC 2020-09-06 15-18-12\OnlineEdited--069.D
Sample Name: YH-17-126-RAC

===

Acq. Operator : SYSTEM   Seq. Line : 69
Acq. Instrument : HPLC1260   Location : P1-A7
Injection Date : 9/8/2020 5:49:33 AM   Inj : 1
                                                     Inj Volume : 3.000 µl
Different Inj Volume from Sample Entry! Actual Inj Volume : 1.000 µl
Acq. Method : E:\DATA\20200905\LC 2020-09-06 15-18-12\0IPA_50_4_3.M
Last changed : 9/7/2020 9:42:12 AM by SYSTEM
Analysis Method : E:\DATA\20200905\LC 2020-09-06 15-18-12\0IPA_50_4_3.M (Sequence Method)
Last changed : 9/8/2020 11:31:07 AM by SYSTEM
(modified after loading)
Additional Info : Peak(s) manually integrated

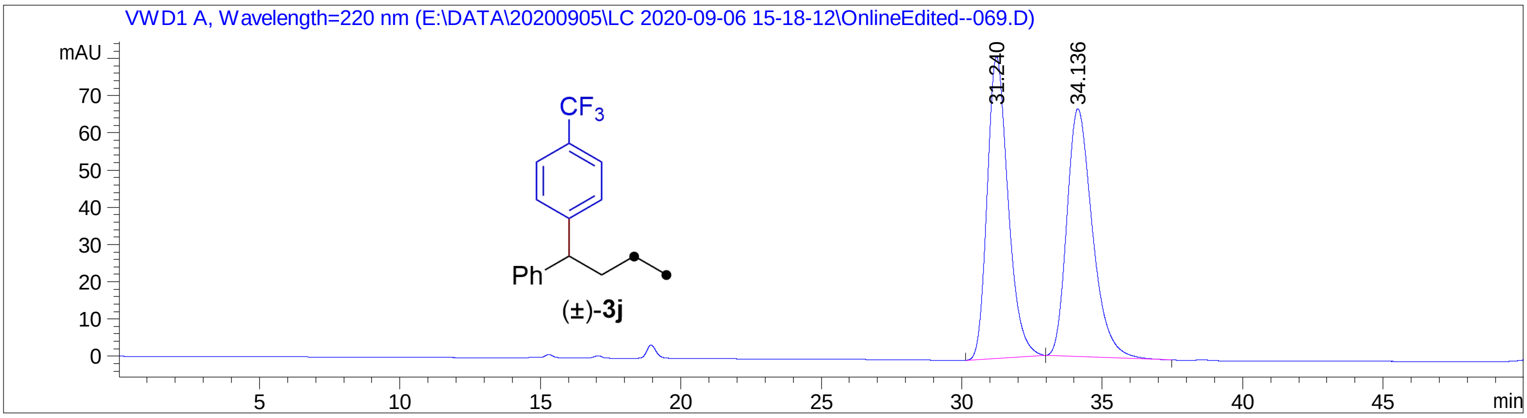

===

Area Percent Report

===

Sorted By : Signal
Multiplier : 1.0000
Dilution : 1.0000
Do not use Multiplier & Dilution Factor with ISTDs

Signal 1: VWD1 A, Wavelength=220 nm

Peak #	RetTime [min]	Type	Width [min]	Area [mAU*s]	Height [mAU]	Area %
1	31.240	BB	0.7946	4194.71387	81.07690	49.9003
2	34.136	BB	0.9644	4211.47217	66.53973	50.0997

Totals : 8406.18604 147.61663

===

*** End of Report ***

Data File E:\DATA\20200905\LC 2020-09-06 15-18-12\OnlineEdited--070.D
Sample Name: YH-17-126-EE

===

Acq. Operator : SYSTEM Seq. Line : 70
Acq. Instrument : HPLC1260 Location : P1-A8
Injection Date : 9/8/2020 6:40:17 AM Inj : 1
Inj Volume : 3.000 µl
Different Inj Volume from Sample Entry! Actual Inj Volume : 1.000 µl
Acq. Method : E:\DATA\20200905\LC 2020-09-06 15-18-12\0IPA_50_4_3.M
Last changed : 9/7/2020 9:42:12 AM by SYSTEM
Analysis Method : E:\DATA\20200905\LC 2020-09-06 15-18-12\0IPA_50_4_3.M (Sequence Method)
Last changed : 9/8/2020 11:31:07 AM by SYSTEM
(modified after loading)
Additional Info : Peak(s) manually integrated

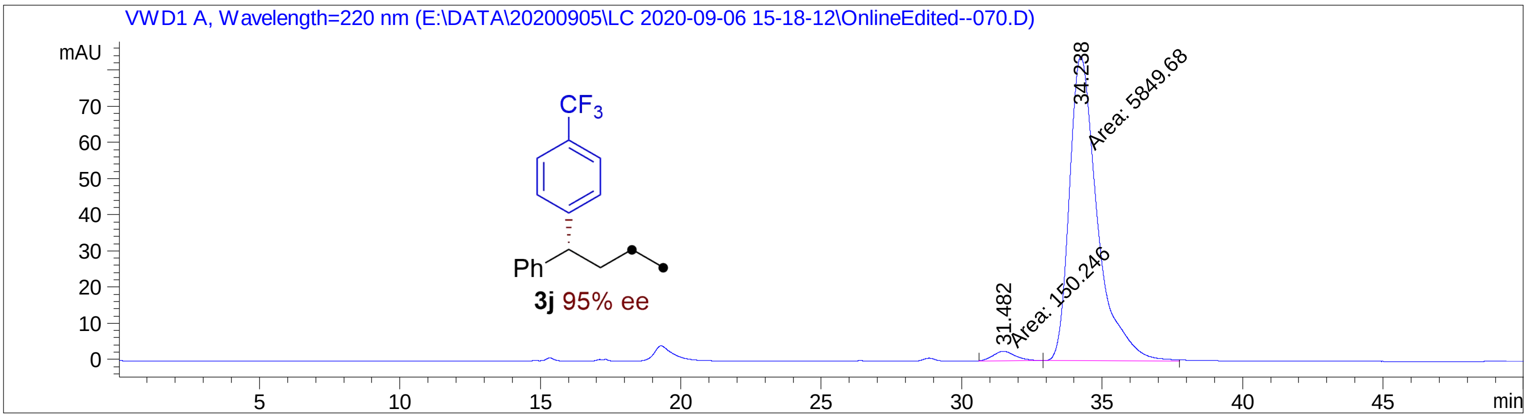

===
Area Percent Report
===

Sorted By : Signal
Multiplier : 1.0000
Dilution : 1.0000
Do not use Multiplier & Dilution Factor with ISTDs

Signal 1: VWD1 A, Wavelength=220 nm

Peak #	RetTime [min]	Type	Width [min]	Area [mAU*s]	Height [mAU]	Area %
1	31.482	FM	0.9381	150.24557	2.66936	2.5041
2	34.238	MF	1.1592	5849.67920	84.10281	97.4959

Totals : 5999.92477 86.77216

===
*** End of Report ***

Data File E:\DATA\20200823\LC 2020-08-26 16-46-12\OnlineEdited--015.D
Sample Name: YH-17-102-RAC

===

Acq. Operator : SYSTEM Seq. Line : 15
Acq. Instrument : HPLC1260 Location : P1-A1
Injection Date : 8/26/2020 10:26:09 PM Inj : 1
Inj Volume : 3.000 µl
Different Inj Volume from Sample Entry! Actual Inj Volume : 1.000 µl
Acq. Method : E:\DATA\20200823\LC 2020-08-26 16-46-12\1IPA_40_8_1.M
Last changed : 8/26/2020 10:49:00 PM by SYSTEM
(modified after loading)
Analysis Method : E:\DATA\20200823\LC 2020-08-26 16-46-12\1IPA_40_8_1.M (Sequence Method)
Last changed : 8/27/2020 10:35:59 AM by SYSTEM
(modified after loading)
Additional Info : Peak(s) manually integrated

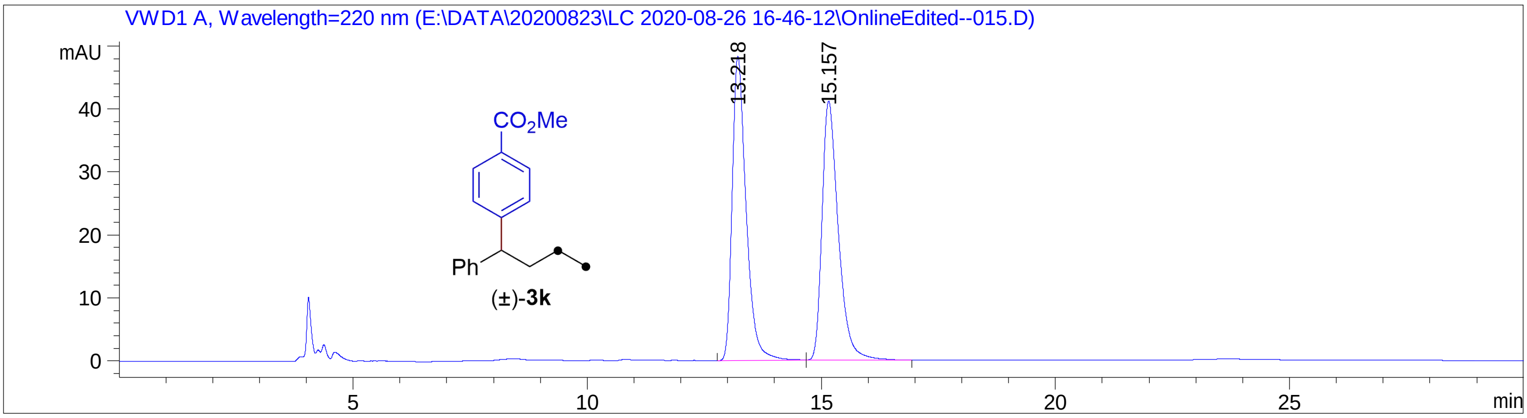

===

Area Percent Report

===

Sorted By : Signal
Multiplier : 1.0000
Dilution : 1.0000
Do not use Multiplier & Dilution Factor with ISTDs

Signal 1: VWD1 A, Wavelength=220 nm

Peak #	RetTime [min]	Type	Width [min]	Area [mAU*s]	Height [mAU]	Area %
1	13.218	BB	0.3101	997.52032	48.26073	50.0790
2	15.157	BB	0.3641	994.37354	41.12718	49.9210

Totals : 1991.89386 89.38791

===

*** End of Report ***

Data File E:\DATA\20200823\LC 2020-08-26 16-46-12\OnlineEdited--016.D
Sample Name: YH-17-102-EE

===

Acq. Operator : SYSTEM   Seq. Line : 16
Acq. Instrument : HPLC1260   Location : P1-A2
Injection Date : 8/26/2020 10:56:55 PM   Inj : 1
Inj Volume : 3.000 µl
Different Inj Volume from Sample Entry! Actual Inj Volume : 1.000 µl
Acq. Method : E:\DATA\20200823\LC 2020-08-26 16-46-12\1IPA_40_8_1.M
Last changed : 8/26/2020 10:49:00 PM by SYSTEM
Analysis Method : E:\DATA\20200823\LC 2020-08-26 16-46-12\1IPA_40_8_1.M (Sequence Method)
Last changed : 8/27/2020 10:35:59 AM by SYSTEM
(modified after loading)
Additional Info : Peak(s) manually integrated

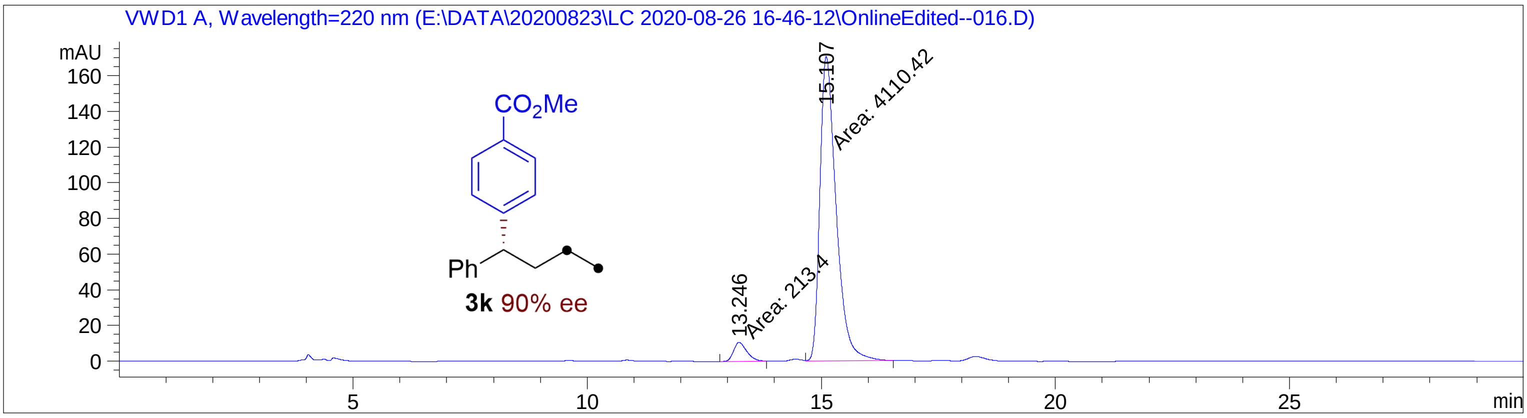

===

Area Percent Report

===

Sorted By : Signal
Multiplier : 1.0000
Dilution : 1.0000
Do not use Multiplier & Dilution Factor with ISTDs

Signal 1: VWD1 A, Wavelength=220 nm

Peak #	RetTime [min]	Type	Width [min]	Area [mAU*s]	Height [mAU]	Area %
1	13.246	MF	0.3319	213.40047	10.71743	4.9355
2	15.107	FM	0.4015	4110.41992	170.60970	95.0645

Totals : 4323.82039 181.32712

===

*** End of Report ***

Data File E:\DATA\20200905\LC 2020-09-06 15-18-12\OnlineEdited--133.D
Sample Name: YH-17-121-RAC

===

Acq. Operator : SYSTEM Seq. Line : 133
Acq. Instrument : HPLC1260 Location : P1-B1
Injection Date : 9/9/2020 10:12:47 AM Inj : 1
Inj Volume : 3.000 µl
Different Inj Volume from Sample Entry! Actual Inj Volume : 1.000 µl
Acq. Method : E:\DATA\20200905\LC 2020-09-06 15-18-12\5IPA IN PENTANE_40_5_4.M
Last changed : 9/9/2020 10:12:24 AM by SYSTEM
(modified after loading)
Analysis Method : E:\DATA\20200905\LC 2020-09-06 15-18-12\5IPA IN PENTANE_40_5_4.M (Sequence Method)
Last changed : 11/25/2021 5:03:12 PM by SYSTEM
(modified after loading)
Additional Info : Peak(s) manually integrated

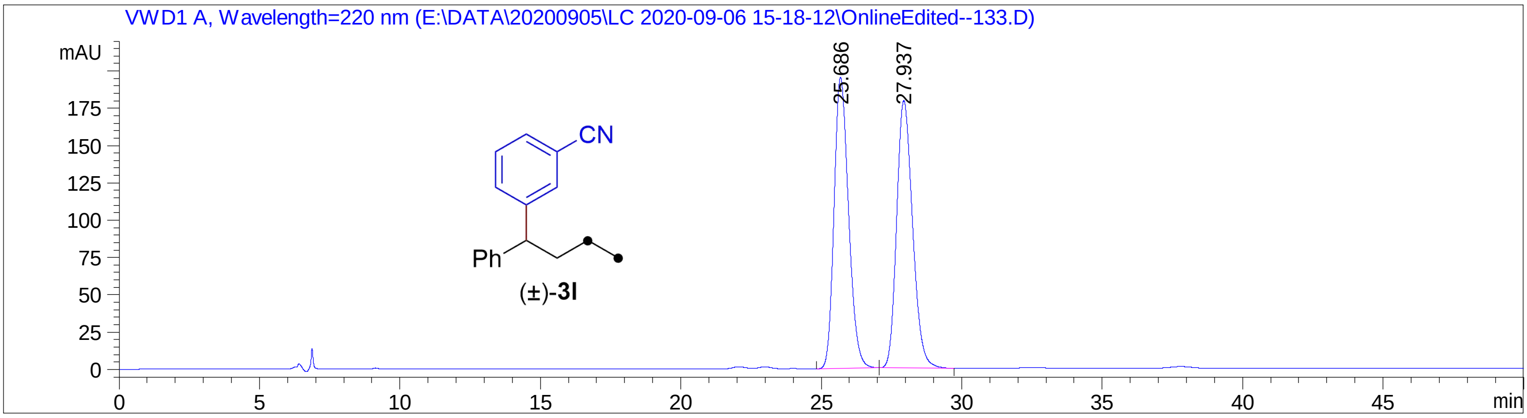

===
Area Percent Report
===

Sorted By : Signal
Multiplier : 1.0000
Dilution : 1.0000
Do not use Multiplier & Dilution Factor with ISTDs

Signal 1: VWD1 A, Wavelength=220 nm

Peak #	RetTime [min]	Type	Width [min]	Area [mAU*s]	Height [mAU]	Area %
1	25.686	BB	0.5548	7002.60303	195.05595	49.7788
2	27.937	BB	0.6088	7064.82617	179.27194	50.2212

Totals : 1.40674e4 374.32790

===
*** End of Report ***

Data File E:\DATA\20200905\LC 2020-09-06 15-18-12\OnlineEdited--134.D
Sample Name: YH-17-121-EE

===

Acq. Operator : SYSTEM Seq. Line : 134
Acq. Instrument : HPLC1260 Location : P1-B2
Injection Date : 9/9/2020 11:03:32 AM Inj : 1
Inj Volume : 3.000 µl
Different Inj Volume from Sample Entry! Actual Inj Volume : 1.000 µl
Acq. Method : E:\DATA\20200905\LC 2020-09-06 15-18-12\5IPA IN PENTANE_40_5_4.M
Last changed : 9/9/2020 10:12:24 AM by SYSTEM
Analysis Method : E:\DATA\20200905\LC 2020-09-06 15-18-12\5IPA IN PENTANE_40_5_4.M (Sequence Method)
Last changed : 11/25/2021 5:03:48 PM by SYSTEM
(modified after loading)
Additional Info : Peak(s) manually integrated

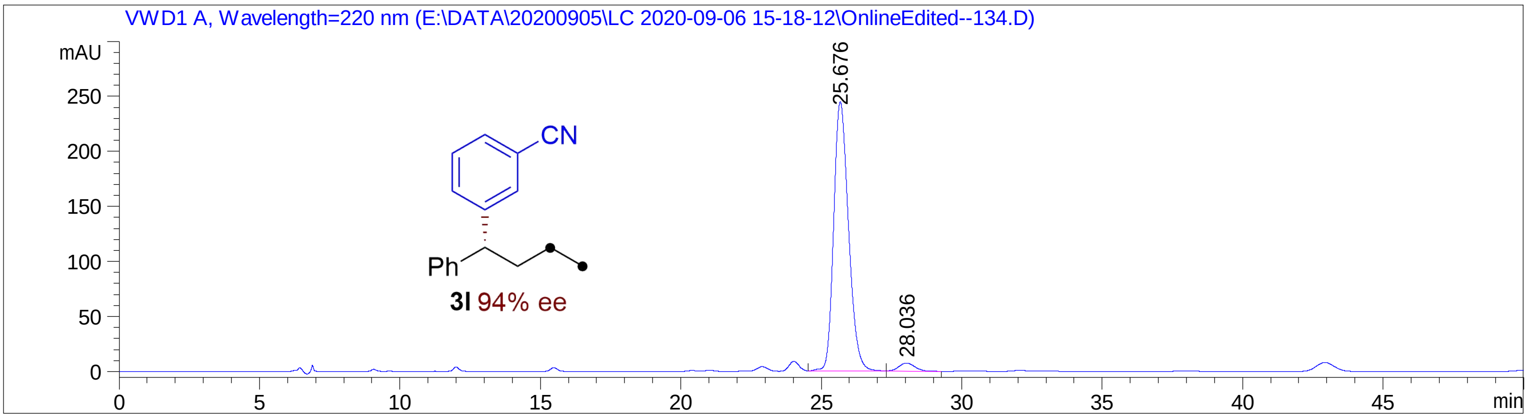

===

Area Percent Report

===

Sorted By : Signal
Multiplier : 1.0000
Dilution : 1.0000
Do not use Multiplier & Dilution Factor with ISTDs

Signal 1: VWD1 A, Wavelength=220 nm

Peak #	RetTime [min]	Type	Width [min]	Area [mAU*s]	Height [mAU]	Area %
1	25.676	VB	0.5684	8981.67285	244.61131	96.9631
2	28.036	BB	0.5930	281.30905	7.29287	3.0369

Totals : 9262.98190 251.90418

===

*** End of Report ***

Data File E:\DATA\20200831\LC 2020-08-31 17-14-33\OnlineEdited--021.D
Sample Name: YH-17-118-RAC

===

Acq. Operator : SYSTEM Seq. Line : 21
Acq. Instrument : HPLC1260 Location : P1-B3
Injection Date : 9/1/2020 4:47:15 AM Inj : 1
Inj Volume : 3.000 µl
Different Inj Volume from Sample Entry! Actual Inj Volume : 1.000 µl
Acq. Method : E:\DATA\20200831\LC 2020-08-31 17-14-33\20IPA_20_0.8_1.M
Last changed : 8/31/2020 10:07:17 PM by SYSTEM
Analysis Method : E:\DATA\20200831\LC 2020-08-31 17-14-33\20IPA_20_0.8_1.M (Sequence Method)
Last changed : 9/1/2020 8:54:30 AM by SYSTEM
(modified after loading)
Additional Info : Peak(s) manually integrated

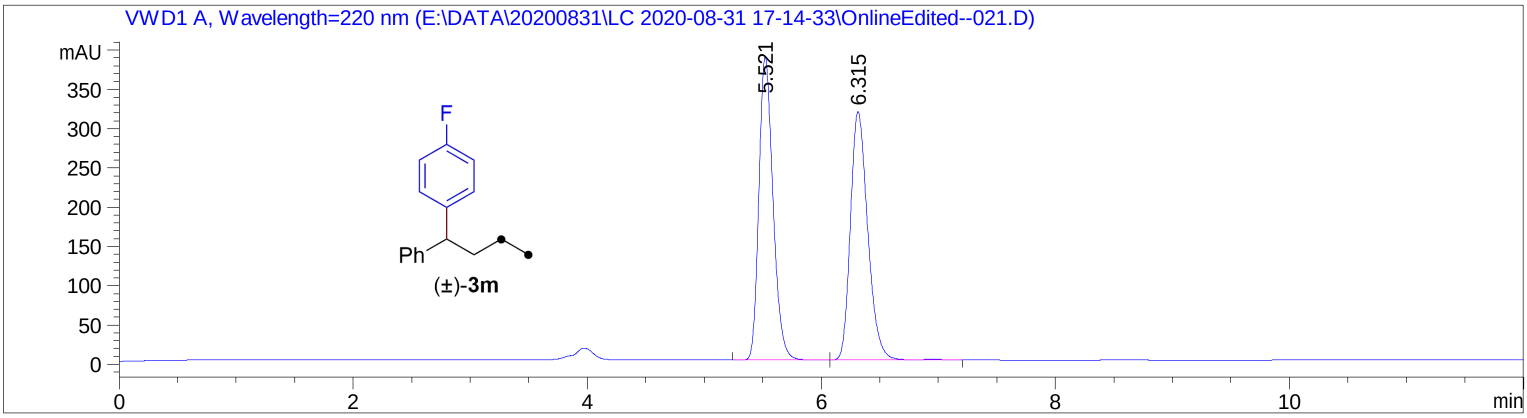

===
Area Percent Report
===

Sorted By : Signal
Multiplier : 1.0000
Dilution : 1.0000
Do not use Multiplier & Dilution Factor with ISTDs

Signal 1: VWD1 A, Wavelength=220 nm

Peak #	RetTime [min]	Type	Width [min]	Area [mAU*s]	Height [mAU]	Area %
1	5.521	BB	0.1286	3231.76294	386.16724	49.8112
2	6.315	BV R	0.1582	3256.26318	316.06775	50.1888

Totals : 6488.02612 702.23499

===
*** End of Report ***

Data File E:\DATA\20200831\LC 2020-08-31 17-14-33\OnlineEdited--022.D
Sample Name: YH-17-118-EE

===

Acq. Operator : SYSTEM　　Seq. Line : 22
Acq. Instrument : HPLC1260　　Location : P1-B4
Injection Date : 9/1/2020 5:08:01 AM　　Inj : 1
Inj Volume : 3.000 µl
Different Inj Volume from Sample Entry! Actual Inj Volume : 1.000 µl
Acq. Method : E:\DATA\20200831\LC 2020-08-31 17-14-33\20IPA_20_0.8_1.M
Last changed : 8/31/2020 10:07:17 PM by SYSTEM
Analysis Method : E:\DATA\20200831\LC 2020-08-31 17-14-33\20IPA_20_0.8_1.M (Sequence Method)
Last changed : 9/1/2020 8:54:30 AM by SYSTEM
(modified after loading)
Additional Info : Peak(s) manually integrated

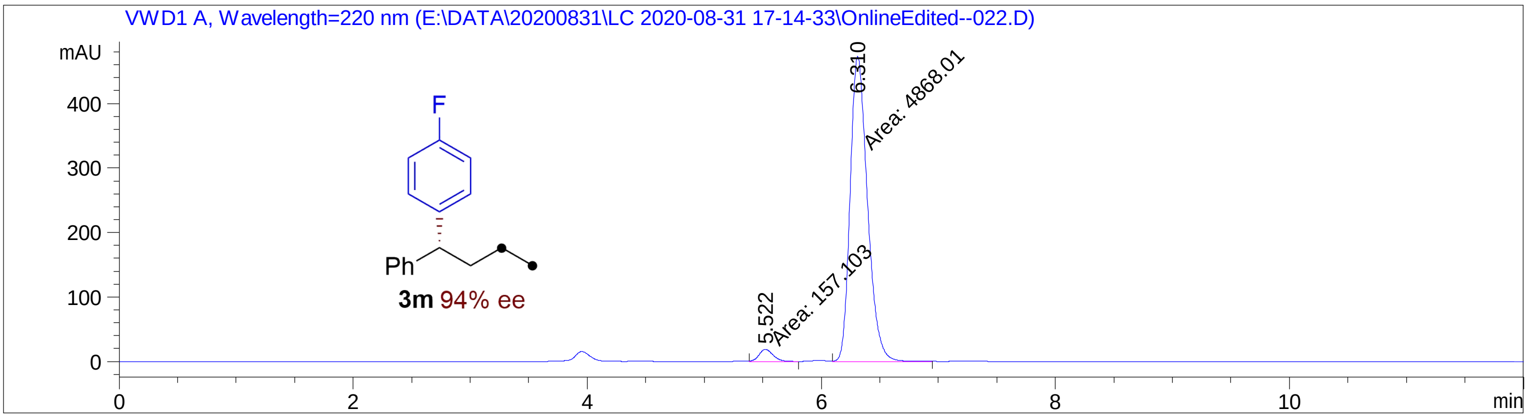

===

Area Percent Report

===

Sorted By : Signal
Multiplier : 1.0000
Dilution : 1.0000
Do not use Multiplier & Dilution Factor with ISTDs

Signal 1: VWD1 A, Wavelength=220 nm

Peak #	RetTime [min]	Type	Width [min]	Area [mAU*s]	Height [mAU]	Area %
1	5.522	FM	0.1391	157.10300	18.82444	3.1264
2	6.310	MF	0.1716	4868.01270	472.90283	96.8736

Totals : 5025.11569 491.72727

===

*** End of Report ***

Data File E:\DATA\20200828\LC 2020-08-29 10-07-26\OnlineEdited--006.D
Sample Name: YH-17-111-RAC

===

Acq. Operator	: SYSTEM	Seq. Line :	6
Acq. Instrument	: HPLC1260	Location :	P1-A3
Injection Date	: 8/29/2020 1:38:25 PM	Inj :	1
		Inj Volume :	3.000 µl
Different Inj Volume from Sample Entry!		Actual Inj Volume :	0.500 µl

Acq. Method : E:\DATA\20200828\LC 2020-08-29 10-07-26\5IPA_20_10_1.M
Last changed : 8/29/2020 10:48:42 AM by SYSTEM
Analysis Method : E:\DATA\20200828\LC 2020-08-29 10-07-26\5IPA_20_10_1.M (Sequence Method)
Last changed : 8/29/2020 3:25:50 PM by SYSTEM
(modified after loading)
Additional Info : Peak(s) manually integrated

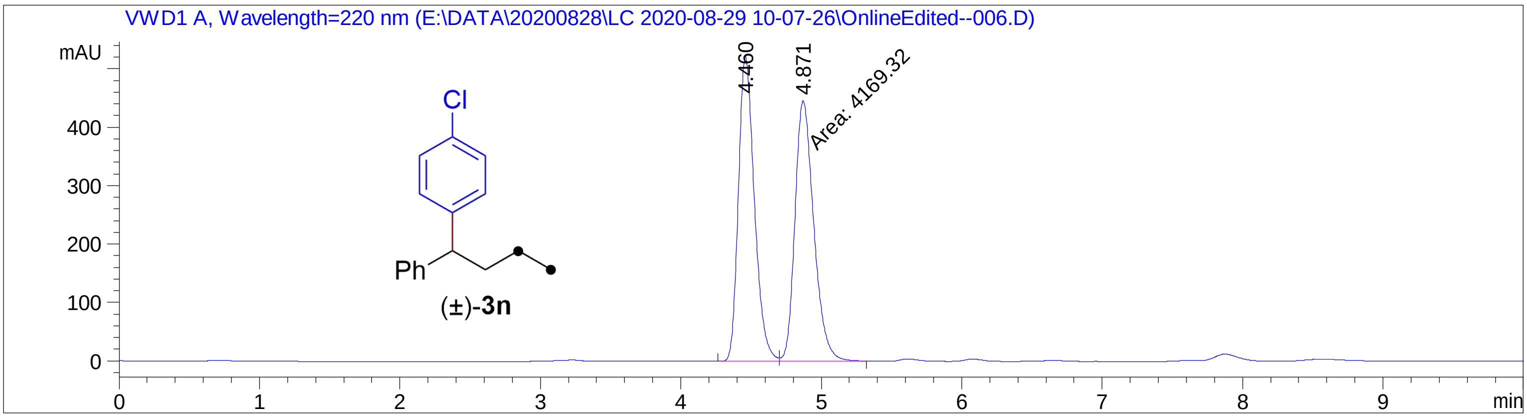

===

Area Percent Report

===

Sorted By : Signal
Multiplier : 1.0000
Dilution : 1.0000
Do not use Multiplier & Dilution Factor with ISTDs

Signal 1: VWD1 A, Wavelength=220 nm

Peak #	RetTime [min]	Type	Width [min]	Area [mAU*s]	Height [mAU]	Area %
1	4.460	BV	0.1215	4133.57275	521.56091	49.7847
2	4.871	MF	0.1557	4169.31836	446.18970	50.2153
Totals :				8302.89111	967.75061	

===

*** End of Report ***

Data File E:\DATA\20200828\LC 2020-08-29 10-07-26\OnlineEdited--007.D
Sample Name: YH-17-111-EE

===

Acq. Operator   : SYSTEM   Seq. Line :   7
Acq. Instrument : HPLC1260   Location :   P1-A4
Injection Date : 8/29/2020 1:59:10 PM   Inj :   1
                                           Inj Volume : 3.000 µl
Different Inj Volume from Sample Entry! Actual Inj Volume : 1.000 µl
Acq. Method   : E:\DATA\20200828\LC 2020-08-29 10-07-26\5IPA_20_10_1.M
Last changed   : 8/29/2020 2:14:17 PM by SYSTEM
                  (modified after loading)
Analysis Method : E:\DATA\20200828\LC 2020-08-29 10-07-26\5IPA_20_10_1.M (Sequence Method)
Last changed   : 8/29/2020 3:26:45 PM by SYSTEM
                  (modified after loading)
Additional Info : Peak(s) manually integrated

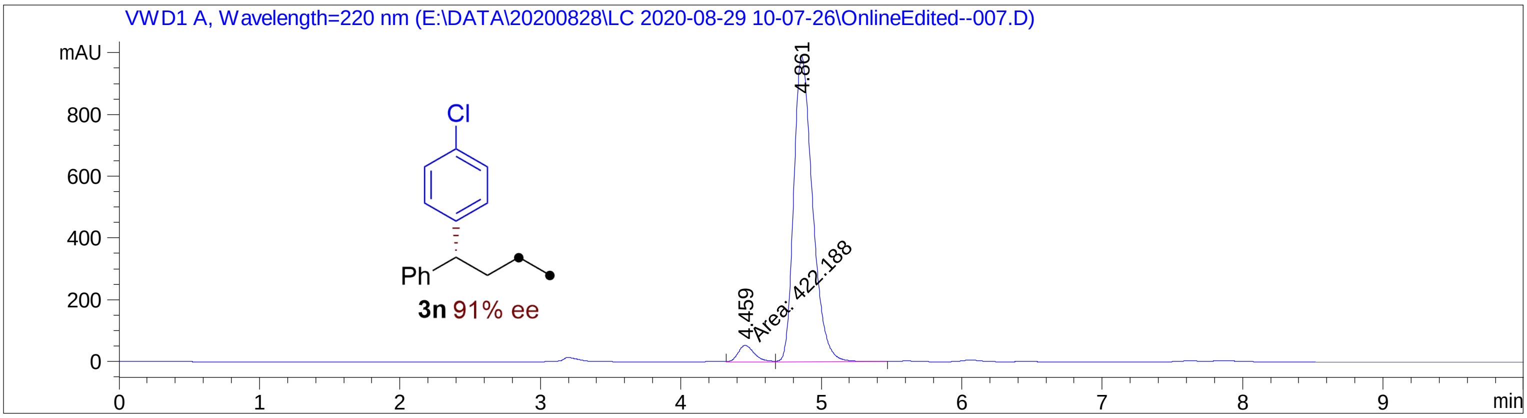

===
Area Percent Report
===

Sorted By : Signal
Multiplier : 1.0000
Dilution : 1.0000
Do not use Multiplier & Dilution Factor with ISTDs

Signal 1: VWD1 A, Wavelength=220 nm

Peak #	RetTime [min]	Type	Width [min]	Area [mAU*s]	Height [mAU]	Area %
1	4.459	FM	0.1327	422.18765	53.02592	4.3768
2	4.861	VB	0.1428	9223.73828	988.79614	95.6232

Totals : 9645.92593 1041.82207

===
*** End of Report ***

Data File E:\DATA\20200831\LC 2020-09-04 14-19-23\OnlineEdited--014.D
Sample Name: YH-17-125-RAC

===

Acq. Operator : SYSTEM   Seq. Line : 14
Acq. Instrument : HPLC1260   Location : P1-A1
Injection Date : 9/4/2020 6:46:37 PM   Inj : 1
Inj Volume : 3.000 µl
Different Inj Volume from Sample Entry! Actual Inj Volume : 1.000 µl
Acq. Method : E:\DATA\20200831\LC 2020-09-04 14-19-23\1IPA_20_8_3.M
Last changed : 9/4/2020 6:17:46 PM by SYSTEM
Analysis Method : E:\DATA\20200831\LC 2020-09-04 14-19-23\1IPA_20_8_3.M (Sequence Method)
Last changed : 9/4/2020 7:31:13 PM by SYSTEM
(modified after loading)
Additional Info : Peak(s) manually integrated

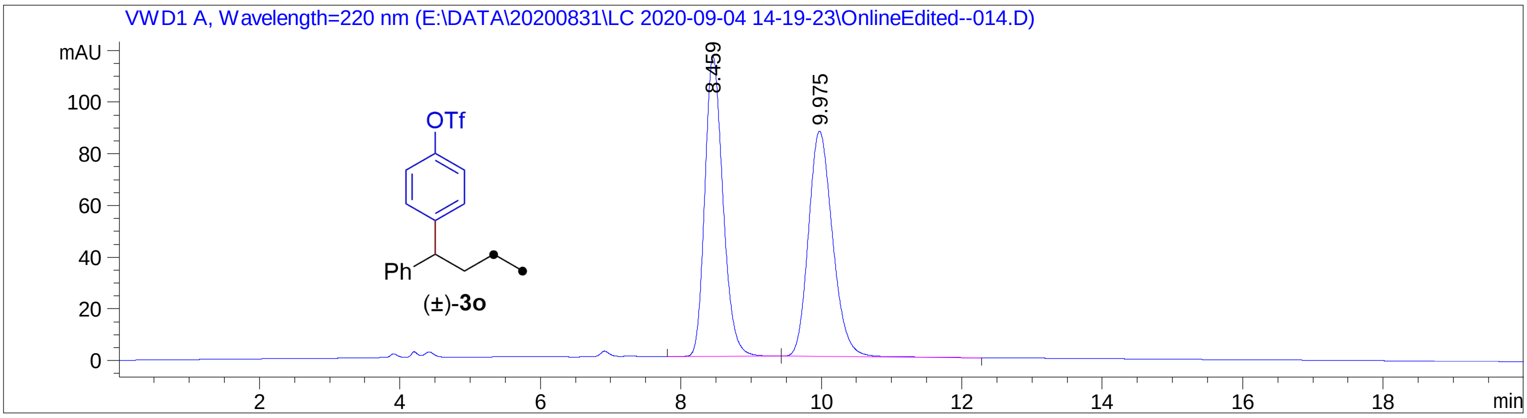

===

Area Percent Report

===

Sorted By : Signal
Multiplier : 1.0000
Dilution : 1.0000
Do not use Multiplier & Dilution Factor with ISTDs

Signal 1: VWD1 A, Wavelength=220 nm

Peak #	RetTime [min]	Type	Width [min]	Area [mAU*s]	Height [mAU]	Area %
1	8.459	BB	0.2765	2082.61841	115.99145	49.9658
2	9.975	BB	0.3689	2085.46729	87.23469	50.0342

Totals : 4168.08569 203.22614

===

*** End of Report ***

Data File E:\DATA\20200831\LC 2020-09-04 14-19-23\OnlineEdited--015.D
Sample Name: YH-17-125-EE

===

Acq. Operator : SYSTEM Seq. Line : 15
Acq. Instrument : HPLC1260 Location : P1-A2
Injection Date : 9/4/2020 7:07:23 PM Inj : 1
Inj Volume : 3.000 µl
Different Inj Volume from Sample Entry! Actual Inj Volume : 1.000 µl
Acq. Method : E:\DATA\20200831\LC 2020-09-04 14-19-23\1IPA_20_8_3.M
Last changed : 9/4/2020 6:17:46 PM by SYSTEM
Analysis Method : E:\DATA\20200831\LC 2020-09-04 14-19-23\1IPA_20_8_3.M (Sequence Method)
Last changed : 9/4/2020 7:31:13 PM by SYSTEM
(modified after loading)
Additional Info : Peak(s) manually integrated

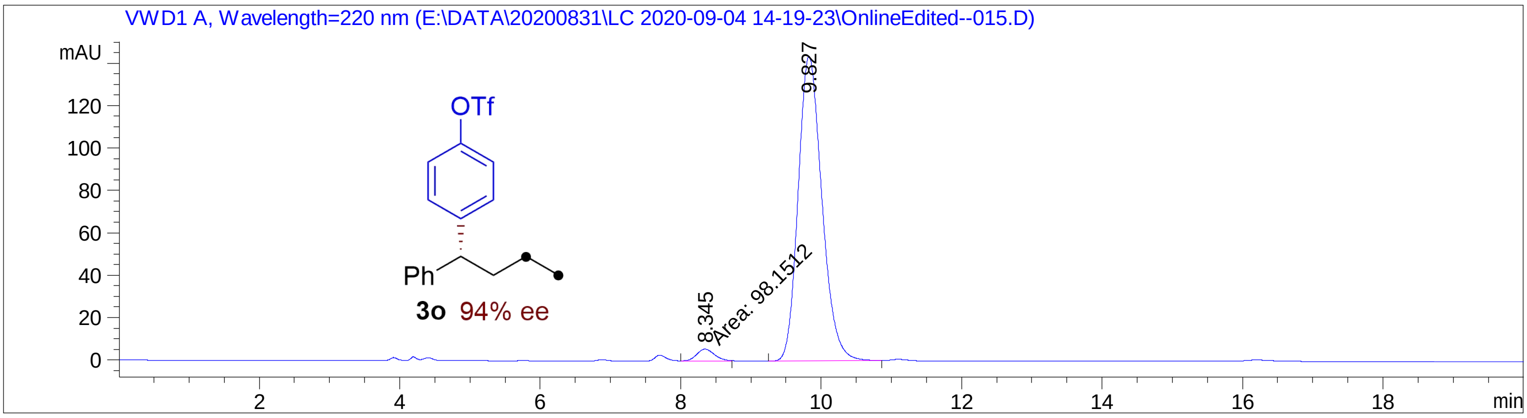

===

Area Percent Report

===

Sorted By : Signal
Multiplier : 1.0000
Dilution : 1.0000
Do not use Multiplier & Dilution Factor with ISTDs

Signal 1: VWD1 A, Wavelength=220 nm

Peak #	RetTime [min]	Type	Width [min]	Area [mAU*s]	Height [mAU]	Area %
1	8.345	MF	0.2935	98.15123	5.57303	2.8188
2	9.827	BB	0.3639	3383.88989	143.63464	97.1812

Totals : 3482.04112 149.20767

===

*** End of Report ***

Data File E:\DATA\20200823\LC 2020-08-26 16-46-12\1AC-0601--007.D
Sample Name: YH-17-103-EE

===

Acq. Operator : SYSTEM   Seq. Line : 7
Acq. Instrument : HPLC1260   Location : P1-A3
Injection Date : 8/26/2020 7:22:51 PM   Inj : 1
Inj Volume : 3.000 µl
Different Inj Volume from Sample Entry! Actual Inj Volume : 1.000 µl
Acq. Method : E:\DATA\20200823\LC 2020-08-26 16-46-12\10EtOH_25_8_1.M
Last changed : 8/26/2020 7:41:02 PM by SYSTEM
(modified after loading)
Analysis Method : E:\DATA\20200823\LC 2020-08-26 16-46-12\10EtOH_25_8_1.M (Sequence Method)
Last changed : 8/26/2020 8:10:22 PM by SYSTEM
(modified after loading)
Additional Info : Peak(s) manually integrated

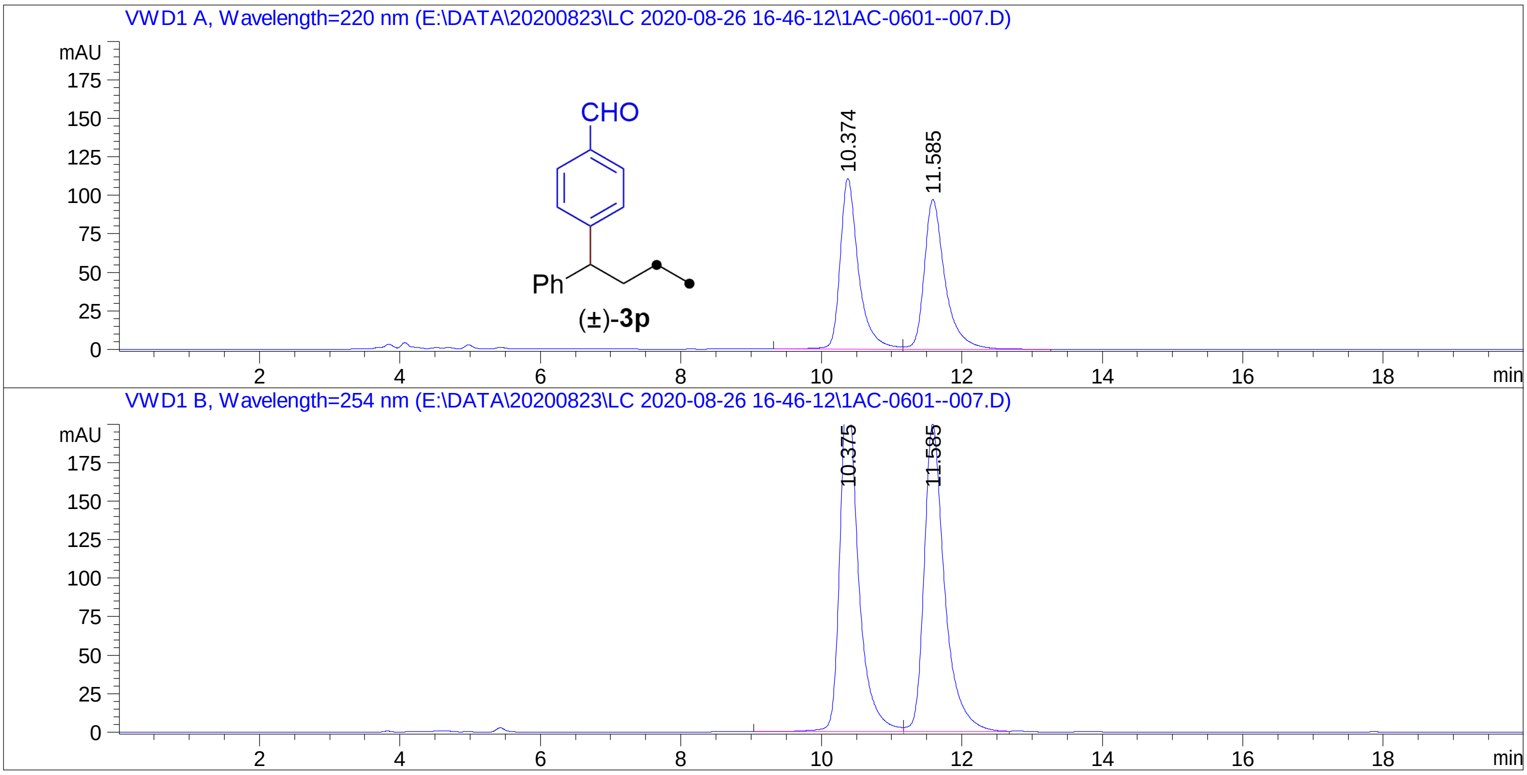

===

Area Percent Report

===

Sorted By : Signal
Multiplier : 1.0000
Dilution : 1.0000
Do not use Multiplier & Dilution Factor with ISTDs

Signal 1: VWD1 A, Wavelength=220 nm

Peak #	RetTime [min]	Type	Width [min]	Area [mAU*s]	Height [mAU]	Area %
1	10.374	BV	0.2695	2007.94751	110.75328	50.1692
2	11.585	VB	0.3057	1994.40405	97.07459	49.8308

Totals : 4002.35156 207.82787

Signal 2: VWD1 B, Wavelength=254 nm

Peak #	RetTime [min]	Type	Width [min]	Area [mAU*s]	Height [mAU]	Area %
1	10.375	BV	0.2672	4089.62842	228.06958	50.3680
2	11.585	VB	0.3019	4029.86670	199.26624	49.6320

Totals : 8119.49512 427.33582

==

*** End of Report ***

Data File E:\DATA\20200823\LC 2020-08-26 16-46-12\OnlineEdited--008.D
Sample Name: YH-17-103-EE-EE

===

Acq. Operator : SYSTEM Seq. Line : 8
Acq. Instrument : HPLC1260 Location : P1-A4
Injection Date : 8/26/2020 7:43:38 PM Inj : 1
Inj Volume : 3.000 µl
Different Inj Volume from Sample Entry! Actual Inj Volume : 1.000 µl
Acq. Method : E:\DATA\20200823\LC 2020-08-26 16-46-12\10EtOH_25_8_1.M
Last changed : 8/26/2020 7:41:02 PM by SYSTEM
Analysis Method : E:\DATA\20200823\LC 2020-08-26 16-46-12\10EtOH_25_8_1.M (Sequence Method)
Last changed : 8/26/2020 8:10:22 PM by SYSTEM
(modified after loading)
Additional Info : Peak(s) manually integrated

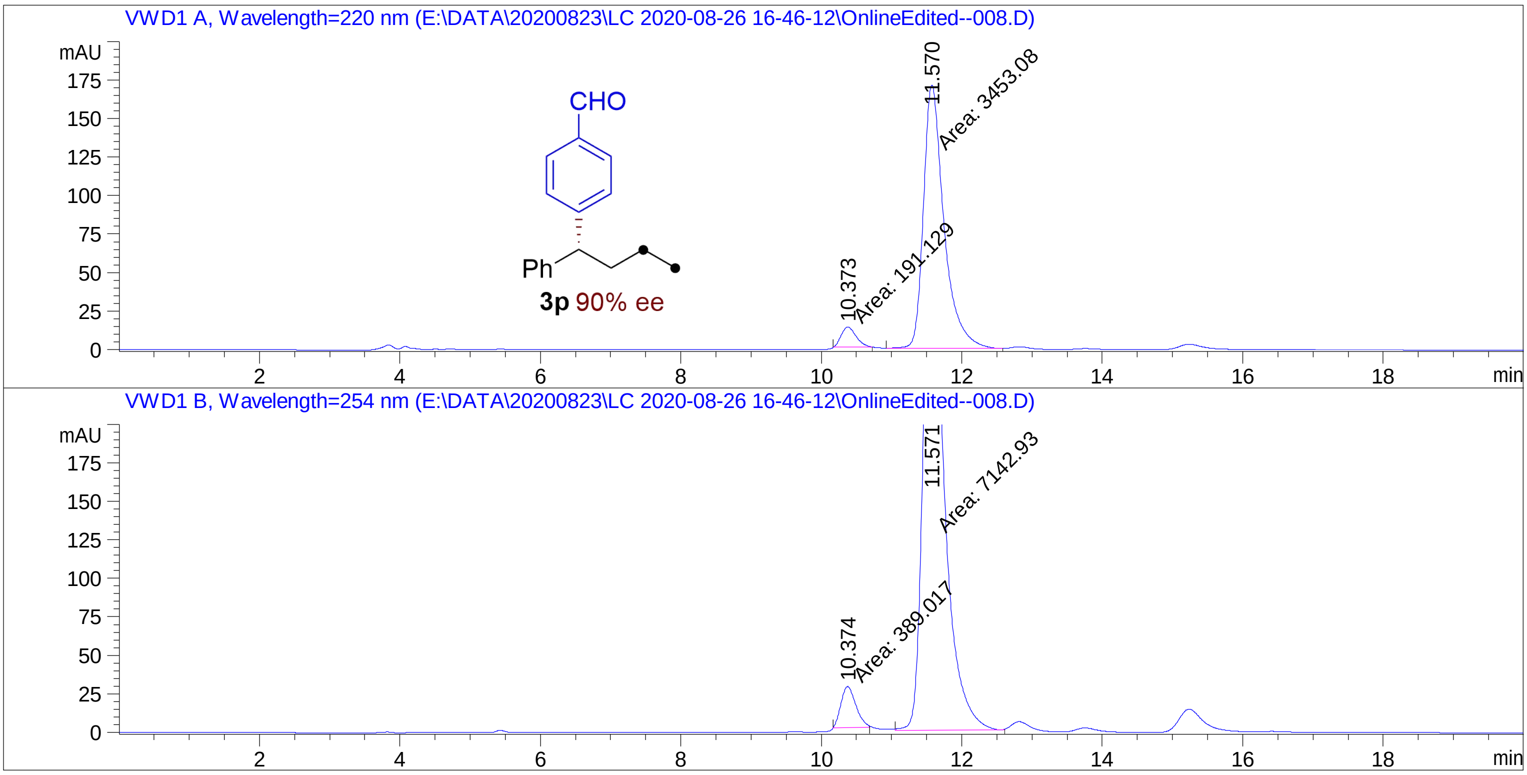

===
Area Percent Report
===

Sorted By : Signal
Multiplier : 1.0000
Dilution : 1.0000
Do not use Multiplier & Dilution Factor with ISTDs

Signal 1: VWD1 A, Wavelength=220 nm

Peak #	RetTime [min]	Type	Width [min]	Area [mAU*s]	Height [mAU]	Area %
1	10.373	MM	0.2455	191.12875	12.97701	5.2447
2	11.570	MM	0.3368	3453.07617	170.87949	94.7553

Totals : 3644.20493 183.85650

Signal 2: VWD1 B, Wavelength=254 nm

Peak #	RetTime [min]	Type	Width [min]	Area [mAU*s]	Height [mAU]	Area %
1	10.374	MM	0.2447	389.01724	26.49381	5.1649
2	11.571	MM	0.3337	7142.92676	356.73471	94.8351
Totals :				7531.94400	383.22852	

===

*** End of Report ***

Data File E:\DATA\20200831\LC 2020-09-01 21-08-08\OnlineEdited--003.D
Sample Name: YH-17-123-RAC

===

Acq. Operator : SYSTEM Seq. Line : 3
Acq. Instrument : HPLC1260 Location : P1-A3
Injection Date : 9/1/2020 9:51:22 PM Inj : 1
Inj Volume : 3.000 µl
Different Inj Volume from Sample Entry! Actual Inj Volume : 1.000 µl
Acq. Method : E:\DATA\20200831\LC 2020-09-01 21-08-08\10EtOH_25_8_1.M
Last changed : 9/1/2020 9:22:16 PM by SYSTEM
Analysis Method : E:\DATA\20200831\LC 2020-09-01 21-08-08\10EtOH_25_8_1.M (Sequence Method)
Last changed : 9/1/2020 10:37:13 PM by SYSTEM
(modified after loading)
Additional Info : Peak(s) manually integrated

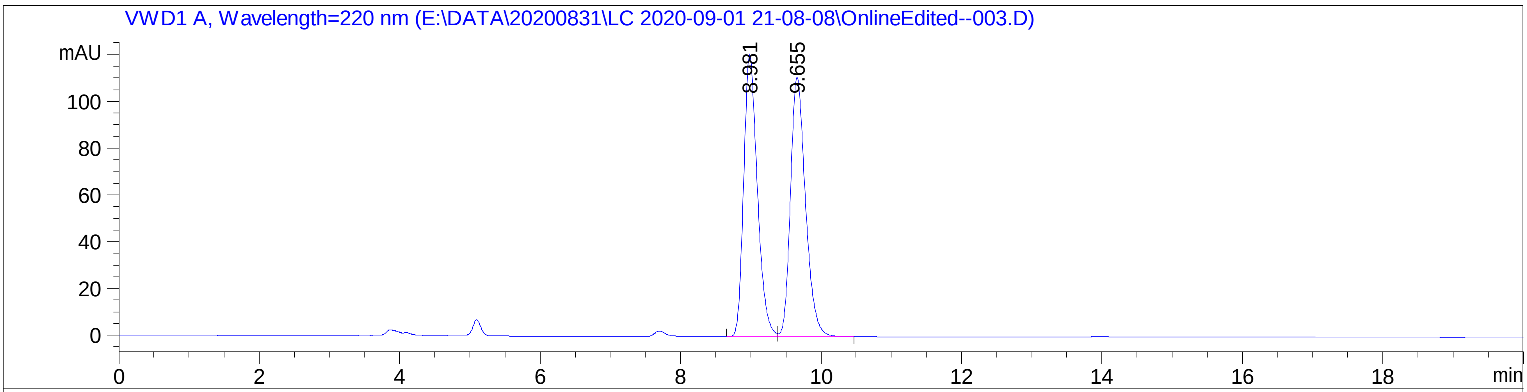

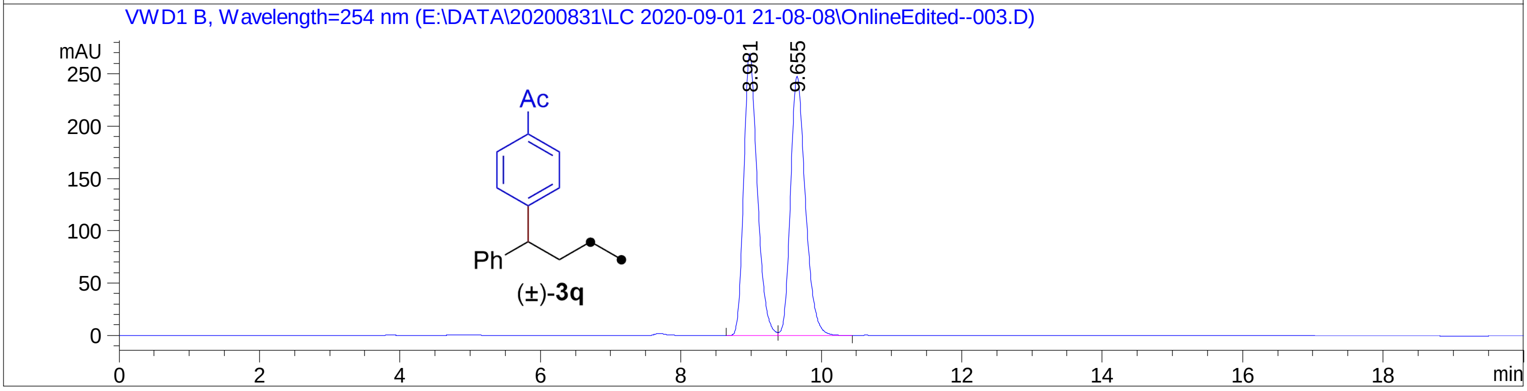

===

Area Percent Report

===

Sorted By : Signal
Multiplier : 1.0000
Dilution : 1.0000
Do not use Multiplier & Dilution Factor with ISTDs

Signal 1: VWD1 A, Wavelength=220 nm

Peak #	RetTime [min]	Type	Width [min]	Area [mAU*s]	Height [mAU]	Area %
1	8.981	BV	0.2086	1632.90027	120.14957	49.8140
2	9.655	VB	0.2270	1645.09265	110.95697	50.1860

Totals : 3277.99292 231.10654

Signal 2: VWD1 B, Wavelength=254 nm

Peak #	RetTime [min]	Type	Width [min]	Area [mAU*s]	Height [mAU]	Area %
1	8.981	BV	0.2077	3637.07153	269.06482	49.8745
2	9.655	VB	0.2260	3655.37085	247.92865	50.1255

Totals : 7292.44238 516.99347

===

*** End of Report ***

Data File E:\DATA\20200831\LC 2020-09-01 21-08-08\OnlineEdited--004.D
Sample Name: YH-17-123-EE

===

Acq. Operator : SYSTEM Seq. Line : 4
Acq. Instrument : HPLC1260 Location : P1-A4
Injection Date : 9/1/2020 10:17:09 PM Inj : 1
Inj Volume : 3.000 µl
Different Inj Volume from Sample Entry! Actual Inj Volume : 0.800 µl
Acq. Method : E:\DATA\20200831\LC 2020-09-01 21-08-08\10EtOH_25_8_1.M
Last changed : 9/1/2020 10:36:44 PM by SYSTEM
(modified after loading)
Analysis Method : E:\DATA\20200831\LC 2020-09-01 21-08-08\10EtOH_25_8_1.M (Sequence Method)
Last changed : 9/1/2020 10:40:37 PM by SYSTEM
(modified after loading)
Additional Info : Peak(s) manually integrated

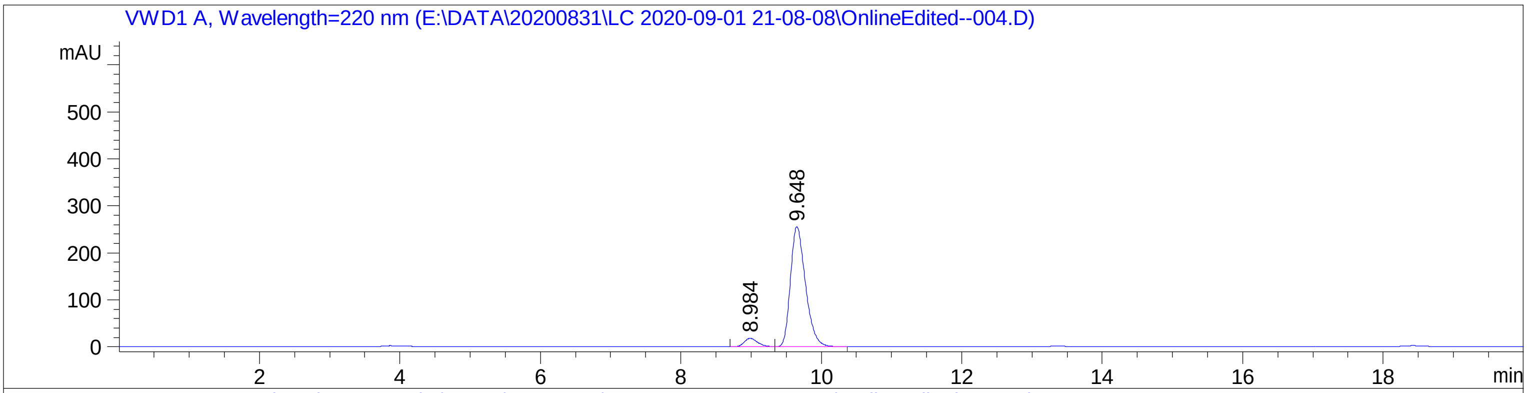

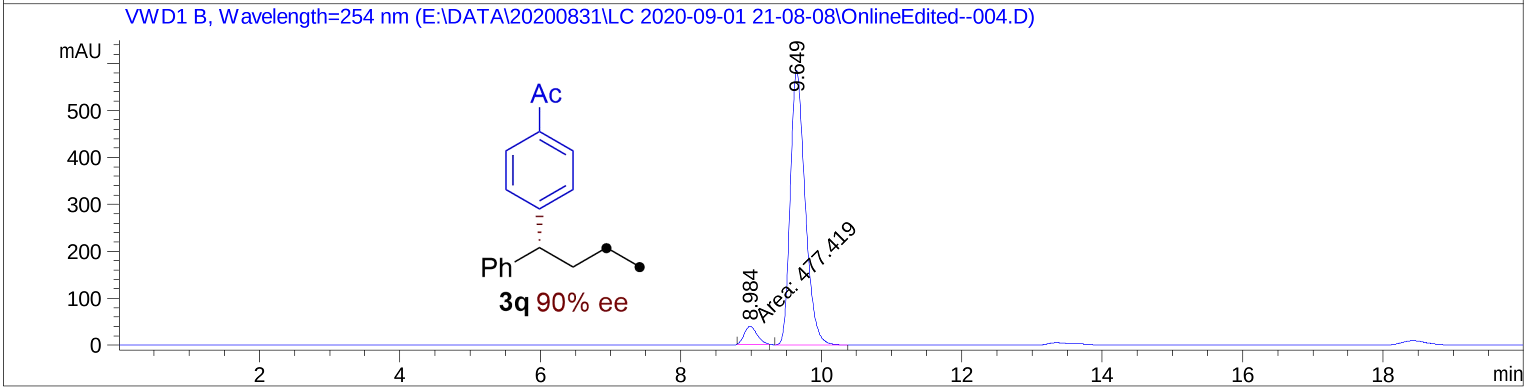

===

Area Percent Report

===

Sorted By : Signal
Multiplier : 1.0000
Dilution : 1.0000
Do not use Multiplier & Dilution Factor with ISTDs

Signal 1: VWD1 A, Wavelength=220 nm

Peak #	RetTime [min]	Type	Width [min]	Area [mAU*s]	Height [mAU]	Area %
1	8.984	BV	0.2057	243.22253	18.21900	6.0116
2	9.648	VB	0.2278	3802.66626	255.24548	93.9884

Totals : 4045.88879 273.46448

Signal 2: VWD1 B, Wavelength=254 nm

Peak #	RetTime [min]	Type	Width [min]	Area [mAU*s]	Height [mAU]	Area %
1	8.984	MM	0.2075	477.41904	38.34625	5.2539
2	9.649	VB	0.2259	8609.58887	584.47095	94.7461

Totals : 9087.00790 622.81720

==

*** End of Report ***

Data File E:\DATA\20200829\LC 2020-08-29 17-33-08\OnlineEdited--023.D
Sample Name: YH-17-113-RAC

===
Acq. Operator   : SYSTEM   Seq. Line : 23
Acq. Instrument : HPLC1260   Location :   P1-A3
Injection Date : 8/30/2020 4:27:17 AM   Inj :   1
                                                Inj Volume : 3.000 µl
Different Inj Volume from Sample Entry! Actual Inj Volume : 0.200 µl
Method   : E:\DATA\20200829\LC 2020-08-29 17-33-08\1EtOH_25_5_2.M (Sequence Method)
Last changed   : 8/29/2020 9:51:08 PM by SYSTEM
Additional Info : Peak(s) manually integrated

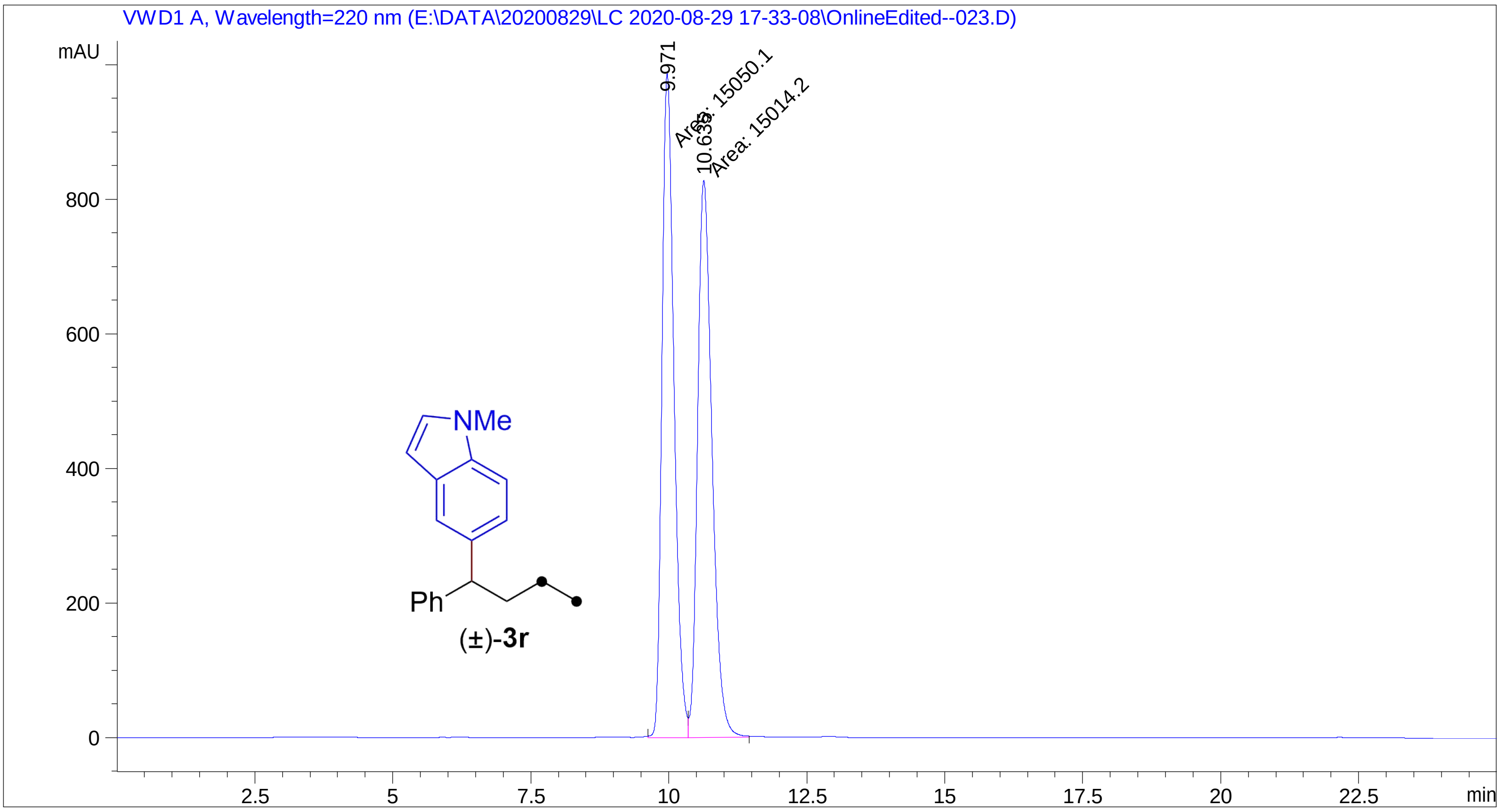

===
Area Percent Report
===

Sorted By : Signal
Multiplier : 1.0000
Dilution : 1.0000
Use Multiplier & Dilution Factor with ISTDs

Signal 1: VWD1 A, Wavelength=220 nm

Peak #	RetTime [min]	Type	Width [min]	Area [mAU*s]	Height [mAU]	Area %
1	9.971	FM	0.2544	1.50501e4	986.08356	50.0596
2	10.635	MF	0.3023	1.50142e4	827.73993	49.9404

Totals : 3.00643e4 1813.82349

===
*** End of Report ***

Data File E:\DATA\20200829\LC 2020-08-29 17-33-08\OnlineEdited--024.D
Sample Name: YH-17-113-EE

===
Acq. Operator : SYSTEM Seq. Line : 24
Acq. Instrument : HPLC1260 Location : P1-A4
Injection Date : 8/30/2020 4:53:01 AM Inj : 1
Inj Volume : 3.000 µl
Different Inj Volume from Sample Entry! Actual Inj Volume : 0.500 µl
Method : E:\DATA\20200829\LC 2020-08-29 17-33-08\1EtOH_25_5_2.M (Sequence Method)
Last changed : 8/29/2020 9:51:08 PM by SYSTEM
Additional Info : Peak(s) manually integrated

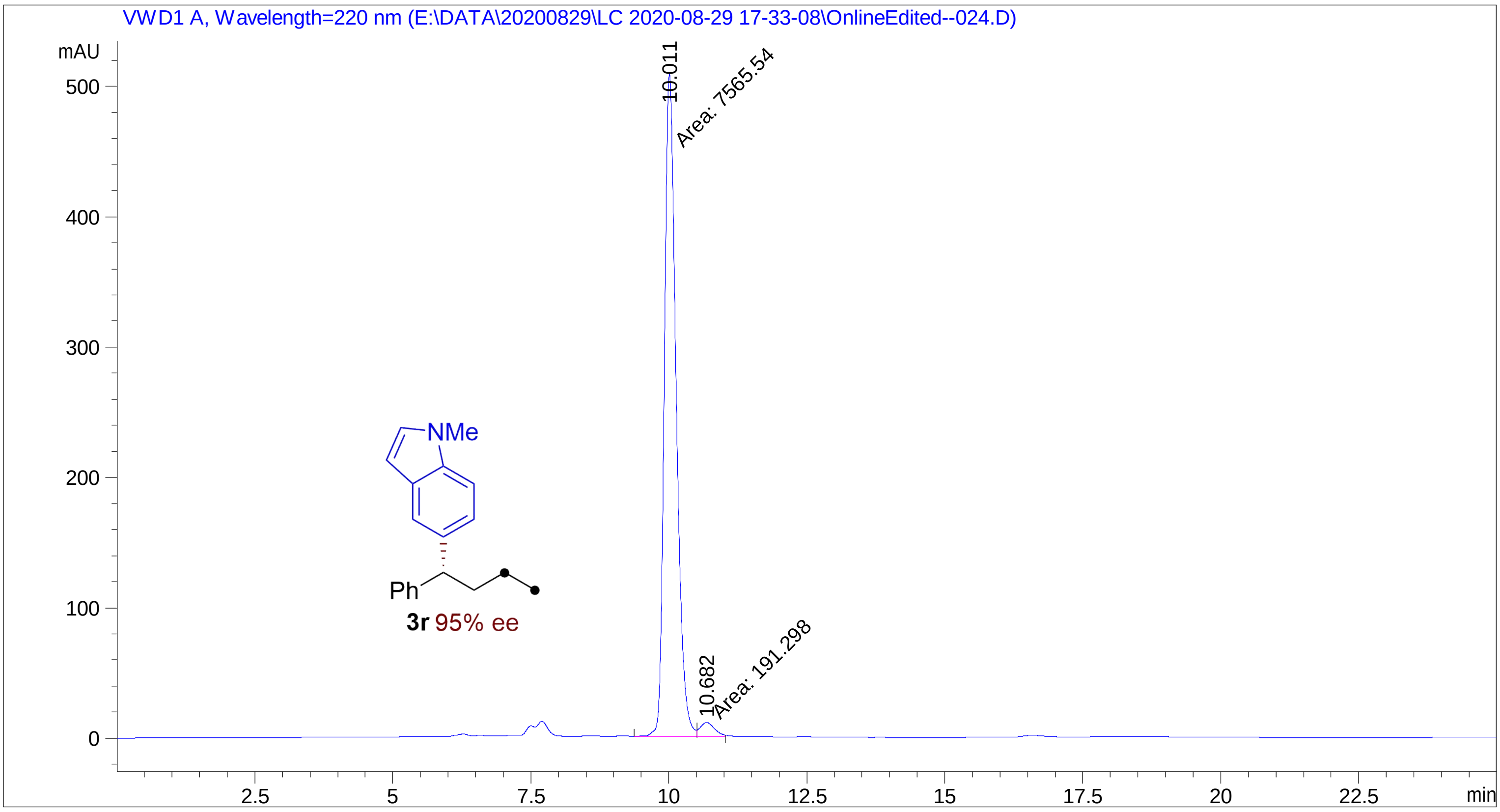

===
Area Percent Report
===

Sorted By : Signal
Multiplier : 1.0000
Dilution : 1.0000
Use Multiplier & Dilution Factor with ISTDs

Signal 1: VWD1 A, Wavelength=220 nm

Peak #	RetTime [min]	Type	Width [min]	Area [mAU*s]	Height [mAU]	Area %
1	10.011	MF	0.2481	7565.53662	508.18222	97.5338
2	10.682	MF	0.2979	191.29788	10.70320	2.4662

Totals : 7756.83450 518.88542

===
*** End of Report ***

Data File E:\DATA\20200823\LC 2020-08-26 16-46-12\OnlineEdited--012.D
Sample Name: YH-17-104-RAC

===

Acq. Operator : SYSTEM Seq. Line : 12
Acq. Instrument : HPLC1260 Location : P1-A5
Injection Date : 8/26/2020 9:09:08 PM Inj : 1
Inj Volume : 3.000 µl
Different Inj Volume from Sample Entry! Actual Inj Volume : 1.000 µl
Acq. Method : E:\DATA\20200823\LC 2020-08-26 16-46-12\5IPA_20_8_2.M
Last changed : 8/26/2020 8:31:07 PM by SYSTEM
Analysis Method : E:\DATA\20200823\LC 2020-08-26 16-46-12\5IPA_20_8_2.M (Sequence Method)
Last changed : 8/26/2020 9:46:16 PM by SYSTEM
(modified after loading)
Additional Info : Peak(s) manually integrated

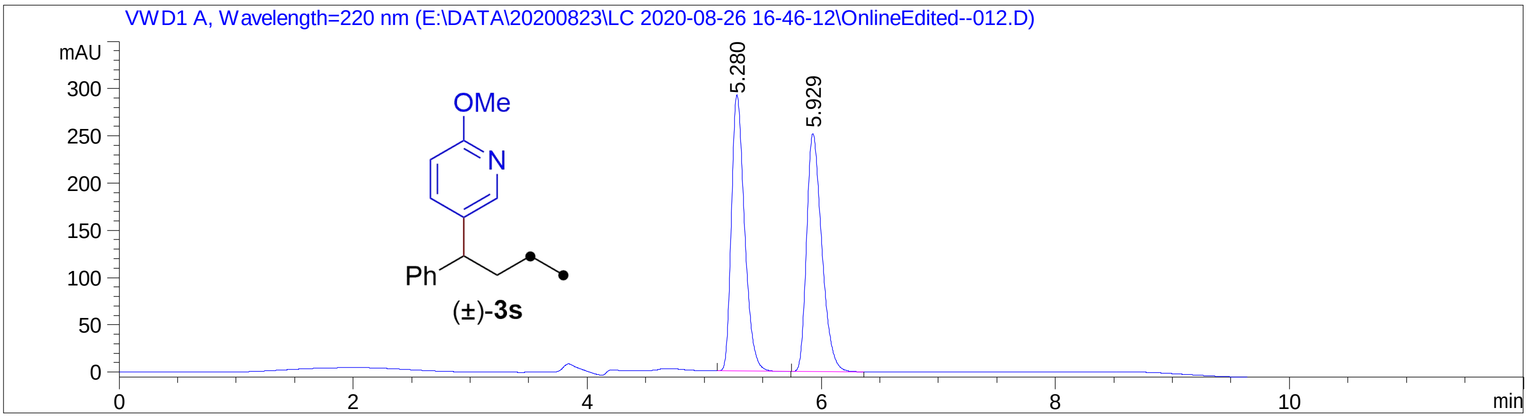

===

Area Percent Report

===

Sorted By : Signal
Multiplier : 1.0000
Dilution : 1.0000
Do not use Multiplier & Dilution Factor with ISTDs

Signal 1: VWD1 A, Wavelength=220 nm

Peak #	RetTime [min]	Type	Width [min]	Area [mAU*s]	Height [mAU]	Area %
1	5.280	BB	0.1202	2286.58057	292.43408	50.1367
2	5.929	BB	0.1383	2274.11108	252.02625	49.8633

Totals : 4560.69165 544.46033

===

*** End of Report ***

Data File E:\DATA\20200823\LC 2020-08-26 16-46-12\OnlineEdited--013.D
Sample Name: YH-17-104-EE

===

Acq. Operator : SYSTEM Seq. Line : 13
Acq. Instrument : HPLC1260 Location : P1-A6
Injection Date : 8/26/2020 9:29:52 PM Inj : 1
Inj Volume : 3.000 µl
Different Inj Volume from Sample Entry! Actual Inj Volume : 1.000 µl
Acq. Method : E:\DATA\20200823\LC 2020-08-26 16-46-12\5IPA_20_8_2.M
Last changed : 8/26/2020 9:44:35 PM by SYSTEM
(modified after loading)
Analysis Method : E:\DATA\20200823\LC 2020-08-26 16-46-12\5IPA_20_8_2.M (Sequence Method)
Last changed : 8/26/2020 9:46:42 PM by SYSTEM
(modified after loading)
Additional Info : Peak(s) manually integrated

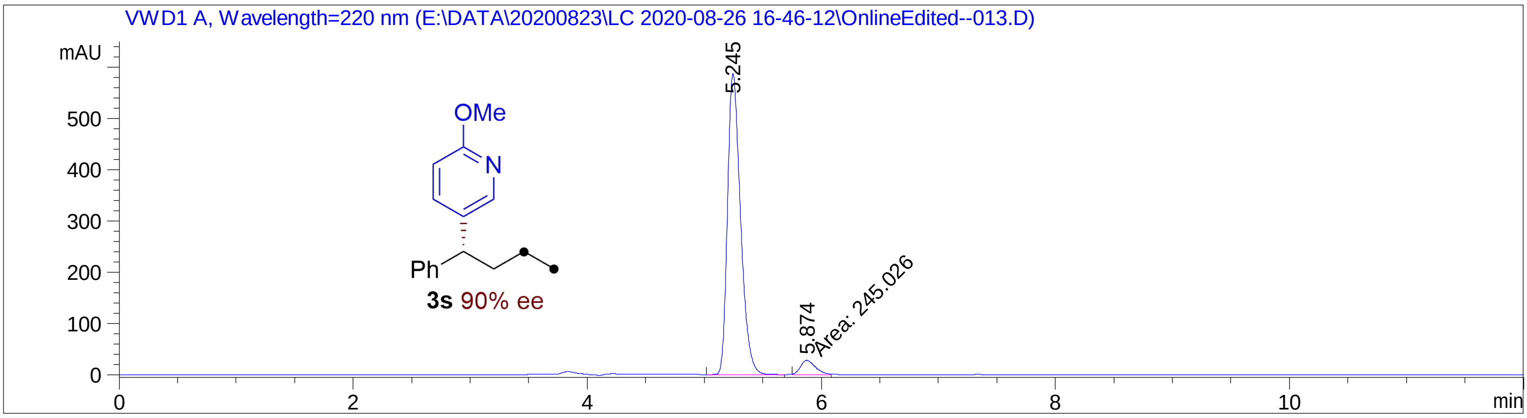

===

Area Percent Report

===

Sorted By : Signal
Multiplier : 1.0000
Dilution : 1.0000
Do not use Multiplier & Dilution Factor with ISTDs

Signal 1: VWD1 A, Wavelength=220 nm

Peak #	RetTime [min]	Type	Width [min]	Area [mAU*s]	Height [mAU]	Area %
1	5.245	BV	0.1190	4532.34863	587.33282	94.8711
2	5.874	FM	0.1473	245.02647	27.72322	5.1289

Totals : 4777.37511 615.05604

===

*** End of Report ***

Data File E:\DATA\20200831\LC 2020-09-02 09-50-30\OnlineEdited--012.D
Sample Name: YH-17-120-RAC

===

Acq. Operator   : SYSTEM   Seq. Line : 12
Acq. Instrument : HPLC1260   Location :   P1-A4
Injection Date : 9/2/2020 5:02:46 PM   Inj :   1
                                               Inj Volume : 3.000 µl
Different Inj Volume from Sample Entry! Actual Inj Volume : 8.000 µl
Acq. Method   : E:\DATA\20200831\LC 2020-09-02 09-50-30\2IPA_45_10_2.M
Last changed   : 9/2/2020 5:16:28 PM by SYSTEM
                  (modified after loading)
Analysis Method : E:\DATA\20200831\LC 2020-09-02 09-50-30\2IPA_45_10_2.M (Sequence Method)
Last changed   : 9/2/2020 5:51:52 PM by SYSTEM
                  (modified after loading)
Additional Info : Peak(s) manually integrated

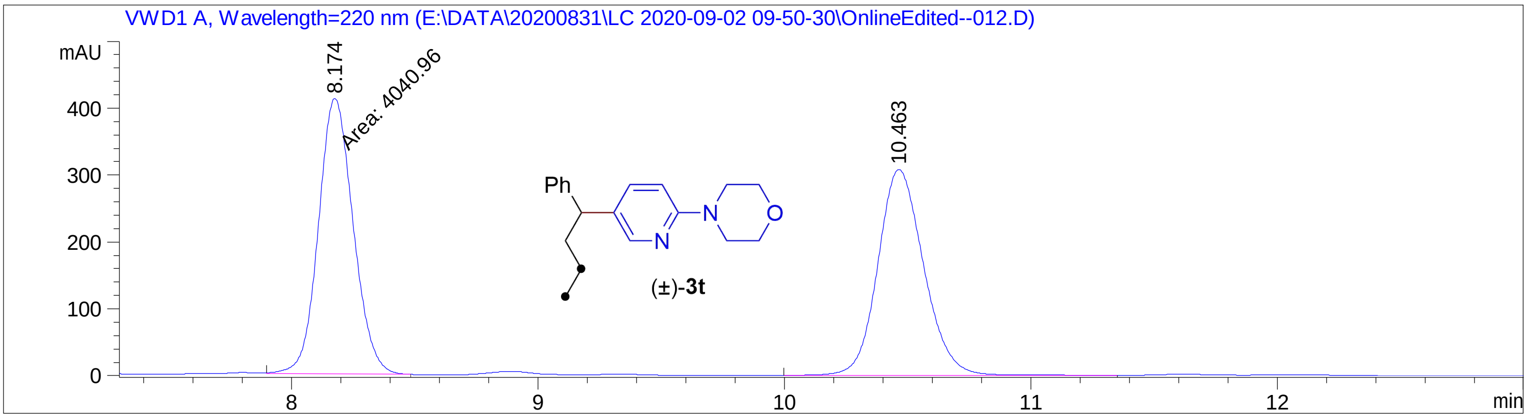

===

Area Percent Report

===

Sorted By   :   Signal
Multiplier   :   1.0000
Dilution   :   1.0000
Do not use Multiplier & Dilution Factor with ISTDs

Signal 1: VWD1 A, Wavelength=220 nm

Peak #	RetTime [min]	Type	Width [min]	Area [mAU*s]	Height [mAU]	Area %
1	8.174	MM	0.1635	4040.95679	411.87637	50.4548
2	10.463	BB	0.1996	3968.10010	307.35077	49.5452

Totals :   8009.05688 719.22714

===

*** End of Report ***

Data File E:\DATA\20200831\LC 2020-09-02 09-50-30\OnlineEdited--013.D
Sample Name: YH-17-120-EE

===

Acq. Operator : SYSTEM Seq. Line : 13
Acq. Instrument : HPLC1260 Location : P1-A5
Injection Date : 9/2/2020 5:23:33 PM Inj : 1
Inj Volume : 3.000 µl
Different Inj Volume from Sample Entry! Actual Inj Volume : 1.000 µl
Acq. Method : E:\DATA\20200831\LC 2020-09-02 09-50-30\2IPA_45_10_2.M
Last changed : 9/2/2020 5:16:28 PM by SYSTEM
Analysis Method : E:\DATA\20200831\LC 2020-09-02 09-50-30\2IPA_45_10_2.M (Sequence Method)
Last changed : 9/2/2020 5:53:00 PM by SYSTEM
(modified after loading)
Additional Info : Peak(s) manually integrated

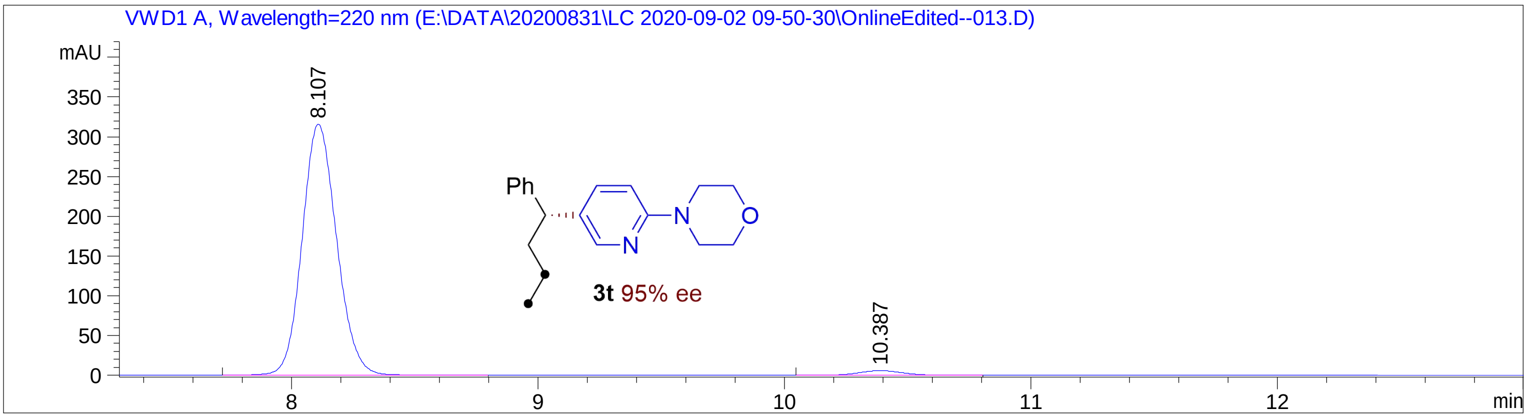

===
Area Percent Report
===

Sorted By : Signal
Multiplier : 1.0000
Dilution : 1.0000
Do not use Multiplier & Dilution Factor with ISTDs

Signal 1: VWD1 A, Wavelength=220 nm

Peak #	RetTime [min]	Type	Width [min]	Area [mAU*s]	Height [mAU]	Area %
1	8.107	BB	0.1505	3073.72876	315.87805	97.6446
2	10.387	BB	0.1928	74.14445	5.97577	2.3554

Totals : 3147.87321 321.85382

===
*** End of Report ***

Data File E:\DATA\20200905\LC 2020-09-10 20-53-53\1AC-0201.D
Sample Name: YH-17-157-RAC

===

Acq. Operator : SYSTEM Seq. Line : 2
Acq. Instrument : HPLC1260 Location : P1-A3
Injection Date : 9/10/2020 9:16:15 PM Inj : 1
Inj Volume : 3.000 µl
Different Inj Volume from Sample Entry! Actual Inj Volume : 0.200 µl
Acq. Method : E:\DATA\20200905\LC 2020-09-10 20-53-53\5IPA_20_8_3.M
Last changed : 9/10/2020 9:28:14 PM by SYSTEM
(modified after loading)
Analysis Method : E:\DATA\20200905\LC 2020-09-10 20-53-53\5IPA_20_8_3.M (Sequence Method)
Last changed : 9/10/2020 9:48:28 PM by SYSTEM
(modified after loading)
Additional Info : Peak(s) manually integrated

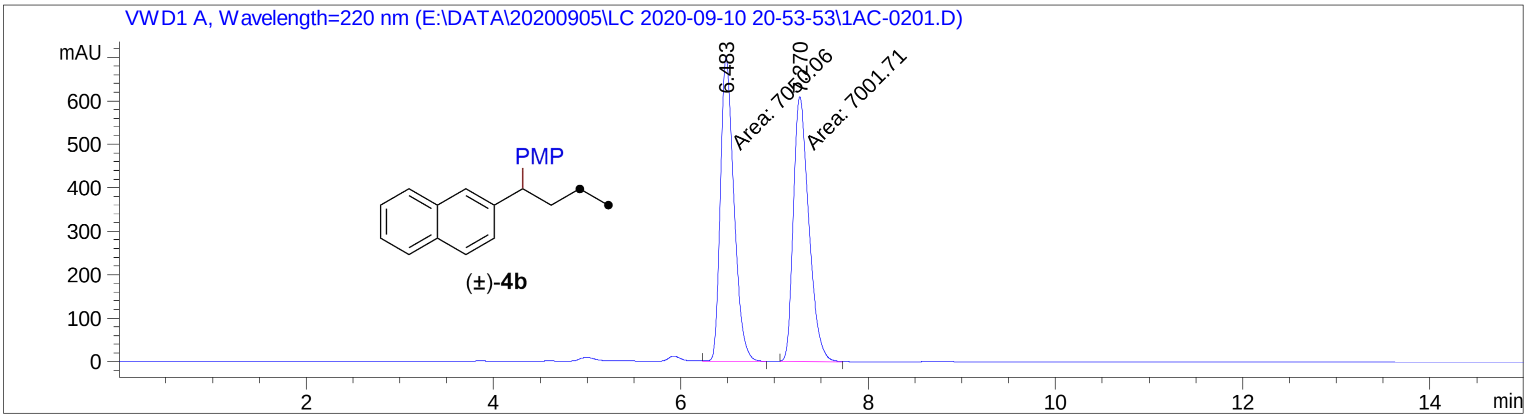

===

Area Percent Report

===

Sorted By : Signal
Multiplier : 1.0000
Dilution : 1.0000
Do not use Multiplier & Dilution Factor with ISTDs

Signal 1: VWD1 A, Wavelength=220 nm

Peak #	RetTime [min]	Type	Width [min]	Area [mAU*s]	Height [mAU]	Area %
1	6.483	FM	0.1674	7050.06348	701.99628	50.1720
2	7.270	MM	0.1916	7001.71338	609.16565	49.8280

Totals : 1.40518e4 1311.16193

===

*** End of Report ***

Data File E:\DATA\20200905\LC 2020-09-10 20-53-53\OnlineEdited--003.D
Sample Name: YH-17-157-EE

===

Acq. Operator : SYSTEM Seq. Line : 3
Acq. Instrument : HPLC1260 Location : P1-A4
Injection Date : 9/10/2020 9:31:58 PM Inj : 1
Inj Volume : 3.000 µl
Different Inj Volume from Sample Entry! Actual Inj Volume : 0.100 µl
Acq. Method : E:\DATA\20200905\LC 2020-09-10 20-53-53\5IPA_20_8_3.M
Last changed : 9/10/2020 9:28:14 PM by SYSTEM
Analysis Method : E:\DATA\20200905\LC 2020-09-10 20-53-53\5IPA_20_8_3.M (Sequence Method)
Last changed : 9/10/2020 9:48:28 PM by SYSTEM
(modified after loading)
Additional Info : Peak(s) manually integrated

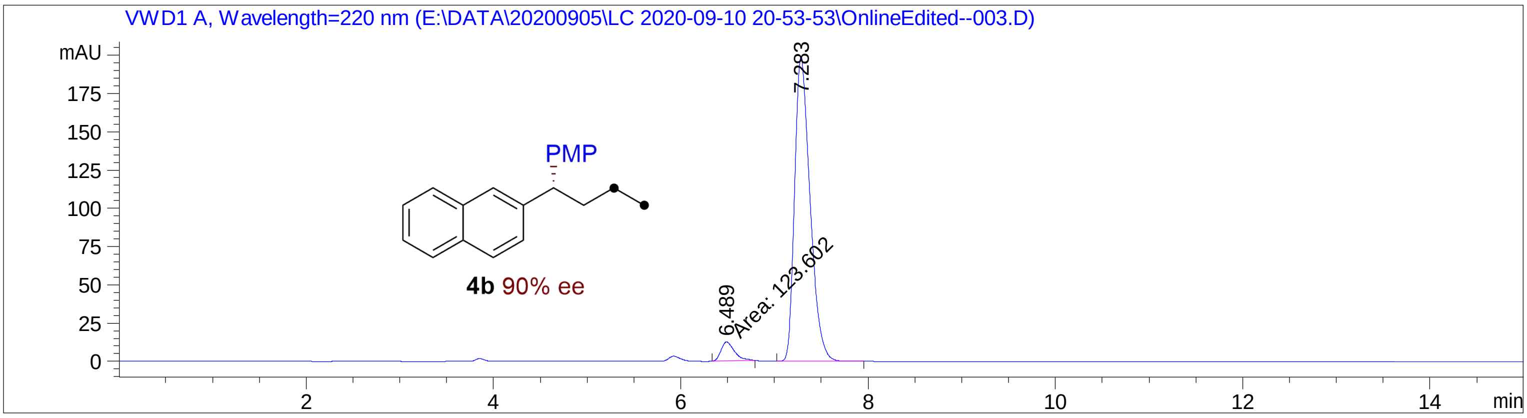

===

Area Percent Report

===

Sorted By : Signal
Multiplier : 1.0000
Dilution : 1.0000
Do not use Multiplier & Dilution Factor with ISTDs

Signal 1: VWD1 A, Wavelength=220 nm

Peak #	RetTime [min]	Type	Width [min]	Area [mAU*s]	Height [mAU]	Area %
1	6.489	MM	0.1657	123.60246	12.43593	5.1238
2	7.283	BB	0.1751	2288.71460	199.18835	94.8762

Totals : 2412.31705 211.62428

===

*** End of Report ***

Data File E:\DATA\20200905\LC 2020-09-06 15-18-12\OnlineEdited--066.D
Sample Name: YH-17-131-RAC

===

Acq. Operator : SYSTEM Seq. Line : 66
Acq. Instrument : HPLC1260 Location : P1-A5
Injection Date : 9/8/2020 2:17:15 AM Inj : 1
Inj Volume : 3.000 µl
Different Inj Volume from Sample Entry! Actual Inj Volume : 1.000 µl
Acq. Method : E:\DATA\20200905\LC 2020-09-06 15-18-12\1ETOH_80_5_3.M
Last changed : 9/7/2020 10:38:58 PM by SYSTEM
Analysis Method : E:\DATA\20200905\LC 2020-09-06 15-18-12\1ETOH_80_5_3.M (Sequence Method)
Last changed : 9/8/2020 11:29:32 AM by SYSTEM
(modified after loading)
Additional Info : Peak(s) manually integrated

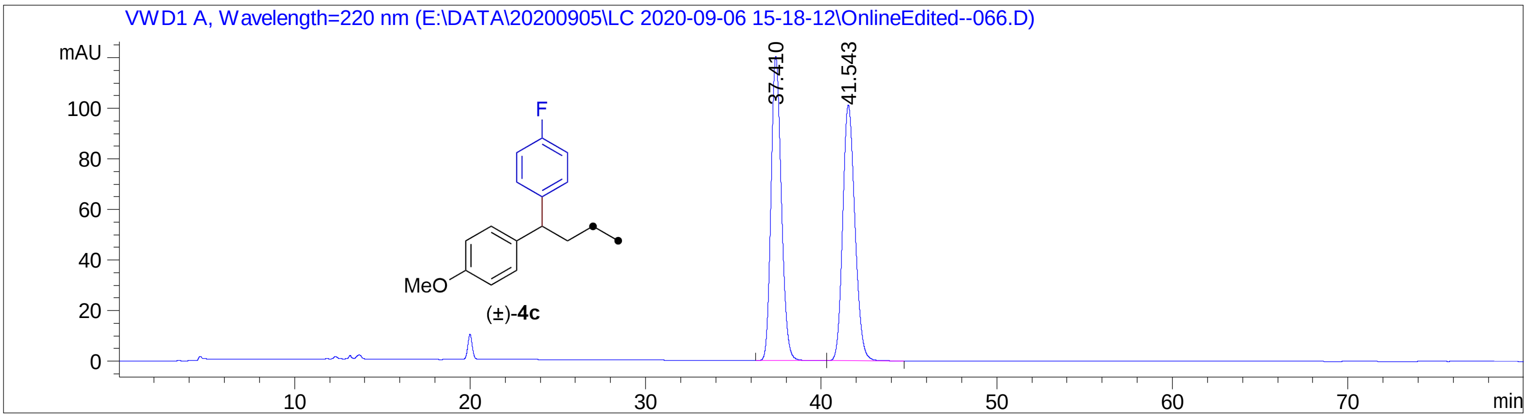

===

Area Percent Report

===

Sorted By : Signal
Multiplier : 1.0000
Dilution : 1.0000
Do not use Multiplier & Dilution Factor with ISTDs

Signal 1: VWD1 A, Wavelength=220 nm

Peak #	RetTime [min]	Type	Width [min]	Area [mAU*s]	Height [mAU]	Area %
1	37.410	BB	0.6312	4908.74658	120.27238	50.0023
2	41.543	BB	0.7496	4908.30127	101.24754	49.9977

Totals : 9817.04785 221.51991

===

*** End of Report ***

Data File E:\DATA\20200905\LC 2020-09-06 15-18-12\OnlineEdited--067.D
Sample Name: YH-17-131-EE

===

Acq. Operator : SYSTEM Seq. Line : 67
Acq. Instrument : HPLC1260 Location : P1-A6
Injection Date : 9/8/2020 3:38:00 AM Inj : 1
Inj Volume : 3.000 µl
Different Inj Volume from Sample Entry! Actual Inj Volume : 1.000 µl
Acq. Method : E:\DATA\20200905\LC 2020-09-06 15-18-12\1ETOH_80_5_3.M
Last changed : 9/7/2020 10:38:58 PM by SYSTEM
Analysis Method : E:\DATA\20200905\LC 2020-09-06 15-18-12\1ETOH_80_5_3.M (Sequence Method)
Last changed : 9/8/2020 11:29:32 AM by SYSTEM
(modified after loading)
Additional Info : Peak(s) manually integrated

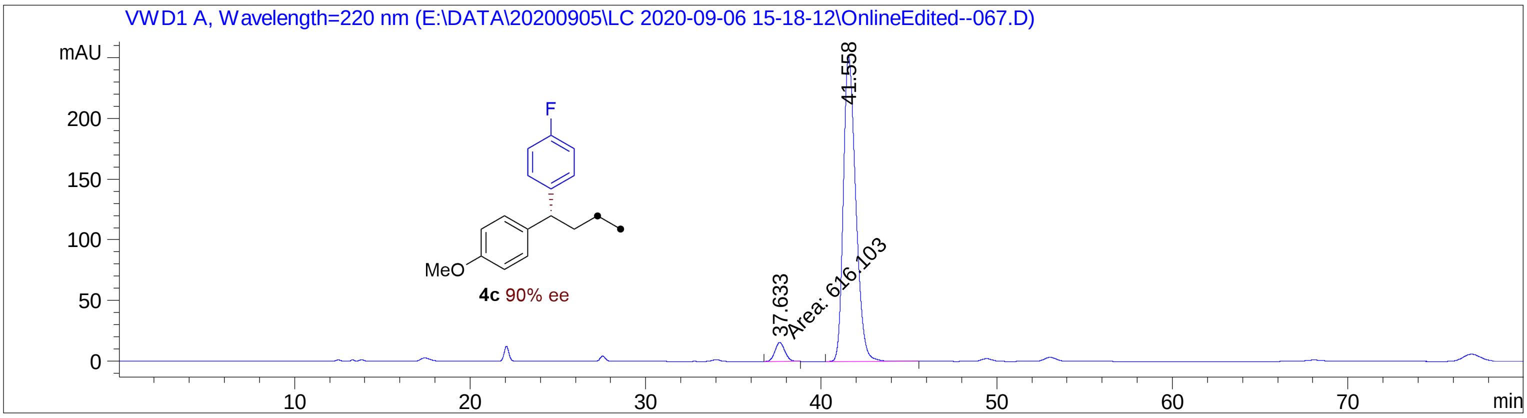

===
Area Percent Report
===

Sorted By : Signal
Multiplier : 1.0000
Dilution : 1.0000
Do not use Multiplier & Dilution Factor with ISTDs

Signal 1: VWD1 A, Wavelength=220 nm

Peak #	RetTime [min]	Type	Width [min]	Area [mAU*s]	Height [mAU]	Area %
1	37.633	MF	0.6604	616.10272	15.54854	4.7461
2	41.558	BB	0.7574	1.23651e4	251.14645	95.2539

Totals : 1.29812e4 266.69500

===
*** End of Report ***

Data File E:\DATA\20200905\LC 2020-09-13 18-37-17\OnlineEdited--067.D
Sample Name: YH-17-162-RAC

===

Acq. Operator : SYSTEM Seq. Line : 67
Acq. Instrument : HPLC1260 Location : P1-A4
Injection Date : 9/15/2020 12:15:47 AM Inj : 1
Inj Volume : 3.000 µl
Different Inj Volume from Sample Entry! Actual Inj Volume : 1.000 µl
Acq. Method : E:\DATA\20200905\LC 2020-09-13 18-37-17\5EtOH_50_5_1.M
Last changed : 9/14/2020 11:15:32 PM by SYSTEM
Analysis Method : E:\DATA\20200905\LC 2020-09-13 18-37-17\5EtOH_50_5_1.M (Sequence Method)
Last changed : 9/15/2020 2:54:21 PM by SYSTEM
(modified after loading)
Additional Info : Peak(s) manually integrated

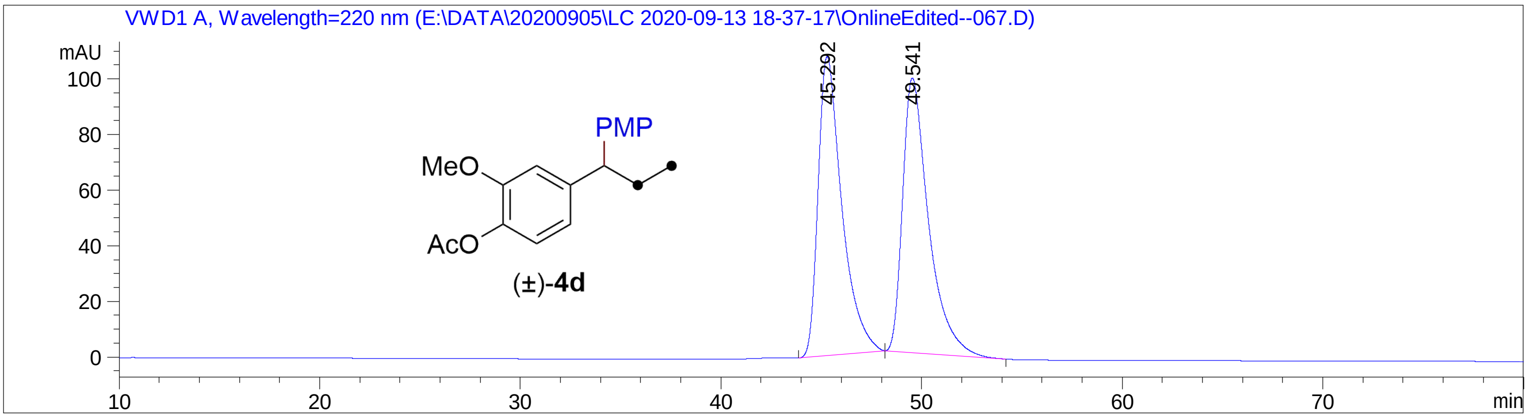

===

Area Percent Report

===

Sorted By : Signal
Multiplier : 1.0000
Dilution : 1.0000
Do not use Multiplier & Dilution Factor with ISTDs

Signal 1: VWD1 A, Wavelength=220 nm

Peak #	RetTime [min]	Type	Width [min]	Area [mAU*s]	Height [mAU]	Area %
1	45.292	BB	1.2246	8861.74219	107.51409	49.9567
2	49.541	BB	1.3363	8877.11035	98.79295	50.0433

Totals : 1.77389e4 206.30704

===

*** End of Report ***

Data File E:\DATA\20200905\LC 2020-09-13 18-37-17\OnlineEdited--066.D
Sample Name: YH-17-162-EE

===

Acq. Operator : SYSTEM   Seq. Line : 66
Acq. Instrument : HPLC1260   Location : P1-A5
Injection Date : 9/14/2020 10:55:01 PM   Inj : 1
Inj Volume : 3.000 µl
Different Inj Volume from Sample Entry! Actual Inj Volume : 1.000 µl
Acq. Method : E:\DATA\20200905\LC 2020-09-13 18-37-17\5EtOH_50_5_1.M
Last changed : 9/14/2020 11:15:32 PM by SYSTEM
(modified after loading)
Analysis Method : E:\DATA\20200905\LC 2020-09-13 18-37-17\5EtOH_50_5_1.M (Sequence Method)
Last changed : 9/15/2020 2:53:28 PM by SYSTEM
(modified after loading)
Additional Info : Peak(s) manually integrated

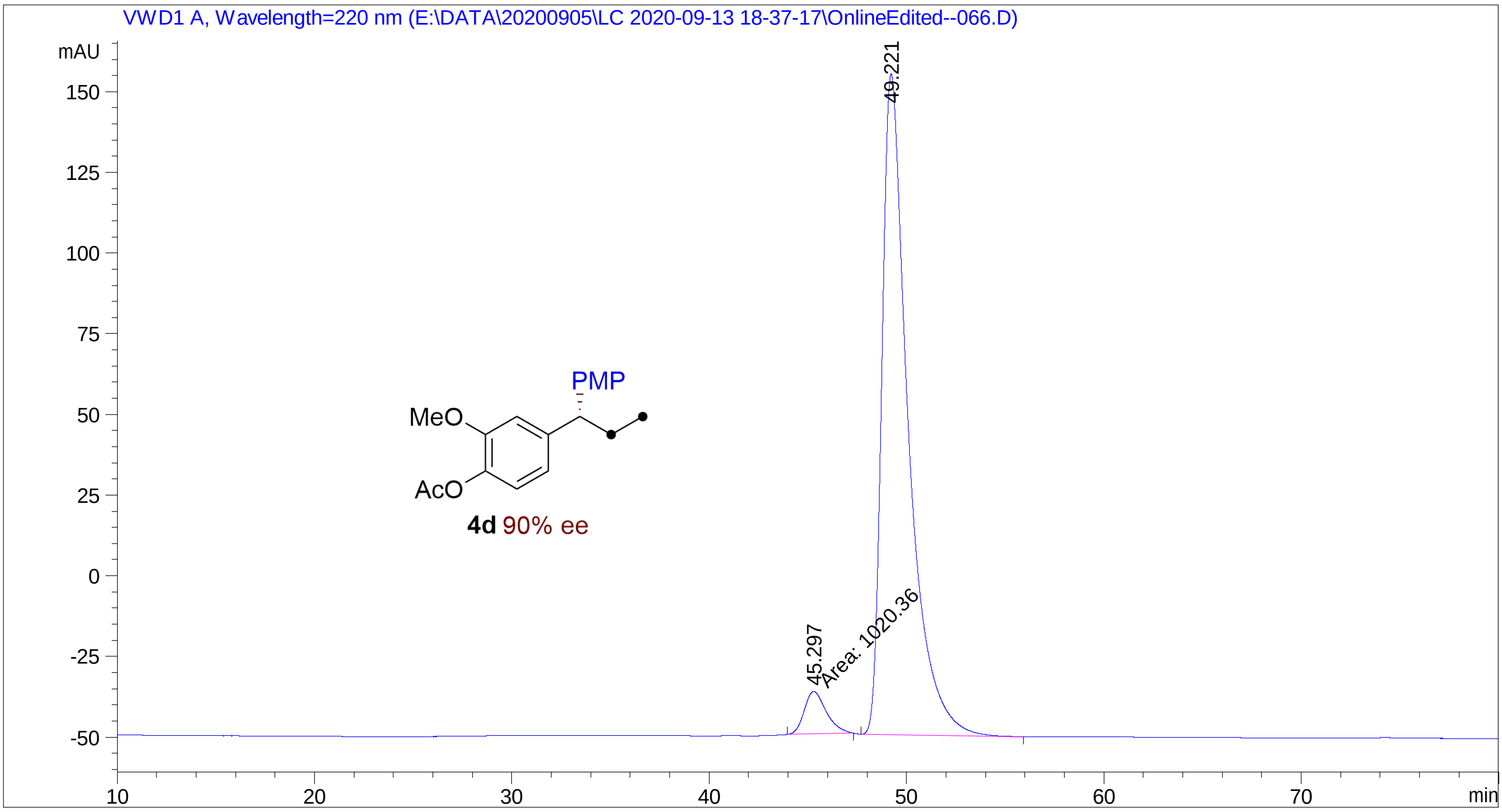

===
Area Percent Report
===

Sorted By : Signal
Multiplier : 1.0000
Dilution : 1.0000
Use Multiplier & Dilution Factor with ISTDs

Signal 1: VWD1 A, Wavelength=220 nm

Peak #	RetTime [min]	Type	Width [min]	Area [mAU*s]	Height [mAU]	Area %
1	45.297	MM	1.2879	1020.36469	13.20450	5.1082
2	49.221	BB	1.3588	1.89546e4	204.78865	94.8918

Totals : 1.99750e4 217.99315

Data File E:\DATA\20200905\LC 2020-09-13 18-37-17\OnlineEdited--060.D
Sample Name: YH-17-158-AD

===

Acq. Operator : SYSTEM Seq. Line : 60
Acq. Instrument : HPLC1260 Location : P1-B3
Injection Date : 9/14/2020 7:30:26 PM Inj : 1
Inj Volume : 3.000 µl
Different Inj Volume from Sample Entry! Actual Inj Volume : 1.000 µl
Acq. Method : E:\DATA\20200905\LC 2020-09-13 18-37-17\10ETOH_25_10_3.M
Last changed : 9/13/2020 8:50:01 PM by SYSTEM
Analysis Method : E:\DATA\20200905\LC 2020-09-13 18-37-17\10ETOH_25_10_3.M (Sequence Method)
Last changed : 9/14/2020 8:36:44 PM by SYSTEM
(modified after loading)
Additional Info : Peak(s) manually integrated

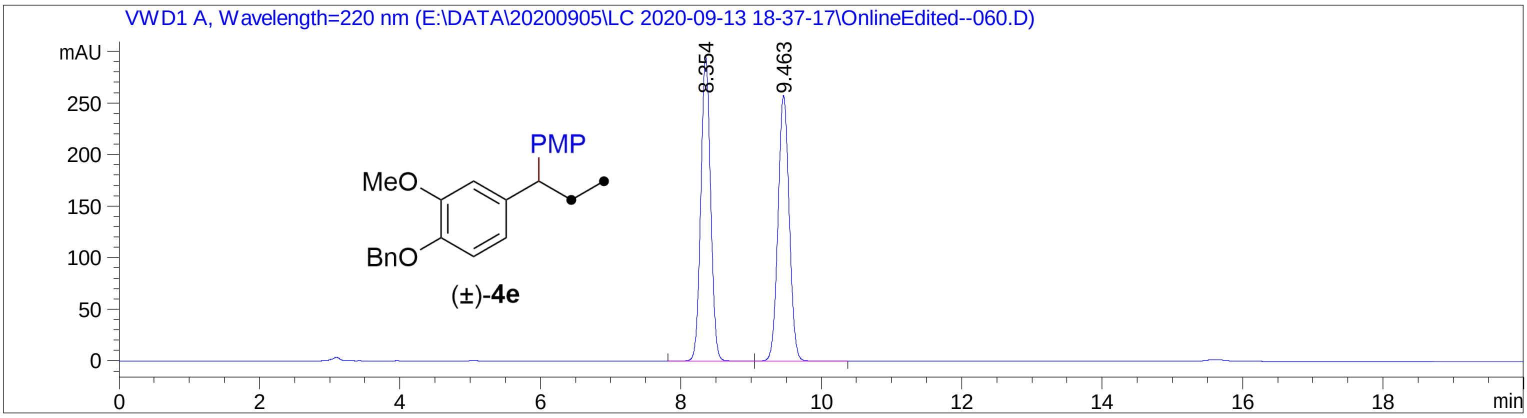

===

Area Percent Report

===

Sorted By : Signal
Multiplier : 1.0000
Dilution : 1.0000
Do not use Multiplier & Dilution Factor with ISTDs

Signal 1: VWD1 A, Wavelength=220 nm

Peak #	RetTime [min]	Type	Width [min]	Area [mAU*s]	Height [mAU]	Area %
1	8.354	BB	0.1500	2859.24878	295.21860	50.0154
2	9.463	BB	0.1724	2857.48560	257.59555	49.9846

Totals : 5716.73438 552.81415

===

*** End of Report ***

Data File E:\DATA\20200905\LC 2020-09-13 18-37-17\OnlineEdited--063.D
Sample Name: YH-17-158-EE

===

Acq. Operator : SYSTEM Seq. Line : 63
Acq. Instrument : HPLC1260 Location : P1-A2
Injection Date : 9/14/2020 8:47:44 PM Inj : 1
Inj Volume : 3.000 µl
Different Inj Volume from Sample Entry! Actual Inj Volume : 1.000 µl
Acq. Method : E:\DATA\20200905\LC 2020-09-13 18-37-17\10ETOH_25_10_3.M
Last changed : 9/13/2020 8:50:01 PM by SYSTEM
Analysis Method : E:\DATA\20200905\LC 2020-09-13 18-37-17\10ETOH_25_10_3.M (Sequence Method)
Last changed : 9/14/2020 9:19:46 PM by SYSTEM
(modified after loading)
Additional Info : Peak(s) manually integrated

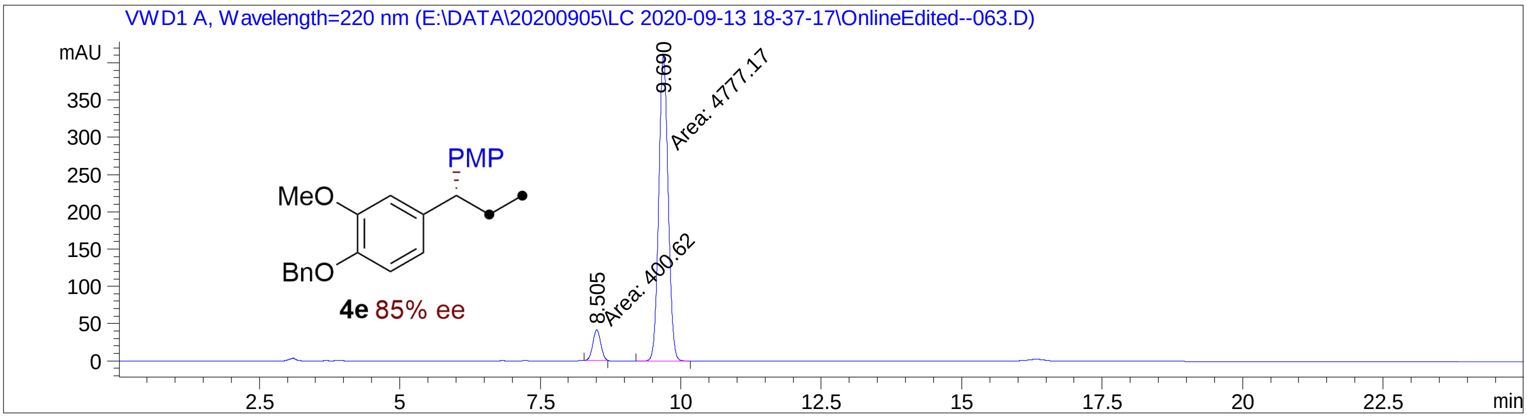

===
Area Percent Report
===

Sorted By : Signal
Multiplier : 1.0000
Dilution : 1.0000
Do not use Multiplier & Dilution Factor with ISTDs

Signal 1: VWD1 A, Wavelength=220 nm

Peak #	RetTime [min]	Type	Width [min]	Area [mAU*s]	Height [mAU]	Area %
1	8.505	MM	0.1624	400.61996	41.11270	7.7373
2	9.690	MF	0.1947	4777.16895	408.85339	92.2627

Totals : 5177.78891 449.96609

===
*** End of Report ***

Data File E:\DATA\20200905\LC 2020-09-13 18-37-17\OnlineEdited--069.D
Sample Name: YH-17-163-RAC

===
Acq. Operator : SYSTEM Seq. Line : 69
Acq. Instrument : HPLC1260 Location : P1-A6
Injection Date : 9/15/2020 2:02:19 AM Inj : 1
Inj Volume : 3.000 µl
Different Inj Volume from Sample Entry! Actual Inj Volume : 1.000 µl
Acq. Method : E:\DATA\20200905\LC 2020-09-13 18-37-17\10EtOH_25_8_1.M
Last changed : 9/14/2020 8:28:42 PM by SYSTEM
Analysis Method : E:\DATA\20200905\LC 2020-09-13 18-37-17\10EtOH_25_8_1.M (Sequence Method)
Last changed : 9/15/2020 2:57:52 PM by SYSTEM
(modified after loading)
Additional Info : Peak(s) manually integrated

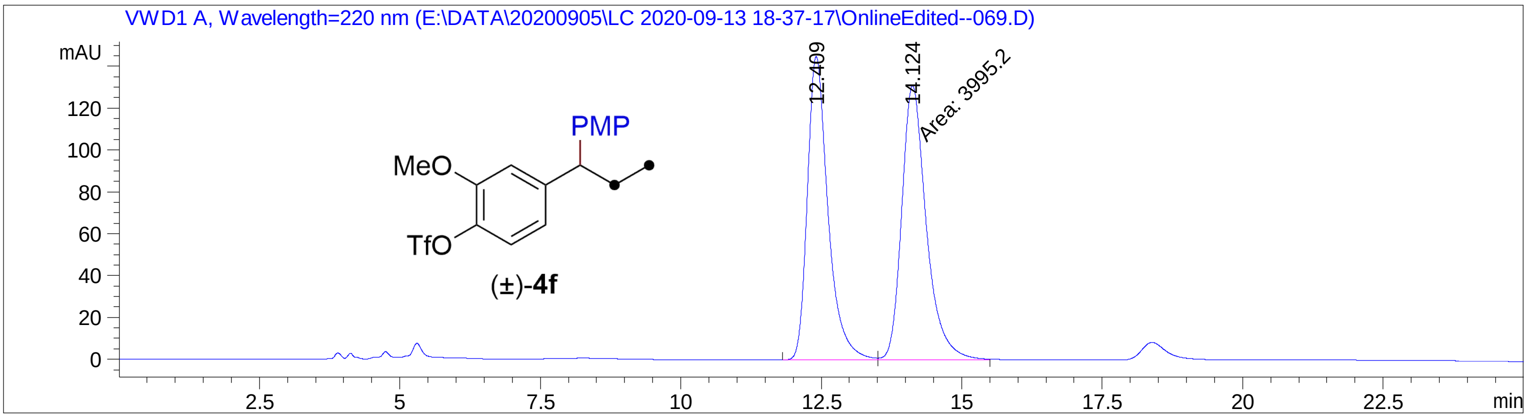

===
Area Percent Report
===

Sorted By : Signal
Multiplier : 1.0000
Dilution : 1.0000
Do not use Multiplier & Dilution Factor with ISTDs

Signal 1: VWD1 A, Wavelength=220 nm

Peak #	RetTime [min]	Type	Width [min]	Area [mAU*s]	Height [mAU]	Area %
1	12.409	BV	0.3871	3712.69434	144.81770	48.1674
2	14.124	MF	0.5084	3995.20142	130.98309	51.8326
Totals :				7707.89575	275.80080	

===
*** End of Report ***

===

Acq. Operator : SYSTEM Seq. Line : 70
Acq. Instrument : HPLC1260 Location : P1-A7
Injection Date : 9/15/2020 2:28:04 AM Inj : 1
Inj Volume : 3.000 µl
Different Inj Volume from Sample Entry! Actual Inj Volume : 1.000 µl
Acq. Method : E:\DATA\20200905\LC 2020-09-13 18-37-17\10EtOH_25_8_1.M
Last changed : 9/14/2020 8:28:42 PM by SYSTEM
Analysis Method : E:\DATA\20200905\LC 2020-09-13 18-37-17\10EtOH_25_8_1.M (Sequence Method)
Last changed : 9/15/2020 2:57:52 PM by SYSTEM
(modified after loading)
Additional Info : Peak(s) manually integrated

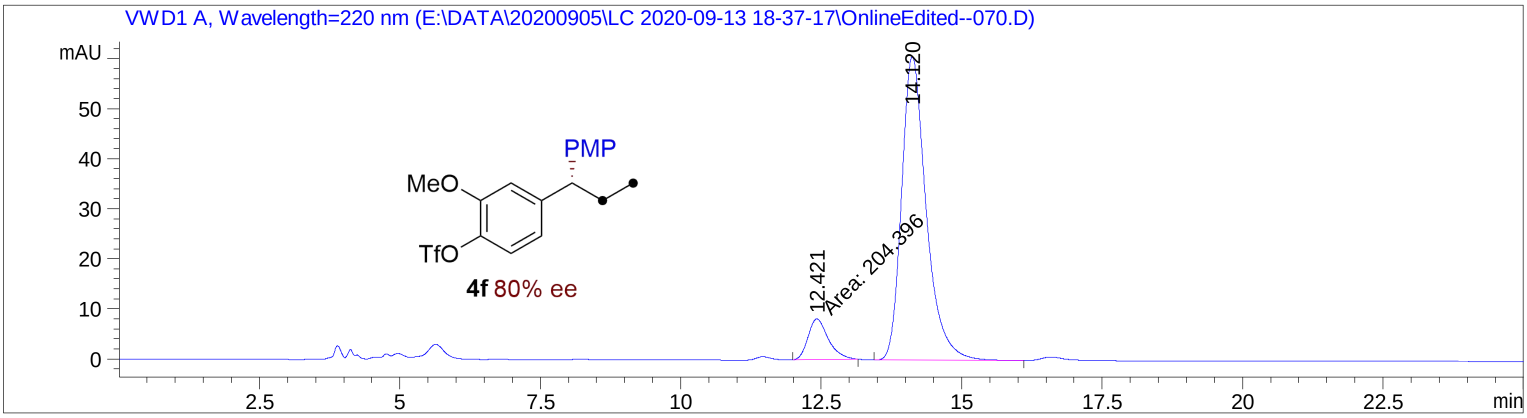

===

Area Percent Report

===

Sorted By : Signal
Multiplier : 1.0000
Dilution : 1.0000
Do not use Multiplier & Dilution Factor with ISTDs

Signal 1: VWD1 A, Wavelength=220 nm

Peak #	RetTime [min]	Type	Width [min]	Area [mAU*s]	Height [mAU]	Area %
1	12.421	MM	0.4185	204.39621	8.14016	9.9366
2	14.120	BB	0.4624	1852.61621	60.63935	90.0634

Totals : 2057.01242 68.77951

===

*** End of Report ***

Data File E:\DATA\20200905\LC 2020-09-06 15-18-12\OnlineEdited--123.D
Sample Name: YH-17-139-RAC

===

Acq. Operator : SYSTEM   Seq. Line : 123
Acq. Instrument : HPLC1260   Location : P1-A1
Injection Date : 9/9/2020 3:00:51 AM   Inj : 1
Inj Volume : 3.000 µl
Different Inj Volume from Sample Entry! Actual Inj Volume : 1.000 µl
Acq. Method : E:\DATA\20200905\LC 2020-09-06 15-18-12\1ETOH_80_5_3.M
Last changed : 9/7/2020 10:38:58 PM by SYSTEM
Analysis Method : E:\DATA\20200905\LC 2020-09-06 15-18-12\1ETOH_80_5_3.M (Sequence Method)
Last changed : 9/9/2020 9:20:26 AM by SYSTEM
(modified after loading)
Additional Info : Peak(s) manually integrated

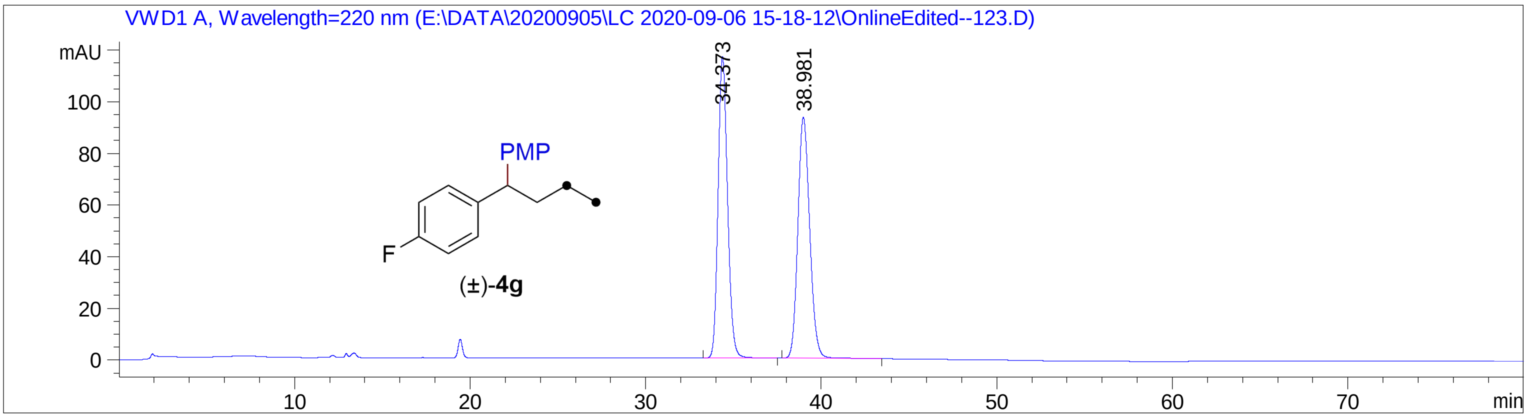

===

Area Percent Report

===

Sorted By : Signal
Multiplier : 1.0000
Dilution : 1.0000
Do not use Multiplier & Dilution Factor with ISTDs

Signal 1: VWD1 A, Wavelength=220 nm

Peak #	RetTime [min]	Type	Width [min]	Area [mAU*s]	Height [mAU]	Area %
1	34.373	BB	0.5767	4356.69092	116.66521	49.9836
2	38.981	BB	0.7241	4359.55127	93.31073	50.0164

Totals : 8716.24219 209.97594

===

*** End of Report ***

===

Acq. Operator : SYSTEM Seq. Line : 124
Acq. Instrument : HPLC1260 Location : P1-A2
Injection Date : 9/9/2020 4:21:36 AM Inj : 1
Inj Volume : 3.000 µl
Different Inj Volume from Sample Entry! Actual Inj Volume : 1.000 µl
Acq. Method : E:\DATA\20200905\LC 2020-09-06 15-18-12\1ETOH_80_5_3.M
Last changed : 9/7/2020 10:38:58 PM by SYSTEM
Analysis Method : E:\DATA\20200905\LC 2020-09-06 15-18-12\1ETOH_80_5_3.M (Sequence Method)
Last changed : 9/9/2020 9:20:26 AM by SYSTEM
(modified after loading)
Additional Info : Peak(s) manually integrated

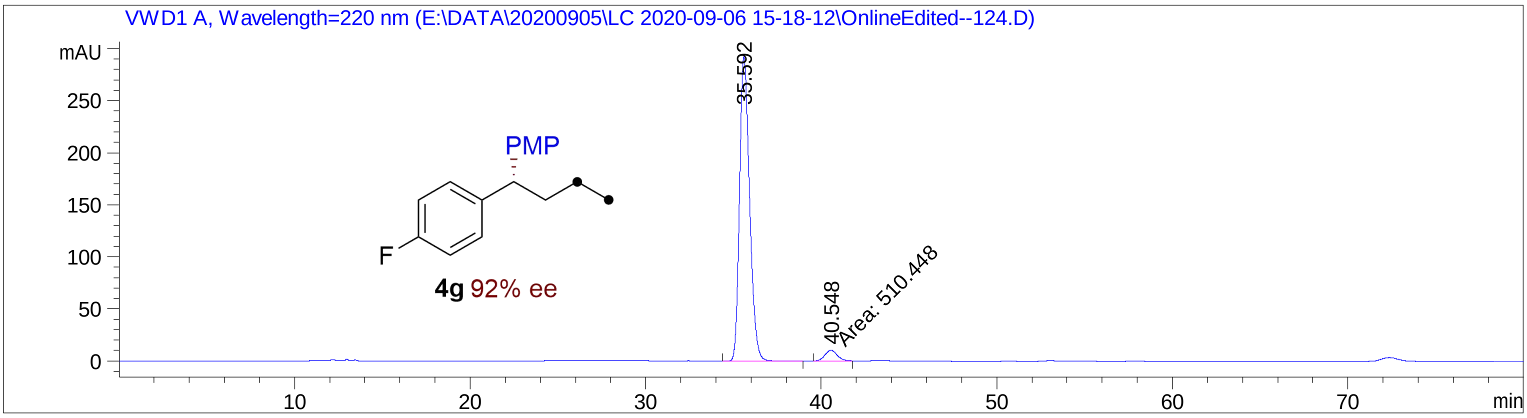

===

Area Percent Report

===

Sorted By : Signal
Multiplier : 1.0000
Dilution : 1.0000
Do not use Multiplier & Dilution Factor with ISTDs

Signal 1: VWD1 A, Wavelength=220 nm

Peak #	RetTime [min]	Type	Width [min]	Area [mAU*s]	Height [mAU]	Area %
1	35.592	BB	0.6119	1.16652e4	292.72021	95.8076
2	40.548	MF	0.8214	510.44781	10.35777	4.1924

Totals : 1.21757e4 303.07798

===

*** End of Report ***

Data File E:\DATA\20200905\LC 2020-09-05 14-35-09\OnlineEdited--004.D
Sample Name: YH-17-130-RAC

===

Acq. Operator : SYSTEM Seq. Line : 4
Acq. Instrument : HPLC1260 Location : P1-A1
Injection Date : 9/5/2020 3:34:37 PM Inj : 1
Inj Volume : 3.000 µl
Different Inj Volume from Sample Entry! Actual Inj Volume : 0.500 µl
Acq. Method : E:\DATA\20200905\LC 2020-09-05 14-35-09\1IPA_20_5_2.M
Last changed : 9/5/2020 3:46:58 PM by SYSTEM
(modified after loading)
Analysis Method : E:\DATA\20200905\LC 2020-09-05 14-35-09\1IPA_20_5_2.M (Sequence Method)
Last changed : 9/5/2020 4:25:03 PM by SYSTEM
(modified after loading)
Additional Info : Peak(s) manually integrated

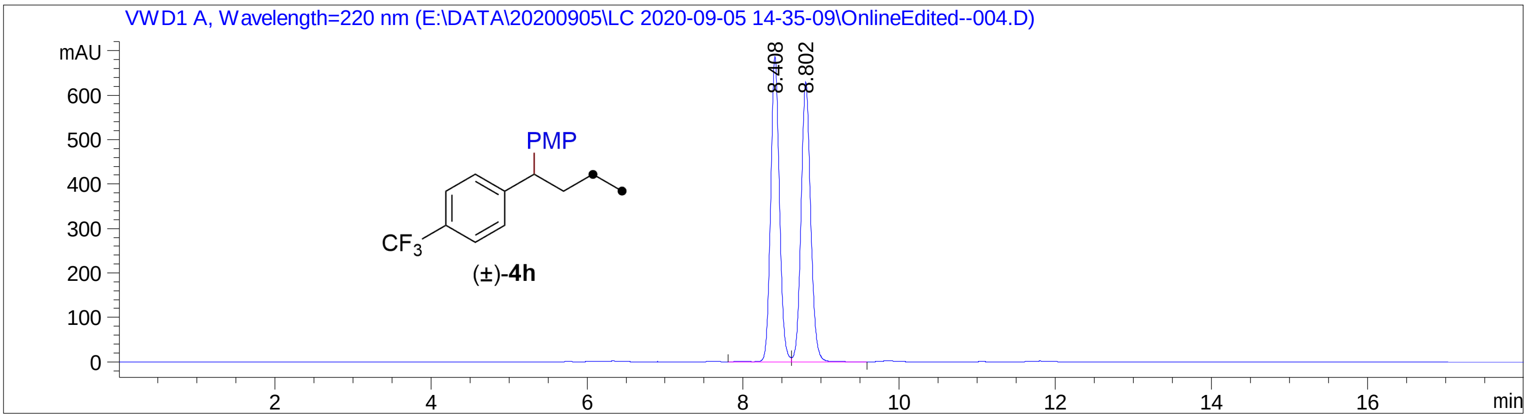

===

Area Percent Report

===

Sorted By : Signal
Multiplier : 1.0000
Dilution : 1.0000
Do not use Multiplier & Dilution Factor with ISTDs

Signal 1: VWD1 A, Wavelength=220 nm

Peak #	RetTime [min]	Type	Width [min]	Area [mAU*s]	Height [mAU]	Area %
1	8.408	VV R	0.1196	5336.57178	686.96912	49.8828
2	8.802	VB	0.1314	5361.65088	629.10931	50.1172

Totals : 1.06982e4 1316.07843

===

*** End of Report ***

===

Acq. Operator : SYSTEM Seq. Line : 6
Acq. Instrument : HPLC1260 Location : P1-A3
Injection Date : 9/5/2020 4:12:07 PM Inj : 1
Inj Volume : 3.000 µl
Different Inj Volume from Sample Entry! Actual Inj Volume : 0.600 µl
Acq. Method : E:\DATA\20200905\LC 2020-09-05 14-35-09\1IPA_20_5_2.M
Last changed : 9/5/2020 3:46:58 PM by SYSTEM
Analysis Method : E:\DATA\20200905\LC 2020-09-05 14-35-09\1IPA_20_5_2.M (Sequence Method)
Last changed : 9/5/2020 3:52:45 PM by SYSTEM
Additional Info : Peak(s) manually integrated

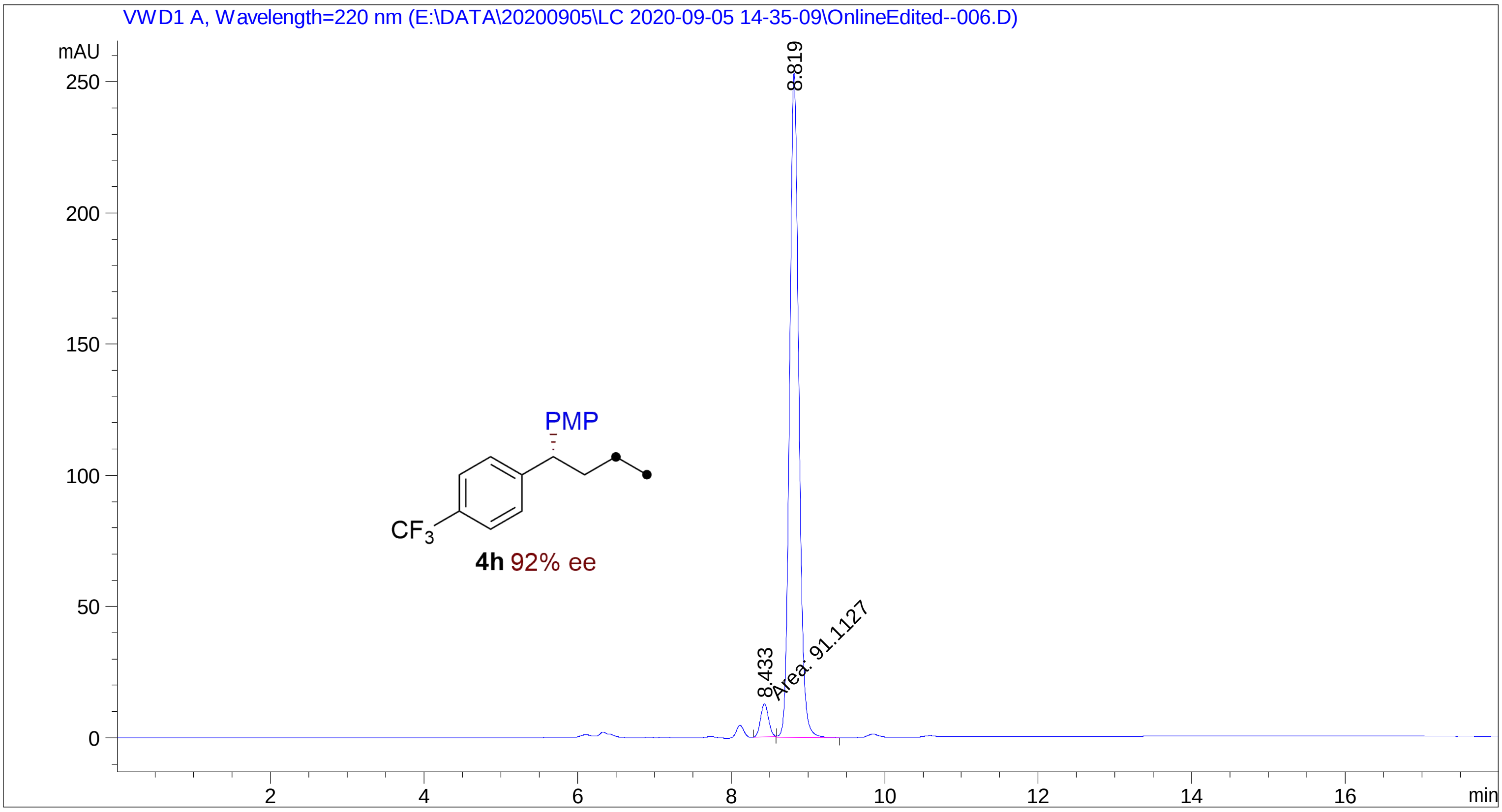

===
Area Percent Report
===

Sorted By : Signal
Multiplier : 1.0000
Dilution : 1.0000
Use Multiplier & Dilution Factor with ISTDs

Signal 1: VWD1 A, Wavelength=220 nm

Peak #	RetTime [min]	Type	Width [min]	Area [mAU*s]	Height [mAU]	Area %
1	8.433	MM	0.1195	91.11269	12.71069	4.1149
2	8.819	VB	0.1299	2123.07544	253.10728	95.8851

Totals : 2214.18813 265.81798

===

Data File E:\DATA\20200905\LC 2020-09-09 14-34-17\OnlineEdited--082.D
Sample Name: YH-17-155-RAC

===

Acq. Operator : SYSTEM Seq. Line : 82
Acq. Instrument : HPLC1260 Location : P1-A1
Injection Date : 9/10/2020 7:24:48 PM Inj : 4
Inj Volume : 3.000 µl
Different Inj Volume from Sample Entry! Actual Inj Volume : 1.000 µl
Acq. Method : E:\DATA\20200905\LC 2020-09-09 14-34-17\1IPA_40_5_4.M
Last changed : 9/10/2020 7:44:03 PM by SYSTEM
(modified after loading)
Analysis Method : E:\DATA\20200905\LC 2020-09-09 14-34-17\1IPA_40_5_4.M (Sequence Method)
Last changed : 9/10/2020 8:00:32 PM by SYSTEM
(modified after loading)
Additional Info : Peak(s) manually integrated

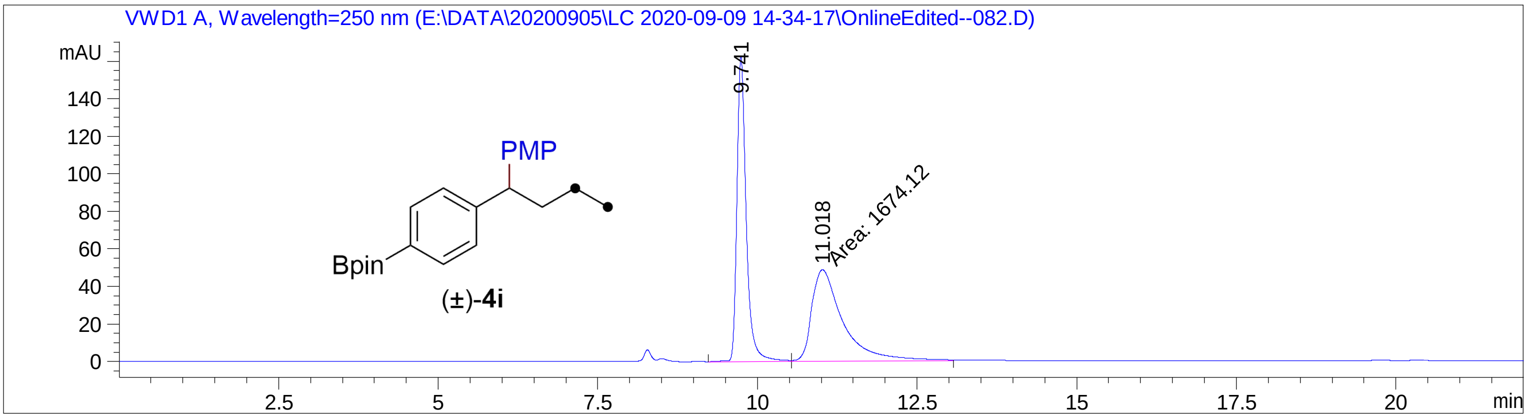

===

Area Percent Report

===

Sorted By : Signal
Multiplier : 1.0000
Dilution : 1.0000
Do not use Multiplier & Dilution Factor with ISTDs

Signal 1: VWD1 A, Wavelength=250 nm

Peak #	RetTime [min]	Type	Width [min]	Area [mAU*s]	Height [mAU]	Area %
1	9.741	VV R	0.1516	1637.99194	162.52676	49.4546
2	11.018	MF	0.5721	1674.11987	48.77103	50.5454

Totals : 3312.11182 211.29780

===

*** End of Report ***

Data File E:\DATA\20200905\LC 2020-09-09 14-34-17\OnlineEdited--083.D
Sample Name: YH-17-155-EE

===

Acq. Operator : SYSTEM Seq. Line : 83
Acq. Instrument : HPLC1260 Location : P1-A2
Injection Date : 9/10/2020 7:47:35 PM Inj : 1
Inj Volume : 3.000 µl
Acq. Method : E:\DATA\20200905\LC 2020-09-09 14-34-17\1IPA_40_5_4.M
Last changed : 9/10/2020 7:44:03 PM by SYSTEM
Analysis Method : E:\DATA\20200905\LC 2020-09-09 14-34-17\1IPA_40_5_4.M (Sequence Method)
Last changed : 9/10/2020 8:26:35 PM by SYSTEM
(modified after loading)
Additional Info : Peak(s) manually integrated

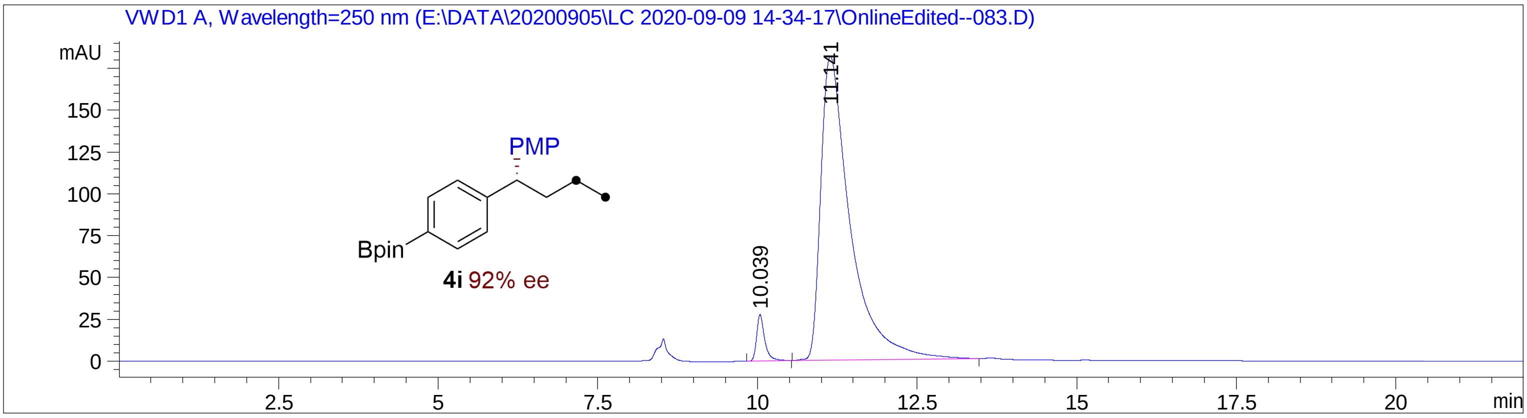

===

Area Percent Report

===

Sorted By : Signal
Multiplier : 1.0000
Dilution : 1.0000
Do not use Multiplier & Dilution Factor with ISTDs

Signal 1: VWD1 A, Wavelength=250 nm

Peak #	RetTime [min]	Type	Width [min]	Area [mAU*s]	Height [mAU]	Area %
1	10.039	BB	0.1305	243.71513	27.74428	4.0130
2	11.141	BB	0.4706	5829.43262	181.53410	95.9870

Totals : 6073.14775 209.27839

===

*** End of Report ***

===

Acq. Operator : SYSTEM   Seq. Line : 91
Acq. Instrument : HPLC1260   Location : P1-A3
Injection Date : 9/8/2020 2:19:49 PM   Inj : 1
Inj Volume : 3.000 µl
Different Inj Volume from Sample Entry! Actual Inj Volume : 0.500 µl
Acq. Method : E:\DATA\20200905\LC 2020-09-06 15-18-12\5IPA_25_8_2.M
Last changed : 9/8/2020 2:03:49 PM by SYSTEM
Analysis Method : E:\DATA\20200905\LC 2020-09-06 15-18-12\5IPA_25_8_2.M (Sequence Method)
Last changed : 9/8/2020 3:04:46 PM by SYSTEM
(modified after loading)
Additional Info : Peak(s) manually integrated

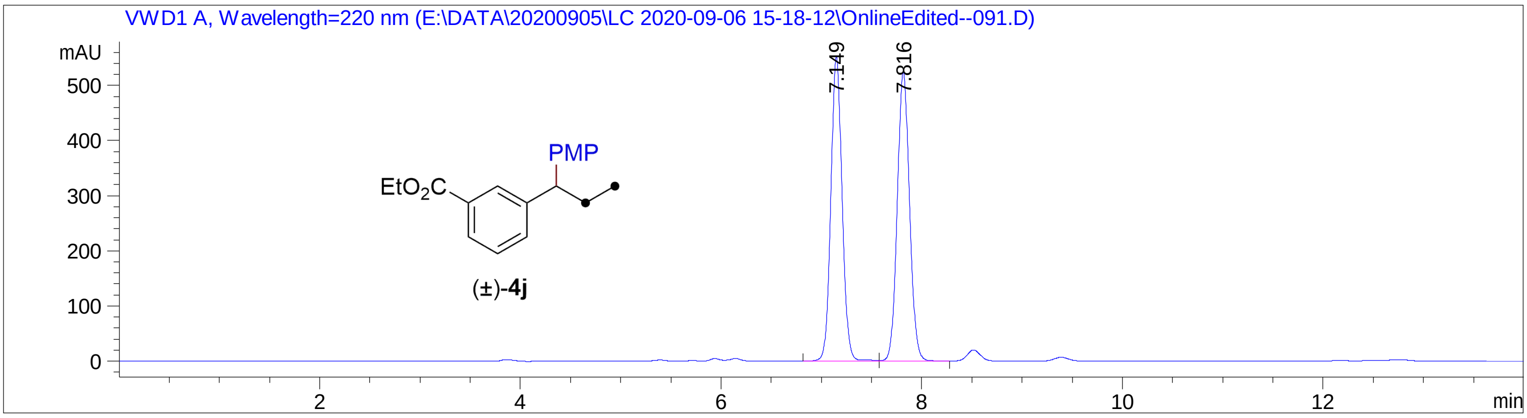

===

Area Percent Report

===

Sorted By : Signal
Multiplier : 1.0000
Dilution : 1.0000
Do not use Multiplier & Dilution Factor with ISTDs

Signal 1: VWD1 A, Wavelength=220 nm

Peak #	RetTime [min]	Type	Width [min]	Area [mAU*s]	Height [mAU]	Area %
1	7.149	BV R	0.1204	4298.37500	552.23834	49.1222
2	7.816	VB	0.1310	4451.99170	524.52710	50.8778

Totals : 8750.36670 1076.76544

===

*** End of Report ***

===

Acq. Operator : SYSTEM Seq. Line : 92
Acq. Instrument : HPLC1260 Location : P1-A4
Injection Date : 9/8/2020 2:34:31 PM Inj : 1
Inj Volume : 3.000 µl
Different Inj Volume from Sample Entry! Actual Inj Volume : 0.500 µl
Acq. Method : E:\DATA\20200905\LC 2020-09-06 15-18-12\5IPA_25_8_2.M
Last changed : 9/8/2020 2:03:49 PM by SYSTEM
Analysis Method : E:\DATA\20200905\LC 2020-09-06 15-18-12\5IPA_25_8_2.M (Sequence Method)
Last changed : 9/8/2020 3:04:46 PM by SYSTEM
(modified after loading)
Additional Info : Peak(s) manually integrated

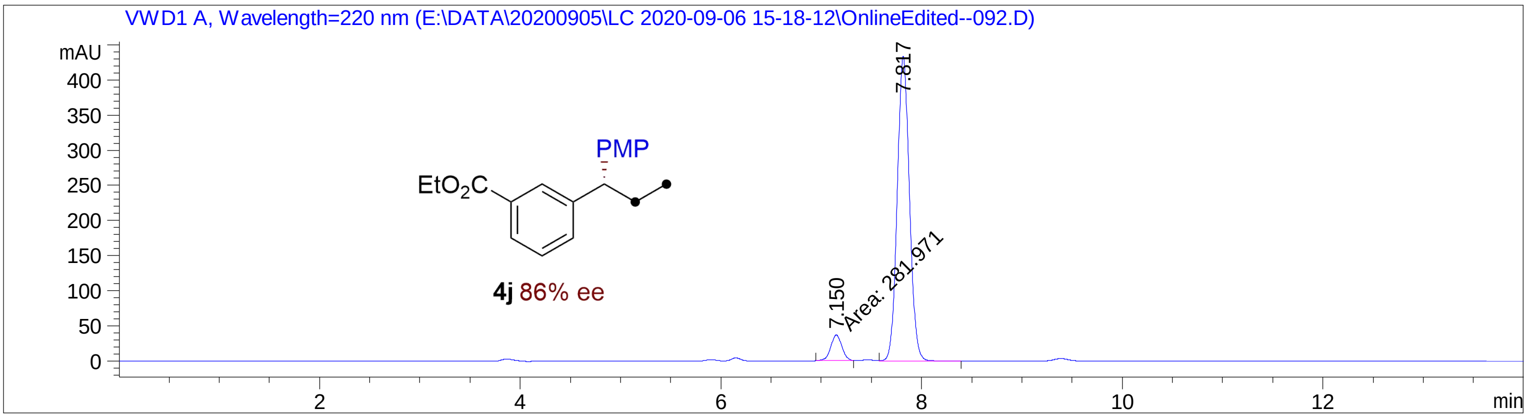

===

Area Percent Report

===

Sorted By : Signal
Multiplier : 1.0000
Dilution : 1.0000
Do not use Multiplier & Dilution Factor with ISTDs

Signal 1: VWD1 A, Wavelength=220 nm

Peak #	RetTime [min]	Type	Width [min]	Area [mAU*s]	Height [mAU]	Area %
1	7.150	MM	0.1281	281.97110	36.67854	7.1251
2	7.817	VB	0.1310	3675.43799	433.33679	92.8749

Totals : 3957.40909 470.01534

===

*** End of Report ***

Data File E:\DATA\20200905\LC 2020-09-06 15-18-12\OnlineEdited--093.D
Sample Name: YH-17-134-RAC

===
Acq. Operator : SYSTEM   Seq. Line : 93
Acq. Instrument : HPLC1260   Location : P1-A5
Injection Date : 9/8/2020 2:49:14 PM   Inj : 1
Inj Volume : 3.000 µl
Different Inj Volume from Sample Entry! Actual Inj Volume : 0.500 µl
Acq. Method : E:\DATA\20200905\LC 2020-09-06 15-18-12\5IPA_25_8_2.M
Last changed : 9/8/2020 2:03:49 PM by SYSTEM
Analysis Method : E:\DATA\20200905\LC 2020-09-06 15-18-12\5IPA_25_8_2.M (Sequence Method)
Last changed : 9/8/2020 3:05:48 PM by SYSTEM
(modified after loading)
Additional Info : Peak(s) manually integrated

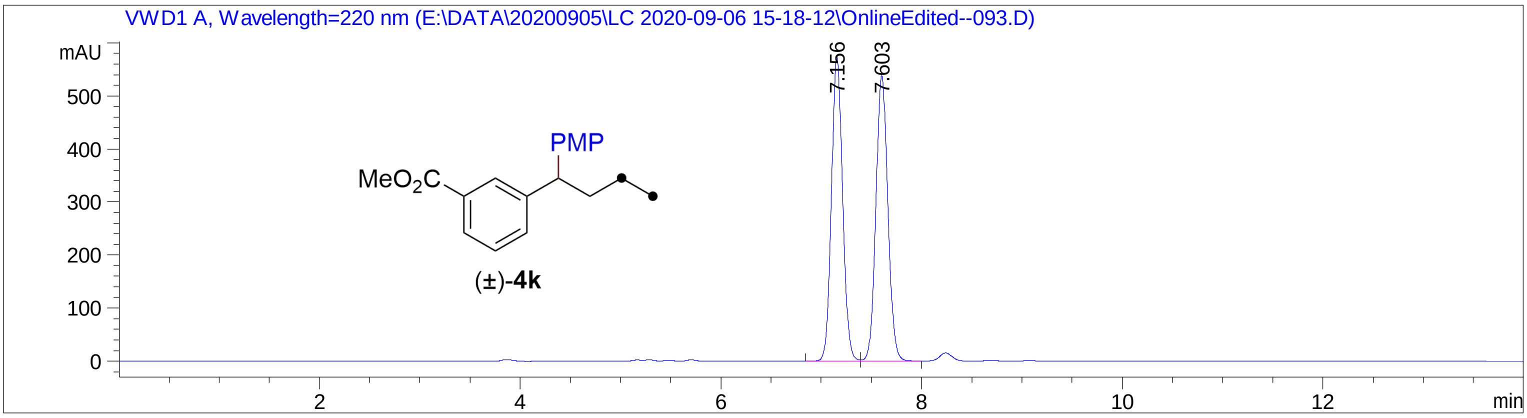

===
Area Percent Report
===

Sorted By : Signal
Multiplier : 1.0000
Dilution : 1.0000
Do not use Multiplier & Dilution Factor with ISTDs

Signal 1: VWD1 A, Wavelength=220 nm

Peak #	RetTime [min]	Type	Width [min]	Area [mAU*s]	Height [mAU]	Area %
1	7.156	BV	0.1194	4448.94434	574.02832	49.9165
2	7.603	VV	0.1287	4463.82129	538.47260	50.0835

Totals : 8912.76563 1112.50092

===
*** End of Report ***

Data File E:\DATA\20200905\LC 2020-09-06 15-18-12\OnlineEdited--094.D
Sample Name: YH-17-134-EE

===

Acq. Operator : SYSTEM Seq. Line : 94
Acq. Instrument : HPLC1260 Location : P1-A6
Injection Date : 9/8/2020 3:03:57 PM Inj : 1
Inj Volume : 3.000 µl
Different Inj Volume from Sample Entry! Actual Inj Volume : 0.500 µl
Acq. Method : E:\DATA\20200905\LC 2020-09-06 15-18-12\5IPA_25_8_2.M
Last changed : 9/8/2020 2:03:49 PM by SYSTEM
Analysis Method : E:\DATA\20200905\LC 2020-09-06 15-18-12\5IPA_25_8_2.M (Sequence Method)
Last changed : 9/8/2020 3:27:14 PM by SYSTEM
(modified after loading)
Additional Info : Peak(s) manually integrated

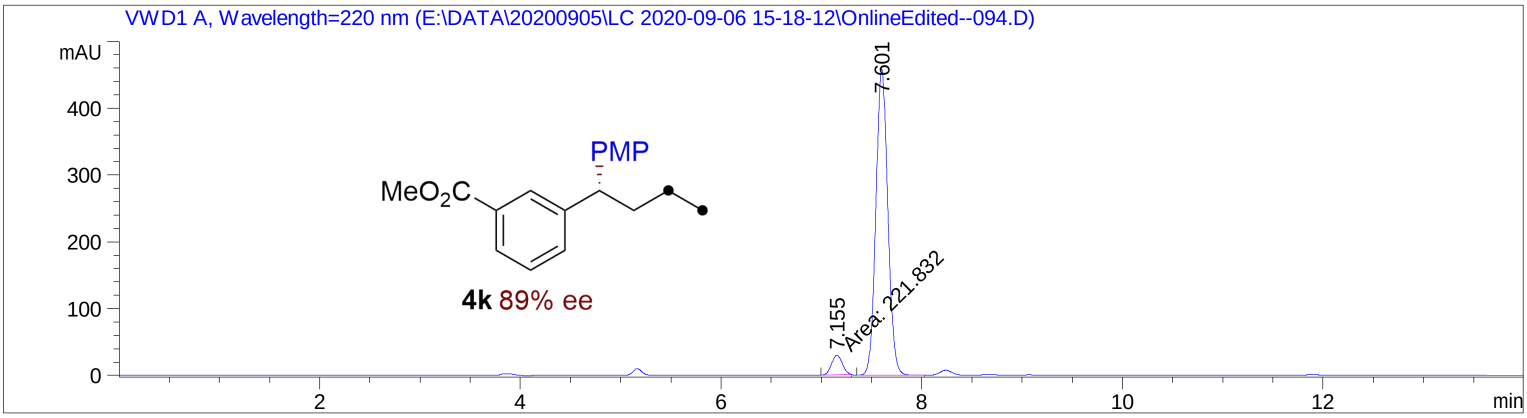

===

Area Percent Report

===

Sorted By : Signal
Multiplier : 1.0000
Dilution : 1.0000
Do not use Multiplier & Dilution Factor with ISTDs

Signal 1: VWD1 A, Wavelength=220 nm

Peak #	RetTime [min]	Type	Width [min]	Area [mAU*s]	Height [mAU]	Area %
1	7.155	MM	0.1241	221.83202	29.79492	5.5158
2	7.601	VB	0.1277	3799.90894	458.56464	94.4842

Totals : 4021.74095 488.35956

===

*** End of Report ***

Data File E:\DATA\20200905\LC 2020-09-09 14-34-17\OnlineEdited--013.D
Sample Name: YH-17-141-RAC

===
Acq. Operator   : SYSTEM   Seq. Line : 13
Acq. Instrument : HPLC1260   Location :   P1-A2
Injection Date : 9/9/2020 6:13:58 PM   Inj :   1
                                                 Inj Volume : 3.000 µl
Different Inj Volume from Sample Entry! Actual Inj Volume : 1.000 µl
Acq. Method   : E:\DATA\20200905\LC 2020-09-09 14-34-17\5IPA_20_8_3.M
Last changed   : 9/9/2020 6:07:48 PM by SYSTEM
Analysis Method : E:\DATA\20200905\LC 2020-09-09 14-34-17\5IPA_20_8_3.M (Sequence Method)
Last changed   : 9/9/2020 7:18:56 PM by SYSTEM
                  (modified after loading)
Additional Info : Peak(s) manually integrated

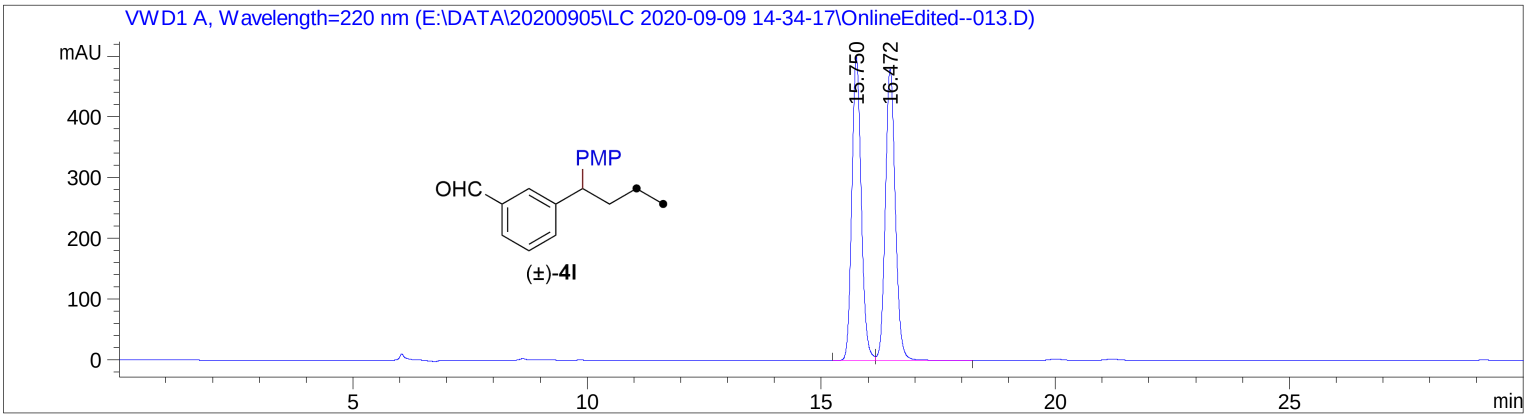

===
Area Percent Report
===

Sorted By   :   Signal
Multiplier   :   1.0000
Dilution   :   1.0000
Do not use Multiplier & Dilution Factor with ISTDs

Signal 1: VWD1 A, Wavelength=220 nm

Peak #	RetTime [min]	Type	Width [min]	Area [mAU*s]	Height [mAU]	Area %
1	15.750	BV	0.2154	6964.64014	500.38995	49.5307
2	16.472	VB	0.2285	7096.62207	479.83856	50.4693

Totals :   1.40613e4   980.22852

===
*** End of Report ***

Data File E:\DATA\20200905\LC 2020-09-09 14-34-17\OnlineEdited--014.D
Sample Name: YH-17-141-EE

===
Acq. Operator : SYSTEM Seq. Line : 14
Acq. Instrument : HPLC1260 Location : P1-A4
Injection Date : 9/9/2020 6:44:43 PM Inj : 1
Inj Volume : 3.000 µl
Different Inj Volume from Sample Entry! Actual Inj Volume : 1.000 µl
Acq. Method : E:\DATA\20200905\LC 2020-09-09 14-34-17\5IPA_20_8_3.M
Last changed : 9/9/2020 6:07:48 PM by SYSTEM
Analysis Method : E:\DATA\20200905\LC 2020-09-09 14-34-17\5IPA_20_8_3.M (Sequence Method)
Last changed : 9/9/2020 7:18:56 PM by SYSTEM
(modified after loading)
Additional Info : Peak(s) manually integrated

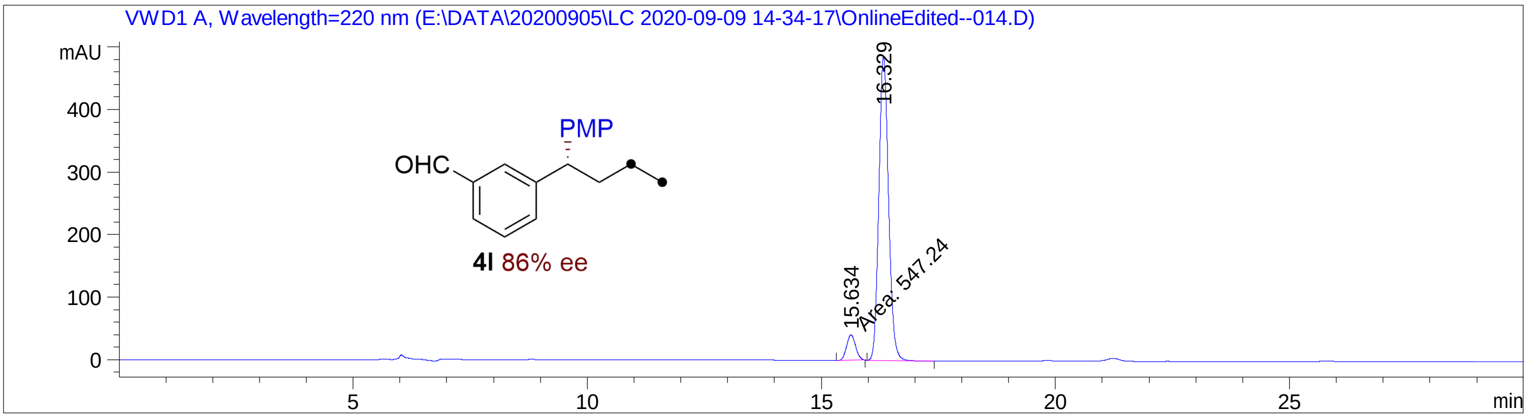

===
Area Percent Report
===

Sorted By : Signal
Multiplier : 1.0000
Dilution : 1.0000
Do not use Multiplier & Dilution Factor with ISTDs

Signal 1: VWD1 A, Wavelength=220 nm

Peak #	RetTime [min]	Type	Width [min]	Area [mAU*s]	Height [mAU]	Area %
1	15.634	MM	0.2229	547.23956	40.90938	7.2131
2	16.329	VB	0.2238	7039.51855	486.58237	92.7869

Totals : 7586.75812 527.49175

===
*** End of Report ***

Data File E:\DATA\20200905\LC 2020-09-11 08-36-22\OnlineEdited--037.D
Sample Name: YH-17-161-RAC

===

```
Acq. Operator   : SYSTEM                          Seq. Line :  37
Acq. Instrument : HPLC1260                         Location :   P1-A1
Injection Date  : 9/12/2020 3:14:35 AM                  Inj :   1
                                                 Inj Volume : 3.000 µl
Different Inj Volume from Sample Entry!  Actual Inj Volume : 1.000 µl
Acq. Method     : E:\DATA\20200905\LC 2020-09-11 08-36-22\1ETOH_60_5_3.M
Last changed    : 9/11/2020 8:29:14 PM by SYSTEM
Analysis Method : E:\DATA\20200905\LC 2020-09-11 08-36-22\1ETOH_60_5_3.M (Sequence Method)
Last changed    : 9/15/2020 6:39:26 PM by SYSTEM
                  (modified after loading)
Additional Info : Peak(s) manually integrated
```

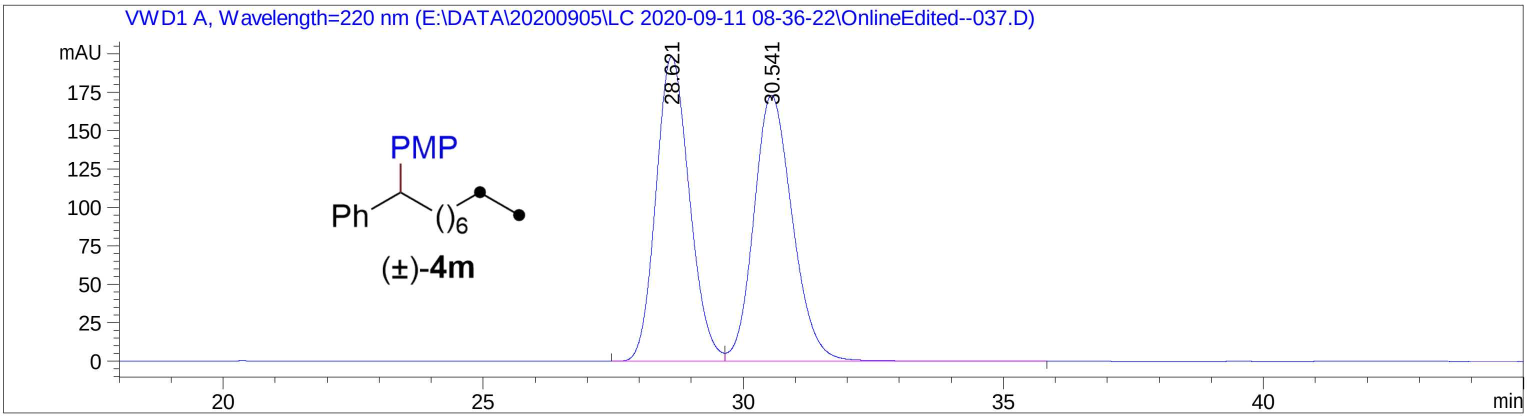

===
Area Percent Report
===

Sorted By : Signal
Multiplier : 1.0000
Dilution : 1.0000
Do not use Multiplier & Dilution Factor with ISTDs

Signal 1: VWD1 A, Wavelength=220 nm

Peak #	RetTime [min]	Type	Width [min]	Area [mAU*s]	Height [mAU]	Area %
1	28.621	BV	0.6944	8867.06055	198.40100	49.6974
2	30.541	VB	0.7973	8975.05762	173.28355	50.3026

Totals : 1.78421e4 371.68456

===
*** End of Report ***

Data File E:\DATA\20200905\LC 2020-09-11 08-36-22\OnlineEdited--038.D
Sample Name: YH-17-161-EE1

===

Acq. Operator : SYSTEM Seq. Line : 38
Acq. Instrument : HPLC1260 Location : P1-A2
Injection Date : 9/12/2020 4:15:20 AM Inj : 1
Inj Volume : 3.000 µl
Different Inj Volume from Sample Entry! Actual Inj Volume : 1.000 µl
Acq. Method : E:\DATA\20200905\LC 2020-09-11 08-36-22\1ETOH_60_5_3.M
Last changed : 9/11/2020 8:29:14 PM by SYSTEM
Analysis Method : E:\DATA\20200905\LC 2020-09-11 08-36-22\1ETOH_60_5_3.M (Sequence Method)
Last changed : 9/15/2020 6:39:26 PM by SYSTEM
(modified after loading)
Additional Info : Peak(s) manually integrated

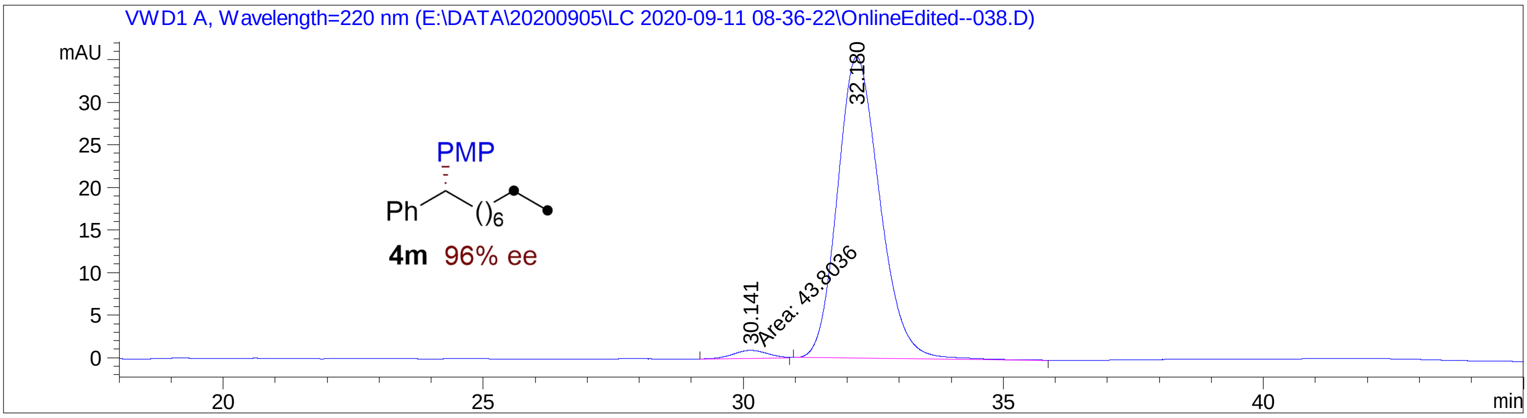

===

Area Percent Report

===

Sorted By : Signal
Multiplier : 1.0000
Dilution : 1.0000
Do not use Multiplier & Dilution Factor with ISTDs

Signal 1: VWD1 A, Wavelength=220 nm

Peak #	RetTime [min]	Type	Width [min]	Area [mAU*s]	Height [mAU]	Area %
1	30.141	MM	0.7755	43.80361	9.41372e-1	2.1410
2	32.180	BB	0.8646	2002.12549	35.41506	97.8590

Totals : 2045.92910 36.35643

===

*** End of Report ***

Data File E:\DATA\20200905\LC 2020-09-11 08-36-22\OnlineEdited--039.D
Sample Name: YH-17-161-EE2

===

Acq. Operator : SYSTEM Seq. Line : 39
Acq. Instrument : HPLC1260 Location : P1-A3
Injection Date : 9/12/2020 5:16:05 AM Inj : 1
Inj Volume : 3.000 µl
Different Inj Volume from Sample Entry! Actual Inj Volume : 1.000 µl
Acq. Method : E:\DATA\20200905\LC 2020-09-11 08-36-22\1ETOH_60_5_3.M
Last changed : 9/11/2020 8:29:14 PM by SYSTEM
Analysis Method : E:\DATA\20200905\LC 2020-09-11 08-36-22\1ETOH_60_5_3.M (Sequence Method)
Last changed : 9/15/2020 6:39:26 PM by SYSTEM
(modified after loading)

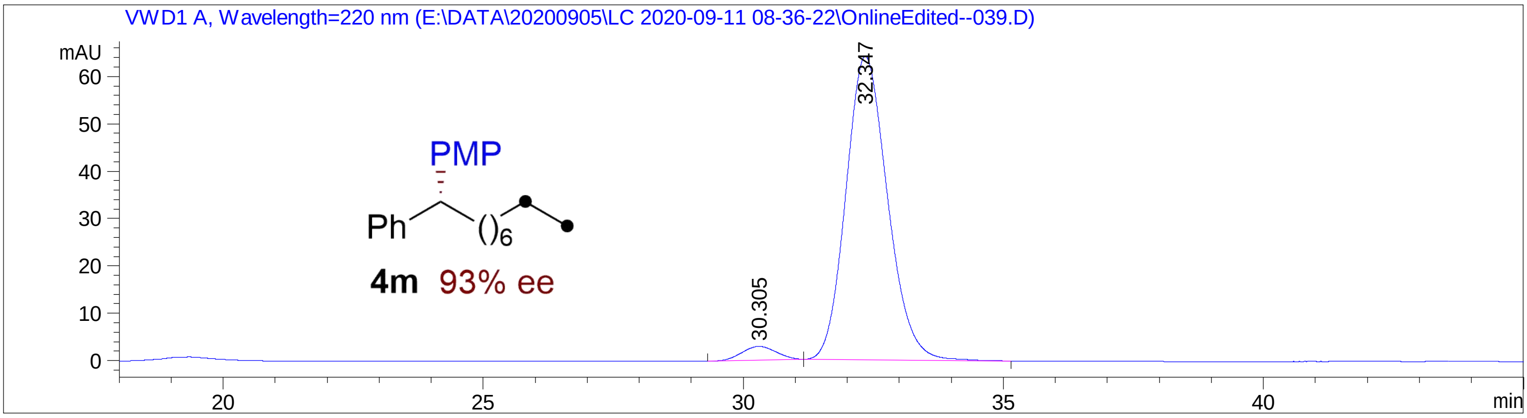

===

Area Percent Report

===

Sorted By : Signal
Multiplier : 1.0000
Dilution : 1.0000
Do not use Multiplier & Dilution Factor with ISTDs

Signal 1: VWD1 A, Wavelength=220 nm

Peak #	RetTime [min]	Type	Width [min]	Area [mAU*s]	Height [mAU]	Area %
1	30.305	BB	0.6861	134.16422	2.91444	3.6283
2	32.347	BB	0.8588	3563.58740	64.08324	96.3717

Totals : 3697.75162 66.99769

===

*** End of Report ***

Data File E:\DATA\20200905\LC 2020-09-13 18-37-17\OnlineEdited--139.D
Sample Name: YH-17-173-RAC

===

Acq. Operator : SYSTEM   Seq. Line : 139
Acq. Instrument : HPLC1260   Location : P1-B1
Injection Date : 9/16/2020 9:46:23 PM   Inj : 1
                                          Inj Volume : 3.000 µl
Different Inj Volume from Sample Entry! Actual Inj Volume : 0.200 µl
Acq. Method : E:\DATA\20200905\LC 2020-09-13 18-37-17\20EtOH_20_5_1.M
Last changed : 9/16/2020 9:10:36 PM by SYSTEM
Analysis Method : E:\DATA\20200905\LC 2020-09-13 18-37-17\20EtOH_20_5_1.M (Sequence Method)
Last changed : 9/17/2020 9:24:25 AM by SYSTEM
(modified after loading)
Additional Info : Peak(s) manually integrated

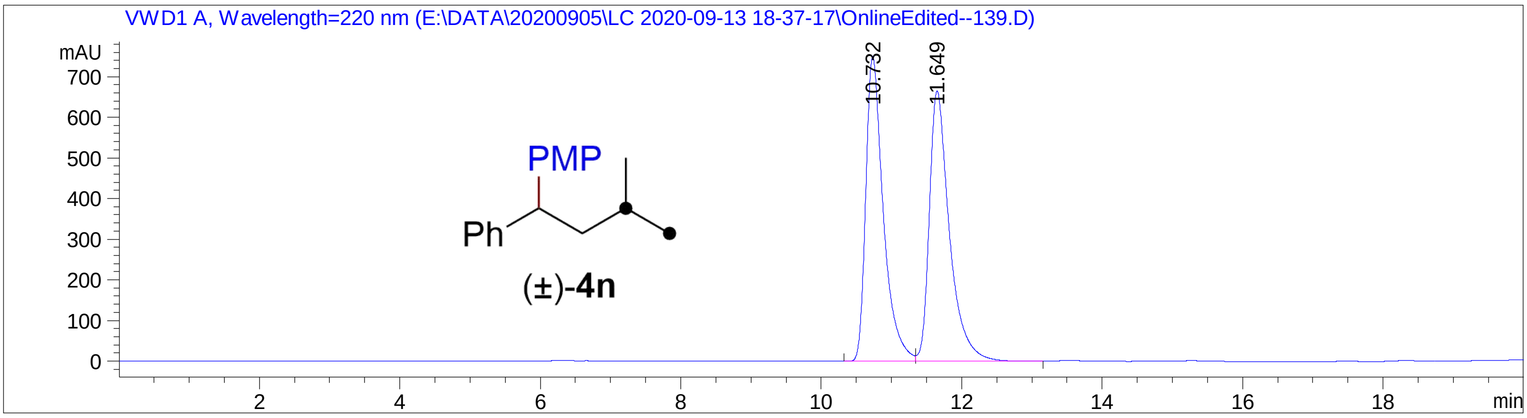

===

Area Percent Report

===

Sorted By : Signal
Multiplier : 1.0000
Dilution : 1.0000
Do not use Multiplier & Dilution Factor with ISTDs

Signal 1: VWD1 A, Wavelength=220 nm

Peak #	RetTime [min]	Type	Width [min]	Area [mAU*s]	Height [mAU]	Area %
1	10.732	BV	0.2598	1.30364e4	749.81757	49.6103
2	11.649	VB	0.2951	1.32412e4	665.68225	50.3897

Totals : 2.62776e4 1415.49982

===

*** End of Report ***

Data File E:\DATA\20200905\LC 2020-09-13 18-37-17\OnlineEdited--140.D
Sample Name: YH-17-173-EE

===

Acq. Operator : SYSTEM Seq. Line : 140
Acq. Instrument : HPLC1260 Location : P1-B2
Injection Date : 9/16/2020 10:07:12 PM Inj : 1
Inj Volume : 3.000 µl
Different Inj Volume from Sample Entry! Actual Inj Volume : 8.000 µl
Acq. Method : E:\DATA\20200905\LC 2020-09-13 18-37-17\20EtOH_20_5_1.M
Last changed : 9/16/2020 9:10:36 PM by SYSTEM
Analysis Method : E:\DATA\20200905\LC 2020-09-13 18-37-17\20EtOH_20_5_1.M (Sequence Method)
Last changed : 9/17/2020 9:24:25 AM by SYSTEM
(modified after loading)
Additional Info : Peak(s) manually integrated

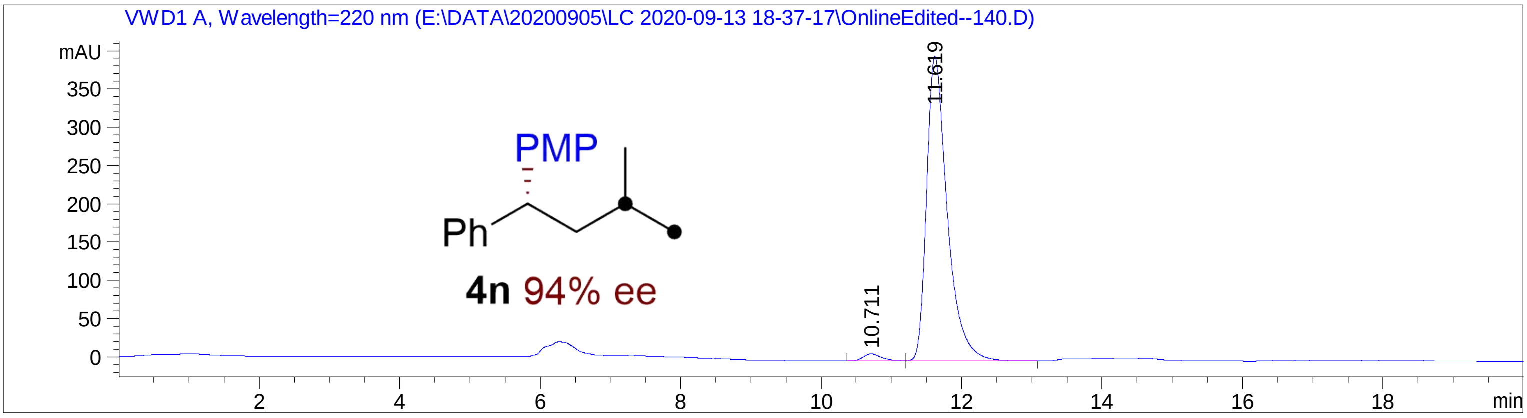

===

Area Percent Report

===

Sorted By : Signal
Multiplier : 1.0000
Dilution : 1.0000
Do not use Multiplier & Dilution Factor with ISTDs

Signal 1: VWD1 A, Wavelength=220 nm

Peak #	RetTime [min]	Type	Width [min]	Area [mAU*s]	Height [mAU]	Area %
1	10.711	BV	0.2739	165.10509	9.17757	1.9828
2	11.619	VB	0.3072	8161.65186	398.03119	98.0172

Totals : 8326.75694 407.20876

===

*** End of Report ***

===

Acq. Operator : SYSTEM Seq. Line : 108
Acq. Instrument : HPLC1260 Location : P1-C1
Injection Date : 9/16/2020 12:07:31 AM Inj : 1
Inj Volume : 3.000 µl
Different Inj Volume from Sample Entry! Actual Inj Volume : 1.000 µl
Acq. Method : E:\DATA\20200905\LC 2020-09-13 18-37-17\10IPA_60_8_1.M
Last changed : 9/15/2020 3:44:04 PM by SYSTEM
Analysis Method : E:\DATA\20200905\LC 2020-09-13 18-37-17\10IPA_60_8_1.M (Sequence Method)
Last changed : 9/16/2020 9:18:31 AM by SYSTEM
(modified after loading)
Additional Info : Peak(s) manually integrated

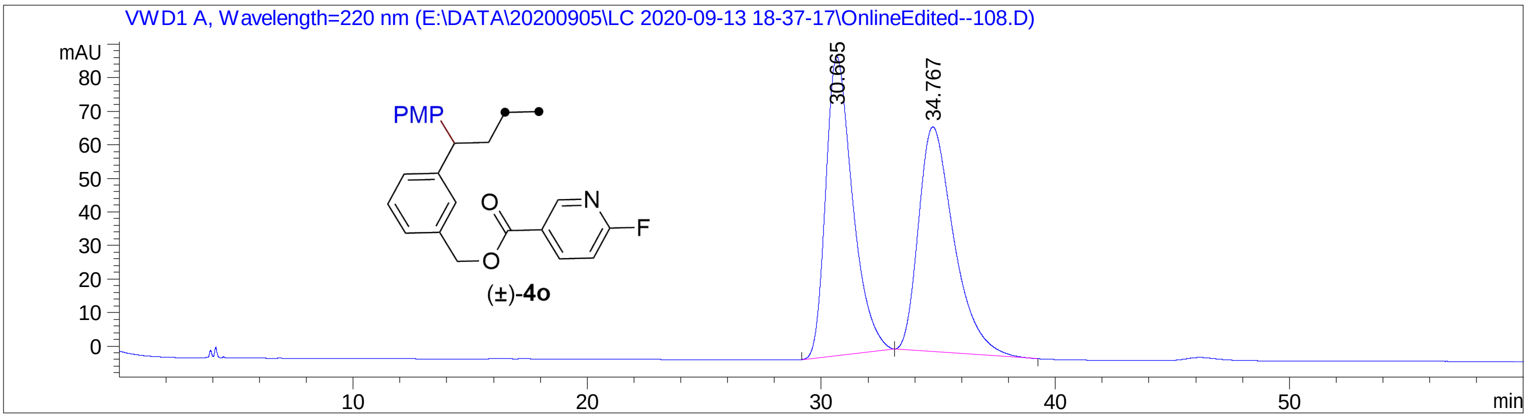

===

Area Percent Report

===

Sorted By : Signal
Multiplier : 1.0000
Dilution : 1.0000
Do not use Multiplier & Dilution Factor with ISTDs

Signal 1: VWD1 A, Wavelength=220 nm

Peak #	RetTime [min]	Type	Width [min]	Area [mAU*s]	Height [mAU]	Area %
1	30.665	BB	1.2106	7133.66357	89.14415	50.2737
2	34.767	BB	1.5855	7056.00000	67.01034	49.7263

Totals : 1.41897e4 156.15449

===

*** End of Report ***

Data File E:\DATA\20200905\LC 2020-09-13 18-37-17\OnlineEdited--109.D
Sample Name: YH-17-166-EE

===
Acq. Operator   : SYSTEM   Seq. Line : 109
Acq. Instrument : HPLC1260   Location :   P1-C2
Injection Date : 9/16/2020 1:08:16 AM   Inj :   1
                                                  Inj Volume : 3.000 µl
Different Inj Volume from Sample Entry! Actual Inj Volume : 1.000 µl
Acq. Method   : E:\DATA\20200905\LC 2020-09-13 18-37-17\10IPA_60_8_1.M
Last changed   : 9/15/2020 3:44:04 PM by SYSTEM
Analysis Method : E:\DATA\20200905\LC 2020-09-13 18-37-17\10IPA_60_8_1.M (Sequence Method)
Last changed   : 9/16/2020 9:18:31 AM by SYSTEM
                  (modified after loading)
Additional Info : Peak(s) manually integrated

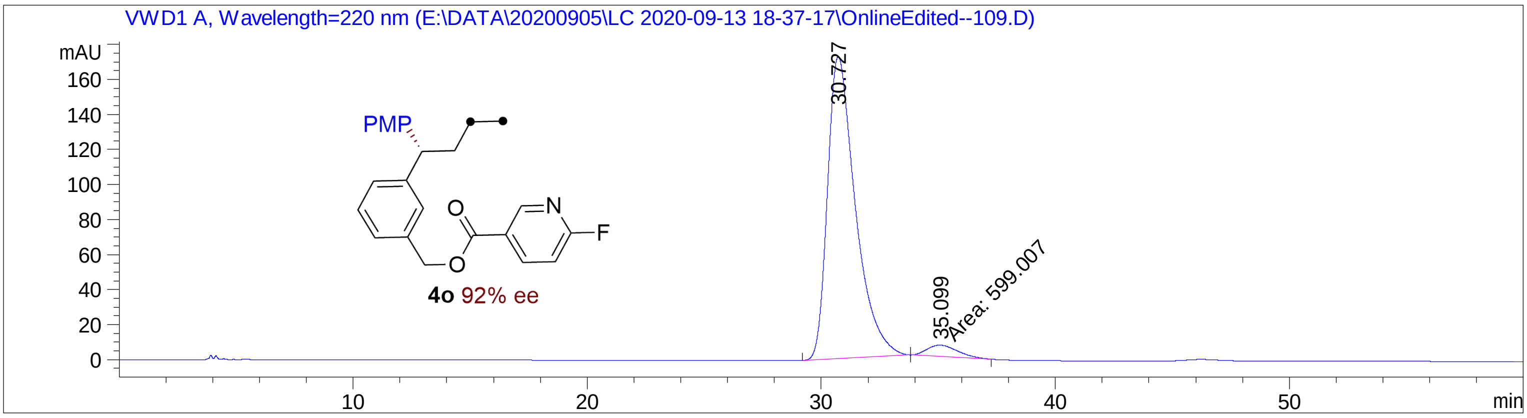

===
Area Percent Report
===

Sorted By   :   Signal
Multiplier   :   1.0000
Dilution   :   1.0000
Do not use Multiplier & Dilution Factor with ISTDs

Signal 1: VWD1 A, Wavelength=220 nm

Peak #	RetTime [min]	Type	Width [min]	Area [mAU*s]	Height [mAU]	Area %
1	30.727	BB	1.2444	1.42822e4	172.41908	95.9748
2	35.099	MF	1.5299	599.00665	6.52567	4.0252

Totals :   1.48812e4   178.94475

===
*** End of Report ***

Data File E:\DATA\20200905\LC 2020-09-13 18-37-17\OnlineEdited--023.D
Sample Name: YH-17-154-RAC

===
Acq. Operator   : SYSTEM   Seq. Line : 23
Acq. Instrument : HPLC1260   Location :   P1-A1
Injection Date : 9/14/2020 3:37:20 AM   Inj :   1
                                                 Inj Volume : 3.000 µl
Different Inj Volume from Sample Entry! Actual Inj Volume : 1.000 µl
Acq. Method   : E:\DATA\20200905\LC 2020-09-13 18-37-17\20IPA_25_5_1.M
Last changed   : 9/13/2020 8:42:18 PM by SYSTEM
Analysis Method : E:\DATA\20200905\LC 2020-09-13 18-37-17\20IPA_25_5_1.M (Sequence Method)
Last changed   : 9/14/2020 8:52:04 AM by SYSTEM
                  (modified after loading)
Additional Info : Peak(s) manually integrated

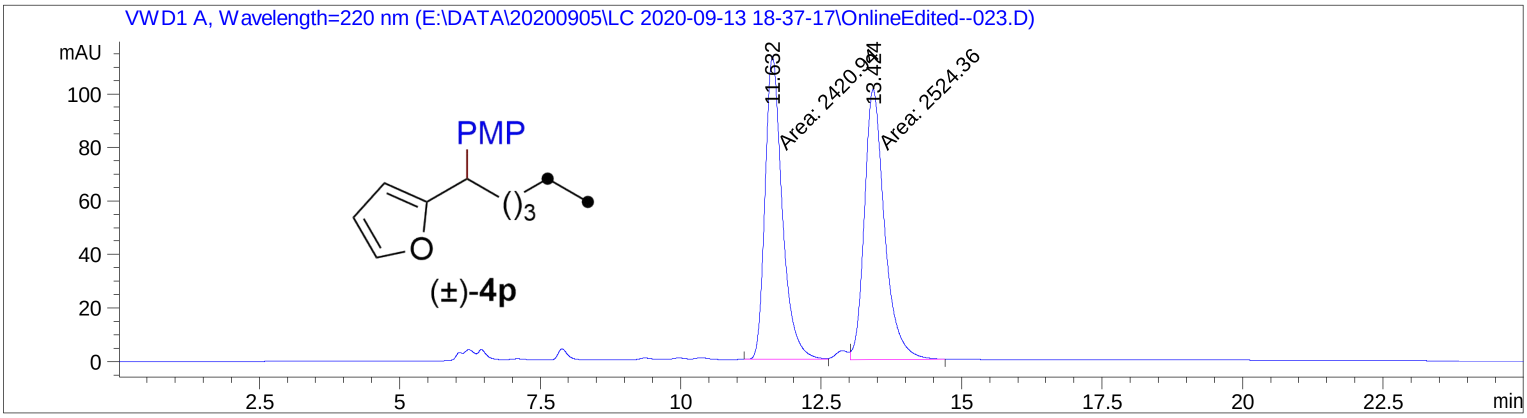

===
Area Percent Report
===

Sorted By : Signal
Multiplier : 1.0000
Dilution : 1.0000
Do not use Multiplier & Dilution Factor with ISTDs

Signal 1: VWD1 A, Wavelength=220 nm

Peak #	RetTime [min]	Type	Width [min]	Area [mAU*s]	Height [mAU]	Area %
1	11.632	MF	0.3565	2420.94312	113.18167	48.9544
2	13.424	MM	0.4165	2524.35938	101.00546	51.0456

Totals : 4945.30249 214.18713

===
*** End of Report ***

Data File E:\DATA\20200905\LC 2020-09-13 18-37-17\OnlineEdited--024.D
Sample Name: YH-17-154-EE

===

Acq. Operator : SYSTEM Seq. Line : 24
Acq. Instrument : HPLC1260 Location : P1-A2
Injection Date : 9/14/2020 4:03:06 AM Inj : 1
Inj Volume : 3.000 µl
Different Inj Volume from Sample Entry! Actual Inj Volume : 1.000 µl
Acq. Method : E:\DATA\20200905\LC 2020-09-13 18-37-17\20IPA_25_5_1.M
Last changed : 9/13/2020 8:42:18 PM by SYSTEM
Analysis Method : E:\DATA\20200905\LC 2020-09-13 18-37-17\20IPA_25_5_1.M (Sequence Method)
Last changed : 9/14/2020 8:52:04 AM by SYSTEM
(modified after loading)
Additional Info : Peak(s) manually integrated

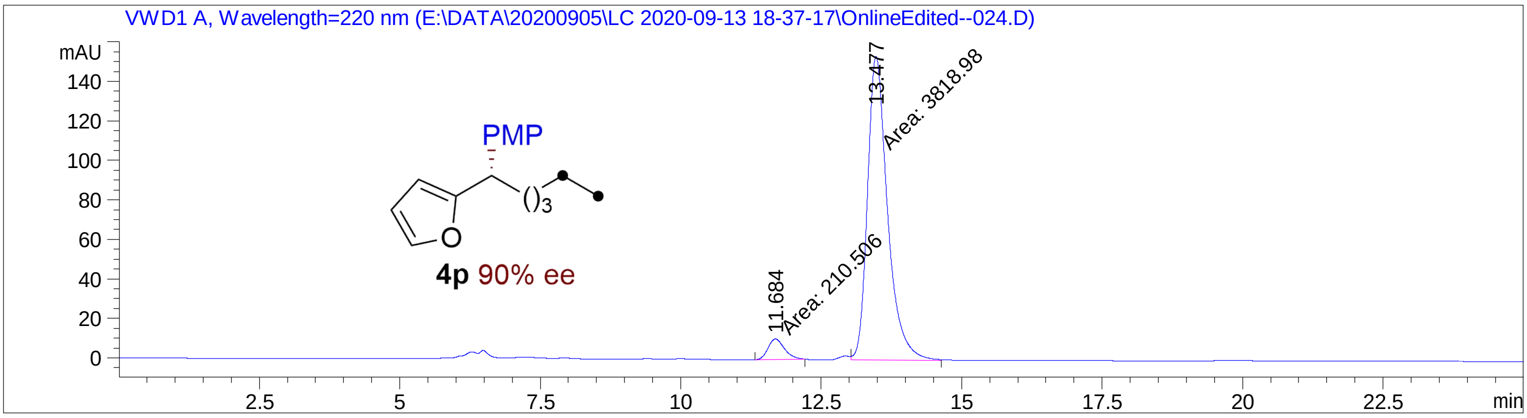

===

Area Percent Report

===

Sorted By : Signal
Multiplier : 1.0000
Dilution : 1.0000
Do not use Multiplier & Dilution Factor with ISTDs

Signal 1: VWD1 A, Wavelength=220 nm

Peak #	RetTime [min]	Type	Width [min]	Area [mAU*s]	Height [mAU]	Area %
1	11.684	MM	0.3366	210.50558	10.42404	5.2241
2	13.477	MF	0.4143	3818.98242	153.64587	94.7759

Totals : 4029.48801 164.06991

===

*** End of Report ***

===

Acq. Operator : SYSTEM Seq. Line : 25
Acq. Instrument : HPLC1260 Location : P1-A3
Injection Date : 9/14/2020 4:28:50 AM Inj : 1
Inj Volume : 3.000 µl
Different Inj Volume from Sample Entry! Actual Inj Volume : 1.000 µl
Acq. Method : E:\DATA\20200905\LC 2020-09-13 18-37-17\20IPA_25_5_1.M
Last changed : 9/13/2020 8:42:18 PM by SYSTEM
Analysis Method : E:\DATA\20200905\LC 2020-09-13 18-37-17\20IPA_25_5_1.M (Sequence Method)
Last changed : 9/14/2020 8:48:48 AM by SYSTEM
(modified after loading)
Additional Info : Peak(s) manually integrated

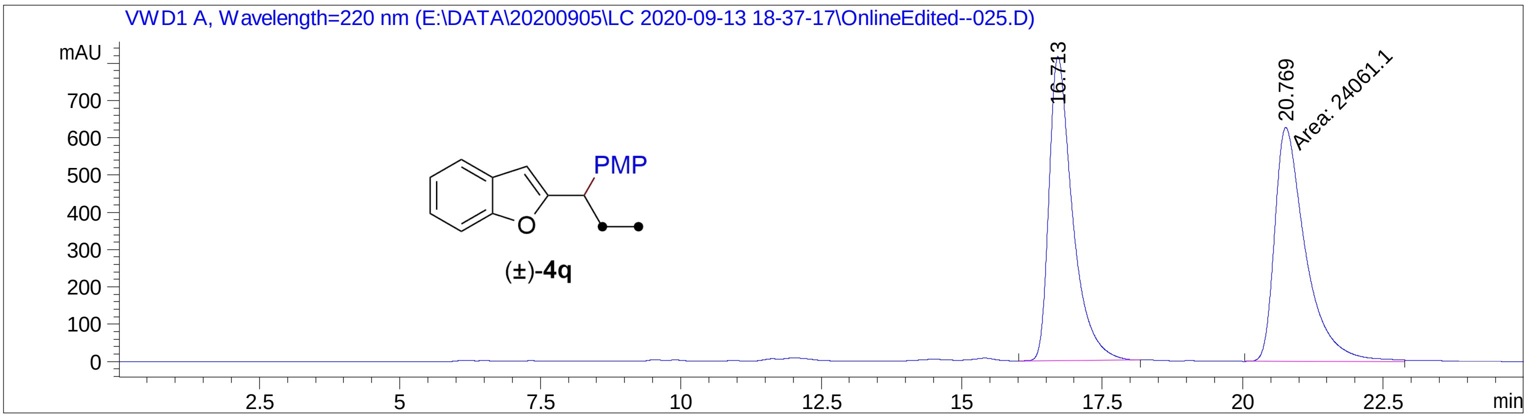

===

Area Percent Report

===

Sorted By : Signal
Multiplier : 1.0000
Dilution : 1.0000
Do not use Multiplier & Dilution Factor with ISTDs

Signal 1: VWD1 A, Wavelength=220 nm

Peak #	RetTime [min]	Type	Width [min]	Area [mAU*s]	Height [mAU]	Area %
1	16.713	BB	0.4327	2.37441e4	815.48669	49.6684
2	20.769	MF	0.6390	2.40611e4	627.55438	50.3316

Totals : 4.78053e4 1443.04108

===

*** End of Report ***

Data File E:\DATA\20200905\LC 2020-09-13 18-37-17\OnlineEdited--026.D
Sample Name: YH-17-156-EE

===

Acq. Operator : SYSTEM Seq. Line : 26
Acq. Instrument : HPLC1260 Location : P1-A4
Injection Date : 9/14/2020 4:54:34 AM Inj : 1
Inj Volume : 3.000 µl
Different Inj Volume from Sample Entry! Actual Inj Volume : 1.000 µl
Acq. Method : E:\DATA\20200905\LC 2020-09-13 18-37-17\20IPA_25_5_1.M
Last changed : 9/13/2020 8:42:18 PM by SYSTEM
Analysis Method : E:\DATA\20200905\LC 2020-09-13 18-37-17\20IPA_25_5_1.M (Sequence Method)
Last changed : 9/14/2020 8:48:48 AM by SYSTEM
(modified after loading)
Additional Info : Peak(s) manually integrated

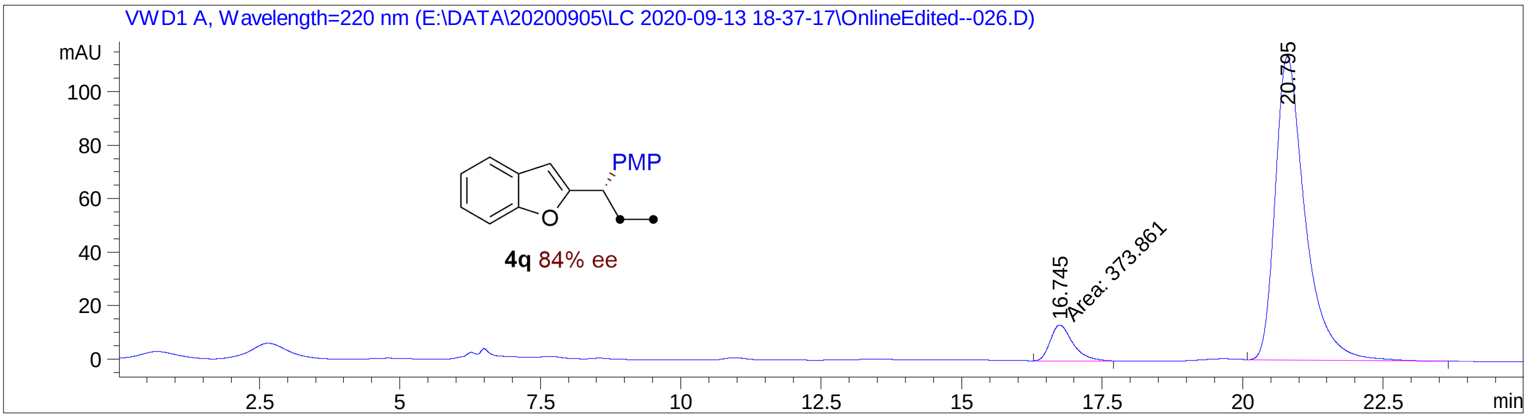

===

Area Percent Report

===

Sorted By : Signal
Multiplier : 1.0000
Dilution : 1.0000
Do not use Multiplier & Dilution Factor with ISTDs

Signal 1: VWD1 A, Wavelength=220 nm

Peak #	RetTime [min]	Type	Width [min]	Area [mAU*s]	Height [mAU]	Area %
1	16.745	MM	0.4636	373.86090	13.43970	8.1749
2	20.795	BB	0.5548	4199.41406	113.45737	91.8251

Totals : 4573.27496 126.89708

===

*** End of Report ***

Data File E:\DATA\20200905\LC 2020-09-11 08-36-22\OnlineEdited--065.D
Sample Name: YH-17-151-RAC

===

Acq. Operator : SYSTEM Seq. Line : 65
Acq. Instrument : HPLC1260 Location : P1-B7
Injection Date : 9/12/2020 6:10:32 PM Inj : 1
Inj Volume : 3.000 µl
Different Inj Volume from Sample Entry! Actual Inj Volume : 0.100 µl
Acq. Method : E:\DATA\20200905\LC 2020-09-11 08-36-22\30IPA_25_8_1.M
Last changed : 9/11/2020 7:15:37 PM by SYSTEM
Analysis Method : E:\DATA\20200905\LC 2020-09-11 08-36-22\30IPA_25_8_1.M (Sequence Method)
Last changed : 11/25/2021 4:57:15 PM by SYSTEM
(modified after loading)
Additional Info : Peak(s) manually integrated

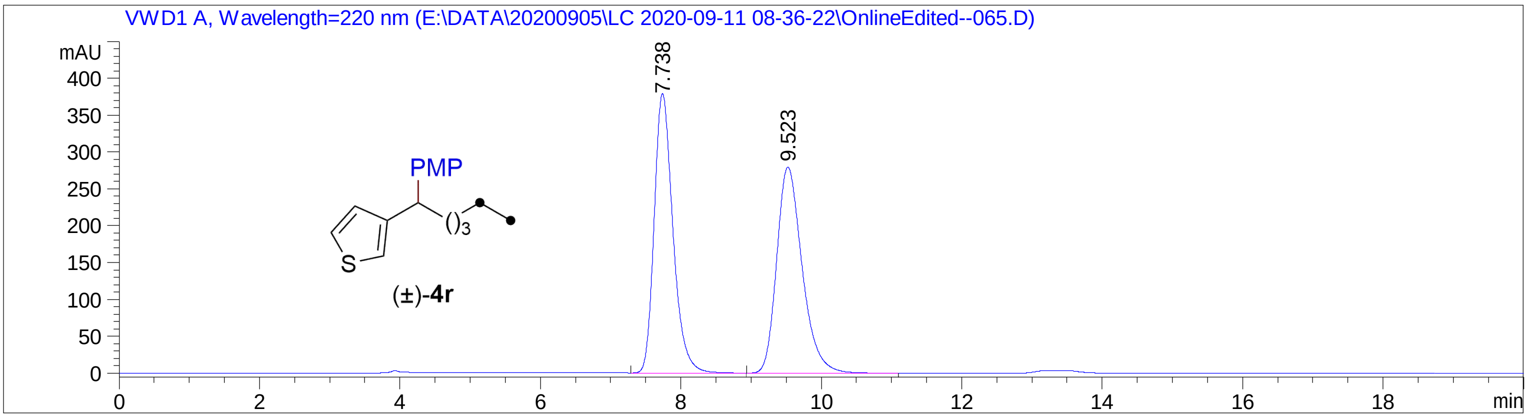

===

Area Percent Report

===

Sorted By : Signal
Multiplier : 1.0000
Dilution : 1.0000
Do not use Multiplier & Dilution Factor with ISTDs

Signal 1: VWD1 A, Wavelength=220 nm

Peak #	RetTime [min]	Type	Width [min]	Area [mAU*s]	Height [mAU]	Area %
1	7.738	VB	0.2845	7035.99609	379.18643	49.9793
2	9.523	BB	0.3858	7041.82764	279.59964	50.0207

Totals : 1.40778e4 658.78607

===

*** End of Report ***

Data File E:\DATA\20200905\LC 2020-09-11 08-36-22\OnlineEdited--066.D
Sample Name: YH-17-151-EE

===

Acq. Operator : SYSTEM Seq. Line : 66
Acq. Instrument : HPLC1260 Location : P1-B8
Injection Date : 9/12/2020 6:36:16 PM Inj : 1
Inj Volume : 3.000 µl
Different Inj Volume from Sample Entry! Actual Inj Volume : 1.000 µl
Acq. Method : E:\DATA\20200905\LC 2020-09-11 08-36-22\30IPA_25_8_1.M
Last changed : 9/11/2020 7:15:37 PM by SYSTEM
Analysis Method : E:\DATA\20200905\LC 2020-09-11 08-36-22\30IPA_25_8_1.M (Sequence Method)
Last changed : 11/25/2021 4:58:08 PM by SYSTEM
(modified after loading)
Additional Info : Peak(s) manually integrated

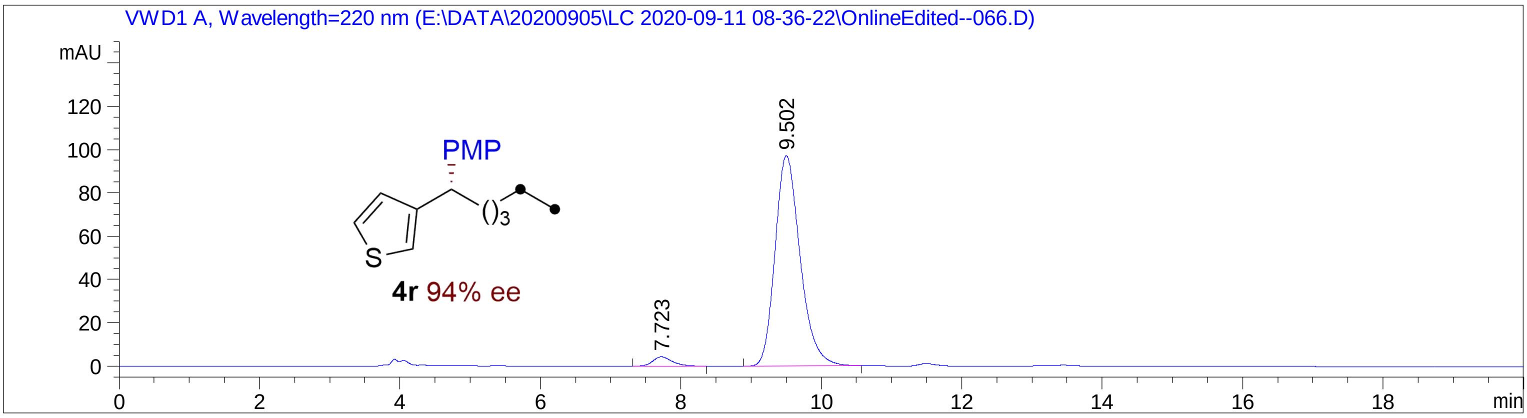

===

Area Percent Report

===

Sorted By : Signal
Multiplier : 1.0000
Dilution : 1.0000
Do not use Multiplier & Dilution Factor with ISTDs

Signal 1: VWD1 A, Wavelength=220 nm

Peak #	RetTime [min]	Type	Width [min]	Area [mAU*s]	Height [mAU]	Area %
1	7.723	BB	0.2825	79.95707	4.26963	3.2048
2	9.502	BB	0.3809	2414.99536	97.17395	96.7952
Totals :				2494.95243	101.44358	

===

*** End of Report ***

Data File E:\DATA\20200905\LC 2020-09-13 18-37-17\OnlineEdited--061.D
Sample Name: YH-17-159-AD

===

Acq. Operator : SYSTEM Seq. Line : 61
Acq. Instrument : HPLC1260 Location : P1-B4
Injection Date : 9/14/2020 7:56:12 PM Inj : 1
Inj Volume : 3.000 µl
Different Inj Volume from Sample Entry! Actual Inj Volume : 1.000 µl
Acq. Method : E:\DATA\20200905\LC 2020-09-13 18-37-17\10ETOH_25_10_3.M
Last changed : 9/13/2020 8:50:01 PM by SYSTEM
Analysis Method : E:\DATA\20200905\LC 2020-09-13 18-37-17\10ETOH_25_10_3.M (Sequence Method)
Last changed : 9/14/2020 8:35:55 PM by SYSTEM
(modified after loading)
Additional Info : Peak(s) manually integrated

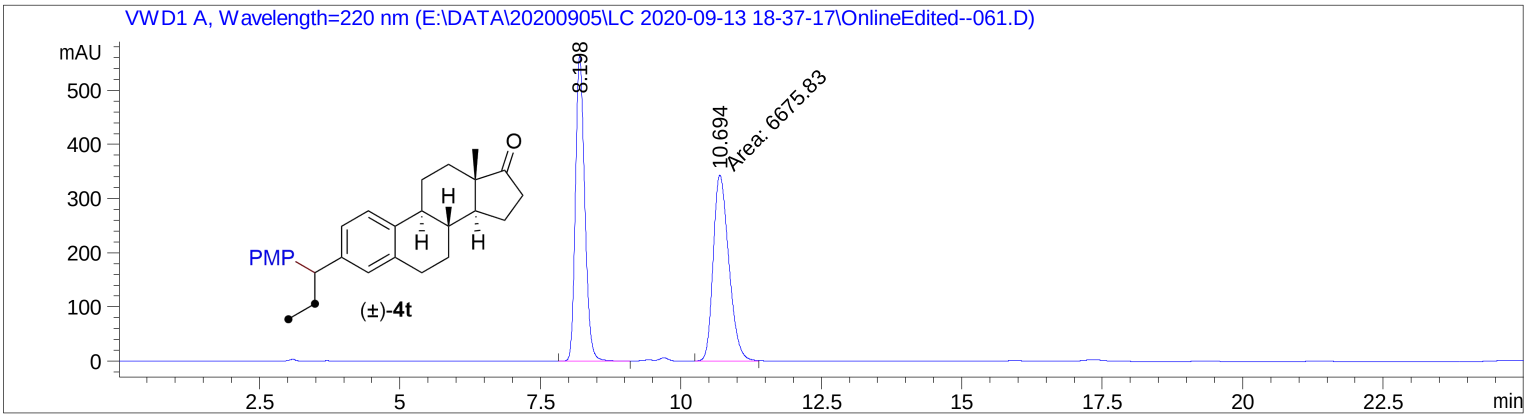

===

Area Percent Report

===

Sorted By : Signal
Multiplier : 1.0000
Dilution : 1.0000
Do not use Multiplier & Dilution Factor with ISTDs

Signal 1: VWD1 A, Wavelength=220 nm

Peak #	RetTime [min]	Type	Width [min]	Area [mAU*s]	Height [mAU]	Area %
1	8.198	BB	0.1780	6505.39844	562.11786	49.3535
2	10.694	FM	0.3238	6675.82568	343.59470	50.6465

Totals : 1.31812e4 905.71255

===

*** End of Report ***

Data File E:\DATA\20200905\LC 2020-09-13 18-37-17\OnlineEdited--062.D
Sample Name: YH-17-159-EE

===

Acq. Operator : SYSTEM Seq. Line : 62
Acq. Instrument : HPLC1260 Location : P1-A3
Injection Date : 9/14/2020 8:21:57 PM Inj : 1
Inj Volume : 3.000 µl
Different Inj Volume from Sample Entry! Actual Inj Volume : 1.000 µl
Acq. Method : E:\DATA\20200905\LC 2020-09-13 18-37-17\10ETOH_25_10_3.M
Last changed : 9/13/2020 8:50:01 PM by SYSTEM
Analysis Method : E:\DATA\20200905\LC 2020-09-13 18-37-17\10ETOH_25_10_3.M (Sequence Method)
Last changed : 9/14/2020 8:49:41 PM by SYSTEM
(modified after loading)
Additional Info : Peak(s) manually integrated

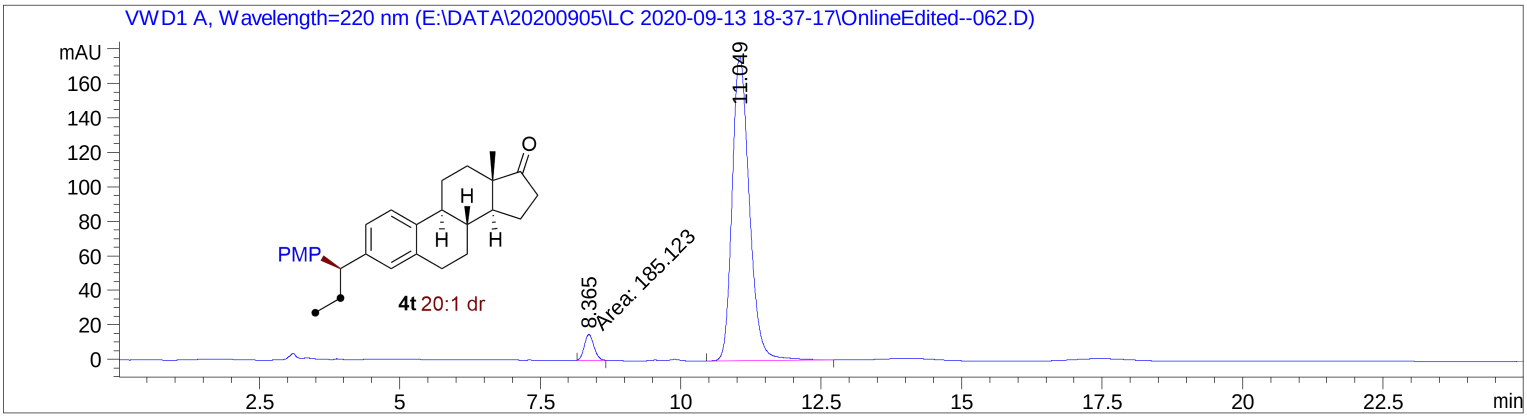

===

Area Percent Report

===

Sorted By : Signal
Multiplier : 1.0000
Dilution : 1.0000
Do not use Multiplier & Dilution Factor with ISTDs

Signal 1: VWD1 A, Wavelength=220 nm

Peak #	RetTime [min]	Type	Width [min]	Area [mAU*s]	Height [mAU]	Area %
1	8.365	FM	0.2016	185.12340	15.30253	4.6858
2	11.049	BB	0.3282	3765.57959	176.31880	95.3142

Totals : 3950.70299 191.62134

===

*** End of Report ***

Data File E:\DATA\20200905\LC 2020-09-05 14-35-09\OnlineEdited--026.D
Sample Name: YH-17-127-RAC

===

Acq. Operator : SYSTEM   Seq. Line : 26
Acq. Instrument : HPLC1260   Location : P1-A1
Injection Date : 9/6/2020 12:37:13 AM   Inj : 1
Inj Volume : 3.000 µl
Different Inj Volume from Sample Entry! Actual Inj Volume : 1.000 µl
Acq. Method : E:\DATA\20200905\LC 2020-09-05 14-35-09\1ETOH_60_5_3.M
Last changed : 9/5/2020 7:29:37 PM by SYSTEM
Analysis Method : E:\DATA\20200905\LC 2020-09-05 14-35-09\1ETOH_60_5_3.M (Sequence Method)
Last changed : 9/6/2020 12:19:17 PM by SYSTEM
(modified after loading)
Additional Info : Peak(s) manually integrated

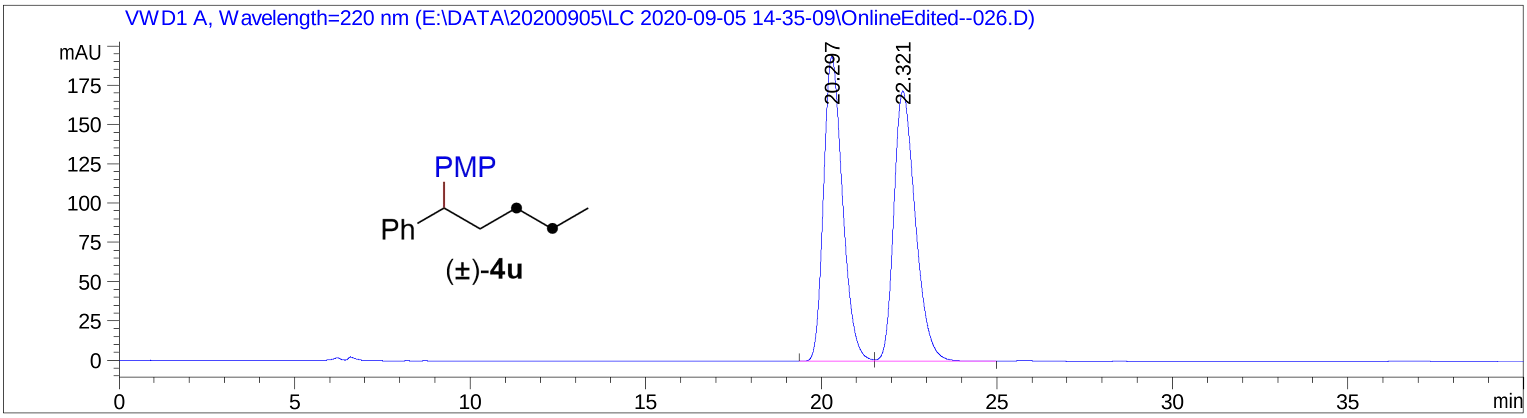

===
Area Percent Report
===

Sorted By : Signal
Multiplier : 1.0000
Dilution : 1.0000
Do not use Multiplier & Dilution Factor with ISTDs

Signal 1: VWD1 A, Wavelength=220 nm

Peak #	RetTime [min]	Type	Width [min]	Area [mAU*s]	Height [mAU]	Area %
1	20.297	BV	0.5805	7305.88867	193.95717	49.9074
2	22.321	VB	0.6543	7332.99609	172.01820	50.0926

Totals : 1.46389e4 365.97537

===
*** End of Report ***

Data File E:\DATA\20200905\LC 2020-09-05 14-35-09\OnlineEdited--027.D
Sample Name: YH-17-127-EE

===
Acq. Operator : SYSTEM Seq. Line : 27
Acq. Instrument : HPLC1260 Location : P1-A2
Injection Date : 9/6/2020 1:37:58 AM Inj : 1
Inj Volume : 3.000 µl
Different Inj Volume from Sample Entry! Actual Inj Volume : 1.000 µl
Acq. Method : E:\DATA\20200905\LC 2020-09-05 14-35-09\1ETOH_60_5_3.M
Last changed : 9/5/2020 7:29:37 PM by SYSTEM
Analysis Method : E:\DATA\20200905\LC 2020-09-05 14-35-09\1ETOH_60_5_3.M (Sequence Method)
Last changed : 9/6/2020 12:19:17 PM by SYSTEM
(modified after loading)
Additional Info : Peak(s) manually integrated

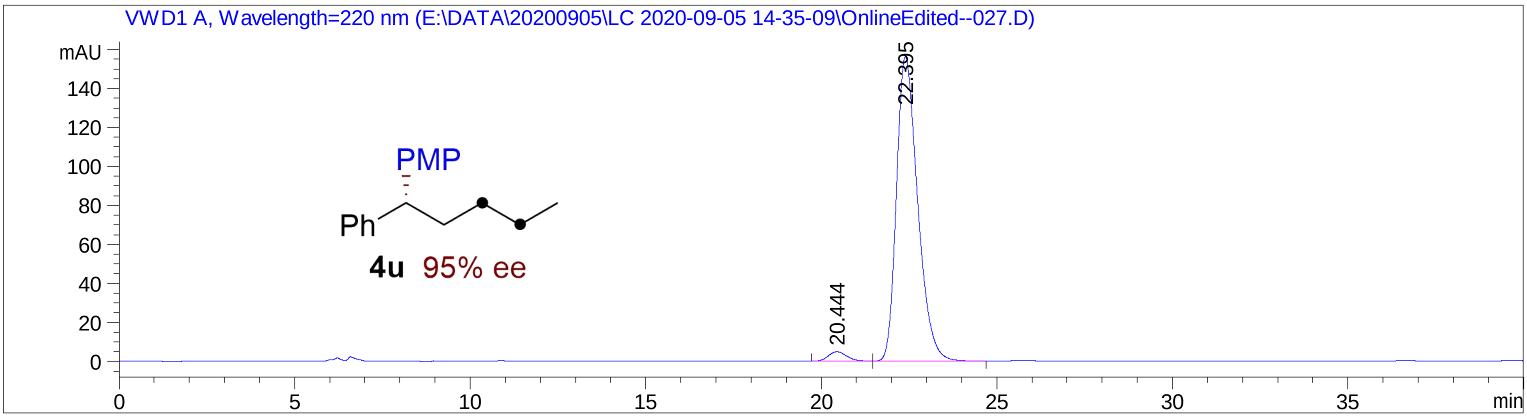

===
Area Percent Report
===

Sorted By : Signal
Multiplier : 1.0000
Dilution : 1.0000
Do not use Multiplier & Dilution Factor with ISTDs

Signal 1: VWD1 A, Wavelength=220 nm

Peak #	RetTime [min]	Type	Width [min]	Area [mAU*s]	Height [mAU]	Area %
1	20.444	BB	0.5498	174.51820	4.85016	2.5958
2	22.395	BB	0.6443	6548.65283	156.13647	97.4042

Totals : 6723.17104 160.98663

===
*** End of Report ***

===

Acq. Operator : SYSTEM   Seq. Line : 42
Acq. Instrument : HPLC1260   Location : P1-A3
Injection Date : 9/7/2020 11:47:35 AM   Inj : 1
                                          Inj Volume : 3.000 µl
Different Inj Volume from Sample Entry! Actual Inj Volume : 1.000 µl
Acq. Method : E:\DATA\20200905\LC 2020-09-06 15-18-12\1ETOH_60_5_3.M
Last changed : 9/7/2020 10:03:52 AM by SYSTEM
Analysis Method : E:\DATA\20200905\LC 2020-09-06 15-18-12\1ETOH_60_5_3.M (Sequence Method)
Last changed : 9/7/2020 1:51:58 PM by SYSTEM
(modified after loading)

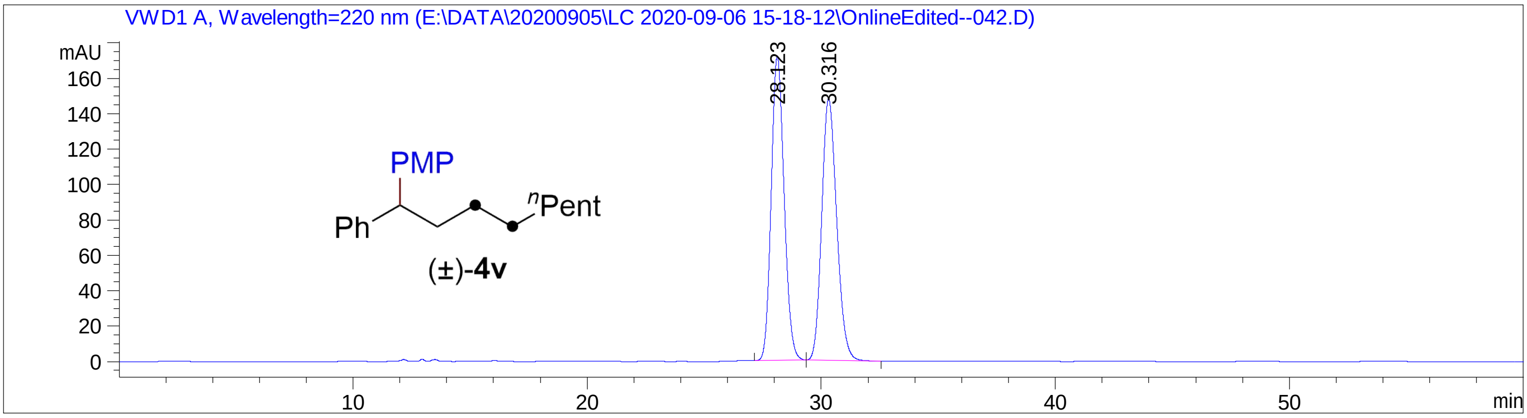

===

Area Percent Report

===

Sorted By : Signal
Multiplier : 1.0000
Dilution : 1.0000
Do not use Multiplier & Dilution Factor with ISTDs

Signal 1: VWD1 A, Wavelength=220 nm

Peak #	RetTime [min]	Type	Width [min]	Area [mAU*s]	Height [mAU]	Area %
1	28.123	BB	0.5939	6577.11133	171.68488	49.9985
2	30.316	BB	0.6894	6577.51123	147.74504	50.0015

Totals : 1.31546e4 319.42992

===

*** End of Report ***

Data File E:\DATA\20200905\LC 2020-09-06 15-18-12\OnlineEdited--043.D
Sample Name: YH-17-129-EE

===

Acq. Operator : SYSTEM Seq. Line : 43
Acq. Instrument : HPLC1260 Location : P1-A4
Injection Date : 9/7/2020 12:48:19 PM Inj : 1
Inj Volume : 3.000 µl
Different Inj Volume from Sample Entry! Actual Inj Volume : 1.000 µl
Acq. Method : E:\DATA\20200905\LC 2020-09-06 15-18-12\1ETOH_60_5_3.M
Last changed : 9/7/2020 10:03:52 AM by SYSTEM
Analysis Method : E:\DATA\20200905\LC 2020-09-06 15-18-12\1ETOH_60_5_3.M (Sequence Method)
Last changed : 9/7/2020 1:51:58 PM by SYSTEM
(modified after loading)
Additional Info : Peak(s) manually integrated

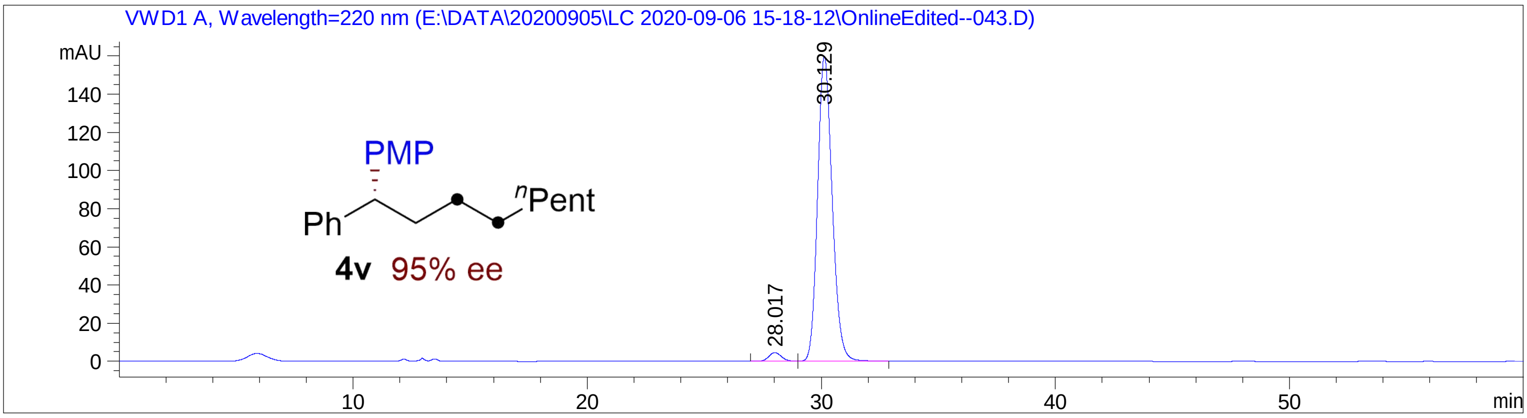

===

Area Percent Report

===

Sorted By : Signal
Multiplier : 1.0000
Dilution : 1.0000
Do not use Multiplier & Dilution Factor with ISTDs

Signal 1: VWD1 A, Wavelength=220 nm

Peak #	RetTime [min]	Type	Width [min]	Area [mAU*s]	Height [mAU]	Area %
1	28.017	BB	0.5799	171.90323	4.55951	2.4223
2	30.129	BB	0.6701	6924.69043	159.64058	97.5777

Totals : 7096.59366 164.20009

===

*** End of Report ***

Data File E:\DATA\20200905\LC 2020-09-06 15-18-12\OnlineEdited--040.D
Sample Name: YH-17-128-RAC

===

Acq. Operator : SYSTEM Seq. Line : 40
Acq. Instrument : HPLC1260 Location : P1-A1
Injection Date : 9/7/2020 9:46:04 AM Inj : 1
Inj Volume : 3.000 µl
Different Inj Volume from Sample Entry! Actual Inj Volume : 1.000 µl
Acq. Method : E:\DATA\20200905\LC 2020-09-06 15-18-12\1ETOH_60_5_3.M
Last changed : 9/7/2020 10:03:52 AM by SYSTEM
(modified after loading)
Analysis Method : E:\DATA\20200905\LC 2020-09-06 15-18-12\1ETOH_60_5_3.M (Sequence Method)
Last changed : 9/7/2020 1:51:58 PM by SYSTEM
(modified after loading)

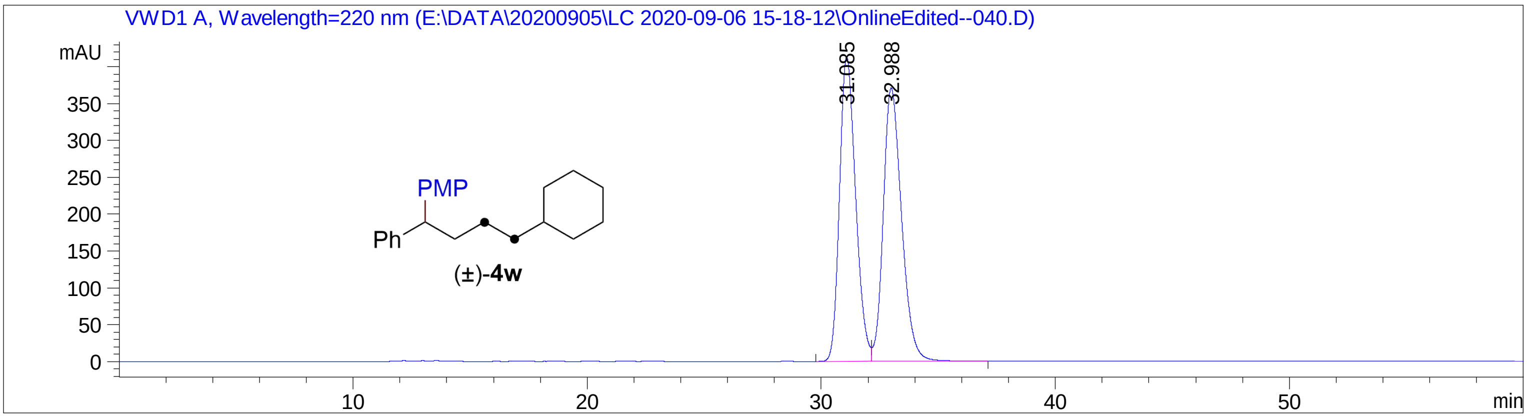

===

Area Percent Report

===

Sorted By : Signal
Multiplier : 1.0000
Dilution : 1.0000
Do not use Multiplier & Dilution Factor with ISTDs

Signal 1: VWD1 A, Wavelength=220 nm

Peak #	RetTime [min]	Type	Width [min]	Area [mAU*s]	Height [mAU]	Area %
1	31.085	BV	0.7392	1.97897e4	413.64362	49.5118
2	32.988	VB	0.8355	2.01799e4	371.04105	50.4882

Totals : 3.99696e4 784.68466

===

*** End of Report ***

Data File E:\DATA\20200905\LC 2020-09-06 15-18-12\OnlineEdited--041.D
Sample Name: YH-17-128-EE

===

Acq. Operator : SYSTEM Seq. Line : 41
Acq. Instrument : HPLC1260 Location : P1-A2
Injection Date : 9/7/2020 10:46:50 AM Inj : 1
Inj Volume : 3.000 µl
Different Inj Volume from Sample Entry! Actual Inj Volume : 1.000 µl
Acq. Method : E:\DATA\20200905\LC 2020-09-06 15-18-12\1ETOH_60_5_3.M
Last changed : 9/7/2020 10:03:52 AM by SYSTEM
Analysis Method : E:\DATA\20200905\LC 2020-09-06 15-18-12\1ETOH_60_5_3.M (Sequence Method)
Last changed : 9/7/2020 1:51:58 PM by SYSTEM
(modified after loading)
Additional Info : Peak(s) manually integrated

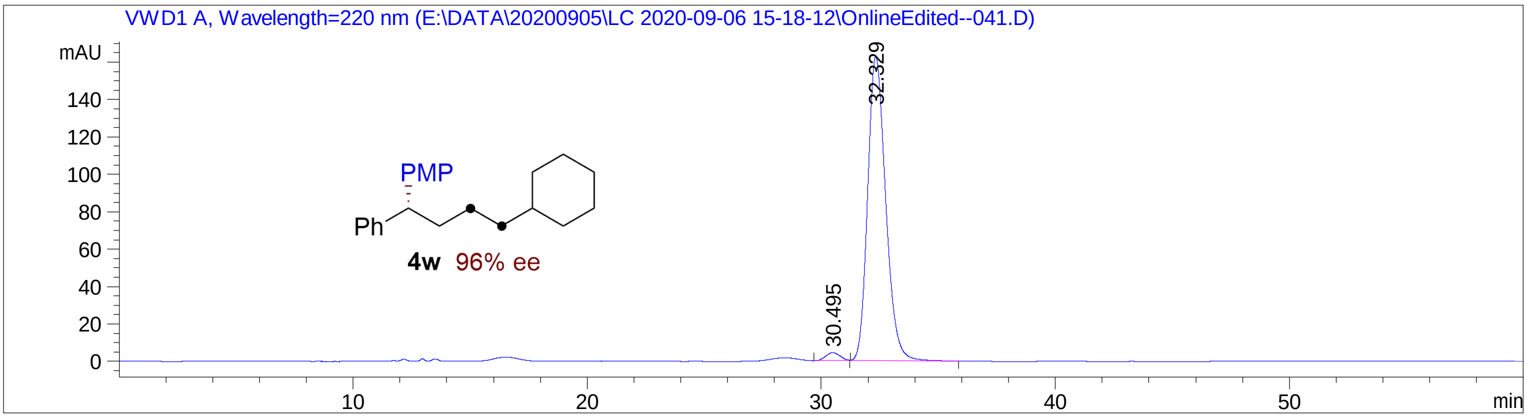

===
Area Percent Report
===

Sorted By : Signal
Multiplier : 1.0000
Dilution : 1.0000
Do not use Multiplier & Dilution Factor with ISTDs

Signal 1: VWD1 A, Wavelength=220 nm

Peak #	RetTime [min]	Type	Width [min]	Area [mAU*s]	Height [mAU]	Area %
1	30.495	BB	0.6426	172.03056	4.15022	1.9424
2	32.329	BB	0.8267	8684.74316	162.44980	98.0576

Totals : 8856.77373 166.60002

===
*** End of Report ***

===

Acq. Operator : SYSTEM Seq. Line : 69
Acq. Instrument : HPLC1260 Location : P1-B4
Injection Date : 9/12/2020 8:23:31 PM Inj : 1
Inj Volume : 3.000 µl
Different Inj Volume from Sample Entry! Actual Inj Volume : 1.000 µl
Acq. Method : E:\DATA\20200905\LC 2020-09-11 08-36-22\5IPA_40_8_2.M
Last changed : 9/11/2020 3:58:07 PM by SYSTEM
Analysis Method : E:\DATA\20200905\LC 2020-09-11 08-36-22\5IPA_40_8_2.M (Sequence Method)
Last changed : 9/12/2020 9:54:34 PM by SYSTEM
(modified after loading)
Additional Info : Peak(s) manually integrated

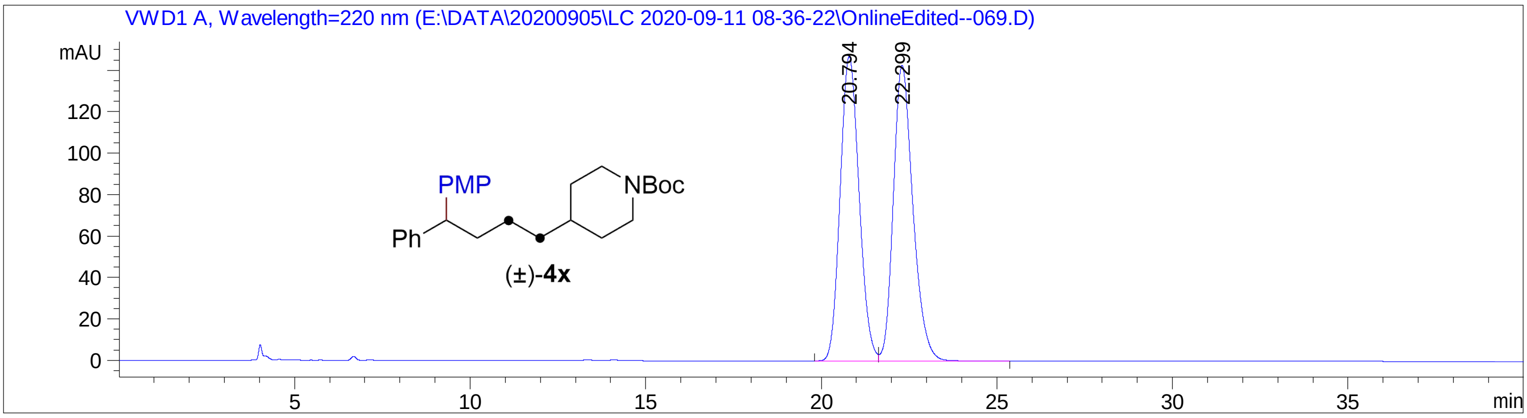

===

Area Percent Report

===

Sorted By : Signal
Multiplier : 1.0000
Dilution : 1.0000
Do not use Multiplier & Dilution Factor with ISTDs

Signal 1: VWD1 A, Wavelength=220 nm

Peak #	RetTime [min]	Type	Width [min]	Area [mAU*s]	Height [mAU]	Area %
1	20.794	BV	0.5852	5505.28906	146.92302	49.8768
2	22.299	VB	0.5918	5532.49512	142.85559	50.1232

Totals : 1.10378e4 289.77861

===

*** End of Report ***

Data File E:\DATA\20200905\LC 2020-09-11 08-36-22\OnlineEdited--068.D
Sample Name: YH-17-148-RAC

===

Acq. Operator : SYSTEM   Seq. Line : 68
Acq. Instrument : HPLC1260   Location : P1-B3
Injection Date : 9/12/2020 7:42:47 PM   Inj : 1
Inj Volume : 3.000 µl
Different Inj Volume from Sample Entry! Actual Inj Volume : 1.000 µl
Acq. Method : E:\DATA\20200905\LC 2020-09-11 08-36-22\5IPA_40_8_2.M
Last changed : 9/11/2020 3:58:07 PM by SYSTEM
Analysis Method : E:\DATA\20200905\LC 2020-09-11 08-36-22\5IPA_40_8_2.M (Sequence Method)
Last changed : 9/12/2020 9:54:34 PM by SYSTEM
(modified after loading)
Additional Info : Peak(s) manually integrated

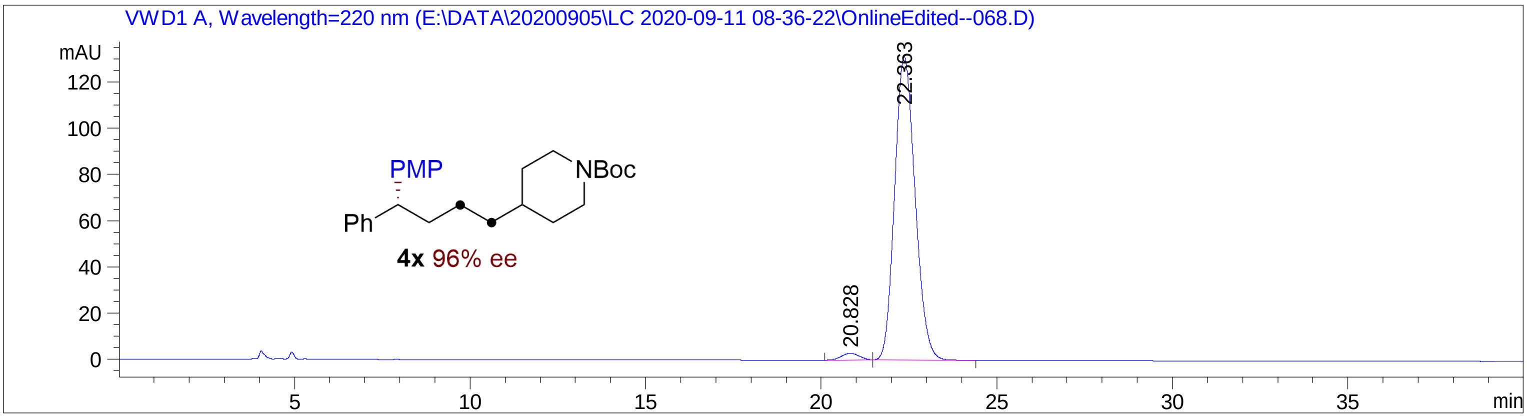

===
Area Percent Report
===

Sorted By : Signal
Multiplier : 1.0000
Dilution : 1.0000
Do not use Multiplier & Dilution Factor with ISTDs

Signal 1: VWD1 A, Wavelength=220 nm

Peak #	RetTime [min]	Type	Width [min]	Area [mAU*s]	Height [mAU]	Area %
1	20.828	BB	0.5403	100.21465	2.86358	1.8463
2	22.363	BB	0.6315	5327.57861	131.53824	98.1537

Totals : 5427.79326 134.40182

===
*** End of Report ***

Data File E:\DATA\20200905\LC 2020-09-11 08-36-22\OnlineEdited--034.D
Sample Name: YH-17-147-RAC

===

Acq. Operator : SYSTEM   Seq. Line : 34
Acq. Instrument : HPLC1260   Location : P1-B1
Injection Date : 9/11/2020 11:12:14 PM   Inj : 5
Inj Volume : 3.000 µl
Different Inj Volume from Sample Entry! Actual Inj Volume : 1.000 µl
Acq. Method : E:\DATA\20200905\LC 2020-09-11 08-36-22\1IPA_40_5_1.M
Last changed : 9/11/2020 11:12:05 PM by SYSTEM
(modified after loading)
Analysis Method : E:\DATA\20200905\LC 2020-09-11 08-36-22\1IPA_40_5_1.M (Sequence Method)
Last changed : 9/12/2020 9:39:00 AM by SYSTEM
(modified after loading)
Additional Info : Peak(s) manually integrated

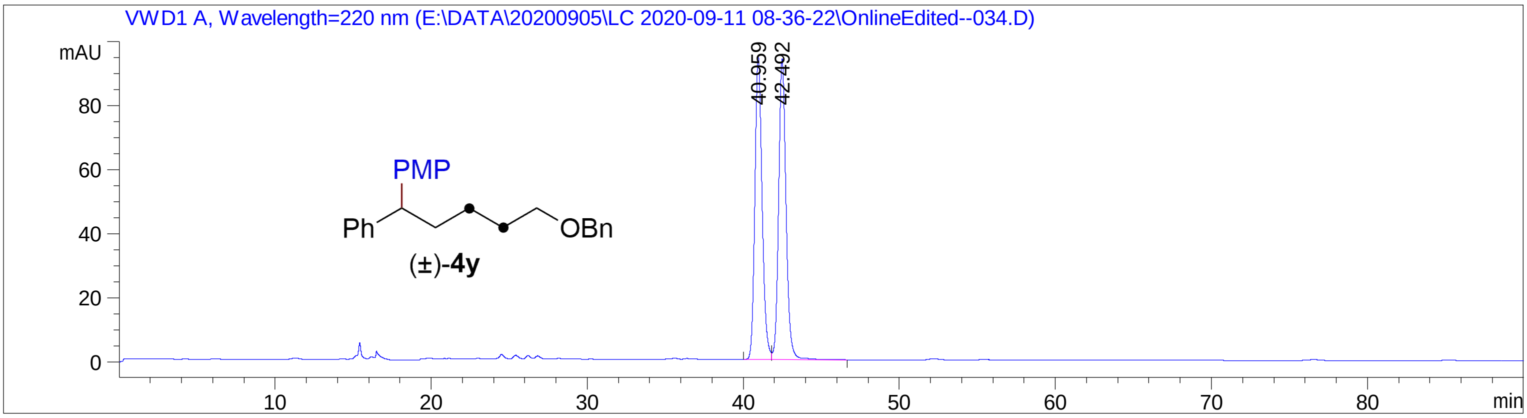

===

Area Percent Report

===

Sorted By : Signal
Multiplier : 1.0000
Dilution : 1.0000
Do not use Multiplier & Dilution Factor with ISTDs

Signal 1: VWD1 A, Wavelength=220 nm

Peak #	RetTime [min]	Type	Width [min]	Area [mAU*s]	Height [mAU]	Area %
1	40.959	BV	0.4954	3048.32300	94.47705	49.6760
2	42.492	VB	0.5020	3088.08325	93.81505	50.3240

Totals : 6136.40625 188.29210

===

*** End of Report ***

Data File E:\DATA\20200905\LC 2020-09-11 08-36-22\OnlineEdited--035.D
Sample Name: YH-17-147-EE

===

Acq. Operator : SYSTEM   Seq. Line : 35
Acq. Instrument : HPLC1260   Location : P1-B2
Injection Date : 9/12/2020 12:43:00 AM   Inj : 1
Inj Volume : 3.000 µl
Different Inj Volume from Sample Entry! Actual Inj Volume : 2.000 µl
Acq. Method : E:\DATA\20200905\LC 2020-09-11 08-36-22\1IPA_40_5_1.M
Last changed : 9/11/2020 11:12:05 PM by SYSTEM
Analysis Method : E:\DATA\20200905\LC 2020-09-11 08-36-22\1IPA_40_5_1.M (Sequence Method)
Last changed : 9/12/2020 9:39:00 AM by SYSTEM
(modified after loading)
Additional Info : Peak(s) manually integrated

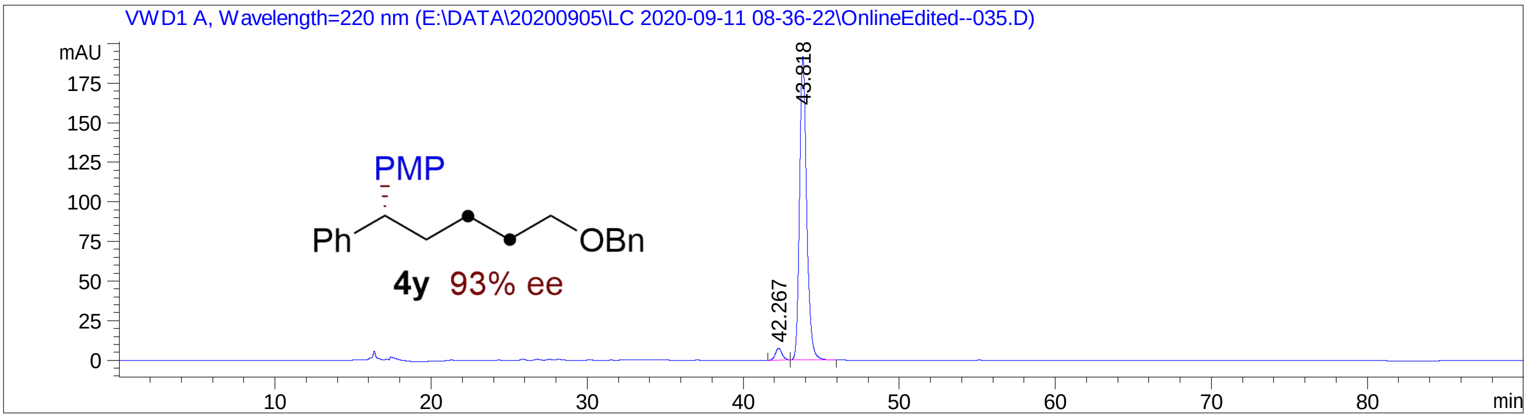

===

Area Percent Report

===

Sorted By : Signal
Multiplier : 1.0000
Dilution : 1.0000
Do not use Multiplier & Dilution Factor with ISTDs

Signal 1: VWD1 A, Wavelength=220 nm

Peak #	RetTime [min]	Type	Width [min]	Area [mAU*s]	Height [mAU]	Area %
1	42.267	BB	0.4521	217.21002	7.42821	3.4565
2	43.818	BB	0.4847	6066.84277	191.43550	96.5435

Totals : 6284.05280 198.86371

===

*** End of Report ***

Data File E:\DATA\20200905\LC 2020-09-11 08-36-22\OnlineEdited--062.D
Sample Name: YH-17-150-RAC

===

Acq. Operator : SYSTEM   Seq. Line : 62
Acq. Instrument : HPLC1260   Location : P1-B5
Injection Date : 9/12/2020 5:03:14 PM   Inj : 1
Inj Volume : 3.000 µl
Different Inj Volume from Sample Entry! Actual Inj Volume : 1.000 µl
Acq. Method : E:\DATA\20200905\LC 2020-09-11 08-36-22\10IPA_20_8_4.M
Last changed : 9/11/2020 8:14:55 PM by SYSTEM
Analysis Method : E:\DATA\20200905\LC 2020-09-11 08-36-22\10IPA_20_8_4.M (Sequence Method)
Last changed : 9/12/2020 9:52:06 PM by SYSTEM
(modified after loading)
Additional Info : Peak(s) manually integrated

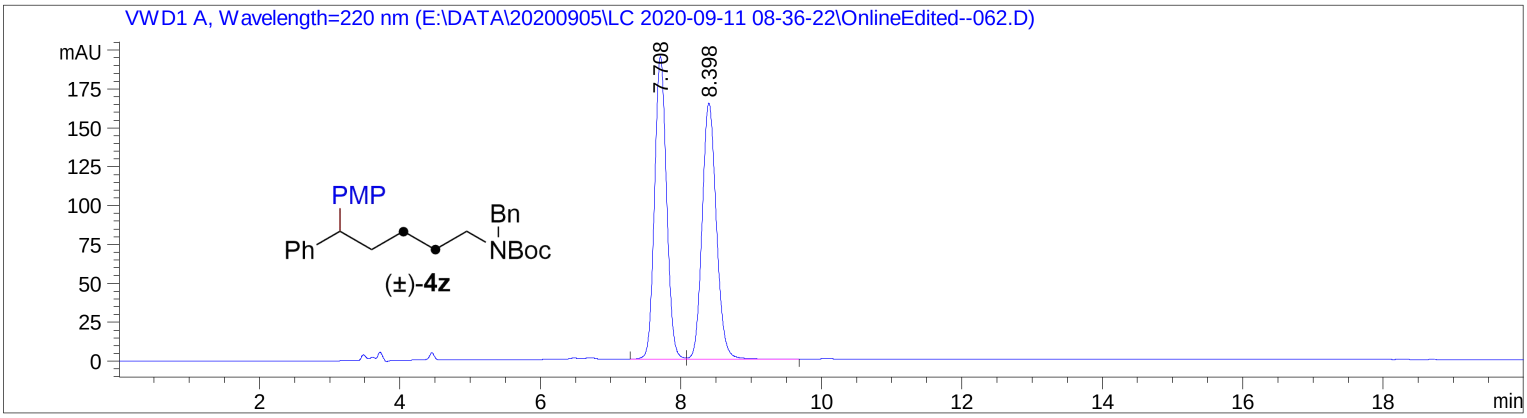

===

Area Percent Report

===

Sorted By : Signal
Multiplier : 1.0000
Dilution : 1.0000
Do not use Multiplier & Dilution Factor with ISTDs

Signal 1: VWD1 A, Wavelength=220 nm

Peak #	RetTime [min]	Type	Width [min]	Area [mAU*s]	Height [mAU]	Area %
1	7.708	BV	0.1850	2317.88989	194.59912	50.0108
2	8.398	VB	0.2180	2316.88647	164.78603	49.9892

Totals : 4634.77637 359.38515

===

*** End of Report ***

Data File E:\DATA\20200905\LC 2020-09-11 08-36-22\OnlineEdited--063.D
Sample Name: YH-17-150-EE

===

Acq. Operator : SYSTEM Seq. Line : 63
Acq. Instrument : HPLC1260 Location : P1-B6
Injection Date : 9/12/2020 5:24:00 PM Inj : 1
Inj Volume : 3.000 µl
Different Inj Volume from Sample Entry! Actual Inj Volume : 2.000 µl
Acq. Method : E:\DATA\20200905\LC 2020-09-11 08-36-22\10IPA_20_8_4.M
Last changed : 9/11/2020 8:14:55 PM by SYSTEM
Analysis Method : E:\DATA\20200905\LC 2020-09-11 08-36-22\10IPA_20_8_4.M (Sequence Method)
Last changed : 9/12/2020 9:52:06 PM by SYSTEM
(modified after loading)
Additional Info : Peak(s) manually integrated

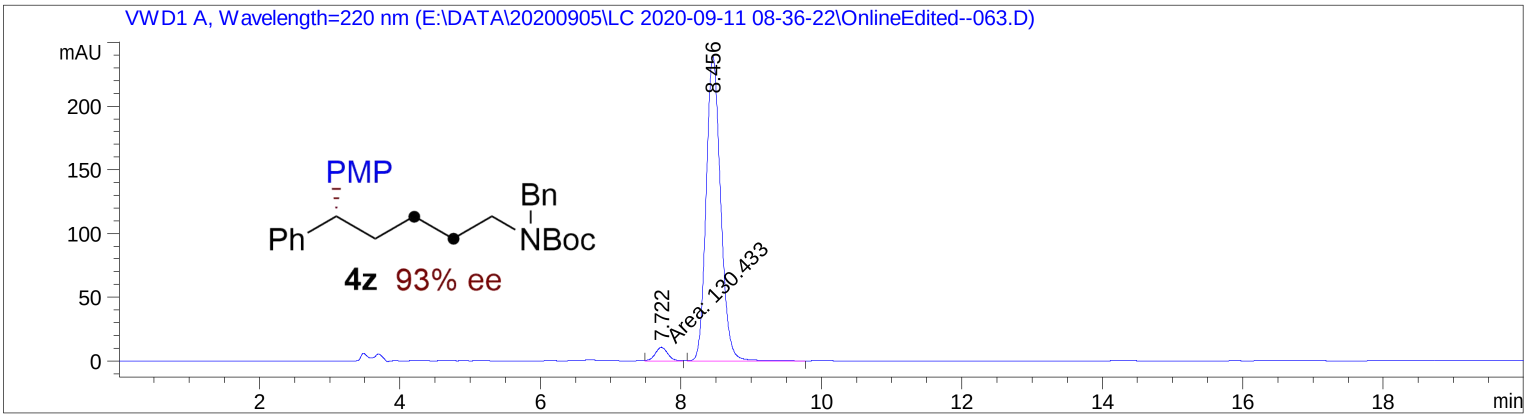

===

Area Percent Report

===

Sorted By : Signal
Multiplier : 1.0000
Dilution : 1.0000
Do not use Multiplier & Dilution Factor with ISTDs

Signal 1: VWD1 A, Wavelength=220 nm

Peak #	RetTime [min]	Type	Width [min]	Area [mAU*s]	Height [mAU]	Area %
1	7.722	FM	0.2043	130.43301	10.64253	3.6710
2	8.456	VB	0.2212	3422.60986	238.79257	96.3290

Totals : 3553.04288 249.43510

===

*** End of Report ***

Data File E:\DATA\20200905\LC 2020-09-13 18-37-17\OnlineEdited--111.D
Sample Name: YH-17-170-RAC

===

Acq. Operator : SYSTEM Seq. Line : 111
Acq. Instrument : HPLC1260 Location : P1-C3
Injection Date : 9/16/2020 2:59:49 AM Inj : 1
Inj Volume : 3.000 µl
Different Inj Volume from Sample Entry! Actual Inj Volume : 0.100 µl
Acq. Method : E:\DATA\20200905\LC 2020-09-13 18-37-17\1EtOH_50_5_1.M
Last changed : 9/15/2020 3:46:59 PM by SYSTEM
Analysis Method : E:\DATA\20200905\LC 2020-09-13 18-37-17\1EtOH_50_5_1.M (Sequence Method)
Last changed : 9/16/2020 9:20:20 AM by SYSTEM
(modified after loading)
Additional Info : Peak(s) manually integrated

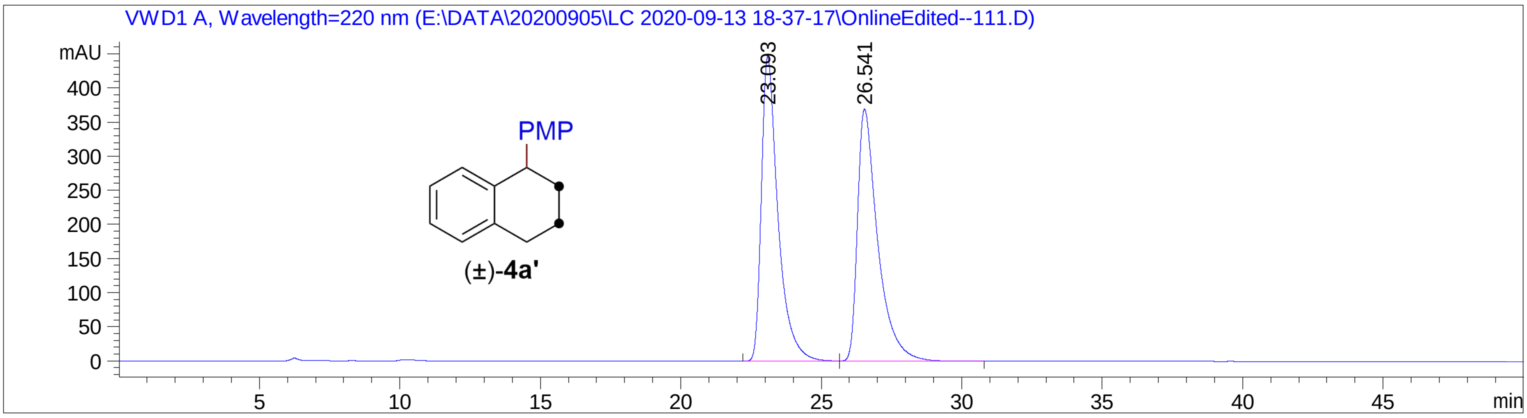

===

Area Percent Report

===

Sorted By : Signal
Multiplier : 1.0000
Dilution : 1.0000
Do not use Multiplier & Dilution Factor with ISTDs

Signal 1: VWD1 A, Wavelength=220 nm

Peak #	RetTime [min]	Type	Width [min]	Area [mAU*s]	Height [mAU]	Area %
1	23.093	BB	0.6134	1.83690e4	446.12729	49.8547
2	26.541	BB	0.7399	1.84760e4	369.18671	50.1453

Totals : 3.68450e4 815.31400

===

*** End of Report ***

Data File E:\DATA\20200905\LC 2020-09-13 18-37-17\OnlineEdited--112.D
Sample Name: YH-17-170-EE

===

Acq. Operator : SYSTEM Seq. Line : 112
Acq. Instrument : HPLC1260 Location : P1-C4
Injection Date : 9/16/2020 3:50:38 AM Inj : 1
Inj Volume : 3.000 µl
Different Inj Volume from Sample Entry! Actual Inj Volume : 1.000 µl
Acq. Method : E:\DATA\20200905\LC 2020-09-13 18-37-17\1EtOH_50_5_1.M
Last changed : 9/15/2020 3:46:59 PM by SYSTEM
Analysis Method : E:\DATA\20200905\LC 2020-09-13 18-37-17\1EtOH_50_5_1.M (Sequence Method)
Last changed : 9/16/2020 9:20:20 AM by SYSTEM
(modified after loading)
Additional Info : Peak(s) manually integrated

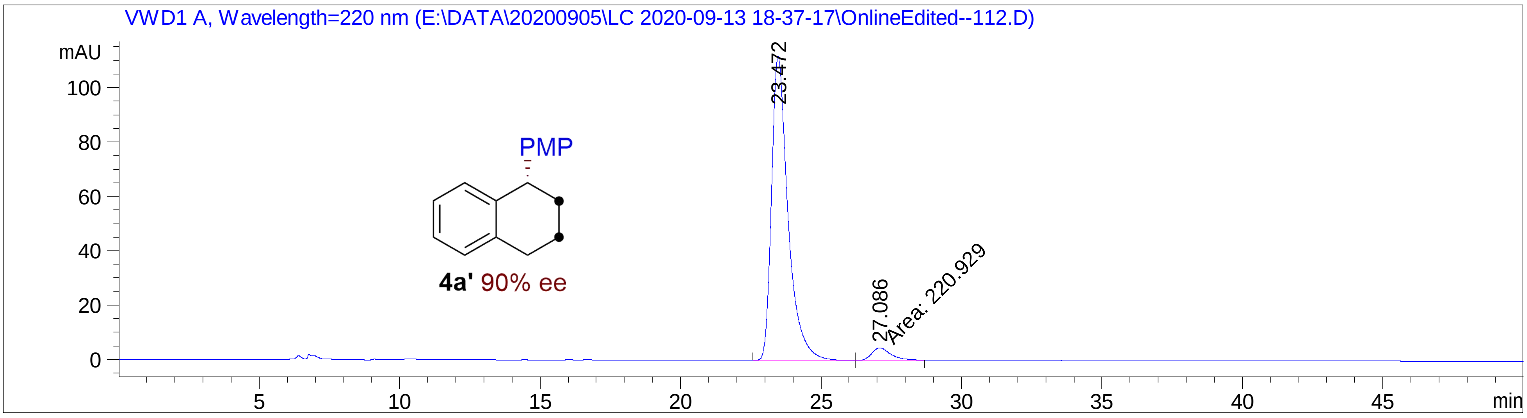

===

Area Percent Report

===

Sorted By : Signal
Multiplier : 1.0000
Dilution : 1.0000
Do not use Multiplier & Dilution Factor with ISTDs

Signal 1: VWD1 A, Wavelength=220 nm

Peak #	RetTime [min]	Type	Width [min]	Area [mAU*s]	Height [mAU]	Area %
1	23.472	BB	0.6120	4572.08252	111.81881	95.3906
2	27.086	MF	0.8030	220.92908	4.58552	4.6094

Totals : 4793.01160 116.40432

===

*** End of Report ***

Data File E:\DATA\20201027\LC 2020-11-02 19-12-04\OnlineEdited--005.D
Sample Name: YH-18-81-RAC

===

Acq. Operator : SYSTEM Seq. Line : 5
Acq. Instrument : HPLC1260 Location : P1-A1
Injection Date : 11/2/2020 9:17:03 PM Inj : 1
Inj Volume : 3.000 µl
Different Inj Volume from Sample Entry! Actual Inj Volume : 2.000 µl
Acq. Method : E:\DATA\20201027\LC 2020-11-02 19-12-04\5IPA_60_8_3.M
Last changed : 11/2/2020 8:34:42 PM by SYSTEM
Analysis Method : E:\DATA\20201027\LC 2020-11-02 19-12-04\5IPA_60_8_3.M (Sequence Method)
Last changed : 11/3/2020 8:16:37 AM by SYSTEM
(modified after loading)
Additional Info : Peak(s) manually integrated

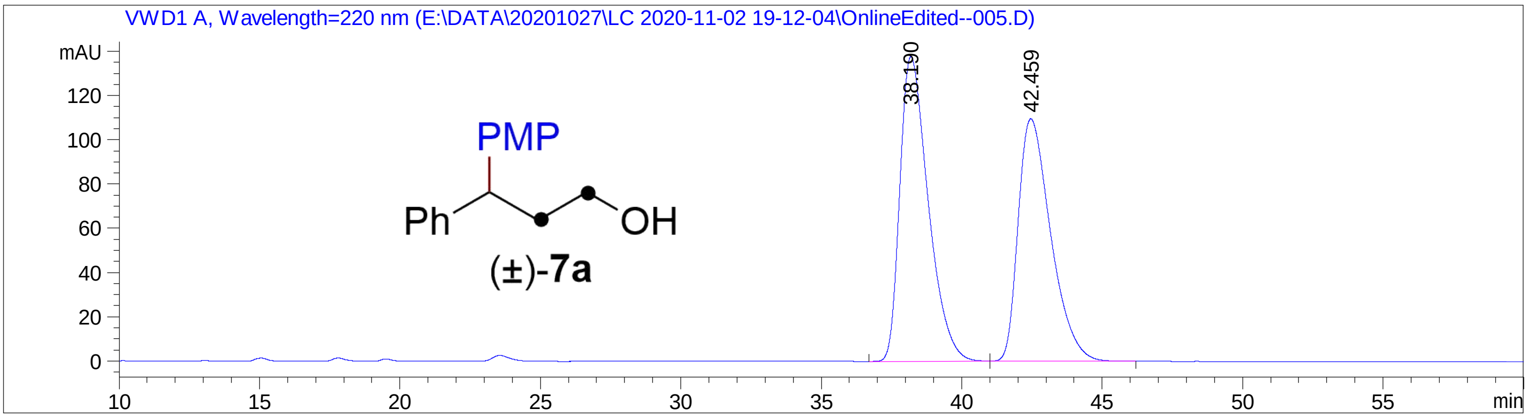

===

Area Percent Report

===

Sorted By : Signal
Multiplier : 1.0000
Dilution : 1.0000
Do not use Multiplier & Dilution Factor with ISTDs

Signal 1: VWD1 A, Wavelength=220 nm

Peak #	RetTime [min]	Type	Width [min]	Area [mAU*s]	Height [mAU]	Area %
1	38.190	BB	1.0737	9665.29883	137.73904	52.2905
2	42.459	BB	1.2331	8818.56152	109.55659	47.7095

Totals : 1.84839e4 247.29563

===

*** End of Report ***

Data File E:\DATA\20201027\LC 2020-11-02 19-12-04\OnlineEdited--006.D
Sample Name: YH-18-81-EE

===

Acq. Operator : SYSTEM   Seq. Line : 6
Acq. Instrument : HPLC1260   Location : P1-A2
Injection Date : 11/2/2020 10:17:51 PM   Inj : 1
Inj Volume : 3.000 µl
Acq. Method : E:\DATA\20201027\LC 2020-11-02 19-12-04\5IPA_60_8_3.M
Last changed : 11/2/2020 8:34:42 PM by SYSTEM
Analysis Method : E:\DATA\20201027\LC 2020-11-02 19-12-04\5IPA_60_8_3.M (Sequence Method)
Last changed : 11/3/2020 8:16:37 AM by SYSTEM
(modified after loading)
Additional Info : Peak(s) manually integrated

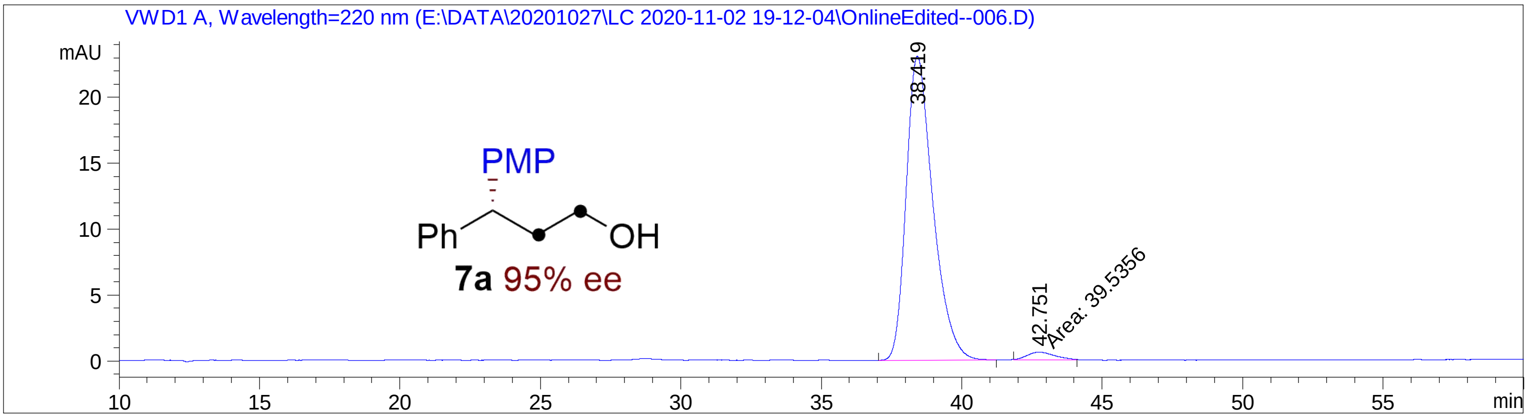

===

Area Percent Report

===

Sorted By : Signal
Multiplier : 1.0000
Dilution : 1.0000
Do not use Multiplier & Dilution Factor with ISTDs

Signal 1: VWD1 A, Wavelength=220 nm

Peak #	RetTime [min]	Type	Width [min]	Area [mAU*s]	Height [mAU]	Area %
1	38.419	BB	0.9930	1529.97595	23.06288	97.4810
2	42.751	MF	1.1403	39.53560	5.77867e-1	2.5190

Totals : 1569.51155 23.64074

===

*** End of Report ***

Data File E:\DATA\20201027\LC 2020-11-08 10-17-40\OnlineEdited--090.D
Sample Name: YH-18-92-RAC-AD

===
Acq. Operator : SYSTEM Seq. Line : 90
Acq. Instrument : HPLC1260 Location : P1-D3
Injection Date : 11/10/2020 5:13:10 AM Inj : 1
Inj Volume : 3.000 µl
Different Inj Volume from Sample Entry! Actual Inj Volume : 2.000 µl
Acq. Method : E:\DATA\20201027\LC 2020-11-08 10-17-40\5IPA_60_10_3.M
Last changed : 11/9/2020 3:42:20 PM by SYSTEM
Analysis Method : E:\DATA\20201027\LC 2020-11-08 10-17-40\5IPA_60_10_3.M (Sequence Method)
Last changed : 11/10/2020 9:02:46 AM by SYSTEM
(modified after loading)

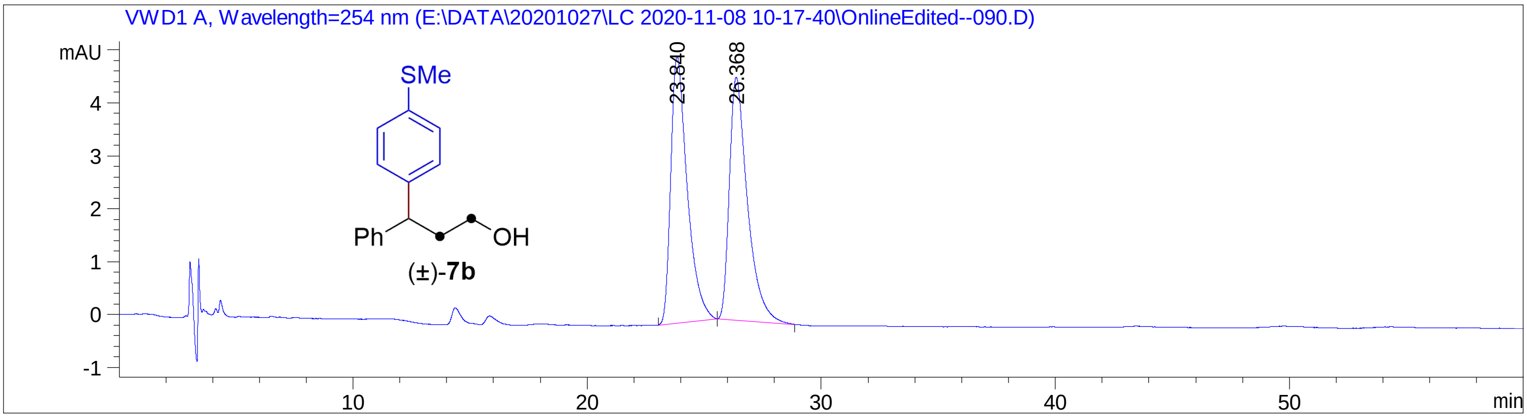

===
Area Percent Report
===

Sorted By : Signal
Multiplier : 1.0000
Dilution : 1.0000
Do not use Multiplier & Dilution Factor with ISTDs

Signal 1: VWD1 A, Wavelength=254 nm

Peak #	RetTime [min]	Type	Width [min]	Area [mAU*s]	Height [mAU]	Area %
1	23.840	BB	0.6979	246.77345	5.04253	50.0282
2	26.368	BB	0.7664	246.49480	4.59865	49.9718

Totals : 493.26825 9.64117

===
*** End of Report ***

Data File E:\DATA\20201027\LC 2020-11-08 10-17-40\OnlineEdited--091.D
Sample Name: YH-18-92-EE

===

Acq. Operator : SYSTEM Seq. Line : 91
Acq. Instrument : HPLC1260 Location : P1-D4
Injection Date : 11/10/2020 6:14:00 AM Inj : 1
Inj Volume : 3.000 µl
Different Inj Volume from Sample Entry! Actual Inj Volume : 2.000 µl
Acq. Method : E:\DATA\20201027\LC 2020-11-08 10-17-40\5IPA_60_10_3.M
Last changed : 11/9/2020 3:42:20 PM by SYSTEM
Analysis Method : E:\DATA\20201027\LC 2020-11-08 10-17-40\5IPA_60_10_3.M (Sequence Method)
Last changed : 11/10/2020 9:02:46 AM by SYSTEM
(modified after loading)
Additional Info : Peak(s) manually integrated

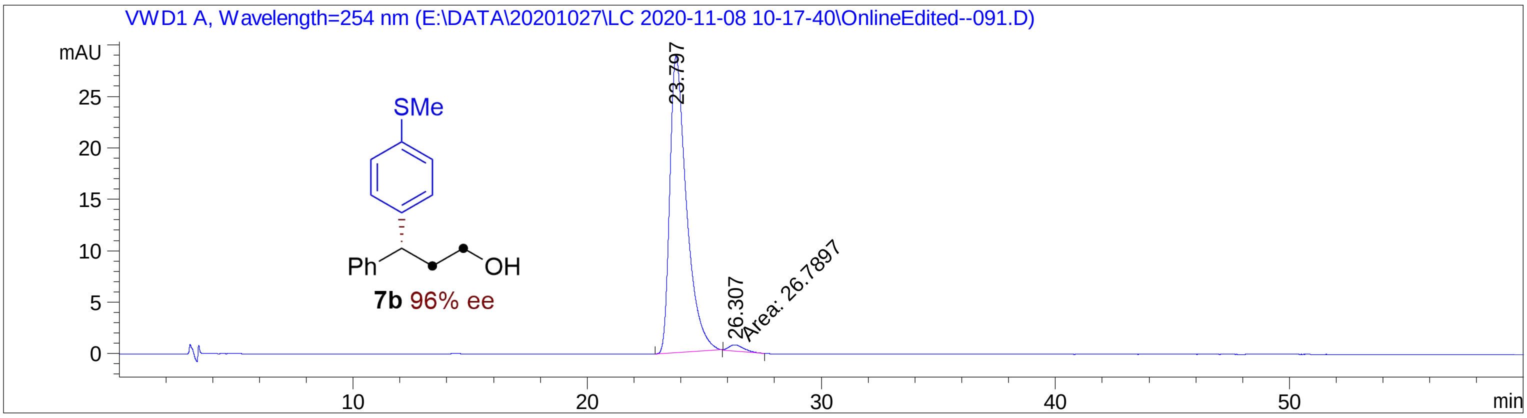

===
Area Percent Report
===

Sorted By : Signal
Multiplier : 1.0000
Dilution : 1.0000
Do not use Multiplier & Dilution Factor with ISTDs

Signal 1: VWD1 A, Wavelength=254 nm

Peak #	RetTime [min]	Type	Width [min]	Area [mAU*s]	Height [mAU]	Area %
1	23.797	BB	0.7363	1428.68213	28.77318	98.1594
2	26.307	MM	0.7397	26.78973	6.03619e-1	1.8406

Totals : 1455.47186 29.37680

===
*** End of Report ***

Data File E:\DATA\20201027\LC 2020-11-08 10-17-40\OnlineEdited--084.D
Sample Name: YH-18-90-RAC

===

Acq. Operator : SYSTEM   Seq. Line : 84
Acq. Instrument : HPLC1260   Location : P1-D1
Injection Date : 11/10/2020 12:57:55 AM   Inj : 1
                                    Inj Volume : 3.000 µl
Different Inj Volume from Sample Entry! Actual Inj Volume : 2.000 µl
Acq. Method : E:\DATA\20201027\LC 2020-11-08 10-17-40\25IPA_40_10_4.M
Last changed : 11/9/2020 3:38:40 PM by SYSTEM
Analysis Method : E:\DATA\20201027\LC 2020-11-08 10-17-40\25IPA_40_10_4.M (Sequence Method)
Last changed : 11/10/2020 9:00:06 AM by SYSTEM
(modified after loading)
Additional Info : Peak(s) manually integrated

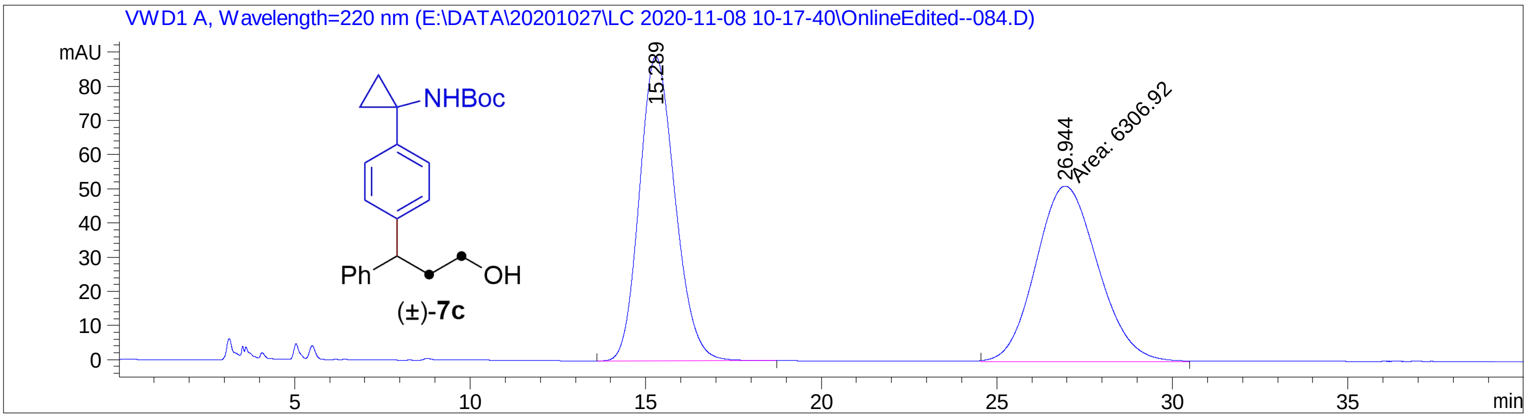

===

Area Percent Report

===

Sorted By : Signal
Multiplier : 1.0000
Dilution : 1.0000
Do not use Multiplier & Dilution Factor with ISTDs

Signal 1: VWD1 A, Wavelength=220 nm

Peak #	RetTime [min]	Type	Width [min]	Area [mAU*s]	Height [mAU]	Area %
1	15.289	BB	1.0925	6273.77100	88.99331	49.8683
2	26.944	MM	2.0482	6306.91895	51.32027	50.1317

Totals : 1.25807e4 140.31358

===

*** End of Report ***

Data File E:\DATA\20201027\LC 2020-11-08 10-17-40\OnlineEdited--085.D
Sample Name: YH-18-90-EE

===

Acq. Operator : SYSTEM   Seq. Line : 85
Acq. Instrument : HPLC1260   Location : P1-D2
Injection Date : 11/10/2020 1:38:46 AM   Inj : 1
                                          Inj Volume : 3.000 µl
Different Inj Volume from Sample Entry! Actual Inj Volume : 2.000 µl
Acq. Method : E:\DATA\20201027\LC 2020-11-08 10-17-40\25IPA_40_10_4.M
Last changed : 11/9/2020 3:38:40 PM by SYSTEM
Analysis Method : E:\DATA\20201027\LC 2020-11-08 10-17-40\25IPA_40_10_4.M (Sequence Method)
Last changed : 11/10/2020 9:00:06 AM by SYSTEM
(modified after loading)
Additional Info : Peak(s) manually integrated

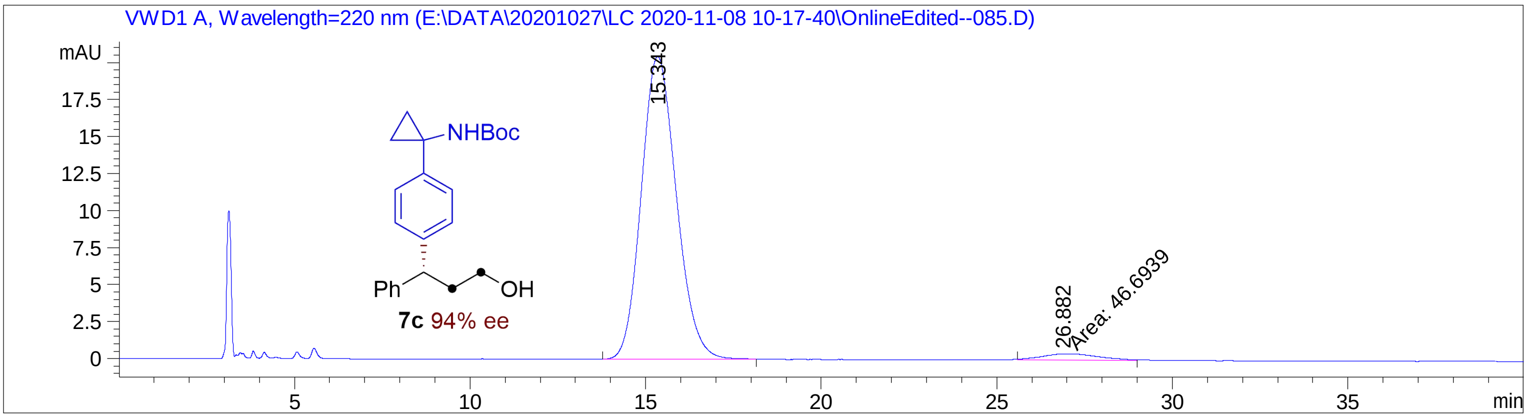

===
Area Percent Report
===

Sorted By : Signal
Multiplier : 1.0000
Dilution : 1.0000
Do not use Multiplier & Dilution Factor with ISTDs

Signal 1: VWD1 A, Wavelength=220 nm

Peak #	RetTime [min]	Type	Width [min]	Area [mAU*s]	Height [mAU]	Area %
1	15.343	BB	1.0508	1418.77441	20.43937	96.8137
2	26.882	FM	1.8297	46.69392	4.25322e-1	3.1863

Totals : 1465.46833 20.86470

===
*** End of Report ***

Data File E:\DATA\20201027\LC 2020-11-05 12-38-40\OnlineEdited--007.D
Sample Name: YH-18-83-RAC

===

Acq. Operator : SYSTEM Seq. Line : 7
Acq. Instrument : HPLC1260 Location : P1-B1
Injection Date : 11/5/2020 3:21:53 PM Inj : 1
Inj Volume : 3.000 µl
Different Inj Volume from Sample Entry! Actual Inj Volume : 1.000 µl
Acq. Method : E:\DATA\20201027\LC 2020-11-05 12-38-40\10IPA_30_10_1.M
Last changed : 11/5/2020 1:49:04 PM by SYSTEM
Analysis Method : E:\DATA\20201027\LC 2020-11-05 12-38-40\10IPA_30_10_1.M (Sequence Method)
Last changed : 11/5/2020 2:19:41 PM by SYSTEM
Additional Info : Peak(s) manually integrated

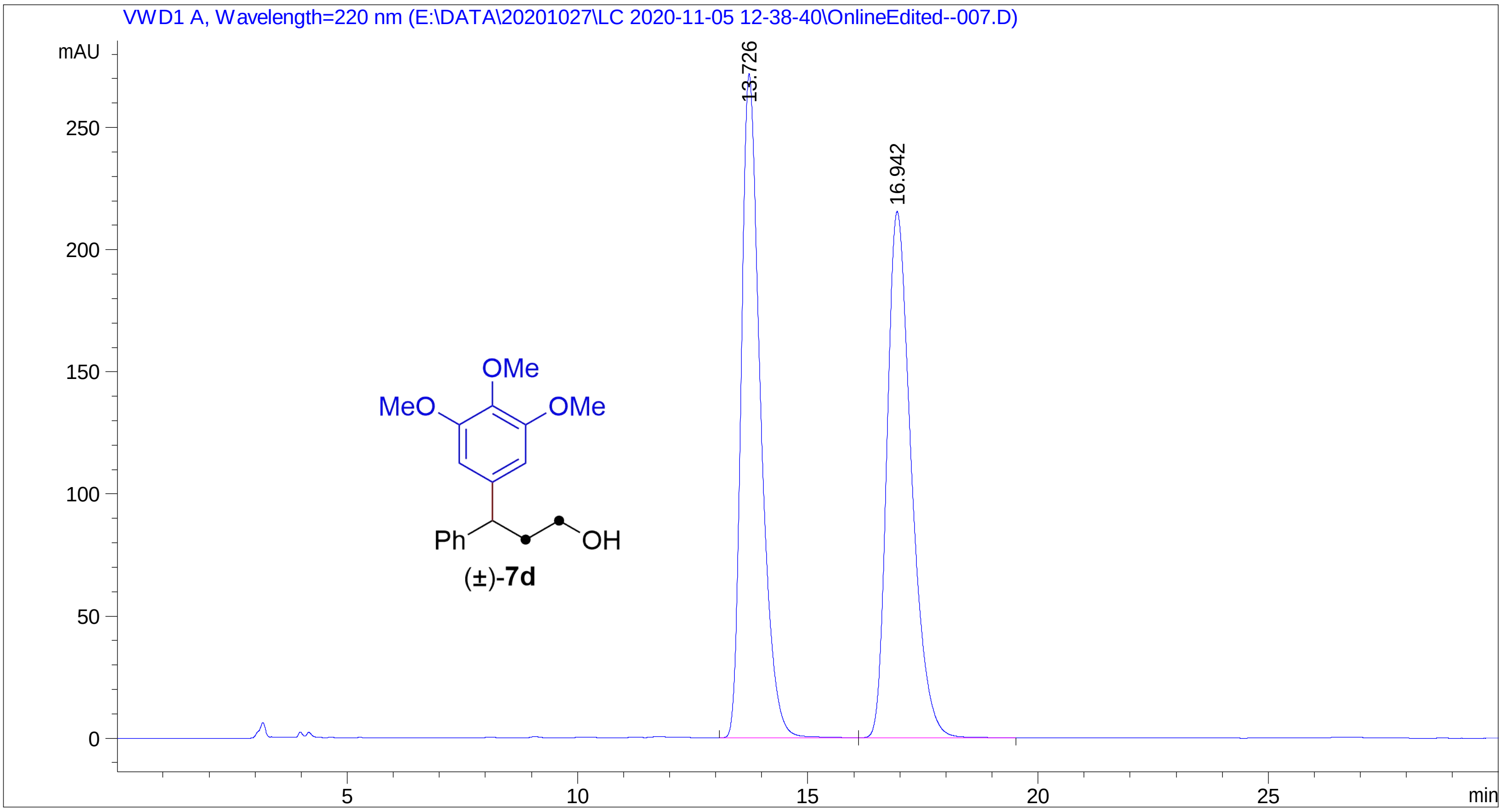

===
Area Percent Report
===

Sorted By : Signal
Multiplier : 1.0000
Dilution : 1.0000
Use Multiplier & Dilution Factor with ISTDs

Signal 1: VWD1 A, Wavelength=220 nm

Peak #	RetTime [min]	Type	Width [min]	Area [mAU*s]	Height [mAU]	Area %
1	13.726	BB	0.4386	7893.44824	271.93649	49.9928
2	16.942	BB	0.5595	7895.72559	215.45660	50.0072

Totals : 1.57892e4 487.39310

===

Data File E:\DATA\20201027\LC 2020-11-05 12-38-40\OnlineEdited--008.D
Sample Name: YH-18-83-EE

===

Acq. Operator	: SYSTEM	Seq. Line :	8
Acq. Instrument	: HPLC1260	Location :	P1-B2
Injection Date	: 11/5/2020 3:52:38 PM	Inj :	1
		Inj Volume :	3.000 µl

Different Inj Volume from Sample Entry! Actual Inj Volume : 2.000 µl

Acq. Method : E:\DATA\20201027\LC 2020-11-05 12-38-40\10IPA_30_10_1.M
Last changed : 11/5/2020 1:49:04 PM by SYSTEM
Analysis Method : E:\DATA\20201027\LC 2020-11-05 12-38-40\10IPA_30_10_1.M (Sequence Method)
Last changed : 11/5/2020 2:19:41 PM by SYSTEM
Additional Info : Peak(s) manually integrated

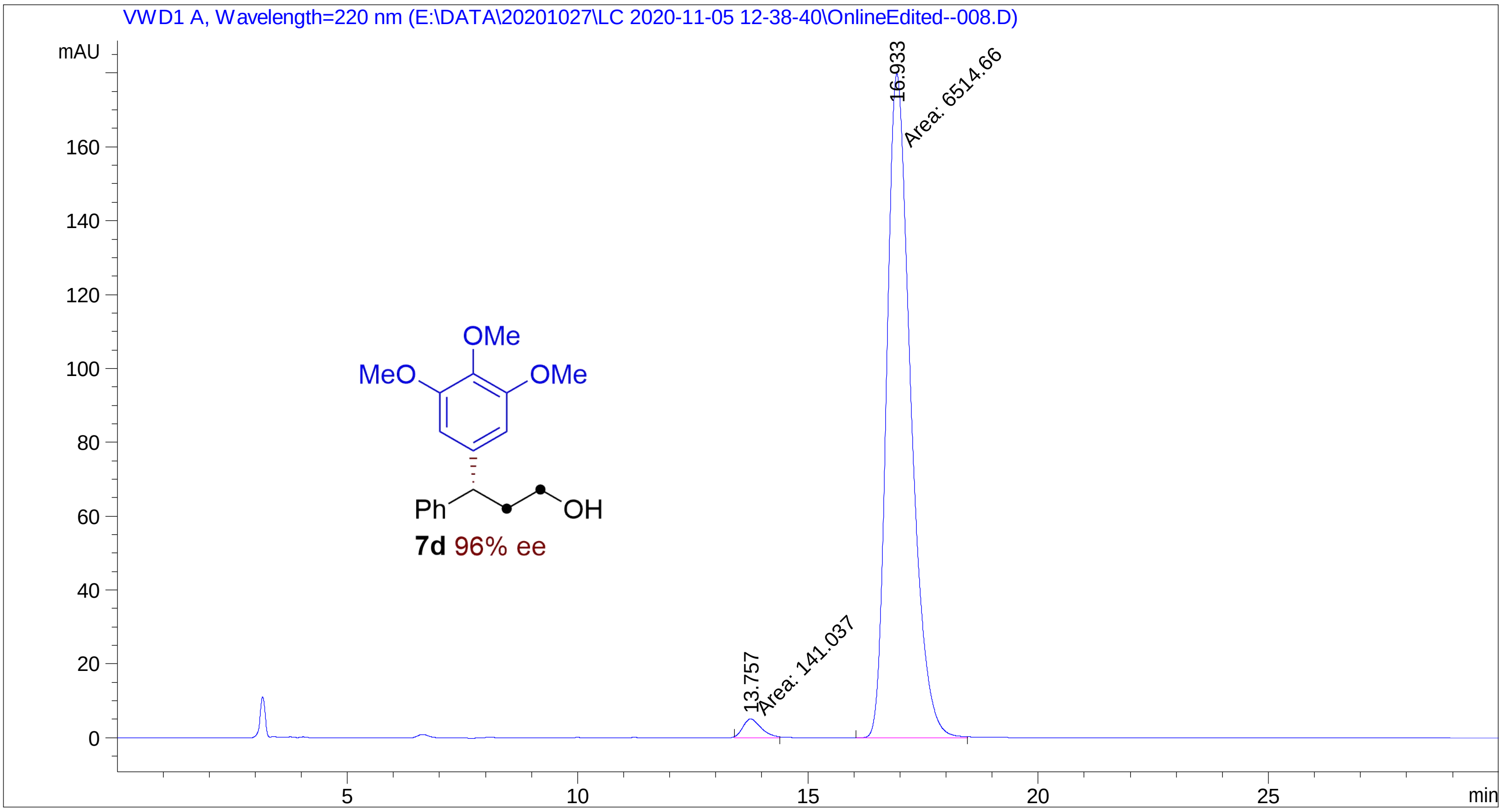

===
Area Percent Report
===

Sorted By : Signal
Multiplier : 1.0000
Dilution : 1.0000
Use Multiplier & Dilution Factor with ISTDs

Signal 1: VWD1 A, Wavelength=220 nm

Peak #	RetTime [min]	Type	Width [min]	Area [mAU*s]	Height [mAU]	Area %
1	13.757	FM	0.4611	141.03728	5.09765	2.1190
2	16.933	MF	0.6036	6514.66016	179.87325	97.8810

Totals : 6655.69743 184.97089

===

Data File E:\DATA\20201027\LC 2020-11-02 19-12-04\1AC-0501--008.D
Sample Name: YH-18-82-RAC

===

Acq. Operator : SYSTEM Seq. Line : 8
Acq. Instrument : HPLC1260 Location : P1-A3
Injection Date : 11/2/2020 11:39:29 PM Inj : 1
Inj Volume : 3.000 µl
Different Inj Volume from Sample Entry! Actual Inj Volume : 2.000 µl
Acq. Method : E:\DATA\20201027\LC 2020-11-02 19-12-04\20IPA_30_10_3.M
Last changed : 11/2/2020 7:12:04 PM by SYSTEM
Analysis Method : E:\DATA\20201027\LC 2020-11-02 19-12-04\20IPA_30_10_3.M (Sequence Method)
Last changed : 11/3/2020 8:15:39 AM by SYSTEM
(modified after loading)
Additional Info : Peak(s) manually integrated

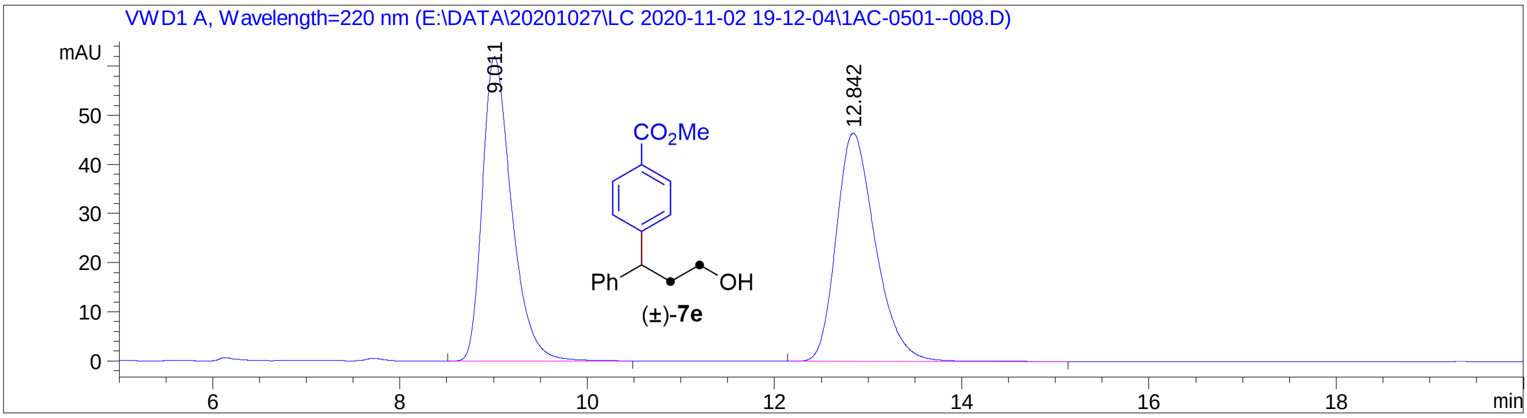

===

Area Percent Report

===

Sorted By : Signal
Multiplier : 1.0000
Dilution : 1.0000
Do not use Multiplier & Dilution Factor with ISTDs

Signal 1: VWD1 A, Wavelength=220 nm

Peak #	RetTime [min]	Type	Width [min]	Area [mAU*s]	Height [mAU]	Area %
1	9.011	BB	0.3291	1344.02332	61.95635	49.8827
2	12.842	BB	0.4452	1350.34583	46.45029	50.1173

Totals : 2694.36914 108.40664

===

*** End of Report ***

Data File E:\DATA\20201027\LC 2020-11-02 19-12-04\1AD-0601--009.D
Sample Name: YH-18-82-EE

===

Acq. Operator : SYSTEM Seq. Line : 9
Acq. Instrument : HPLC1260 Location : P1-A4
Injection Date : 11/3/2020 12:10:16 AM Inj : 1
Inj Volume : 3.000 µl
Acq. Method : E:\DATA\20201027\LC 2020-11-02 19-12-04\20IPA_30_10_3.M
Last changed : 11/2/2020 7:12:04 PM by SYSTEM
Analysis Method : E:\DATA\20201027\LC 2020-11-02 19-12-04\20IPA_30_10_3.M (Sequence Method)
Last changed : 11/3/2020 8:15:01 AM by SYSTEM
(modified after loading)
Additional Info : Peak(s) manually integrated

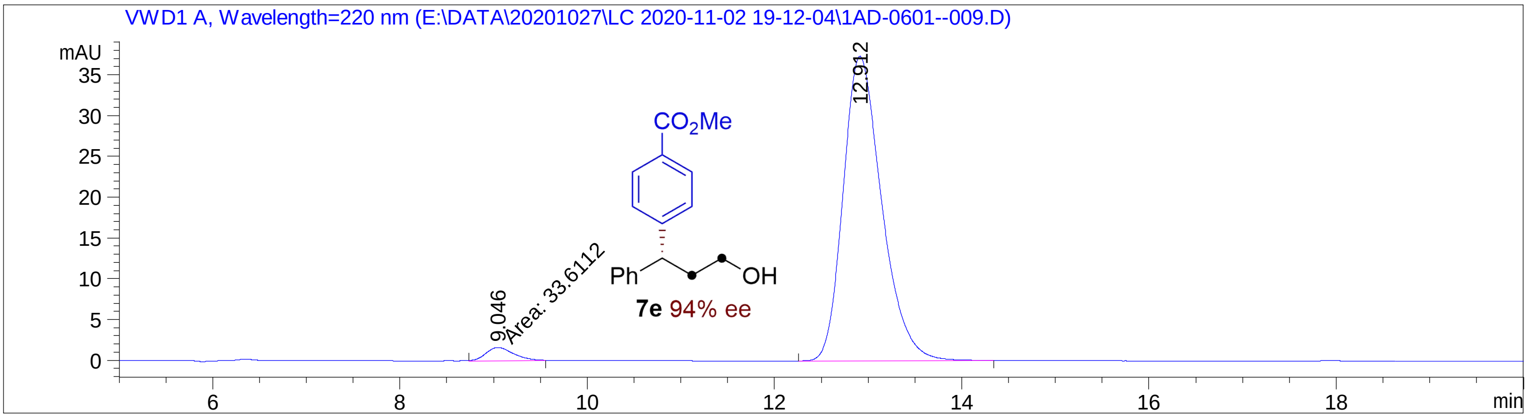

===

Area Percent Report

===

Sorted By : Signal
Multiplier : 1.0000
Dilution : 1.0000
Do not use Multiplier & Dilution Factor with ISTDs

Signal 1: VWD1 A, Wavelength=220 nm

Peak #	RetTime [min]	Type	Width [min]	Area [mAU*s]	Height [mAU]	Area %
1	9.046	MM	0.3439	33.61117	1.62896	3.0469
2	12.912	BB	0.4383	1069.49963	37.32675	96.9531

Totals : 1103.11081 38.95571

===

*** End of Report ***

===

Acq. Operator : SYSTEM Seq. Line : 63
Acq. Instrument : HPLC1260 Location : P1-A3
Injection Date : 11/6/2020 3:02:15 PM Inj : 2
Inj Volume : 3.000 µl
Different Inj Volume from Sample Entry! Actual Inj Volume : 1.000 µl
Acq. Method : E:\DATA\20201027\LC 2020-11-05 12-38-40\30IPA_30_0.8_3.M
Last changed : 11/6/2020 2:57:07 PM by SYSTEM
Analysis Method : E:\DATA\20201027\LC 2020-11-05 12-38-40\30IPA_30_0.8_3.M (Sequence Method)
Last changed : 11/6/2020 7:47:03 PM by SYSTEM
(modified after loading)
Additional Info : Peak(s) manually integrated

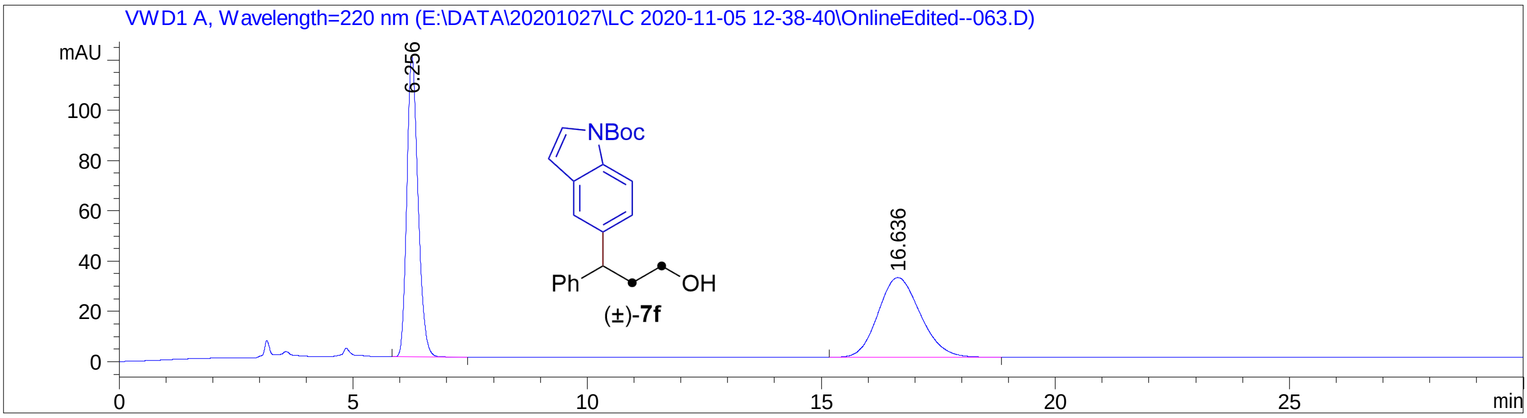

===

Area Percent Report

===

Sorted By : Signal
Multiplier : 1.0000
Dilution : 1.0000
Do not use Multiplier & Dilution Factor with ISTDs

Signal 1: VWD1 A, Wavelength=220 nm

Peak #	RetTime [min]	Type	Width [min]	Area [mAU*s]	Height [mAU]	Area %
1	6.256	BB	0.2551	1986.54639	119.43031	50.0496
2	16.636	BB	0.9632	1982.60645	31.67539	49.9504

Totals : 3969.15283 151.10570

===

*** End of Report ***

Data File E:\DATA\20201027\LC 2020-11-05 12-38-40\OnlineEdited--064.D
Sample Name: YH-18-89-EE

===

Acq. Operator   : SYSTEM   Seq. Line : 64
Acq. Instrument : HPLC1260   Location :   P1-A4
Injection Date : 11/6/2020 3:33:01 PM   Inj :   1
                                               Inj Volume : 3.000 µl
Different Inj Volume from Sample Entry! Actual Inj Volume : 2.000 µl
Acq. Method   : E:\DATA\20201027\LC 2020-11-05 12-38-40\30IPA_30_0.8_3.M
Last changed   : 11/6/2020 2:57:07 PM by SYSTEM
Analysis Method : E:\DATA\20201027\LC 2020-11-05 12-38-40\30IPA_30_0.8_3.M (Sequence Method)
Last changed   : 11/6/2020 7:47:03 PM by SYSTEM
                  (modified after loading)
Additional Info : Peak(s) manually integrated

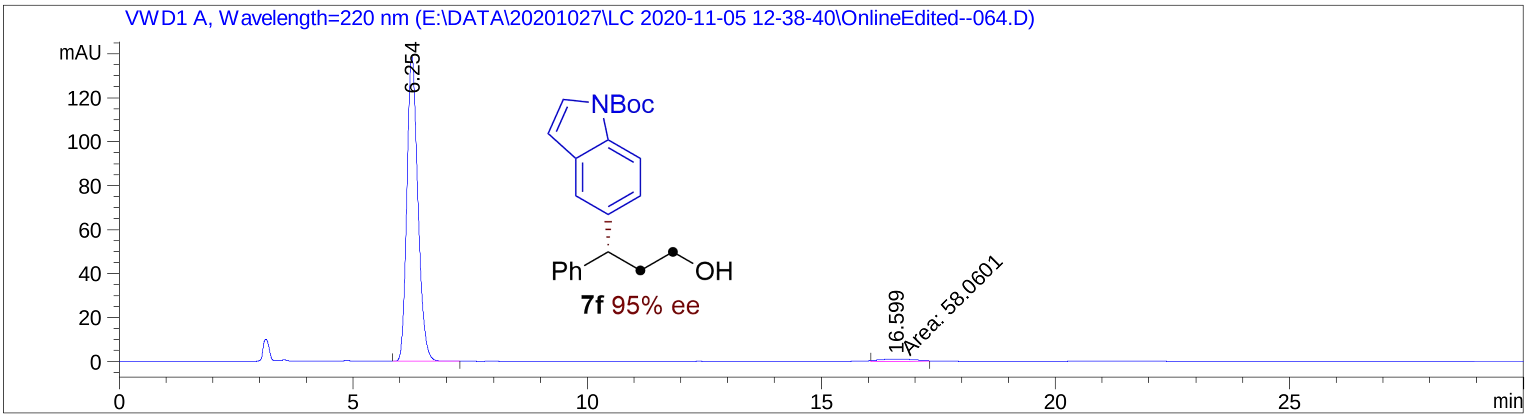

===

Area Percent Report

===

Sorted By   :   Signal
Multiplier   :   1.0000
Dilution   :   1.0000
Do not use Multiplier & Dilution Factor with ISTDs

Signal 1: VWD1 A, Wavelength=220 nm

Peak #	RetTime [min]	Type	Width [min]	Area [mAU*s]	Height [mAU]	Area %
1	6.254	BB	0.2525	2276.00708	138.65160	97.5125
2	16.599	MF	0.8830	58.06006	1.09585	2.4875

Totals :   2334.06714 139.74745

===

*** End of Report ***

Data File E:\DATA\20201027\LC 2020-11-05 12-38-40\OnlineEdited--012.D
Sample Name: YH-18-85-RAC

===

Acq. Operator : SYSTEM   Seq. Line : 12
Acq. Instrument : HPLC1260   Location : P1-B3
Injection Date : 11/5/2020 5:26:51 PM   Inj : 1
Inj Volume : 3.000 µl
Different Inj Volume from Sample Entry! Actual Inj Volume : 1.000 µl
Acq. Method : E:\DATA\20201027\LC 2020-11-05 12-38-40\20IPA_60_10_3.M
Last changed : 11/5/2020 6:11:04 PM by SYSTEM
(modified after loading)
Analysis Method : E:\DATA\20201027\LC 2020-11-05 12-38-40\20IPA_60_10_3.M (Sequence Method)
Last changed : 11/5/2020 7:05:40 PM by SYSTEM
(modified after loading)
Additional Info : Peak(s) manually integrated

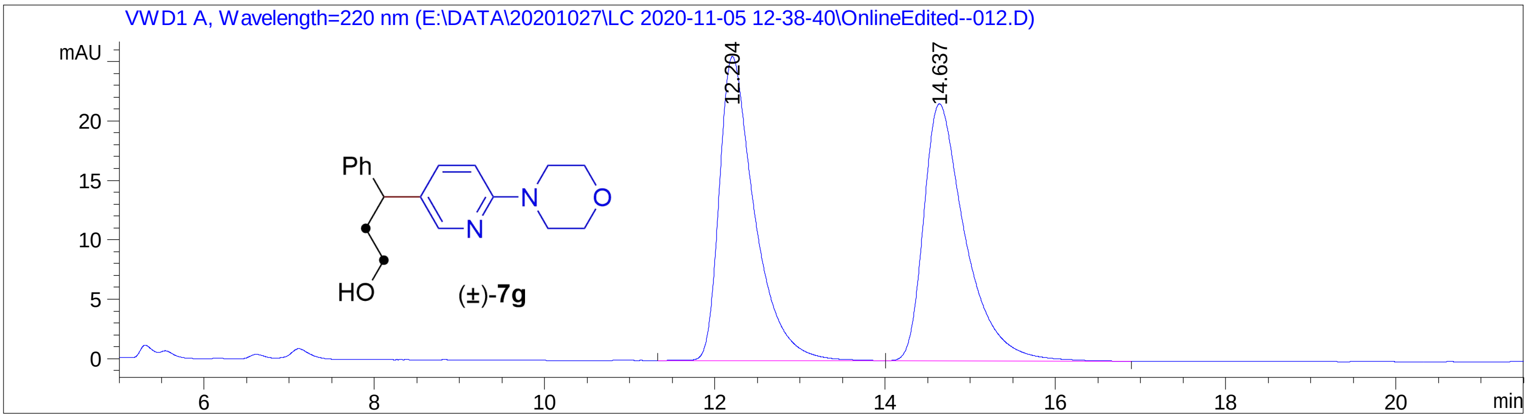

===

Area Percent Report

===

Sorted By : Signal
Multiplier : 1.0000
Dilution : 1.0000
Do not use Multiplier & Dilution Factor with ISTDs

Signal 1: VWD1 A, Wavelength=220 nm

Peak #	RetTime [min]	Type	Width [min]	Area [mAU*s]	Height [mAU]	Area %
1	12.204	BB	0.4263	739.40247	25.57660	50.0571
2	14.637	BB	0.5043	737.71674	21.60793	49.9429

Totals : 1477.11920 47.18453

===

*** End of Report ***

Data File E:\DATA\20201027\LC 2020-11-05 12-38-40\OnlineEdited--013.D
Sample Name: YH-18-85-EE

===

Acq. Operator : SYSTEM Seq. Line : 13
Acq. Instrument : HPLC1260 Location : P1-B4
Injection Date : 11/5/2020 6:11:50 PM Inj : 1
Inj Volume : 3.000 µl
Different Inj Volume from Sample Entry! Actual Inj Volume : 2.000 µl
Acq. Method : E:\DATA\20201027\LC 2020-11-05 12-38-40\20IPA_60_10_3.M
Last changed : 11/5/2020 6:11:04 PM by SYSTEM
Analysis Method : E:\DATA\20201027\LC 2020-11-05 12-38-40\20IPA_60_10_3.M (Sequence Method)
Last changed : 11/5/2020 7:06:07 PM by SYSTEM
(modified after loading)
Additional Info : Peak(s) manually integrated

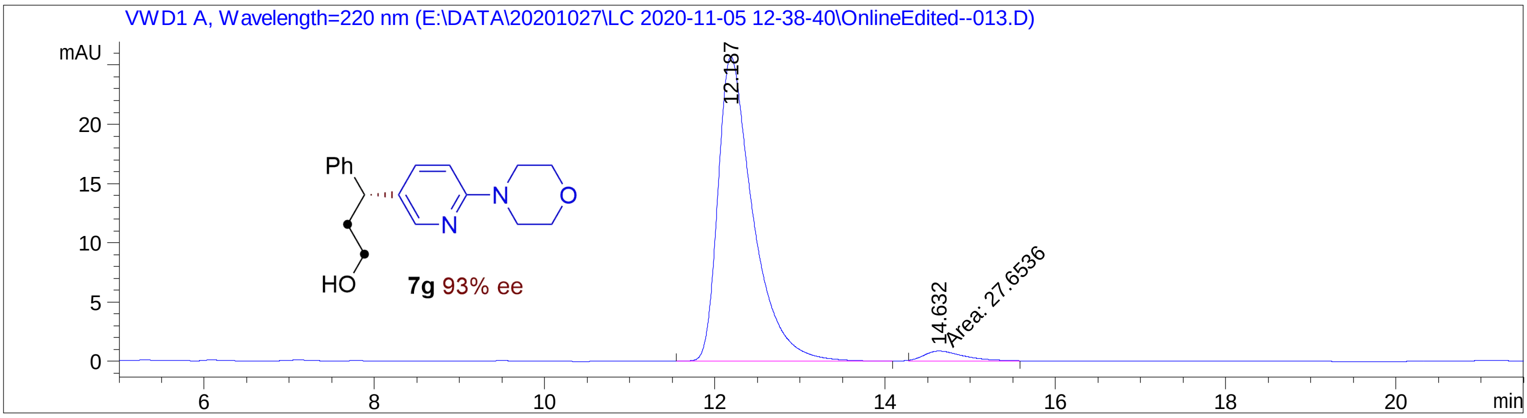

===

Area Percent Report

===

Sorted By : Signal
Multiplier : 1.0000
Dilution : 1.0000
Do not use Multiplier & Dilution Factor with ISTDs

Signal 1: VWD1 A, Wavelength=220 nm

Peak #	RetTime [min]	Type	Width [min]	Area [mAU*s]	Height [mAU]	Area %
1	12.187	BB	0.4210	735.36993	25.69036	96.3758
2	14.632	FM	0.5482	27.65362	8.40750e-1	3.6242

Totals : 763.02355 26.53111

===

*** End of Report ***

Data File E:\DATA\20201027\LC 2020-11-08 10-17-40\OnlineEdited--096.D
Sample Name: YH-18-93-RAC-AD

===

Acq. Operator : SYSTEM Seq. Line : 96
Acq. Instrument : HPLC1260 Location : P1-D5
Injection Date : 11/10/2020 9:18:26 AM Inj : 1
Inj Volume : 3.000 µl
Different Inj Volume from Sample Entry! Actual Inj Volume : 2.000 µl
Acq. Method : E:\DATA\20201027\LC 2020-11-08 10-17-40\20IPA_40_10_3.M
Last changed : 11/9/2020 3:44:13 PM by SYSTEM
Analysis Method : E:\DATA\20201027\LC 2020-11-08 10-17-40\20IPA_40_10_3.M (Sequence Method)
Last changed : 11/10/2020 10:59:07 AM by SYSTEM
(modified after loading)
Additional Info : Peak(s) manually integrated

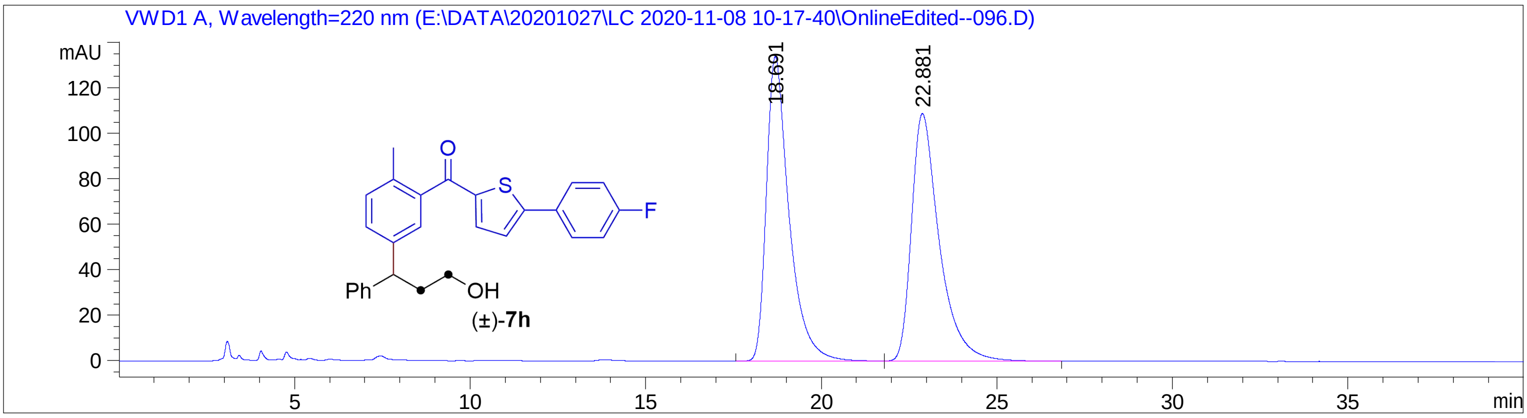

===
Area Percent Report
===

Sorted By : Signal
Multiplier : 1.0000
Dilution : 1.0000
Do not use Multiplier & Dilution Factor with ISTDs

Signal 1: VWD1 A, Wavelength=220 nm

Peak #	RetTime [min]	Type	Width [min]	Area [mAU*s]	Height [mAU]	Area %
1	18.691	BB	0.6668	6010.69727	133.88478	50.0093
2	22.881	BB	0.8188	6008.46924	108.88432	49.9907

Totals : 1.20192e4 242.76910

===
*** End of Report ***

Data File E:\DATA\20201027\LC 2020-11-08 10-17-40\OnlineEdited--097.D
Sample Name: YH-18-93-EE

===

Acq. Operator : SYSTEM Seq. Line : 97
Acq. Instrument : HPLC1260 Location : P1-D6
Injection Date : 11/10/2020 9:59:13 AM Inj : 1
Inj Volume : 3.000 µl
Different Inj Volume from Sample Entry! Actual Inj Volume : 2.000 µl
Acq. Method : E:\DATA\20201027\LC 2020-11-08 10-17-40\20IPA_40_10_3.M
Last changed : 11/9/2020 3:44:13 PM by SYSTEM
Analysis Method : E:\DATA\20201027\LC 2020-11-08 10-17-40\20IPA_40_10_3.M (Sequence Method)
Last changed : 11/10/2020 10:59:07 AM by SYSTEM
(modified after loading)
Additional Info : Peak(s) manually integrated

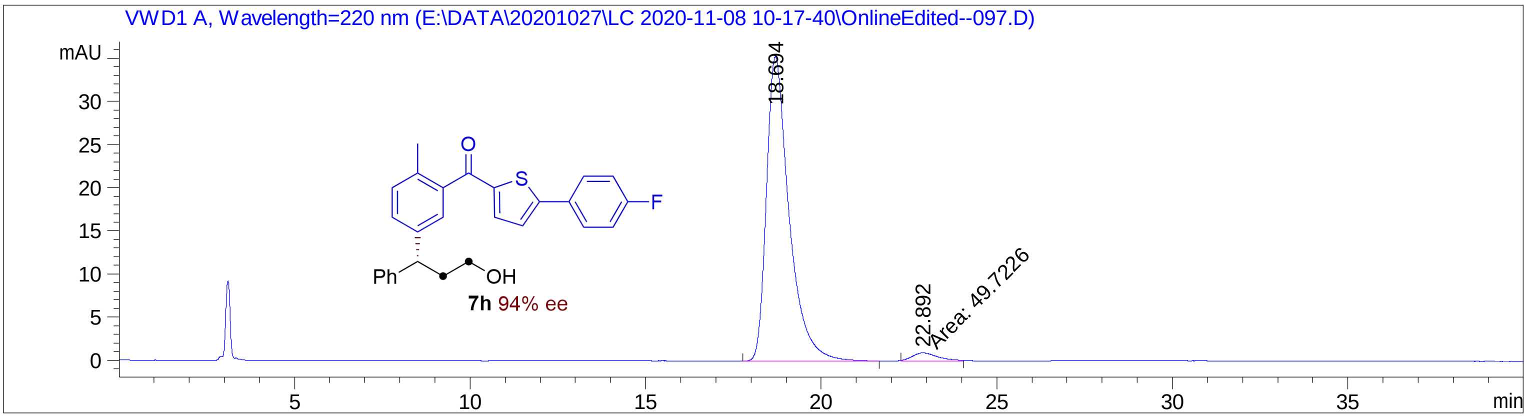

===

Area Percent Report

===

Sorted By : Signal
Multiplier : 1.0000
Dilution : 1.0000
Do not use Multiplier & Dilution Factor with ISTDs

Signal 1: VWD1 A, Wavelength=220 nm

Peak #	RetTime [min]	Type	Width [min]	Area [mAU*s]	Height [mAU]	Area %
1	18.694	BB	0.6654	1585.69543	35.28029	96.9596
2	22.892	FM	0.8705	49.72256	9.52012e-1	3.0404

Totals : 1635.41800 36.23230

===

*** End of Report ***

Data File E:\DATA\20201027\LC 2020-11-16 13-35-16\OnlineEdited--037.D
Sample Name: YH-18-101-RAC

===

Acq. Operator   : SYSTEM   Seq. Line : 37
Acq. Instrument : HPLC1260   Location :   P1-F1
Injection Date : 11/17/2020 3:35:54 AM   Inj :   1
                                               Inj Volume : 3.000 µl
Different Inj Volume from Sample Entry! Actual Inj Volume : 1.000 µl
Acq. Method   : E:\DATA\20201027\LC 2020-11-16 13-35-16\20IPA_50_10_4.M
Last changed   : 11/16/2020 8:05:16 PM by SYSTEM
Analysis Method : E:\DATA\20201027\LC 2020-11-16 13-35-16\20IPA_50_10_4.M (Sequence Method)
Last changed   : 1/20/2022 10:11:35 AM by SYSTEM
                  (modified after loading)
Additional Info : Peak(s) manually integrated

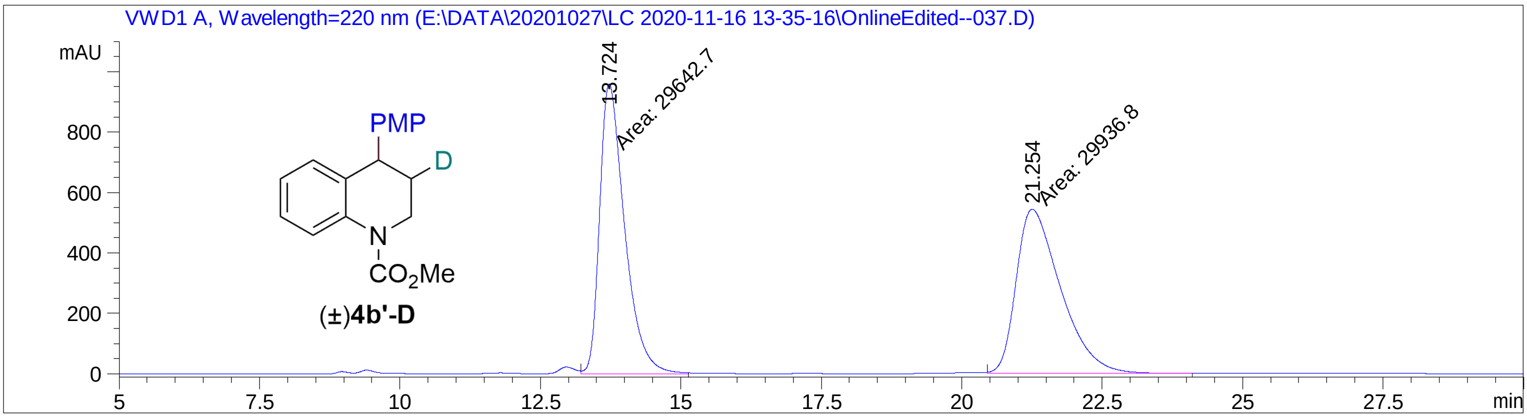

===

Area Percent Report

===

Sorted By   :   Signal
Multiplier   :   1.0000
Dilution   :   1.0000
Do not use Multiplier & Dilution Factor with ISTDs

Signal 1: VWD1 A, Wavelength=220 nm

Peak #	RetTime [min]	Type	Width [min]	Area [mAU*s]	Height [mAU]	Area %
1	13.724	MF	0.5154	2.96427e4	958.56610	49.7532
2	21.254	FM	0.9173	2.99368e4	543.94635	50.2468

Totals :   5.95795e4 1502.51245

===

*** End of Report ***

Data File E:\DATA\20201027\LC 2020-11-16 13-35-16\OnlineEdited--038.D
Sample Name: YH-18-101-1

===

Acq. Operator : SYSTEM   Seq. Line : 38
Acq. Instrument : HPLC1260   Location : P1-F2
Injection Date : 11/17/2020 4:26:42 AM   Inj : 1
Inj Volume : 3.000 µl
Acq. Method : E:\DATA\20201027\LC 2020-11-16 13-35-16\20IPA_50_10_4.M
Last changed : 11/16/2020 8:05:16 PM by SYSTEM
Analysis Method : E:\DATA\20201027\LC 2020-11-16 13-35-16\20IPA_50_10_4.M (Sequence Method)
Last changed : 1/20/2022 10:12:48 AM by SYSTEM
(modified after loading)
Additional Info : Peak(s) manually integrated

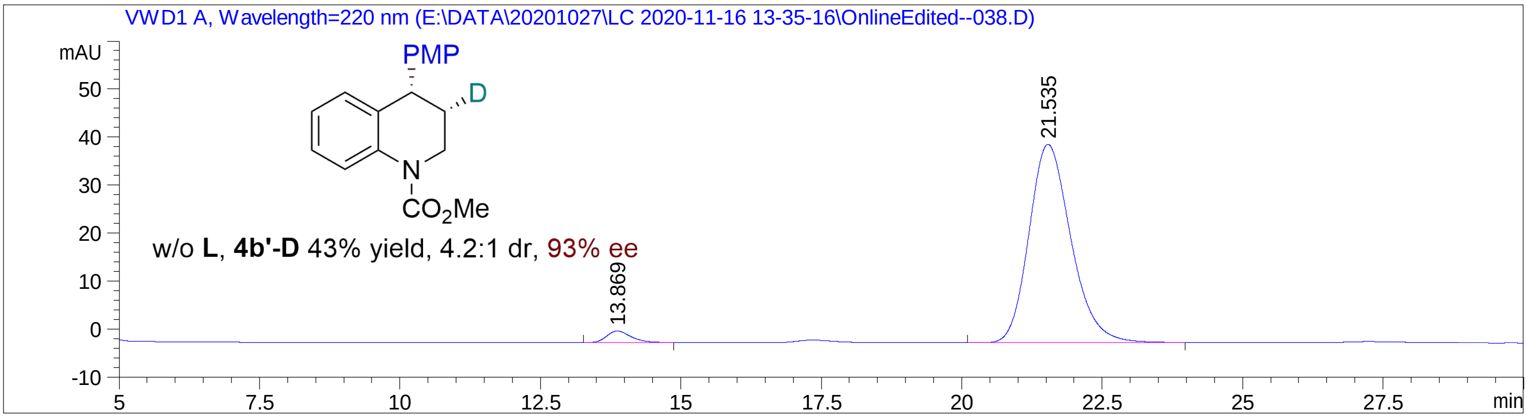

===

Area Percent Report

===

Sorted By : Signal
Multiplier : 1.0000
Dilution : 1.0000
Do not use Multiplier & Dilution Factor with ISTDs

Signal 1: VWD1 A, Wavelength=220 nm

Peak #	RetTime [min]	Type	Width [min]	Area [mAU*s]	Height [mAU]	Area %
1	13.869	BB	0.4527	72.94081	2.40570	3.2806
2	21.535	BB	0.7966	2150.43115	41.36130	96.7194

Totals : 2223.37196 43.76700

===

*** End of Report ***

Data File E:\DATA\20201027\LC 2020-11-18 10-58-39\OnlineEdited--035.D
Sample Name: YH-18-101-3-EE

===

Acq. Operator : SYSTEM   Seq. Line : 35
Acq. Instrument : HPLC1260   Location : P1-A2
Injection Date : 11/19/2020 2:18:25 AM   Inj : 1
Inj Volume : 3.000 µl
Different Inj Volume from Sample Entry! Actual Inj Volume : 5.000 µl
Acq. Method : E:\DATA\20201027\LC 2020-11-18 10-58-39\20IPA_50_10_3.M
Last changed : 11/18/2020 8:24:13 PM by SYSTEM
Analysis Method : E:\DATA\20201027\LC 2020-11-18 10-58-39\20IPA_50_10_3.M (Sequence Method)
Last changed : 1/20/2022 10:14:52 AM by SYSTEM
(modified after loading)
Additional Info : Peak(s) manually integrated

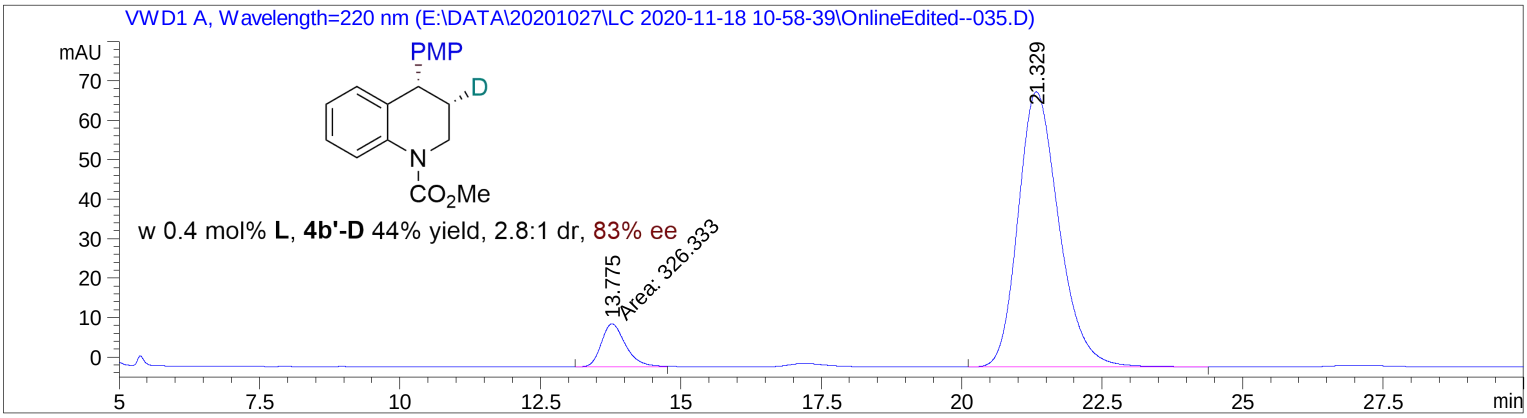

===

Area Percent Report

===

Sorted By : Signal
Multiplier : 1.0000
Dilution : 1.0000
Do not use Multiplier & Dilution Factor with ISTDs

Signal 1: VWD1 A, Wavelength=220 nm

Peak #	RetTime [min]	Type	Width [min]	Area [mAU*s]	Height [mAU]	Area %
1	13.775	MF	0.4988	326.33282	10.90301	8.4664
2	21.329	BB	0.7763	3528.12524	69.72406	91.5336

Totals : 3854.45807 80.62707

===

*** End of Report ***

Data File E:\DATA\20201027\LC 2020-11-16 13-35-16\OnlineEdited--039.D
Sample Name: YH-18-101-2

===

Acq. Operator : SYSTEM Seq. Line : 39
Acq. Instrument : HPLC1260 Location : P1-F3
Injection Date : 11/17/2020 5:17:32 AM Inj : 1
Inj Volume : 3.000 µl
Acq. Method : E:\DATA\20201027\LC 2020-11-16 13-35-16\20IPA_50_10_4.M
Last changed : 11/16/2020 8:05:16 PM by SYSTEM
Analysis Method : E:\DATA\20201027\LC 2020-11-16 13-35-16\20IPA_50_10_4.M (Sequence Method)
Last changed : 1/20/2022 10:13:33 AM by SYSTEM
(modified after loading)
Additional Info : Peak(s) manually integrated

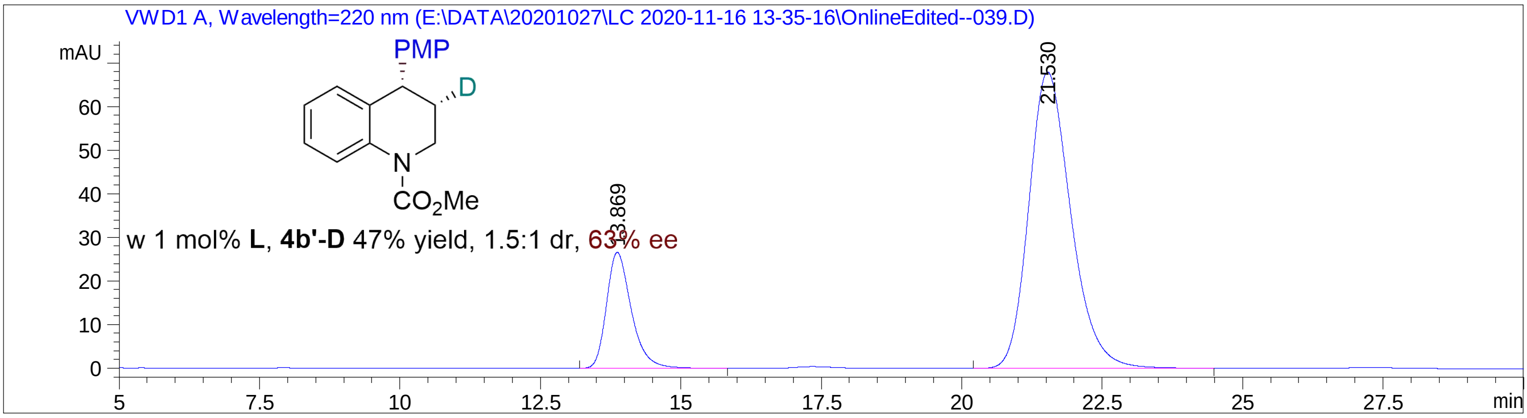

===

Area Percent Report

===

Sorted By : Signal
Multiplier : 1.0000
Dilution : 1.0000
Do not use Multiplier & Dilution Factor with ISTDs

Signal 1: VWD1 A, Wavelength=220 nm

Peak #	RetTime [min]	Type	Width [min]	Area [mAU*s]	Height [mAU]	Area %
1	13.869	BB	0.4684	815.76105	26.55793	18.7263
2	21.530	BB	0.7967	3540.47559	67.96634	81.2737

Totals : 4356.23663 94.52427

===

*** End of Report ***

3. Supplementary References

1. He, Y., Liu, C., Yu, L. & Zhu, S. Enantio- and regioselective NiH-catalyzed reductive hydroarylation of vinylarenes with aryl iodides. *Angew. Chem. Int. Ed.* **59**, 21530–21534 (2020).

2. Do, H.-Q., Chandrashekar, E. R. R. & Fu, G. C. Nickel/bis(oxazoline)-catalyzed asymmetric negishi arylations of racemic secondary benzylic electrophiles to generate enantioenriched 1,1-diarylalkanes. *J. Am. Chem. Soc.* **135**, 44, 16288–16291 (2013).

3. Frisch, M. J., Trucks, G. W., Schlegel, H. B., Scuseria, G. E., Robb, M. A., Cheeseman, J. R., Scalmani, G., Barone, V., Mennucci, B., Petersson, G. A., Nakatsuji, H., Caricato, M., Li, X., Hratchian, H. P., Izmaylov, A. F., Bloino, J., Zheng, G., Sonnenberg, J. L., Hada, M., Ehara, M., Toyota, K., Fukuda, R., Hasegawa, J., Ishida, M., Nakajima, T., Honda, Y., Kitao, O., Nakai, H., Vreven, T., Montgomery, J. A. Jr., Peralta, J. E., Ogliaro, F., Bearpark, M., Heyd, J. J., Brothers, E., Kudin, K. N., Staroverov, V. N., Keith, T., Kobayashi, R., Normand, J., Raghavachari, K., Rendell, A., Burant, J. C., Iyengar, S. S., Tomasi, J., Cossi, M., Rega, N., Millam, J. M., Klene, M., Knox, J. E., Cross, J. B., Bakken, V., Adamo, C., Jaramillo, J., Gomperts, R., Stratmann, R. E., Yazyev, O., Austin, A. J., Cammi, R., Pomelli, C., Ochterski, J. W., Martin, R. L., Morokuma, K., Zakrzewski, V. G., Voth, G. A., Salvador, P., Dannenberg, J. J., Dapprich, S., Daniels, A. D., Farkas, O., Foresman, J. B., Ortiz, J. V., Cioslowski, J. & Fox, D. J. Gaussian 09, Revision E.01. Gaussian, Inc., Wallingford CT, 2013.

4. Becke, A. D. Density-functional thermochemistry. III. the role of exact exchange. *J. Chem. Phys.* **98**, 5648–5652 (1993).

5. Lee, C., Yang, W. & Parr, R. G. Development of the Colle-Salvetti correlation-energy formula into a functional of the electron density. *Phys. Rev. B* **37**, 785–789 (1988).

6. Hay, P. J. & Wadt, W. R. Ab initio effective core potentials for molecular calculations - potentials for the transition-metal atoms Sc to Hg. *J. Chem. Phys.* **82**, 270–283 (1985).

7. Wadt, W. R. & Hay, P. J. Ab initio effective core potentials for molecular calculations - potentials for main group elements Na to Bi. *J. Chem. Phys.* **82**, 284–298 (1985).

8. Fukui, K. The path of chemical reactions - the IRC approach. *Acc. Chem. Res.* **14**, 363–368 (1981).

9. Zhao, Y. & Truhlar, D. G. The M06 suite of density functionals for main group thermochemistry, thermochemical kinetics, noncovalent interactions, excited states, and transition elements: two new functionals and systematic testing of four M06-class functionals and 12 other functionals. *Theor. Chem. Acc.* **120**, 215–241 (2008).

10. Dolg, M., Wedig, U., Stoll, H. & Preuss, H. Energy-adjusted ab initio pseudopotentials for the 1st-row transition-elements. *J. Chem. Phys.* **86**, 866-872 (1987).

11. Bergner, A., Dolg, M., Kuchle, W., Stoll, H. & Preuss, H. Ab-initio energy-adjusted pseudopotentials for elements of groups 13-17. *Mol. Phys.* **80**, 1431–1441 (1993).

12. Marenich, A. V., Cramer, C. J. & Truhlar, D. G. Universal solvation model based on solute electron density and on a continuum model of the solvent defined by the bulk dielectric constant and atomic surface tensions. *J. Phys. Chem. B* **113**, 6378–6396 (2009).

13. Rosenfarb, J., Huffman, H. L. Jr. & Caruso, J. A. Dielectric constants, viscosities, and related physical properties of several substituted liquid ureas at various temperatures. *J. Chem. Eng. Data* **21**, 150–153 (1976).
