# Supplementary material for: Regio- and enantioselective remote hydroarylation using a ligand-relay strategy
Source: Nat Commun. 2022 May 5;13:2471. doi: 10.1038/s41467-022-30006-2 (PMC9072428; doi:10.1038/s41467-022-30006-2)
Supplement: Supplementary file 3 — Description of Additional Supplementary Files [file 41467_2022_30006_MOESM3_ESM.pdf]

Description of Additional Supplementary files

File name: Supplementary Data 1

Description: Computational study of the asymmetric remote hydroarylation
